# A chiral phosphazane reagent strategy for the determination of enantiomeric excess of amines†

**DOI:** 10.1039/d2sc01692c

**Published:** 2022-04-25

**Authors:** Andrew J. Peel, Alexandros Terzopoulos, Rajesh B. Jethwa, Dipanjana Choudhury, Hao-Che Niu, Andrew D. Bond, Jonathan Slaughter, Dominic S. Wright

**Affiliations:** Yusuf Hamied Department of Chemistry, Cambridge University Lensfield Road Cambridge CB2 1EW UK dsw1000@cam.ac.uk; The Faraday Institution Quad One, Harwell Science and Innovation Campus Didcot UK

## Abstract

Methods for measuring enantiomeric excess (*ee*) of organic molecules by NMR spectroscopy provide rapid analysis using a standard technique that is readily available. Commonly this is accomplished by chiral derivatisation of the detector molecule (producing a chiral derivatisation agent, CDA), which is reacted with the mixture of enantiomers under investigation. However, these CDAs have almost exclusively been based on carbon frameworks, which are generally costly and/or difficult to prepare. In this work, a methodology based on the readily prepared inorganic cyclodiphosph(iii)azane CDA ClP(μ-N^*t*^Bu)_2_POBorn (Born = *endo*-(1*S*)-1,7,7-trimethylbicyclo[2.2.1]heptan-2-yl) is shown to be highly effective in the measurement of *ee*’s of chiral amines, involving *in situ* reaction of the chiral amines (R*NH_2_) with the P–Cl bond of the CDA followed by quaternization of the phosphorus framework with methyl iodide. This results in sharp ^31^P NMR signals with distinct chemical shift differences between the diastereomers that are formed, which can be used to obtain the *ee* directly by integration. Spectroscopic, X-ray structural and DFT studies suggest that the NMR chemical shift differences between diastereomers is steric in origin, with the sharpness of these signals resulting from conformational locking of the bornyl group relative to the P_2_N_2_ ring induced by the presence of the P(v)-bonded amino group (R*NH). This study showcases cheap inorganic phosphazane CDAs as simple alternatives to organic variants for the rapid determination of *ee*.

## Abstract 2

The simple inorganic cyclodiphosph(iii)azane chiral derivatisation agent ClP(μ-^*t*^BuN)_2_POBorn (Born = *endo*-(1*S*)-1,7,7-trimethylbicyclo[2.2.1]heptan-2-yl) is shown to be effective in the measurement of *ee*’s of chiral amines using ^31^P NMR spectroscopy.

## Graphical abstract

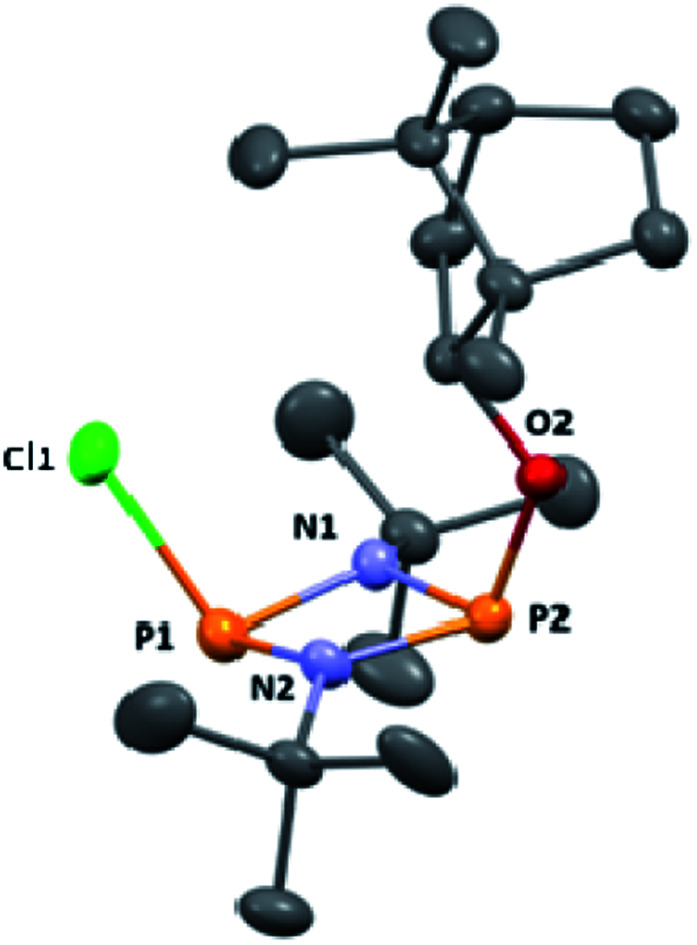

## Introduction

The creation of chiral molecules plays a major role in numerous chemical processes, from natural product synthesis to the design of new materials. Crucial to the search for efficient methods of producing enantiopure substances is the ability to detect and measure chiral composition.^1^ Many methods are established for the determination of the chiral purity of a sample, including optical rotation and circular dichroism,^2^ gas chromatography (GC) or liquid chromatography (HPLC)^3^ with a chiral stationary phase. Methods based on NMR spectroscopy have been studied extensively and remain at the forefront of this area due to the simplicity and availability of this technique and the potential for fast turnaround.^4^

In the last few decades, considerable effort has gone into developing new methodologies and reagents for the rapid and convenient determination of enantiomeric excess (*ee*) using NMR spectroscopy.^5^ Current methods involve the use of enantiomerically-pure compounds as chiral auxiliaries.^6^ A number are based on non-covalent interactions, including chiral solvating agents (CSAs),^7^ chiral lanthanide shift reagents,^8^ ion-pairing agents^5*c*^ and liquid crystals.^9^ However, chiral derivatisation agents (CDAs)^10^ that form a covalent bond with the chiral analyte are perhaps the most commonly used species for determining *ee*'s. Their popularity stems from the robustness of the covalent link between the auxiliary and analyte, greater freedom in solvent selection and their added potential for use in assignment of absolute configuration.^11^

**Scheme 1:**
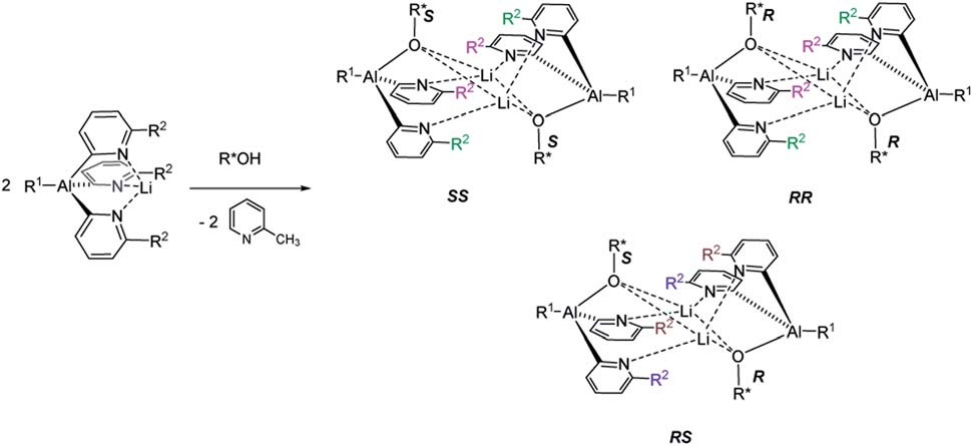
The reaction of an achiral pyridyl aluminate with a chiral alcohol, producing diastereoisomeric dimers. The result is the asymmetrisation of the reporter R^2^-groups of the pyridyl rings (marked in magenta, green, brown and purple), which can be detected by ^1^H NMR spectroscopy. R^1^, R^2^ = alkyl.

The reaction of an organic CDA with a chiral analyte typically creates a new C–X bond that has a relatively high bond dissociation energy and low-to-moderate bond polarity, *e*.*g*. C–N or C–O. The kinetic stability of these bonds is essential to prevent interchange of chiral components during analysis, and this has led to the dominance of organic chemistry in chiral derivatisation. So far, however, the potential of readily prepared inorganic CDAs has rarely been explored. Success in this endeavour hinges on selection of inorganic frameworks that can compete with the stability of classical carbon-based systems. A recent example is pyridyl aluminate reagents which can be used for the chiral discrimination of alcohols, but their mechanism of discrimination relies on the *in situ* formation of diastereomeric dimers, rather than monomeric diastereomers (Scheme 1).^12^ This, together with the substantial ionic character of organometallic CDAs of this type, leads to hydrolytic sensitivity and oxygen intolerance, which are major drawbacks to bench-top applications.

Molecules based on phosphorus–nitrogen skeletons are of interest in this regard because of the similarity between the C–C and P–N bond dissociation energies (346 kJ mol^−1^*vs.* 290 kJ mol^−1^, respectively) and their low polarity (Δ*χ*_P–N_ = 0.85).^13^ A particularly well-developed subset of P–N compounds are cyclodiphosph(iii)azanes [XP(NR)]_2_ (X = halogen, -OR, -N(H)R, -NR_2_, -R; R = organic group).^14^ Many different examples are accessible from the inexpensive precursor [ClPN^*t*^Bu]_2_, which has excellent stability and is easy to functionalise.^15,16^ Cyclodiphosphazane molecules have been investigated extensively as building blocks to inorganic macrocycles,^17–20^ as ligands in supramolecular coordination complexes^21^ and in MOFs.^22^ Furthermore, they have found applications in homogeneous catalysis,^23^ anti-tumour metallodrug development^24^ and anion recognition/transport.^25,26^ A role for P–N compounds in the chiral realm is emerging with a recent report on chiral amino-phosphonium salts which exhibit highly enantioselective recognition of chiral molecules, including carboxylic acids, amines and alcohols, through non-covalent interactions.^27^ However, in this case the ready detection of chirality and the measurement of *ee*'s was not possible. Inspired by this, and our earlier work on aluminates, we decided to explore the potential of cyclodiphosphazanes to build covalent chiral reagents that can be used to probe enantiomeric excess and absolute configuration using NMR spectroscopy.

## Results and discussion

### Establishing the methodology

**Scheme 2:**
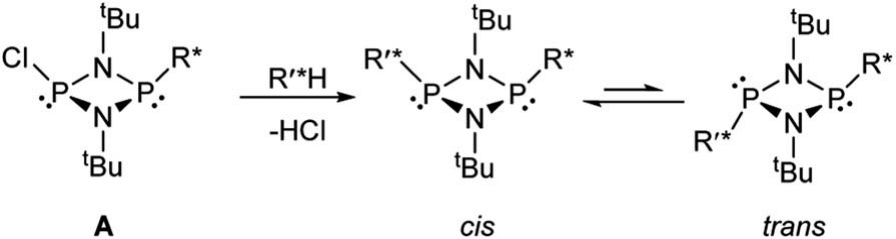
The concept of forming diastereomeric cyclodiphosph(iii)azanes, where R* is a chiral auxiliary and R′*H is a chiral analyte.

Based on the mechanism of chiral discrimination we had observed previously using pyridyl aluminate reagents, involving the formation of diastereomeric dimers (Scheme 1), we reasoned that the more rigid P_2_N_2_ ring units of cyclodiphosph(iii)azanes could form the basis of a new type of inorganic CDA. Our prototype CDA was of type A, shown in Scheme 2, in which one of the P atoms is bonded to a chiral auxiliary (R*) while the other has a reactive P–Cl bond. The highly reactive nature of this bond means that reaction with an acidic H-atom of a chiral analyte (R′*H) leads to a pair of diastereomers. An important feature of cyclodiphosphazanes of this type is the general thermodynamic preference for the *cis* isomer in the solution and solid states,^28^ so that the chiral groups R* and R′* should be in close proximity. This, it was thought, should induce the maximum effect on the chemical shifts of the *R* and *S* isomers of R′*.

**Scheme 3:**
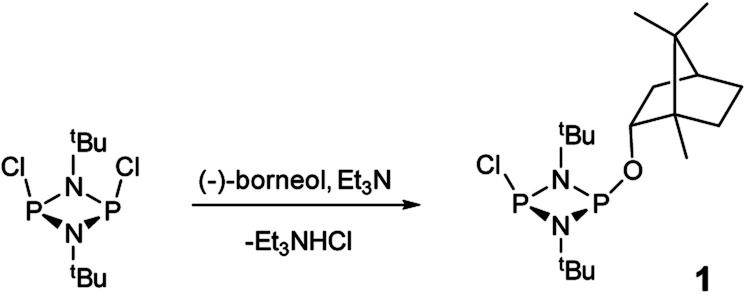
Synthesis of 1.

**Fig. 1:**
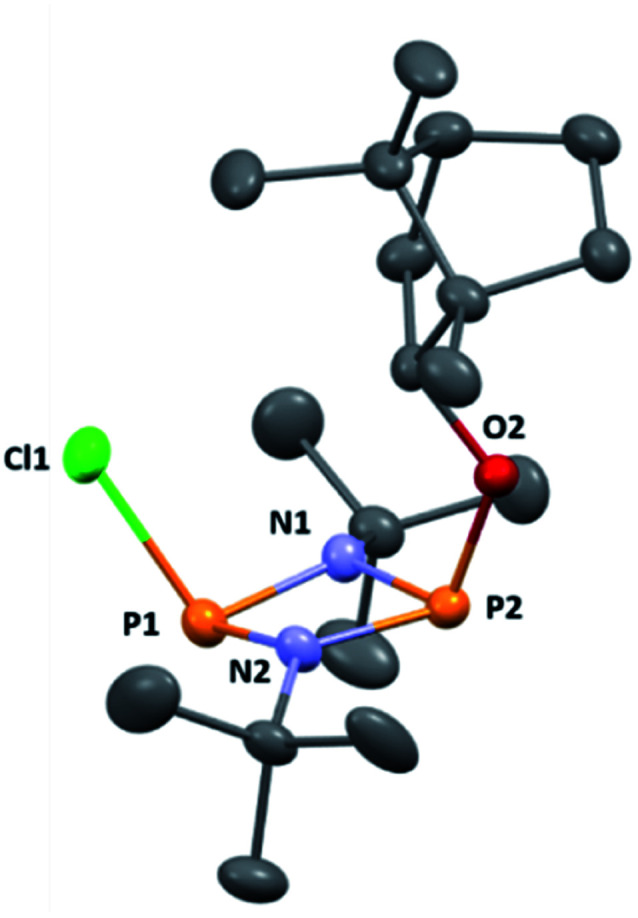
The molecular structure of 1 (ellipsoids at 30% probability) with H atoms omitted for clarity. One representative molecule from the asymmetric unit is depicted. The other molecule has essentially identical conformation, aside from rotation of one of the ^*t*^Bu groups. Selected bond lengths (Å) and angles (°): P1–N1 1.682(3), P1–N2 1.695(3), P2–N1 1.732(3), P2–N2 1.729(3), P1–Cl1 2.1619(3), P2–O1 1.612(2), P1–N1–P2 97.93(13), P1–N2–P2 97.52(13), N1–P1–N2 82.77(13), N1–P2–N2 80.36(12), N1–P1–Cl1 104.05(10), N2–P1–Cl1 104.14(10), N1–P2–O1 106.83(12), N2–P2–O1 106.80(12).

The reaction of [ClPN^*t*^Bu]_2_^16^ with the chiral, naturally occurring alcohol (−)-menthol has been reported previously,^23*c*^ however, the bicyclic alcohol *endo*-(−)-borneol was selected as a chiral auxiliary in the current study on the basis that it should exhibit less conformational freedom than menthol (and is nonetheless inexpensive). Furthermore, it has been reported that the formation of borneol derivatives, where O–H is replaced by O–X (X ≠ H) reduces the number of accessible conformers that can arise due to rotation about the O–X bond, and this was anticipated to be of benefit in developing a CDA.^29^ The prototype CDA was prepared by reacting [ClPN^*t*^Bu]_2_ with *endo*-(−)-borneol in a 1 : 1 ratio in THF in the presence of Et_3_N (Scheme 3). After trituration with Et_2_O and extraction into toluene, the crude product was recrystallised from *n*-pentane to give ClP(μ-N^*t*^Bu)_2_POBorn (Born = bornyl, *endo*-(1*S*)-1,7,7-trimethylbicyclo[2.2.1]heptan-2-yl) (1) in 50% yield (from two fractions). ^1^H NMR spectroscopy in C_6_D_6_ showed signals belonging to bornyl and ^*t*^Bu groups in a 1 : 2 ratio (see ESI Fig. S1†). Meanwhile, ^31^P{^1^H} NMR spectroscopy revealed two doublets, at *δ* 190.5 ppm and 140.0 ppm, which could be attributed to the –Cl and –OR substituted P atoms of a P_2_N_2_ ring, respectively. The observed inequivalence of the ^1^H NMR signals of the ^*t*^Bu groups within the P_2_N_2_ ring unit can be attributed to the effect of spatial orientation of the chiral axillary, though evidently the influence is small, with a separation of *δ* 0.02 ppm between these signals. Furthermore, this separation appears to be solvent dependent as only one singlet is observed in more polar CDCl_3_. X-ray diffraction revealed that 1 crystallises in the Sohncke space group *P*2_1_ (consistent with its enantiopurity), with two molecules in the asymmetric unit (Fig. 1). These molecules have essentially identical conformations, except for rotation of one ^*t*^Bu group. The P_2_N_2_ rings in 1 remain intact, as also observed in the only other solid-state structure of an alkoxy(chloro)phosphazane, Hyp_2_N_2_P_2_(Cl)OCH(CF_3_)_2_ (Hyp = (Me_3_Si)_3_Si).^30^ The P_2_N_2_ ring can be described as kite-shaped, which is a result of the different P–N bond lengths and angles at phosphorus associated with the OBorn (P–N_av_ = 1.689 Å; N–P–N_av_ = 80.3°) and Cl groups (P–N_av_ = 1.732 Å; N–P–N_av_ = 82.9°) that arise due to differences in electronegativity. These groups are arranged *cis* to one another in 1, as is seen in most other cyclodiphosphazanes in the solid state.^14^

NMR spectroscopy on batches of the crude product showed the only significant impurities to be starting material, the disubstituted product [^*t*^BuNPOBorn]_2_ (2), and phosphinic acid-type hydrolysis product H(O)P(μ-^*t*^BuN)_2_POBorn (3) (see ESI for full characterisation and Fig. S53 and S54† for molecular structures).^31,32^ Significantly, for preparative purposes, purification of crude 1 was not necessary.

**Scheme 4:**
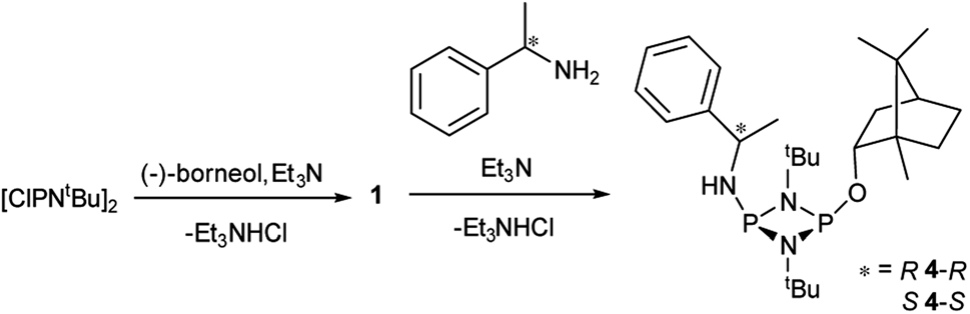
Synthesis of 4-*R* and 4-*S*.

Reactions of 1 with the amines (*R*)-1-phenylethylamine and (*S*)-1-phenylethylamine (both of which are benchmark chiral amine reagents in the literature) were undertaken to explore its potential as a CDA. *In situ*-prepared 1 was reacted in a 1 : 1 ratio with (*R*)- or (*S*)-1-phenylethylamine in the presence of excess Et_3_N (Scheme 4). Recrystallisation of the solid residues from toluene gave crystals of (*R*)-PhCH(Me)NHP(μ-N^*t*^Bu)_2_POBorn (4-*R*) or (*S*)-PhCH(Me)NHP(μ-N^*t*^Bu)_2_POBorn (4-*S*).

**Fig. 2:**
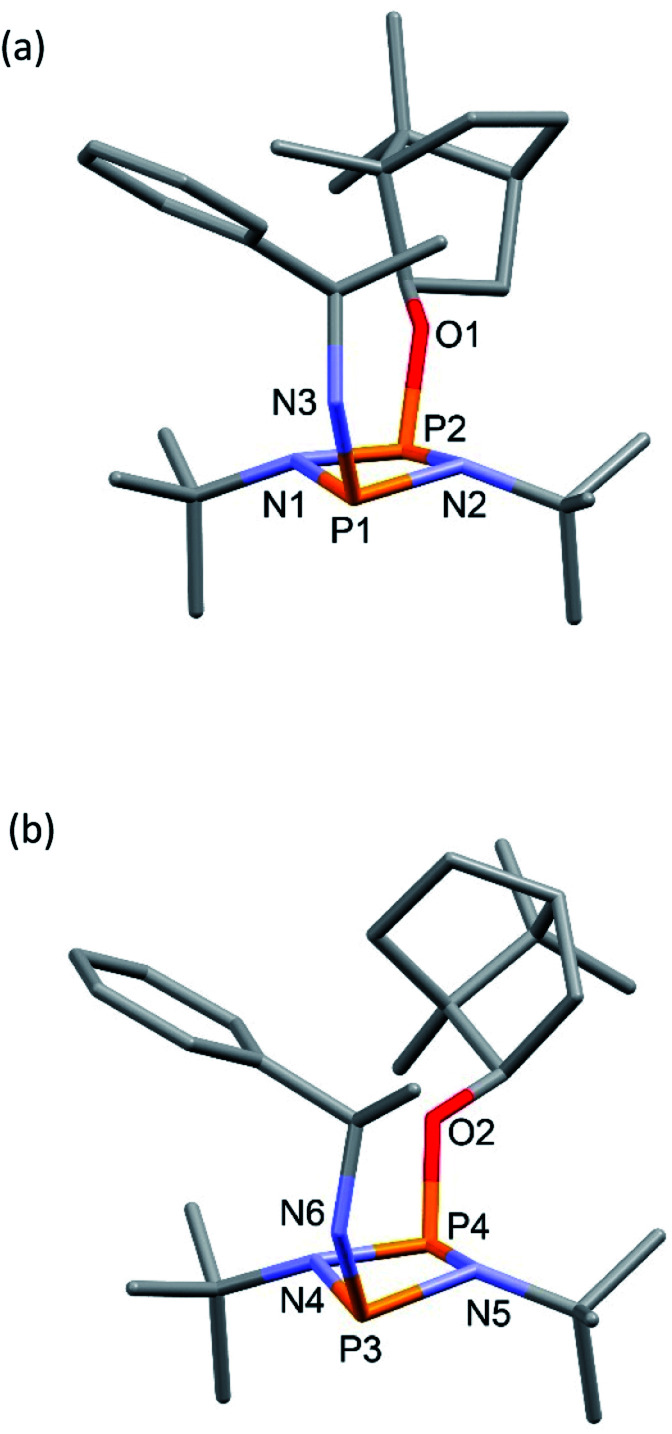
Two of the independent molecules in the asymmetric unit of 4-*R*, showing different conformations at the bornyl group (see ESI Fig. S55† for ellipsoid representations and selected metric parameters).

The solid-state structures of 4-*R* and 4-*S* are significantly different. In 4-*R* there are three independent molecules in the asymmetric unit (see ESI Fig. S55 and S56†). Two of these (one of which is shown in Fig. 2a) are similar, but distinct from the conformation of the third (Fig. 2b). The differences arise largely due to pivoting around the P–O and C–O bonds at the bornyl group (see ESI†). None of these arrangements allow for a good ‘fit’ of the groups on the chiral amine within the pocket offered by the *endo* face of the bornyl ring and their conformations are probably dictated by packing effects, suggesting relatively small energy differences between them.

**Fig. 3:**
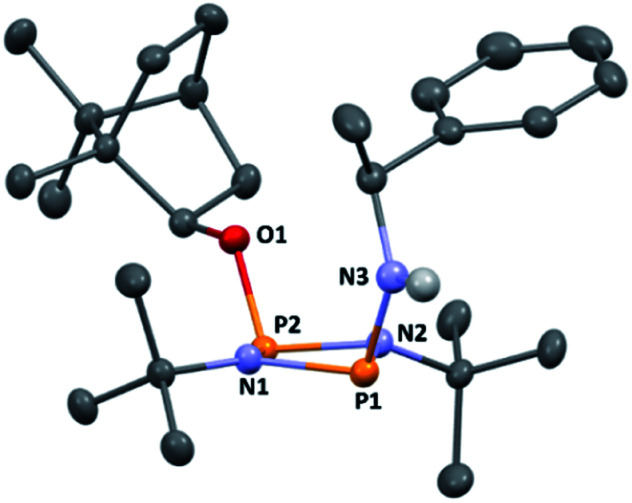
Molecular structure of 4-*S* (ellipsoids at 30% probability) with H atoms on C omitted for clarity. Selected bond lengths (Å) and angles (°): P1–N1 1.724(2), P1–N2 1.7373(19), P2–N1 1.6967(19), P2–N2 1.7136(19), P1–N 3 1.671(2), P2–O1 1.6492(16); P1–N1–P2 99.04(9), N1–P2–N2 81.48(9), P2–N2–P1 97.88(9), N2–P1–N1 80.03(9), N3–P1–N1 106.45(10), N3–P1–N2 106.71(10), O1–P2–N1 103.69(9) O1–P2–N2 104.35(9).

In contrast to 4-*R*, the solid-state structure of 4-*S* reveals a single unique molecule (in space group *P*2_1_2_1_2_1_) and the structural effect of inverting the stereochemistry at the α-carbon of the amine is unexpectedly large. While in 4-*R* the phenyl group projects away from the bornyl group, in 4-*S* it faces it, fitting inside the pocket on the *endo* face (Fig. 3). Some steric repulsion between the Ph- and ^*t*^Bu-groups is evident (also in 4-*R*), which involves ‘pushing down’ on the ^*t*^Bu group [the sums of the bond angles at N1 and N2 in 4-*S* are 359 and 347°, respectively].

**Fig. 4:**
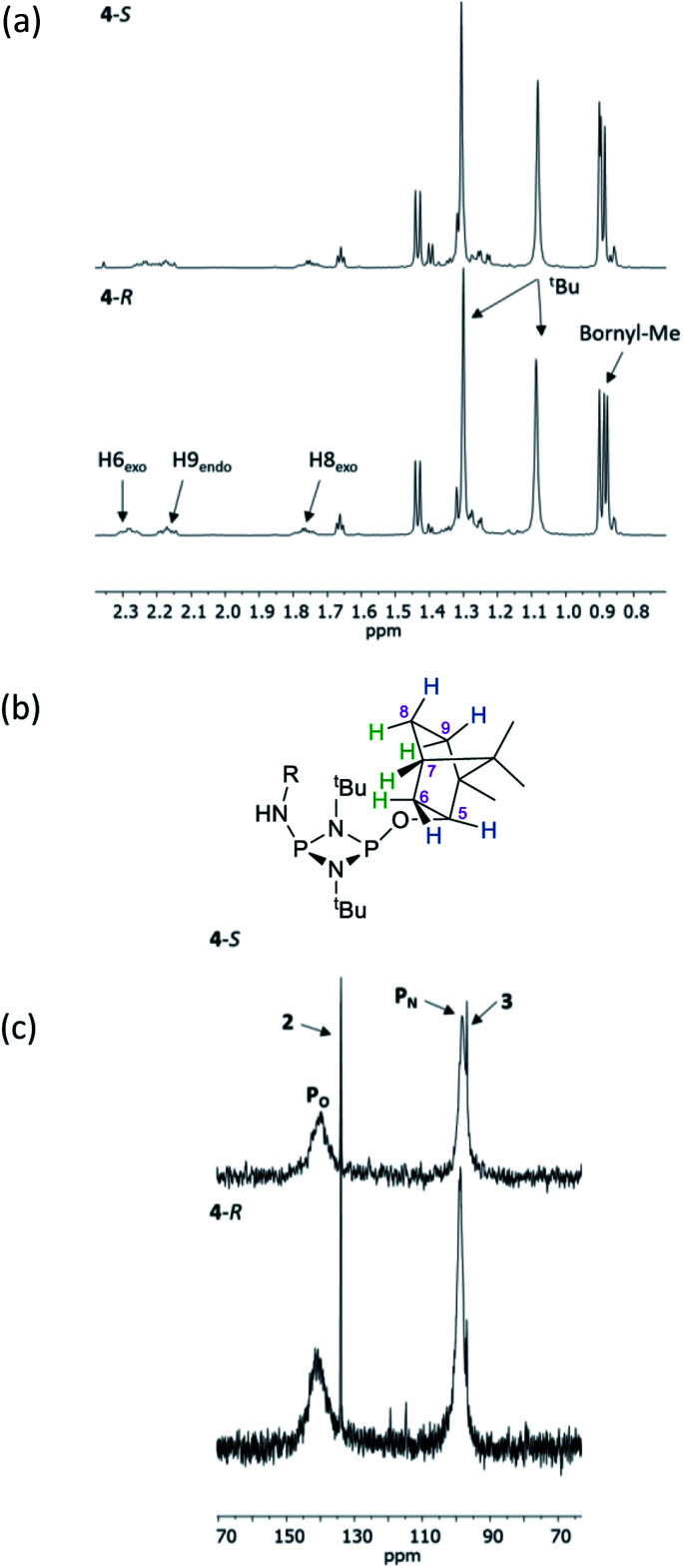
(a) Expansions of the ^1^H NMR spectra of 4-*R* and 4-*S* in CDCl_3_; (b) numbering scheme for bornyl ring hydrogens (blue = *exo*, green = *endo*) and (c) an expansion of the ^31^P{^1^H} NMR spectra of 4-*R* and 4-*S*.

The structural features seen in 4-*R* and 4-*S* would be expected to translate into measurable differences in the NMR spectra of these diastereomers if their solid-state structures were retained in solution. However, at a glance this does not appear to be the case. A comparison of the ^1^H NMR spectra of 4-*R* and 4-*S* in CDCl_3_ reveals few differences, with the notable exceptions being the methyl resonances of the bornyl group and of the two hydrogens of the bornyl framework in the region *δ* 2.3–2.1 ppm (see Fig. 4a and b). Regarding the chiral amine substituents, the resonance associated with the CH group, centred around *δ* 4.9 ppm, is broadened significantly in both compounds, while the N–H resonances, centred around *δ* 3.3 ppm, remain as sharp multiplets. Meanwhile, ^31^P{^1^H} NMR spectroscopy shows very broad peaks at *δ* 142 and 99 ppm in CDCl_3_ (Fig. 4c) that suggest that dynamic processes are occurring, such as *exo*–*endo* interconversion of the substituents (resulting from rotation of the P–O or P–N bonds; the resultant conformers denoted O_exo_/O_endo_ and N_exo_/N_endo_, respectively) and/or *cis*–*trans* isomerism.^23^ Cooling of 4-*R* in CD_2_Cl_2_ (down to 233 K) only resulted in sharpening of the ^31^P NMR resonances, and the fact that the chemical shifts remained in the region expected for the *cis* isomer (ESI Fig. S34b†) suggest that *endo-exo* interconversion is more likely to be responsible for the signal broadening at room temperature.^33^

**Fig. 5:**
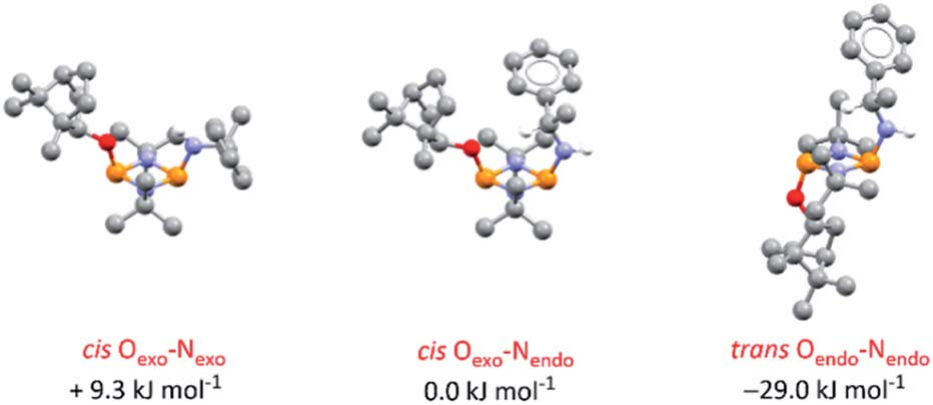
Optimised structures for the three most stable conformers of 4-*S* and their relative energies compared to the *cis* O_exo_–N_endo_ conformer (experimentally observed in the solid-state structure). Calculations are at the B3LYP/TZVP level and included an applied polarised continuum model (PCM) using the dielectric constant for chloroform to model the solution structure. Analogous isomers of 4-*R* are depicted in ESI Fig. S82 and S83.†

DFT calculations on 4-*S* in chloroform indicate that its various conformers possess energies in very close proximity (see ESI Table S6 and Fig. S74–S80†); the three most stable are shown in Fig. 5. Though NMR spectroscopy hints at the preference for *cis* isomers in solution, and the calculations reveal a small energy difference between the O_exo_–N_endo_ and O_exo_–N_exo_ forms of these, energetically accessible *trans* isomers are also predicted.^28*b*^ Hence, whilst we believe O_exo_–N_endo_–O_exo_–N_exo_ interconversion to be the dominant fluxional process, *cis*–*trans* isomerism cannot be ruled out. In contrast, calculations based on the experimentally observed *cis* O_exo_–N_endo_ isomer show that rotation of the chiral Ph(Me)CH group in 4-*S* about the C–N bond, switching the C–H bond from facing the P_2_N_2_ ring unit (as in the observed solid-state structure) to away from the P_2_N_2_ ring unit, is thermodynamically unfavourable (with an activation energy of around 45.6 kJ mol^−1^) (see ESI Table S7 and Fig. S81†).

**Scheme 5:**
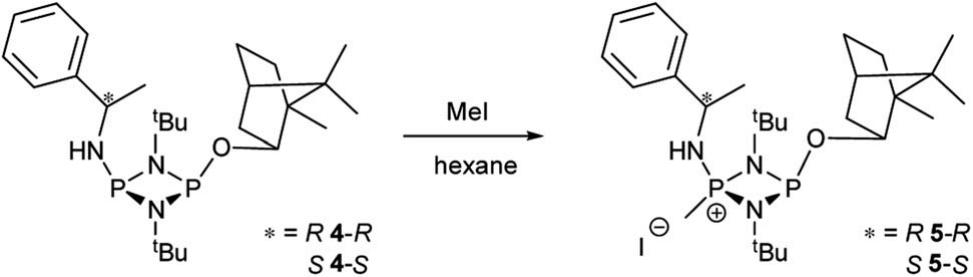
Selective reaction of 4-*R* and 4-*S* with methyl iodide.

**Fig. 6:**
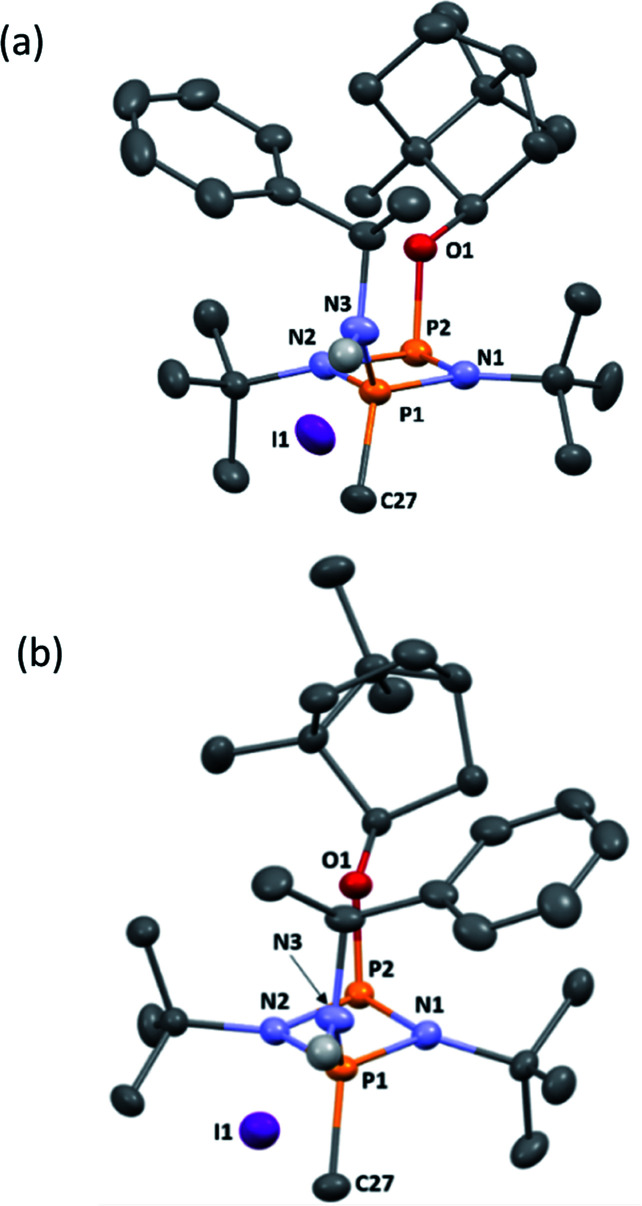
(a) The molecular structure of 5-*R* (ellipsoids at 30% probability) and with H atoms on C omitted for clarity. Selected bond lengths (Å) and angles (°): P1–N1 1.640(3), N1–P2 1.739(3), P2–N2 1.741(3), N2–P1 1.655(3), P1–N3 1.602(3), P2–O1 1.629(2), P1–C27 1.782(4); P1–N1–P2 97.11(16), N1–P2–N2 80.34(14), P2–N2–P1 96.48(15), N1–P1–N2 85.90(15), N1–P1–N3 116.25(17), N2–P1–N3 116.89(15), N1–P2–O1 103.91(14), N2–P2–O1 100.75(13), N1–P1–C27 116.07(17), N2–P1–C27 116.09(17), N3–P1–C27 105.41(18). (b) The molecular structure of 5-*S* (ellipsoids at 30% probability) and with H atoms on C omitted for clarity. Selected bond lengths (Å) and angles (°): P1–N1 1.646(3), N1–P2 1.755(2), P2–N2 1.731(3), N2–P1 1.640(3), P1–N3 1.600(3), P2–O1 1.627(2), P1–C27 1.781(3); P1–N2–P2 96.37(13), N1–P2–N2 80.06(12), P2–N2–P1 97.54(14), N2–P1–N2 86.03(13), N1–P1–N3 115.37(14), N2–P1–N3 117.40(14), N1–P2–O1 103.46(12), N2–P2–O1 100.54(11), N1–P1–C27 117.55(16), N2–P1–C27 114.83(16), N3–P1–C27 105.45(15).

While these results clearly show measurable differences in the ^1^H NMR spectra of the diastereomers formed from the reactions of 1, the small chemical shift separations encountered in a mixture would lead to overlap of signals and make it impossible to measure enatiomeric excess. Similarly, the operation of fluxional processes at ambient temperature eliminates ^31^P NMR spectroscopy as a viable spectroscopic alternative in these P(iii) dimers. Attempts to obtain sharp ^31^P NMR signals by formation of an internally coordinated lithiate (with Li envisaged to bridge the N and O atoms) were unsuccessful. However previous reports on the selective methylation of phosphorus atoms in cyclodiphosphazanes using methyl iodide hinted at another option.^21*b*,*c*^ Quaternisation has been observed to provide advantages for this type of P/N compound such as improved solubility in polar solvents and even air/water stability. Therefore we reasoned that quaternisation might also suppress the fluxional processes observed above and provide a further means of increasing the difference in the chemical shifts between the P atoms in diasteromeric pairs. 4-*R* and 4-*S* were therefore combined with excess methyl iodide in *n*-hexane which led to the formation of the salts [{(*R*)-PhCH(Me)NH}P(Me)(μ-N^*t*^Bu)_2_POBorn]I (5-*R*) and [{(*S*)-PhCH(Me)NH}P(Me)(μ-N^*t*^Bu)_2_POBorn]I (5-*S*) (Scheme 5, Fig. 6).

Following recrystallisation from THF, X-ray diffraction reveals that the amino/alkoxide substituents of 5-*R* and 5-*S* remain in a *cis* relationship with respect to each other and confirms that selective reaction of the more electron-rich amine-substituted P atom has occurred in both cases, leaving the O-substituted P and N atom of the chiral amino groups untouched. The orientation of the substituents of the amino groups in 5-*R* is identical to one of the molecules in 4-*R* (specifically the one depicted in Fig. 2b), and the conformation of 5-*S* is identical to 4-*S*. Not unexpectedly, the exocyclic P–N bond lengths associated with the P(v) centre in 5-*R* (1.602(3) Å) and 5-*S* (1.600(3) Å) are all shortened with respect to the corresponding P(iii) centre in 4-*R* (1.667(5) Å [average]) and 4-*S* (1.671(2) Å). While the asymmetry in these cycles does not allow for a direct comparison, it is evident that a shortening of the endocyclic P^V^–N bonds also occurs to within the range 1.640–1.655 Å (compared with 1.716–1.747 Å for 4-*R* and 4-*S*). Ostensibly, due to bond shortening there is an expansion of the N_chiral_–P–N_ring_ angles around the newly generated P^V^ centre (range 103.7–108.1° for 4-*R*/4-*S*; 115.3–117.4° for 5-*R*/5-S). Nonetheless, the effect on the exocyclic N⋯O distance between the amino-N and alkoxide-O atoms is marginal (being *ca.* 4 Å in every case). Both 5-*R* and 5-*S* show near linear interactions between the N–H hydrogen atoms and the nearby iodide ions in their lattices, at distances comparable to those reported in the literature for N–H⋯I contacts (2.65 Å for 5-*R*, 2.68 Å for 5-*S* based on calculated positions; literature value 2.69 Å).^34^ The most important conclusion from this structural analysis is that despite clear changes in bonding within the P_2_N_2_ ring, interactions between the exocyclic substituents are not significantly affected by methylation.

**Fig. 7:**
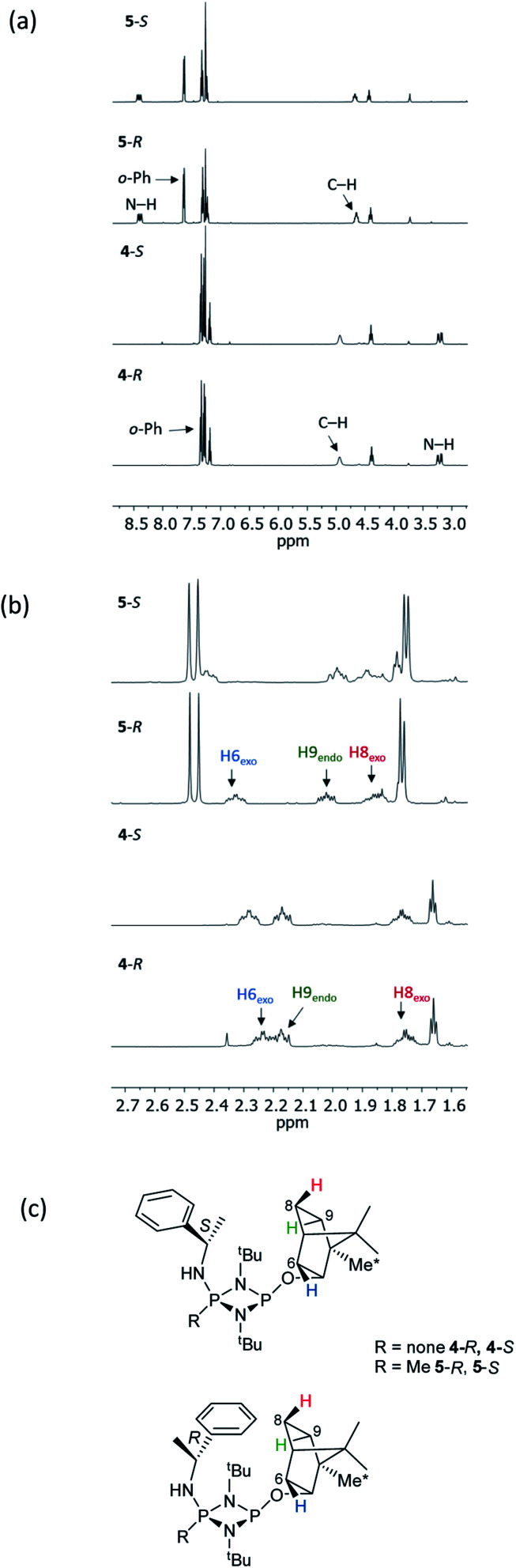
Expansions of the ^1^H NMR spectra of 4-*R*, 4-*S*, 5-*R* and 5-*S*, showing (a) the signals of selected bornyl ring hydrogens and (b) ^*t*^Bu groups/Me groups as indicated in (c).

NMR spectroscopy reveals much more pronounced differences between parent molecules 4-*R*/4-*S* and iodide salts 5-*R*/5-*S*. In CDCl_3_, the most noticeable change is the shift of the N–H resonance, from *ca*. *δ* 3.2 ppm to *δ* 8.4 ppm, which is associated with incorporation of the P(v) centre (Fig. 7a).^24*a*^ The increased electron withdrawing character of this P atom is also evident from deshielding of the Ph- and Me-group hydrogens. Meanwhile, the CH resonances of the Ph(Me)CH groups, formerly broad singlets centred at *δ* 4.94 ppm in 4-*R*/4-*S*, transform into clearly defined multiplets, at *δ* 4.65 and 4.68 ppm for 5-*R* and 5-*S*, respectively. This was the first indication that the dynamic processes evident in solutions of 4-*R* and 4-*S* are suppressed by methylation in 5-*R* and 5-*S*. This was reasoned to arise from enhanced N(p) → P–N(σ*) donation upon oxidation of the P_N_(iii) centre in 4-*R*/4-*S* to P_N_(v) increasing the barrier to P–N bond rotation. Possibly as result of this, the separation of the inequivalent ^1^H NMR resonances of the ^*t*^Bu groups is amplified approximately twofold moving from 4-*R*/4-*S* to 5-*R*/5-*S*, as the phenyl ring is more rigidly located with respect to each ^*t*^Bu group. The bornyl ring hydrogens still appear at slightly different positions in the new diastereomers but the differences between 5-*R* and 5-*S* are no larger than between 4-*R* and 4-*S* (Fig. 7b). On the other hand, the bornyl Me-peaks show a more distinctive pattern, whereby Me* (Fig. 7c) appears more deshielded in 5-*S* (*δ* 0.96 ppm) than in 5-*R* (*δ* 0.92 ppm), consistent with Me* being positioned further from the shielding Ph-ring.

**Fig. 8:**
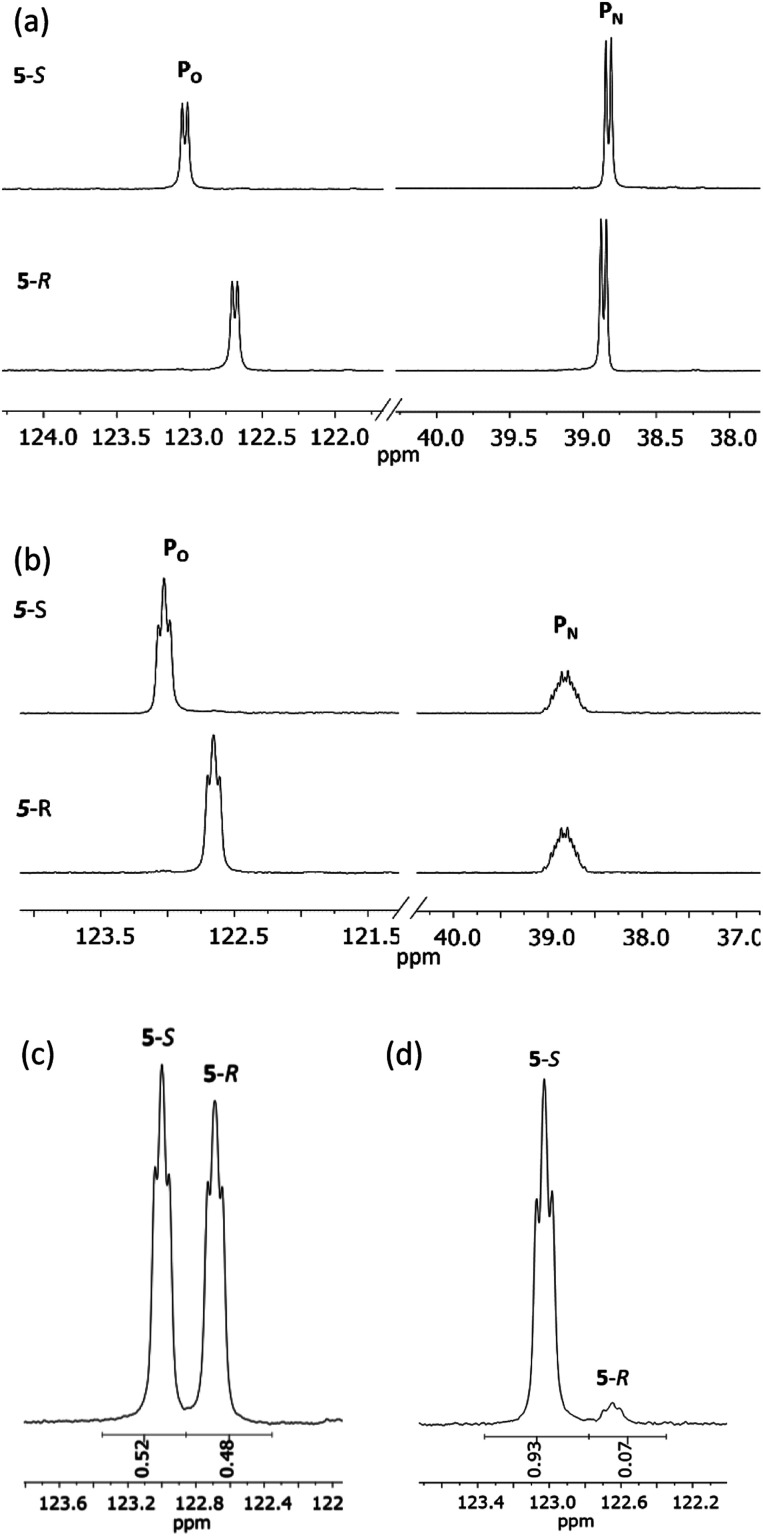
Expanded regions of (a) ^31^P{^1^H} NMR spectra of 5-*R* and 5-*S*; (b) ^31^P NMR spectra of 5-*R* and 5-*S*; (c) an aliquot from a reaction mixture containing roughly equal portions of (*R*)- and (*S*)-1-phenylethylamine and (d) the product of this reaction following recrystallization.

^31^P{^1^H} NMR spectroscopy provides more concrete evidence of the change in the dynamics by showing the disappearance of the broad resonances for 4-*R*/4-*S* (*ca*. *δ* 140 and 100 ppm), which are replaced by sharp doublets, at *δ* 122.7 and 38.8 ppm for 5-*R*, and *δ* 123.0 and 38.8 ppm for 5-*S* (Fig. 8a). The significance of this is that the *δ* 0.3 ppm separation in chemical shift of the O-bound P atom is sufficient to avoid overlap (see Fig. 8b), in turn allowing for accurate integration of the ^31^P NMR peaks. This provided the necessary proof of principle for the use of cyclodiphosphazanes as CDAs since integration can be used directly to determine the *ee*. This is described in more detail later in this section. In the meantime, it is worth noting that the physical characteristics of diastereomers 5-*R* and 5-*S* are substantially different. For example, the decomposition temperature of 5-*S* is some 20 °C higher than 5-*R*. More importantly, from the reaction of the roughly 50 : 50 mixture of (*S*)- and (*R*)-1-phenylethylamine with 1 followed by *in situ* quaternisation with methyl iodide, 5-*S* is selectively crystallised, as verified by X-ray crystallography and analysis of the bulk crystalline material by ^31^P NMR spectroscopy (Fig. 8c and d). The ratio of 5-*S* and 5-*R* was found to be 93 : 7. The tendency of these iodide salts to crystallise presents a promising avenue for further exploration, since the presence of a heavy atom allows reliable assignment of absolute structure—essential in assessing a reaction's stereoselectivity.

**Fig. 9:**
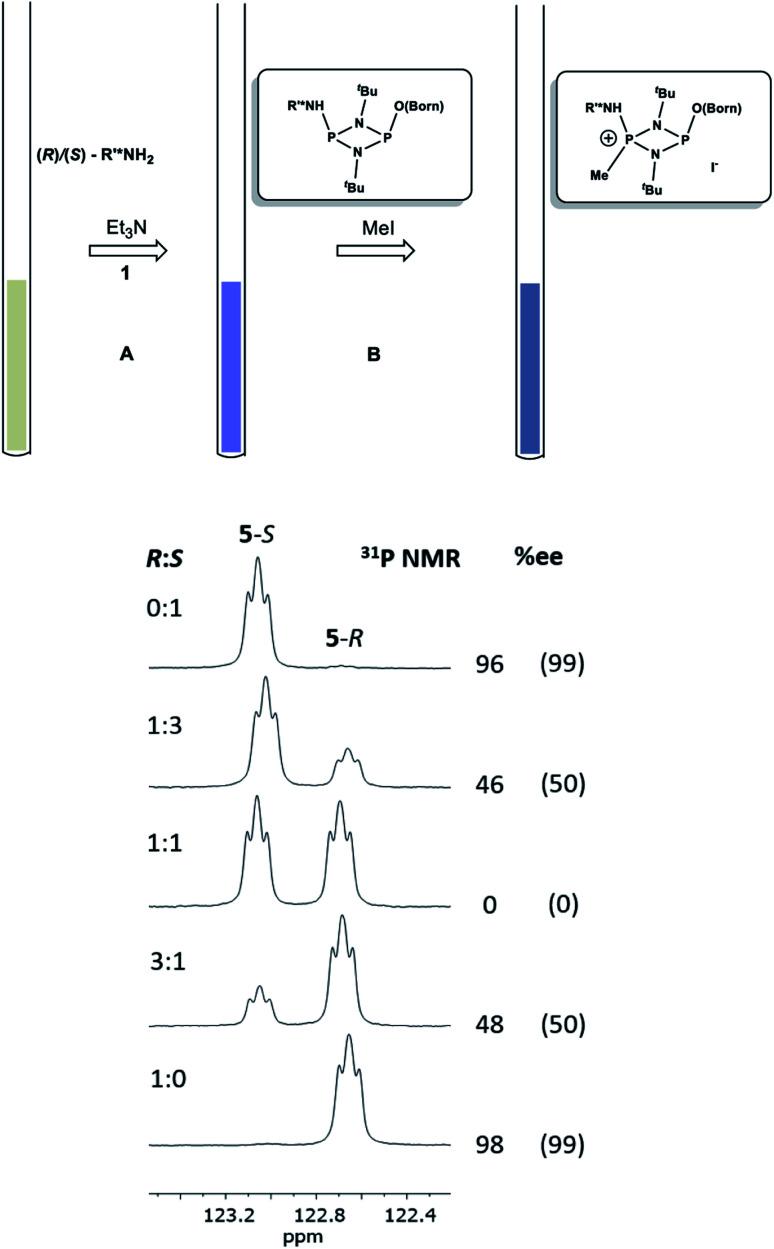
Method for the *in situ* derivatisation and analysis of a non-enantiopure amine (in this case 1-phenylethylamine) using 1. Conditions: (A) Et_3_N (2 eq.), THF, 4 h at RT; (B) methyl iodide (3 eq.), THF, 3 h at RT. The reaction was stopped after stages (A) and (B) by removal of the volatiles. Below are shown expansions of the ^31^P NMR spectra for scalemic mixtures of 1-phenylethylamine. The determined %*ee*'s are given on the right of the spectra, and their predicted *ee*'s based on the ratios of the enantiomers added are shown in parentheses. The *ee*'s predicted for the pure enantiomers are those stated by the supplier (Acros Organics).

Having established the ability to distinguish 5-*R* and 5-*S* in their isolated form using ^31^P NMR spectroscopy, we developed an *in situ* methodology that can be used to measure enantiomeric excess in mixtures of commercial (*R*)/(*S*)-1-phenylethylamine. Predetermined mixtures of these amines on the 50 μmol scale (yielding sufficient product for NMR spectroscopy) were treated *in situ* with 1 and then methyl iodide (Fig. 9, top). In all cases, some hydrolysis of 1 was unavoidable (producing 3), however, residual 1 in the reaction mixture indicated that sufficient CDA was available to consume all of the amine added. The results of ^31^P NMR spectroscopy (Fig. 9, bottom) show that the *in situ* method works well across a range of chiral compositions, with the main error in the measurement of the *ee* being due to the sensitivity of ^31^P NMR as well as the presence of trace amounts of either isomer in the commercially-supplied samples of the *R*- and *S*-isomers.

### Solution studies and detection limit

Although we had established the use of 1 as a CDA, there were two important questions which we wanted to address, namely, (i) the relationship between solid-state and solution structure, and (ii) the detection limit of our methodology.

The first of these was explored (somewhat fortuitously) using the related amine 1-phenylpropylamine in which the methyl group of 1-phenylethylamine is replaced by an ethyl group, offering a slightly more sterically hindered chiral centre. Though the intermediate phosphazane analogous to 4-*R*/4-*S* could not be isolated in pure form from the reactions of *R*- and (*S*)-1-phenylpropylamine with 1, *in situ* reactions of them with methyl iodide proceeded smoothly to give diffraction-quality crystals of [{(*R*)-PhCH(Et)NH}P(Me)(μ-N^*t*^Bu)_2_POBorn]I (6-*R*) and [{(*S*)-PhCH(Et)NH}P(Me)(μ-N^*t*^Bu)_2_POBorn]I (6-*S*). X-ray crystallography demonstrated that 6-*R*/6-*S* retain effectively identical conformations to the related salts 5-*R*/5-*S* (see ESI Fig. S57 and S58†), with the aromatic group sitting above opposite ^*t*^Bu groups within the pair of diastereomers and hence the C–H group pointing towards the P_2_N_2_ ring units. Bearing in mind this similarity it is not surprising that many spectroscopic features found in 5-*R*/5-*S* are also seen in the NMR spectra of 6-*R*/6-*S* in CDCl_3_. Most importantly, two well-separated low-field multiplets are observed in their ^31^P NMR spectra, at *δ* 122.6 ppm for 6-*R* and *δ* 123.1 ppm for 6-*S*, showing that assessment of the *ee* will be possible here also.

**Fig. 10:**
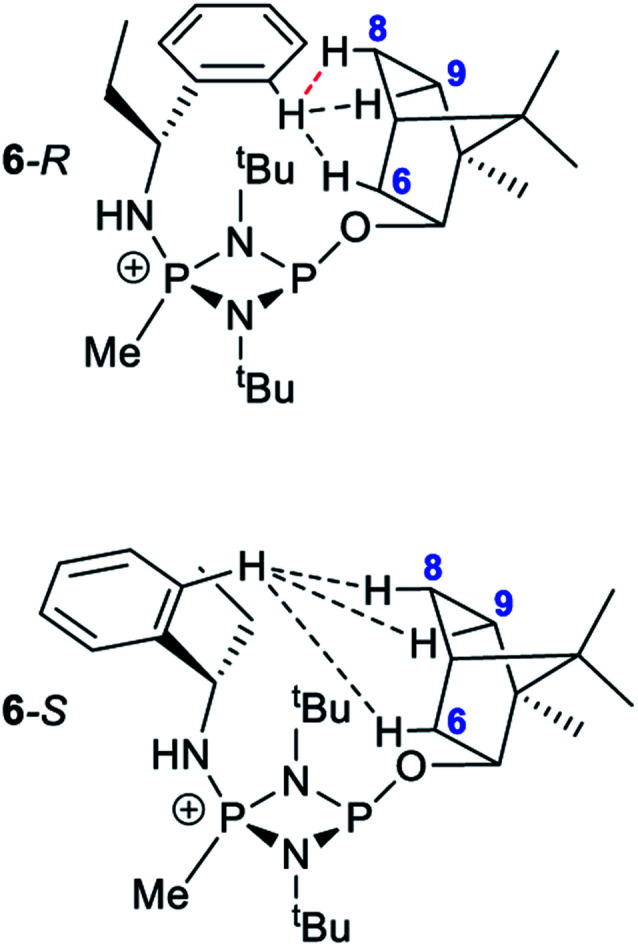
Diagram showing the hydrogen atoms for which NOEs are observed in the cations of 6-*R* and 6-*S*. The red-dotted line indicates a likely NOE.

Several features of the ^1^H NMR spectra of diastereomers 6-*R* and 6-*S* provide greater insight into their solution-state structures (than was possible for 5-*R* and 5-*S*). The first of these is the emergence of well-defined multiplets for the diastereotopic CH_2_-hydrogens of the Et group within the chiral Ph(Et)CH-substituent. In the parent amine (*R*)/(*S*)-Ph(Et)CHNH_2_, only one multiplet (a doublet of quintets, at *δ* 1.69 ppm) is seen. However, incorporation into 6-*R*/6-*S* results in resolution into two signals, at *δ* 2.30 and 2.09 ppm, suggesting conformational stability. NOESY experiments in CDCl_3_ reveal weak NOE's for 6-*R*/6-*S* between the phenyl group *ortho* protons and bornyl ring protons (see ESI Fig. S9e†). A cross peak to H6_*endo*_ is revealed in each case and while for 6-*R* overlap of H8_*endo*_ and H9_*exo*_ precludes firm conclusions regarding the spatial relationship to the amino group, H8_*endo*_ and H9_*exo*_ are well-separated for 6-*S* and there is a clear correlation of H8_*endo*_ with the phenyl group *ortho* protons. Taken together with the visible NOEs between H9_*endo*_ and the *ortho*-phenyl protons in both 6-*R* and 6-*S*, we conclude that their solid-state structures are preserved in solution, in which the phenyl group and *endo* face of the bornyl group are next to each other. This is also consistent with the crystallographically determined H⋯H distances involved (Fig. 10 and Table 1). This conclusion is important because it shows that there are no significant fluxional processes occurring which would alter the spatial arrangement between the OBorn and chiral substituents.

**Table 1:** A comparison of the distances between the hydrogen atoms of 6-*R*/6-*S* indicated in Fig. 10, based upon the calculated positions in their crystal structures, normalised to average neutron C–H distances

Ph-H_ortho_⋯H contact	Distance	
	6-*R* (Å)	6-*S* (Å)
H6	3.91	2.59
H8	2.88	2.11
H9	2.10	2.32

**Fig. 11:**
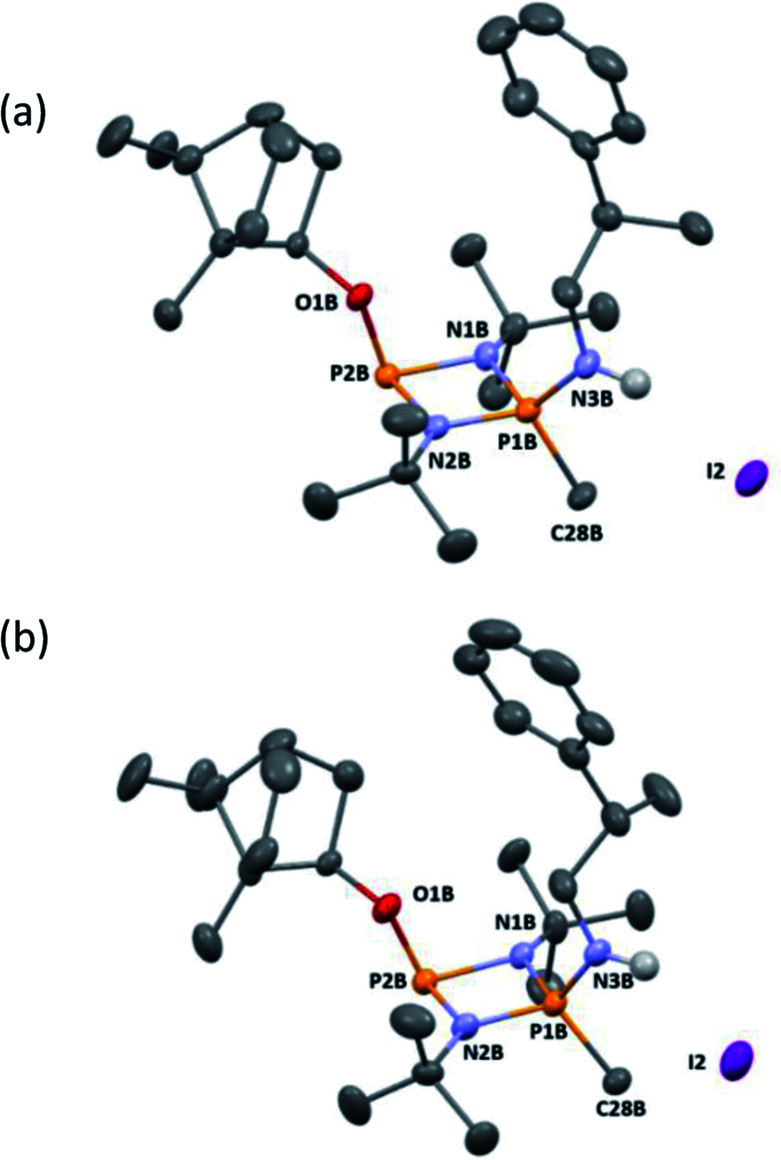
The structure of a representative molecule from the asymmetric unit of 8-*R* (ellipsoids at 30% probability). H atoms on C are omitted for clarity. Selected bond lengths (Å) and angles (°): P1B–N1B 1.635(6), N1B–P2B 1.748(5), P2B–N2B 1.721(6), N2B–P1B 1.642(5), P1B–N3B 1.609(6), P2B–O1B 1.613(5), P1B–C28B 1.780(7); P1B–N1B–P2B 96.5(3), N1B–P2B–N2B 80.2(3), P2B–N2B–P1B 97.3(3), N2B–P1B–N1B 85.9(3), N1B–P1B–N3B 116.3(3), N2B–P1B–N3B 117.0(3), N1B–P1B–C28B 116.2(3), N2B–P1B–C28B 115.7(3), N3B–P1B–C28B 105.5(3), N1B–P2B–O1B 104.3(2), N2B–P2B–O1B 100.4(3). (b) The structure of a representative molecule from the asymmetric unit of 8-*S* (ellipsoids at 30% probability). H atoms on C are omitted for clarity. Selected bond lengths (Å) and angles (°): P1B–N1B 1.639(6), N1B–P2B 1.764(6), P2B–N2B 1.733(6), N2B–P1B 1.637(6), P1B–N3B 1.608(6), P2B–O1B 1.614(6), P1B–C28B 1.781(8); P1B–N1B–P2B 95.9(3), N1B–P2B–N2B 80.0(3), P2B–N2B–P1B 97.2(3), N1B–P1B–N3B 115.3(4), N2B–P1B–N3B 117.0(4), N1B–P1B–C28B 116.9(4), N2B–P1B–C28B 115.4(4), N3B–P1B–C28B 105.4(4), N1B–P2B–O1B 104.2(3), N2B–P2B–O1B 100.3(4).

Ultimately, the utility of our protocol will be limited by the peak separation of the ^31^P NMR resonances of *R*- and *S*- analytes and therefore the ability to integrate accurately. In order to explore this, we next considered 2-phenylpropylamine, PhCH(Me)CH_2_NH_2_, in which the chiral centre is separated by a CH_2_ group from the amine functionality (anticipating a reduction in peak separation). The previous protocol was used, in which 1 was prepared without purification and subsequently reacted with 2-phenylpropylamine in the presence of a Brønsted base (NEt_3_) to give the cyclodiphosph(iii)azane intermediates (*R*)-PhCH(Me)CH_2_NHP(μ-^*t*^BuN)_2_POBorn (7-*R*) and (*S*)-PhCH(Me)CH_2_NHP(μ-^*t*^BuN)_2_POBorn (7-*S*) with the pure enantiomers and (*R*/*S*)-PhCH(Me)CH_2_NHP(μ-^*t*^BuN)_2_POBorn (7-*R*/*S*) with racemic 2-phenylpropylamine. Further treatment of crude intermediates 7-*R*, 7-*S*, 7-*R*/*S* with methyl iodide and recrystallization from toluene afforded excellent crystals of [{(*R*)-PhCH(Me)CH_2_NH}(Me)P(μ-^*t*^BuN)_2_POBorn]I (8-*R*), [{(*S*)-PhCH(Me)CH_2_NH}(Me)P(μ-^*t*^BuN)_2_POBorn]I (8-*S*) and [{(*R*/*S*)-PhCH(Me)CH_2_NH}(Me)P(μ-^*t*^BuN)_2_POBorn]I (8-*R*/*S*). The solid-state structures of 7-*R*, 7-*S*, 7-*R*/*S*, 8-*R*, 8-*S* and 8-*R*/*S* (ESI Fig. S60–S64†) were obtained. The structures of 8-*R* and 8-*S* (one of the crystallographically independent molecules) are shown in Fig. 11.

Notably, the crystal structures of the diastereomers are isostructural for both 7-*R*/7-*S* and 8-*R*/8-*S*, showing that the molecular conformation (in the solid state) is effectively invariant for the two diastereomers in each case. The *R* or *S* configuration at the chiral centre of the amine is accommodated with no change in conformation for the remainder of the molecule, and in particular no change to the orientation of the bornyl group. The molecular similarity is demonstrated explicitly by the crystal structures of the racemic compounds 7-*R*/*S* and 8-*R*/*S*, which show disorder indicative of a solid solution of the two diastereomers. If this situation should be retained in solution, we would expect the NMR spectra of the diastereomers of 8-*R* and 8-*S* to show much smaller differences compared to the previous examples 5 and 6.

**Fig. 12:**
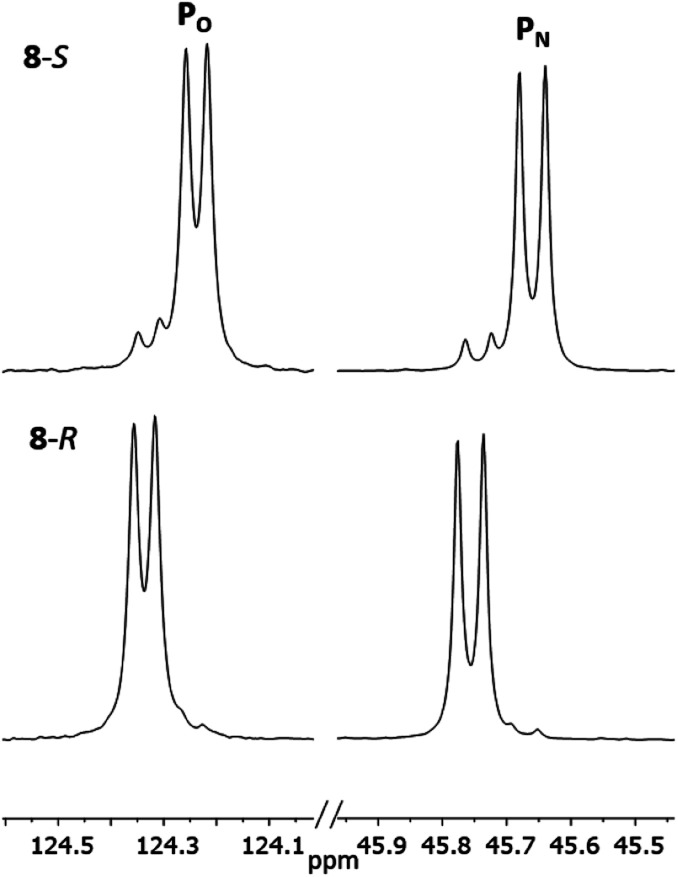
Expansions of the ^31^P{^1^H} NMR spectra (CDCl_3_) of 8-*R* and 8-*S*.

The ^1^H NMR spectra of 8-*R* and 8-*S* are indeed very similar (see ESI Fig. S13a, S14a and S15a†). Importantly, however, very sharp ^31^P{^1^H} NMR signals are still observed (at *δ* 124.3 and 45.7 ppm for 8-*R* and *δ* 124.2 and 45.7 ppm for 8-*S*; Fig. 12). This can be compared to 7-*R* and 7-*S* which show two broad peaks at *δ* 138 and 103 ppm for both. ^31^P{^1^H} NMR spectroscopy gave sufficient line separation for integration of the P_N_ peaks at *ca*. *δ* 46 ppm for the two diastereomers in racemic 8-*R*/*S*, with the best peak resolution obtained by inverse-gated decoupling (with recycle delays ≥30 s, 30° pulse) and this was additionally required in order to obtain reliable integration values.

**Fig. 13:**
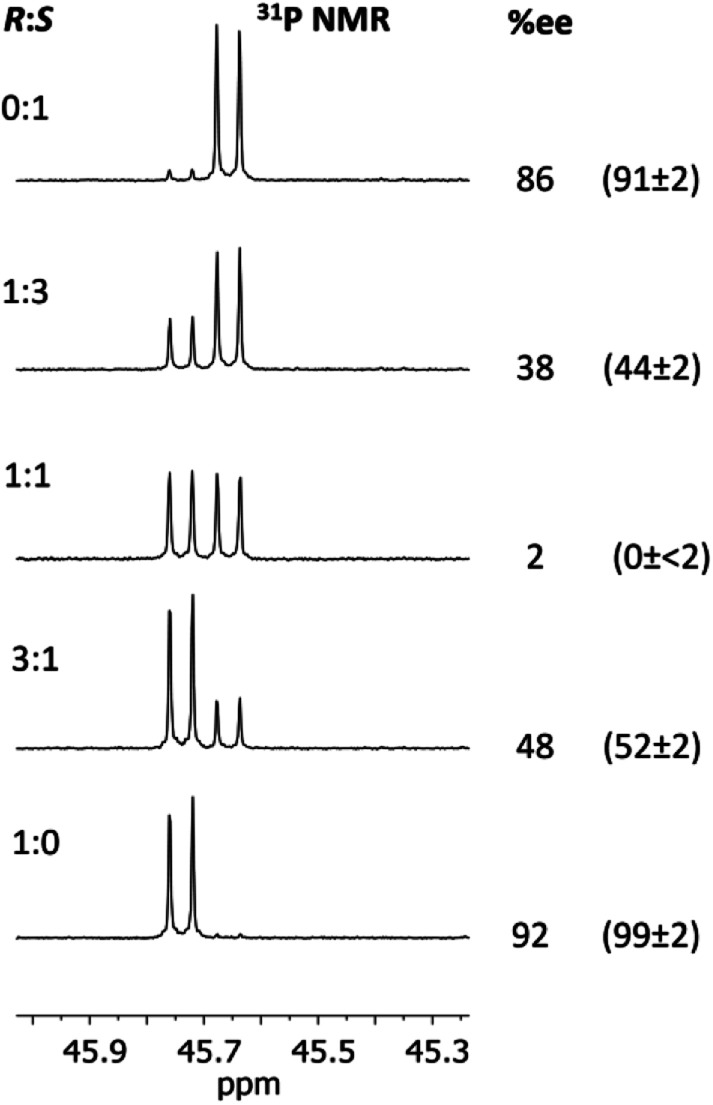
Determination of the *ee*'s for mixture of 2-phenylpropylamine using 1 The conditions are the same as those given in the caption to Fig. 9. The determined %*ee*'s are shown on the right, and their predicted *ee*'s based on the ratios of the enantiomers added are given in parentheses. The *ee*'s of the pure enantiomers and racemic amine were determined independently by chiral HPLC (since these were not stated by the supplier, Sigma). The amines were converted into amides by reaction with acetic anhydride for this purpose. There is an estimated error of up to 2% in the determined *ee*'s due to poor separation on the column. The other predicted values (1 : 3 and 3 : 1) were calculated based on the *ee*'s of the ‘pure’ enantiomers determined from HPLC (so an error of up to *ca* 2% applies to these values too).

Fig. 13 shows the determination of the *ee*'s of mixtures of the *R*- and *S*-isomers of 2-phenylpropylamine using our *in situ* protocol. It is clear from these data that the measured and predicted values are reasonably close to the values determined by chiral HPLC (bearing in mind the estimated *ca* 2% error in these values due to poor separation of the acetyl amide on the column). However, the extent to which the overall molecular shape is affected by changing the chiral centre in the amine from *R* to *S* probably makes our method less suitable for amines in which the chiral substituent is remote from the amine functionality.

### Substrate scope

**Scheme 6:**
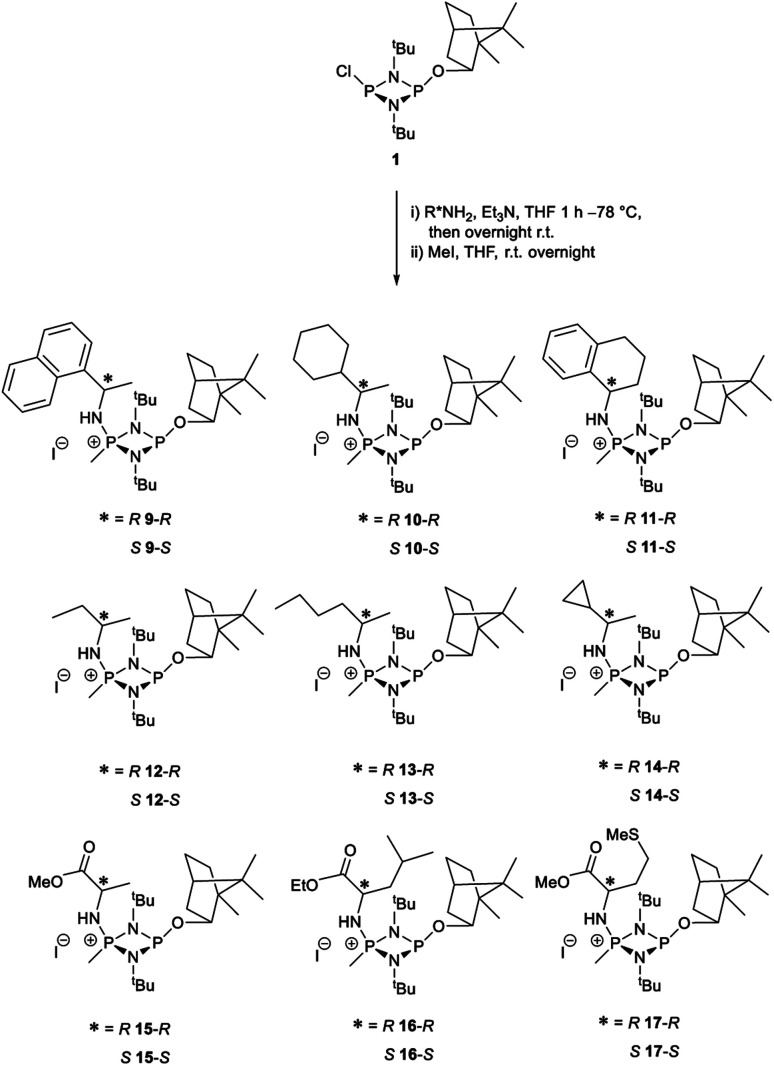
Synthesis of 9–17-*R*/*S*. Chiral centre in the amino groups marked *.

The most important condition that must be met to apply our methodology for measuring *ee* is adequate line separation of the ^31^P NMR signals. In order to establish the scope of the method, a greatly expanded range of commercially available chiral α-primary amines incorporating a variety of functional groups and steric characteristics was explored to establish if there are sufficiently large chemical shift separations of the ^31^P NMR resonances (Scheme 6). The eighteen new compounds (9–17, -*R* and -*S*) were prepared starting from enantiopure amines and fully characterised, including the single-crystal X-ray structures of 10-*R*, 10-*S*, 12-*R*, 12-*S*, 13-*R* and 13-*S*. Full details of the spectroscopic analyses and characterisation of 9–17 are included in the ESI. Of particular interest are the expansions of ^31^P{^1^H} NMR spectra showing the peak separation between products with the pairs of enantiomeric substrates (ESI Fig. S43–S51†). The results (including those for previously discussed 5, 6 and 8) are summarised in Table 2 which also indicates whether ^31^P or ^31^P{^1^H} NMR provide sufficient separation of the resonances for measurement of the *ee*.

**Table 2:** A summary of the ^31^P NMR data, separation of low-field (P_O_) and high-field (P_N_) signals, and an indication of the suitability of ^31^P NMR for measuring *ee* (with and without decoupling)

Substrate (compound number)	P_O_ (*δ* ppm)	P_N_ (*δ* ppm)	ΔP_O_	ΔP_N_	Suitability for *ee* measurement	
					^31^P	^31^P{^1^H}
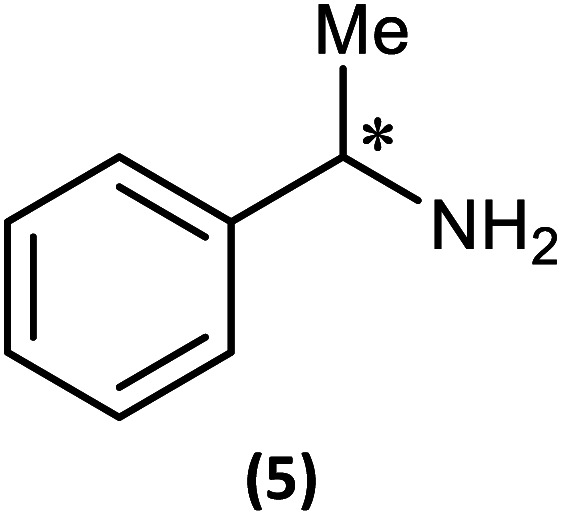	123.0 (*S*)	38.8 (*R*/*S*)	0.33	0.03	Good	Good
	122.7 (*R*)	*δ*_R_ > *δ*_S_				
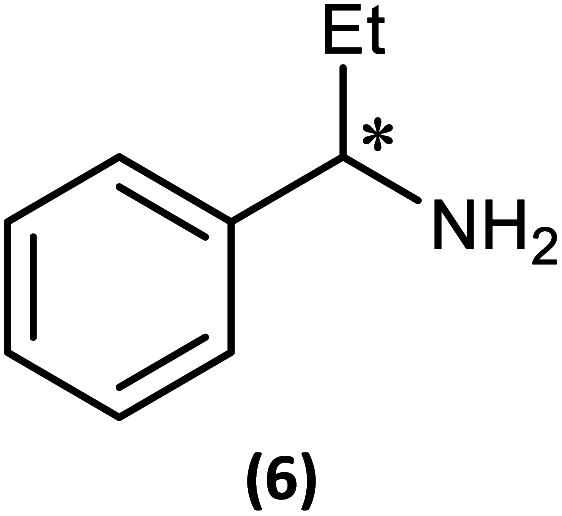	123.1 (*S*)	38.9 (*S*)	0.47	0.04	Good	Good
	122.6 (*R*)	38.8 (*R*)				
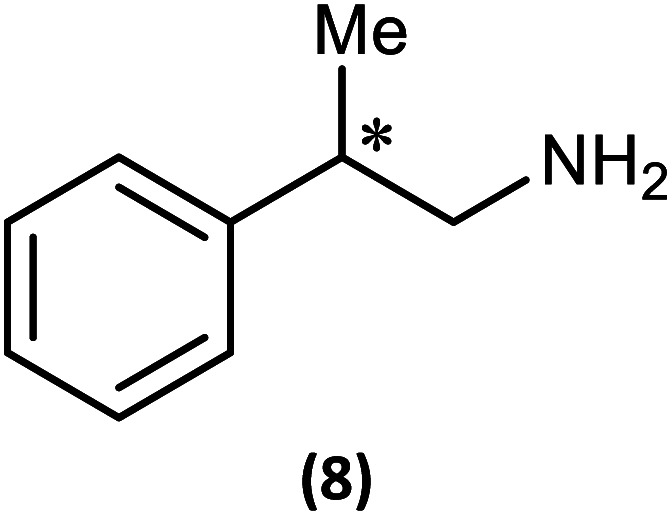	124.3 (*R*/*S*)	45.7 (*R*/*S*)	0.09	0.09	Poor	Medium
	*δ*_R_ > *δ*_S_	*δ*_R_ > *δ*_S_				
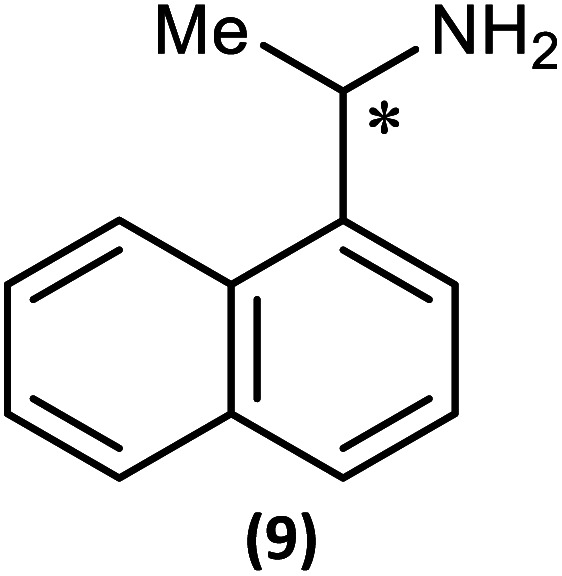	122.9 (*S*)	39.2 (*R*/*S*)	0.34	0.07	Good	Good
	122.6 (*R*)	*δ*_R_ > *δ*_S_				
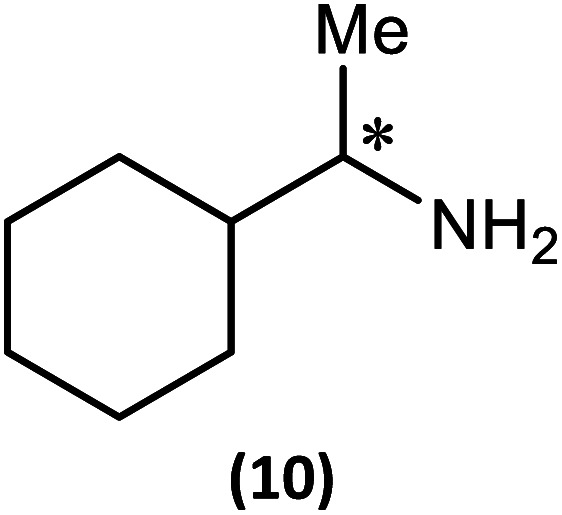	122.5 (*S*)	41.2 (*R*/*S*)	0.22	0	Poor	Good
	122.2 (*R*)	*δ*_R_ ≈ *δ*_S_				
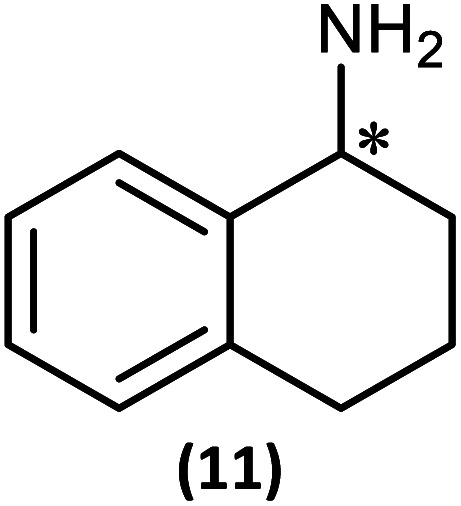	123.7 (*S*)	41.9 (*S*)	0.16	0.10	Poor	Good
	123.5 (*R*)	42.0 (*R*)				
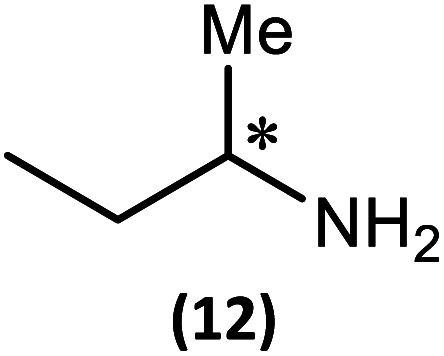	122.4 (*R*/*S*)	41.2 (*R*/*S*)	0.01	0.04	Poor	Medium
	*δ*_S_ > *δ*_R_	*δ*_R_ > *δ*_S_				
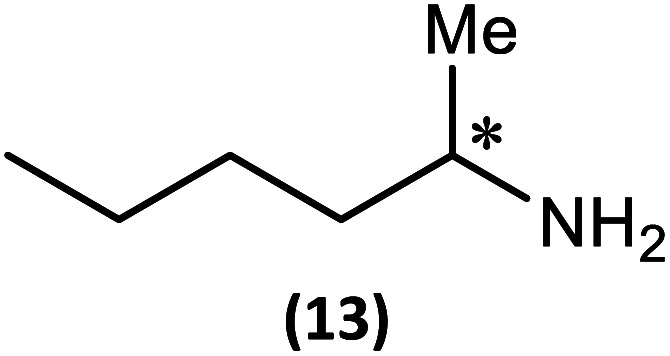	122.2 (*R*/*S*)	40.7 (*R*/*S*)	0	0.07	Poor	Good
	*δ*_S_ ≈ *δ*_R_	*δ*_R_ > *δ*_S_				
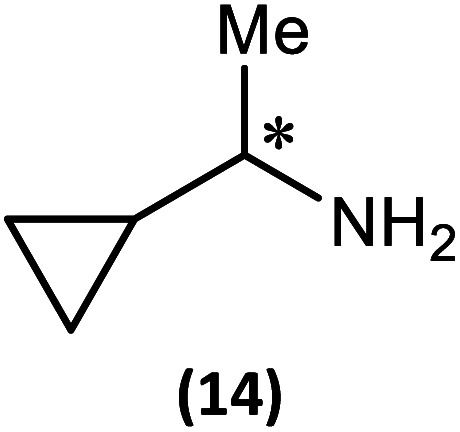	121.8 (*S*)	39.2 (*R*/*S*)	0.52	0.02	Good	Good
	121.3 (*R*)	*δ*_S_ > *δ*_R_				
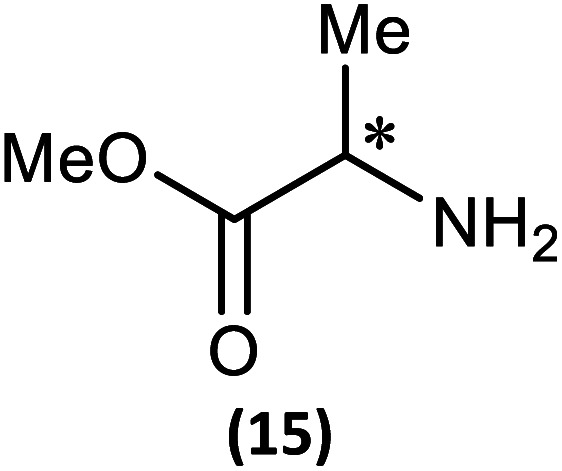	124.1 (*S*)	44.2 (*S*)	0.39	0.28	Good	Good
	123.7 (*R*)	43.9 (*R*)				
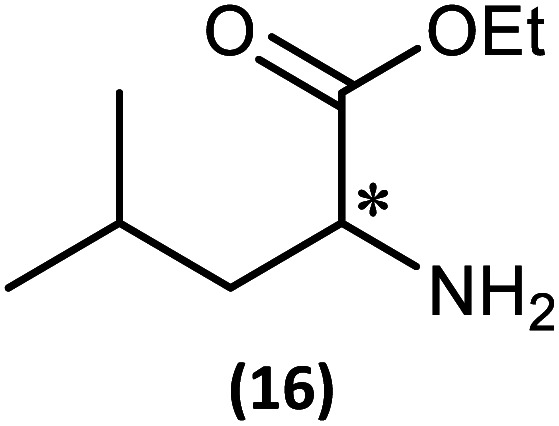	123.4 (*S*)	42.3 (*S*)	0.30	0.67	Good	Good
	123.1 (*R*)	41.6 (*R*)				
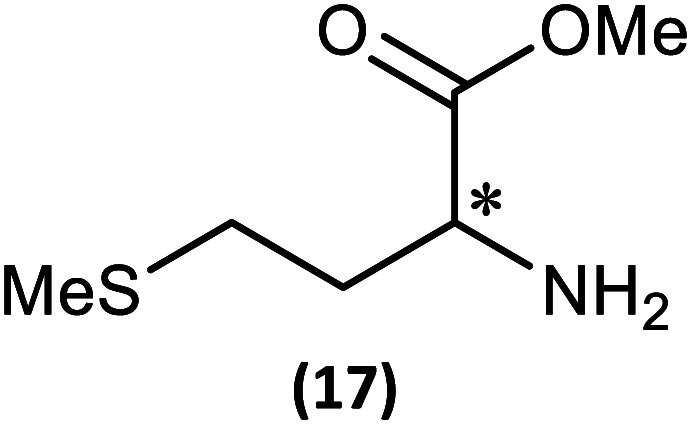	123.9 (*R*/*S*)	43.5 (*S*)	0.02	0.16	Poor	Good
	*δ*_S_ > *δ*_R_	43.4 (*R*)				

**Scheme 7:**
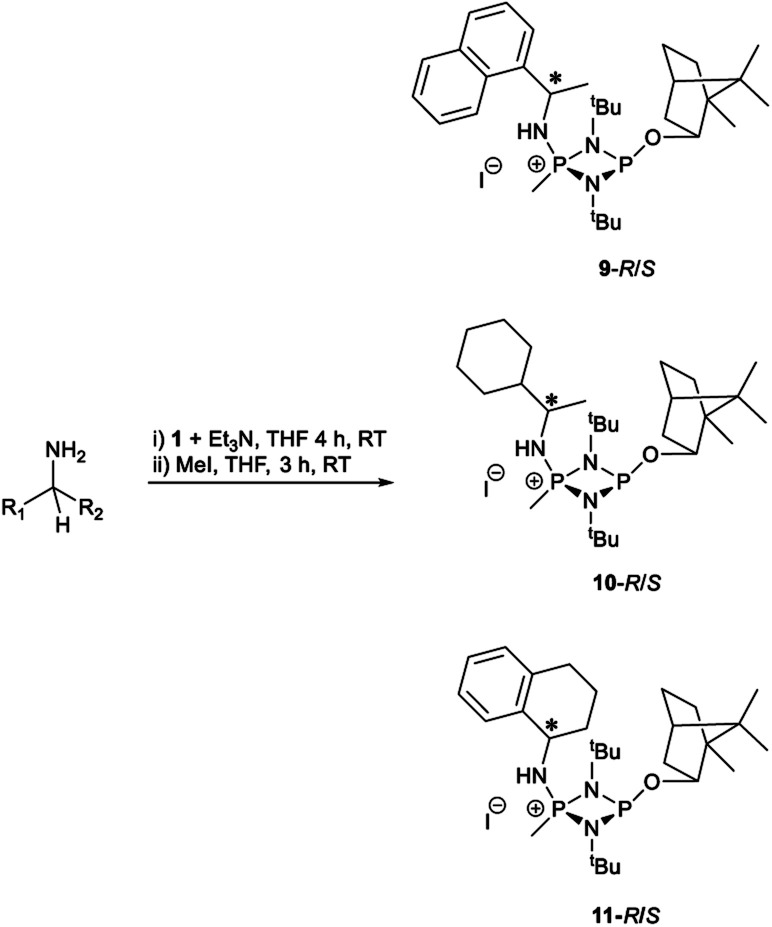
*In situ* formation of 9–11-*R*/*S*. Chiral centre in the amines marked *.

Products 9–11 were also prepared using our *in situ* method outlined in Fig. 8 (see Scheme 7) to confirm its applicability over a larger number of substrates. The resulting ^31^P NMR spectra starting from racemic and 2 : 1 *R* : *S* scalemic mixtures of 9–11 are well resolved and appended in the ESI (Fig. S37–S39, S52†). The assignment thereof is supported by the spectra of the corresponding enantiopure compounds prepared individually.

The major conclusion that can be drawn from the data shown in Table 2 is that 1 can be used to discriminate between all of the chiral amines studied, although the small values of Δ*δ* for both P_O_ and P_N_ in the cases of 8, 12 and 13 would lead to less accurate determination of *ee*. In these cases the use of an inverse gated ^31^P{^1^H} decoupling routine is, however, found to provide sufficient peak resolution for these substrates. While the inherent difficulties in obtaining accurate *ee*'s using the integration method limit the sensitivity of the method for ^31^P NMR spectroscopy, the sensitivity is clearly more than adequate to provide a quick assessment of chiral purity on a convenient scale and in a straightforward manner (using the P_O_ or P_N_ signals).

There are four other conclusions that we can draw from these data:

(i) the presence of aromatic substituents on the primary amine is not required to promote significant chiral inequivalence; rather, the origin of the separation of the P_O_ and P_N_ resonances of the *R* and *S* diastereomers is probably steric/spatial in origin.

(ii) The poorest peak separations are seen in 8, 12, 13 and 17 in which there is apparently the greatest degree of conformational flexibility in the aliphatic groups of the primary amines. This is particularly noticeable in the case of 13 in which the conformational flexibility present in the *n*-butyl group results in the smallest P_O_ and P_N_ peak separation between the diastereomers.

(iii) Perhaps unsurprisingly bearing in mind the mild conditions and reagents involved, there appears to be good functional group tolerance across the spectrum of primary amines investigated, especially in the case of amino acid esters which bear greater chemical functionality (15–17).

(iv) There is a noticeably strong correlation between the P_O_ chemical shift and the absolute configuration for the eleven α-amines explored (*i.e.*5, 6, 9–17). With the exception of 13 (where *δ*_S_ ≈ *δ*_R_), the P_O_ chemical shift of the *S*-isomer is always higher than for the *R*-isomer (*δ*_S_ > *δ*_R_). It can be noted that the β-amine 2-phenylpropylamine (8) does not fit the trend. In contrast, the P_N_ resonances do not exhibit any such clear correlation; 5, 8, 9, 11, 12, 13 have higher P_N_ chemical shifts for the *R*-isomer (*δ*_R_ > *δ*_S_) whereas the opposite holds for 6, 14, 15, 16, 17 (*δ*_S_ > *δ*_R_), and only for 10*δ*_S_ ≈ *δ*_R_.

Our reagent 1 can therefore be used to determine the absolute configuration of chiral α-amines, with the P_O_ chemical shift being a reliable indicator. This is important because, uniquely, this can be done at the same time as the determination of the *ee* in a single ^31^P NMR experiment of a scalemic mixture.

**Fig. 14:**
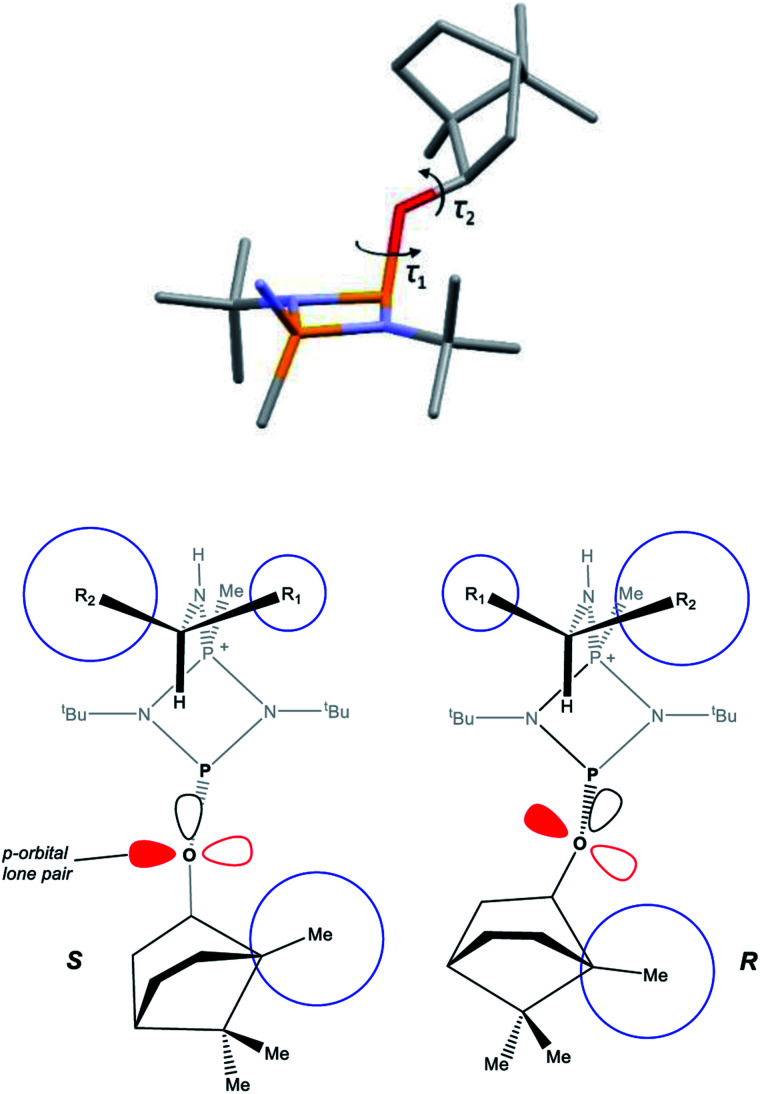
(a) Defining the P⋯P–O–C_Born_ (*τ*_1_) and P–O–C_Born_–C(Me) (*τ*_2_) torsion angles; (b) diagrammatic representation of the solid-state structures of *R*- and *S*-isomers (in which the O atoms are all sp^2^ hybridised, consistent with the X-ray structures which show C–O–P angles close to 120°), showing the orientation of the lone pair in p and sp^2^ orbitals on the O atom. The blue circles are used to highlight the relative steric sizes of the groups. The steric interactions between the two ends of the molecule are mediated through the *t*-butyl groups, which show rotational variation.

A clue to the probable feature influencing the P_O_ chemical shifts is provided by the numerous snapshots of the molecular conformation established within the crystal structures. Since several of the structures show more than one independent molecule and/or disorder of the bornyl group, more than 30 such snapshots are available (see ESI Section 4†). The orientation of the bornyl group with respect to the P_2_N_2_ ring is described by the P⋯P–O–C_Born_ (*τ*_1_) and P–O–C_Born_–C(Me) (*τ*_2_) torsion angles, as shown in Fig. 14a. A scatterplot of *τ*_1_*vs. τ*_2_ (ESI, Fig. S73†) shows an overall broad spread of points (indicating that the bornyl conformation is not inherently “locked”), but with a prominent cluster comprising 14 points centred at *τ*_1_ ≈ 157°, *τ*_2_ ≈ 120°, which corresponds to the bornyl conformation shown in Fig. 2b and 6a. The cluster includes at least one molecule from *all* characterised compounds showing the O_exo_ conformation, which indicates that it is likely to represent (at least a local) energy minimum for the orientation of the bornyl group. Although the snapshots are taken from the crystal structures, while the P_O_ resonances are measured in solution, any conformation observed in the solid state must be intrinsically accessible in solution, while a conformation not observed in the solid state is not proven to be. Considering the quaternised compounds, both the *R*- and *S*- diastereomers are seen within the cluster for 8, 12 and 13, and little difference is seen between *δ*_S_ and *δ*_R_ in the ^31^P NMR. For 5, 6 and 10, however, the *R*-diastereomer is seen within the cluster but the *S*-diasteromer is not, and there is a clear distinction between *δ*_S_ and *δ*_R_ in the ^31^P NMR. Hence, we conclude that discrimination between *δ*_S_ and *δ*_R_ for the P_O_ resonance arises when the *S*-substrate blocks access to the low-energy bornyl conformation. The sharpness of the signals results from conformational locking of the bornyl group, while the difference between *δ*_S_ and *δ*_R_ indicates the extent of the difference between the two locked conformations. For amines where both the *R*- and the *S*-substrate permit access to the same bornyl conformation, the discrimination will be reduced.

The observed conformational differences are interpreted to be steric in origin. The ‘Me-side’ of the bornyl group appears to have a large steric influence, with the most sterically demanding group of the amine (R_2_ in Fig. 14b) being diagonally opposite to the Me-side in the *S*-isomer, but adjacent in the *R*-isomer. The distance between bornyl and amine is too large to have a direct effect, but the steric interaction is mediated by the *tert*-butyl group, which changes its orientation in the *R*- and *S*- diastereomers. The effect is clearest in 5 and 6, where the *tert*-butyl groups show an eclipsed conformation in the *R*- but a staggered conformation in the *S*-diastereomer.

DFT (B3LYP/TZVP) geometry optimisations of 4-*R*, 4-*S* and 5-*R* in CHCl_3_ reproduce their solid-state structures closely (Fig. S74, S82 and S84, ESI†), with calculated C_Born_–O–P⋯P dihedrals being very similar to those found experimentally. A survey of the frontier orbitals of 5-*R* and 5-*S* does not indicate any donation of the O-lone pairs into the σ* orbital of the ring P–N bonds, and NBO analysis shows that the charges on the principal atoms of both are almost identical (ESI, Table S8†). Given the above, we conclude that the most likely origin of the difference in the chemical shift arising from a change in orientation of the bornyl group is the relative orientation of the O- and P-lone pairs.^35^

## Conclusions

In summary, we have developed a simple inorganic chiral derivatisation agent based on a cyclodiphosphazane, which can be used to detect chirality and measure enantiomeric excess in chiral amines and employs a cheap chiral auxiliary. A two-step process, involving the substitution of the P–Cl group with the amine, followed by selective quaternisation of one of the P centres gives diastereomeric iodide salts of the type [(R*NH)(Me)P(μ-N^*t*^Bu)_2_POBorn]I, where R* is a chiral group and OBorn is the chiral ancillary. ^31^P NMR spectroscopy proves to be particularly useful for measuring the ratio of diastereomers produced from scalemic mixtures, with the small but useable separations in chemical shifts of diastereomeric pairs. The origin of the effect is the steric influence of the chiral amine on the orientation of the bornyl group. Though the method does not give highly accurate measures of enantiomeric excess, it is nonetheless sensitive enough to provide a rapid assay of chiral composition. Screening a range of commercial chiral amines indicates that measurement of *ee* is possible by integration of the P_O_ and/or P_N_^31^P/^31^P{^1^H} NMR resonances. The method is most suited to α-chiral amines in which greatest separation of the P_O_ and P_N_ resonances occurs, but can also be applied to β-chiral amines. Using ^31^P NMR spectroscopy allows detection of chiral derivatives in relatively small amounts (suitable for NMR assays) without any significant interference of side products with the signals of interest. Finally, the relative values of the P_O_ chemical shifts are diagnostic of the absolute configuration of α-alkyl-amines, so that our CDA performs all three tasks; detection of chirality, measurement of *ee* and determination of absolute configuration; in one simple experiment. This contrasts with previously developed organic CDAs (like Mosher's acid) where two separate experiments are required to determine *ee*'s and absolute configuration.^11*a*^ Clearly, our CDA design concept is general, and better selectivity and more accurate measurement of *ee*'s is likely to be possible using other chiral auxiliaries and/or P_2_N_2_ ring substituents. Work on refining these systems and on extending the scope to chiral alcohols and carboxylic acids is underway.

## Conflicts of interest

There are no conflicts to declare.

## Conflicting interests

The authors declare no conflicting interests.

## Data availability

Synthetic procedures for all new compounds, their characterisation and NMR data can be found in the ESI.† Crystallographic data for 1, 2, 3, 4-*R*, 4-*S*, 5-*R*, 5-*S*, 6-*R*, 6-*S*, 7-*R*, 7-*S*, 7-*R*/*S*, 8-*R*, 8-*S*, 8-*R*/*S*, 10-*R*, 10-*S*, 12-*R*, 12-*S*, 13-*R*, 13-*S* have been deposited at the CCDC (deposition numbers 2105705, 2105711, 2105707, 2105709, 2105718, 2105714, 2105706, 2105708, 2105717, 2105710, 2105715, 2105712, 2105716, 2105713, 2105719, 2157896, 2157893, 2157895, 2157894, 2157891, 2157892, respectively) and can be obtained on request. Full coordinates of DFT optimised structures are also available as a separate data file in the ESI.†

## Author contributions

A. J. P. and A. T. undertook experimental work, which was supervised by A. J. P. and J. S.; R. B. J. performed DFT calculations; A. J. P., A. T. and D. C. undertook NMR studies; H.-C. N. aided in experimental work; A. D. B. carried out X-ray crystallography and analysis of the solid-state structures; D. S. W. and A. J. P. conceived the original idea. All authors were involved in the writing of the paper.

## Supplementary Material

SC-013-D2SC01692C-s001

SC-013-D2SC01692C-s002

SC-013-D2SC01692C-s003
