# Supplementary material for: A chiral phosphazane reagent strategy for the determination of enantiomeric excess of amines
Source: Chem Sci. 2022 Apr 25;13(18):5398–412. doi: 10.1039/d2sc01692c (PMC9093139; doi:10.1039/d2sc01692c)
Supplement: SC-013-D2SC01692C-s001 [file SC-013-D2SC01692C-s001.pdf]

Electronic Supplementary Material (ESI) for Chemical Science.
This journal is © The Royal Society of Chemistry 2022

A chiral phosphazane reagent strategy for the determination of enantiomeric excess of amines

Andrew J. Peel,[a,*] Alexandros Terzopoulos,[a] Rajesh B. Jethwa,[a] Dipanjana Choudhury,[a] Hao-Che Niu,[a] Andrew D. Bond,[a] Jonathan Slaughter[a] and Dominic S. Wright[a,*]

[a] *Yusuf Hamied Department of Chemistry, Cambridge University, Lensfield Road, Cambridge CB2 1EW (U.K.)*

Supplementary Information

Table of Contents

1. Synthesis and Characterisation of New Compounds 1
2. NMR Spectroscopy 35
3. Substrate Scope 105
4. X-ray Crystallography 113
5. Calculations 132
6. Bibliography 143

1. Synthesis and Characterisation of New Compounds

General synthetic and analytical details

Reactions were carried out under dry nitrogen, using double manifold and glove-box methods. Solvents were distilled off sodium (toluene), sodium-potassium amalgam (THF, Et_2O, hexane and pentane) or CaH_2 (triethylamine) immediately before use. Reagents were purchased from commercial suppliers (Sigma Aldrich, Alfa Aesar, or Acros Organics). Phosphorus trichloride was distilled prior to use, *tert*-butylamine was used as received, (*R*)- and (*S*)-1,2,3,4-tetrahydro-1-naphthylamine were distilled under reduced pressure in a nitrogen atmosphere and dried over molecular sieves (4 Å), other amines were dried over molecular sieves (4 Å) and stored under nitrogen. Amino acid ester hydrochloride salts were dried under vacuum overnight and stored under nitrogen. The synthesis of $[ClPN^tBu]_2$ was based on the literature.[1] NMR data were collected on a Bruker Avance III HD 500 MHz Smart Probe FT NMR spectrometer (500.200 MHz for ^1H, 125.775 MHz for ^{13}C and 202.485 MHz for ^{31}P). Spectra were obtained at 25 °C (unless otherwise stated) using deuterated solvent stored over molecular sieves (4 Å). For ^1H, chemical shifts are internally referenced to deuterated solvent and calculated relative to TMS.[2] All other nuclei are reported relative to ^1H using standard absolute frequency ratios.[3] Chemical shifts are expressed in δ ppm. The following abbreviations are used: br = broad, m = multiplet, s = singlet, sh = shoulder. Elemental analysis for carbon, hydrogen, and nitrogen was performed using a Perkin Elmer 240 Elemental Analyser or an Exeter Analytical CE-440 Elemental Analyser. Melting points were recorded using a Griffin melting point apparatus. X-ray crystallographic data were collected using a Nonius KappaCCD (Mo Kα, λ= 0.71073 Å) or Bruker D8-QUEST PHOTON-100 diffractometer equipped with an Incoatec IµS Cu microsource (Cu Kα, λ= 1.5418 Å). The temperature was held at 180(2) or 220(2) K using an Oxford Cryosystems N_2 cryostat. Data integration and reduction were undertaken with HKL Denzo/Scalepack (Nonius) or with SAINT in the APEX3 software suite (Bruker). Multi-scan corrections were applied using SORTAV (Nonius) or SADABS (Bruker). Structures were solved using SHELXT[4] and refined using SHELXL.[5]

Synthesis and characterisation of ClP(μ-N^{t}Bu)$_2$POBorn 1

[ClPN^{t}Bu]$_2$ (550 mg, 2 mmol) was dissolved in THF (12 ml). Triethylamine (1 ml, excess) was added and the solution was cooled to −78 °C. *Endo*-(−)-borneol (310 mg, 2 mmol) was added, the solution was warmed to room temperature and then stirred overnight, by which time a thick white precipitate had formed. The solvent was removed in vacuo and the residue triturated with Et_2O (12 ml). The solid was digested in toluene (12 ml) and filtered. Removal of the solvent *in vacuo* gave a viscous, slightly cloudy liquid that solidified upon standing at room temperature. The solid was dissolved in the minimum amount of pentane and stored at room temperature for 24 hr, after which time colourless crystals formed (200 mg, 26%). A second crop of crystal was obtained from the supernatant (190 mg, 24%). Combined yield of 390 mg, 50%. M.P. 92–94 °C. Elemental analysis $C_{18}H_{35}ClN_2OP_2$ requires (%): C, 55.03; H, 8.98; N, 7.13. Found (%): C, 54.95; H, 8.92; N, 7.11.

^{1}H NMR spectroscopy (500 MHz, C_6D_6, 298 K): δ 5.09 (1H, m, H5), 2.74 (1H, m, H6$_{exo}$), 2.35 (1H, m, H9$_{endo}$), 1.74 (1H, m, H8$_{exo}$), 1.64 (1H, t, *J* = 5 Hz, H7), 1.38–1.33 (1H, m, H8$_{endo}$), 1.30 (9H, s, H2, H4), 1.38–1.30 (1H, br, H9$_{exo}$), 1.28 (9H, s, H2;H4), 1.02 (3H, s, H14), 0.99 (3H, s, H12), 0.815 (3H, s, H13).

^{13}C NMR spectroscopy (126 MHz, C_6D_6, 298 K): δ 80.3 (d, *J* = 11 Hz, C5), 53.2 (m; C1, C3), 49.7 (d, *J* = 2 Hz, C10), 47.4 (C11) 45.3 (C7), 39.5 (C6), 30.6 (m, C2/C4), 30.5 (m, C2/C4), 28.6 (C8), 27.2 (C9), 19.8 (C13), 18.8 (C14), 14.4 (C12).

^{31}P{^{1}H} NMR spectroscopy (202 MHz, C_6D_6, 298 K): δ 190.5 (d, $^2J_{P\text{-}P}$ = 35 Hz, P_{Cl}), 140.0 Hz (d, $^2J_{P\text{-}P}$ = 35 Hz, P_O).

^{31}P NMR spectroscopy (202 MHz, C_6D_6, 298 K): δ 190.6 (d, $^2J_{P\text{-}P}$ = 35 Hz, P_{Cl}), 140.0 Hz (dd, $^2J_{P\text{-}P}$ = 35 Hz; $^3J_{P\text{-}H}$ = 9 Hz, P_O).

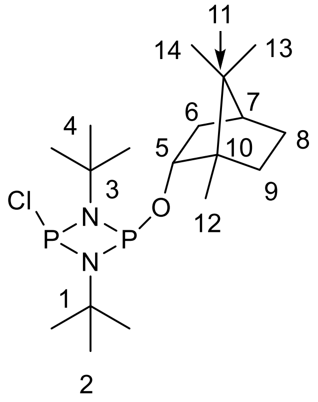

Synthesis and characterisation of [P(μ-N^{t}Bu)OBorn]$_2$ 2

[ClPN^{t}Bu]$_2$ (550 mg, 2 mmol) was dissolved in THF (12 ml). The solution was cooled to −78 °C and triethylamine (0.5 ml, excess) was added, followed by *endo*-(-)-borneol (0.62 g, 4 mmol). The solution was warmed to room temperature overnight. The solvents were removed *in vacuo*, the residue was triturated with diethyl ether and then extracted into toluene (12 ml). The suspension was filtered, concentrated, and stored at −27 °C for 2 weeks to give colourless crystals (75 mg, 7%). M.P. 106–108 °C. Elemental analysis $C_{28}H_{52}N_2O_2P_2$ requires (%): C, 65.85; H, 10.26; N, 5.49. Found (%): C, 65.63; H, 10.26; N, 5.68.

^{1}H NMR spectroscopy (500 MHz, $CDCl_3$, 298 K): δ 5.60 (2H, m, H5), 2.28 (2H, m, H6$_{exo}$), 2.03 (2H, m, H9$_{endo}$), 1.70 (2H, m, H8$_{exo}$), 1.60 (2H, t, J = 5 Hz, H7), 1.32 (18H, s, H2, H4), 1.27–1.11 (6H, m; H6$_{endo}$, H8$_{endo}$, H9$_{exo}$), 0.90 (6H, s, H14), 0.85 (12H, s; H12, H13).

^{13}C NMR spectroscopy (126 MHz, $CDCl_3$, 298 K): δ 78.0 (C5), 51.6 (t, J = 14 Hz, C1, C3), 49.3 (C10), 47.4 (C11), 45.1 (C7), 39.1 (C6), 31.3 (t, J = 6 Hz, C2, C4), 28.3 (C8), 26.9 (C9), 20.1 (C14), 19.0 (C13), 13.9 (C12).

^{31}P{^{1}H} NMR spectroscopy (203 MHz, $CDCl_3$, 298 K): δ 133.9.

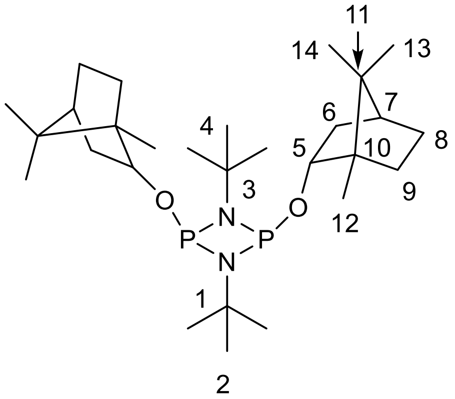

Synthesis and characterisation of (H)(O)P(µ-N^{t}Bu)$_2$POBorn 3

Pre-isolated **1** (140 mg, 0.51 mmol) was dissolved in THF (12 ml) and triethylamine (0.5 ml) was added followed by a few drops of water. The solution was stirred overnight by which time a white precipitate had formed. The solvents were removed *in vacuo*, the residue was triturated with Et_2O (12 ml) and the solid digested in toluene (6 ml). Filtration, removal of the volatiles and recrystallisation from hexane (ca. 2 ml) at −15 °C gave colourless crystals (75 mg, 39%). M.P. 88–90 °C. Elemental analysis $C_{18}H_{36}N_2O_2P$ requires (%): C, 57.74; H, 9.69; N, 7.48. Found (%): C, 57.49, H; 9.71, N, 7.65.

^{1}H NMR spectroscopy (500 MHz, $CDCl_3$, 298 K): δ 7.42 (1H, d, J = 585 Hz, P(=O)H), 4.51 (1H, m, H5), 2.33 (1H, m, H6$_{exo}$), 1.98 (1H, m, H9$_{endo}$), 1.70 (1H, m, H8$_{exo}$), 1.64 (1H, t, J = 5 Hz, H7), 1.39 (9H, s, H2/H4), 1.38 (9H, s, H2/H4), 1.27–1.18 (3H, m, H6$_{endo}$, H8$_{endo}$, H9$_{exo}$), 0.88 (3H, s, H14), 0.86 (3H, s, H12), 0.85 (3H, s, H13).

^{13}C NMR spectroscopy (126 MHz, $CDCl_3$, 298 K): δ 79.3 (d, J = 2 Hz, C5), 52.4 (m, C1, C3), 49.5 (d, J = 3Hz, C10), 47.4 (C11), 45.0 (C7), 38.5 (d, J = 2 Hz, C6), 31.4–31.2 (C2, C4), 28.3 (C8), 26.8 (C9), 19.9 (C14), 18.8 (C13), 13.9 (C12).

^{31}P{^{1}H} NMR spectroscopy (203 MHz, $CDCl_3$, 298 K): δ 96.9 (d, J = 10 Hz, P$_{OBorn}$), −7.01 (d, $^2J_{P\text{-}P}$ = 10 Hz, P$_{(=O)H}$).

^{31}P NMR spectroscopy (203 MHz, $CDCl_3$, 298 K): δ 96.9 (t, J = 10 Hz, P$_{OBorn}$), –7.01 (dd, $^1J_{P\text{-}H}$ = 585 Hz, $^2J_{P\text{-}P}$ = 10 Hz, P$_{(=O)H}$).

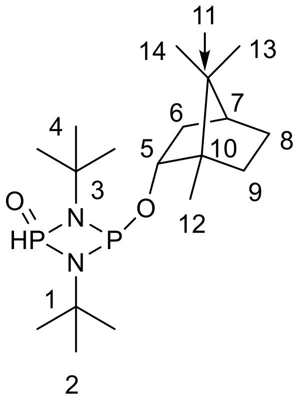

Synthesis and characterisation of {(*R*)-$C_6H_5CH(CH_3)NH$}P(μ-N^tBu)$_2$POBorn 4-*R*

1 was prepared *in situ*, without purification from [ClPN^{t}Bu]$_2$ (0.55 g, 2.0 mmol) and dissolved in THF (12 ml). (*R*)-1-phenylethylamine (0.24 ml, 2 mmol) and triethylamine (1 ml, excess) were added at −78 °C and the solution was warmed to room temperature overnight with stirring. The THF was removed *in vacuo*, the residue was triturated with Et_2O (12 ml) and digested in toluene (12 ml). The solution was filtered, and the solvent removed *in vacuo* to yield a solid. A few drops of toluene were added and the solid was melted. Cooling of the melt to room temperature yielded colourless crystals after 24 h (100 mg, 10%). M.P. 89–90 °C. Elemental analysis $C_{26}H_{45}N_3OP_2$ requires (%): C, 65.38; H, 9.50; N, 8.80. Found (%): C, 65.32; H, 9.52; N, 8.81.

^{1}H NMR spectroscopy (500 MHz, $CDCl_3$, 298 K): δ 7.34 (2H, d, *J* = 7 Hz, H18), 7.28 (2H, t, *J* = 7 Hz, H19), 7.18 (1H, t, *J* = 7 Hz, H20), 4.94 (1H, s, br, H15), 4.34 (1H, t, *J* = 11 Hz, H5), 3.22 (1H, dd, *J* = 30, 10 Hz, N–H), 2.28 (1H, m, $H6_{exo}$), 2.17 (1H, m, $H9_{endo}$), 1.77 (1H, m, $C8_{exo}$), 1.66 (1H, t, *J* = 5 Hz, H7), 1.43 (3H, d, J = 7 Hz, H16), 1.40–1.24 (3H, br, m, $H6_{endo}$, $H9_{exo}$, $H8_{endo}$), 1.30 (9H, s, H2), 1.09 (9H, s, H4), 0.90 (3H, s, H14), 0.89 (3H, s, H13), 0.88 (3H, s, H12).

^{13}C NMR spectroscopy (126 MHz, $CDCl_3$, 298 K): δ 147.2 (C17), 128.1 (C19), 126.8 (C18), 126.2 (C20), 78.5 (C5), 51.5 (t, *J* = 14 Hz, C1), 51.2 (t, *J* = 14 Hz, C3), 49.6 (d, *J* = 4 Hz, C10), 48.7 (C15 from *HSQC*), 47.5 (C11), 45.2 (C7), 39.1 (C6), 31.3 (t, *J* = 7 Hz, C2), 30.8 (t, *J* = 6 Hz, C4), 28.4 (C8), 26.9 (C9, C16), 20.1 (C13), 18.9 (C14), 13.8 (C12).

^{31}P{^{1}H} NMR spectroscopy (202 MHz, $CDCl_3$, 298 K): δ 142 (br, P_O),134.4 (**2**), 98 (br, P_N).

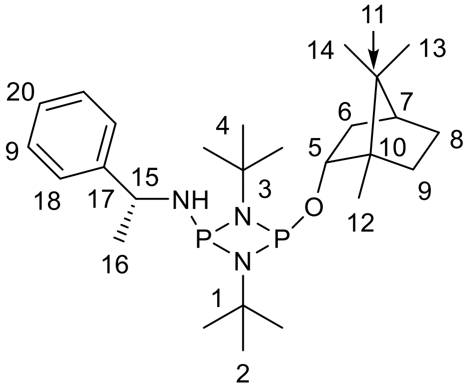

Synthesis and characterisation of {(S)-$C_6H_5CH(CH_3)NH$}P(μ-N^tBu)$_2$POBorn 4-*S*

1 was prepared *in situ*, without purification from [ClPN^{t}Bu]$_2$ (1.47 g, 5.3 mmol) and dissolved in THF (24 ml). (*S*)-1-phenylethylamine (0.68 ml, 5.3 mmol) and triethylamine (1 ml, excess) were added at −78 °C and the solution was warmed to room temperature overnight with stirring. The solvent was removed *in vacuo*, the solid triturated with Et_2O (24 ml) and digested in toluene (24 ml). The solution was filtered, and the toluene was removed *in vacuo*. A few drops of toluene were added and the solid was melted. Cooling of the melt gave colourless crystals after 24 h (480 mg, 19%). M.P. 98–100 °C. Elemental analysis $C_{26}H_{45}N_3OP_2$ requires (%): C, 65.38; H, 9.50; N, 8.80. Found (%): C, 64.72; H, 9.44; N, 8.58.

^{1}H NMR spectroscopy (500 MHz, $CDCl_3$, 298 K): *δ* 7.33 (2H, d, *J* = 7 Hz, H18), 7.29 (2H, t, *J* = 7 Hz, H19), 7.00 (1H, t, *J* = 7 Hz, H20), 4.94 (1H, s, br, H15), 4.40 (1H, m, H5), 3.21 (1H, dd, *J* = 30, 10 Hz, N–H), 2.35 (2H, m, $H6_{exo}$, $H9_{endo}$), 1.76 (1H, m, $H8_{exo}$), 1.66 (1H, t, *J* = 5 Hz, H7), 1.43 (3H, d, *J* = 7 Hz, H16), 1.39 (1H, d, *J* = 5 Hz, unidentified), 1.31 (9H, s, H4), 1.30–1.15 (3H, m, $H6_{endo}$, $H9_{exo}$, $H8_{endo}$), 1.09 (9H, s, H2), 0.90 (3H, s, H12), 0.90 (3H, s, H14), 0.88 (3H, s, H13).

^{13}C NMR spectroscopy (126 MHz, $CDCl_3$, 298 K): *δ* 147.8 (C17), 128.1 (C19), 126.3 (C18), 126.2 (C20), 78.6 (C5), 51.4 (m, C1, C3), 49.6 (d, *J* = 4 Hz, C10), 48.2 (C15), 47.4 (C11), 45.2 (C7), 38.9 (C6), 31.3 (t, *J* = 7 Hz, C4), 30.7 (t, *J* = 7 Hz, C2), 28.4 (C8), 27.1 (C9), 26.8 (C16), 20.1 (C13), 18.9 (C14), 13.9 (C12).

^{31}P{^{1}H} NMR spectroscopy (202 MHz, $CDCl_3$, 298 K): *δ* 139 (br, P_O), 133.2 (**2**), 98 (br, P_N), 96.9 (**3**) −6.9 (**3**).

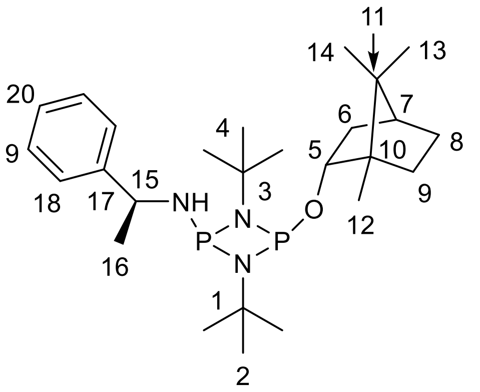

Synthesis and characterisation of [{(*R*)-$C_6H_5CH(CH_3)NH$}(Me)P(µ-N^tBu)$_2$POBorn]I 5-*R*

4-*R* was prepared *in situ*, without purification from [$ClPN^tBu$]$_2$ (0.55 g, 2 mmol) and dissolved in hexane (12 ml). MeI (1 ml, excess) was added, and the solution was stirred at room temperature overnight, during which time a thick white precipitate had formed. The volatiles were removed *in vacuo* and the residue was recrystallised from hot THF (ca. 6 ml) to give colourless crystals (280 mg, 23%). M.P. decomp ca. 230 °C. Elemental analysis $C_{27}H_{48}IN_3OP_2$ requires (%): C, 52.34; H, 7.81; N, 6.78. Found (%): C, 51.78; H, 7.98; N, 6.58.

^{1}H NMR spectroscopy (500 MHz, $CDCl_3$, 298 K): δ 8.40 (1H, dd, *J* = 23, 11 Hz, N–H), 7.64 (2H, d, *J* = 8 Hz, H18), 7.30 (2H, t, *J* = 8 Hz, H19), 7.23 (1H, t, *J* = 8 Hz, H20), 4.65 (1H, m, H15), 4.40 (1H, t, *J* = 11 Hz, H5), 2.47 (1H, d, *J* = 15 Hz, C21), 2.43 (1H, m, $H6_{exo}$), 1.99 (1H, m, $H9_{endo}$), 1.89 (1H, m, $H8_{exo}$), 1.78 (1H, t, *J* = 5 Hz, H7), 1.75 (3H, d, *J* = 7 Hz, H16), 1.48 (9H, s, H4), 1.44–1.27 (3H, m, $H6_{endo}$, $H8_{endo}$, $H9_{exo}$), 0.92 (3H, sh, H13), 0.90 (15H, s, br, H14, H12, H2).

^{13}C NMR spectroscopy (126 MHz, $CDCl_3$, 298 K): δ 143.7 (C17), 128.6 (C19), 127.7 (C20), 127.3 (C18), 83.2 (d, *J* = 20 Hz, C5), 54.4 (d, *J* = 8 Hz, C4), 54.1 (d, *J* = 9 Hz, C3), 52.3 (d, br, *J* = 1 Hz, C15), 50.0 (d, *J* = 4 Hz, C10), 48.1 (C11), 44.8 (C7), 38.5 (d, *J* = 3 Hz, C6), 31.4 (t, *J* = 5 Hz, C2), 30.7 (t, *J* = 5 Hz, C4), 28.3 (C8), 26.3 (C9), 25.0 (d, *J* = 8 Hz, C16), 19.9 (C13), 18.6 (C14), 18.3 (d, *J* = 90 Hz, C21), 13.7 (C12).

^{31}P{^{1}H} NMR spectroscopy (202 MHz, $CDCl_3$, 298 K): δ 122.7 (d, *J* = 7 Hz, P_O), 38.8 (d, *J* = 7 Hz, P_N)

^{31}P NMR spectroscopy (202 MHz, $CDCl_3$, 298 K): δ 122.7 (1P, t, *J*= 9 Hz, P_O), 38.8 (1P, m, P_N).

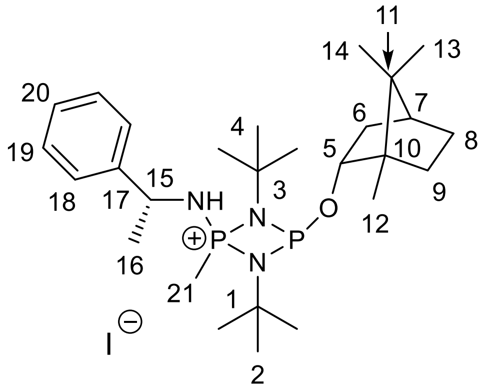

Synthesis and characterisation of [{(*S*)-$C_6H_5CH(CH_3)NH$}(Me)P(μ-N^{t}Bu)$_2$POBorn]I 5-*S*

4-*S* was prepared *in situ*, without purification from [ClPN^{t}Bu]$_2$ (0.55 g, 2 mmol) and dissolved in hexane (12 ml). MeI (1 ml, excess) was added, and the solution was stirred at room temperature overnight, during which time a thick white precipitate had formed. The volatiles were removed *in vacuo* and the residue was recrystallised from hot THF (ca. 6 ml) to give colourless crystals (270 mg, 22 %). M.P. decomp. ca. 250 °C. Elemental analysis $C_{27}H_{48}IN_3OP_2$ requires (%): C, 52.34; H, 7.81; N, 6.78. Found (%): C, 52.27; H, 7.80; N, 7.05.

^{1}H NMR spectroscopy (500 MHz, $CDCl_3$, 298 K): δ 8.41 (1H, dd, *J* = 23, 10 Hz, N–H), 7.63 (2H, d, *J* = 8 Hz, H18), 7.32 (2H, t, *J* = 8 Hz, H19), 7.24 (1H, t, *J* = 7 Hz, H20), 4.67 (1H, m, H15), 4.43 (1H, t, *J* = 10 Hz, H5), 2.47 (3H, d, *J* = 15 Hz, H21), 2.33 (1H, m, H6$_{exo}$), 2.03 (1H, m, H9$_{endo}$), 1.86 (1H, m, H8$_{exo}$), 1.78 (1H, sh, H7), 1.77 (3H, d, *J* = 7 Hz, H16), 1.49 (9H, s, H4), 1.42 (1H, m, H9$_{exo}$), 1.33 (1H, m, H8$_{endo}$), 1.25 (1H, dd, *J* = 14, 3 Hz, H6$_{endo}$), 0.96 (3H, s, H12), 0.92 (3H, s, H13), 0.91 (3H, sh, 14), 0.90 (9H, s, H2).

^{13}C NMR spectroscopy (126 MHz, $CDCl_3$, 298 K): δ 143.6 (d, *J* = 2 Hz, C17), 128.6 (C19), 127.7 (C20), 127.3 (C18), 83.4 (d, *J* = 20 Hz, C5), 54.3 (d, *J* = 8 Hz, C3), 54.1 (d, *J* = 9 Hz, C1), 52.3 (C15), 50.0 (d, *J* = 5 Hz, C10), 47.9 (C11), 44.8 (C7), 38.0 (d, *J* = 3 Hz, C6), 31.6 (t, *J* = 5 Hz, C2), 30.6 (t, *J* = 5 Hz, C4), 28.3 (C8), 26.6 (C9), 25.0 (d, *J* = 7 Hz, C16), 19.9 (C13), 18.7 (C14), 18.3 (d, J = 90 Hz, C21), 13.9 (C12).

^{31}P{^{1}H} NMR spectroscopy (202 MHz, $CDCl_3$, 298 K): δ 123.0 (d, *J* = 7 Hz, P_O), 38.8 (d, *J* = 7 Hz, P_N).

^{31}P NMR spectroscopy (202 MHz, $CDCl_3$, 298 K): δ 123.0 (1P, t, *J* = 9 Hz, P_O), 38.8 (1P, m, P_N).

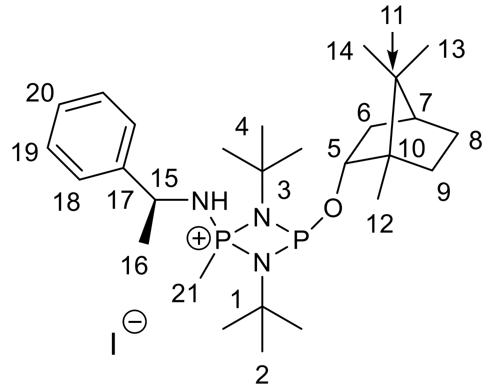

Synthesis and characterisation of [{(*R*)-$C_6H_5CH(CH_2CH_3)NH$}(Me)P(μ-N^{t}Bu)$_2$POBorn]I 6-*R*

1 was prepared *in situ*, without purification from [ClPN^{t}Bu]$_2$ (0.35 g, 1.3 mmol) and dissolved in THF (12 ml). The solution was cooled to −78 °C and triethylamine (1 ml) was added followed by (*R*)-(+)-1-phenylpropylamine (0.19 ml, 1.3 mmol). The mixture was allowed to warm to room temperature over the course of several hours. The volatiles were removed *in vacuo* and the residue was triturated with Et_2O (12 ml). The solid was digested in toluene (12 ml) and the mixture was filtered. The toluene was removed *in vacuo* and replaced with THF (12 ml). MeI (0.5 ml, excess) was added, and the solution was stirred overnight, after which time the colour changed to pale yellow. The THF was removed *in vacuo*, toluene (ca. 6 ml) was added, the solid was heated gently into solution and the solution was filtered. The solution was left to stand at room temperature for 24 h after which time colourless crystals formed (160 mg, 20%). M.P. 229–231 °C. Elemental analysis $C_{28}H_{50}IN_3OP_2$ requires (%): C, 53.08; H, 7.95; N, 6.63. Found (%): C, 52.53; H, 7.84; N, 6.44.

^{1}H NMR spectroscopy (500 MHz, $CDCl_3$, 298 K): *δ* 8.38 (1H, dd, *J* = 23, 10 Hz, N–H), 7.60 (2H, d, *J* = 7 Hz, H19), 7.31 (2H, t, *J* = 7 Hz, H 20), 7.23 (1H, t, *J* = 7 Hz, C21), 4.40 (1H, t, *J* = 10 Hz, H5), 4.32 (1H, qd, J = 11 Hz, 5 Hz, H15), 2.47 (3H, d, *J* = 15 Hz, H22), 2.43 (1H, m, H6$_{exo}$), 2.30 (1H, m, H16), 2.08 (1H, m, H16), 2.00 (1H, m, H9$_{endo}$), 1.89 (1H, m, H8$_{exo}$), 1.78 (1H, t, *J* = 4 Hz, H7), 1.47 (9H, s, H2), 1.43–1.32 (2H, m, H8$_{endo}$, H9$_{exo}$), 1.29 (1H, dd, *J* = 13, 3 Hz, H6$_{endo}$), 0.92 (3H, s, sh, H13), 0.91 (6H, s, H14, H12), 0.88 (9H, s, H4), 0.74 (3H, t, *J* = 7 Hz, H17)

^{13}C NMR spectroscopy (126 MHz, $CDCl_3$, 298 K): *δ* 141.5 (d, *J* = 2 Hz, C18), 128.4 (C20), 128.3 (C19), 127.7 (C21), 84.2 (d, *J* = 20 Hz, C5), 58.2 (d, *J* = 2 Hz, C15), 54.4 (d, J = 8 Hz, C3), 54.0 (d, *J* = 9 Hz, C1), 50. 0 (d, *J* = 4 Hz, C10), 48.1 (C11), 44.8 (C7), 38.5 (d, *J* = 3 Hz, C6), 31.4 (t, *J* = 5 Hz, C2), 31.3 (d, *J* = 7 Hz, C16), 30.7 (t, *J* = 4 Hz, C4), 28.3 (C8), 26.3 (C9), 19.9 (C13), 18.8 (C14), 18.3 (d, *J* = 90 Hz, C22), 13.7 (C12), 11.2 (C17).

^{31}P{^{1}H} NMR spectroscopy (202 MHz, $CDCl_3$, 298 K): *δ* 122.6 (d, *J* = 8 Hz, P_O), 38.8 (d, *J* = 8 Hz, P_N).

^{31}P NMR spectroscopy (202 MHz, $CDCl_3$, 298 K): *δ* 122.6 (1P, t, *J* = 9 Hz, P_O), 38.8 (1P, m, P_N).

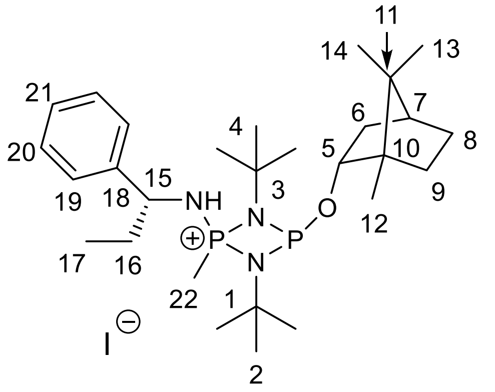

Synthesis and characterisation of [{(S)-$C_6H_5CH(CH_2CH_3)NH$}(Me)P(μ-N^tBu)$_2$POBorn]I 6-*S*

1 (390 mg, 1 mmol) was dissolved in THF (12 ml), the solution was cooled to −78 °C and Et_3N (0.5 ml, excess) was added followed by (*S*)-1-phenylpropylamine (0.15 ml, 1 mmol). The solution was stirred and warmed to room temperature over the course of several hours and then filtered. MeI (0.5 ml, excess) was added, and the solution was stirred overnight during which time the colour changed to pale yellow. Removal of the solvent gave a pale-yellow solid which was dissolved in toluene (3 ml) with heating. Standing the solution at room temperature for 1 day gave colourless crystals (152 mg, 24%). M.P. 214–216 °C. Elemental analysis $C_{28}H_{50}IN_3OP_2$ requires (%): C, 53.08; H, 7.95; N, 6.63. Found (%): C, 52.84; H, 7.97; N, 6.64.

^{1}H NMR spectroscopy (500 MHz, $CDCl_3$, 298 K): δ 8.39 (1H, dd, *J* = 22, 11 Hz, N–H), 7.60 (2H, d, *J* = 8 Hz, H19), 7.32 (2H, t, *J* = 7 Hz, H20), 7.24 (1H, t, *J* = 7 Hz, H21), 4.44 (1H, m, H5), 4.35 (1H, dd, *J* = 11, 5 Hz, H15), 2.46 (3H, d, *J* = 15 Hz, H22), 2.32 (2H, m, $H6_{exo}$, H16), 2.10 (1H, m, H16), 2.04 (1H, m, $H9_{endo}$), 1.87 (1H, m, $H8_{exo}$), 1.78 (1H, t, *J* = 4 Hz, H7), 1.49 (9H, s, H4), 1.43 (1H, m, $H9_{exo}$), 1.34 (1H, m, $H8_{endo}$), 1.27 (1H, dd, *J* = 14, 3 Hz, $H6_{endo}$), 0.96 (3H, s, H12), 0.92 (3H, s, H13), 0.91 (3H, s, H14), 0.89 (9H, s, H2), 0.74 (3H, t, *J* = 8 Hz, H17)

^{13}C NMR spectroscopy (126 MHz, $CDCl_3$, 298 K): δ 141.5 (d, *J* = 2 Hz, C18), 128.5 (C20), 128.3 (C19), 127.7 (C21), 83.5 (d, *J* = 20 Hz, C5), 58.2 (d, *J* = 2 Hz, C15), 54.3 (d, *J* = 8 Hz, C3), 54.1 (d, *J* = 9 Hz, C1), 50.0 (d, *J* = 5 Hz, C10), 47.9 (C11), 44.8 (C7), 38.0 (d, *J* =3 Hz, C6), 31.5 (t, *J* = 5 Hz, C4), 31.3 (d, *J* = 7 Hz, C16), 30.5 (t, *J* = 5 Hz, C2), 28.4 (C8), 26.5 (C9), 19.9 (C13), 18.7 (C14), 18.3 (d, *J* = 90 Hz, C22), 13.9 (C12), 11.2 (C17).

^{31}P{^{1}H} NMR spectroscopy (202 MHz, $CDCl_3$, 298 K): δ 123.1 (d, *J* = 8 Hz, P_O), 38.9 (d, *J* = 8 Hz, P_N),

^{31}P NMR spectroscopy (202 MHz, $CDCl_3$, 298 K): δ 123.1 (1P, t, *J* = 9 Hz, P_O), 38.9 (1P, m, P_N).

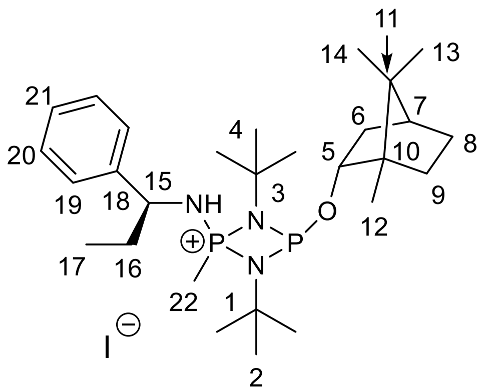

Synthesis and characterisation of {(*R*)-$C_6H_5CH(CH_3)CH_2NH$}P(μ-N^tBu)$_2$POBorn 7-*R*

1 was prepared *in situ*, without purification from [$ClPN^tBu$]$_2$ (0.28 g, 1 mmol) and dissolved in THF (12 ml). The solution was cooled to −78 °C and triethylamine (0.5 ml, excess) was added, followed by (*R*)-2-phenylpropylamine (0.15 ml, 1 mmol). The solution was allowed to warm to room temperature and stirred overnight. The solvents were removed *in vacuo*, the residue was triturated with Et_2O (12 ml), extracted into toluene (12 ml) and the mixture was filtered. The toluene was removed to give a solid, which was recrystallized from pentane (ca. 1 ml). Yield 75 mg (15%). M.P. 93–95 °C. Elemental analysis $C_{27}H_{47}N_3OP_2$ requires (%): C, 65.96; H, 9.64; N, 8.55. Found (%): C, 66.08; H, 9.77; N, 8.61.

^{1}H NMR spectroscopy (500 MHz, $CDCl_3$, 298 K): *δ* 7.30 (2 H, t, *J* = 8 Hz, H20), 7.23–7.16 (3H, m, H19, H21), 4.30 (1H, m, H5), 3.50 (1H, m, H15), 3.06 (1H, m, H15), 2.83–2.64 (2H, m, H16, N–H), 2.18 (1H, m, $H6_{exo}$), 2.05 (1H, m, $H9_{endo}$), 1.68 (1H, m, $H8_{exo}$), 1.60 (1H, t, *J* = 5 Hz, H7), 1.29 (9H, s, H2/H4), 1.28–1.24 (12H, d + s, H17, H2/H4), 1.23–1.11 (3H, m, br, $H6_{endo}$, $H9_{exo}$, $H8_{endo}$), 0.85 (3H, s, H13), 0.83 (3H, s, H12), 0.82 (3H, s, H14).

^{13}C NMR spectroscopy (126 MHz, $CDCl_3$, 298 K): *δ* 145.5 (C18), 128.5 (C19), 127.3 (C20) 126.2 (C21), 78.0 (C5), 51.1 (m, C1,C3), 49.6 (d, *J* = 4 Hz, C10), 47.4 (C11), 45.9 (br, C15), 45.2 (C7), 42.3 (d, *J* = 2 Hz, C16), 38.9 (d, *J* = 2 Hz, C6), 31.3 (t, *J* = 7 Hz, C2/C4), 31.0 (t, *J* = 7 Hz, C2/C4), 28.2 (C8), 27.0 (C9), 20.1 (C13), 19.9 (C17), 18.9 (C14), 13.8 (C12).

^{31}P{^{1}H} NMR spectroscopy (202 MHz, $CDCl_3$, 298 K): *δ* 138 (br, P_O), 103 (br, P_N).

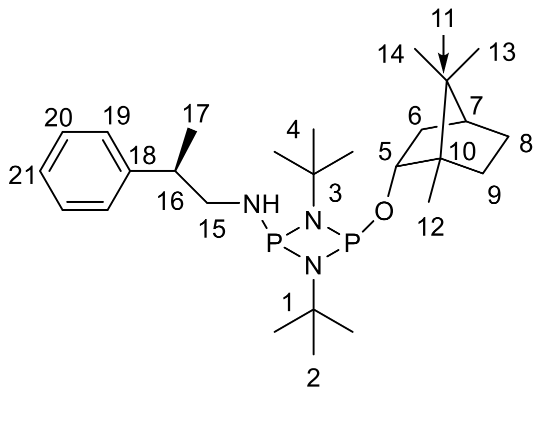

Synthesis and characterisation of {(*S*)-$C_6H_5CH(CH_3)CH_2NH$}P(μ-N^tBu)$_2$POBorn 7-*S*

1 was prepared *in situ*, without purification from [$ClPN^tBu$]$_2$ (0.28 g, 1 mmol) and dissolved in THF (12 ml). The solution was cooled to −78 °C and triethylamine (0.5 ml, excess) was added, followed by (*S*)-2-phenylpropylamine (0.15 ml, 1 mmol). The solution was allowed to warm to room temperature and stirred overnight. The solvents were removed *in vacuo*, the residue was triturated with Et_2O (12 ml), extracted into toluene (12 ml) and the mixture was filtered. The toluene was removed to give a solid, which was recrystallized from pentane (ca. 1 ml). Yield 95 mg (19%). M.P. 88–90 °C. Elemental analysis $C_{27}H_{47}N_3OP_2$ requires (%): C, 65.96; H, 9.64; N, 8.55. Found (%): C, 66.01; H, 9.66; N, 8.82.

^{1}H NMR spectroscopy (500 MHz, $CDCl_3$, 298 K): *δ* 7.30 (2H, t, *J* = 8 Hz, H20), 7.25–7.15 (3H, m, H19, H21), 4.30 (1H, m, H5), 3.50 (1H, m, H15), 3.06 (1H, m, H15), 2.82–2.69 (2H, m, H16, N–H), 2.14 (1H, m, $H6_{exo}$), 2.05 (1H, m, $H9_{endo}$), 1.68 (1H, m, $H8_{exo}$), 1.59 (1H, t, *J* = 4 Hz, H7) 1.29 (9H, s, H2/H4), 1.28 (1H, sh, H17), 1.26 (11H, s, H17, H2/H4), 1.22–1.09 (3H, m, $H6_{endo}$, $H9_{exo}$, $H8_{endo}$), 0.85 (3H, s, H13), 0.83 (3H, s, H12), 0.81 (3H, s, H14).

^{13}C NMR spectroscopy (126 MHz, $CDCl_3$, 298 K): *δ* 145.4 (C18), 128.5 (C20), 128.2 (C19), 126.2 (C21), 78.0 (C5), 51.3 (t, *J* = 13 Hz, C1/C3), 51.0 (t, *J* = 13 Hz, C1/C3), 49.6 (d, *J* = 4 Hz, C10), 47.3 (C11), 46.2 (br, C15), 45.2 (C7), 42.4 (C16), 49.0 (C6), 31.2 (m, C2/C4), 28.3 (C8), 27.0 (C9), 20.1 (C13), 19.9 (C17), 18.9 (C14), 13.8 (C12).

^{31}P{^{1}H} NMR spectroscopy (202 MHz, $CDCl_3$, 298 K): *δ* 138 (P_O), 103 (P_N).

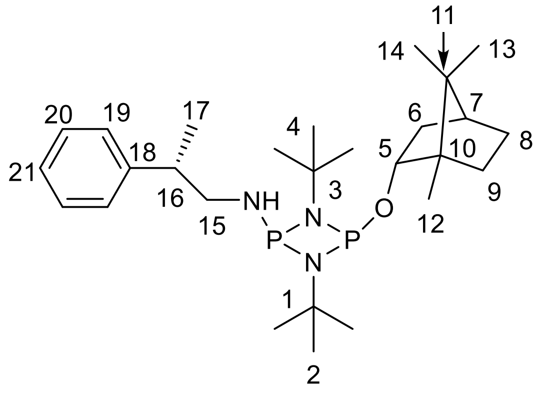

Synthesis and characterisation of {(*R/S*)-$C_6H_5CH(CH_3)CH_2NH$}P(μ-N^{t}Bu)$_2$POBorn 7-*R/S*

1 was prepared *in situ*, without purification from [ClPN^{t}Bu]$_2$ (0.28 g, 1 mmol) and dissolved in THF (12 ml). The solution was cooled to −78 °C and triethylamine (0.5 ml, excess) was added, followed by (*R/S*)-2-phenylpropylamine (0.15 ml, 1 mmol). The solution was allowed to warm to room temperature and stirred overnight. The solvents were removed *in vacuo*, the residue was triturated with Et_2O (12 ml), extracted into toluene (12 ml) and the mixture was filtered. The toluene was removed to give a solid, which was recrystallised from hot hexane (ca. 0.5 ml) containing a few drops of toluene (110 mg, 23%). M.P. 79–81 °C. Elemental analysis $C_{27}H_{47}N_3OP_2$ requires (%): C, 65.96; H, 9.64; N, 8.55. Found (%): C, 65.77; H, 9.61; N, 8.55.

^{1}H NMR spectroscopy (500 MHz, $CDCl_3$, 298 K): δ 7.30 (2H, t, *J* = 8 Hz, H20), 7.25–7.16 (3H, m, H19, H21), 4.30 (1H, m, H5), 3.51 (1H, m, H15), 3.06 (1H, m, H15), 2.82–2.62 (2H, m, H16, N–H), 2.15 (1H, m, H6$_{exo}$), 2.04 (1H, m, H9$_{endo}$), 1.68 (1H, m, H8$_{exo}$), 1.60 (1H, q, *J* = 5 Hz, H7), 1.30 (4.5H, s, H2/H4), 1.29 (4.5H, s, H2/H4), 1.26 (3H, sh, H17), 1.26 (4.5H, s, H2/H4), 1.25 (4.5H, s, H2/H4), 1.23–1.10 (3H, m, br, H6$_{endo}$, H9$_{exo}$, H8$_{endo}$), 0.90–0.80 (br, H12, H13, H14).

^{13}C NMR spectroscopy (126 MHz, $CDCl_3$, 298 K): δ 145.5, 145.4 (C18); 128.5 (C20), 127.3 (C19), 126.2 (C21), 78.0 (C5), 51.5–50.8 (C1, C3), 49.6 (C10), 47.4–47.3 (C11), 45.9 (C15), 45.2 (C7), 42.4, 42.3 (C16); 39.0 (C6), 31.5–30.9 (C2/C4), 28.3, 28.2 (C8); 27.0 (C9), 20.1 (C13), 19.9 (C17), 18.9 (C14), 13.8 (12).

^{31}P{^{1}H} NMR spectroscopy (202 MHz, $CDCl_3$, 298 K): δ 138 (br, P$_O$), 103 (P$_N$).

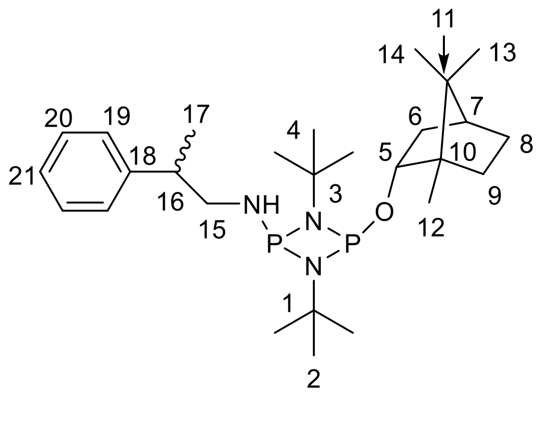

Synthesis and characterisation of [{(*R*)-$C_6H_5CH(CH_3)CH_2NH$}(Me)P(μ-N^tBu)$_2$POBorn]I 8-*R*

1 was prepared *in situ*, without purification, from [ClPN^{t}Bu]$_2$ (0.28 g, 1 mmol) and dissolved in THF (12 ml). The solution was cooled to −78 °C and triethylamine (0.5 ml, excess) was added, followed by (*R*)-2-phenylpropylamine (0.15 ml, 1 mmol). The solution was allowed to warm to room temperature and stirred overnight. The solvents were removed *in vacuo*, the residue was triturated with Et_2O (12 ml) and digested in toluene. The toluene was removed *in vacuo* and the solid was dissolved in THF (12 ml). MeI (0.5 ml, excess) was added at room temperature and the mixture was stirred overnight, during which time the colourless solution became pale yellow. All volatiles were removed to leave a foamy solid, which was triturated with hexane (6 ml). The residue was recrystallised from hot toluene to give large colourless crystals (150 mg, 24%). M.P. 198–200 °C Elemental analysis $C_{28}H_{50}IN_3OP_2$ requires (%): C, 53.08; H, 7.95; N, 6.63. Found (%):C, 52.80; H, 7.99; N, 6.76.

^{1}H NMR spectroscopy (500 MHz, $CDCl_3$, 298 K): δ 7.72 (1H, dq, *J* = 20, 4 Hz, N–H), 7.30–7.25 (4H, br, H19, H20), 7.21–7.15 (1H, br, H21), 4.33 (1H, m, H5), 3.36 (1H, m, H15), 3.26 (1H, m, H16, 3.19 (1H, m, H15), 2.49 (1H, d, *J* = 15 Hz, H22), 2.30 (1H, m, $H6_{exo}$), 1.76–1.65 (3H, m, $H9_{endo}$, $H8_{exo}$, H7), 1.46 (3H, d, *J* = 7 Hz, H17), 1.41 (9H, s, H2/H4), 1.29–1.16 (1H, m, $H8_{endo}$) 1.23 (9H, s, H2/H4), 1.08 (1H, dd, *J* = 13, 3 Hz, $H6_{endo}$), 1.02 (1H, m, $H9_{exo}$), 0.86 (6H, s, H14, H13), 0.81 (3H, s, H12).

^{13}C NMR spectroscopy (126 MHz, $CDCl_3$, 298 K): δ 144.0 (C18), 128.6 (C20), 127.3 (C19), 126.7 (C21), 82.8 (d, *J* = 17 Hz, C5), 54.2 (d, *J* = 8 Hz, C1/C3), 54.1 (d, *J* = 7 Hz, C1/C3), 49.9 (d, *J* = 5 Hz, C10), 47.7 (C11), 47.7 (C15), 44.8 (C7), 39.9 (d, *J* = 8 Hz, C16), 38.2 (d, *J* = 2 Hz, C6), 31.4 (t, *J* = 5 Hz, C2/C4), 31.1 (t, *J* = 5 Hz, C2/C4), 28.0 (C8), 26.4 (C9), 20.1 (C17), 19.9 (C13), 18.7 (C14), 18.0 (d, *J* = 89 Hz, C22), 13.7 (C12).

^{31}P{^{1}H} NMR spectroscopy (202 MHz, $CDCl_3$, 298 K): δ 124.3 (d, *J* = 8 Hz, P_O), 45.7 (d, *J* = 8 Hz, P_N).

^{31}P NMR spectroscopy (202 MHz, $CDCl_3$, 298 K): δ 124.3 (1P, t, *J* = 9 Hz, P_O), 45.7 (1P, m, P_N).

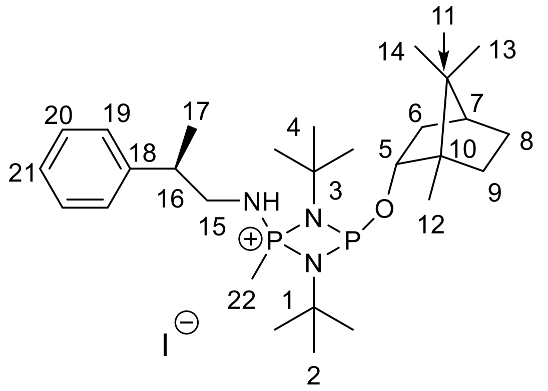

Synthesis and characterisation of [{(*S*)-$C_6H_5CH(CH_3)CH_2NH$}(Me)P(μ-N^tBu)$_2$POBorn]I 8-*S*

1 was prepared *in situ*, without purification, from [ClPN^{t}Bu]$_2$ (0.28 g, 1 mmol) and dissolved in THF (12 ml). The solution was cooled to −78 °C and triethylamine (0.5 ml, excess) was added, followed by (*S*)-2-phenylpropylamine (0.15 ml, 1 mmol). The solution was allowed to warm to room temperature and stirred overnight. The solvents were removed *in vacuo*, the residue was triturated with Et_2O (12 ml) and digested in toluene. The toluene was removed *in vacuo* and the solid was dissolved in THF (12 ml). MeI (0.5 ml, excess) was added at room temperature and the mixture was stirred overnight, during which time the colourless solution became pale yellow. All volatiles were removed to leave a foamy solid, which was triturated with hexane (6 ml). The residue was recrystallised from hot toluene to give large colourless crystals (80 mg, 13%). M.P. 193–195 °C. Elemental analysis $C_{28}H_{50}IN_3OP_2$ requires (%): C, 53.08; H, 7.95; N, 6.63. Found (%):C, 52.71; H, 7.99; N, 6.74.

^{1}H NMR spectroscopy (500 MHz, $CDCl_3$, 298 K): δ 7.72 (1H, dq, 20, 4 Hz, N–H), 7.30–7.25 (4H, m, H19, H20), 7.18 (1H, m, H21), 4.32 (1H, m, H5), 3.35 (1H, m, H15), 3.26 (1H, m, H16), 3.18 (1H, m, H15), 2.49 (3H, d, *J* = 15 Hz, H22), 2.32 (1H, m, H6$_{exo}$), 1.78–1.65 (3H, m, br, H9$_{endo}$, H8$_{exo}$, H7), 1.47 (3H, d, *J* = 9 Hz, H17), 1.41 (9H, s, H2/H4), 1.23 (9H, s, H2/H4), 1.22–1.16 (1H, m, H9$_{exo}$), 1.15 (1H, dd, *J* = 13, 3 Hz, H6$_{endo}$), 1.07 (1H, m, H8$_{endo}$), 0.86 (3H, s, H13), 0.86 (3H, s, H14), 0.79 (3H, s, H12).

^{13}C NMR spectroscopy (126 MHz, $CDCl_3$, 298 K): δ 144.0 (C18), 128.6 (C20), 127.3 (C19), 126.7 (C21), 82.8 (d, *J* = 18 Hz, C5), 54.1 (d, *J* = 8 Hz, C1/C3), 53.9 (d, *J* = 8 Hz, C1/C3), 49.9 (d, *J* = 4 Hz, C10), 47.8 (C11), 47.8 (C15), 44.8 (C7), 39.8 (d, *J* = 8 Hz, C16), 38.2 (d, *J* = 3 Hz, C6), 31.3 (t, *J* = 5 Hz, C2,C4), 28.1 (C8), 26.4 (C9), 20.1 (C17), 19.9 (C13), 18.7 (C14), 18.0 (C22), 13.7 (C12).

^{31}P{^{1}H} NMR spectroscopy (202 MHz, $CDCl_3$, 298 K): δ 124.3 ("*R*" P_O), 124.2 ("*S*" P_O), 45.7 ("*R*" P_N), 45.7 ("*S*" P_N).

^{31}P NMR spectroscopy (202 MHz, $CDCl_3$, 298 K): δ 124.3 (1P, t, *J* = 9 Hz, P_O), 45.7 (1P, m, P_N).

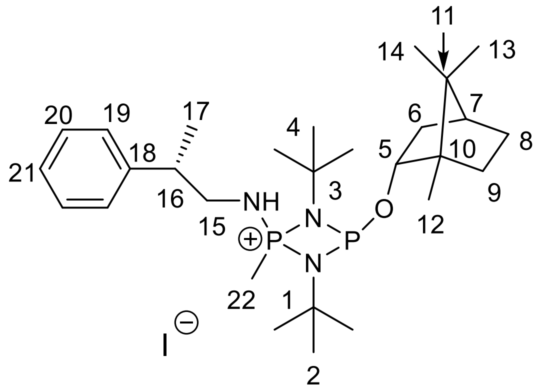

Synthesis and characterisation of [{(*R*/*S*)-$C_6H_5CH(CH_3)CH_2NH$}(Me)P(μ-N^{t}Bu)$_2$POBorn]I 8-*R*/*S*

1 was prepared *in situ*, without purification, from [ClPN^{t}Bu]$_2$ (0.28 g, 1 mmol) and dissolved in THF (12 ml). The solution was cooled to −78 °C and triethylamine (0.5 ml, excess) was added, followed by (*R*/*S*)-2-phenylpropylamine (0.15 ml, 1 mmol). The solution was allowed to warm to room temperature and stirred overnight. The solvents were removed *in vacuo*, the residue was triturated with Et_2O (12 ml), extracted into toluene (12 ml) and the mixture was filtered. The toluene was removed *in vacuo* and the solid was re-dissolved in THF (12 ml). MeI (0.5 ml, excess), was added at room temperature and the mixture was stirred overnight. Removal of the volatiles gave a pale yellow foamy solid. The solid was recrystallised from hot toluene (ca. 3ml) to give colourless crystals (147 mg, 23 %). M.P. 188–190 °C. Elemental analysis $C_{28}H_{50}IN_3OP_2$ requires (%): C, 53.08; H, 7.95; N, 6.63. Found (%): C, 53.20; H, 8.03; N, 6.64.

^{1}H NMR spectroscopy (500 MHz, $CDCl_3$, 298 K): δ 7.70 (1H, m, N–H), 7.40–7.20 (2H, m, br, H19, H20), 7.18 (1H, m, br, H21), 4.32 (1H, m, H5), 3.36 (1H, m, H15), 3.26 (1H, m, H16), 3.18 (1H, m, H15), 2.49 (d, *J* = 15 Hz, H22), 2.31 (1H, m, H6$_{exo}$), 1.78–1.63 (3H, m, H9$_{endo}$, H8$_{exo}$, H7), 1.48–1.44 (3H, m, H17), 1.40 (9H, s, H2/H4), 1.29–1.16 (1H, m, br, H8$_{endo}$), 1.23 (9H, s, H2/H4), 1.15–0.95 (2H, m, H6$_{endo}$, H9$_{exo}$), 0.86 (6H, s, H13, H14), 0.79 (3H, s, H12).

^{13}C NMR spectroscopy (126 MHz, $CDCl_3$, 298 K): δ 144.0 (C18), 128.6 (C20), 127.3 (C19), 126.7 (C21), 82.8 (C5), 54.3–53.9 (C1, C3), 49.9 (C10), 47.8 (C11, C15), 44.8 (C7), 39.9 (C16), 38.2 (C6), 31.5–31.0 (C2/C4), 28.1, 28.0 (C8); 26.4 (C9), 20.1 (C17), 19.9 (C13), 18.7 (C14), 18.0 (C22), 13.7 (C12).

^{31}P{^{1}H} NMR spectroscopy (202 MHz, $CDCl_3$, 298 K): δ 124.3 (0.53P, d, *J* = 8 Hz, "*R*" P$_o$), 124.2 (0.45P, d, *J* = 8 Hz, "*S*" P$_O$), 45.7 (0.54P, d, *J* = 8 Hz, "*R*" P$_N$), 45.7 (0.46P, d, *J* = 8 Hz, "*S*" P$_N$).

^{31}P NMR spectroscopy (202 MHz, $CDCl_3$, 298 K): δ 124.3 (1P, m, P$_O$), 45.7 (1P, m, P$_N$).

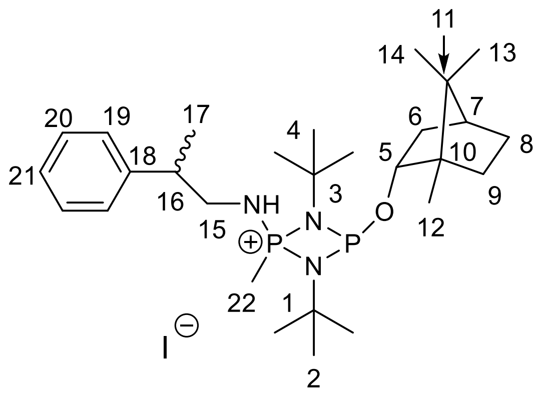

Synthesis and characterisation of [{(*R*)- $C_{10}H_7CH(CH_3)NH$}(Me)P(μ-N^tBu)$_2$POBorn]I 9-*R*

Previously prepared **1** (0.39 g, 1 mmol) was dissolved in THF (3 ml). The solution was cooled to −78 °C and triethylamine (0.5 ml, excess) was added, followed by a solution of (*R*)-1-(2-naphthyl)ethylamine (0.17 g, 1 mmol) in THF (3 ml). The reaction mixture was allowed to warm to room temperature and stirred overnight. The solvents were removed *in vacuo*, the residue was triturated with Et_2O (6 ml), extracted into toluene (12 ml) and the mixture was filtered. The toluene was removed *in vacuo* and the solid was dissolved in THF (12 ml). MeI (0.5 ml, excess) was added at room temperature and the mixture was stirred overnight, during which time the colourless solution became pale yellow. All volatiles were removed to leave a cream powder. The solid was purified *via* digestion in hot toluene (ca. 4 ml) and precipitation upon the dropwise addition of pentane to yield a white acicular material (290 mg, 43%). M.P. 158–160 °C. Elemental analysis $C_{31}H_{50}IN_3OP_2$ requires (%): C, 55.61; H, 7.53; N, 6.28. Found (%): C, 55.40; H, 7.48; N, 6.00.

^{1}H NMR spectroscopy (500 MHz, $CDCl_3$, 298 K): 8.57 (1H, dd, *J* = 21, 11 Hz, N–H), 7.99–7.95 (2H, m, H21, H24), 7.88 (1H, d, *J* = 8 Hz, H19), 7.85–7.80 (2H, m, H22, H26), 7.49–7.46 (2H, m, H18, H23), 4.88 (1H, m, H15), 4.47 (1H, tq, *J* = 10, 2 Hz, H5), 2.53 (3H, d, *J* = 15 Hz, H27), 2.49 (1H, m, $H6_{exo}$), 2.14 (1H, m, $H9_{endo}$), 2.00 (1H, m, $H8_{exo}$), 1.86 (3H, d, *J* = 7 Hz, H16), 1.79 (1H, t, *J* = 4 Hz, H7), 1.54 (9H, s, H2/H4), 1.48–1.36 (3H, m, $H6_{endo}$, $H8_{endo}$, $H9_{exo}$), 0.99 (3H, s, H12), 0.96 (6H, s, H13/H14), 0.85 (9H, s, H2/H4).

^{13}C NMR spectroscopy (126 MHz, $CDCl_3$, 298 K): 141.1 (d, *J* = 2 Hz, C17), 133.1 (C25), 132.8 (C20), 128.9 (C18), 127.8 (C21), 127.6 (C24), 126.3 (C19), 125.9 (C23), 125.4 (C22), 125.3 (C26), 83.2 (d, *J* = 19 Hz, C5), 54.4 (d, *J* = 8 Hz, C1/C3), 54.1 (d, *J* = 9 Hz, C1/C3), 52.4 (d, *J* = 2 Hz, C15), 50.1 (d, *J* = 5 Hz, C10), 48.2 (C11), 44.8 (C7), 38.6 (d, *J* = 3 Hz, C6), 31.5 (t, *J* = 5 Hz, C2/C4), 30.7 (t, *J* = 5 Hz, C2/C4), 28.1 (C8), 26.5 (C9), 25.2 (d, *J* = 7 Hz, C16), 20.0 (C13), 18.8 (C14), 18.3 (d, *J* = 89 Hz, C27), 13.8 (C12).

^{31}P{^{1}H} NMR spectroscopy (202 MHz, $CDCl_3$, 298 K): δ 122.6 (d, *J* = 8 Hz, P_O), 39.2 (d, *J* = 8 Hz, P_N).

^{31}P NMR spectroscopy (202 MHz, $CDCl_3$, 298 K): δ 122.6 (1P, t, *J* = 9 Hz, P_O), 39.2 (1P, m, P_N).

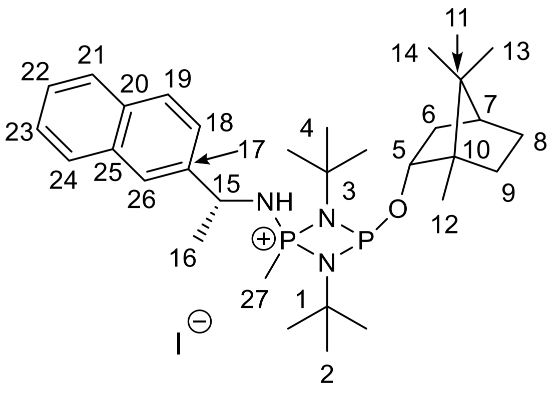

Synthesis and characterisation of [{(*S*)- $C_{10}H_7CH(CH_3)NH$}(Me)P(μ-N^tBu)$_2$POBorn]I 9-*S*

Previously prepared **1** (0.39 g, 1 mmol) was dissolved in THF (3 ml). The solution was cooled to −78 °C and triethylamine (0.5 ml, excess) was added, followed by a solution of (*S*)-1-(2-naphthyl)ethylamine (0.17 g, 1 mmol) in THF (3 ml). The reaction mixture was allowed to warm to room temperature and stirred overnight. The solvents were removed *in vacuo*, the residue was triturated with Et_2O (6 ml), extracted into toluene (12 ml) and the mixture was filtered. The toluene was removed *in vacuo* and the solid was dissolved in THF (12 ml). MeI (0.5 ml, excess) was added at room temperature and the mixture was stirred overnight, during which time the colourless solution became pale yellow. All volatiles were removed to leave a pale yellow powder. The solid was purified *via* digestion in hot toluene (ca. 6 ml) and precipitation upon the dropwise addition of pentane to yield a white powdery material (330 mg, 49%). M.P. 150–152 °C. Elemental analysis $C_{31}H_{50}IN_3OP_2$ requires (%): C, 55.61; H, 7.53; N, 6.28. Found (%): C, 54.89; H, 7.51; N, 5.99.

^{1}H NMR spectroscopy (500 MHz, $CDCl_3$, 298 K): 8.57 (1H, dd, *J* = 21, 11 Hz, N–H), 8.02–7.93 (2H, m, H21, H24), 7.90 (1H, d, *J* = 8 Hz, H19), 7.86–7.81 (2H, m, H22, H26), 7.51–7.47 (2H, m, H18, H23), 4.90 (1H, m, H15), 4.49 (1H, tq, *J* = 10, 2 Hz, H5), 2.53 (3H, d, *J* = 15 Hz, H27), 2.39 (1H, m, $H6_{exo}$), 2.15 (1H, m, $H9_{endo}$), 1.97 (1H, m, $H8_{exo}$), 1.88 (3H, d, *J* = 7 Hz, H16), 1.79 (1H, t, *J* = 4 Hz, H7), 1.55 (9H, s, H2/H4), 1.48–1.36 (3H, m, $H6_{endo}$, $H8_{endo}$, $H9_{exo}$), 1.04 (3H, s, H12), 0.99 (3H, s, H13/H14), 0.96 (3H, s, H13/H14), 0.85 (9H, s, H2/H4).

^{13}C NMR spectroscopy (126 MHz, $CDCl_3$, 298 K): 141.1 (d, *J* = 2 Hz, C17), 133.0 (C25), 132.8 (C20), 128.9 (C18), 127.8 (C21), 127.6 (C24), 126.3 (C19), 125.9 (C23), 125.4 (C22), 125.3 (C26), 83.4 (d, *J* = 19 Hz, C5), 54.3 (d, *J* = 8 Hz, C1/C3), 54.2 (d, *J* = 9 Hz, C1/C3), 52.4 (d, *J* = 2 Hz, C15), 50.1 (d, *J* = 5 Hz, C10), 48.0 (C11), 44.8 (C7), 38.1 (d, *J* = 3 Hz, C6), 31.6 (t, *J* = 5 Hz, C2/C4), 30.6 (t, *J* = 5 Hz, C2/C4), 28.1 (C8), 26.7 (C9), 25.2 (d, *J* = 7 Hz, C16), 20.0 (C13), 18.8 (C14), 18.3 (d, *J* = 89 Hz, C27), 13.9 (C12).

^{31}P{^{1}H} NMR spectroscopy (202 MHz, $CDCl_3$, 298 K): δ 122.9 (d, *J* = 8 Hz, P_O), 39.2 (d, *J* = 8 Hz, P_N).

^{31}P NMR spectroscopy (202 MHz, $CDCl_3$, 298 K): δ 122.9 (1P, t, *J* = 9 Hz, P_O), 39.2 (1P, m, P_N).

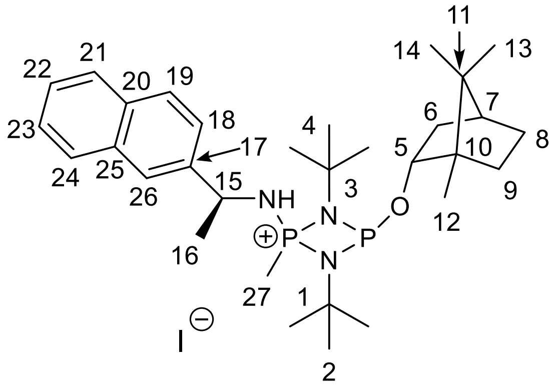

Synthesis and characterisation of [{(*R*)-$C_6H_{11}CH(CH_3)NH$}(Me)P(μ-N^tBu)$_2$POBorn]I 10-*R*

Previously prepared **1** (0.39 g, 1 mmol) was dissolved in THF (3 ml). The solution was cooled to −78 °C and triethylamine (0.5 ml, excess) was added, followed by a solution of (*R*)-1-cyclohexylethylamine (127 mg, ca. 0.15 ml, 1 mmol) in THF (3 ml). The reaction mixture was allowed to warm to room temperature and stirred overnight. The solvents were removed *in vacuo*, the residue was triturated with Et_2O (6 ml), extracted into toluene (12 ml) and the mixture was filtered. The toluene was removed *in vacuo* and the solid was dissolved in THF (12 ml). MeI (0.5 ml, excess) was added at room temperature and the mixture was stirred overnight. Removal of the volatiles gave a white powder. The solid was recrystallised from hot toluene (ca. 4 ml) to give colourless plate-like crystals (120 mg, 19%). A second crop of crystal was obtained from the supernatant (200 mg, 32%). Combined yield of 320 mg, 51%. M.P. 192–194 °C. Elemental analysis $C_{27}H_{54}IN_3OP_2$ requires (%): C, 51.84; H, 8.70; N, 6.72. Found (%):C, 51.42; H, 8.77; N, 6.64.

^{1}H NMR spectroscopy (500 MHz, $CDCl_3$, 298 K): 7.51 (1H, dd, *J* = 22, 10 Hz, N–H), 4.38 (1H, tq, *J* = 10, 2 Hz, H5), 3.60 (1H, m, H15), 2.55 (3H, d, *J* = 15 Hz, H21), 2.37 (1H, m, $H6_{exo}$), 1.90–1.77 (3H, m, H17, $H9_{endo}$, $H8_{exo}$), 1.74 (1H, t, *J* = 4 Hz, H7), 1.76–1.52 (5H, m, $H18_{eq}$, $H19_{eq}$, $H20_{eq}$), 1.45 (9H, s, H2/H4), 1.43 (9H, s, H2/H4), 1.35 (3H, d, *J* = 7 Hz, H16), 1.33–1.07 (8H, m, $H6_{endo}$, $H18_{ax}$, $H19_{ax}$, $H20_{ax}$, $H9_{exo}$, $H8_{endo}$), 0.91 (3H, s, H13/H14), 0.90 (3H, s, H13/H14), 0.88 (3H, s, H12).

^{13}C NMR spectroscopy (126 MHz, $CDCl_3$, 298 K): 82.7 (d, *J* = 18 Hz, C5), 54.4 (d, *J* = 9 Hz, C1/C3), 54.2 (d, *J* = 9 Hz, C1/C3), 52.9 (d, *J* = 3 Hz, C15), 49.9 (d, *J* = 5 Hz, C10), 47.8 (C11), 44.8 (C7), 44.2 (d, *J* = 3 Hz, C17), 38.2 (d, *J* = 3 Hz, C6), 31.5 (t, *J* = 5 Hz, C2/C4), 31.3 (t, *J* = 5 Hz, C2/C4), 30.8 (C18), 28.1 (C8), 26.6 (C19), 26.4 (C9), 26.1 (C20), 19.9 (C13), 18.7 (C14), 18.7 (d, *J* = 89 Hz, C21), 16.3 (d, *J* = 6 Hz, C16), 13.6 (C12).

^{31}P{^{1}H} NMR spectroscopy (202 MHz, $CDCl_3$, 298 K): δ 122.2 (d, *J* = 8 Hz, P_O), 41.2 (d, *J* = 8 Hz, P_N).

^{31}P NMR spectroscopy (202 MHz, $CDCl_3$, 298 K): δ 122.2 (1P, t, *J* = 9 Hz, P_O), 41.2 (1P, m, P_N).

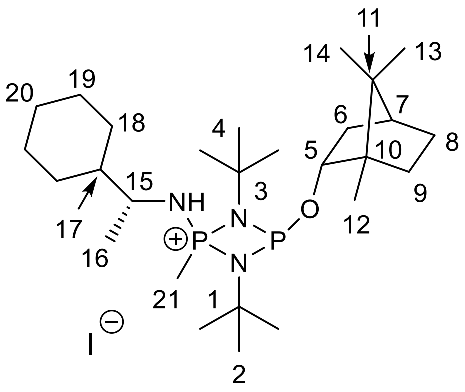

Synthesis and characterisation of [{(*S*)- $C_6H_{11}CH(CH_3)NH$}(Me)P(μ-N^tBu)$_2$POBorn]I 10-*S*

Previously prepared **1** (0.39 g, 1 mmol) was dissolved in THF (3 ml). The solution was cooled to −78 °C and triethylamine (0.5 ml, excess) was added, followed by a solution of (*S*)-1-cyclohexylethylamine (127 mg, ca. 0.15 ml, 1 mmol) in THF (3 ml). The reaction mixture was allowed to warm to room temperature and stirred overnight. The solvents were removed *in vacuo*, the residue was triturated with Et_2O (6 ml), extracted into toluene (12 ml) and the mixture was filtered. The toluene was removed *in vacuo* and the solid was dissolved in THF (12 ml). MeI (0.5 ml, excess) was added at room temperature and the mixture was stirred overnight. Removal of the volatiles gave a white powder. The solid was recrystallised from hot toluene (ca. 4 ml) to give colourless plate-like crystals (120 mg, 19%). A second crop of crystal was obtained from the supernatant (220 mg, 35%). Combined yield of 340 mg, 54%. M.P. 184–186 °C. Elemental analysis $C_{27}H_{54}IN_3OP_2$ requires (%): C, 51.84; H, 8.70; N, 6.72. Found (%):C, 51.25; H, 8.80; N, 6.56.

^{1}H NMR spectroscopy (500 MHz, $CDCl_3$, 298 K): 7.50 (1H, dd, *J* = 22, 10 Hz, N–H), 4.39 (1H, tq, *J* = 10, 2 Hz, H5), 3.61 (1H, m, H15), 2.55 (3H, d, *J* = 15 Hz, H21), 2.36 (1H, m, H6$_{exo}$), 1.89–1.76 (3H, m, H17, H9$_{endo}$, H8$_{exo}$), 1.74 (1H, t, *J* = 4 Hz, H7), 1.72–1.54 (5H, m, H18$_{eq}$, H19$_{eq}$, H20$_{eq}$), 1.45 (9H, s, H2/H4), 1.43 (9H, s, H2/H4), 1.35 (3H, d, *J* = 7 Hz, H16), 1.33–1.06 (8H, m, H6$_{endo}$, H18$_{ax}$, H19$_{ax}$, H20$_{ax}$, H9$_{exo}$, H8$_{endo}$), 0.91 (3H, s, H13/H14), 0.90 (3H, s, H13/H14), 0.89 (3H, s, H12).

^{13}C NMR spectroscopy (126 MHz, $CDCl_3$, 298 K): 82.8 (d, *J* = 18 Hz, C5), 54.4 (d, *J* = 9 Hz, C1/C3), 54.2 (d, *J* = 9 Hz, C1/C3), 52.9 (d, *J* = 3 Hz, C15), 49.9 (d, *J* = 5 Hz, C10), 47.7 (C11), 44.8 (C7), 44.2 (d, *J* = 3 Hz, C17), 38.1 (d, *J* = 3 Hz, C6), 31.5 (t, *J* = 5 Hz, C2/C4), 31.3 (t, *J* = 5 Hz, C2/C4), 30.8 (C18), 28.1 (C8), 26.8 (C19), 26.4 (C9), 26.1 (C20), 19.9 (C13), 18.7 (C14), 18.7 (d, *J* = 89 Hz, C21), 16.3 (d, *J* = 6 Hz, C16), 13.6 (C12).

^{31}P{^{1}H} NMR spectroscopy (202 MHz, $CDCl_3$, 298 K): *δ* 122.5 (d, *J* = 8 Hz, P$_O$), 41.2 (d, *J* = 8 Hz, P$_N$).

^{31}P NMR spectroscopy (202 MHz, $CDCl_3$, 298 K): *δ* 122.5 (1P, t, *J* = 9 Hz, P$_O$), 41.2 (1P, m, P$_N$).

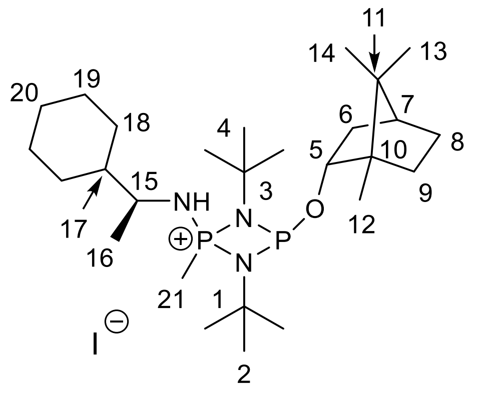

Synthesis and characterisation of [{(*R*)-$C_{10}H_{11}NH$}(Me)P(μ-N^tBu)$_2$POBorn]I 11-*R*

Previously prepared **1** (0.28 g, 0.7 mmol) was dissolved in THF (4 ml). The solution was cooled to −78 °C and triethylamine (0.4 ml, excess) was added, followed by freshly distilled (*R*)-1,2,3,4-tetrahydro-1-naphthylamine (0.10 ml, *ca* 103 mg, 0.7 mmol). The reaction mixture was allowed to warm to room temperature and stirred overnight. The solvents were removed *in vacuo*, the residue was triturated with Et_2O (5 ml), extracted into toluene (10 ml) and the mixture was filtered. The toluene was removed *in vacuo* and the solid was dissolved in THF (8 ml). MeI (0.4 ml, excess) was added at room temperature and the mixture was stirred overnight, during which time the colourless solution turned pale yellow. Removal of the volatiles gave a yellow-cream powder. The solid was purified *via* digestion in hot toluene (ca. 4 ml) and precipitation upon the dropwise addition of pentane to yield a pale yellow powder (350 mg, 78%). M.P. 96–98 °C. Elemental analysis $C_{29}H_{50}IN_3OP_2$ requires (%): C, 53.95; H, 7.81; N, 6.51. Found (%): C, 53.69; H, 7.73; N, 6.21.

^{1}H NMR spectroscopy (500 MHz, $CDCl_3$, 298 K): 7.60 (1H, dd, *J* = 21, 10 Hz, N–H), 7.29–7.11 (4H, m, H20, H21, H22, H23), 4.78 (1H, m, H15), 4.45 (1H, tq, *J* = 10, 2 Hz, H5), 3.05–2.70 (2H, m, $H18_{ax}$, $H18_{eq}$), 2.67 (3H, d, *J* = 15 Hz, H25), 2.39 (2H, m, $H16_{ax}$, $H6_{exo}$), 2.23 (2H, m, $H16_{eq}$, $H17_{ax}$), 1.94–1.78 (3H, m, $H17_{eq}$, $H9_{endo}$, $H8_{exo}$), 1.76 (1H, t, *J* = 4 Hz, H7), 1.50 (9H, s, H2/H4), 1.38 (9H, s, H2/H4), 1.41–1.21 (3H, m, $H6_{endo}$, $H9_{exo}$, $H8_{endo}$), 0.93 (3H, s, H13/H14), 0.92 (3H, s, H13/H14), 0.91 (3H, s, H12).

^{13}C NMR spectroscopy (126 MHz, $CDCl_3$, 298 K): 135.7 (d, *J* = 6 Hz, C24), 129.3 (C19), 128.4 (C21, C22), 127.8 (C23), 125.7 (C20), 83.2 (d, *J* = 19 Hz, C5), 54.7 (d, *J* = 9 Hz, C1/C3), 54.5 (d, *J* = 9 Hz, C1/C3), 50.8 (d, *J* = 3 Hz, C15), 50.0 (d, *J* = 5 Hz, C10), 47.9 (C11), 44.8 (C7), 38.3 (d, *J* = 3 Hz, C6), 31.5 (t, *J* = 5 Hz, C2/C4), 31.3 (t, *J* = 5 Hz, C2/C4), 30.7 (d, *J* = 3 Hz, C16), 28.2 (C8), 26.4 (C9), 19.9 (C18), 19.9 (C13), 19.3 (d, *J* = 89 Hz, C25), 18.8 (C14), 18.7 (C17), 13.7 (C12).

^{31}P{^{1}H} NMR spectroscopy (202 MHz, $CDCl_3$, 298 K): δ 123.5 (d, *J* = 7 Hz, P_O), 42.0 (d, *J* = 7 Hz, P_N).

^{31}P NMR spectroscopy (202 MHz, $CDCl_3$, 298 K): δ 123.5 (1P, t, *J* = 8 Hz, P_O), 42.0 (1P, m, P_N).

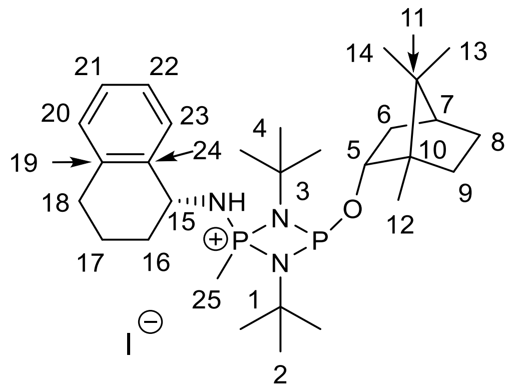

Synthesis and characterisation of [{(*S*)-$C_{10}H_{11}$NH}(Me)P(μ-N^{t}Bu)$_2$POBorn]I 11-*S*

Previously prepared **1** (0.28 g, 0.7 mmol) was dissolved in THF (4 ml). The solution was cooled to −78 °C and triethylamine (0.4 ml, excess) was added, followed by freshly distilled (*S*)-1,2,3,4-tetrahydro-1-naphthylamine (0.10 ml, *ca* 103 mg, 0.7 mmol). The reaction mixture was allowed to warm to room temperature and stirred overnight. The solvents were removed *in vacuo*, the residue was triturated with Et_2O (5 ml), extracted into toluene (10 ml) and the mixture was filtered. The toluene was removed *in vacuo* and the solid was dissolved in THF (8 ml). MeI (0.4 ml, excess) was added at room temperature and the mixture was stirred overnight, during which time the colourless solution turned pale yellow. Removal of the volatiles gave a yellow powder. The solid was purified *via* digestion in hot toluene (ca. 4 ml) and precipitation upon the dropwise addition of pentane to yield a pale yellow foam (390 mg, 86%). M.P. 114–116 °C. Elemental analysis $C_{29}H_{50}IN_3OP_2$ requires (%): C, 53.95; H, 7.81; N, 6.51. Found (%): C, 53.35; H, 7.60; N, 6.26.

^{1}H NMR spectroscopy (500 MHz, $CDCl_3$, 298 K): 7.53 (1H, dd, *J* = 21, 10 Hz, N–H), 7.29–7.11 (4H, m, H20, H21, H22, H23), 4.80 (1H, m, H15), 4.46 (1H, tq, *J* = 10, 2 Hz, H5), 3.03–2.70 (2H, m, H18$_{ax}$, H18$_{eq}$), 2.66 (3H, d, *J* = 15 Hz, H25), 2.36 (2H, m, H16$_{ax}$, H6$_{exo}$), 2.24 (2H, m, H16$_{eq}$, H17$_{ax}$), 1.93–1.78 (3H, m, H17$_{eq}$, H9$_{endo}$, H8$_{exo}$), 1.76 (1H, t, *J* = 4 Hz, H7), 1.50 (9H, s, H2/H4), 1.39 (9H, s, H2/H4), 1.41–1.19 (3H, m, H6$_{endo}$, H9$_{exo}$, H8$_{endo}$), 0.92 (6H, br, H13/H14), 0.91 (3H, s, H12).

^{13}C NMR spectroscopy (126 MHz, $CDCl_3$, 298 K): 135.7 (d, *J* = 6 Hz, C24), 129.3 (C19), 128.4 (C21, C22), 127.8 (C23), 125.7 (C20), 83.2 (d, *J* = 19 Hz, C5), 54.7 (d, *J* = 9 Hz, C1/C3), 54.5 (d, *J* = 9 Hz, C1/C3), 50.8 (d, *J* = 3 Hz, C15), 50.0 (d, *J* = 5 Hz, C10), 47.8 (C11), 44.8 (C7), 38.2 (d, *J* = 3 Hz, C6), 31.7 (t, *J* = 5 Hz, C2/C4), 31.2 (t, *J* = 5 Hz, C2/C4), 30.7 (d, *J* = 3 Hz, C16), 28.2 (C8), 26.5 (C9), 20.0 (C18), 19.9 (C13), 19.4 (d, *J* = 89 Hz, C25), 18.8 (C14), 18.6 (C17), 13.7 (C12).

^{31}P{^{1}H} NMR spectroscopy (202 MHz, $CDCl_3$, 298 K): δ 123.7 (d, *J* = 7 Hz, P_O), 41.9 (d, *J* = 7 Hz, P_N).

^{31}P NMR spectroscopy (202 MHz, $CDCl_3$, 298 K): δ 123.7 (1P, t, *J* = 8 Hz, P_O), 41.9 (1P, m, P_N).

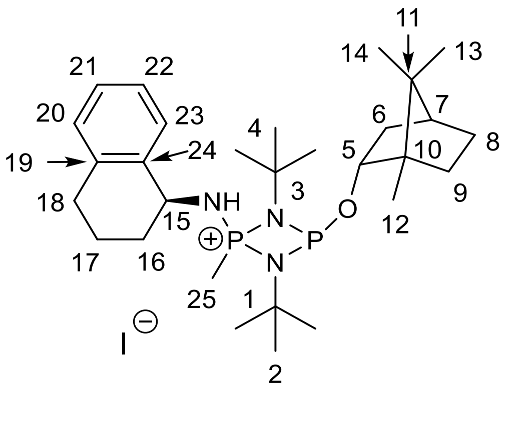

Synthesis and characterisation of [{(*R*)-$C_2H_5CH(CH_3)NH$}(Me)P(μ-N^{t}Bu)$_2$POBorn]I 12-*R*

Previously prepared **1** (0.39 g, 1 mmol) was dissolved in THF (4 ml). The solution was cooled to −78 °C and triethylamine (0.5 ml, excess) was added, followed by a solution of (*R*)-*sec*-butylamine (73 mg, ca. 0.10 ml, 1 mmol) in THF (3 ml). The reaction mixture was allowed to warm to room temperature and stirred overnight. The solvents were removed *in vacuo*, the residue was triturated with Et_2O (6 ml), extracted into toluene (12 ml) and the mixture was filtered. The toluene was removed *in vacuo* and the solid was dissolved in THF (12 ml). MeI (0.5 ml, excess) was added at room temperature and the mixture was stirred overnight, during which time the colourless solution became pale yellow. Removal of the volatiles gave a white powder. The solid was recrystallised from hot THF (ca. 6 ml) to give large colourless needle-like crystals (130 mg, 23%). A second crop of crystal was obtained from the supernatant (180 mg, 31%). Combined yield of 310 mg, 54%. M.P. decomp. ca. 210 °C. Elemental analysis $C_{23}H_{48}IN_3OP_2$ requires (%): C, 48.34; H, 8.47; N, 7.35. Found (%):C, 48.15; H, 8.53; N, 7.27.

^{1}H NMR spectroscopy (500 MHz, $CDCl_3$, 298 K): 7.59 (1H, dd, *J* = 21, 9 Hz, N–H), 4.38 (1H, tt, *J* = 10, 3 Hz, H5), 3.69–3.58 (1H, m, H15), 2.49 (3H, d, *J* = 15 Hz, H19), 2.40–2.33 (1H, m, H6$_{exo}$), 1.92–1.76 (4H, m, H17, H9$_{endo}$, H8$_{exo}$), 1.74 (1H, t, *J* = 4 Hz, H7), 1.45 (9H, s, H2/H4), 1.44 (3H, br, H16), 1.43 (9H, s, H2/H4), 1.35–1.27 (1H, m, H6$_{endo}$), 1.20 (1H, m, H9$_{exo}$), 1.17 (1H, m, H8$_{endo}$), 0.97 (3H, t, *J* = 7 Hz, H18), 0.90 (6H, s, H13, H14), 0.88 (3H, s, H12).

^{13}C NMR spectroscopy (126 MHz, $CDCl_3$, 298 K): 82.6 (d, *J* = 17 Hz, C5), 54.3 (d, *J* = 9 Hz, C1/C3), 54.1 (d, *J* = 9 Hz, C1/C3), 49.9 (d, *J* = 5 Hz, C10), 49.7 (d, *J* = 3 Hz, C15), 47.7 (C11), 44.8 (C7), 38.2 (d, *J* = 3 Hz, C6), 31.5 (t, *J* = 5 Hz, C2/C4), 31.2 (t, *J* = 5 Hz, C2/C4), 30.7 (C17), 28.1 (C8), 26.4 (C9), 20.4 (d, *J* = 5 Hz, C16), 19.8 (C13), 18.8 (C14), 18.2 (d, *J* = 89 Hz, C19), 13.6 (C12), 10.6 (C18).

^{31}P{^{1}H} NMR spectroscopy (202 MHz, $CDCl_3$, 298 K): *δ* 122.4 (d, *J* = 8 Hz, P$_O$), 41.2 (d, *J* = 8 Hz, P$_N$).

^{31}P NMR spectroscopy (202 MHz, $CDCl_3$, 298 K): *δ* 122.4 (1P, t, *J* = 9 Hz, P$_O$), 41.2 (1P, m, P$_N$).

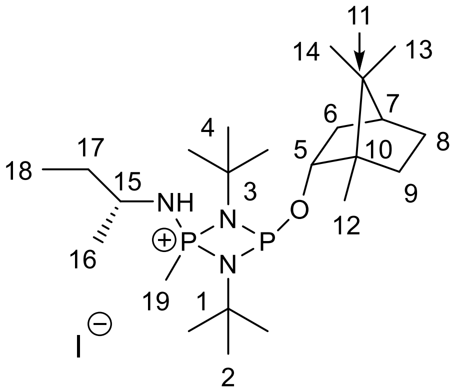

Synthesis and characterisation of [{(*S*)-$C_2H_5CH(CH_3)NH$}(Me)P(μ-N^tBu)$_2$POBorn]I 12-*S*

Previously prepared **1** (0.39 g, 1 mmol) was dissolved in THF (4 ml). The solution was cooled to −78 °C and triethylamine (0.5 ml, excess) was added, followed by a solution of (*S*)-*sec*-butylamine (73 mg, ca. 0.10 ml, 1 mmol) in THF (3 ml). The reaction mixture was allowed to warm to room temperature and stirred overnight. The solvents were removed *in vacuo*, the residue was triturated with Et_2O (6 ml), extracted into toluene (12 ml) and the mixture was filtered. The toluene was removed *in vacuo* and the solid was dissolved in THF (12 ml). MeI (0.5 ml, excess) was added at room temperature and the mixture was stirred overnight, during which time the colourless solution became pale yellow. Removal of the volatiles gave a white powder. The solid was recrystallised from hot THF (ca. 5 ml) to give large colourless needle-like crystals (120 mg, 21%). A second crop of crystal was obtained from the supernatant (200 mg, 35%). Combined yield of 320 mg, 56%. M.P. decomp. ca. 185 °C. Elemental analysis $C_{23}H_{48}IN_3OP_2$ requires (%): C, 48.34; H, 8.47; N, 7.35. Found (%):C, 48.07; H, 8.58; N, 7.22.

^{1}H NMR spectroscopy (500 MHz, $CDCl_3$, 298 K): 7.57 (1H, dd, *J* = 22, 10 Hz, N–H), 4.37 (1H, tt, *J* = 10, 3 Hz, H5), 3.69–3.58 (1H, m, H15), 2.49 (3H, d, *J* = 15 Hz, H19), 2.41–2.32 (1H, m, $H6_{exo}$), 1.91–1.76 (4H, m, H17, $H9_{endo}$, $H8_{exo}$), 1.74 (1H, t, *J* = 5 Hz, H7), 1.44 (9H, s, H2/H4), 1.43 (9H, s, H2/H4), 1.43 (3H, br, H16), 1.35–1.27 (1H, m, $H6_{endo}$), 1.28–1.15 (2H, m, $H8_{endo}$, $H9_{exo}$), 0.98 (3H, t, *J* = 7 Hz, H18), 0.90 (6H, s, H13, H14), 0.88 (3H, s, H12).

^{13}C NMR spectroscopy (126 MHz, $CDCl_3$, 298 K): 82.6 (d, *J* = 17 Hz, C5), 54.3 (d, *J* = 9 Hz, C1/C3), 54.1 (d, *J* = 9 Hz, C1/C3), 49.9 (d, *J* = 5 Hz, C10), 49.7 (d, *J* = 3 Hz, C15), 47.7 (C11), 44.8 (C7), 38.1 (d, *J* = 3 Hz, C6), 31.5 (t, *J* = 5 Hz, C2/C4), 31.3 (t, *J* = 5 Hz, C2/C4), 30.7 (C17), 28.0 (C8), 26.3 (C9), 20.4 (d, *J* = 5 Hz, C16), 19.8 (C13), 18.8 (C14), 18.2 (d, *J* = 89 Hz, C19), 13.6 (C12), 10.6 (C18).

^{31}P{^{1}H} NMR spectroscopy (202 MHz, $CDCl_3$, 298 K): δ 122.4 (d, *J* = 8 Hz, P_O), 41.2 (d, *J* = 8 Hz, P_N).

^{31}P NMR spectroscopy (202 MHz, $CDCl_3$, 298 K): δ 122.4 (1P, t, *J* = 9 Hz, P_O), 41.2 (1P, m, P_N).

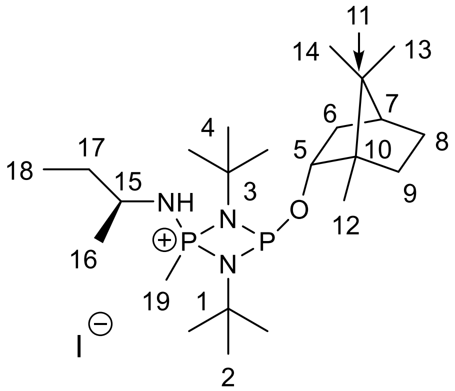

Synthesis and characterisation of [{(*R*)-$C_4H_9CH(CH_3)NH$}(Me)P(μ-N^tBu)$_2$POBorn]I 13-*R*

Previously prepared **1** (0.39 g, 1 mmol) was dissolved in THF (4 ml). The solution was cooled to −78 °C and triethylamine (0.5 ml, excess) was added, followed by a solution of (*R*)-2-aminohexane (101 mg, ca. 0.13 ml, 1 mmol) in THF (3 ml). The reaction mixture was allowed to warm to room temperature and stirred overnight. The solvents were removed *in vacuo*, the residue was triturated with Et_2O (6 ml), extracted into toluene (12 ml) and the mixture was filtered. The toluene was removed *in vacuo* and the solid was dissolved in THF (12 ml). MeI (0.5 ml, excess) was added at room temperature and the mixture was stirred overnight, during which time the colourless solution became pale yellow. All volatiles were removed to leave a white powder. The solid was recrystallised from hot toluene to give colourless star-like crystals (160 mg, 27%). M.P. 166–168 °C. Elemental analysis $C_{25}H_{52}IN_3OP_2$ requires (%): C, 50.08; H, 8.74; N, 7.01. Found (%):C, 49.87; H, 8.85; N, 6.86.

^{1}H NMR spectroscopy (500 MHz, $CDCl_3$, 298 K): 7.60 (1H, dd, *J* = 22, 10 Hz, N–H), 4.38 (1H, tq, *J* = 10, 2 Hz, H5), 3.72–3.62 (1H, m, H15), 2.48 (3H, d, *J* = 15 Hz, H21), 2.40–2.33 (1H, m, $H6_{exo}$), 1.92–1.76 (4H, m, H17, $H9_{endo}$, $H8_{exo}$), 1.74 (1H, t, *J* = 4 Hz, H7), 1.46–1.42 (21H, m, H2, H4, H16), 1.40–1.29 (5H, m, $H6_{endo}$, H18, H19), 1.19 (1H, m, $H9_{exo}$), 1.17 (1H, m, $H8_{endo}$), 0.92–0.85 (12H, m, H12, H13, H14, H20).

^{13}C NMR spectroscopy (126 MHz, $CDCl_3$, 298 K): 82.6 (d, *J* = 18 Hz, C5), 54.3 (d, *J* = 9 Hz, C1/C3), 54.1 (d, *J* = 9 Hz, C1/C3), 49.9 (d, *J* = 5 Hz, C10), 48.3 (d, *J* = 3 Hz, C15), 47.7 (C11), 44.7 (C7), 38.1 (d, *J* = 3 Hz, C6), 37.6 (d, *J* = 5 Hz, C17), 31.4 (t, *J* = 5 Hz, C2/C4), 31.3 (t, *J* = 5 Hz, C2/C4), 28.5 (C18), 28.0 (C8), 26.3 (C9), 22.4 (C19), 21.0 (d, *J* = 5 Hz, C16), 19.8 (C13), 18.7 (C14), 18.1 (d, *J* = 90 Hz, C21), 14.0 (C20), 13.5 (C12).

^{31}P{^{1}H} NMR spectroscopy (202 MHz, $CDCl_3$, 298 K): *δ* 122.2 (d, *J* = 8 Hz, P_O), 40.7 (d, *J* = 8 Hz, P_N).

^{31}P NMR spectroscopy (202 MHz, $CDCl_3$, 298 K): *δ* 122.2 (1P, t, *J* = 9 Hz, P_O), 40.7 (1P, m, P_N).

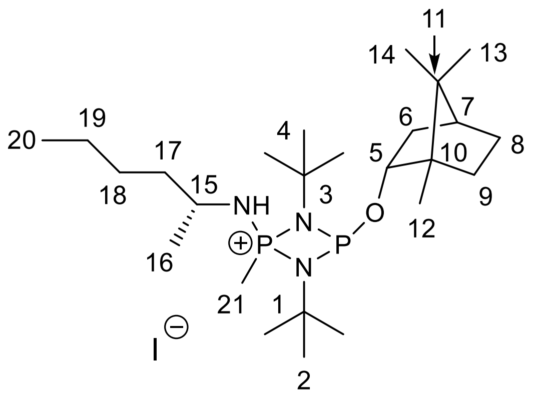

Synthesis and characterisation of [{(*S*)-$C_4H_9CH(CH_3)NH$}(Me)P(μ-N^tBu)$_2$POBorn]I 13-*S*

Previously prepared **1** (0.20 g, 0.5 mmol) was dissolved in THF (3 ml). The solution was cooled to −78 °C and triethylamine (0.25 ml, excess) was added, followed by a solution of (*S*)-2-aminohexane (50 mg, ca. 0.07 ml, 0.5 mmol) in THF (3 ml). The reaction mixture was allowed to warm to room temperature and stirred overnight. The solvents were removed *in vacuo*, the residue was triturated with Et_2O (6 ml), extracted into toluene (6 ml) and the mixture was filtered. The toluene was removed *in vacuo* and the solid was dissolved in THF (8 ml). MeI (0.25 ml, excess) was added at room temperature and the mixture was stirred overnight, during which time the colourless solution became pale yellow. All volatiles were removed to leave a white powder. The solid was recrystallised from hot toluene to give colourless prismatic crystals (150 mg, 50%). M.P. 174–176 °C. Elemental analysis $C_{25}H_{52}IN_3OP_2$ requires (%): C, 50.08; H, 8.74; N, 7.01. Found (%):C, 49.83; H, 8.84; N, 6.92.

^{1}H NMR spectroscopy (500 MHz, $CDCl_3$, 298 K): 7.64 (1H, dd, *J* = 22, 10 Hz, N–H), 4.38 (1H, tq, *J* = 10, 2 Hz, H5), 3.73–3.63 (1H, m, H15), 2.49 (3H, d, *J* = 15 Hz, H21), 2.41–2.33 (1H, m, $H6_{exo}$), 1.91–1.77 (4H, m, H17, $H9_{endo}$, $H8_{exo}$), 1.74 (1H, t, *J* = 4 Hz, H7), 1.46–1.43 (21H, m, H2, H4, H16), 1.41–1.30 (5H, m, $H6_{endo}$, H18, H19), 1.20 (1H, m, $H9_{exo}$), 1.17 (1H, m, $H8_{endo}$), 0.94–0.88 (12H, m, H12, H13, H14, H20).

^{13}C NMR spectroscopy (126 MHz, $CDCl_3$, 298 K): 82.6 (d, *J* = 18 Hz, C5), 54.3 (d, *J* = 9 Hz, C1/C3), 54.1 (d, *J* = 9 Hz, C1/C3), 49.9 (d, *J* = 5 Hz, C10), 48.4 (d, *J* = 3 Hz, C15), 47.7 (C11), 44.8 (C7), 38.1 (d, *J* = 3 Hz, C6), 37.7 (d, *J* = 5 Hz, C17), 31.5 (t, *J* = 5 Hz, C2/C4), 31.3 (t, *J* = 5 Hz, C2/C4), 28.5 (C18), 28.0 (C8), 26.3 (C9), 22.5 (C19), 21.0 (d, *J* = 5 Hz, C16), 19.8 (C13), 18.7 (C14), 18.1 (d, *J* = 89 Hz, C21), 14.0 (C20), 13.6 (C12).

^{31}P{^{1}H} NMR spectroscopy (202 MHz, $CDCl_3$, 298 K): δ 122.2 (d, *J* = 8 Hz, P_O), 40.7 (d, *J* = 8 Hz, P_N).

^{31}P NMR spectroscopy (202 MHz, $CDCl_3$, 298 K): δ 122.2 (1P, t, *J* = 9 Hz, P_O), 40.7 (1P, m, P_N).

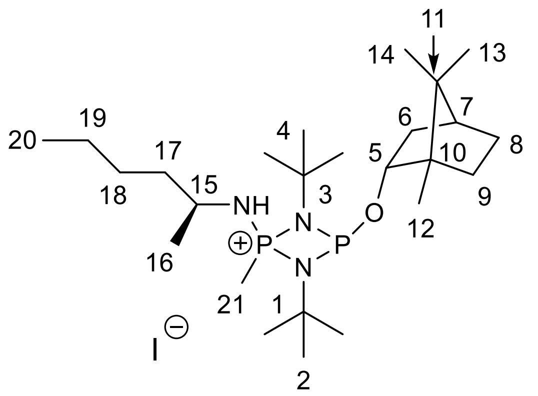

Synthesis and characterisation of [{(*R*)-$C_3H_5CH(CH_3)NH$}(Me)P(μ-N^{t}Bu)$_2$POBorn]I 14-*R*

Previously prepared **1** (0.20 g, 0.5 mmol) was dissolved in THF (3 ml). The solution was cooled to −78 °C and triethylamine (0.25 ml, excess) was added, followed by a solution of (*R*)-1-cyclopropylethylamine (43 mg, ca. 0.05 ml, 0.5 mmol) in THF (3 ml). The reaction mixture was allowed to warm to room temperature and stirred overnight. The solvents were removed *in vacuo*, the residue was triturated with Et_2O (6 ml), extracted into toluene (6 ml) and the mixture was filtered. The toluene was removed *in vacuo* and the solid was dissolved in THF (8 ml). MeI (0.25 ml, excess) was added at room temperature and the mixture was stirred overnight, during which time the colourless solution became pale yellow. All volatiles were removed to leave a white powder. The solid was recrystallised from hot toluene to give colourless prismatic crystals (40 mg, 14%). M.P. decomp. ca. 170 °C. Elemental analysis $C_{24}H_{48}IN_3OP_2$ requires (%): C, 49.40; H, 8.29; N, 7.20. Found (%):C, 48.93; H, 8.33; N, 7.02.

^{1}H NMR spectroscopy (500 MHz, $CDCl_3$, 298 K): *δ* 7.82 (1H, dd, *J* = 21, 10 Hz, N–H), 4.37 (1H, tq, *J* = 10, 2 Hz, H5), 3.02–2.92 (1H, m, H15), 2.49 (3H, d, *J* = 15 Hz, H20), 2.41–2.33 (1H, m, H6$_{exo}$), 1.85–1.75 (2H, m, H9$_{endo}$, H8$_{exo}$), 1.74 (1H, t, *J* = 4 Hz, H7), 1.53 (3H, d, *J* = 7 Hz, H16), 1.48 (9H, s, H2/H4), 1.43 (9H, s, H2/H4), 1.38–1.18 (4H, m, H6$_{endo}$, H8$_{endo}$, H9$_{exo}$, H17), 0.90 (6H, s, H13, H14), 0.89 (3H, s, H12), 0.62 (2H, m, H18$_{trans}$, H19$_{trans}$), 0.22 (1H, m, H18$_{cis}$/H19$_{cis}$), 0.10 (1H, m, H18$_{cis}$/H19$_{cis}$).

^{13}C NMR spectroscopy (126 MHz, $CDCl_3$, 298 K): 83.2 (d, *J* = 20 Hz, C5), 54.4 (d, *J* = 8 Hz, C1/C3), 54.2 (d, *J* = 8 Hz, C1/C3), 53.7 (d, *J* = 3 Hz, C15), 49.9 (d, *J* = 4 Hz, C10), 47.7 (C11), 44.6 (C7), 37.9 (d, *J* = 3 Hz, C6), 31.4 (t, *J* = 5 Hz, C2/C4), 31.1 (t, *J* = 5 Hz, C2/C4), 28.0 (C8), 26.3 (C9), 21.9 (d, *J* = 6 Hz, C16), 19.8 (C13), 18.6 (C14), 18.4 (d, *J* = 4 Hz, C17), 18.2 (d, *J* = 89 Hz, C20), 13.6 (C12), 6.7 (C18/19), 3.7 (C18/19).

^{31}P{^{1}H} NMR spectroscopy (202 MHz, $CDCl_3$, 298 K): *δ* 121.3 (d, *J* = 6 Hz, P$_O$), 39.2 (d, *J* = 6 Hz, P$_N$).

^{31}P NMR spectroscopy (202 MHz, $CDCl_3$, 298 K): *δ* 121.3 (1P, t, *J* = 7 Hz, P$_O$), 39.2 (1P, m, P$_N$).

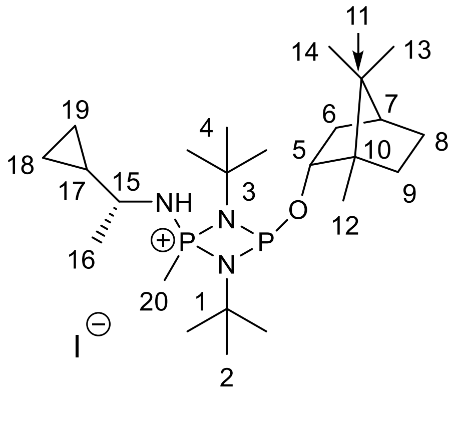

Synthesis and characterisation of [{(*S*)-$C_3H_5CH(CH_3)NH$}(Me)P(μ-N^tBu)$_2$POBorn]I 14-*S*

Previously prepared **1** (0.39 g, 1 mmol) was dissolved in THF (4 ml). The solution was cooled to −78 °C and triethylamine (0.5 ml, excess) was added, followed by a solution of (*S*)-1-cyclopropylethylamine (85 mg, ca. 0.11 ml, 1 mmol) in THF (3 ml). The reaction mixture was allowed to warm to room temperature and stirred overnight. The solvents were removed *in vacuo*, the residue was triturated with Et_2O (6 ml), extracted into toluene (12 ml) and the mixture was filtered. The toluene was removed *in vacuo* and the solid was dissolved in THF (12 ml). MeI (0.5 ml, excess) was added at room temperature and the mixture was stirred overnight, during which time the colourless solution became pale yellow. All volatiles were removed to leave a white powder. The solid was recrystallised from hot toluene to give colourless star-like crystals (180 mg, 31%). M.P. decomp. ca. 160 °C. Elemental analysis $C_{24}H_{48}IN_3OP_2$ requires (%): C, 49.40; H, 8.29; N, 7.20. Found (%):C, 49.21; H, 8.21; N, 7.08.

^{1}H NMR spectroscopy (500 MHz, $CDCl_3$, 298 K): *δ* 7.79 (1H, dd, *J* = 22, 9 Hz, N–H), 4.41 (1H, tq, *J* = 10, 2 Hz, H5), 3.01–2.91 (1H, m, H15), 2.49 (3H, d, *J* = 15 Hz, H20), 2.42–2.30 (1H, m, $H6_{exo}$), 1.83–1.76 (2H, m, $H9_{endo}$, $H8_{exo}$), 1.74 (1H, t, *J* = 4 Hz, H7), 1.54 (3H, d, *J* = 7 Hz, H16), 1.47 (9H, s, H2/H4), 1.43 (9H, s, H2/H4), 1.36–1.17 (4H, m, $H6_{endo}$, $H8_{endo}$, $H9_{exo}$, H17), 0.90 (6H, s, H13, H14), 0.88 (3H, s, H12), 0.62 (2H, m, $H18_{trans}$, $H19_{trans}$), 0.22 (1H, m, $H18_{cis}$/$H19_{cis}$), 0.10 (1H, m, $H18_{cis}$/$H19_{cis}$).

^{13}C NMR spectroscopy (126 MHz, $CDCl_3$, 298 K): 83.2 (d, *J* = 19 Hz, C5), 54.4 (d, *J* = 8 Hz, C1/C3), 54.2 (d, *J* = 8 Hz, C1/C3), 53.7 (d, *J* = 4 Hz, C15), 49.9 (d, *J* = 4 Hz, C10), 47.7 (C11), 44.6 (C7), 37.9 (d, *J* = 3 Hz, C6), 31.4 (t, *J* = 5 Hz, C2/C4), 31.1 (t, *J* = 5 Hz, C2/C4), 28.0 (C8), 26.3 (C9), 21.9 (d, *J* = 7 Hz, C16), 19.8 (C13), 18.6 (C14), 18.4 (d, *J* = 5 Hz, C17), 18.2 (d, *J* = 90 Hz, C20), 13.6 (C12), 6.7 (C18/19), 3.7 (C18/19).

^{31}P{^{1}H} NMR spectroscopy (202 MHz, $CDCl_3$, 298 K): *δ* 121.8 (d, *J* = 7 Hz, P_O), 39.2 (d, *J* = 7 Hz, P_N).

^{31}P NMR spectroscopy (202 MHz, $CDCl_3$, 298 K): *δ* 121.8 (1P, t, *J* = 8 Hz, P_O), 39.2 (1P, m, P_N).

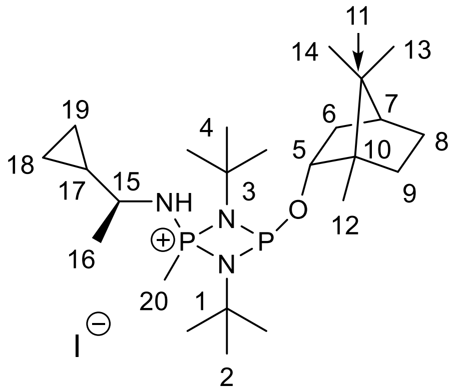

Synthesis and characterisation of [{(*R*)-$CH_3CH(COOCH_3)NH$}(Me)P(μ-N^tBu)$_2$POBorn]I 15-*R*

A suspension of D-alanine methyl ester hydrochloride (70 mg, 0.5 mmol) in THF (3 ml) and triethylamine (0.2 ml, excess) was stirred for 4 hours at room temperature and was then added dropwise to a solution of **1** (0.20 g, 0.5 mmol) in THF (3 ml) and triethylamine (0.2 ml, excess) at −78 °C. The reaction mixture was stirred for 2 hours at −78 °C, then allowed to warm to room temperature and stirred overnight. The solvents were removed *in vacuo*, the residue was triturated with Et_2O (6 ml), extracted into toluene (6 ml) and the mixture was filtered. The toluene was removed *in vacuo* and the solid was dissolved in THF (12 ml). MeI (0.25 ml, excess) was added at room temperature and the mixture was stirred overnight, during which time the colourless solution became pale yellow. Removal of the volatiles gave a white powder. The solid was recrystallised from hot toluene to give colourless block crystals (110 mg, 37%). M.P 140–144 °C. Elemental analysis $C_{23}H_{46}IN_3O_3P_2$ requires (%): C, 45.93; H, 7.71; N, 6.99. Found (%):C, 43.78; H, 7.84; N, 6.71.

^{1}H NMR spectroscopy (500 MHz, $CDCl_3$, 298 K): *δ* 8.02 (1H, dd, *J* = 19, 10 Hz, N–H), 4.38 (1H, tt, *J* = 10, 2 Hz, H5), 4.31 (1H, m, H15), 3.76 (3H, s, H18), 2.61 (3H, d, *J* = 15 Hz, H19), 2.41–2.35 (1H, m, $H6_{exo}$), 1.89–1.77 (2H, m, $H9_{endo}$, $H8_{exo}$), 1.76 (1H, br, H7), 1.74 (1H, br, $H8_{endo}$), 1.50 (3H, s, H16), 1.46 (9H, s, H2/H4), 1.40 (1H, sh, $H6_{endo}$), 1.37 (9H, s, H2/H4), 1.25 (1H, m, $H9_{exo}$), 0.90 (6H, br, H14, H12), 0.88 (3H, s, H13).

^{13}C NMR spectroscopy (126 MHz, $CDCl_3$, 298 K): 171.9 (d, *J* = 5 Hz, C17), 83.1 (d, *J* = 17 Hz, C5), 54.6 (d, *J* = 8 Hz, C1/C3), 54.5 (d, *J* = 8 Hz, C1/C3), 52.5 (C18), 50.0 (d, *J* = 5 Hz, C10), 49.4 (C15), 47.8 (C11), 44.8 (C7), 38.3 (d, *J* = 3 Hz, C6), 31.4 (t, *J* = 5 Hz, C2/C4), 31.2 (t, *J* = 5 Hz, C2/C4), 28.1 (C8), 26.4 (C9), 19.9 (C13), 19.6 (d, *J* = 5 Hz, C16), 18.7 (C14), 18.0 (d, *J* = 89 Hz, C19), 13.5 (C12).

^{31}P{^{1}H} NMR spectroscopy (202 MHz, $CDCl_3$, 298 K): *δ* 123.7 (d, *J* = 7 Hz, P_O), 43.9 (d, *J* = 8 Hz, P_N).

^{31}P NMR spectroscopy (202 MHz, $CDCl_3$, 298 K): *δ* 123.7 (1P, t, *J* = 8 Hz, P_O), 43.9 (1P, m, P_N).

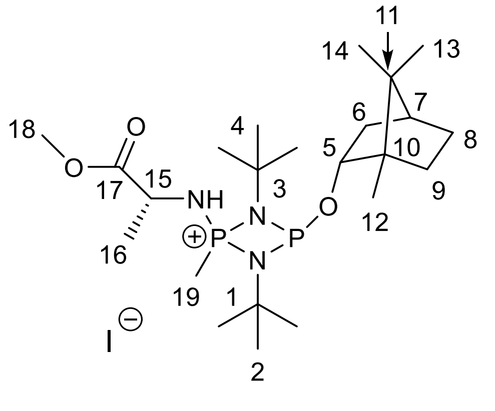

Synthesis and characterisation of [{(*S*)-$CH_3CH(COOCH_3)NH$}(Me)P(μ-N^tBu)$_2$POBorn]I 15-*S*

A suspension of L-alanine methyl ester hydrochloride (0.14 g, 0.5 mmol) in THF (3 ml) and triethylamine (0.5 ml, excess) was stirred for 4 hours at room temperature and was then added dropwise to a solution of **1** (0.39 g, 1.0 mmol) in THF (3 ml) and triethylamine (0.5 ml, excess) at −78 °C. The reaction mixture was stirred for 2 hours at −78 °C, then allowed to warm to room temperature and stirred overnight. The solvents were removed *in vacuo*, the residue was triturated with Et_2O (6 ml), extracted into toluene (12 ml) and the mixture was filtered. The toluene was removed *in vacuo* and the solid was dissolved in THF (12 ml). MeI (0.25 ml, excess) was added at room temperature and the mixture was stirred overnight, during which time the colourless solution became pale yellow. Removal of the volatiles gave a white powder. The solid was recrystallised from hot toluene to give colourless prismatic crystals (310 mg, 51%). M.P. 146–148 °C. Elemental analysis $C_{23}H_{46}IN_3O_3P_2$ requires (%): C, 45.93; H, 7.71; N, 6.99. Found (%):C, 44.73; H, 7.90; N, 6.72.

^{1}H NMR spectroscopy (500 MHz, $CDCl_3$, 298 K): *δ* 7.97 (1H, dd, *J* = 22, 11 Hz, N–H), 4.38 (1H, t, *J* = 10 Hz, H5), 4.33 (1H, m, H15), 3.77 (3H, s, H18), 2.60 (3H, d, *J* = 15 Hz, H19), 2.37–2.29 (1H, m, $H6_{exo}$), 2.17 (3H, s, H16), 1.88–1.77 (2H, m, $H9_{endo}$, $H8_{exo}$), 1.75 (1H, t, *J* = 5 Hz, H7), 1.73 (1H, br, $H8_{endo}$), 1.46 (9H, s, H2/H4), 1.44 (1H, sh, $H6_{endo}$), 1.37 (9H, s, H2/H4), 1.28–1.21 (1H, m, $H9_{exo}$), 0.90 (3H, s, H14), 0.90 (3H, s, H12), 0.89 (3H, s, H13).

^{13}C NMR spectroscopy (126 MHz, $CDCl_3$, 298 K): 171.9 (d, *J* = 5 Hz, C17), 83.0 (d, *J* = 17 Hz, C5), 54.6 (d, *J* = 8 Hz, C1/C3), 54.3 (t, *J* = 8 Hz, C1/C3), 52.4 (C18), 49.9 (d, *J* = 5 Hz, C10), 49.3 (C15), 47.7 (C11), 44.7 (C7), 37.9 (d, *J* = 3 Hz, C6), 31.5 (t, *J* = 5 Hz, C2/C4), 30.9 (t, *J* = 5 Hz, C2/C4), 27.9 (C8), 26.4 (C9), 19.8 (C13), 19.5 (d, *J* = 5 Hz, C16), 18.6 (C14), 17.8 (d, *J* = 89 Hz, C19), 13.6 (C12).

^{31}P{^{1}H} NMR spectroscopy (202 MHz, $CDCl_3$, 298 K): *δ* 124.1 (d, *J* = 7 Hz, P_O), 44.2 (d, *J* = 8 Hz, P_N).

^{31}P NMR spectroscopy (202 MHz, $CDCl_3$, 298 K): *δ* 124.1 (1P, t, *J* = 8 Hz, P_O), 44.2 (1P, m, P_N).

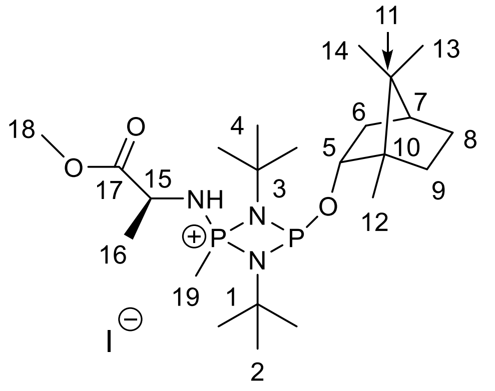

Synthesis and characterisation of [{(*R*)-$C_5H_{11}CH(COOC_2H_5)NH$}(Me)P(μ-N^{t}Bu)$_2$POBorn]I 16-*R*

A suspension of D-leucine ethyl ester hydrochloride (0.20 g, 1 mmol) in THF (3 ml) and triethylamine (0.5 ml, excess) was stirred overnight at room temperature and was then added dropwise to a solution of **1** (0.39 g, 1 mmol) in THF (3 ml) and triethylamine (0.5 ml, excess) at −78 °C. The reaction mixture was stirred for 3 hours at −78 °C, then allowed to warm to room temperature and stirred overnight. The solvents were removed *in vacuo*, the residue was triturated with Et_2O (6 ml), extracted into toluene (12 ml) and the mixture was filtered. The toluene was removed *in vacuo* and the solid was dissolved in THF (12 ml). MeI (0.5 ml, excess) was added at room temperature and the mixture was stirred overnight, during which time the colourless solution became pale yellow. Removal of the volatiles gave a white, hard foamy solid. The solid was recrystallised from hot THF to give colourless flake-like crystals (380 mg, 58%). M.P. 128–130 °C. Elemental analysis $C_{28}H_{56}IN_3O_3P_2$ requires (%): C, 50.07; H, 8.40; N, 6.26. Found (%):C, 49.06; H, 8.27; N, 6.25.

^{1}H NMR spectroscopy (500 MHz, $CDCl_3$, 298 K): *δ* 7.91 (1H, dd, *J* = 23, 11 Hz, N–H), 4.40 (1H, t, *J* = 10 Hz, H5), 4.30–4.17 (3H, m, H15, H21), 2.63 (3H, d, *J* = 15 Hz, H23), 2.41–2.28 (2H, m, H16), 1.95 (1H, m, H6$_{exo}$), 1.79 (1H, m, H9$_{endo}$), 1.74 (1H, t, *J* = 4 Hz, H7), 1.68 (1H, m, H8$_{exo}$), 1.53–1.48 (3H, m, H17, H18), 1.46 (9H, s, H2/H4), 1.36 (9H, s, H2/H4), 1.33 (3H, t, *J* = 7 Hz, H22), 1.29–1.19 (3H, m, H6$_{endo}$, H8$_{endo}$, H9$_{exo}$), 0.99 (3H, d, *J* = 6 Hz, H19$_a$), 0.97 (3H, d, *J* = 6 Hz, H19$_b$), 0.92 (3H, s, H12), 0.90 (6H, s, H13, H14).

^{13}C NMR spectroscopy (126 MHz, $CDCl_3$, 298 K): 170.7 (d, *J* = 4 Hz, C20), 83.2 (d, *J* = 19 Hz, C5), 61.4 (C21), 54.5 (t, *J* = 8 Hz, C1/C3), 54.2 (t, *J* = 8 Hz, C1/C3), 52.5 (C15), 49.9 (d, *J* = 5 Hz, C10), 47.8 (C11), 44.7 (C7), 42.7 (d, *J* = 5 Hz, C16), 38.0 (d, *J* = 3 Hz, C6), 31.4 (t, *J* = 5 Hz, C2/C4), 31.1 (t, *J* = 5 Hz, C2/C4), 28.0 (C8), 28.0 (C17), 26.2 (C9), 25.0 (C18), 23.4 (C19), 19.8 (C13), 18.6 (C14), 18.3 (d, *J* = 89 Hz, C23), 14.1 (C22), 13.4 (C12).

^{31}P{^{1}H} NMR spectroscopy (202 MHz, $CDCl_3$, 298 K): *δ* 123.1 (d, *J* = 7 Hz, P$_O$), 41.6 (d, *J* = 8 Hz, P$_N$).

^{31}P NMR spectroscopy (202 MHz, $CDCl_3$, 298 K): *δ* 123.1 (1P, t, *J* = 8 Hz, P$_O$), 41.6 (1P, m, P$_N$).

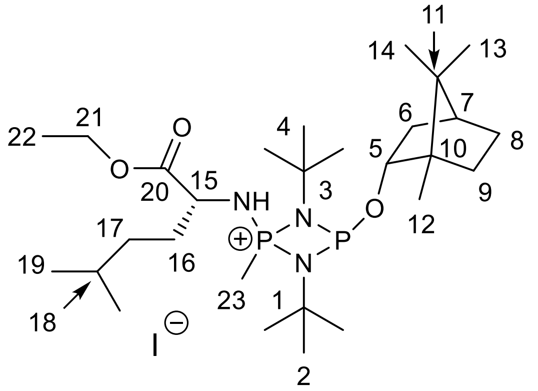

Synthesis and characterisation of [{(*S*)- $C_5H_{11}CH(COOC_2H_5)NH$}(Me)P(μ-N^tBu)$_2$POBorn]I 16-*S*

A suspension of L-leucine ethyl ester hydrochloride (0.20 g, 1 mmol) in THF (3 ml) and triethylamine (0.5 ml, excess) was stirred overnight at room temperature and was then added dropwise to a solution of **1** (0.39 g, 1 mmol) in THF (3 ml) and triethylamine (0.5 ml, excess) at −78 °C. The reaction mixture was stirred for 3 hours at −78 °C, then allowed to warm to room temperature and stirred overnight. The solvents were removed *in vacuo*, the residue was triturated with Et_2O (6 ml), extracted into toluene (12 ml) and the mixture was filtered. The toluene was removed *in vacuo* and the solid was dissolved in THF (12 ml). MeI (0.5 ml, excess) was added at room temperature and the mixture was stirred overnight, during which time the colourless solution became pale yellow. Removal of the volatiles gave a white, hard foamy solid. The solid was recrystallised from hot THF to give colourless flake-like crystals (390 mg, 59%). M.P. 122–124 °C. Elemental analysis $C_{28}H_{56}IN_3O_3P_2$ requires (%): C, 50.07; H, 8.40; N, 6.26. Found (%):C, 49.22; H, 8.17; N, 6.34.

^{1}H NMR spectroscopy (500 MHz, $CDCl_3$, 298 K): *δ* 7.90 (1H, dd, *J* = 22, 11 Hz, N–H), 4.41 (1H, tq, *J* = 11, 1 Hz, H5), 4.30–4.18 (1H, m, H15), 4.22 (2H, q, *J* = 7 Hz, H21), 2.63 (3H, d, *J* = 15 Hz, H23), 2.36–2.28 (2H, m, H16), 1.92 (1H, m, $H6_{exo}$), 1.77 (1H, m, $H9_{endo}$), 1.73 (1H, t, *J* = 4 Hz, H7), 1.66 (1H, m, $H8_{exo}$), 1.51–1.49 (3H, m, H17, H18), 1.47 (9H, s, H2/H4), 1.41–1.29 (3H, m, $H6_{endo}$, $H8_{endo}$, $H9_{exo}$), 1.35 (9H, s, H2/H4), 1.33 (3H, t, *J* = 7 Hz, H22), 0.99 (3H, d, *J* = 6 Hz, $H19_a$), 0.97 (3H, d, *J* = 6 Hz, $H19_b$), 0.90 (6H, s, H13, H14), 0.89 (3H, s, H12).

^{13}C NMR spectroscopy (126 MHz, $CDCl_3$, 298 K): 170.7 (d, *J* = 4 Hz, C20), 83.3 (d, *J* = 19 Hz, C5), 61.3 (C21), 54.4 (t, *J* = 9 Hz, C1, C3), 52.6 (C15), 49.8 (d, *J* = 5 Hz, C10), 47.9 (C11), 44.6 (C7), 42.6 (d, *J* = 5 Hz, C16), 37.6 (d, *J* = 2 Hz, C6), 31.5 (t, *J* = 5 Hz, C2/C4), 30.9 (t, *J* = 5 Hz, C2/C4), 28.1 (C8), 27.8 (C17), 26.2 (C9), 25.1 (C18), 23.4 (C19), 19.8 (C13), 18.6 (C14), 18.2 (d, *J* = 88 Hz, C23), 14.2 (C22), 13.6 (C12).

^{31}P{^{1}H} NMR spectroscopy (202 MHz, $CDCl_3$, 298 K): *δ* 123.4 (d, *J* = 7 Hz, P_O), 42.3 (d, *J* = 7 Hz, P_N).

^{31}P NMR spectroscopy (202 MHz, $CDCl_3$, 298 K): *δ* 123.4 (1P, t, *J* = 9 Hz, P_O), 42.3 (1P, m, P_N).

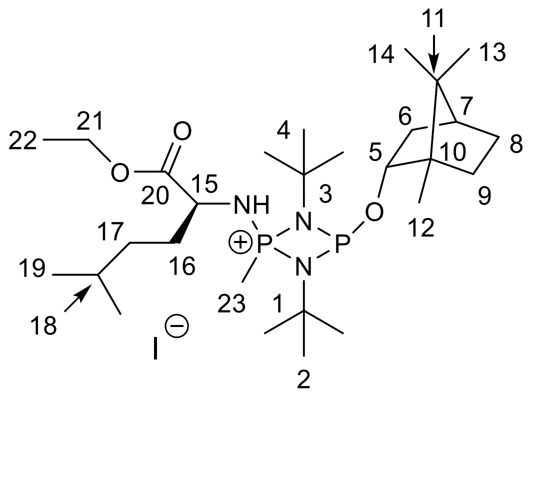

Synthesis and characterisation of [{(*R*)-$CH_3S(CH_2)_2CH(COOCH_3)NH$}(Me)P(μ-$N^tBu$)$_2$POBorn]I 17-*R*

A suspension of D-methionine methyl ester hydrochloride (0.20 g, 1 mmol) in THF (3 ml) and triethylamine (0.5 ml, excess) was stirred overnight at room temperature and was then added dropwise to a solution of **1** (0.39 g, 1 mmol) in THF (3 ml) and triethylamine (0.5 ml, excess) at −78 °C. The reaction mixture was stirred for 3 hours at −78 °C, then allowed to warm to room temperature and stirred overnight. The solvents were removed *in vacuo*, the residue was triturated with Et_2O (6 ml), extracted into toluene (12 ml) and the mixture was filtered. The toluene was removed *in vacuo* and the solid was dissolved in THF (12 ml). MeI (0.25 ml, excess) was added at room temperature and the mixture was stirred overnight. Removal of the volatiles gave a white, hard foamy solid. The solid was purified *via* digestion in hot toluene (ca. 4 ml) and precipitation upon the dropwise addition of pentane to yield a white powder (440 mg, 67%). M.P. 108–110 °C. Elemental analysis $C_{25}H_{50}IN_3O_3P_2S$ requires (%): C, 45.39; H, 7.62; N, 6.35. Found (%):C, 44.93; H, 7.81; N, 6.10.

^{1}H NMR spectroscopy (500 MHz, $CDCl_3$, 298 K): δ 8.00 (1H, dd, *J* = 22, 10 Hz, N–H), 4.39 (1H, t, *J* = 11 Hz, H5), 3.77 (3H, s, H20), 2.80 (1H, dt, *J* = 11, 5 Hz, H15), 2.63 (3H, d, *J* = 15 Hz, H21), 2.50 (2H, m, H17), 2.33 (2H, m, H16), 2.12 (3H, s, H18), 1.91 (1H, m, $H6_{exo}$), 1.79 (1H, m, $H9_{endo}$), 1.74 (1H, t, *J* = 4 Hz, H7), 1.55 (1H, m, $H8_{exo}$), 1.46 (9H, s, H2/H4), 1.37 (9H, s, H2/H4), 1.33–1.24 (3H, m, $H6_{endo}$, $H8_{endo}$, $H9_{exo}$), 0.90 (9H, br, H12, H13, H14).

^{13}C NMR spectroscopy (126 MHz, $CDCl_3$, 298 K): 170.8 (d, *J* = 4 Hz, C19), 83.4 (d, *J* = 19 Hz, C5), 54.7 (d, *J* = 8 Hz, C1/C3), 54.5 (d, *J* = 8 Hz, C1/C3), 52.5 (C15), 52.3 (C20), 50.0 (d, *J* = 5 Hz, C10), 47.9 (C11), 44.8 (C7), 38.1 (d, *J* = 3 Hz, C6), 33.1 (d, *J* = 6 Hz, C16), 31.4 (t, *J* = 5 Hz, C2/C4), 31.2 (t, *J* = 5 Hz, C2/C4), 30.2 (C17), 28.0 (C8), 26.3 (C9), 19.9 (C13), 18.7 (C14), 18.2 (d, *J* = 89 Hz, C21), 15.7 (C18), 13.5 (C12).

^{31}P{^{1}H} NMR spectroscopy (202 MHz, $CDCl_3$, 298 K): δ 123.9 (d, *J* = 7 Hz, P_O), 43.4 (d, *J* = 7 Hz, P_N).

^{31}P NMR spectroscopy (202 MHz, $CDCl_3$, 298 K): δ 123.9 (1P, t, *J* = 8 Hz, P_O), 43.4 (1P, m, P_N).

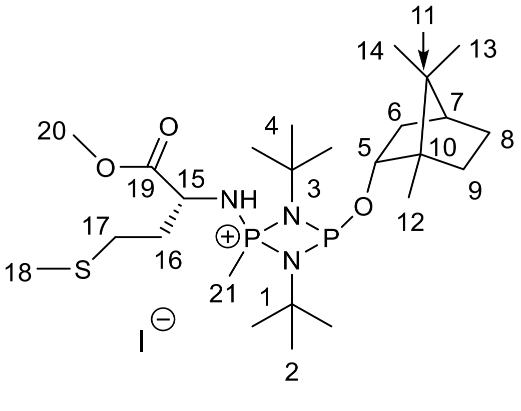

Synthesis and characterisation of [{(*S*)-$CH_3S(CH_2)_2CH(COOCH_3)NH$}(Me)P(μ-$N^tBu$)$_2$POBorn]I 17-*S*

A suspension of L-methionine methyl ester hydrochloride (0.20 g, 1 mmol) in THF (3 ml) and triethylamine (0.5 ml, excess) was stirred overnight at room temperature and was then added dropwise to a solution of **1** (0.39 g, 1 mmol) in THF (3 ml) and triethylamine (0.5 ml, excess) at −78 °C. The reaction mixture was stirred for 3 hours at −78 °C, then allowed to warm to room temperature and stirred overnight. The solvents were removed *in vacuo*, the residue was triturated with Et_2O (6 ml), extracted into toluene (12 ml) and the mixture was filtered. The toluene was removed *in vacuo* and the solid was dissolved in THF (12 ml). MeI (0.25 ml, excess) was added at room temperature and the mixture was stirred overnight, during which time the solution remained colourless. Removal of the volatiles gave a white, hard foamy solid. The solid was purified *via* digestion in hot toluene (ca. 4 ml) and precipitation upon the dropwise addition of pentane to yield a white powder (390 mg, 0.59 mmol, 59%). M.P. 80–82 °C. Elemental analysis $C_{25}H_{50}IN_3O_3P_2S$ requires (%): C, 45.39; H, 7.62; N, 6.35. Found (%):C, 45.15; H, 7.80; N, 6.15.

^{1}H NMR spectroscopy (500 MHz, $CDCl_3$, 298 K): δ 8.04 (1H, dd, *J* = 22, 10 Hz, N–H), 4.41 (1H, t, *J* = 11 Hz, H5), 3.79 (3H, s, H20), 2.80 (1H, dt, *J* = 11, 5 Hz, H15), 2.64 (3H, d, *J* = 15 Hz, H21), 2.51 (2H, m, H17), 2.32 (2H, m, H16), 2.11 (3H, s, H18), 1.91 (1H, m, $H6_{exo}$), 1.79 (1H, m, $H9_{endo}$), 1.74 (1H, t, *J* = 4 Hz, H7), 1.57 (1H, m, $H8_{exo}$), 1.48 (9H, s, H2/H4), 1.37 (9H, s, H2/H4), 1.32–1.27 (3H, m, $H6_{endo}$, $H8_{endo}$, $H9_{exo}$), 0.92 (3H, br, H12), 0.91 (6H, br, H13, H14).

^{13}C NMR spectroscopy (126 MHz, $CDCl_3$, 298 K): 170.8 (d, *J* = 4 Hz, C19), 83.4 (d, *J* = 19 Hz, C5), 54.6 (d, *J* = 8 Hz, C1/C3), 54.5 (d, *J* = 8 Hz, C1/C3), 52.7 (C15), 52.4 (C20), 50.0 (d, *J* = 5 Hz, C10), 47.9 (C11), 44.8 (C7), 37.8 (d, *J* = 3 Hz, C6), 33.0 (d, *J* = 6 Hz, C16), 31.5 (t, *J* = 5 Hz, C2/C4), 31.0 (t, *J* = 5 Hz, C2/C4), 30.2 (C17), 27.9 (C8), 26.4 (C9), 19.9 (C13), 18.7 (C14), 18.1 (d, *J* = 89 Hz, C21), 15.7 (C18), 13.7 (C12).

^{31}P{^{1}H} NMR spectroscopy (202 MHz, $CDCl_3$, 298 K): δ 123.9 (d, *J* = 7 Hz, P_O), 43.5 (d, *J* = 7 Hz, P_N).

^{31}P NMR spectroscopy (202 MHz, $CDCl_3$, 298 K): δ 123.9 (1P, t, *J* = 8 Hz, P_O), 43.5 (1P, m, P_N).

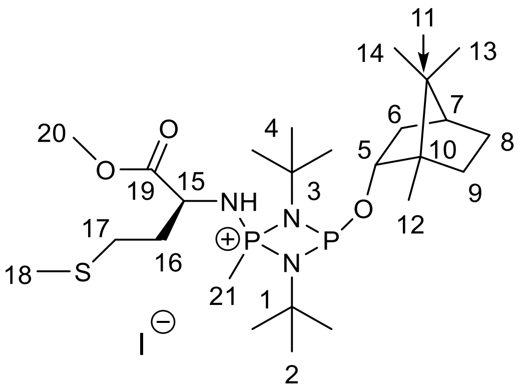

2. NMR Spectroscopy

NMR Characterisation of Compounds

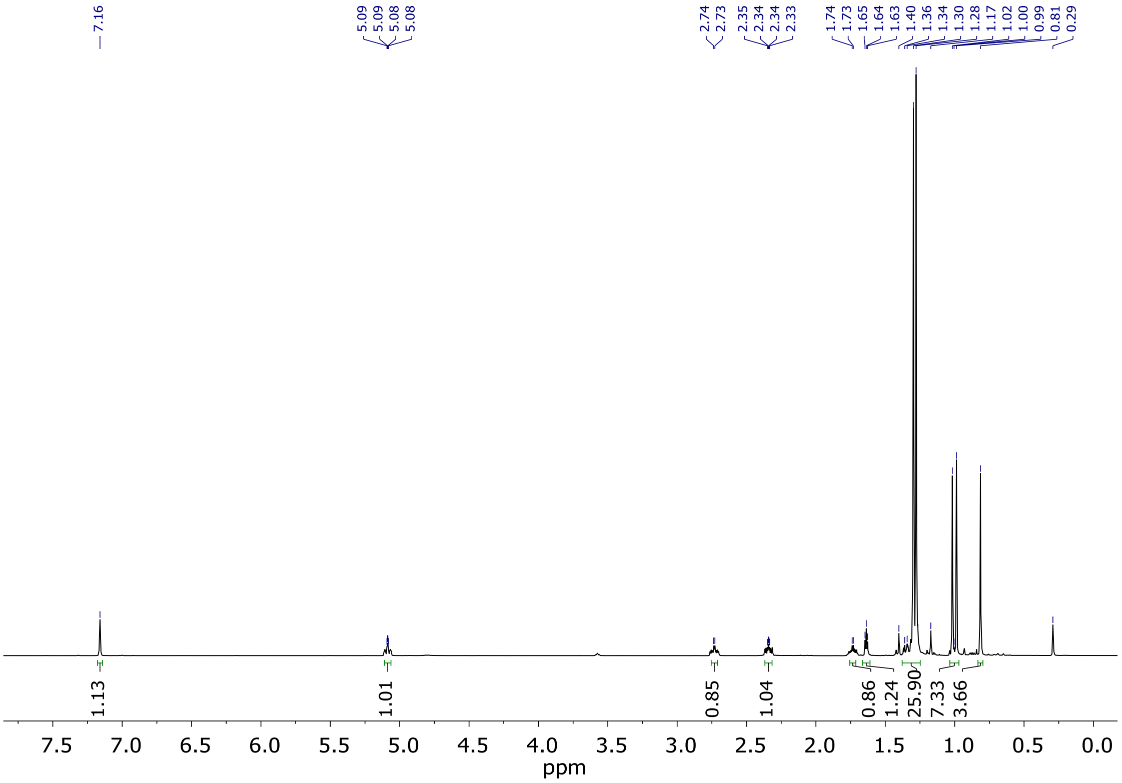

Figure S1a. ^{1}H NMR spectrum of **1** (500 MHz, 298 K, C_6D_6).

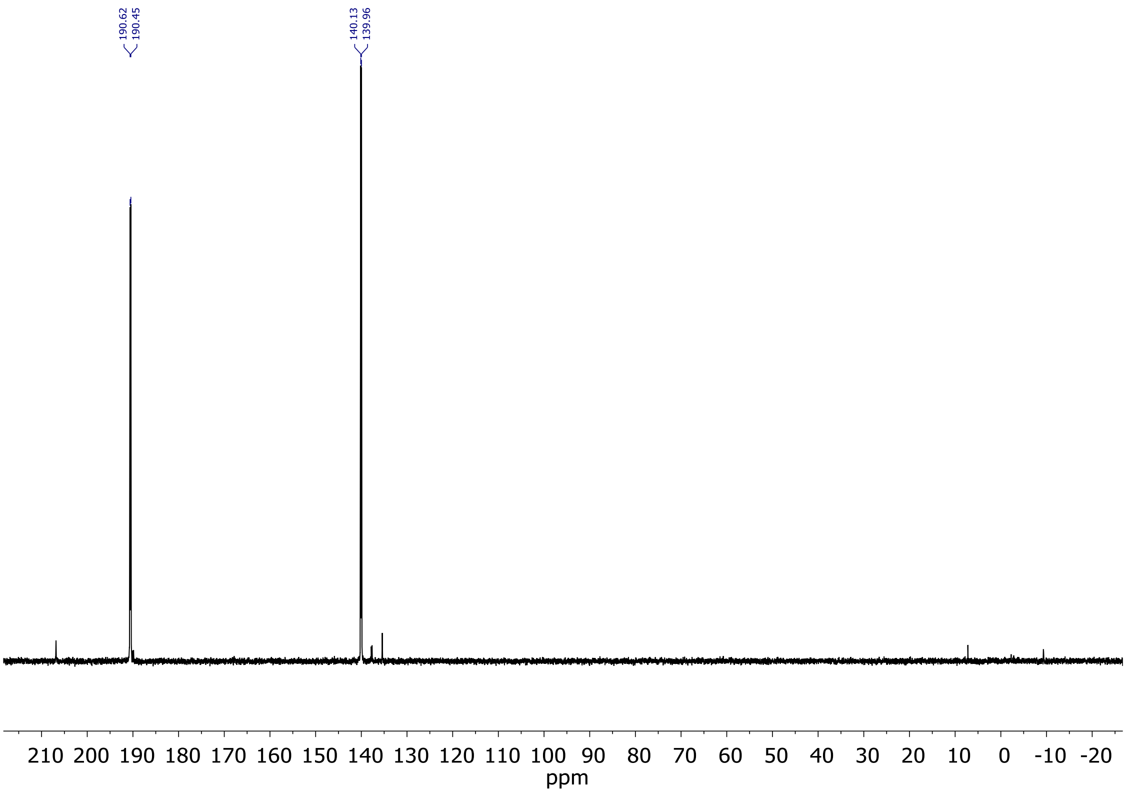

Figure S1b. $^{31}P\{^{1}H\}$ NMR spectrum of **1** (202 MHz, 298 K, C_6D_6).

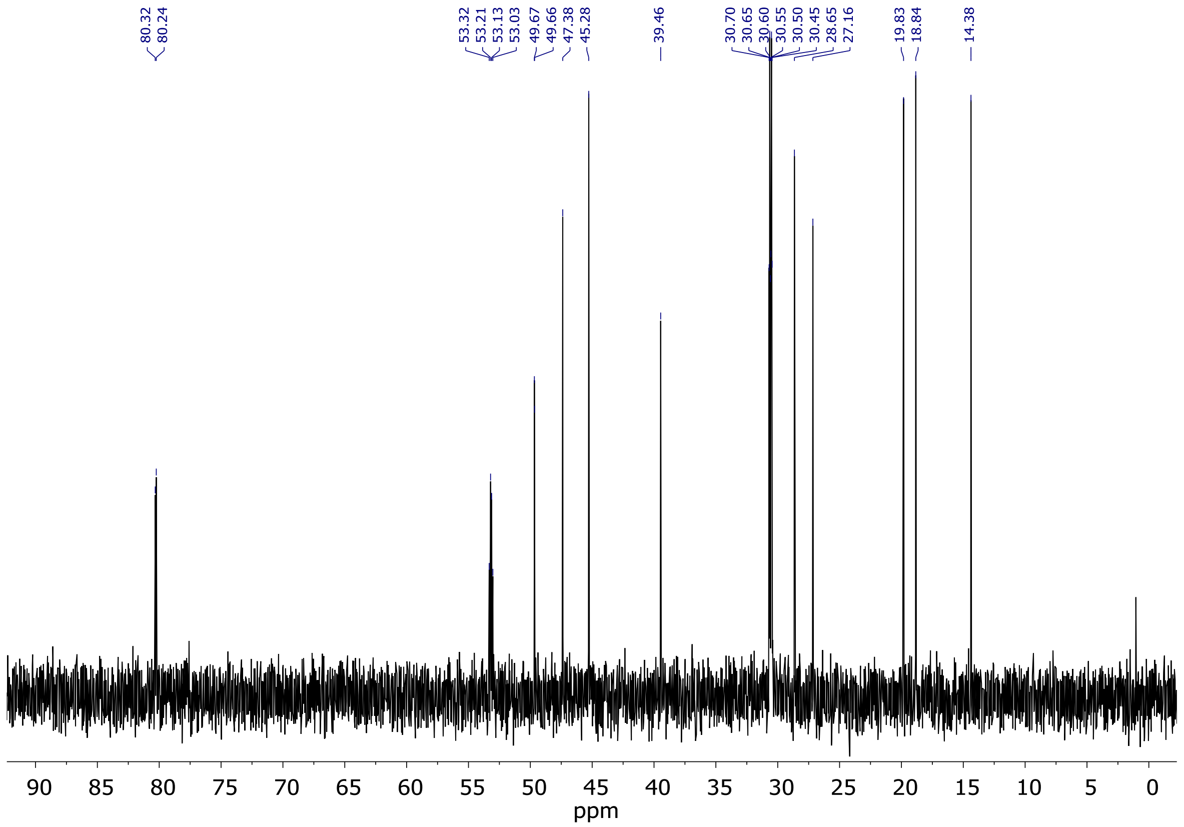

Figure S1c. $^{13}C\{^1H\}$ NMR spectrum of **1** (126 MHz, 298 K, C_6D_6).

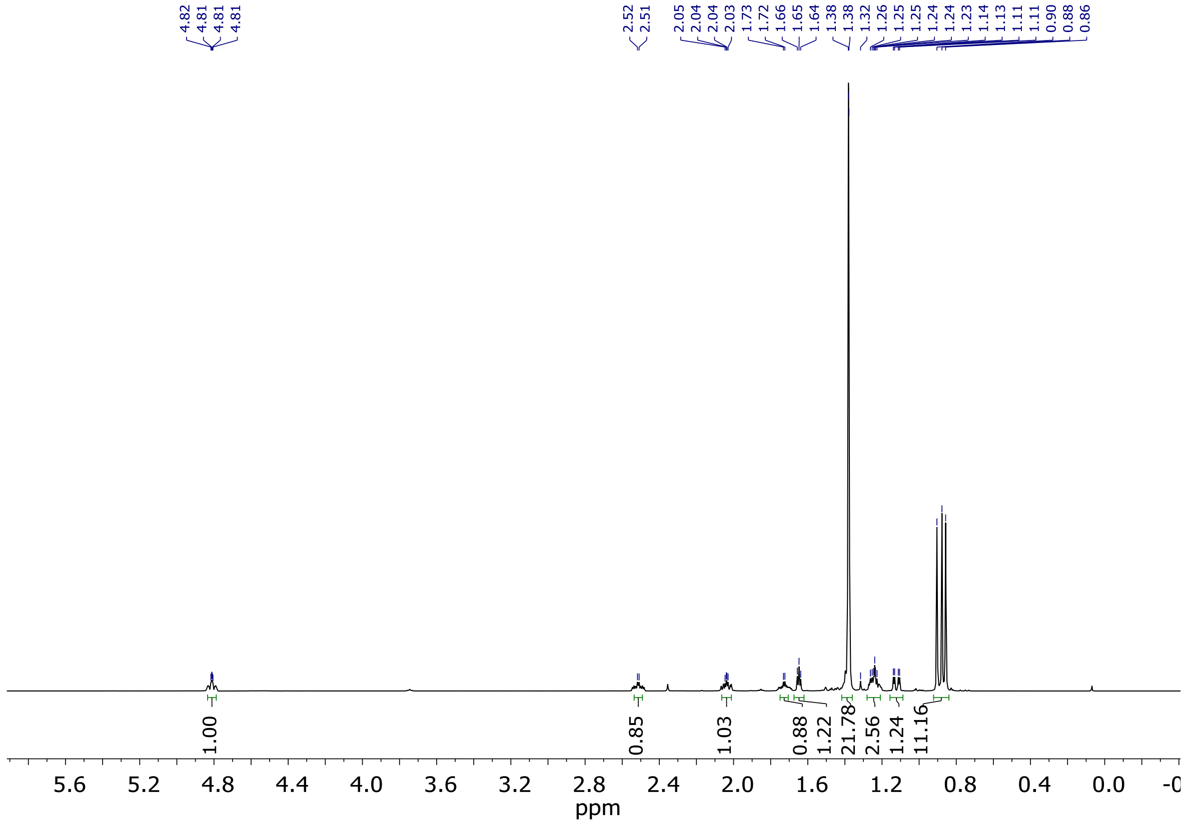

Figure S1d. ^1H NMR spectrum of **1** (500 MHz, 298 K, $CDCl_3$).

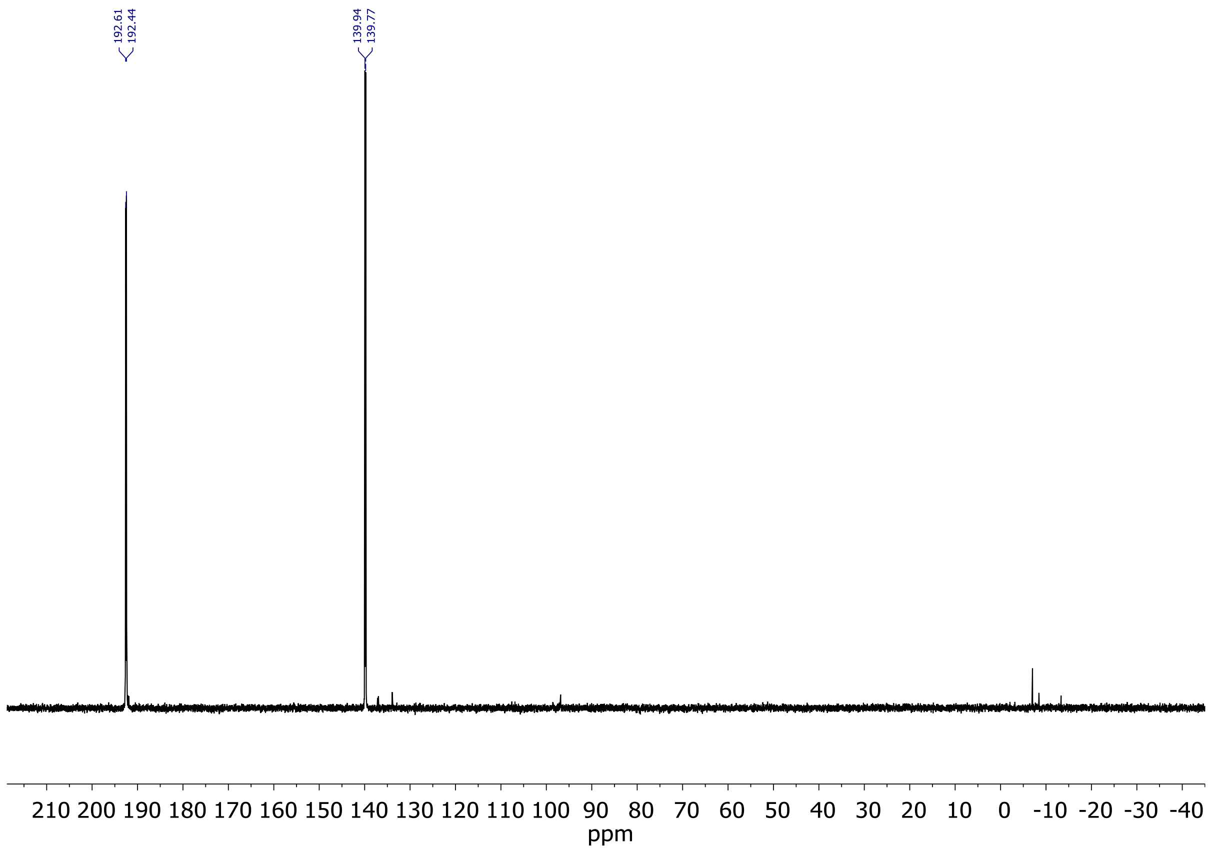

Figure S1e. $^{31}P\{^{1}H\}$ NMR spectrum of **1** (202 MHz, 298 K, $CDCl_3$).

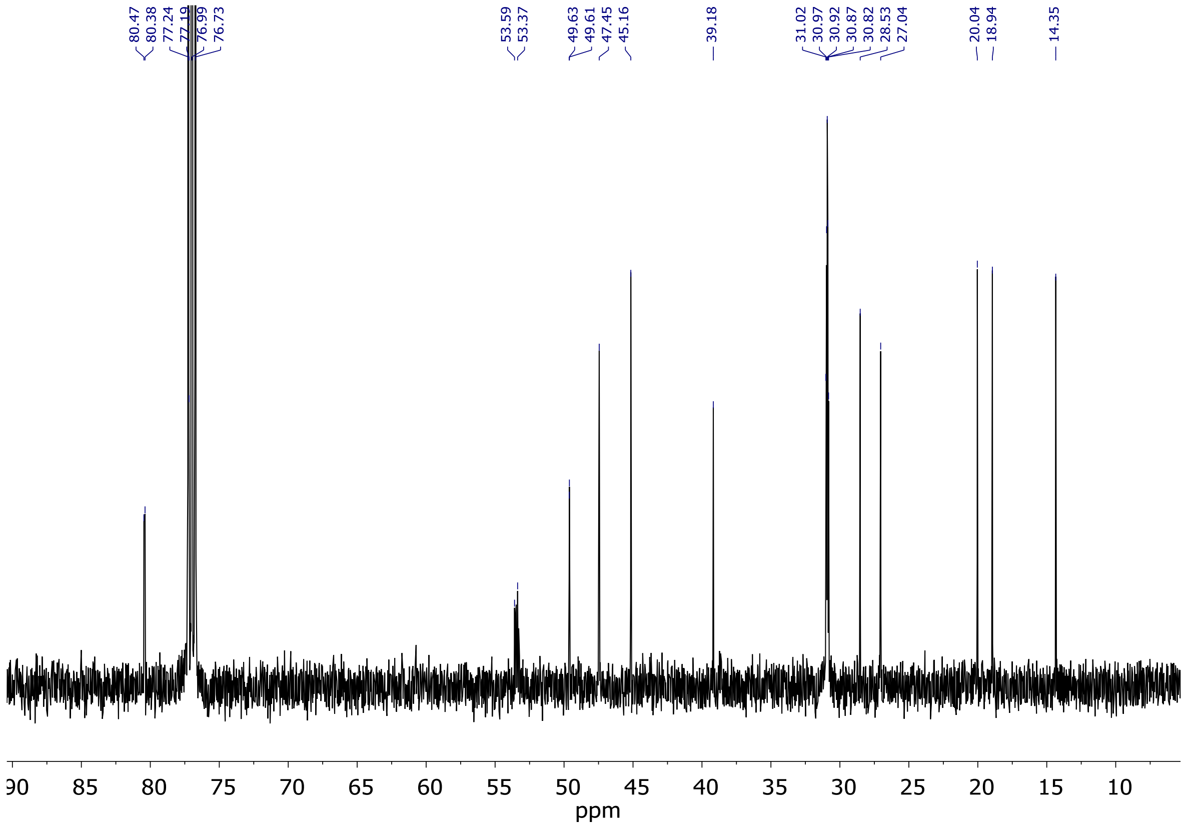

Figure S1f. $^{13}C\{^{1}H\}$ NMR spectrum of **1** (126 MHz, 298 K, $CDCl_3$).

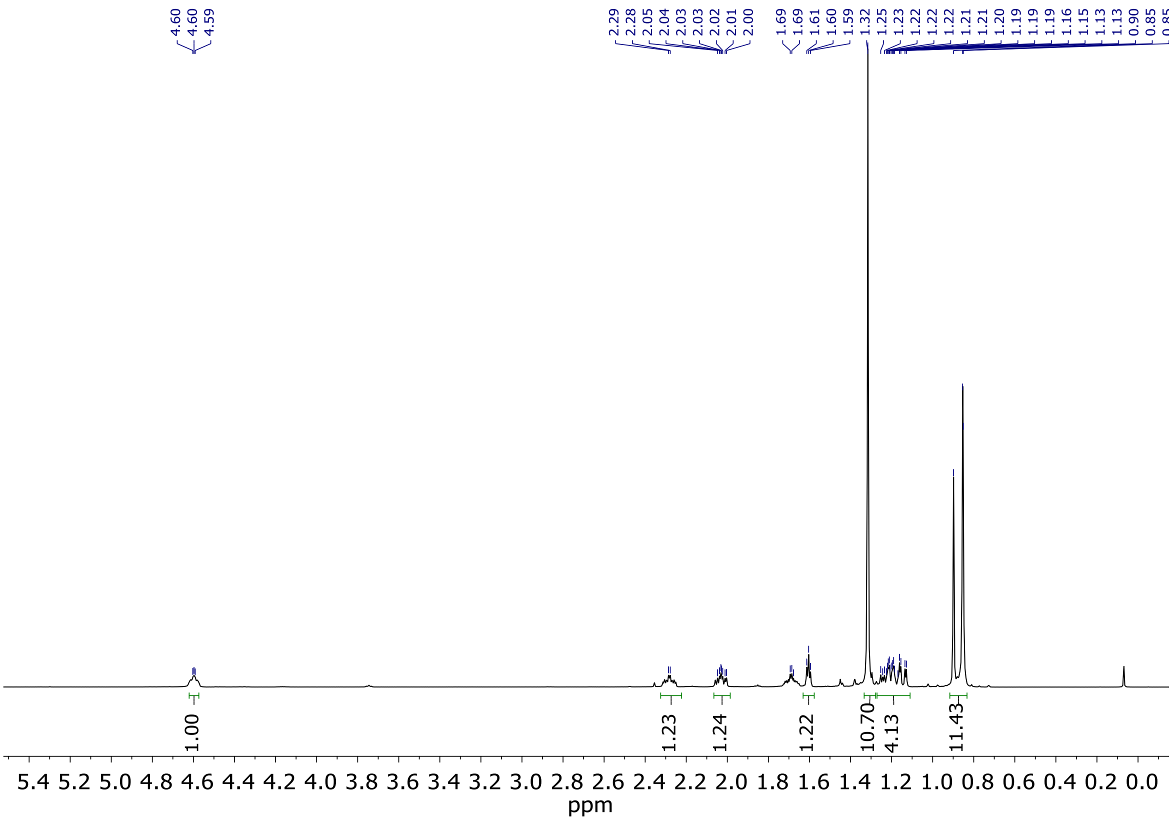

Figure S2a. ^{1}H NMR spectrum of **2** (500 MHz, 298 K, $CDCl_3$).

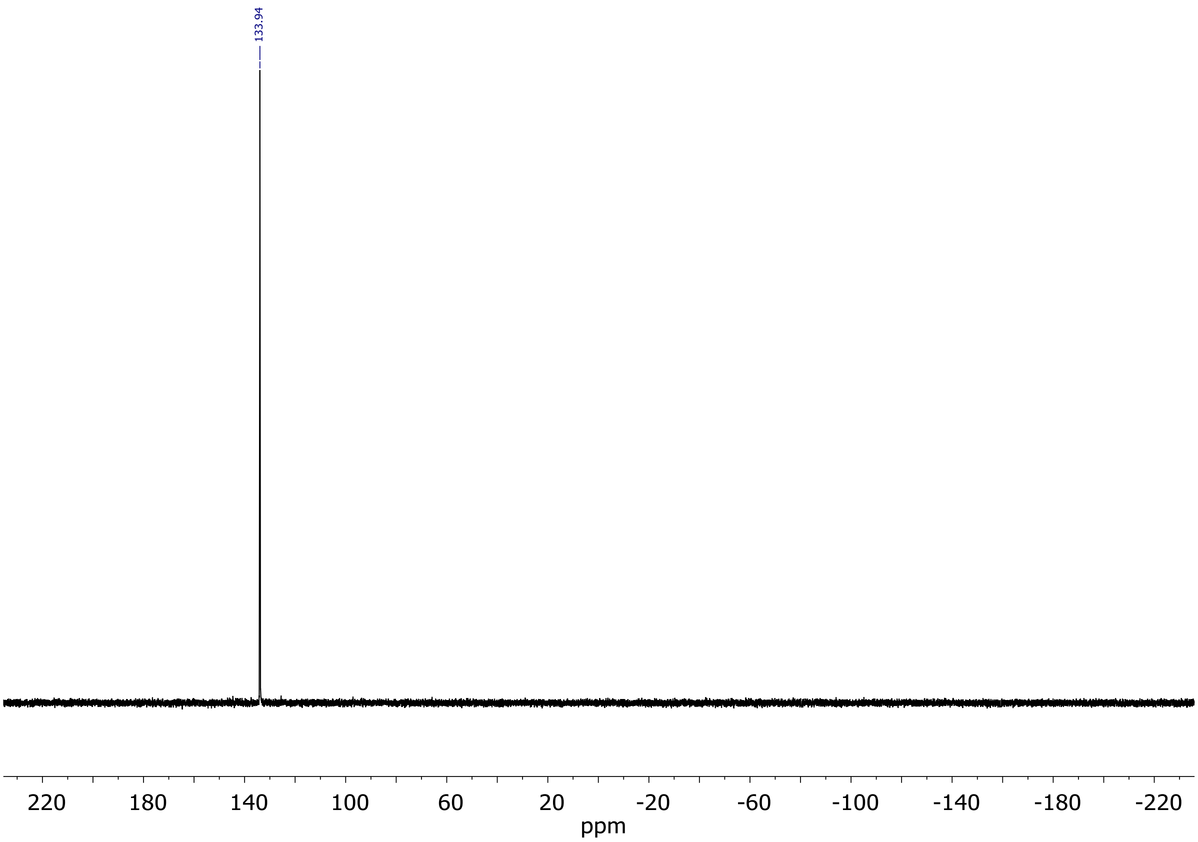

Figure S2b. ^{31}P NMR spectrum of **2** (202 MHz, 298 K, $CDCl_3$).

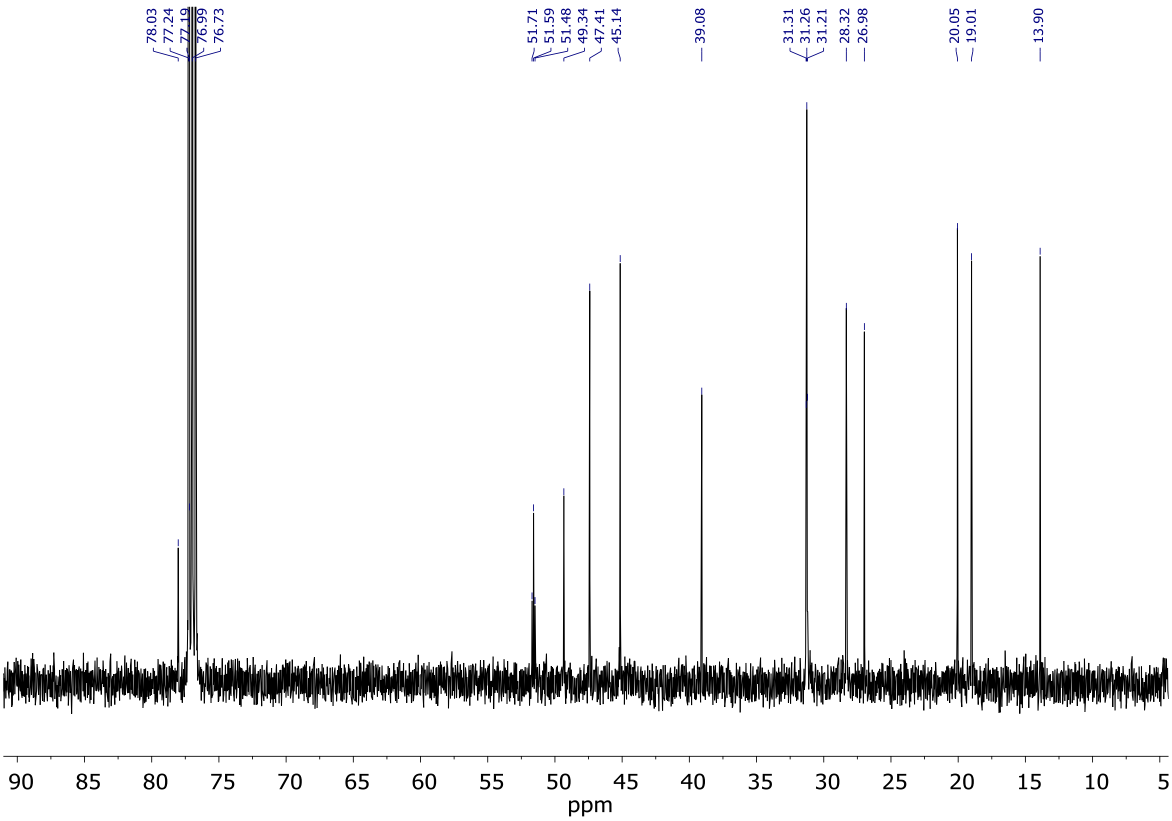

Figure S2c. $^{13}C\{^{1}H\}$ NMR spectrum of **2** (126 MHz, 298 K, $CDCl_3$).

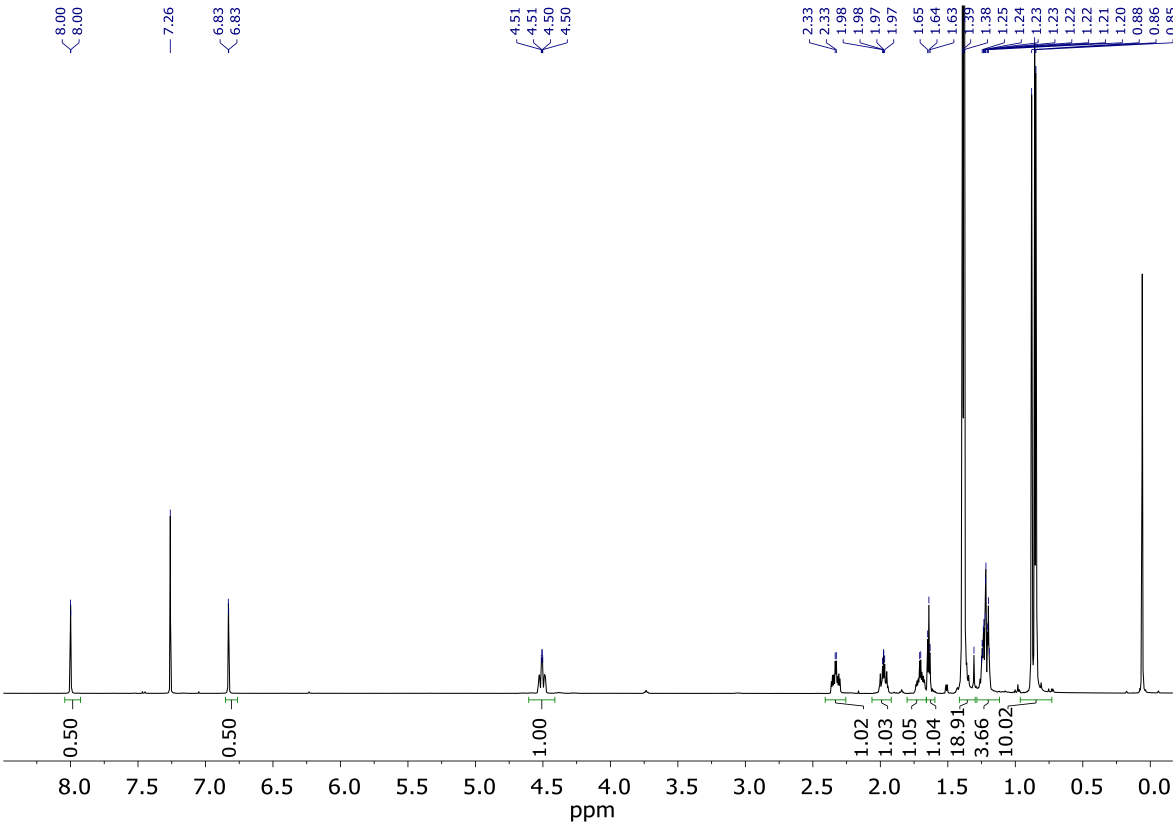

Figure S3a. ^{1}H NMR spectrum of **3** (500 MHz, 298 K, $CDCl_3$)

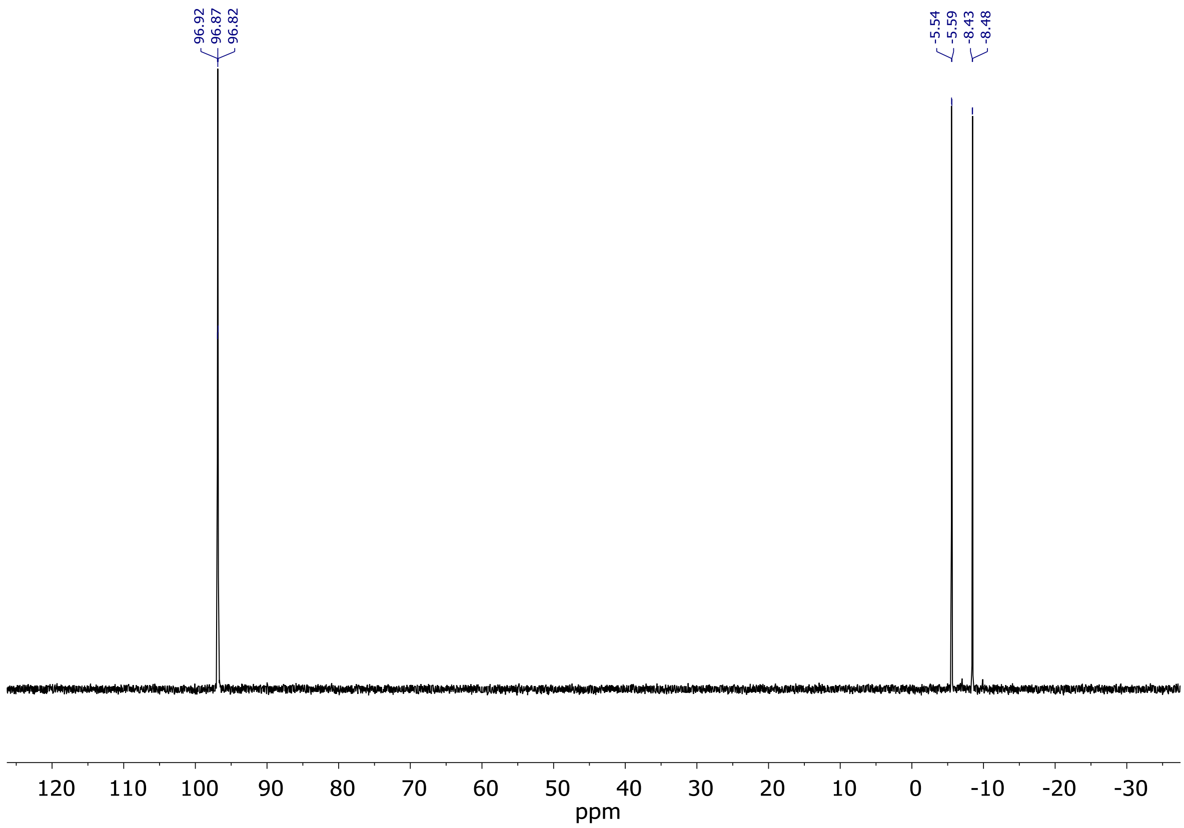

Figure S3b. ^{31}P NMR spectrum of **3** (202 MHz, 298 K, $CDCl_3$).

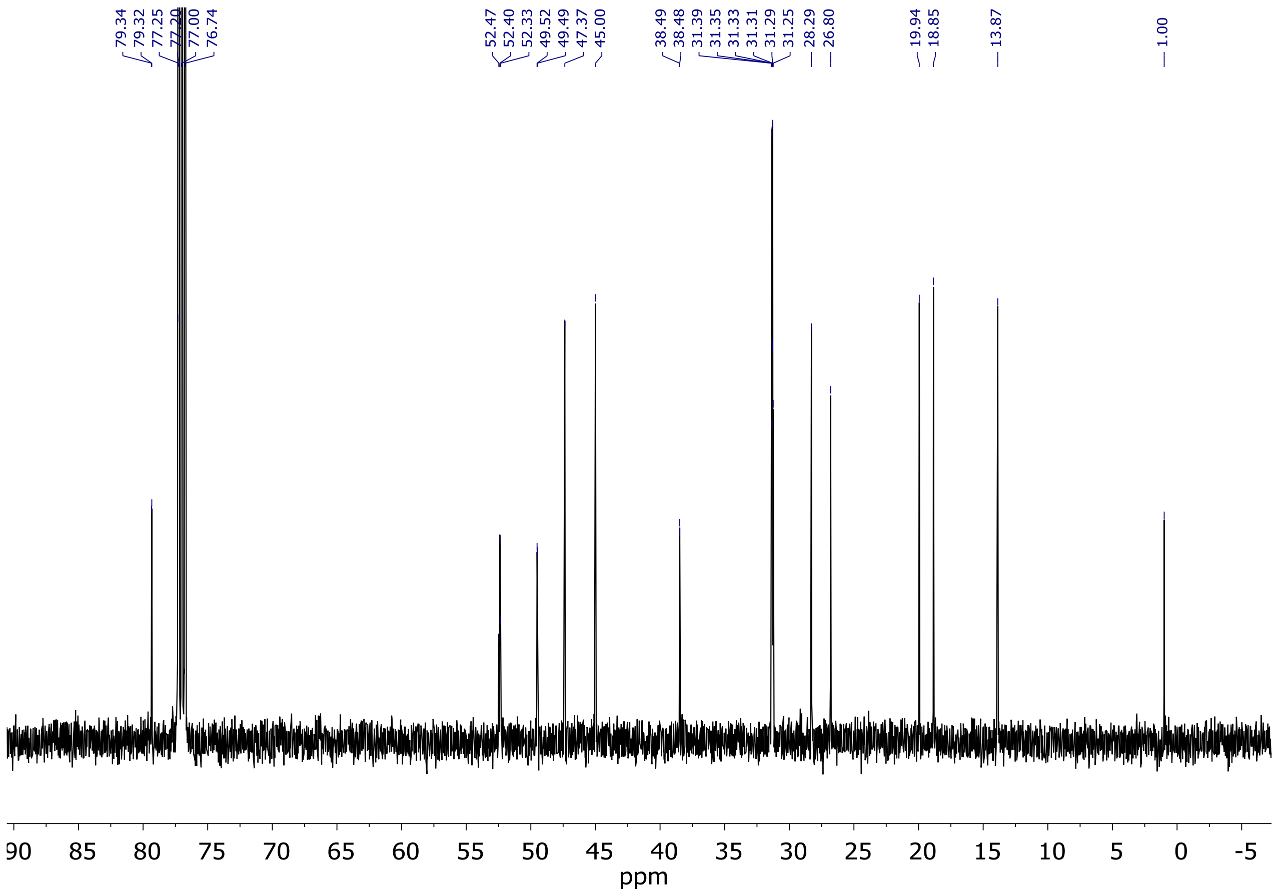

Figure S3c. $^{13}C\{^{1}H\}$ NMR spectrum of **3** (126 MHz, 298 K, $CDCl_3$).

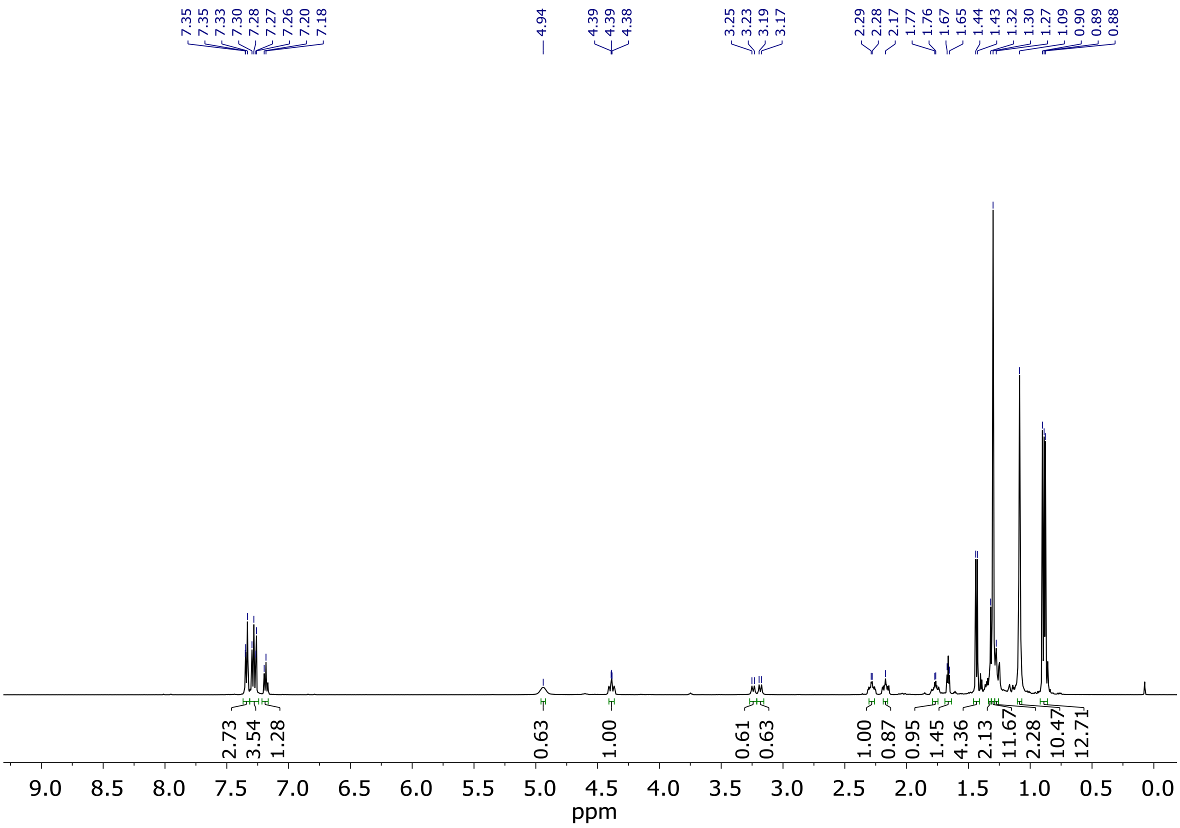

Figure S4a. ^{1}H NMR spectrum of **4**-*R* (500 MHz, 298 K, $CDCl_3$).

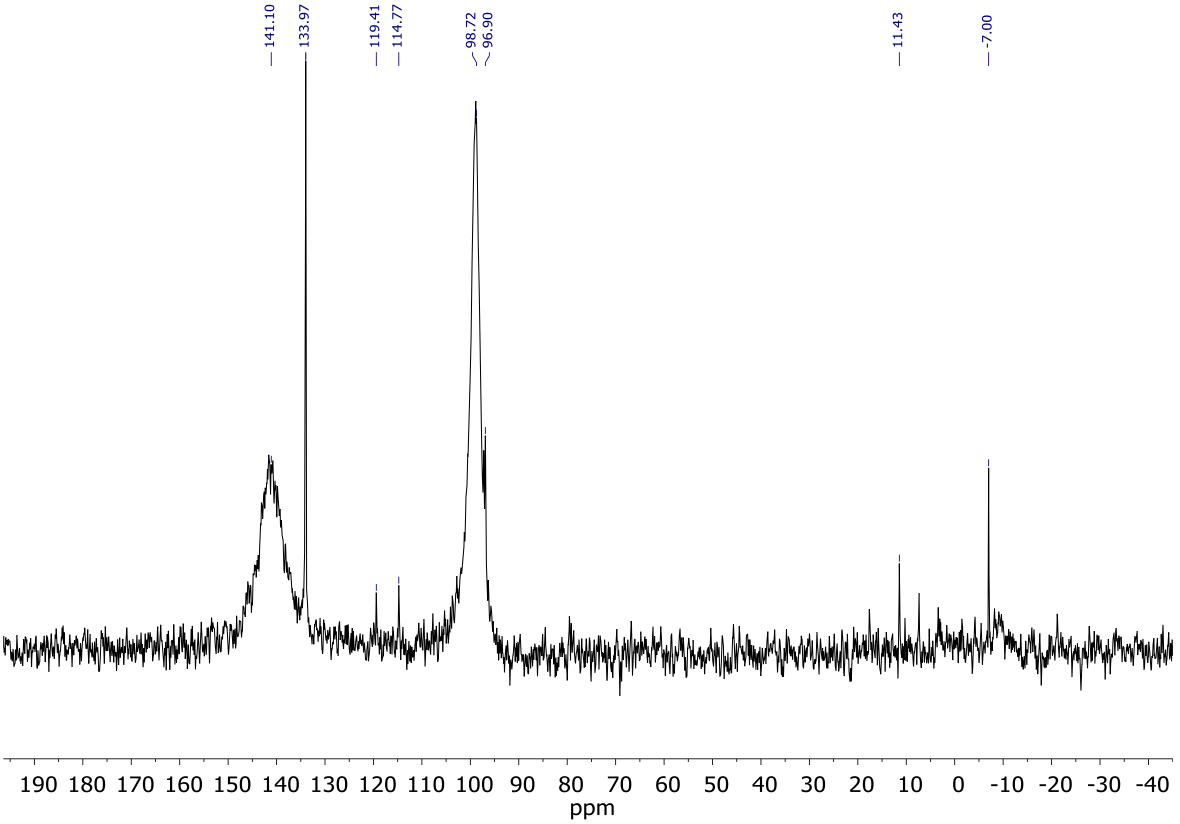

Figure S4b. $^{31}P\{^{1}H\}$ NMR spectrum of **4**-*R* (202 MHz, 298 K, $CDCl_3$).

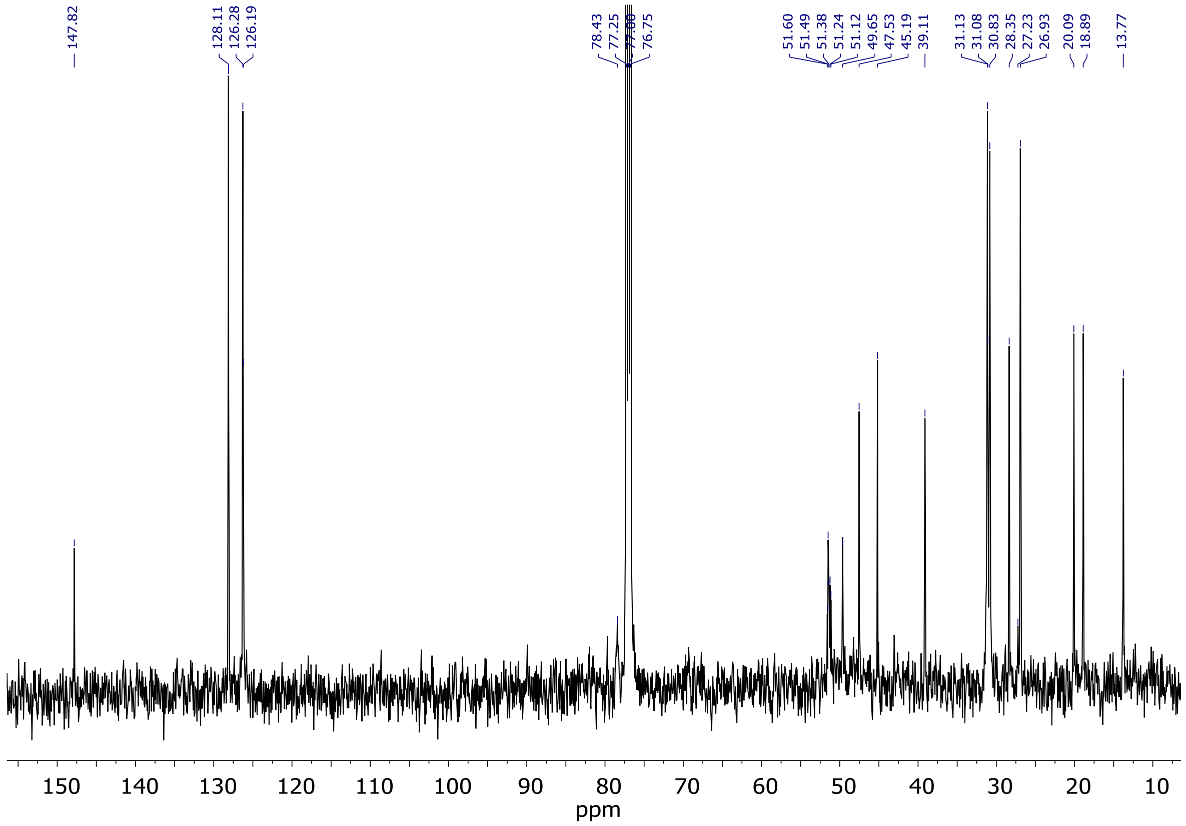

Figure S4c. $^{13}C\{^{1}H\}$ NMR spectrum of **4**-*R* (126 MHz, 298 K, $CDCl_3$).

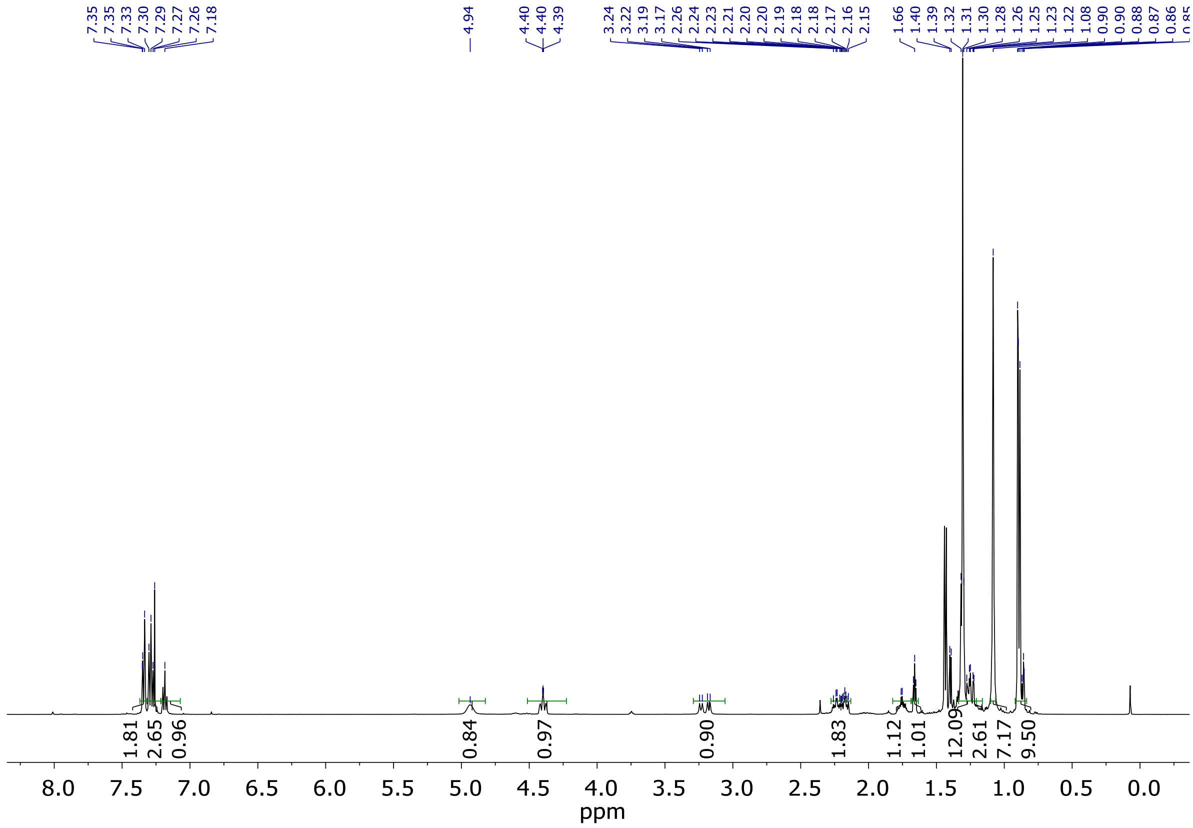

Figure S5a. ^{1}H NMR spectrum of **4**-*S* (500 MHz, 298 K, $CDCl_3$).

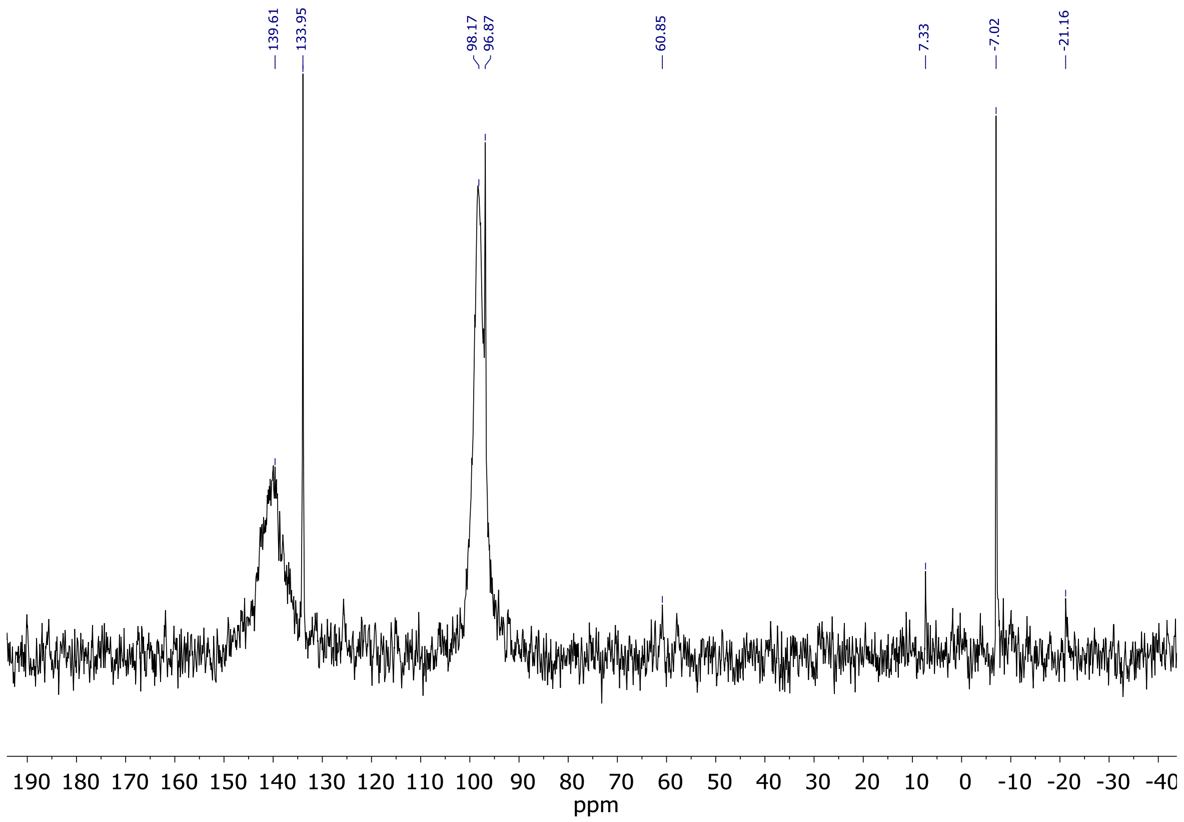

Figure S5b. $^{31}P\{^1H\}$ NMR spectrum of **4**-*S* (202 MHz, 298 K, $CDCl_3$).

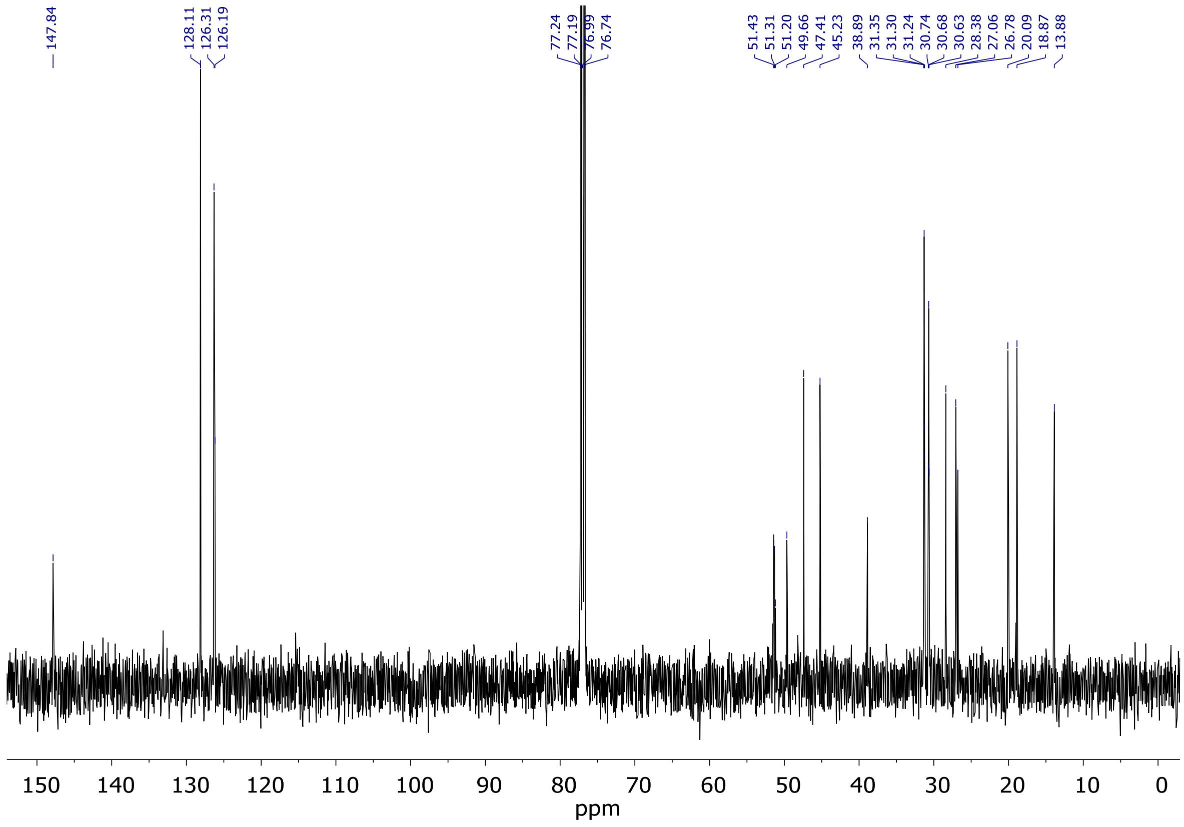

Figure S5c. $^{13}C\{^1H\}$ NMR spectrum of **4**-*S* (126 MHz, 298 K, $CDCl_3$).

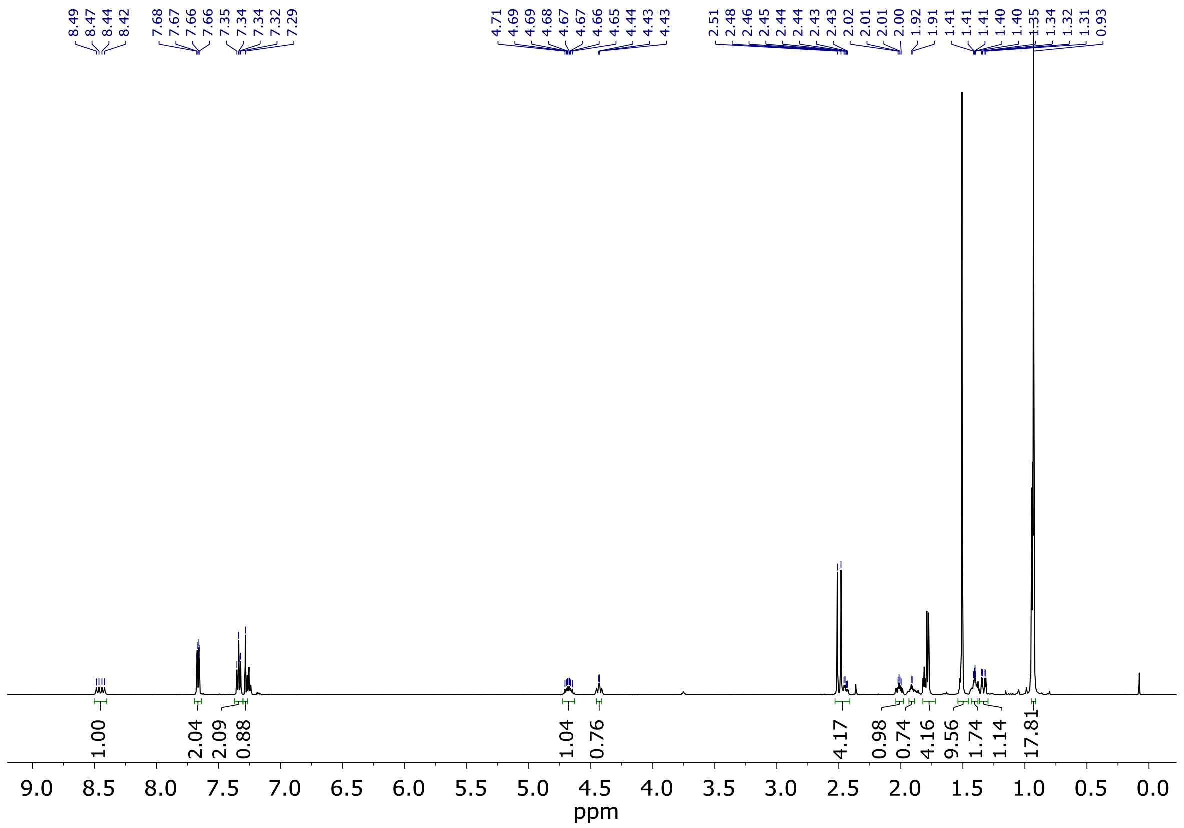

Figure S6a. ^{1}H NMR spectrum of **5**-*R* (500 MHz, 298 K, $CDCl_3$).

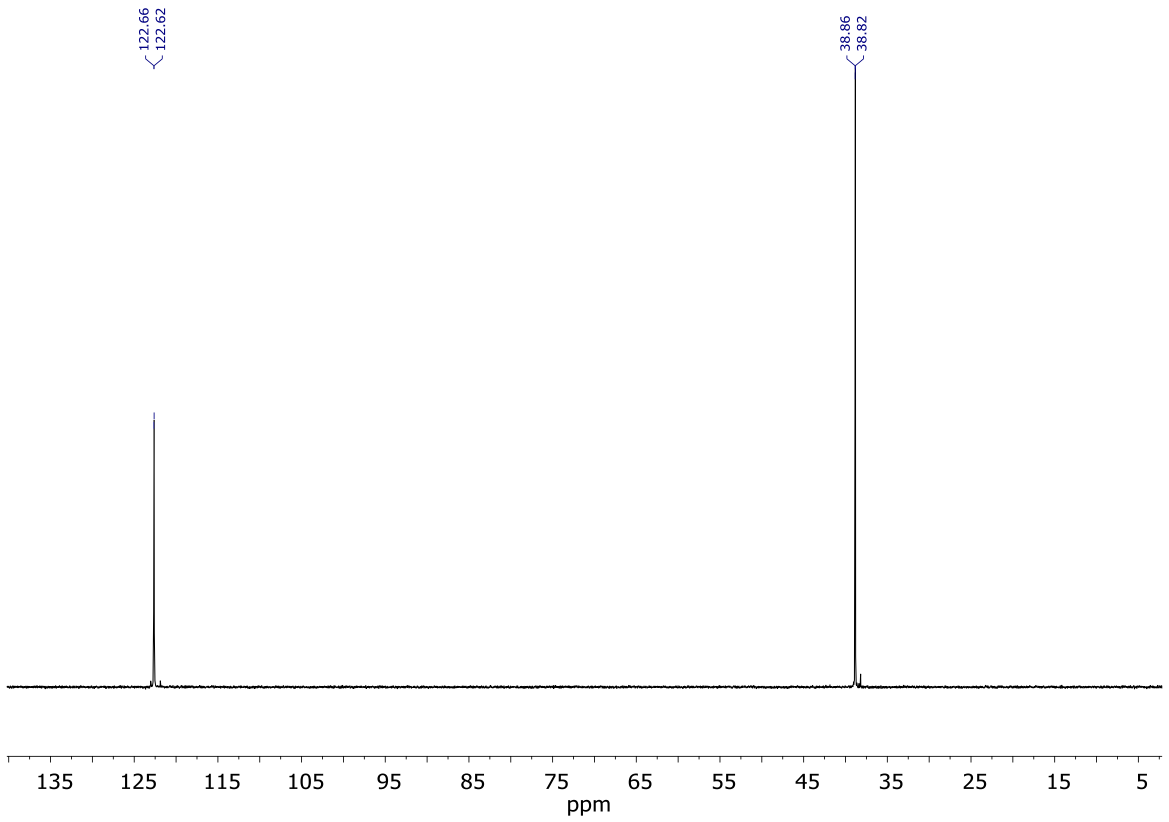

Figure S6b. $^{31}P\{^{1}H\}$ NMR spectrum of **5**-*R* (202 MHz, 298 K, $CDCl_3$).

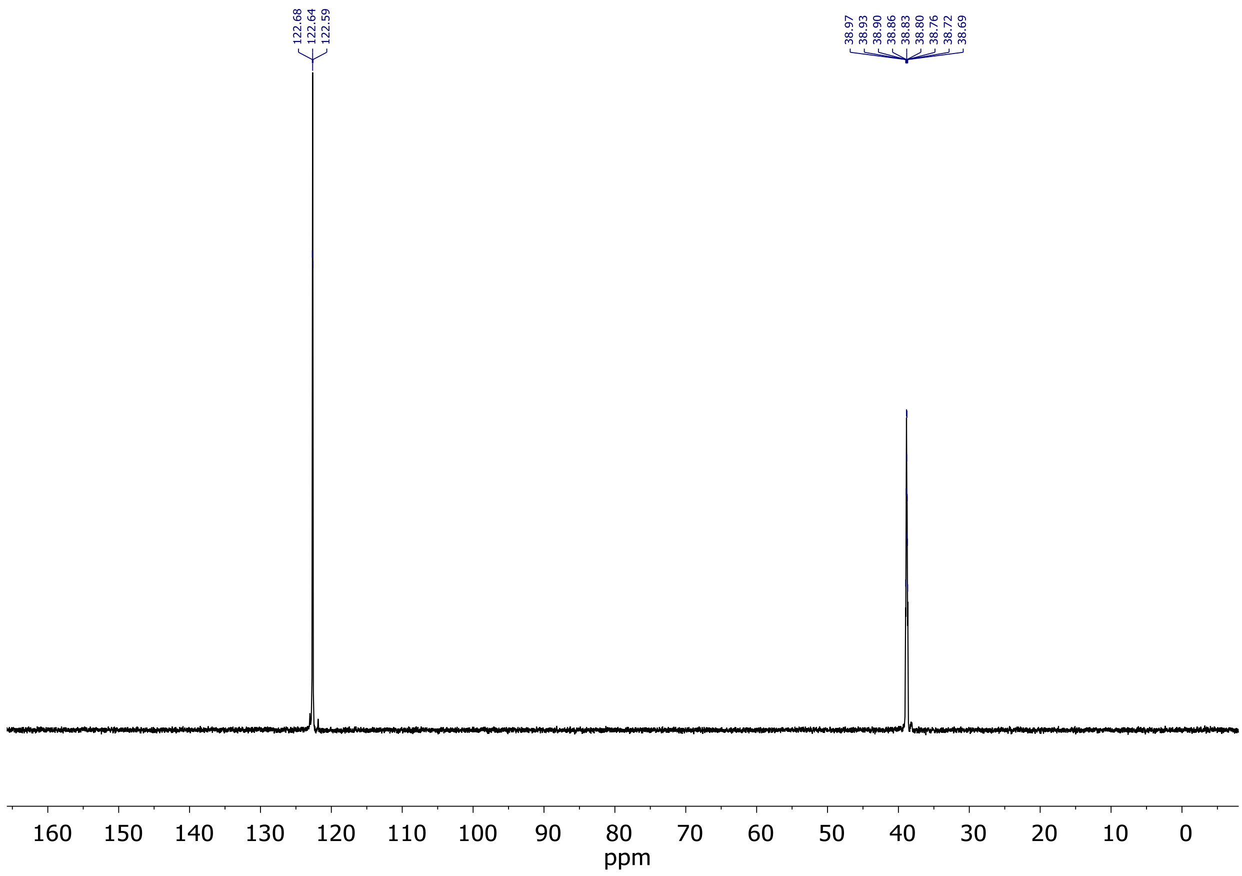

Figure S6c. ^{31}P NMR spectrum of **5**-*R* (202 MHz, 298 K, $CDCl_3$).

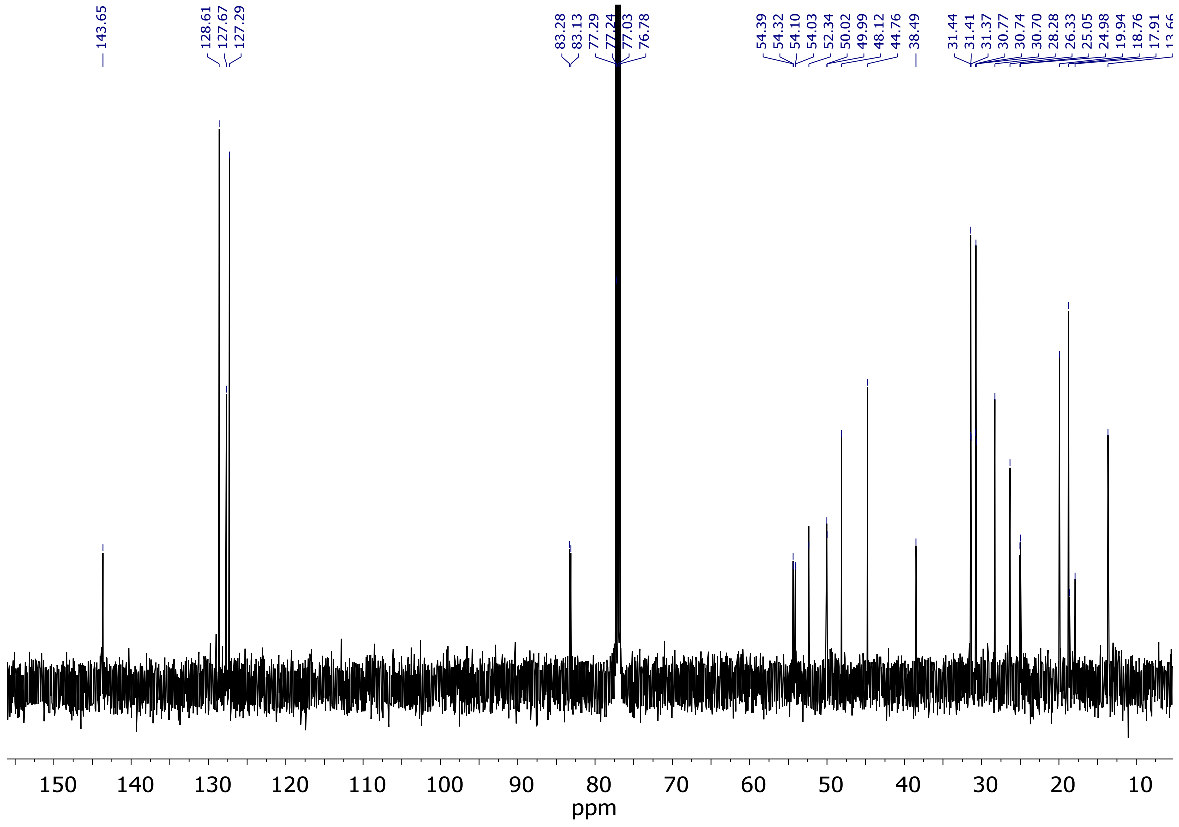

Figure S6d. $^{13}C\{^{1}H\}$ NMR spectrum of **5**-*R* (126 MHz, 298 K, $CDCl_3$).

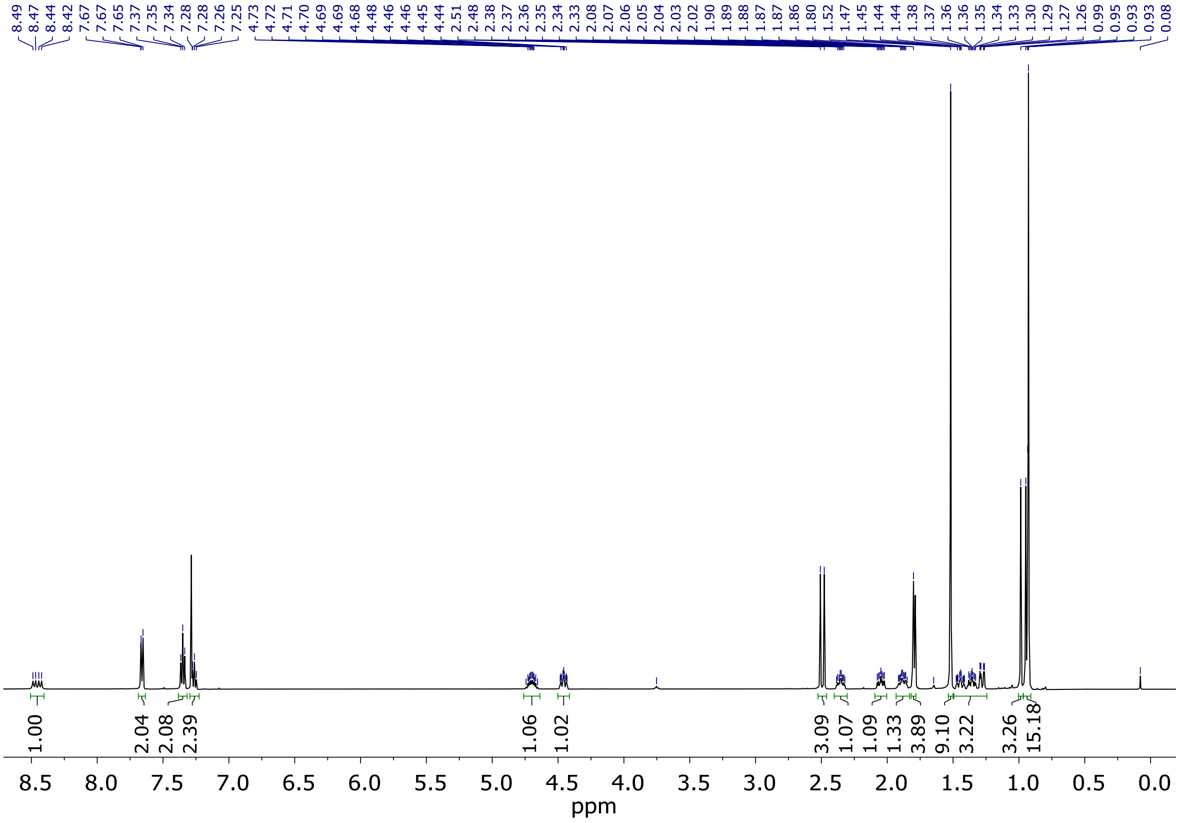

Figure S7a. ^{1}H NMR spectrum of **5**-*S* (500 MHz, 298 K, $CDCl_3$).

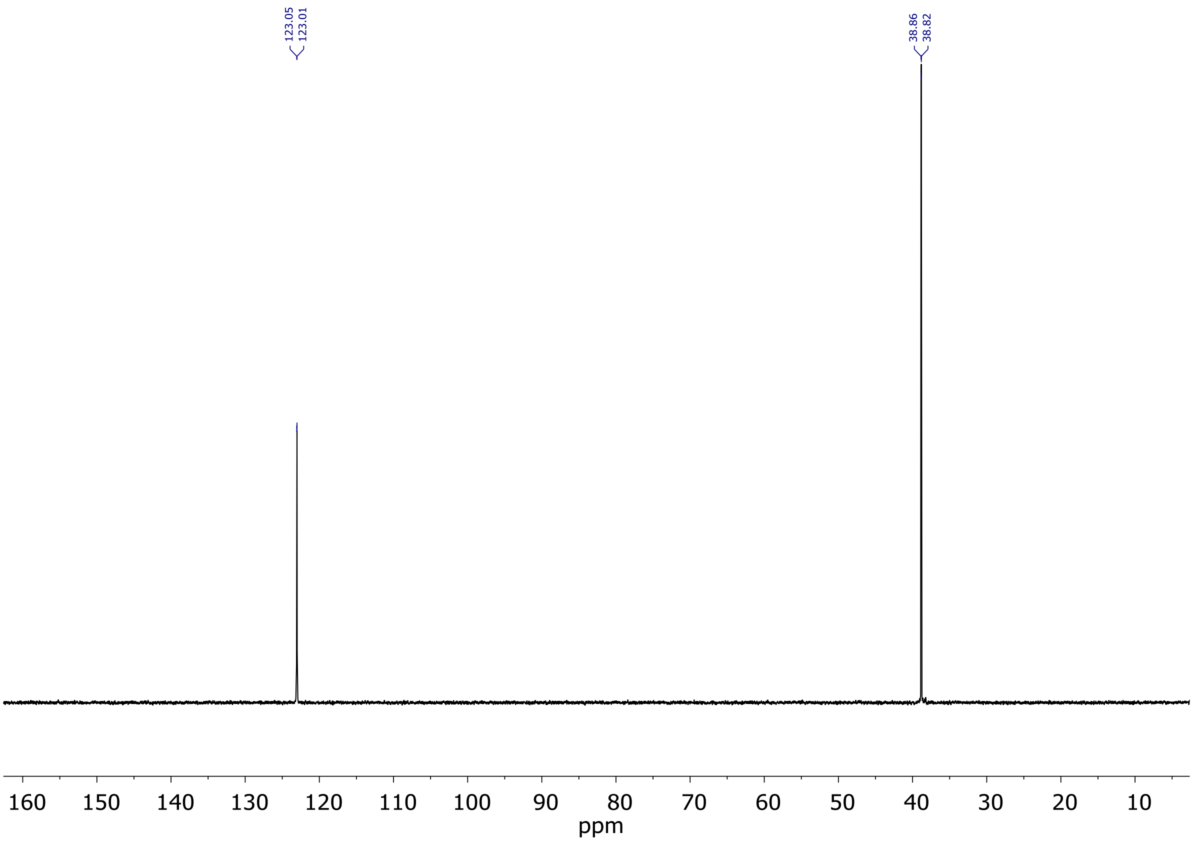

Figure S7b. $^{31}P\{^{1}H\}$ NMR spectrum of **5**-*S* (202 MHz, 298 K, $CDCl_3$).

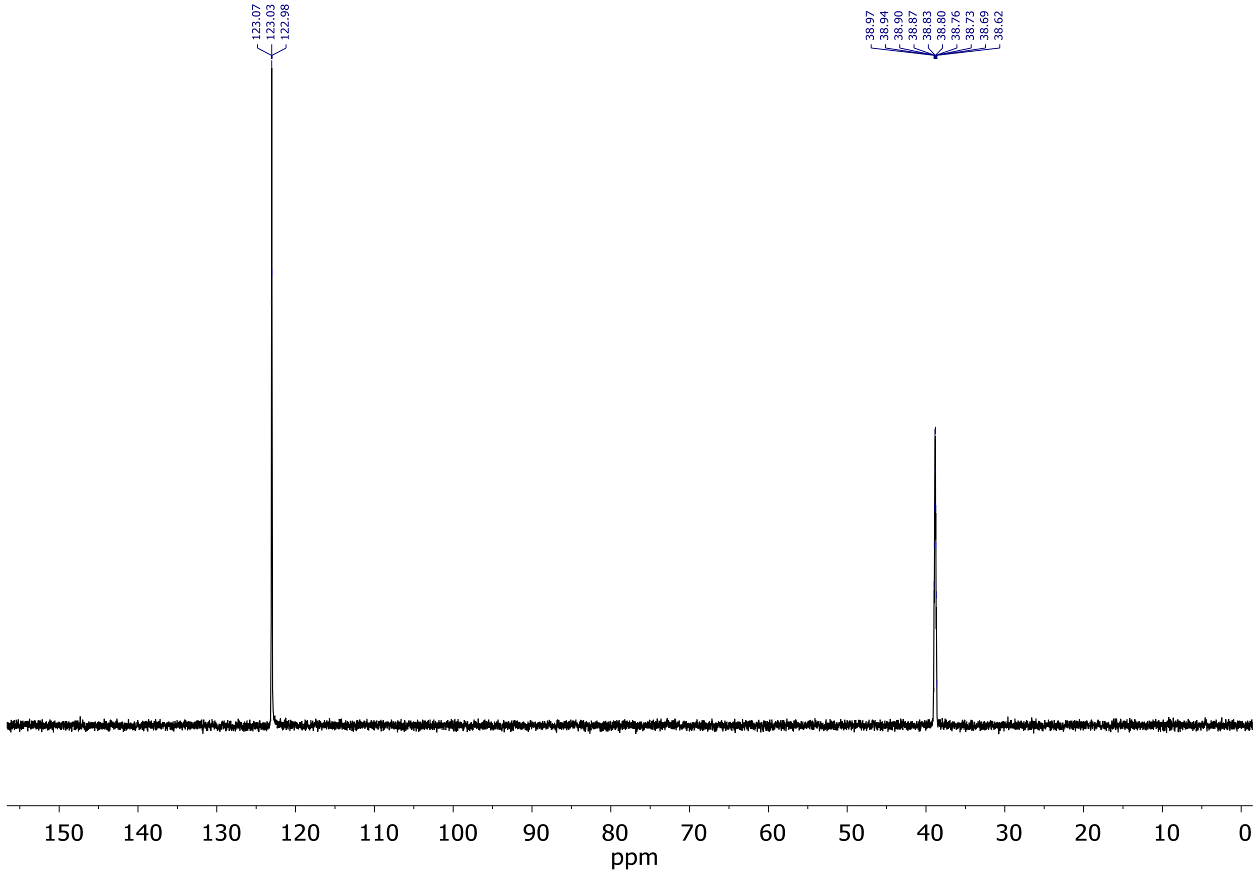

Figure S7c. ^{31}P NMR spectrum of **5**-*S* (202 MHz, 298 K, $CDCl_3$).

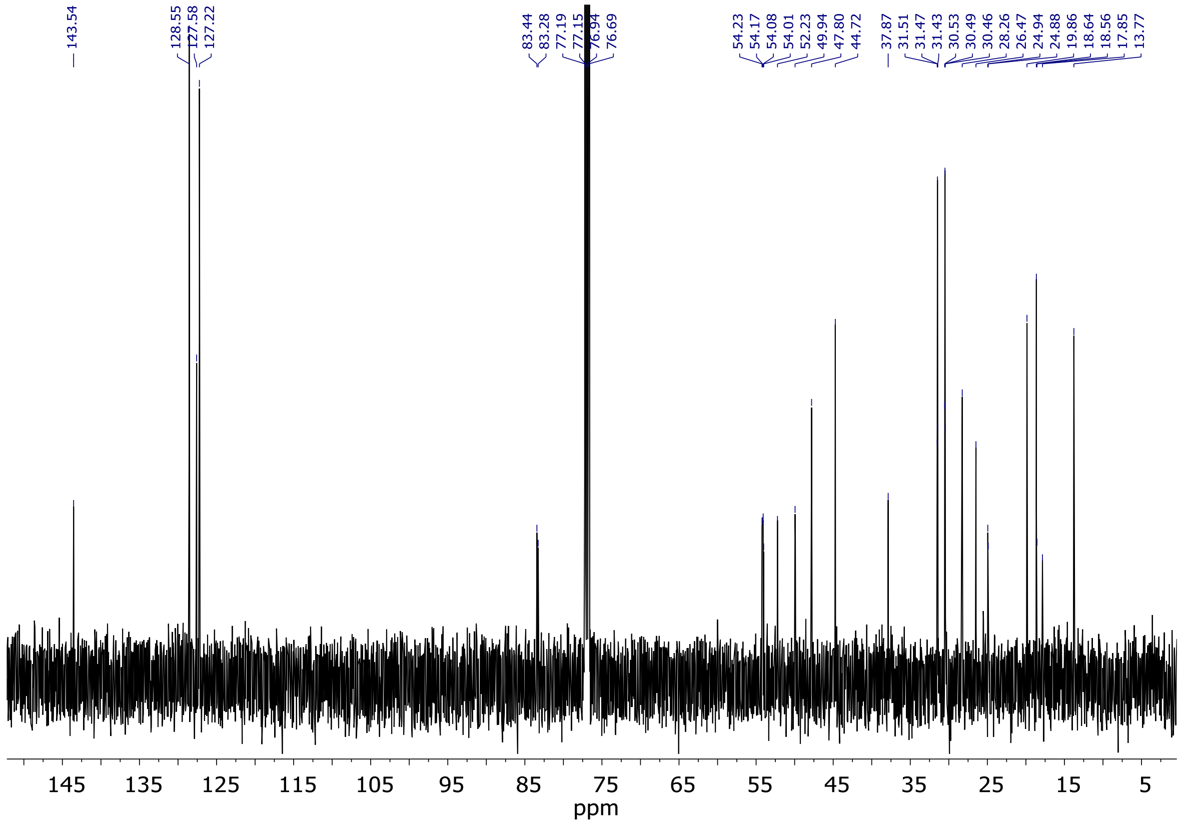

Figure S7d. $^{13}C\{^{1}H\}$ NMR spectrum of **5**-*S* (126 MHz, 298 K, $CDCl_3$).

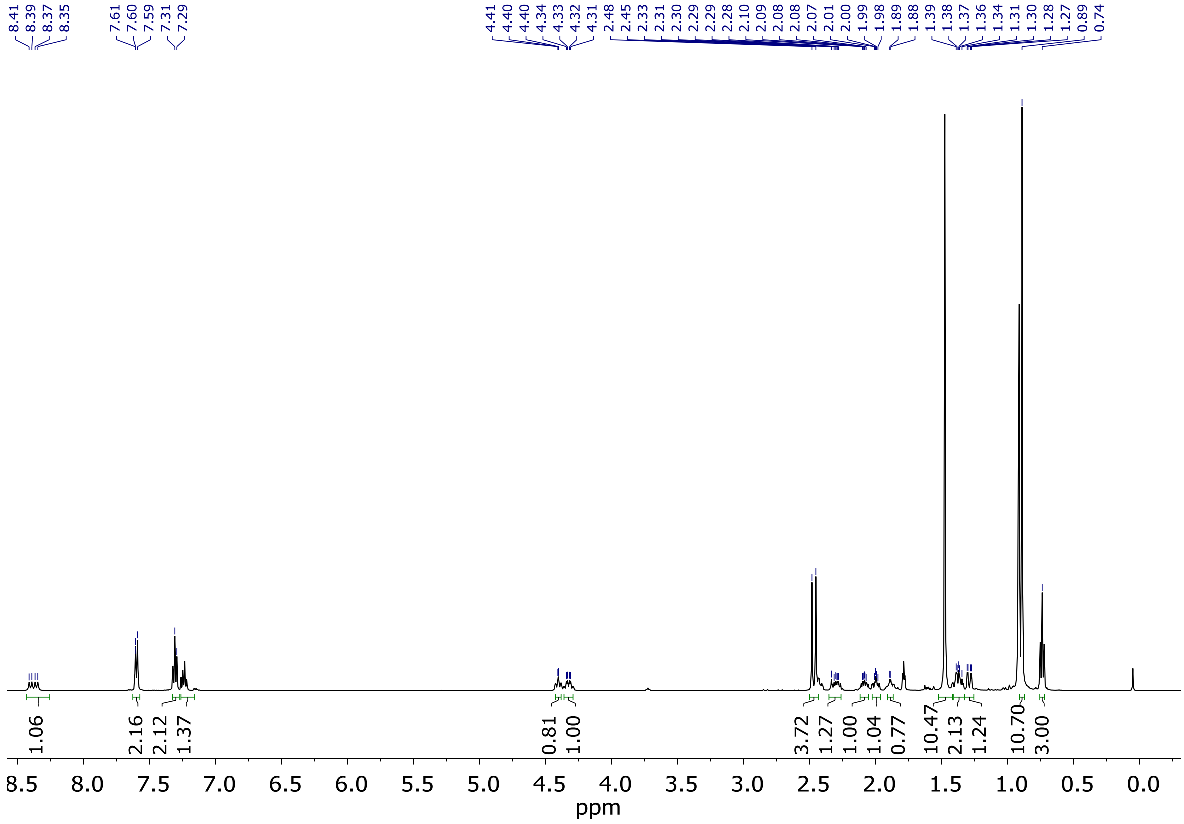

Figure S8a. ^{1}H NMR spectrum of **6**-*R* (500 MHz, 298 K, $CDCl_3$).

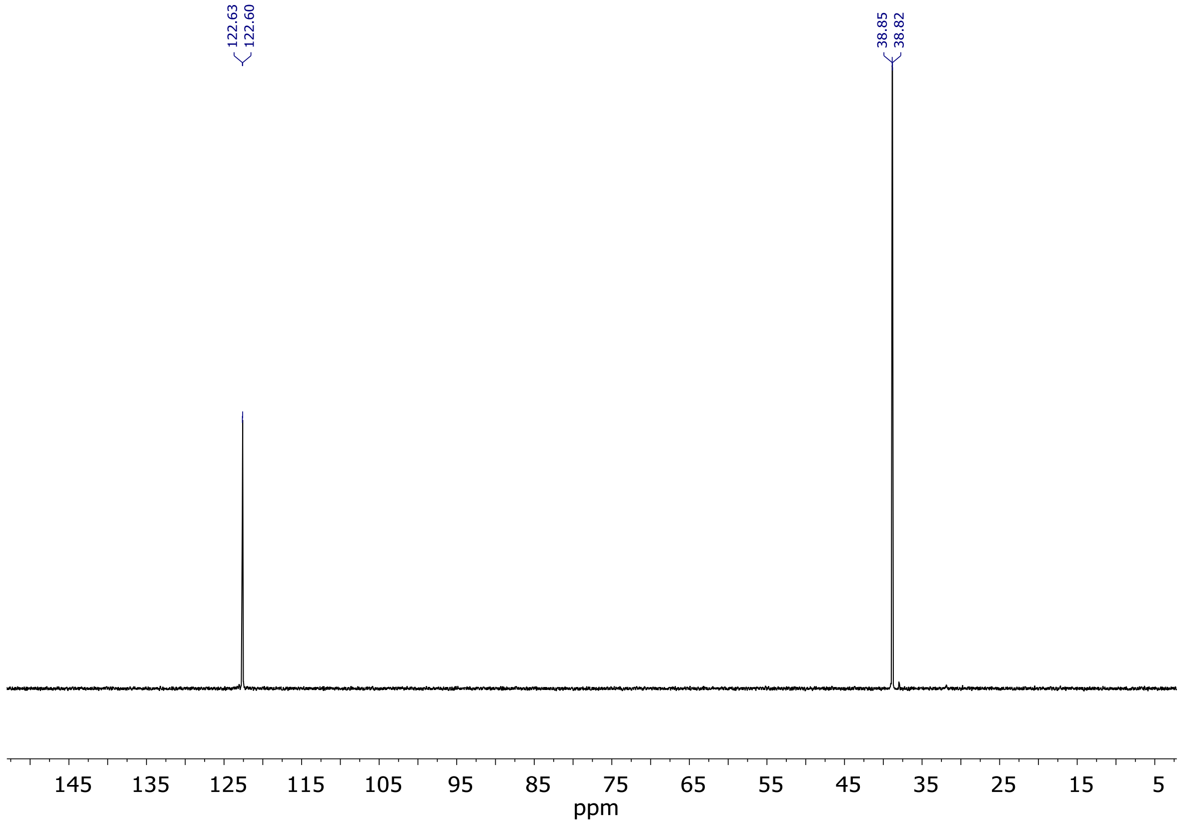

Figure S8b. $^{31}P\{^{1}H\}$ NMR spectrum of **6**-*R* (202 MHz, 298 K, $CDCl_3$).

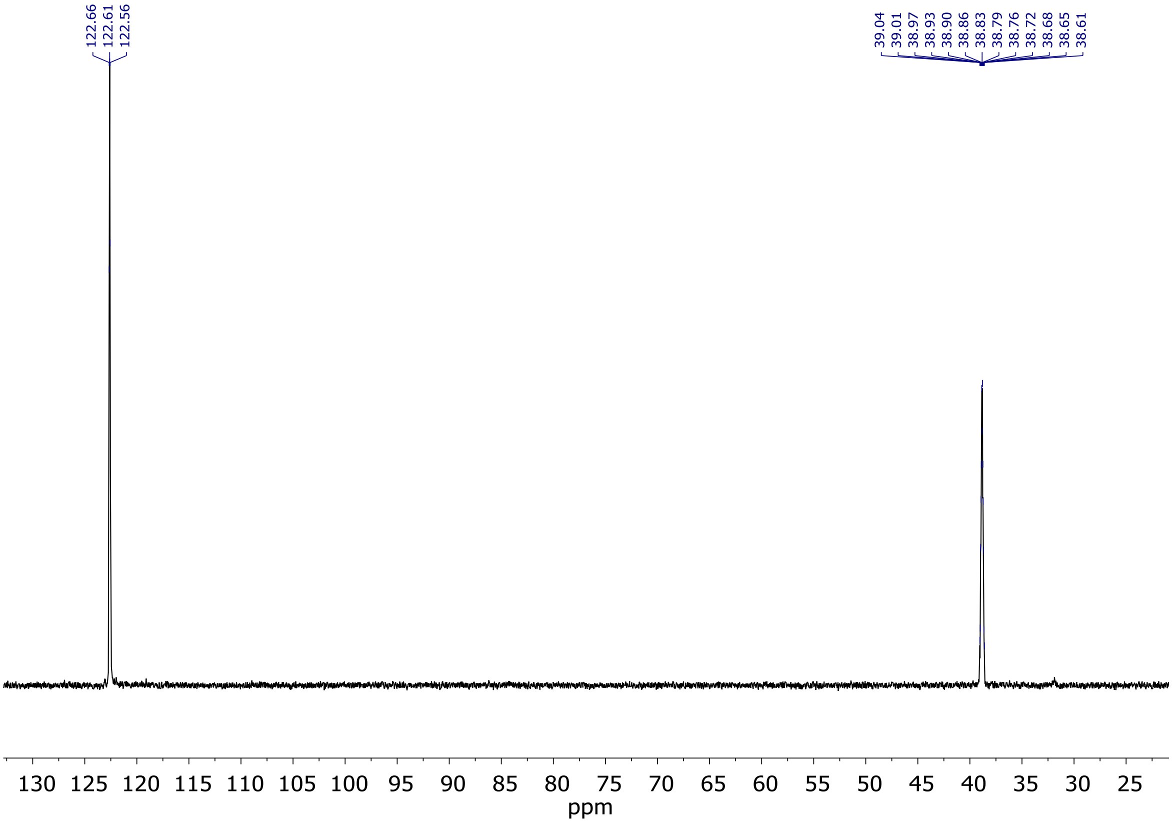

Figure S8c. ^{31}P NMR spectrum of **6**-*R* (202 MHz, 298 K, $CDCl_3$).

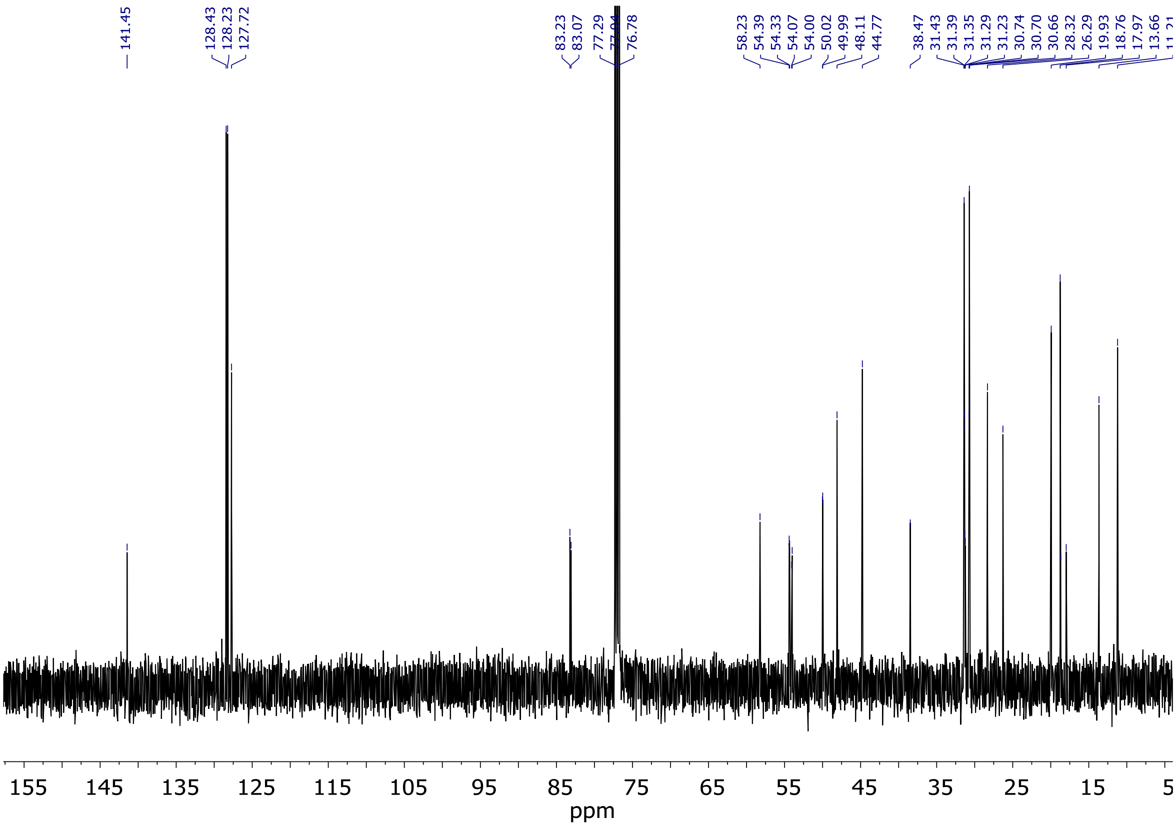

Figure S8d. $^{13}C\{^1H\}$ NMR spectrum of **6**-*R* (126 MHz, 298 K, $CDCl_3$).

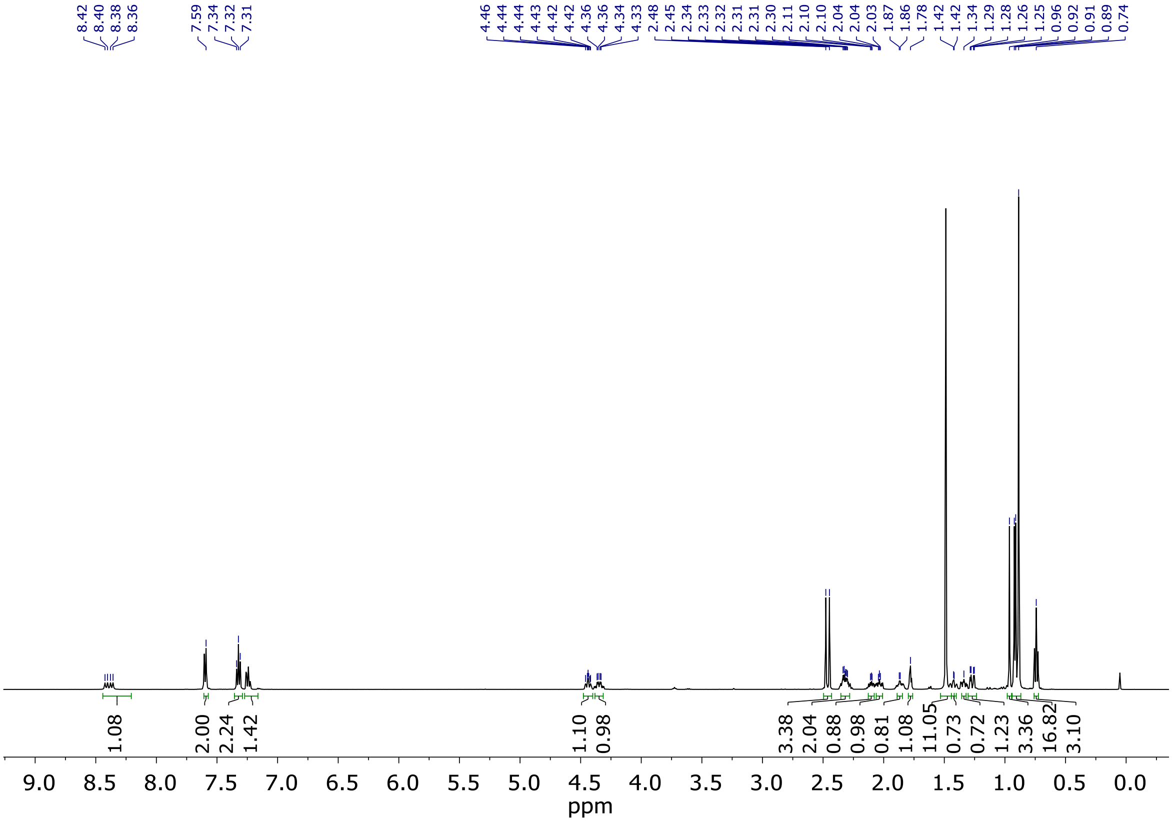

Figure S9a. ^{1}H NMR spectrum of **6**-*S* (500 MHz, 298 K, $CDCl_3$).

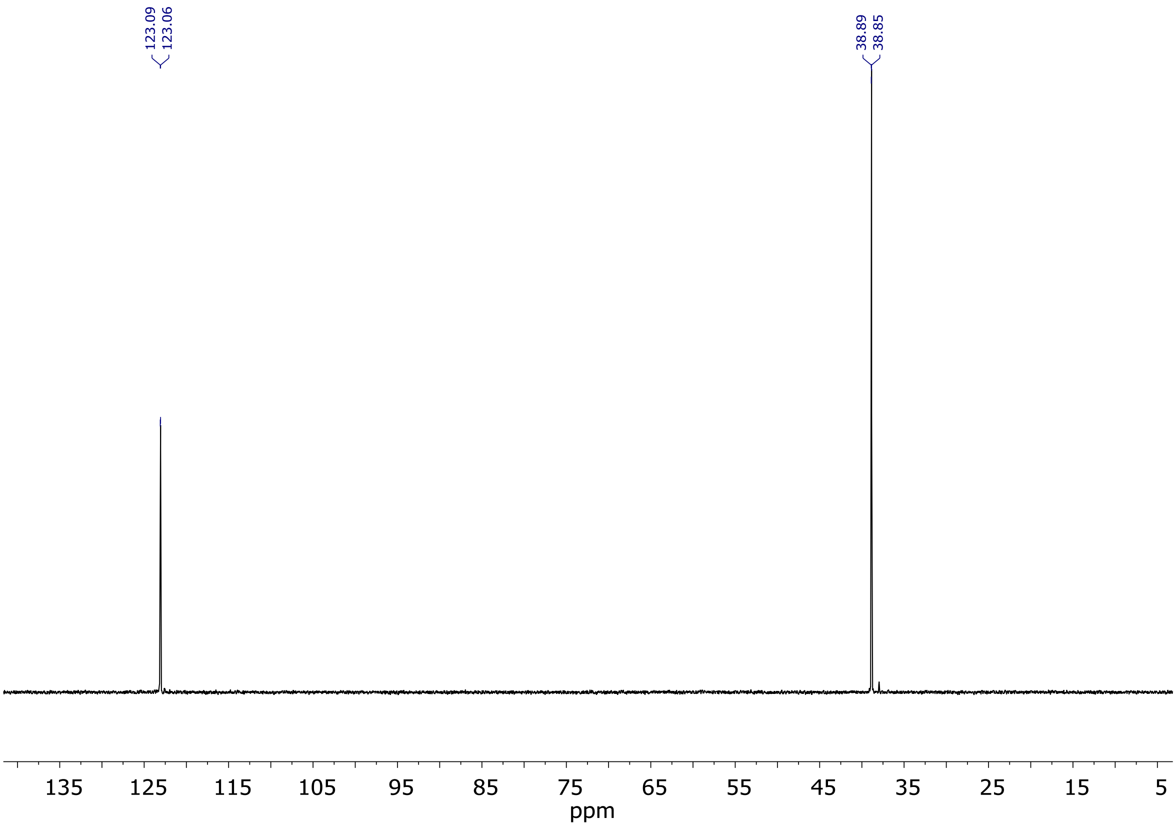

Figure S9b. $^{31}P\{^{1}H\}$ NMR spectrum of **6**-*S* (202 MHz, 298 K, $CDCl_3$).

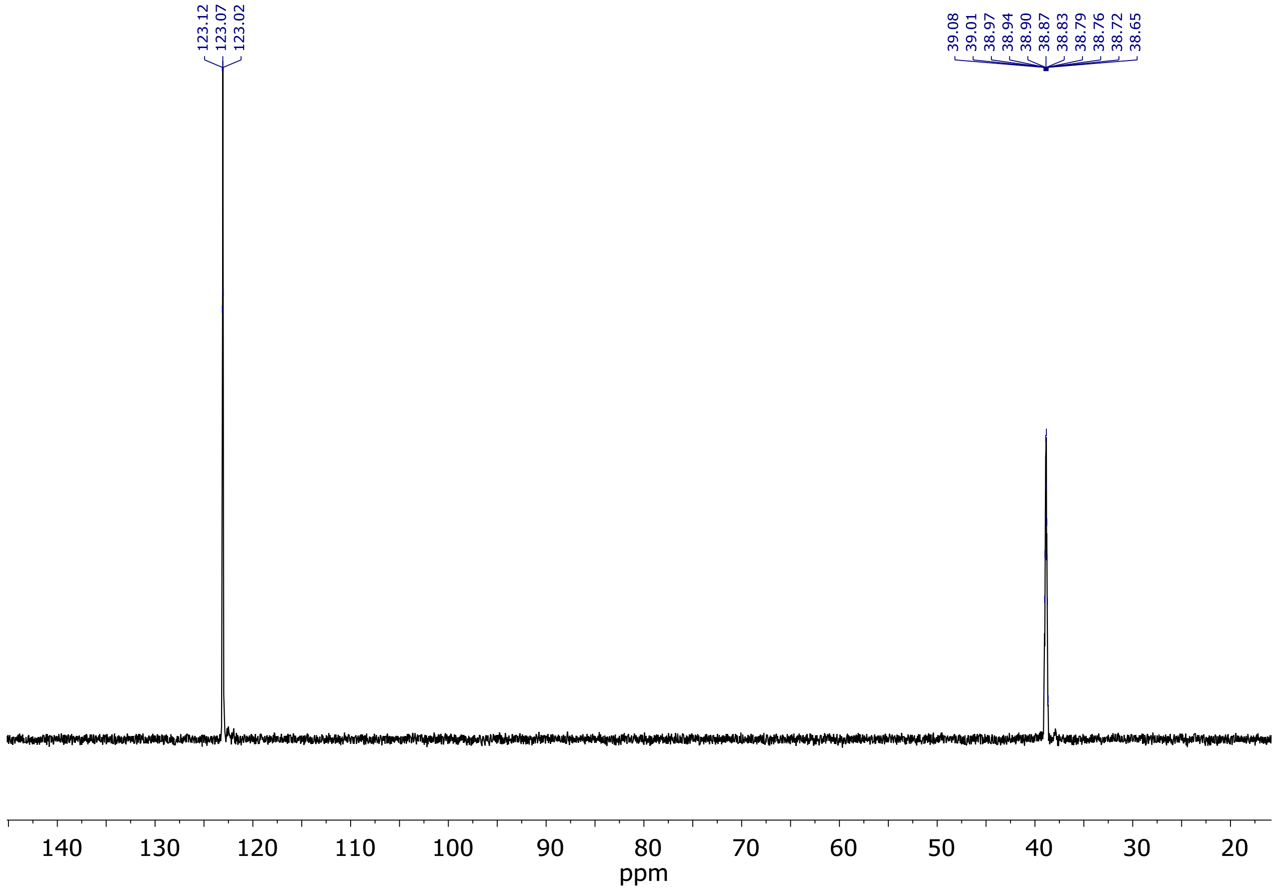

Figure S9c. ^{31}P NMR spectrum of **6**-*S* (202 MHz, 298 K, $CDCl_3$).

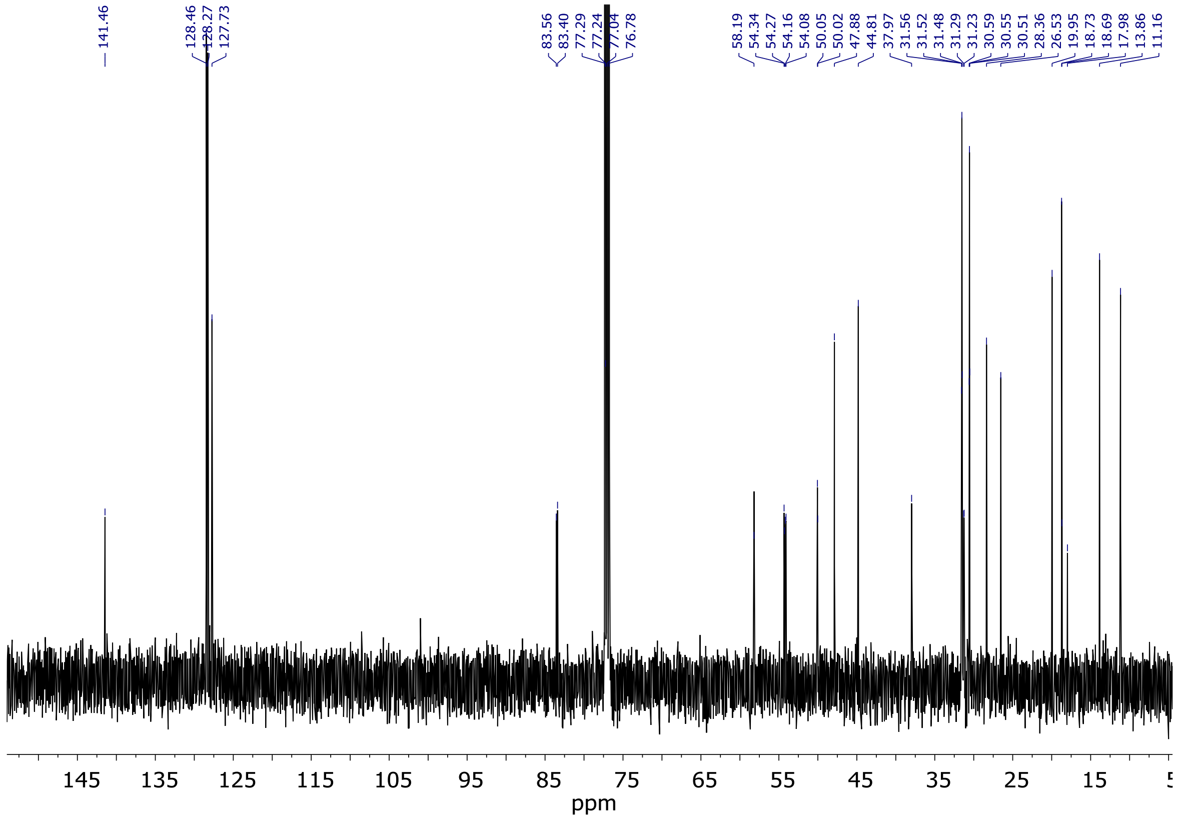

Figure S9d. $^{13}C\{^1H\}$ NMR spectrum of **6**-*S* (126 MHz, 298 K, $CDCl_3$).

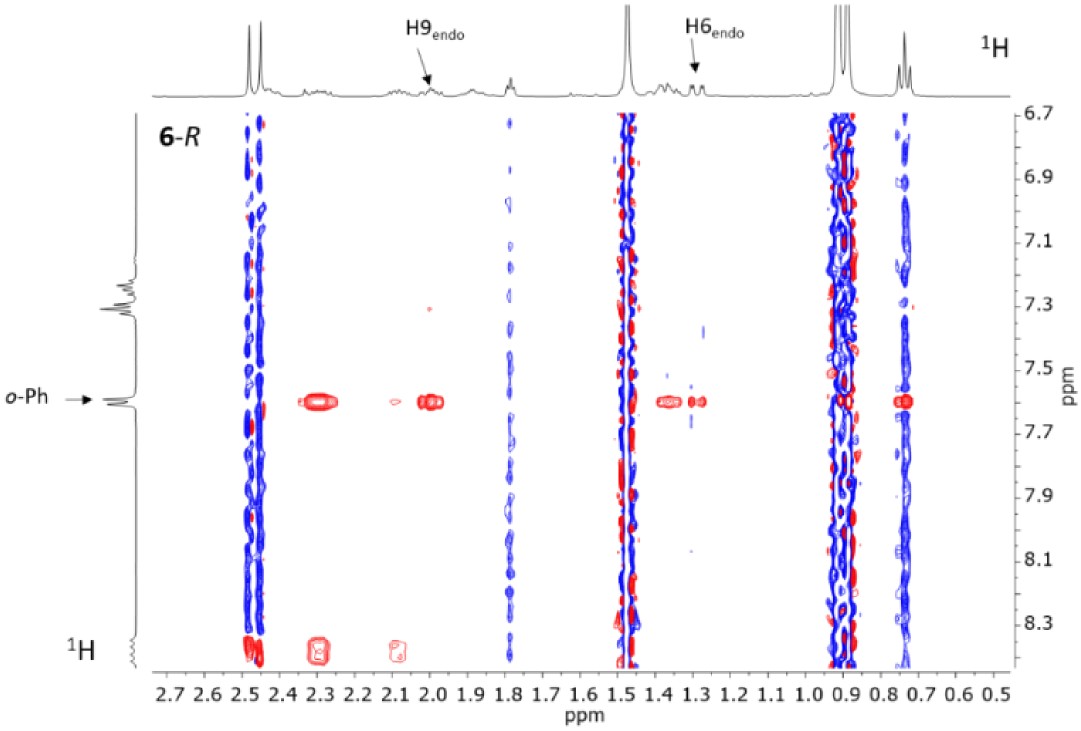

(a)

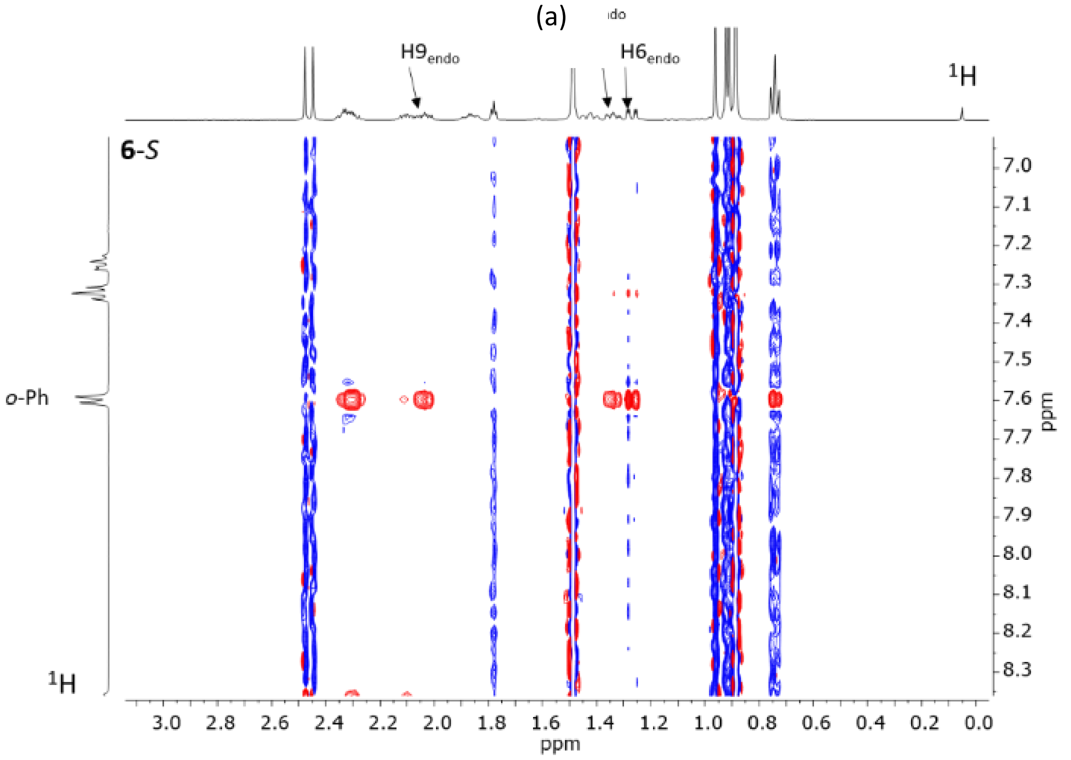

(b)

Figure S9e. Expansions of the NOESY spectrum of (a) **6**-*R* (b) **6**-*S*. The cross peak to $H6_{endo}$ is visibly weaker in the NOESY spectrum of **6**-*R*.

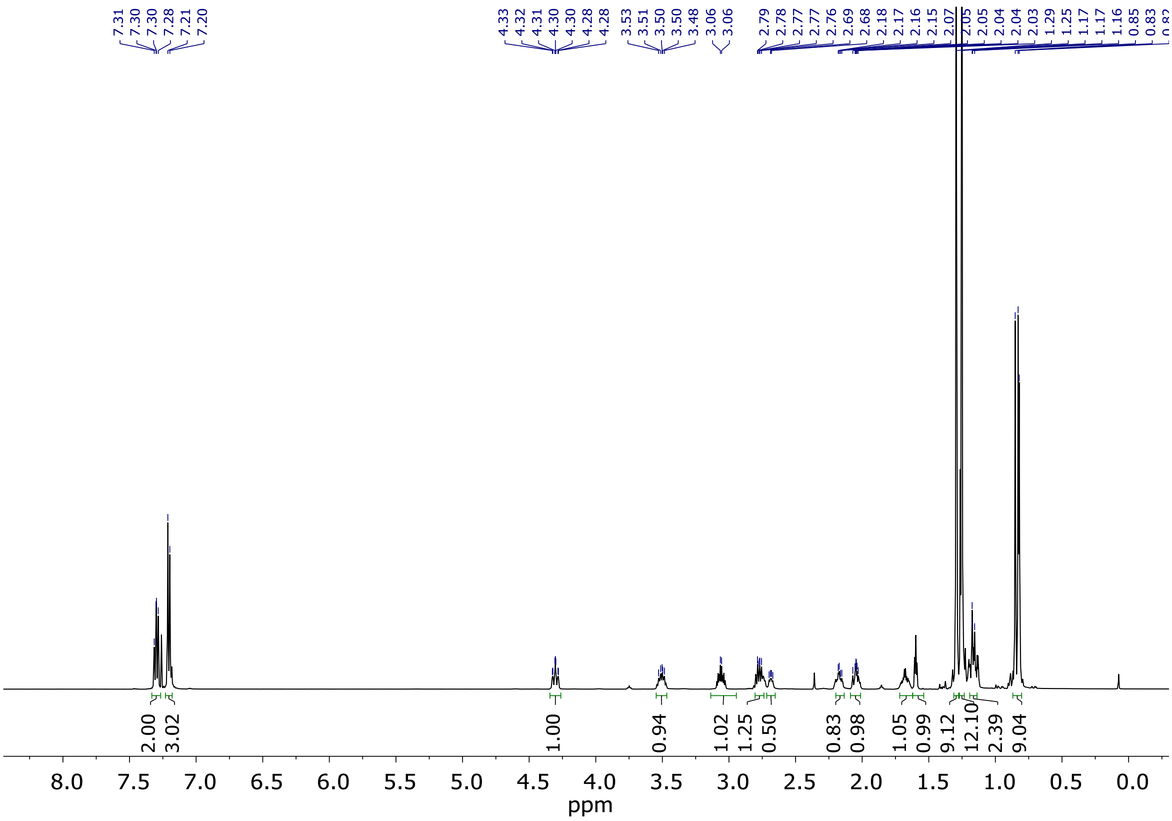

Figure S10a. ^1H NMR spectrum of **7**-*R* (500 MHz, 298 K, $CDCl_3$).

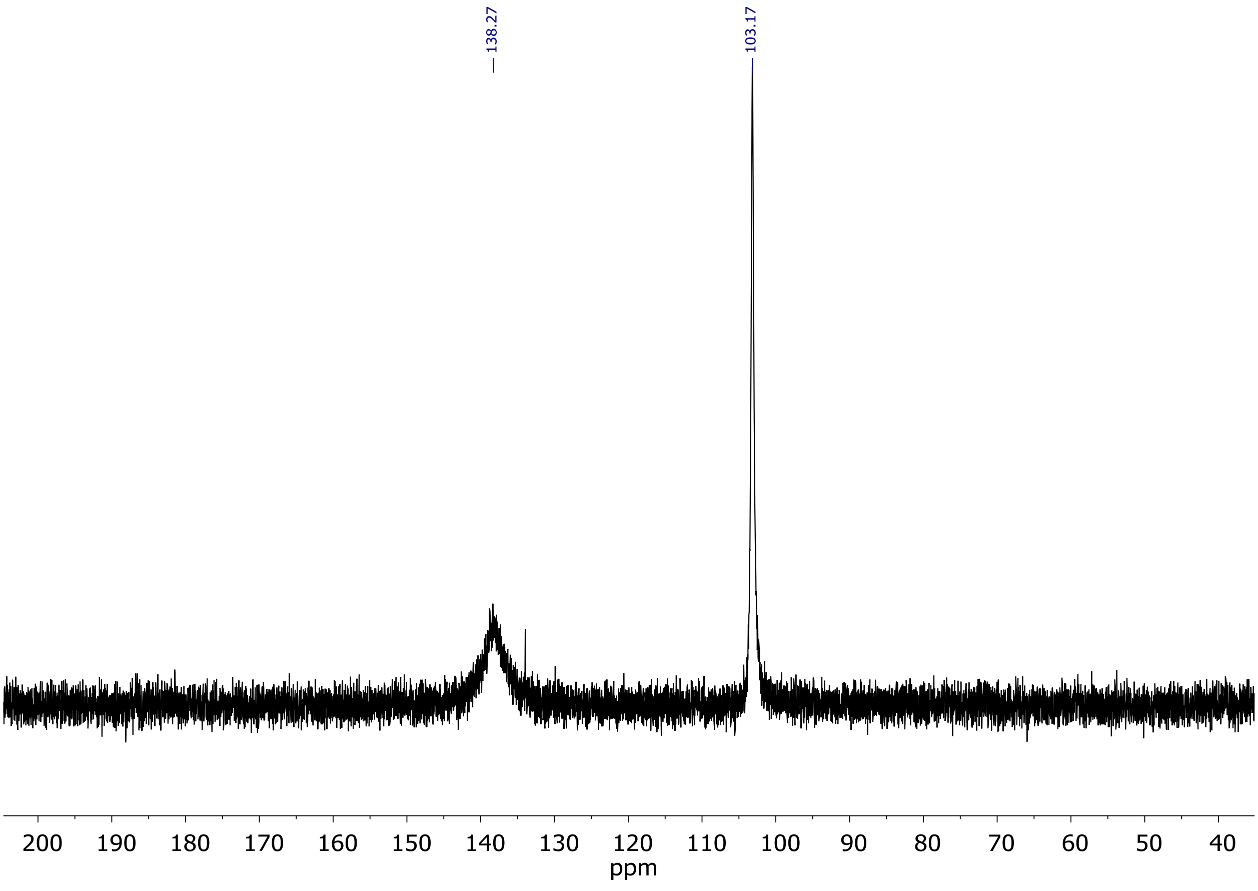

Figure S10b. $^{31}P\{^1H\}$ NMR spectrum of **7**-*R* (202 MHz, 298 K, $CDCl_3$).

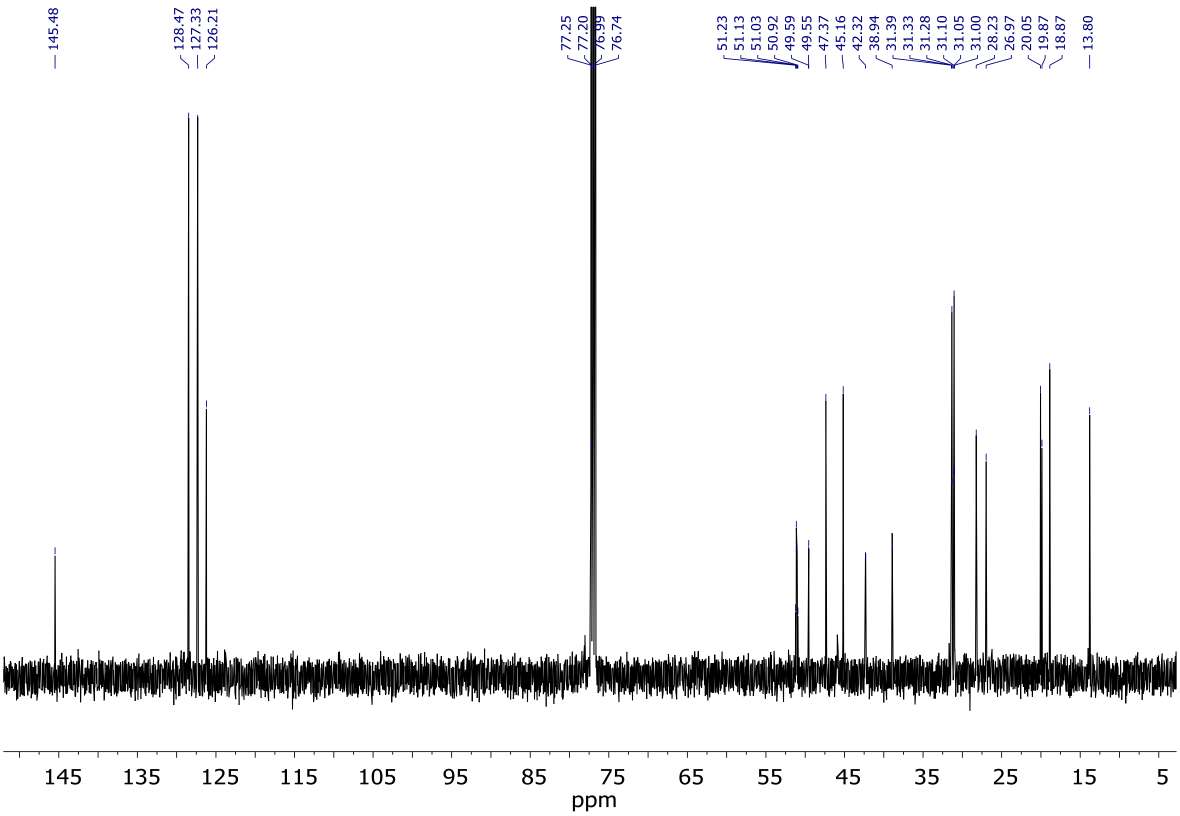

Figure S10c. $^{13}C\{^{1}H\}$ NMR spectrum of **7**-*R* (126 MHz, 298 K, $CDCl_3$).

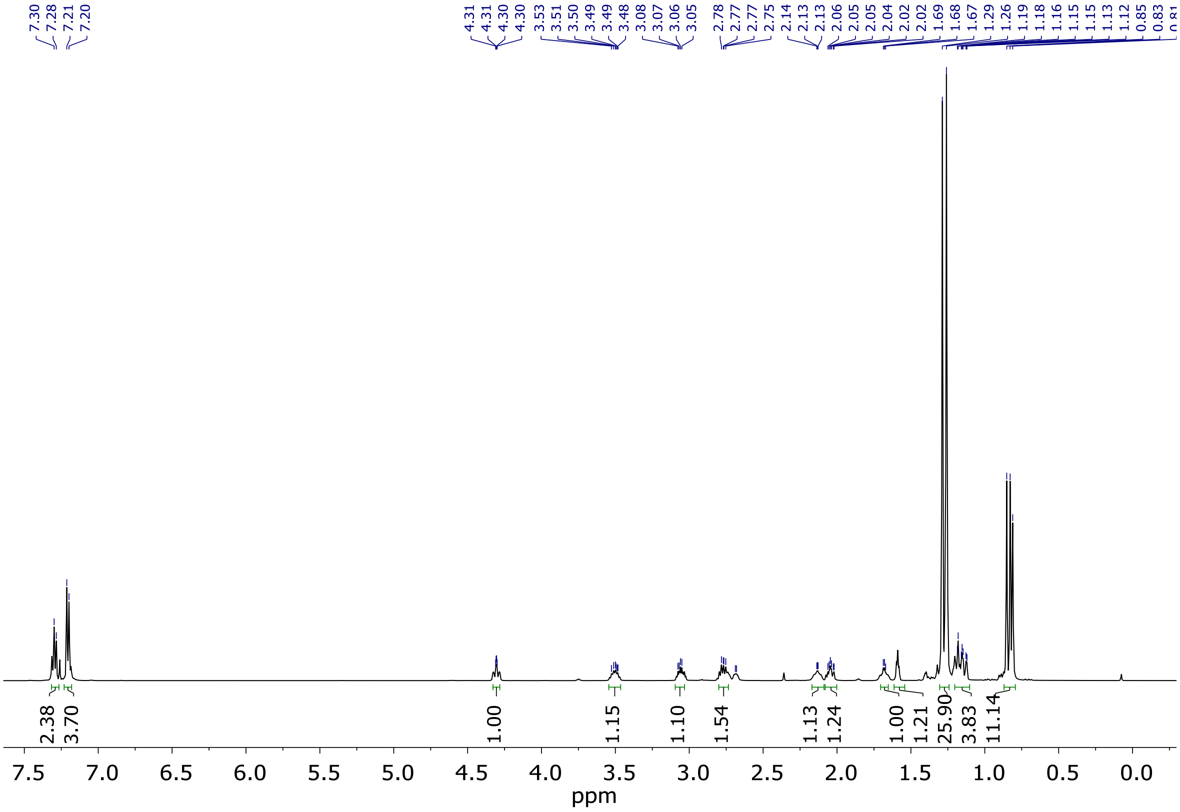

Figure S11a. ^{1}H NMR spectrum of **7**-*S* (500 MHz, 298 K, $CDCl_3$).

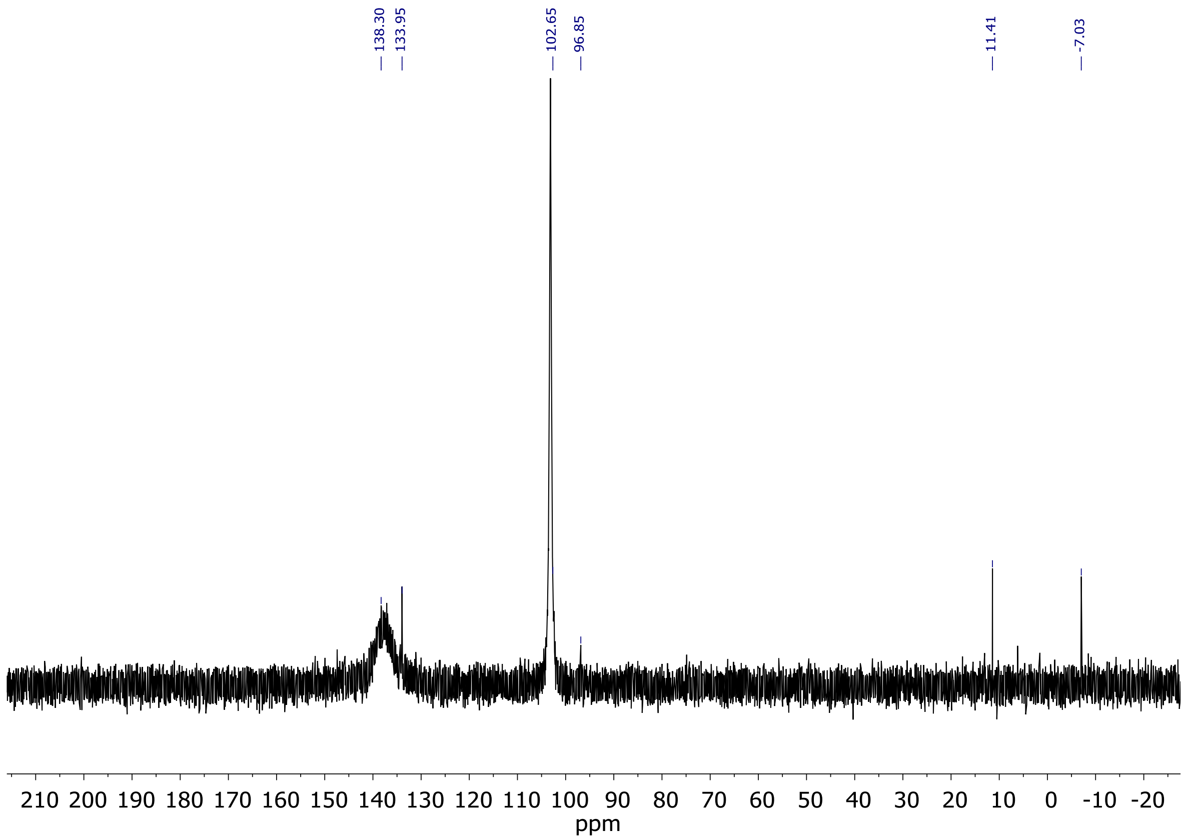

Figure S11b. $^{31}P\{^{1}H\}$ NMR spectrum of **7**-*S* (202 MHz, 298 K, $CDCl_3$).

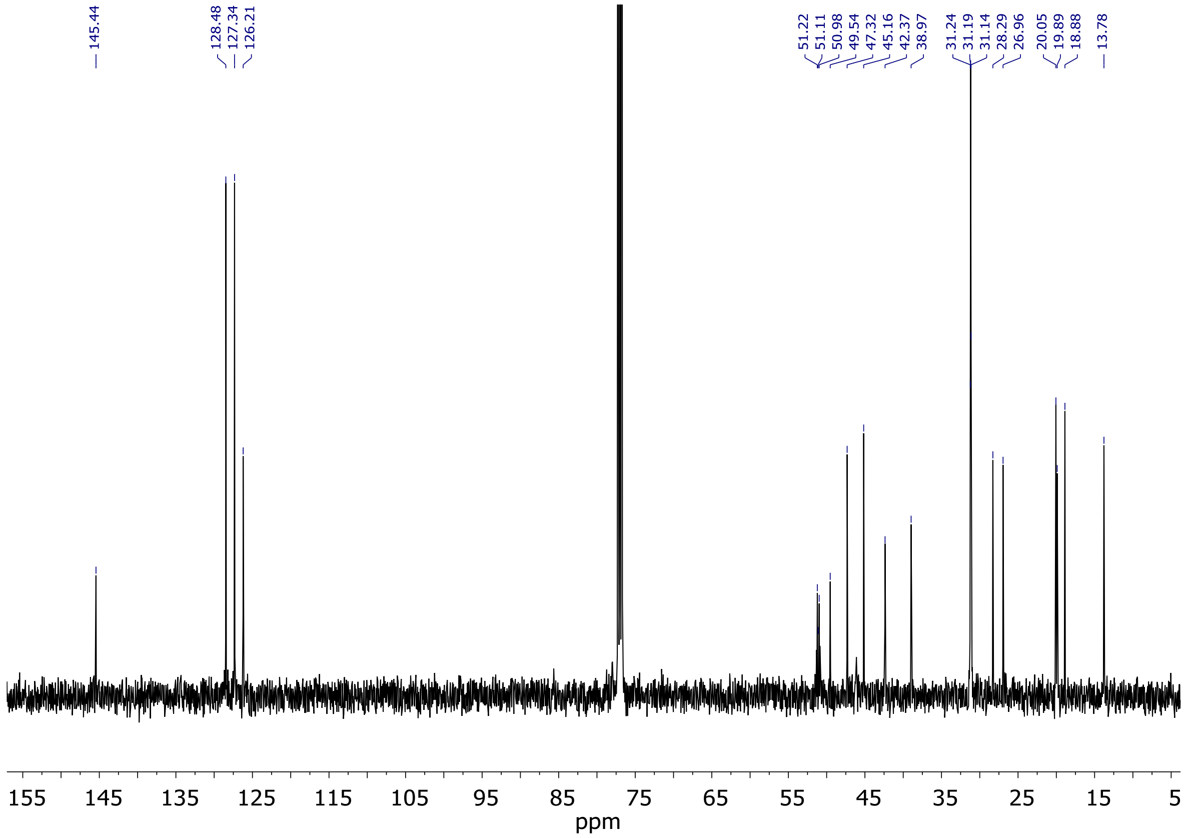

Figure S11c. $^{13}C\{^{1}H\}$ NMR spectrum of **7**-*S* (126 MHz, 298 K, $CDCl_3$).

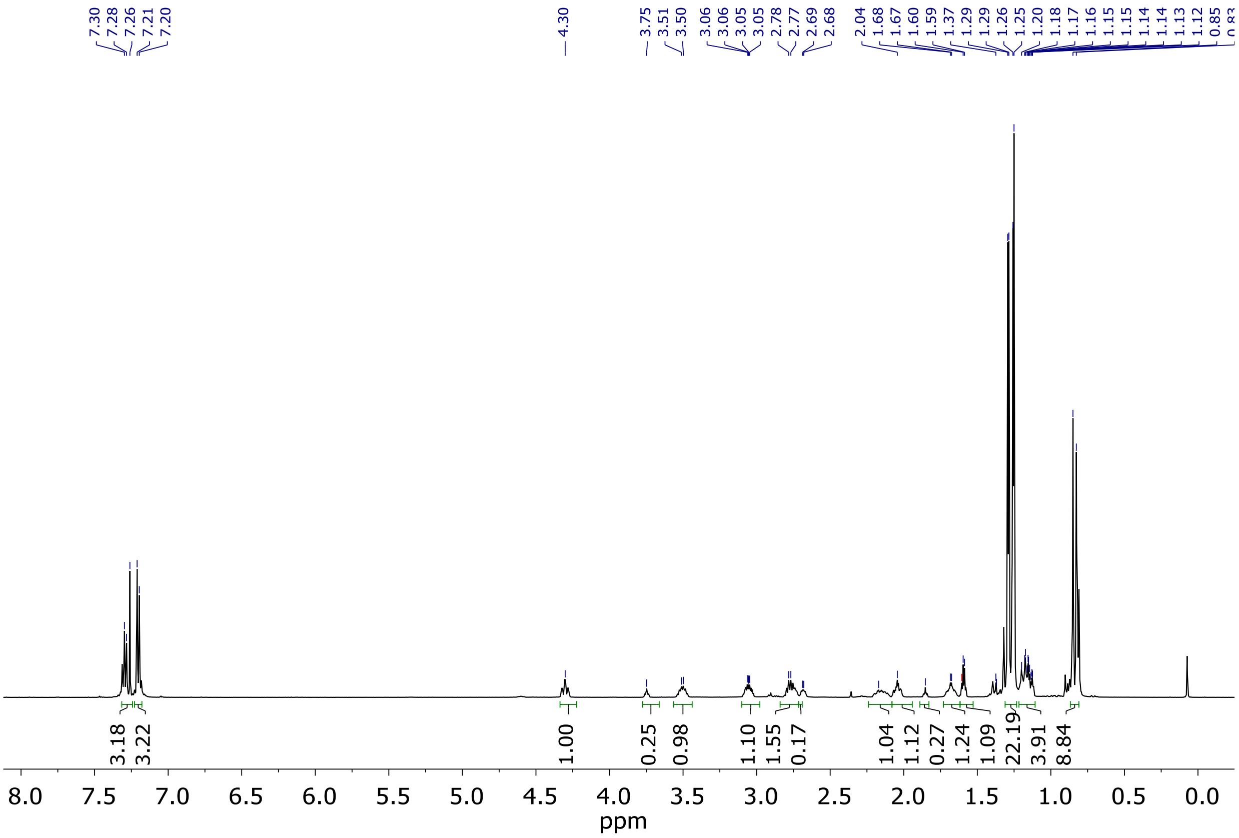

Figure S12a. ^{1}H NMR spectrum of **7**-*R*/*S* (500 MHz, 298 K, $CDCl_3$).

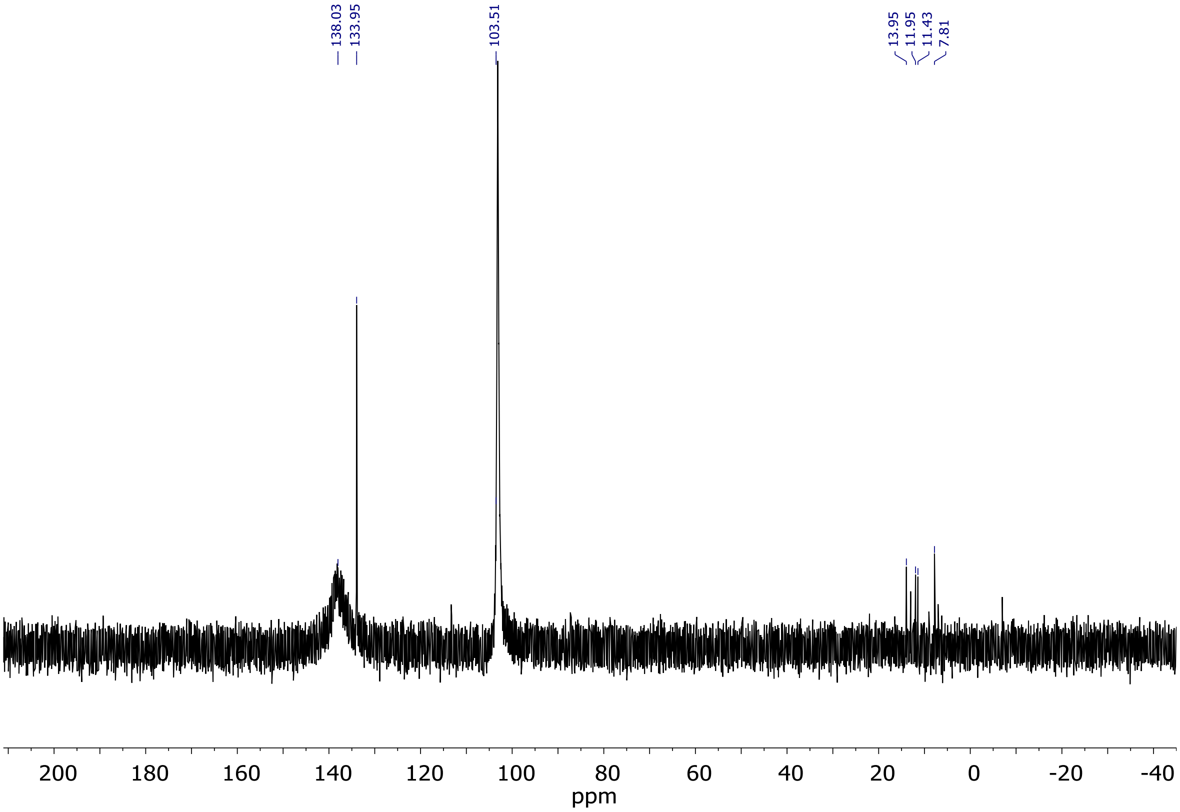

Figure S12b. $^{31}P\{^{1}H\}$ NMR spectrum of **7**-*R*/*S* (202 MHz, 298 K, $CDCl_3$).

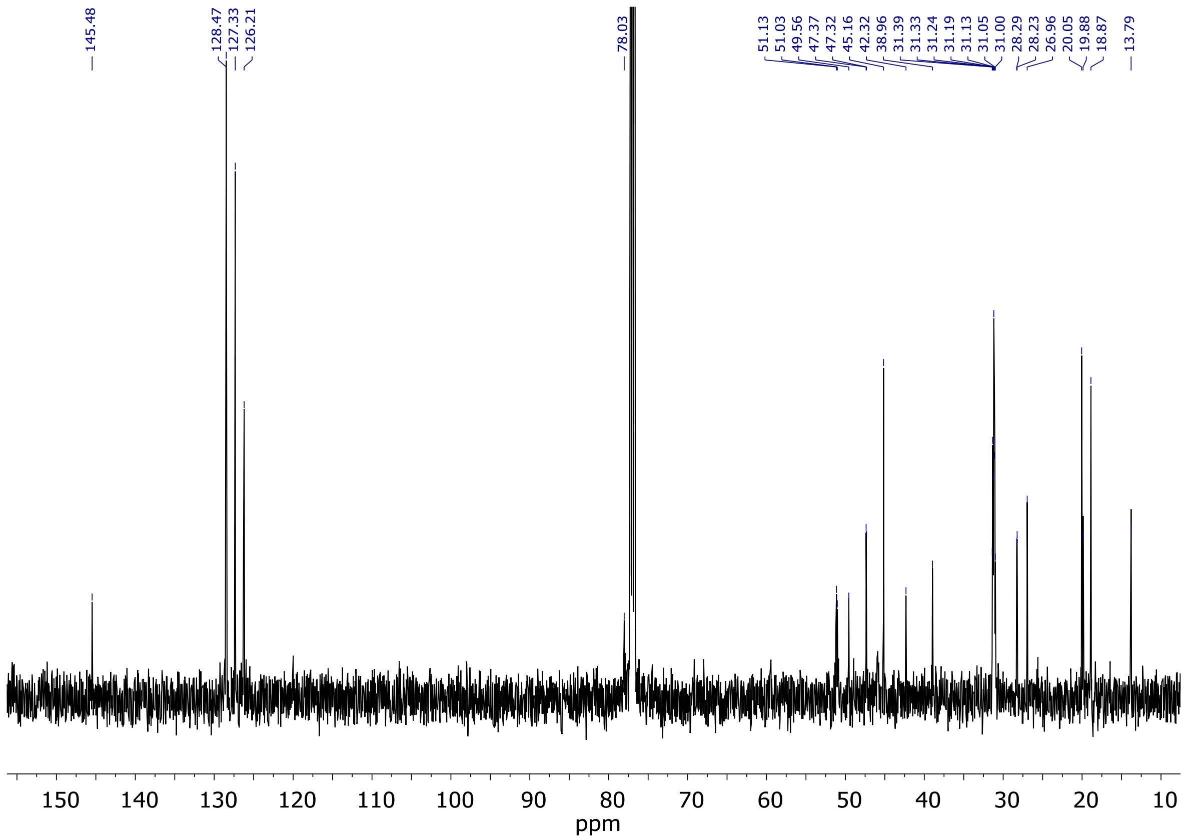

Figure S12c. $^{13}C\{^1H\}$ NMR spectrum of **7**-*R*/*S* (126 MHz, 298 K, $CDCl_3$).

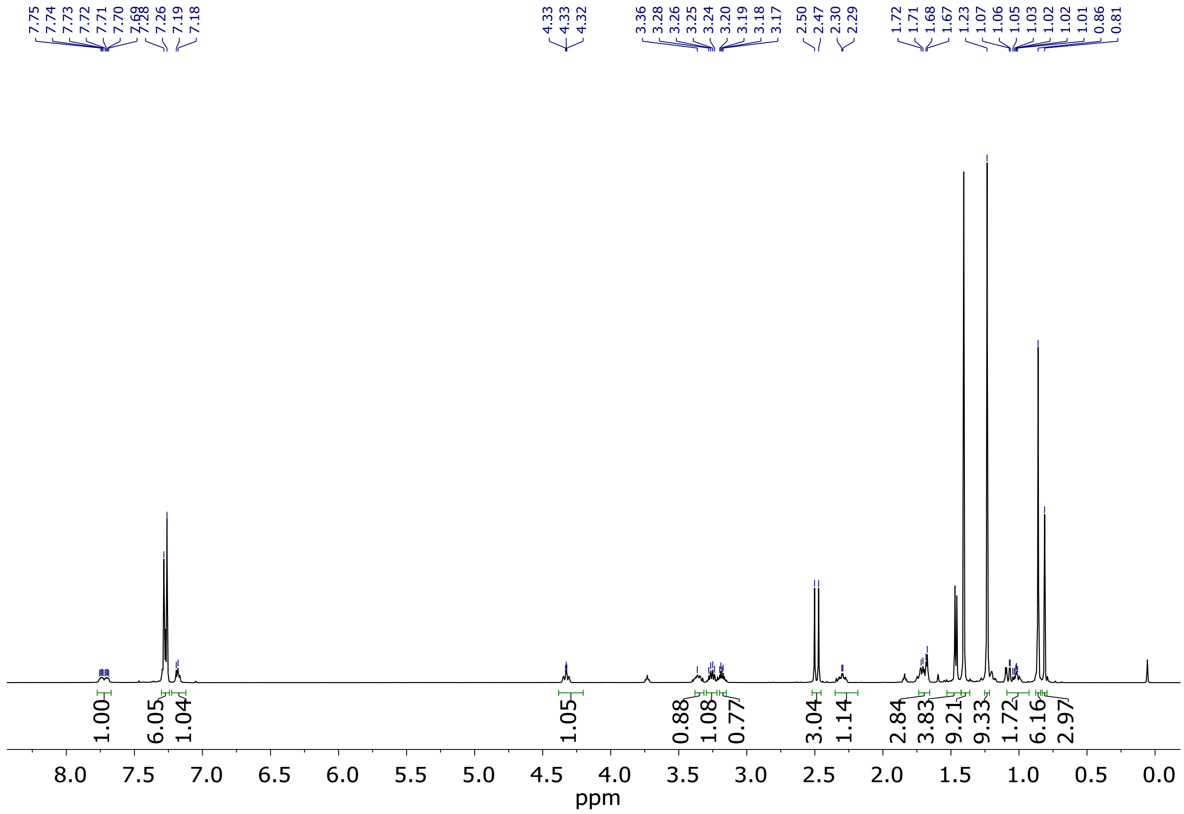

Figure S13a. ^1H NMR spectrum of **8**-*R* (500 MHz, 298 K, $CDCl_3$).

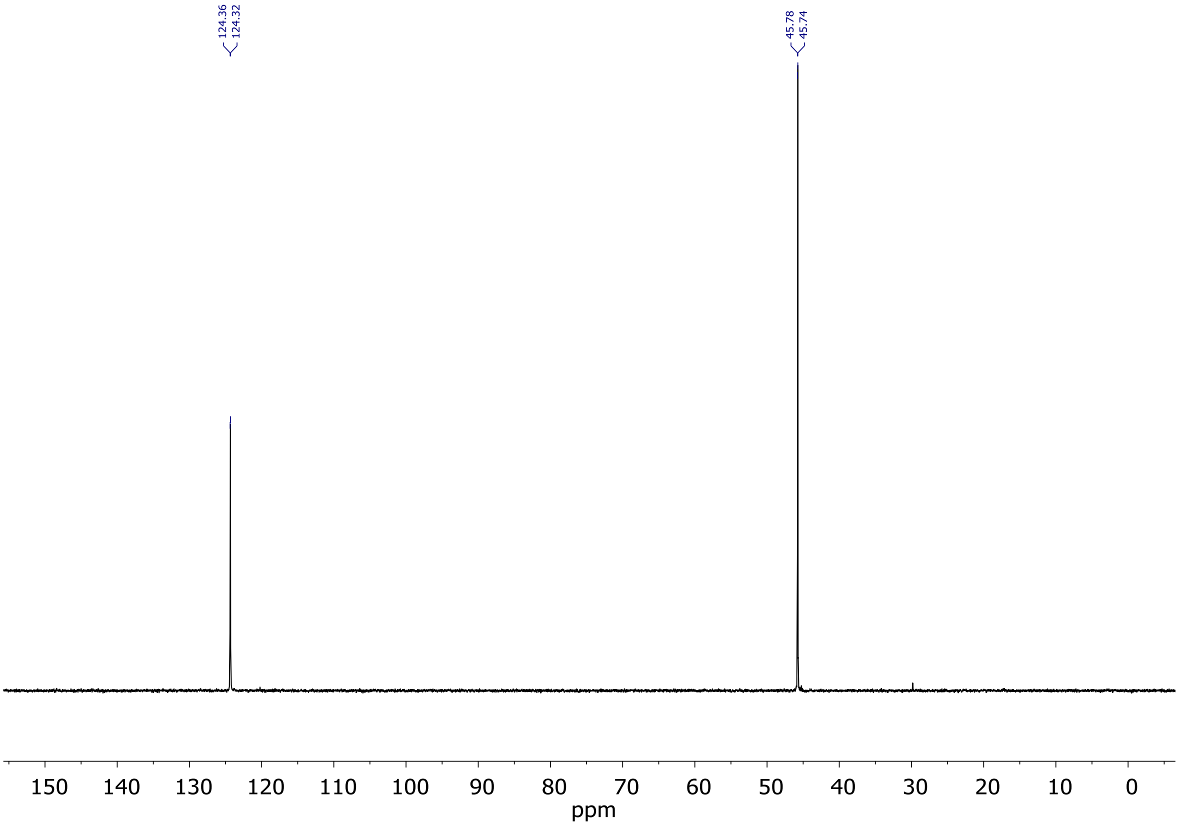

Figure S13b. $^{31}P\{^{1}H\}$ NMR spectrum of **8**-*R* (202 MHz, 298 K, $CDCl_3$).

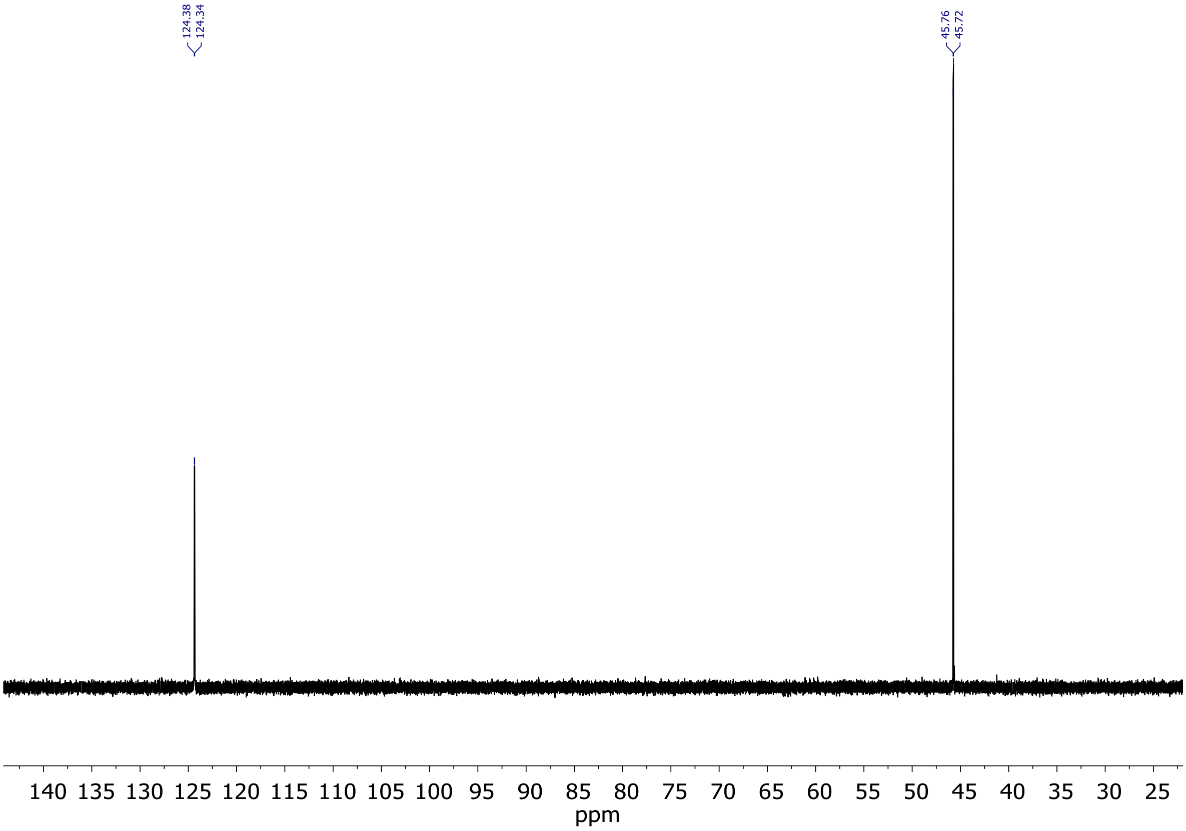

Figure S13c. $^{31}P\{^{1}H\}$ NMR spectrum of **8**-*R* (202 MHz, 298 K, $CDCl_3$; pulse program = zgig30, dummy scans = 0, recycle, delay = 30 s).

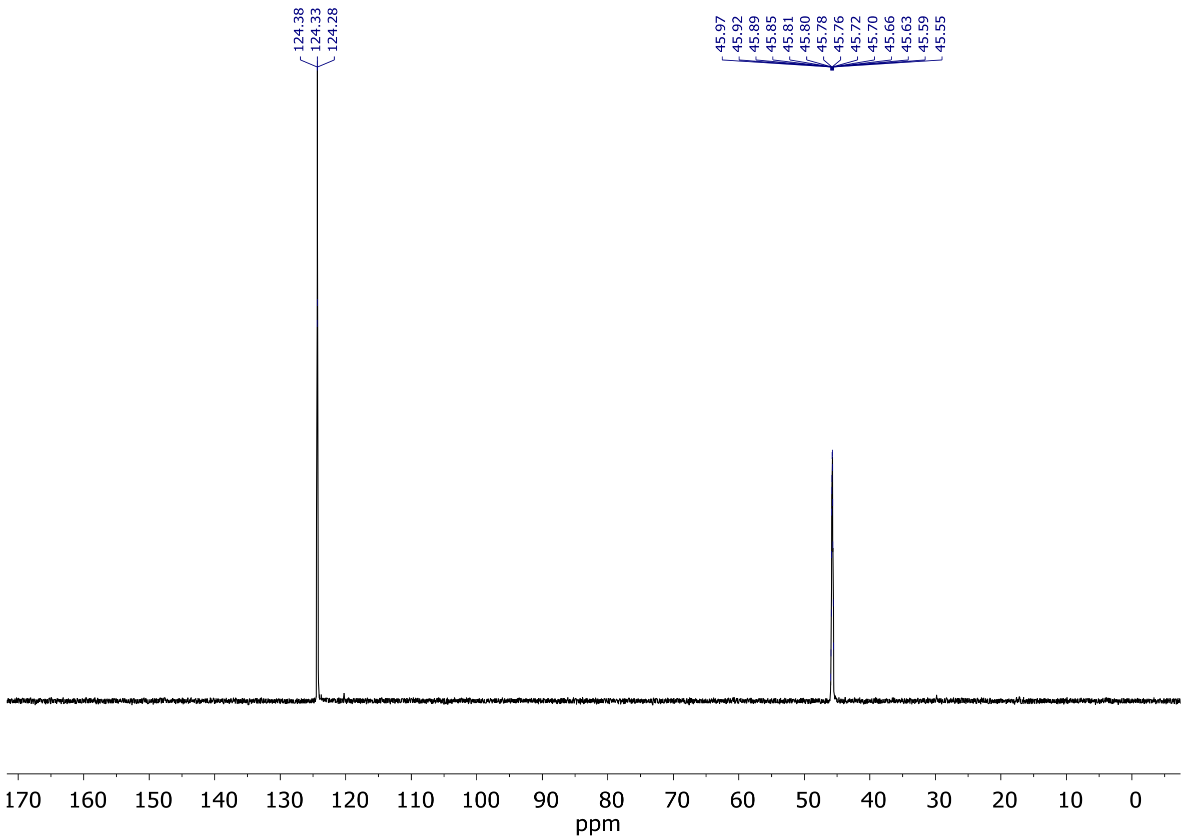

Figure S13d. ^{31}P NMR spectrum of **8**-*R* (202 MHz, 298 K, $CDCl_3$).

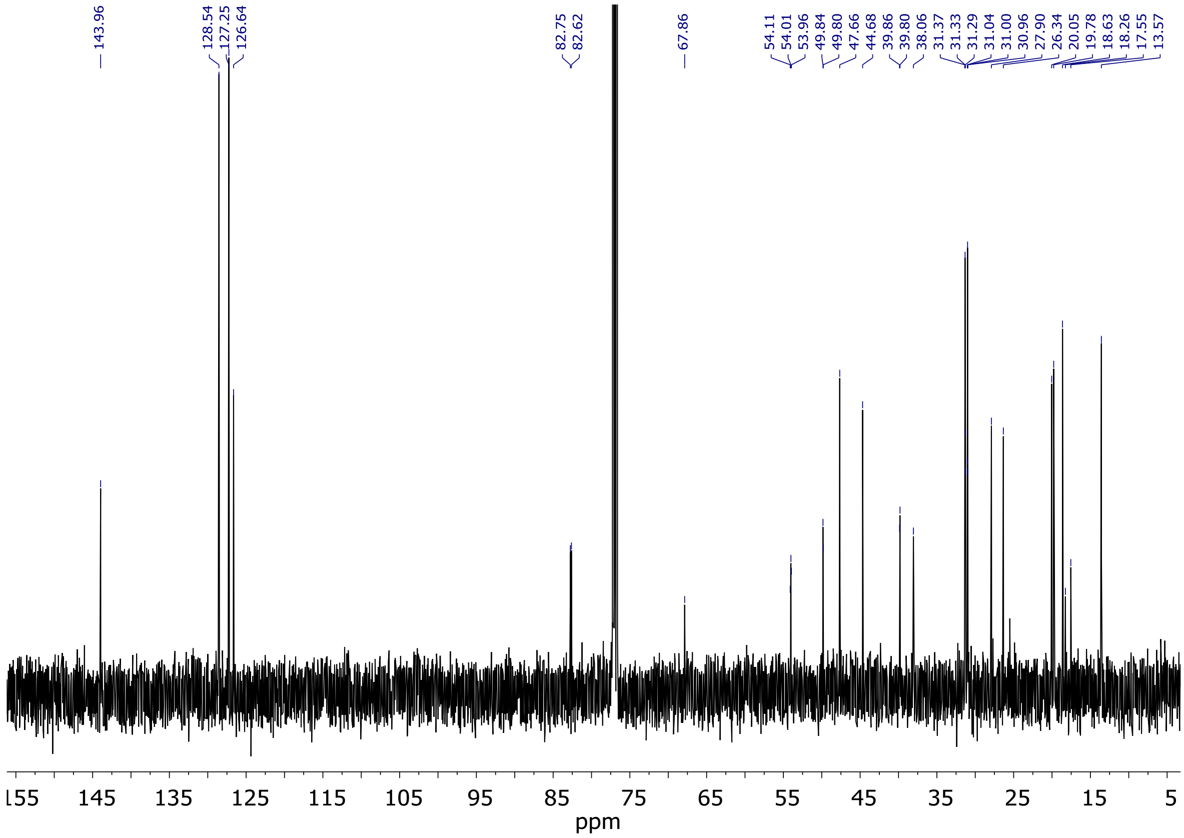

Figure S13e. $^{13}C\{^1H\}$ NMR spectrum of **8**-*R* (126 MHz, 298 K, $CDCl_3$).

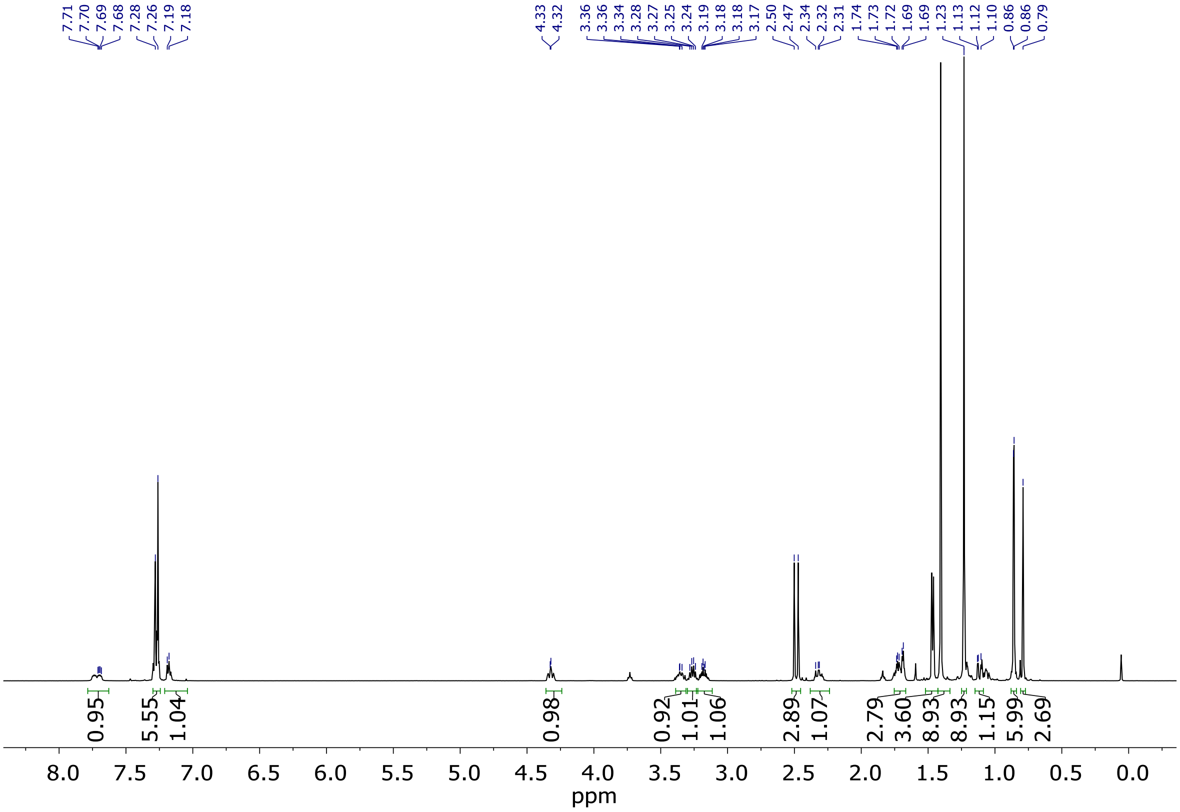

Figure S14a. ^{1}H NMR spectrum of **8**-*S* (500 MHz, 298 K, $CDCl_3$).

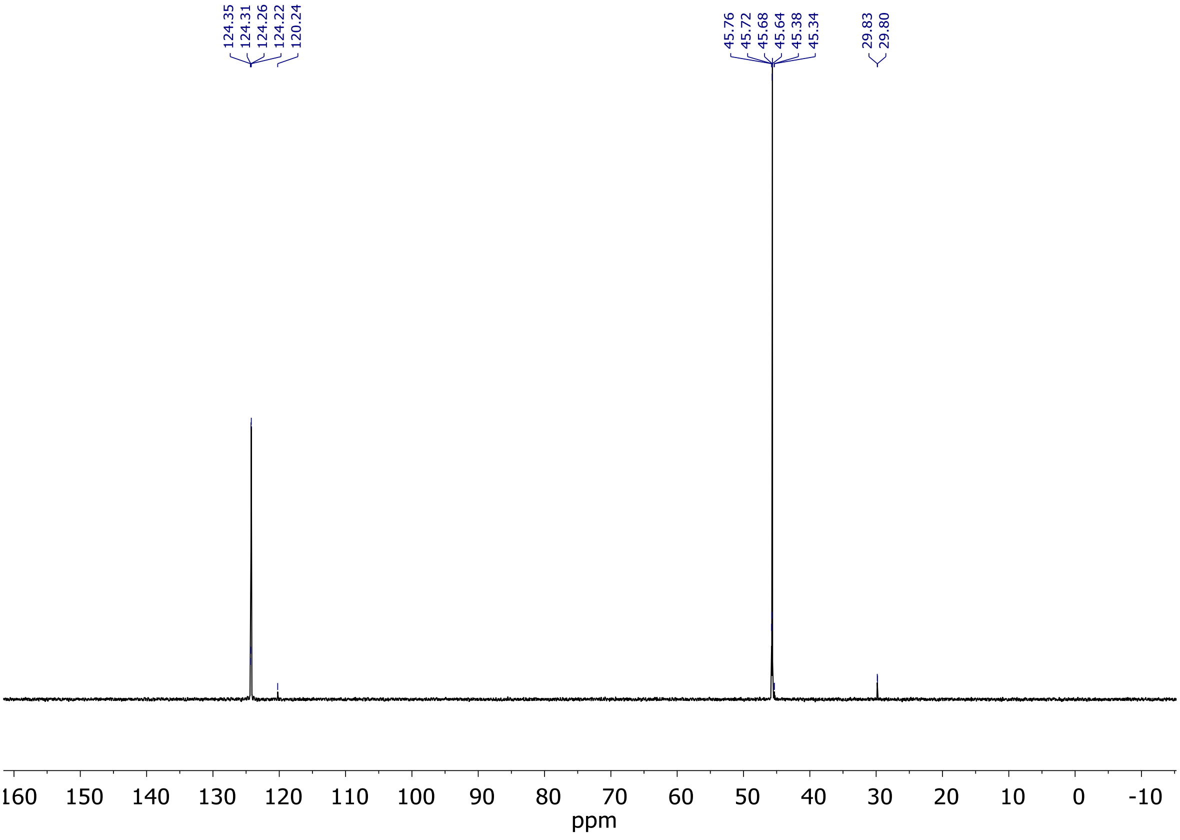

Figure S14b. $^{31}P\{^1H\}$ NMR spectrum of **8**-*S* (202 MHz, 298 K, $CDCl_3$).

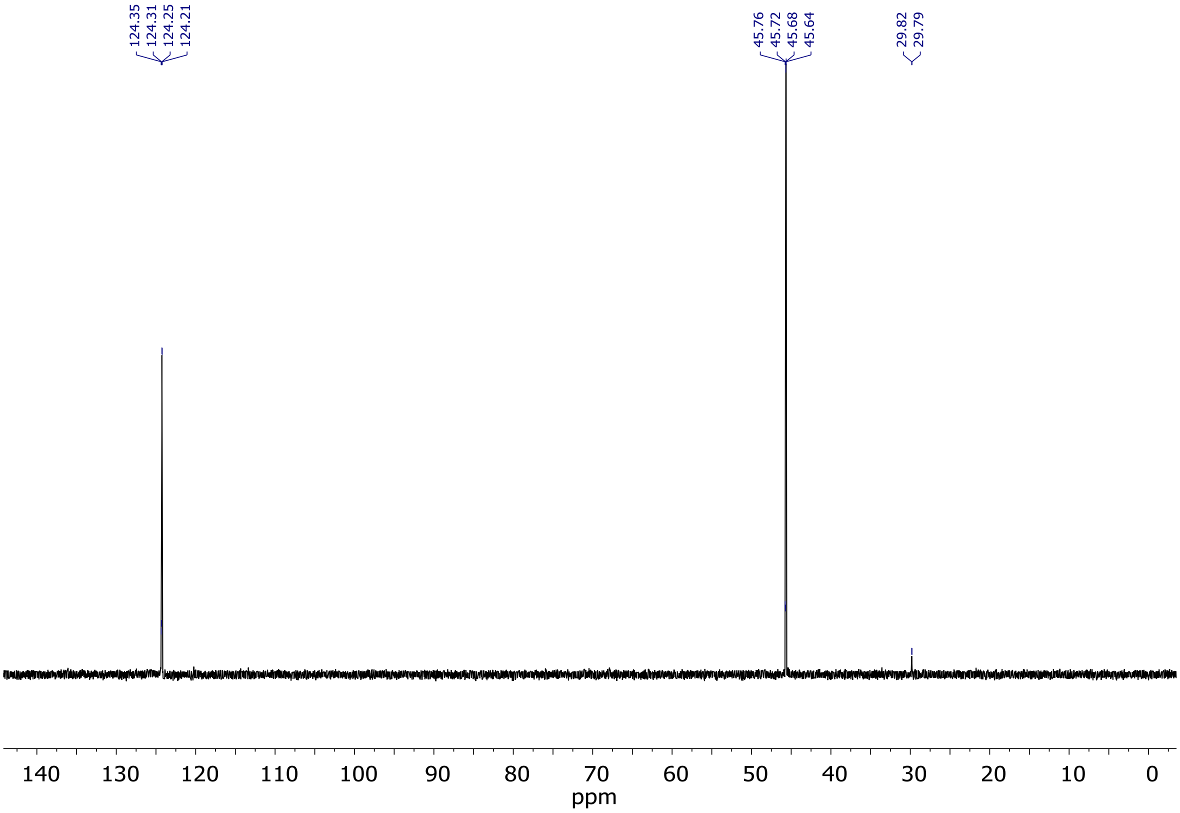

Figure S14c. $^{31}P\{^{1}H\}$ NMR spectrum of **8**-*S* (202 MHz, 298 K, $CDCl_3$; pulse program = zgig30, dummy scans = 0, recycle, delay = 30 s).

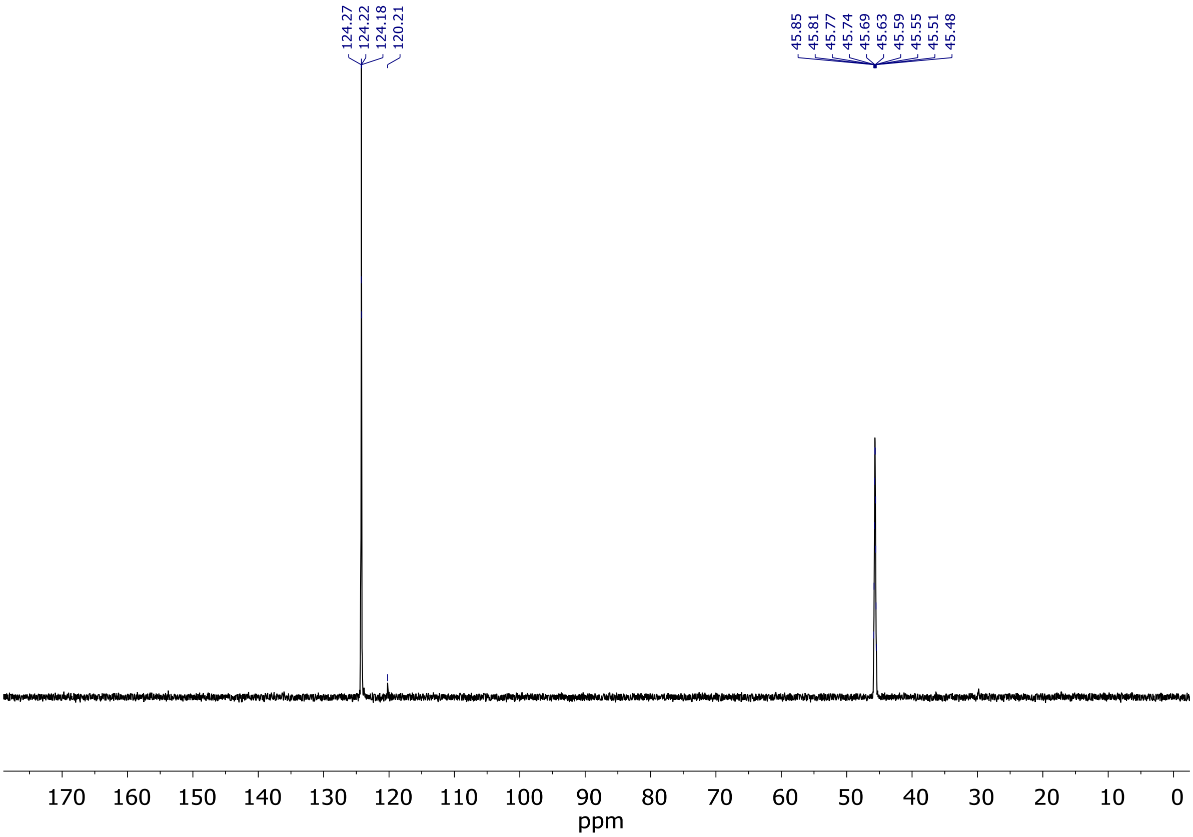

Figure S14d. ^{31}P NMR spectrum of **8**-*S* (202 MHz, 298 K, $CDCl_3$).

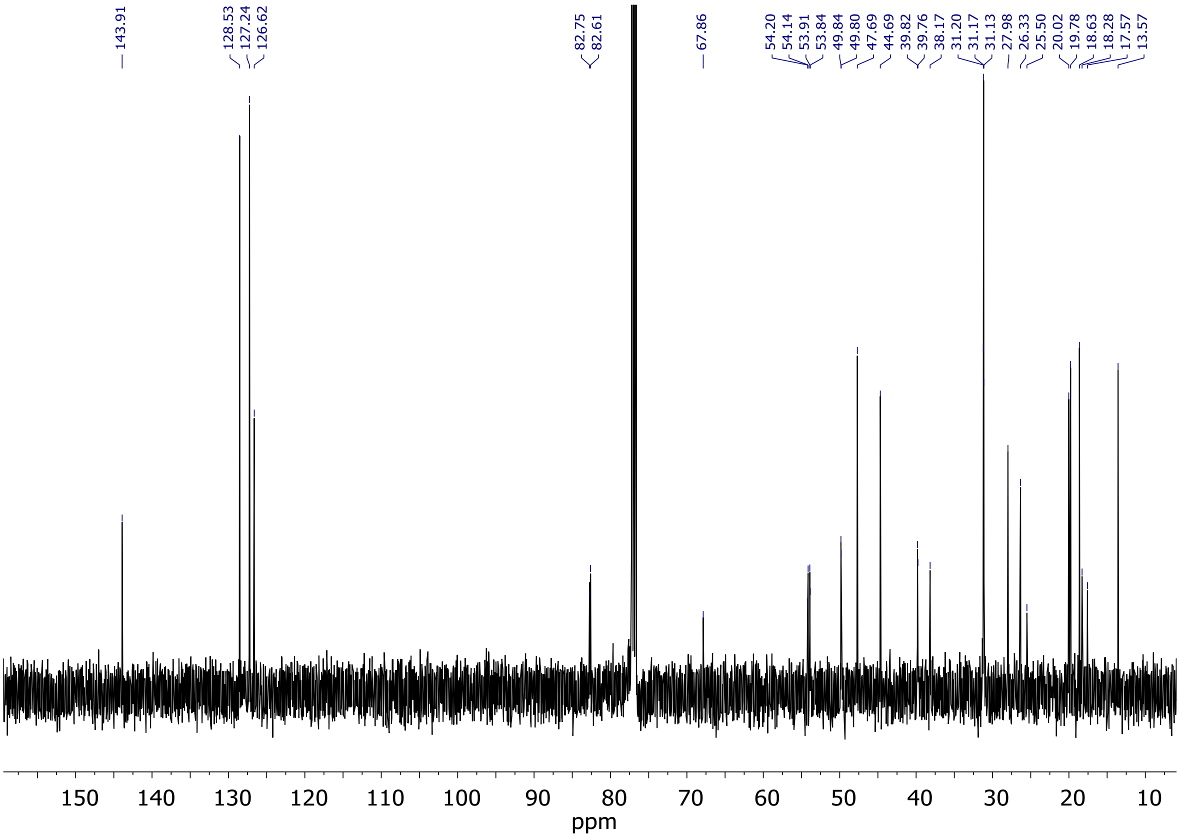

Figure S14e. $^{13}C\{^{1}H\}$ NMR spectrum of **8**-*S* (126 MHz, 298 K, $CDCl_3$).

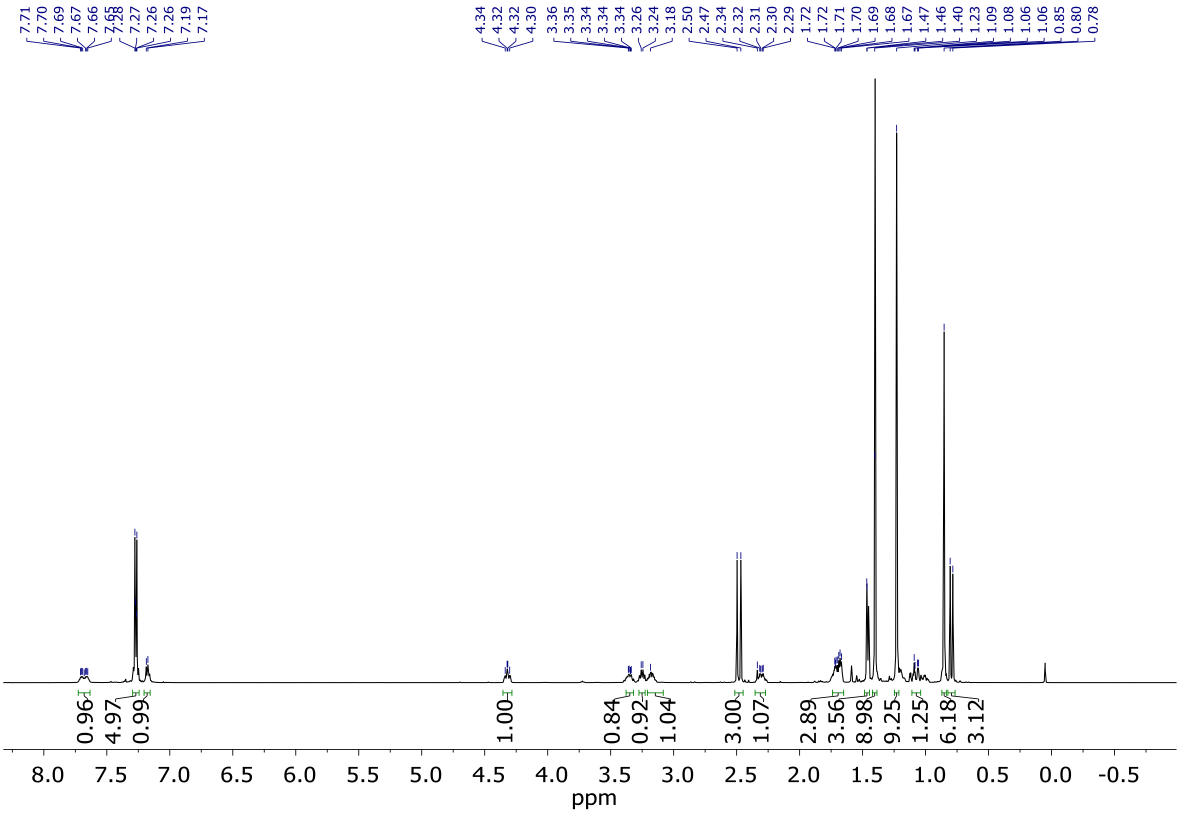

Figure S15a. ^{1}H NMR spectrum of **8**-*R*/*S* (500 MHz, 298 K, $CDCl_3$).

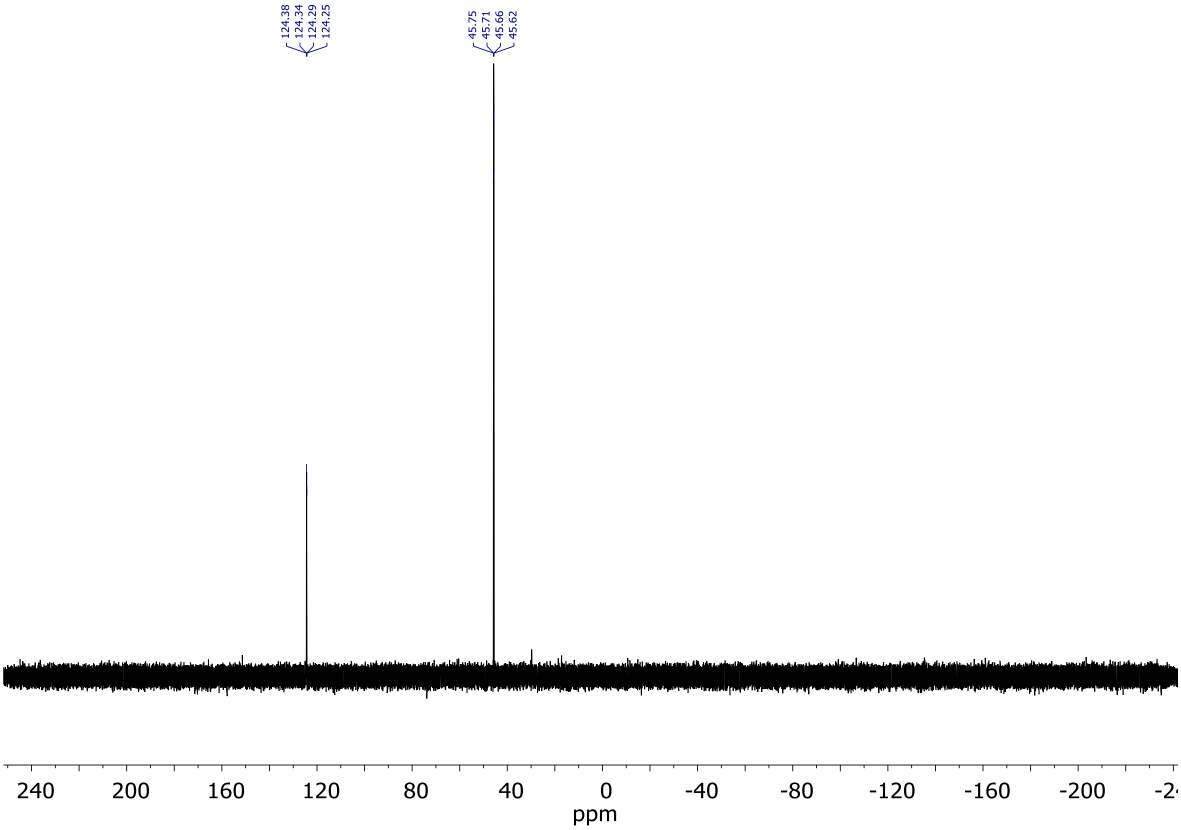

Figure S15b. $^{31}P\{^1H\}$ NMR spectrum of **8**-*R*/*S* (202 MHz, 298 K, $CDCl_3$).

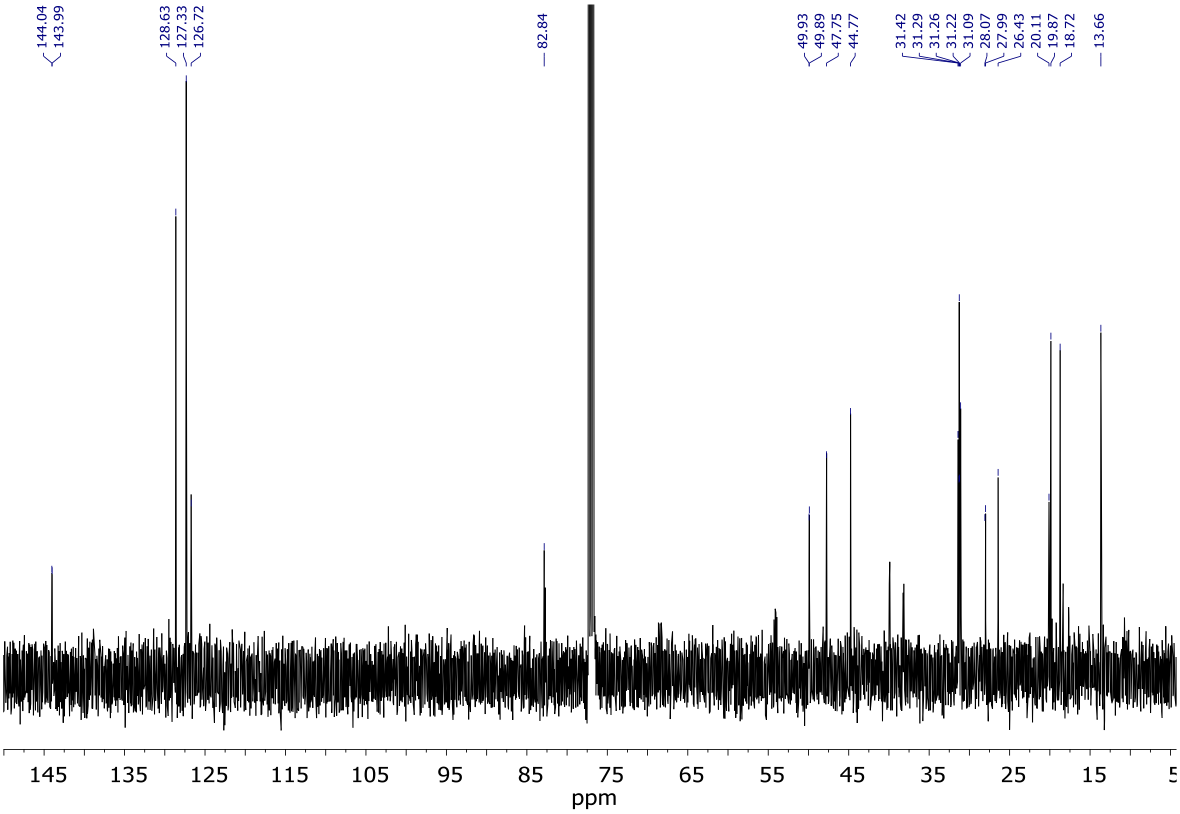

Figure S15c. $^{13}C\{^1H\}$ NMR spectrum of **8**-*R*/*S* (126 MHz, 298 K, $CDCl_3$).

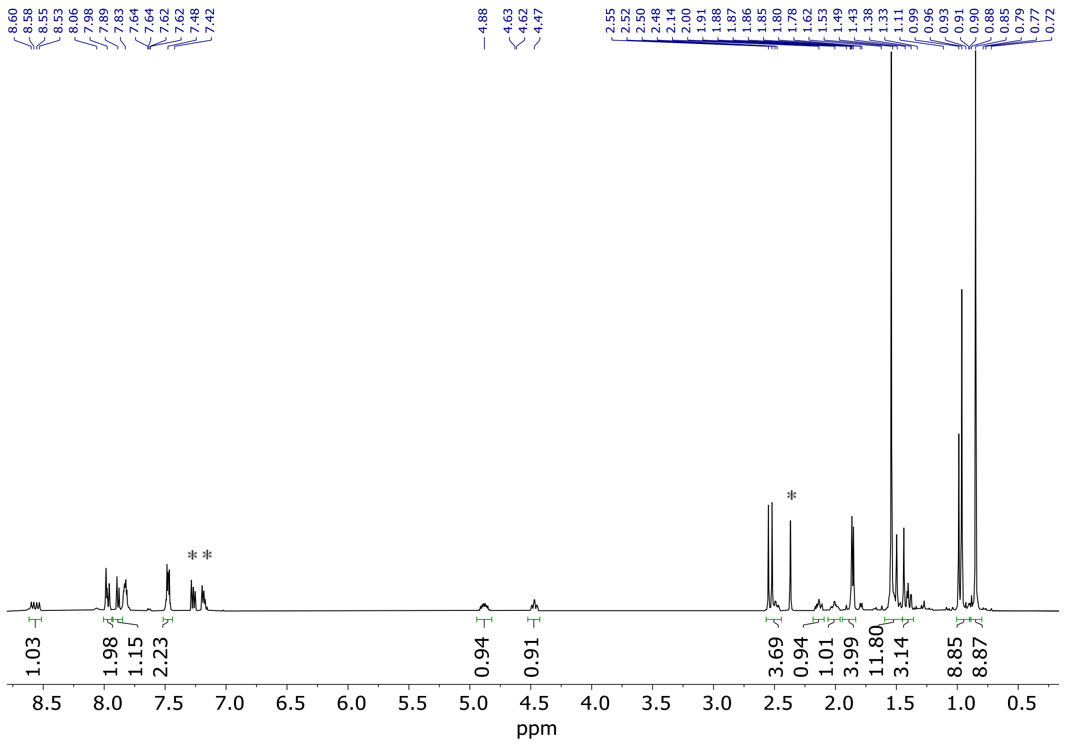

Figure S16a. ^{1}H NMR spectrum of **9**-*R* (500 MHz, 298 K, $CDCl_3$). Starred peaks are from remnant toluene (δ 7.25, 7.17 and 2.36 ppm).

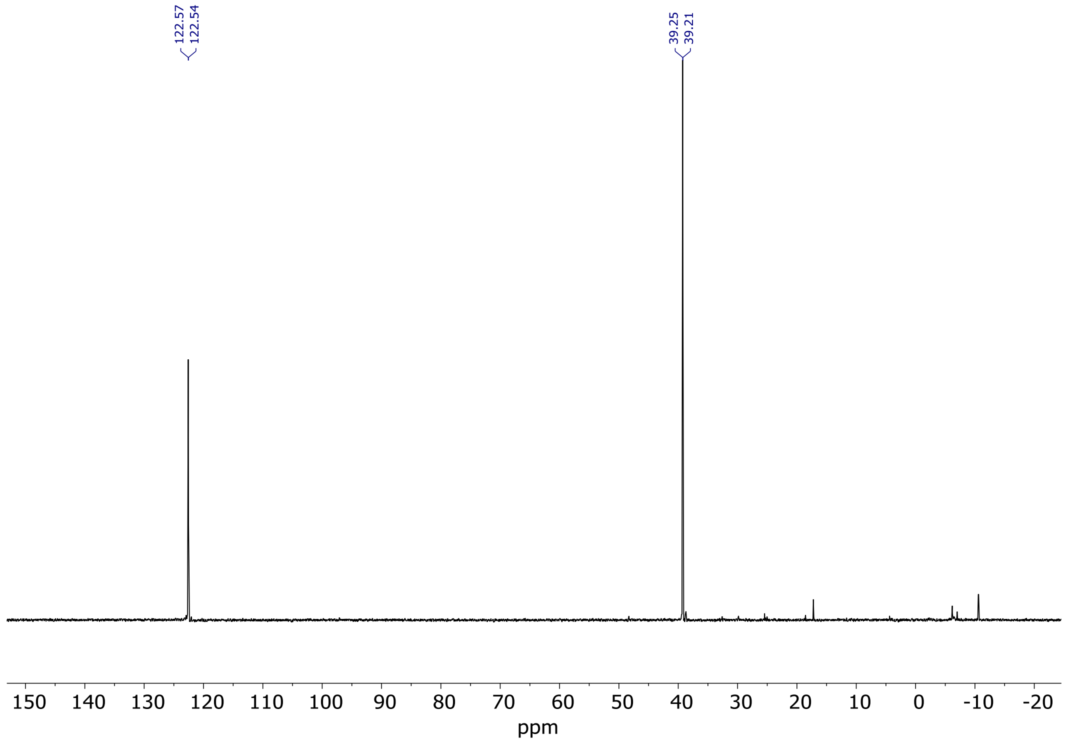

Figure S16b. $^{31}P\{^{1}H\}$ NMR spectrum of **9**-*R* (202 MHz, 298 K, $CDCl_3$).

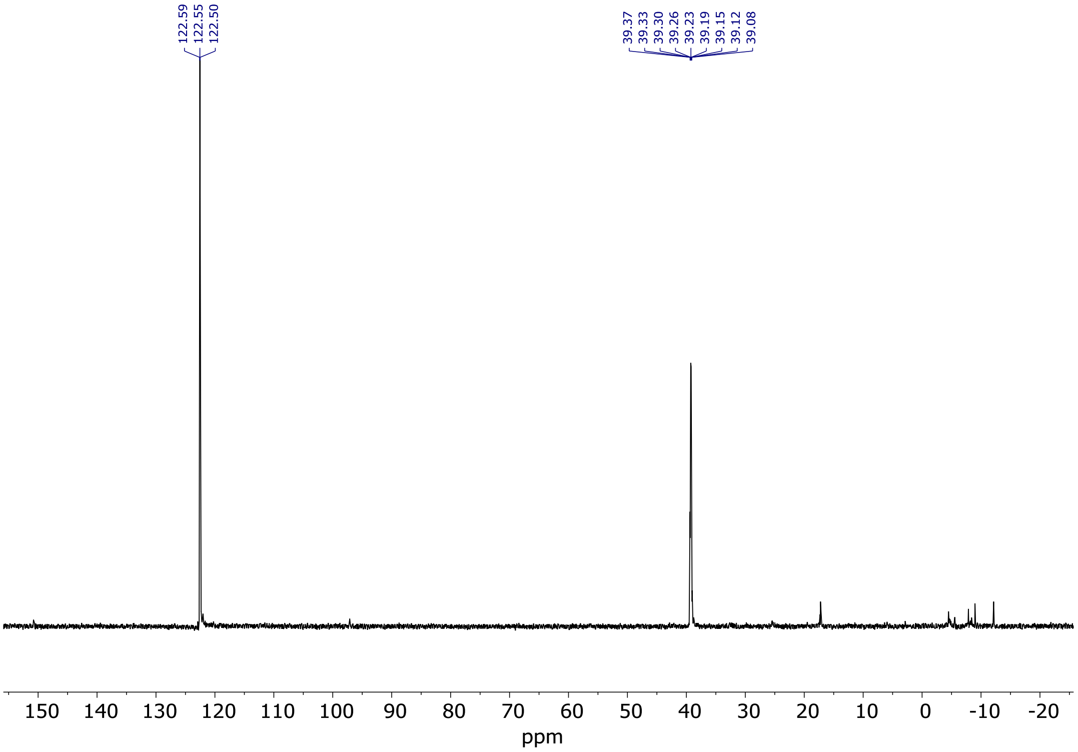

Figure S16c. ^{31}P NMR spectrum of **9**-*R* (202 MHz, 298 K, $CDCl_3$).

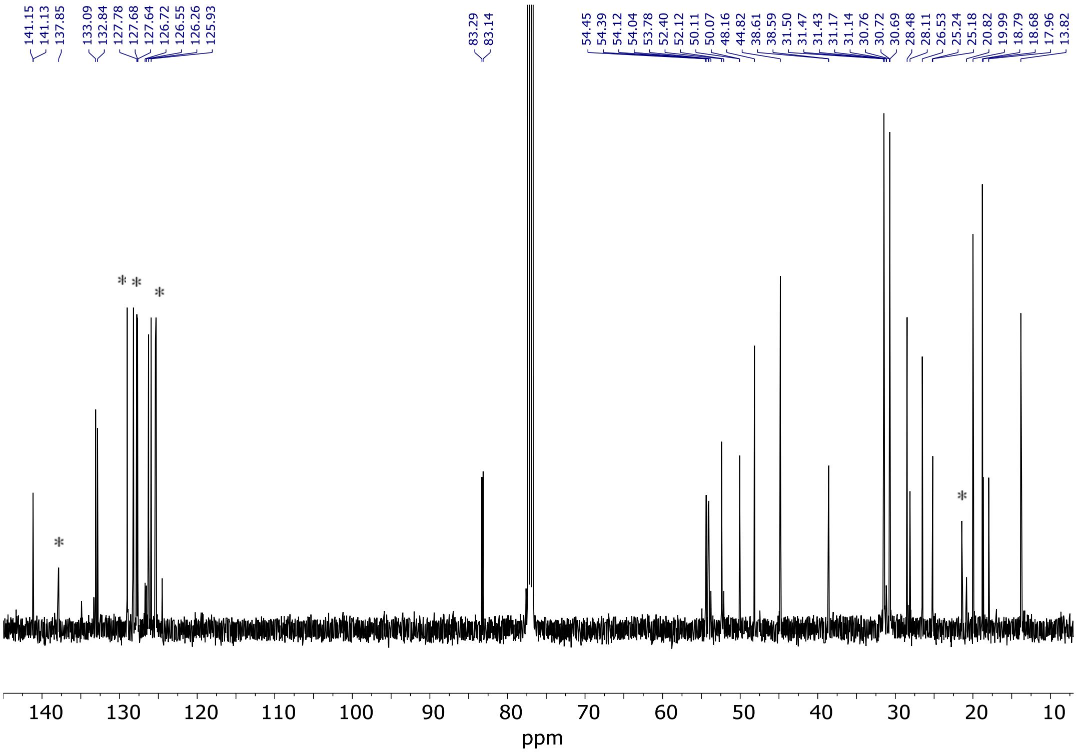

Figure S16d. ^{13}C{^{1}H} NMR spectrum of **9**-*R* (126 MHz, 298 K, $CDCl_3$). Starred peaks are from remnant toluene (δ 137.9, 129.1, 128.3, 125.3 and 21.5 ppm).

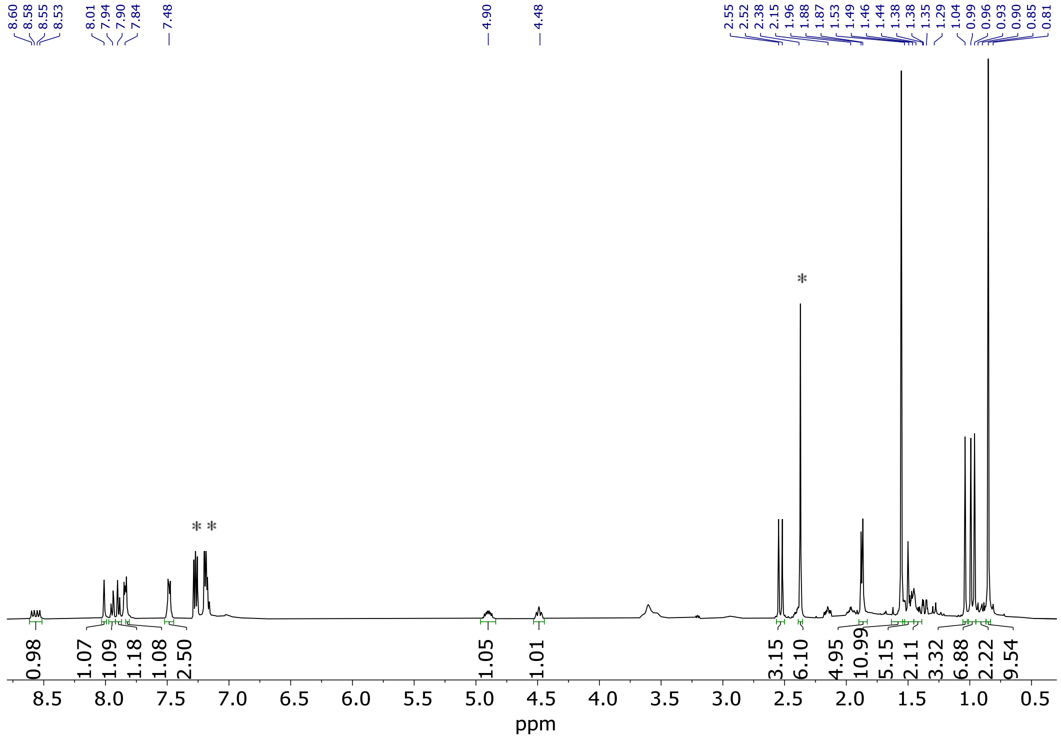

Figure S17a. ^{1}H NMR spectrum of **9**-*S* (500 MHz, 298 K, $CDCl_3$). Starred peaks are from remnant toluene (δ 7.25, 7.17 and 2.36 ppm).

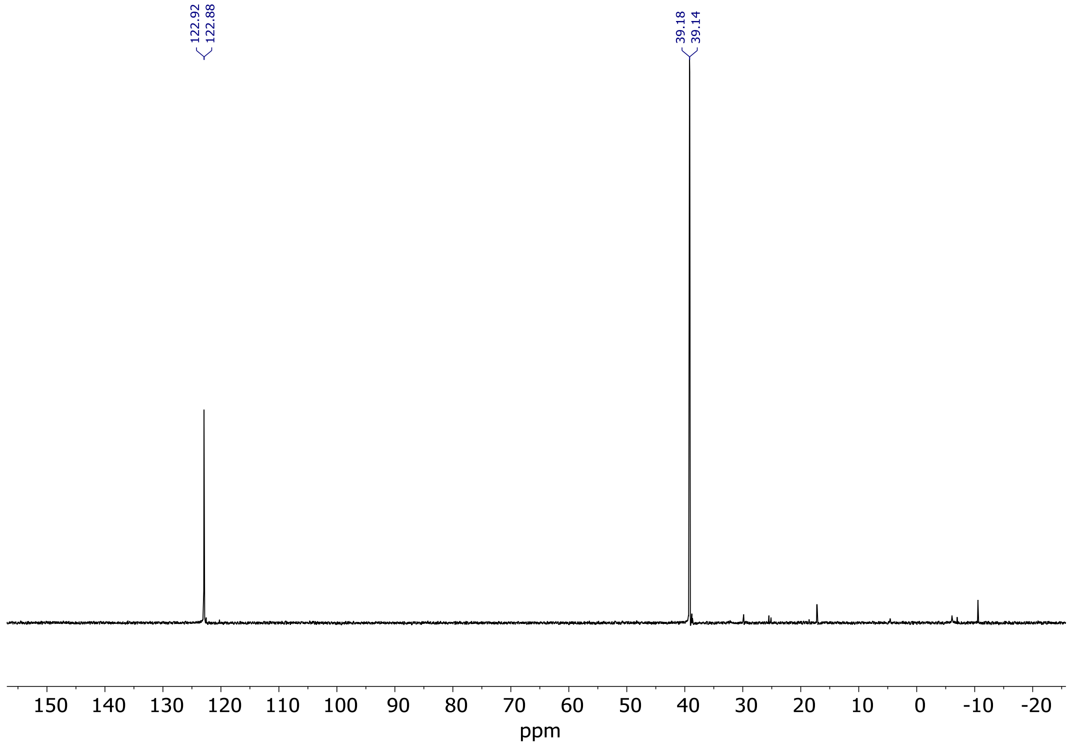

Figure S17b. $^{31}P\{^{1}H\}$ NMR spectrum of **9**-*S* (202 MHz, 298 K, $CDCl_3$).

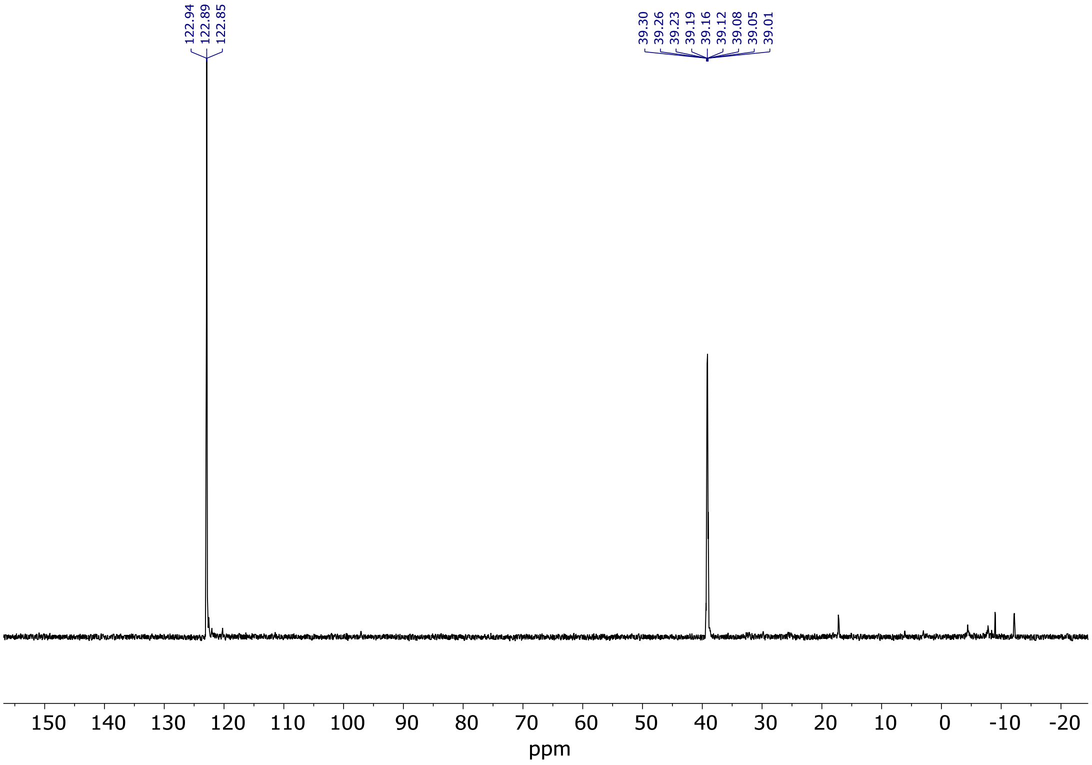

Figure S17c. ^{31}P NMR spectrum of **9**-*S* (202 MHz, 298 K, $CDCl_3$).

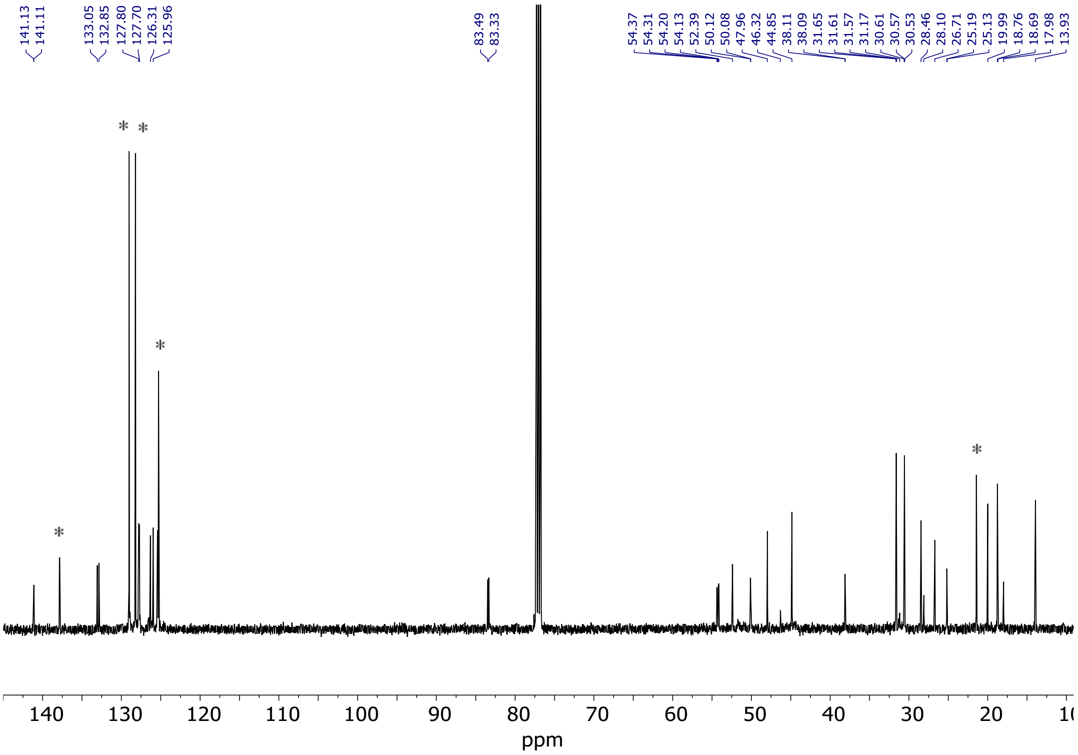

Figure S17d. $^{13}C\{^1H\}$ NMR spectrum of **9**-*S* (126 MHz, 298 K, $CDCl_3$). Starred peaks are from remnant toluene (*δ* 137.9, 129.1, 128.3, 125.3 and 21.5 ppm).

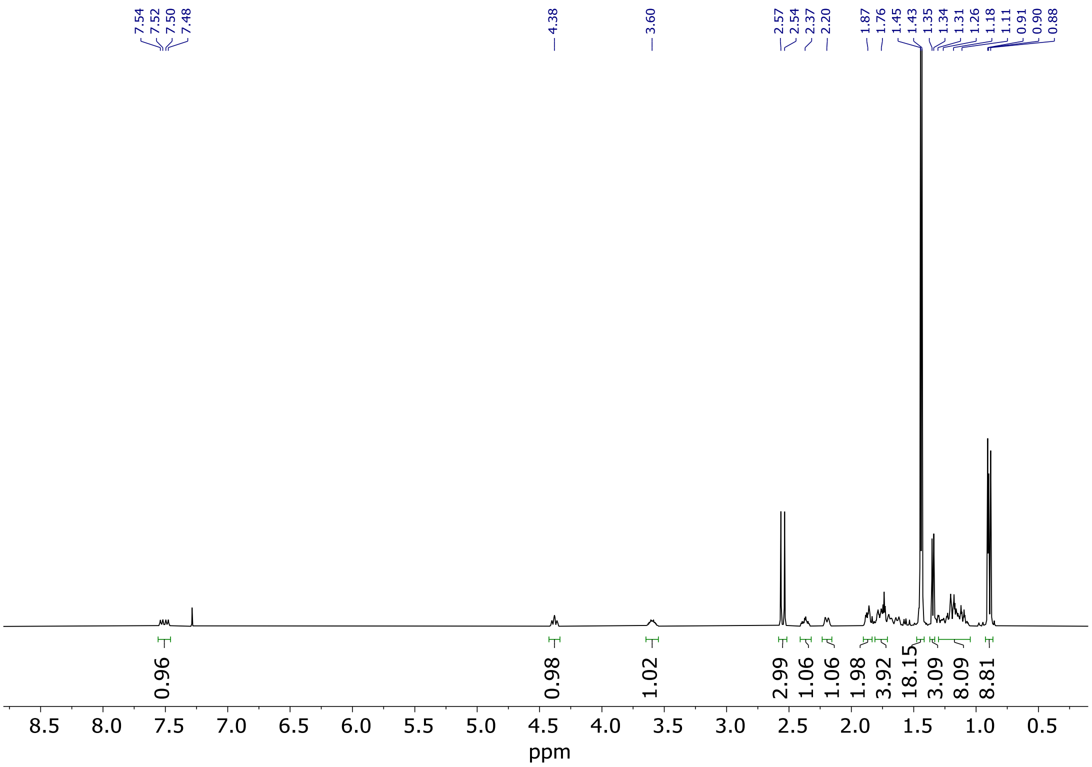

Figure S18a. ^{1}H NMR spectrum of **10**-*R* (500 MHz, 298 K, $CDCl_3$).

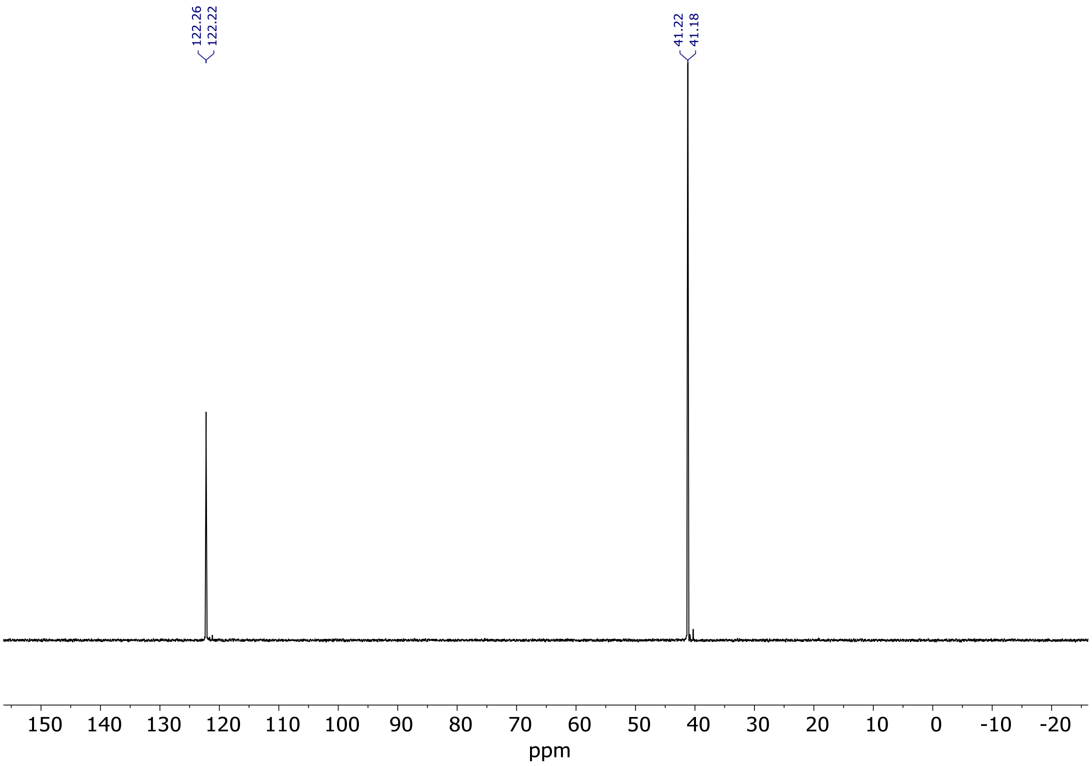

Figure S18b. $^{31}P\{^{1}H\}$ NMR spectrum of **10**-*R* (202 MHz, 298 K, $CDCl_3$).

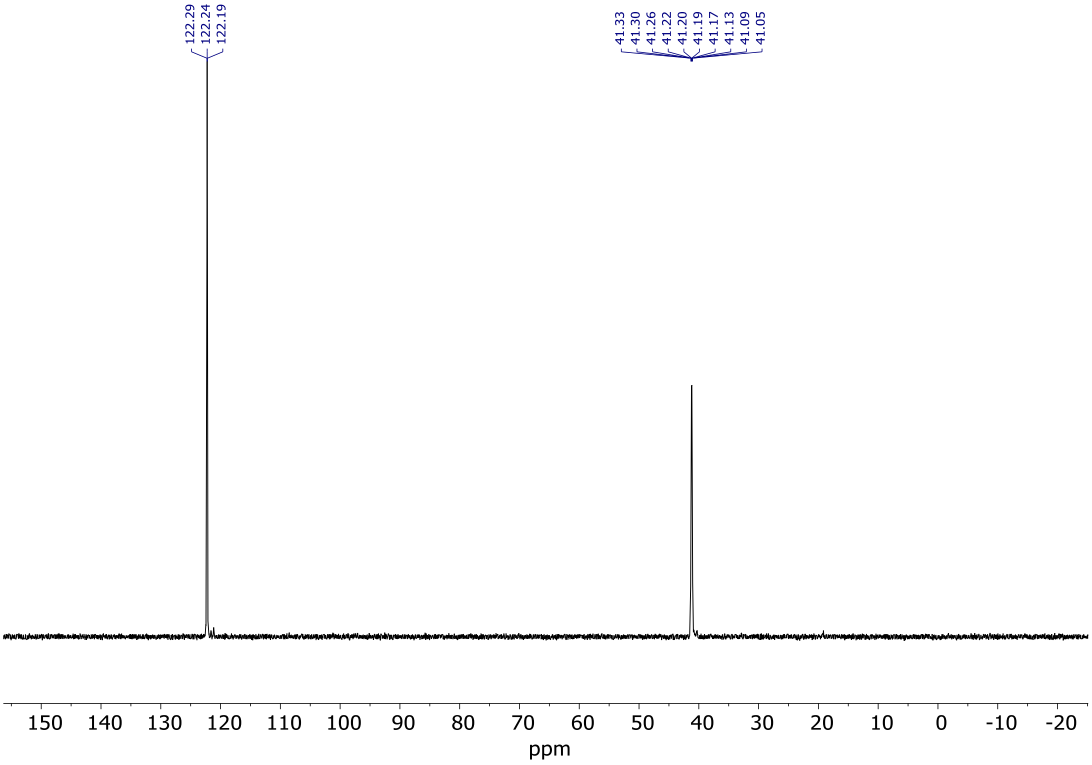

Figure S18c. ^{31}P NMR spectrum of **10**-*R* (202 MHz, 298 K, $CDCl_3$).

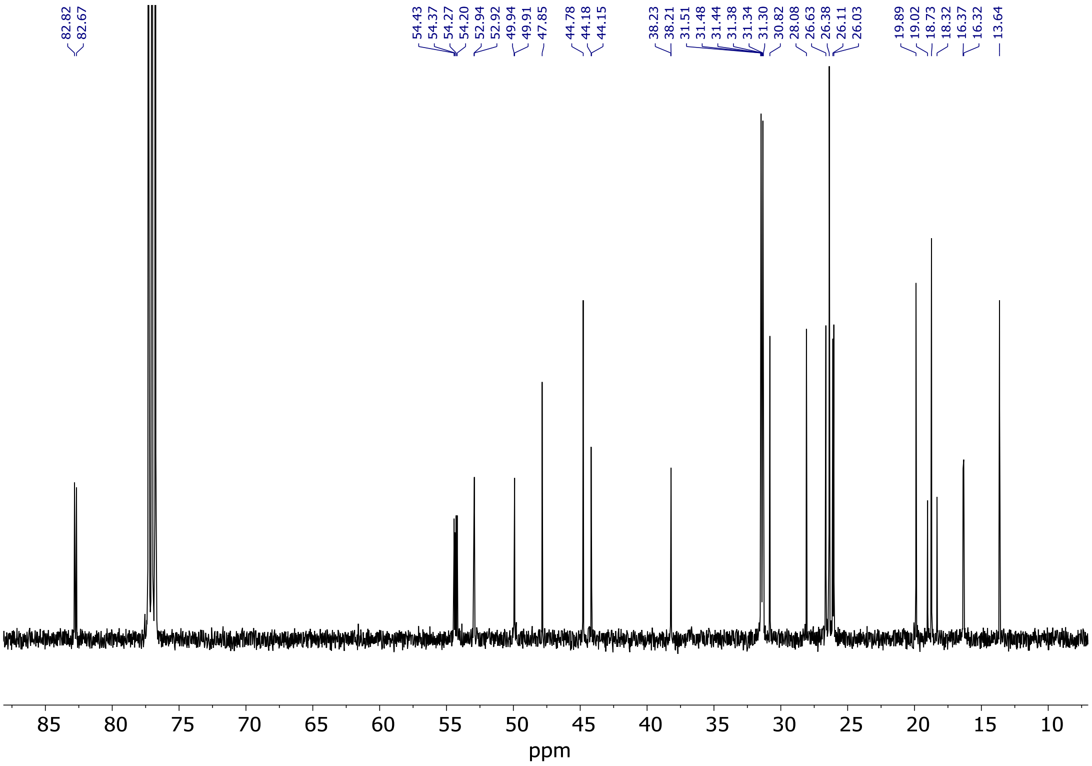

Figure S18d. $^{13}C\{^{1}H\}$ NMR spectrum of **10**-*R* (126 MHz, 298 K, $CDCl_3$).

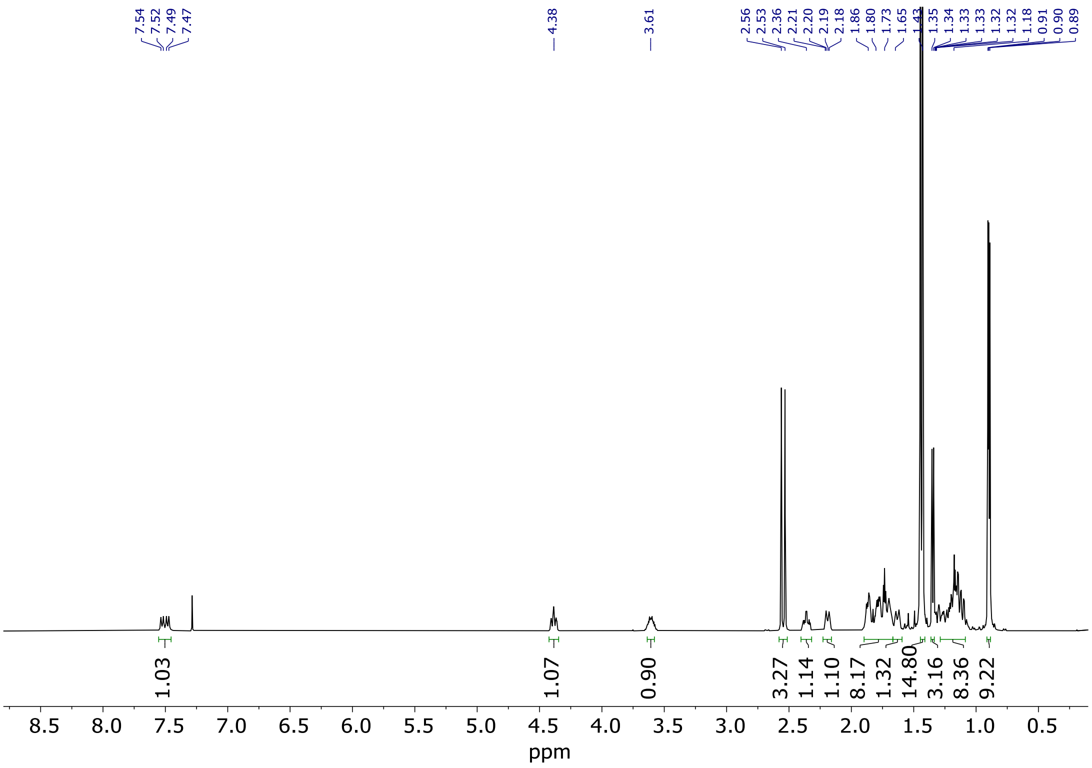

Figure S19a. ^{1}H NMR spectrum of **10**-*S* (500 MHz, 298 K, $CDCl_3$).

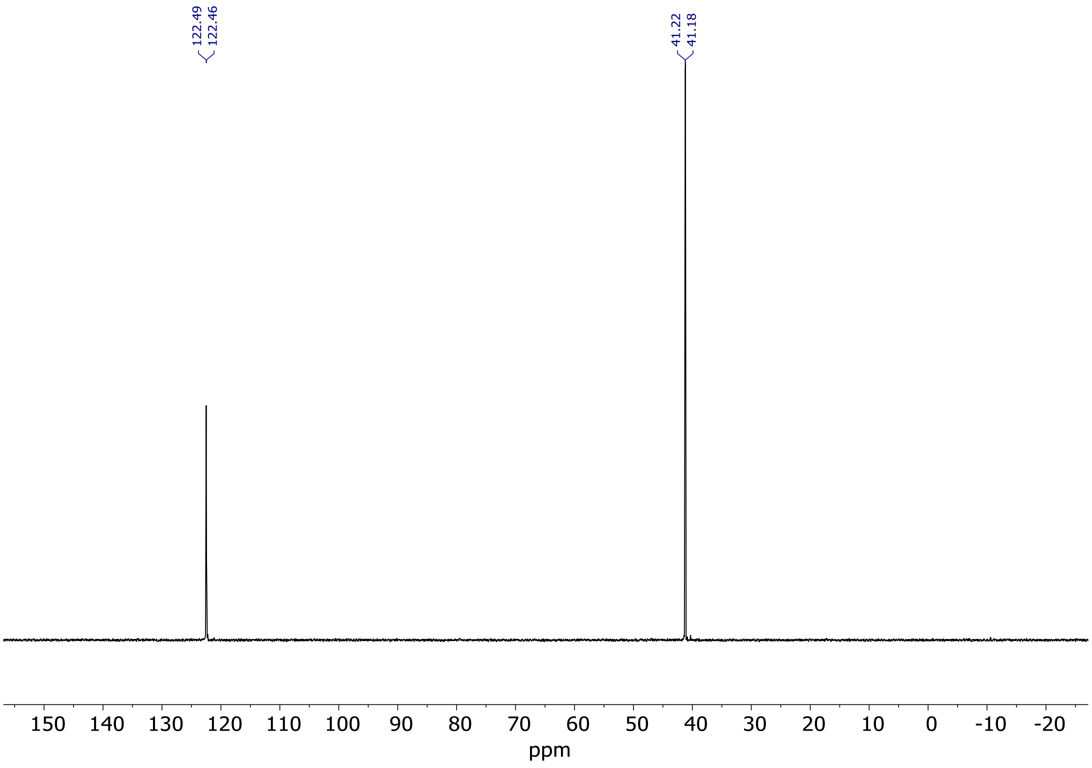

Figure S19b. $^{31}P\{^{1}H\}$ NMR spectrum of **10**-*S* (202 MHz, 298 K, $CDCl_3$).

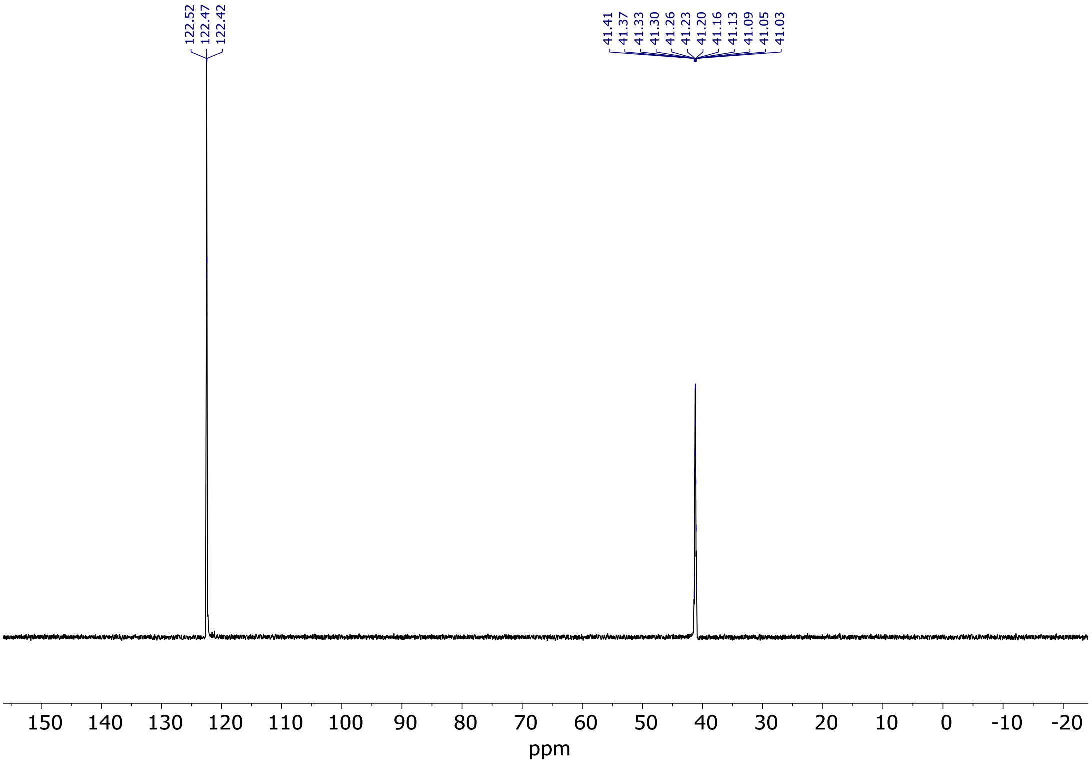

Figure S19c. ^{31}P NMR spectrum of **10**-*S* (202 MHz, 298 K, $CDCl_3$).

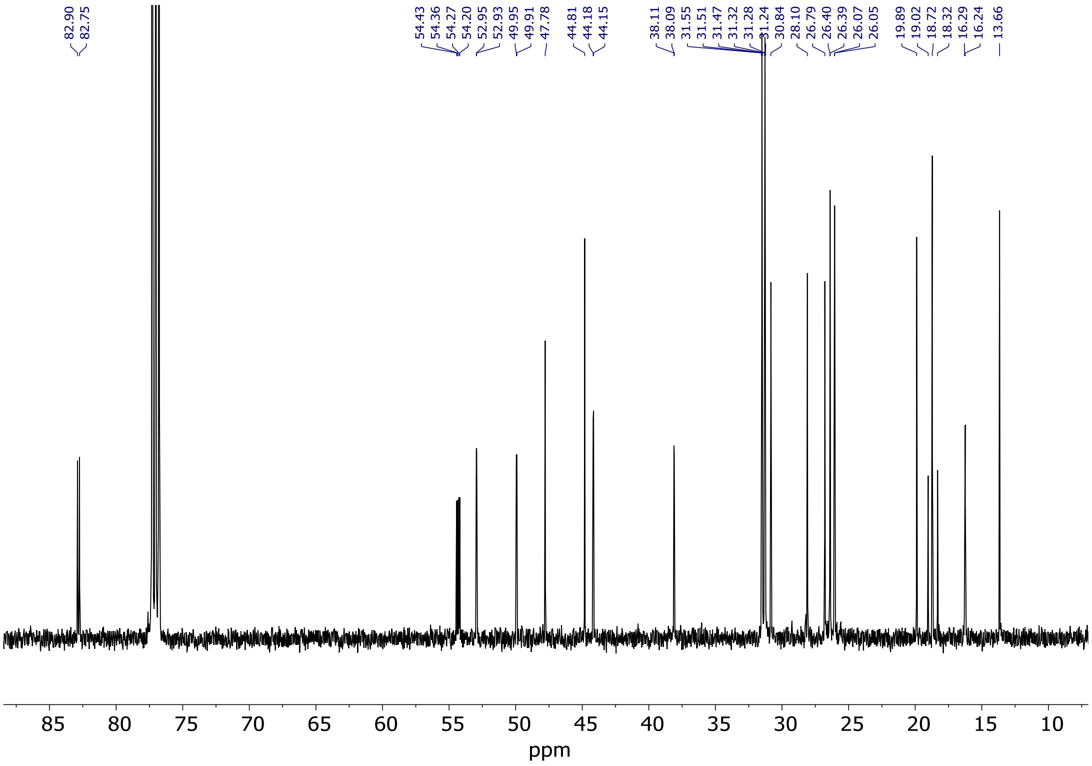

Figure S19d. $^{13}C\{^1H\}$ NMR spectrum of **10**-*S* (126 MHz, 298 K, $CDCl_3$).

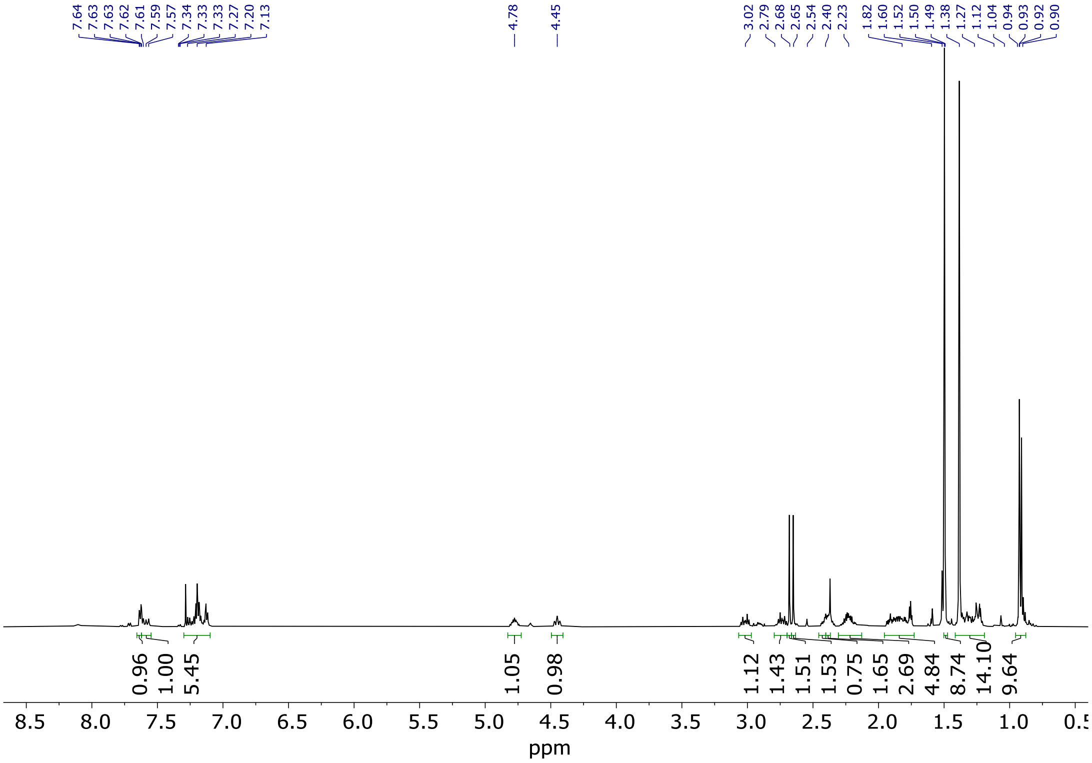

Figure S20a. ^{1}H NMR spectrum of **11**-*R* (500 MHz, 298 K, $CDCl_3$).

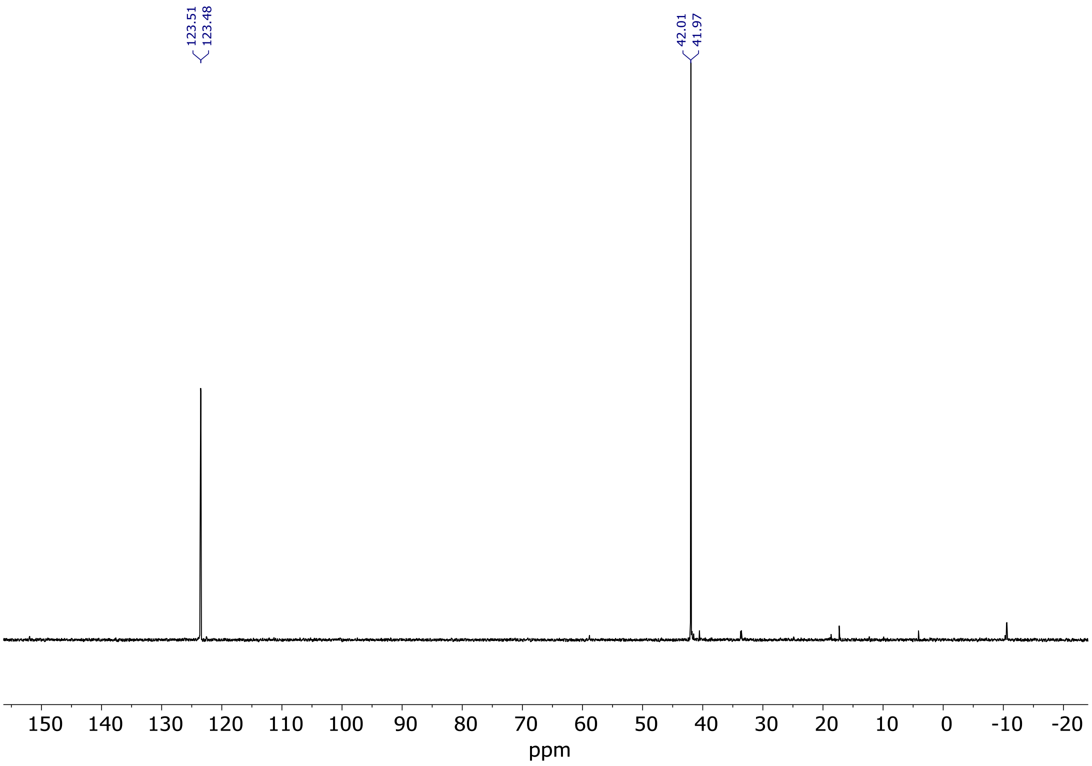

Figure S20b. $^{31}P\{^{1}H\}$ NMR spectrum of **11**-*R* (202 MHz, 298 K, $CDCl_3$).

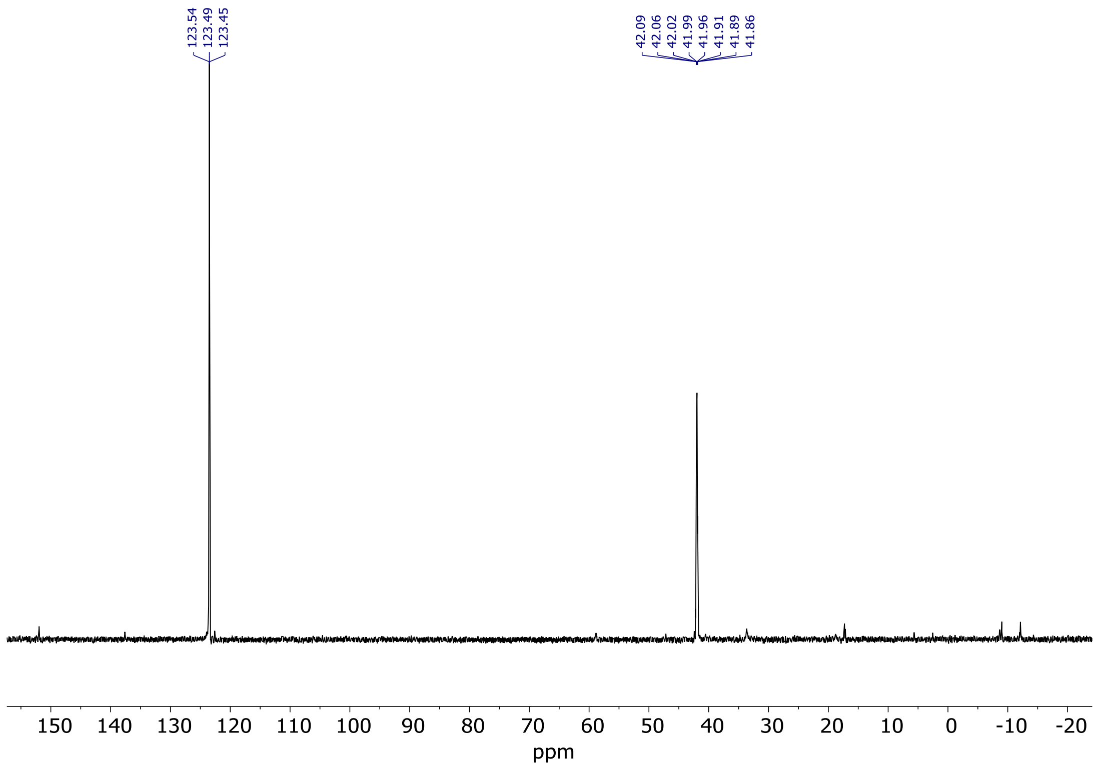

Figure S20c. ^{31}P NMR spectrum of **11**-*R* (202 MHz, 298 K, $CDCl_3$).

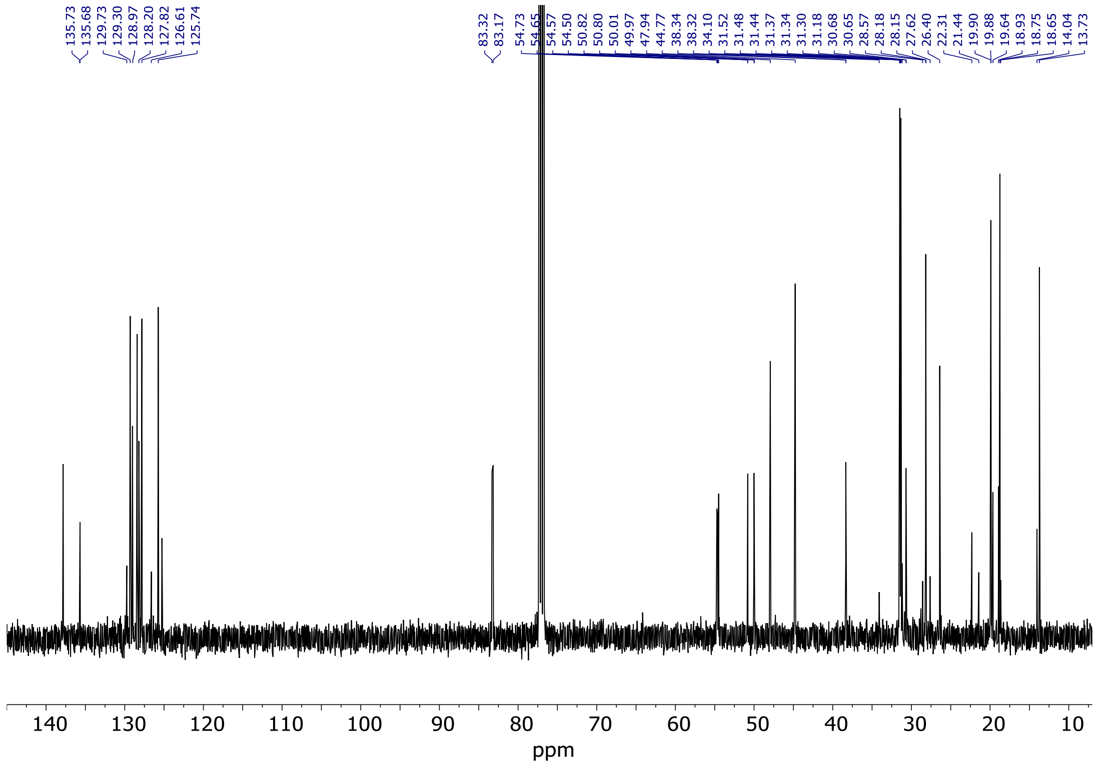

Figure S20d. $^{13}C\{^{1}H\}$ NMR spectrum of **11**-*R* (126 MHz, 298 K, $CDCl_3$).

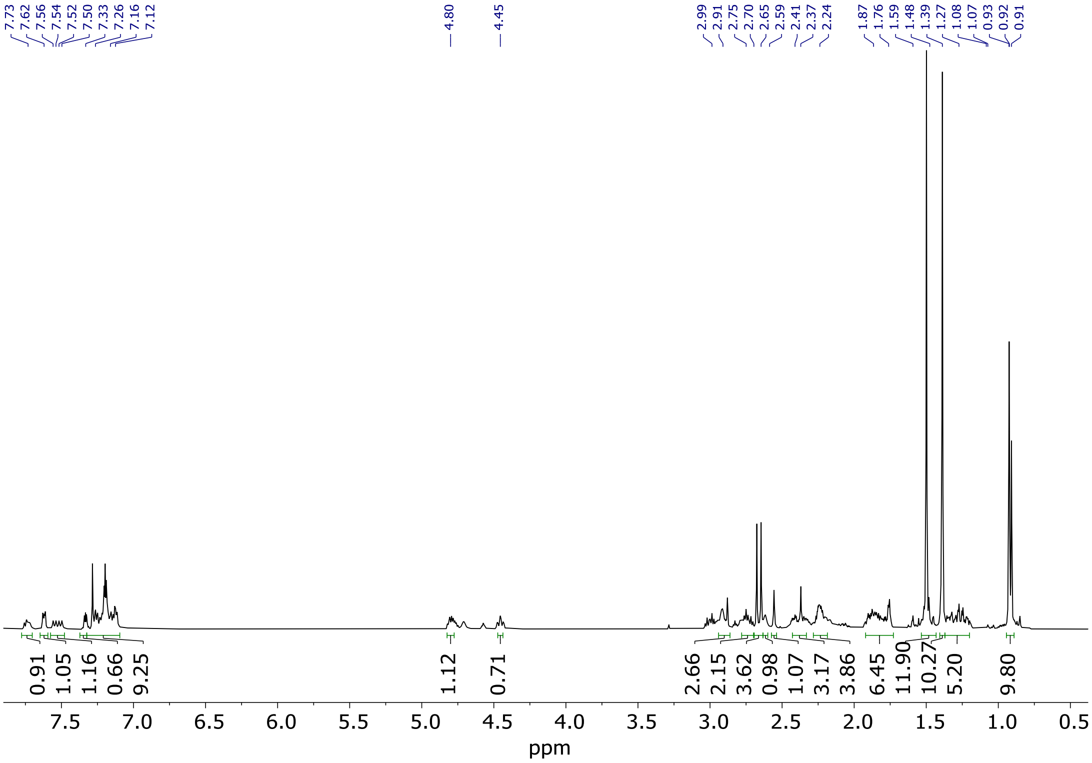

Figure S21a. ^{1}H NMR spectrum of **11**-*S* (500 MHz, 298 K, $CDCl_3$).

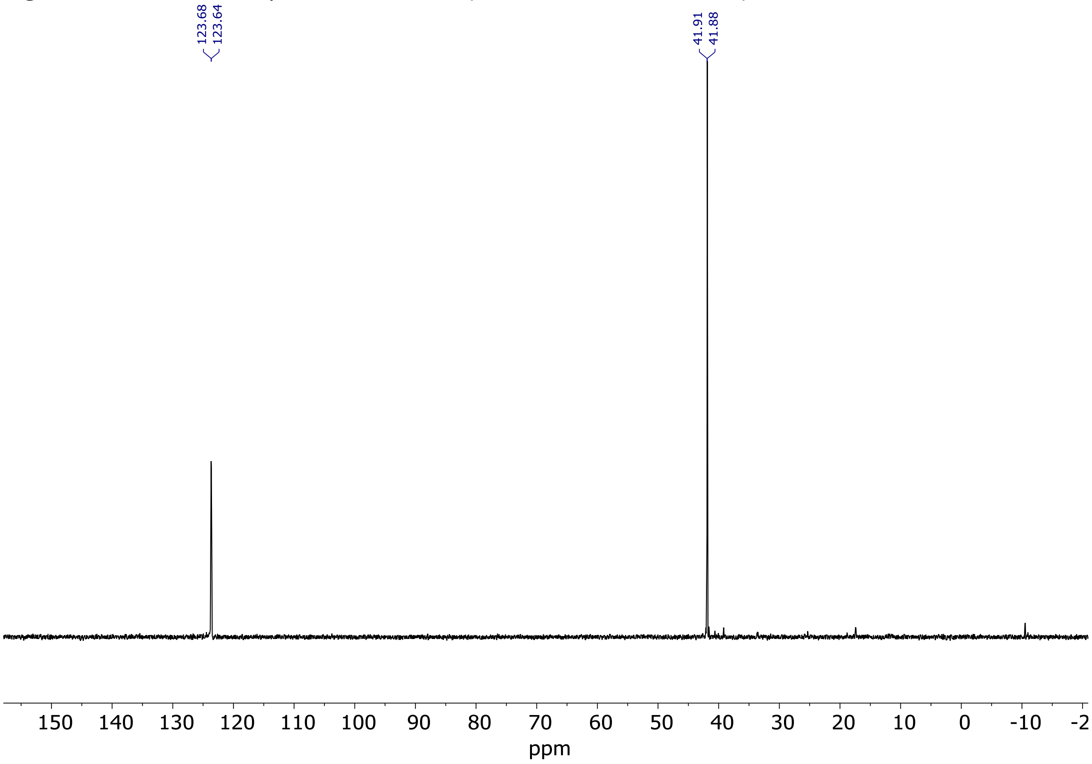

Figure S21b. $^{31}P\{^{1}H\}$ NMR spectrum of **11**-*S* (202 MHz, 298 K, $CDCl_3$).

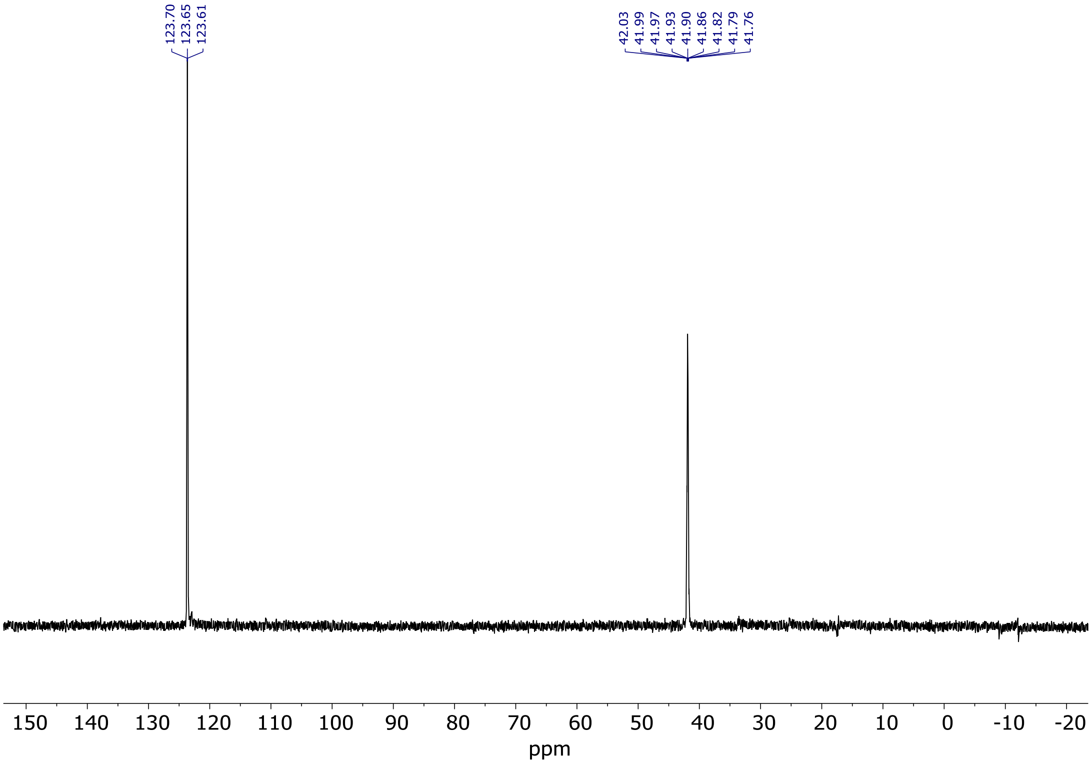

Figure S21c. ^{31}P NMR spectrum of **11**-*S* (202 MHz, 298 K, $CDCl_3$).

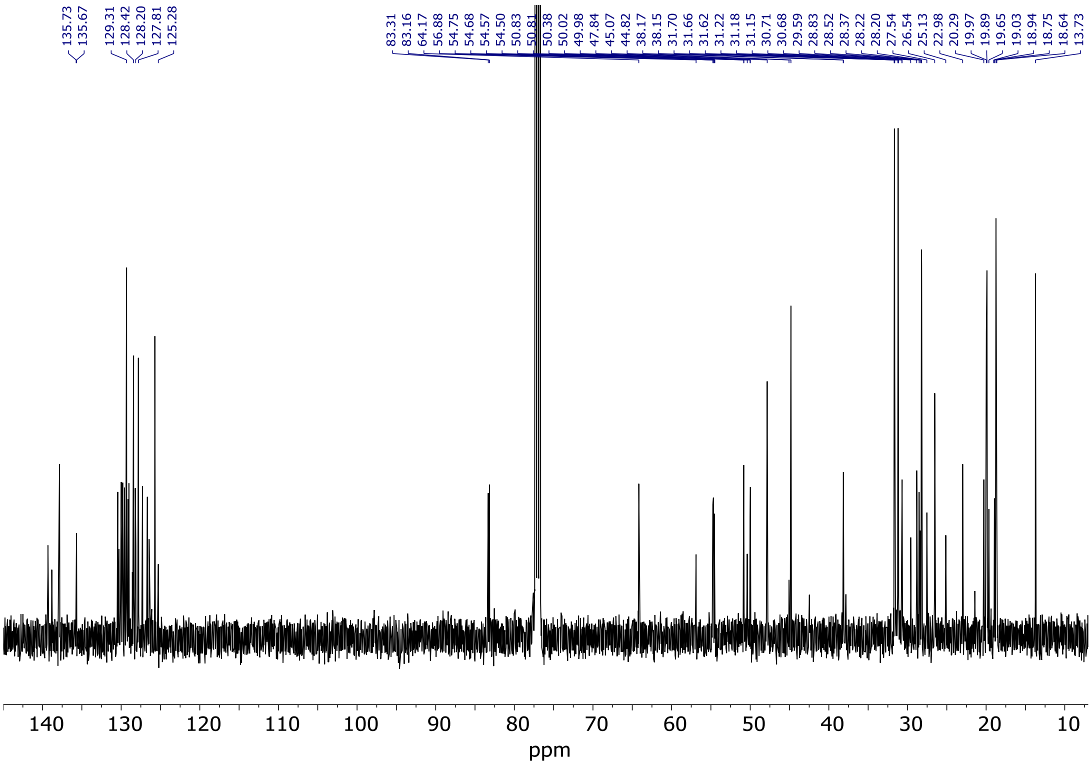

Figure S21d. $^{13}C\{^1H\}$ NMR spectrum of **11**-*S* (126 MHz, 298 K, $CDCl_3$).

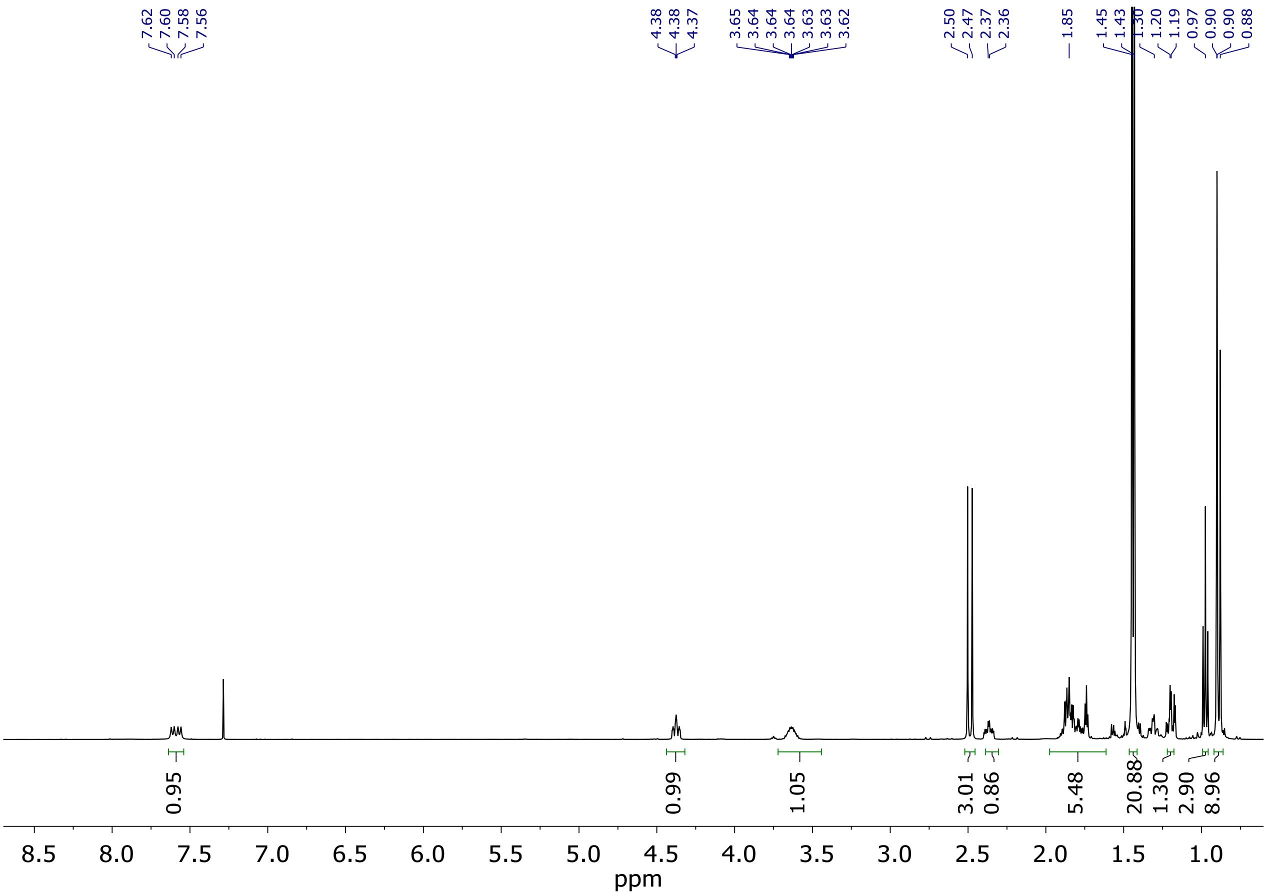

Figure S22a. ^{1}H NMR spectrum of **12**-*R* (500 MHz, 298 K, $CDCl_3$).

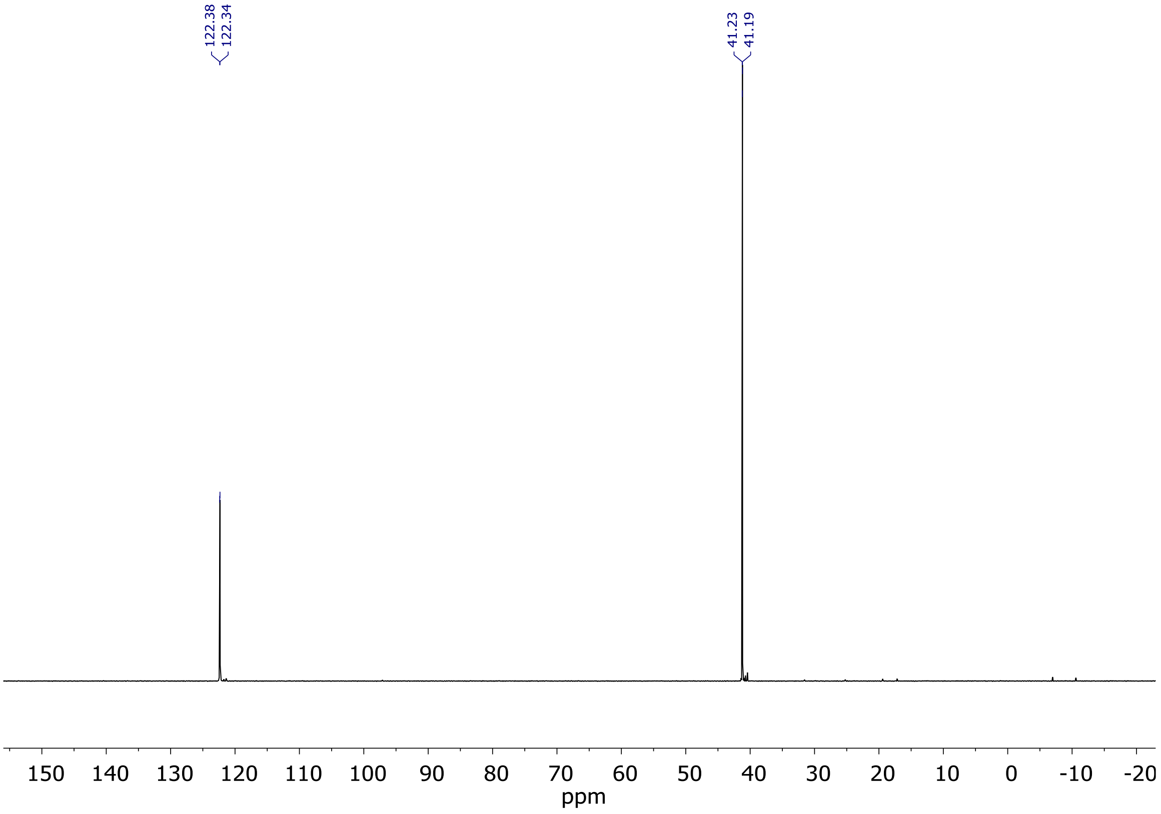

Figure S22b. $^{31}P\{^{1}H\}$ NMR spectrum of **12**-*R* (202 MHz, 298 K, $CDCl_3$).

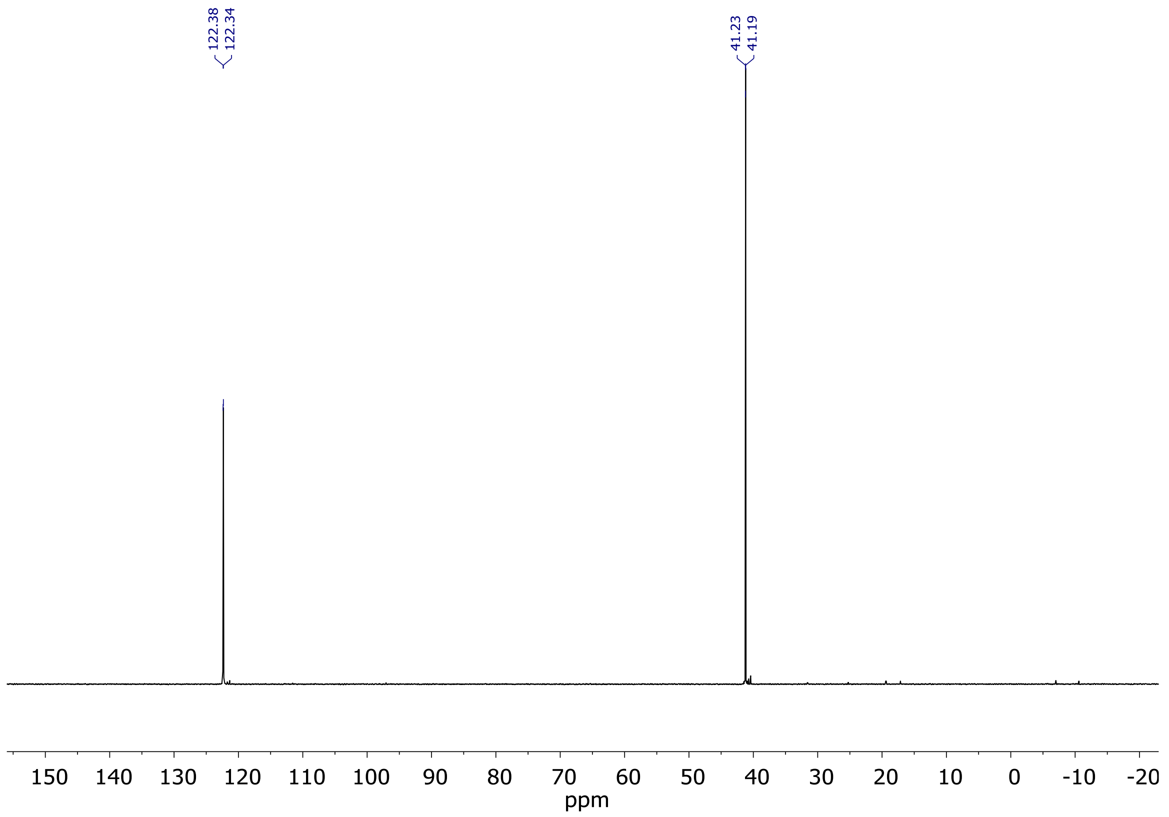

Figure S22c. $^{31}P\{^{1}H\}$ NMR spectrum of **12**-*R* (202 MHz, 298 K, $CDCl_3$; pulse program = zgig30, dummy scans = 0, recycle, delay = 30 s).

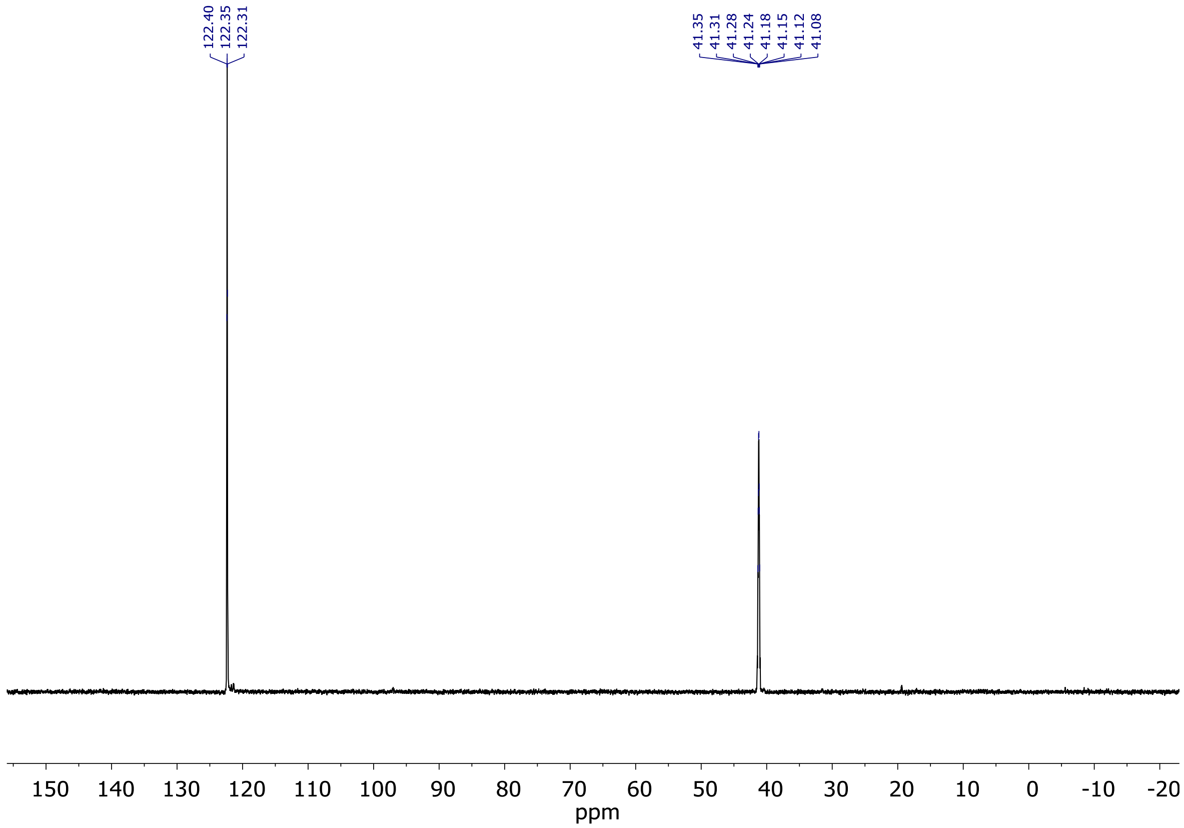

Figure S22d. ^{31}P NMR spectrum of **12**-*R* (202 MHz, 298 K, $CDCl_3$).

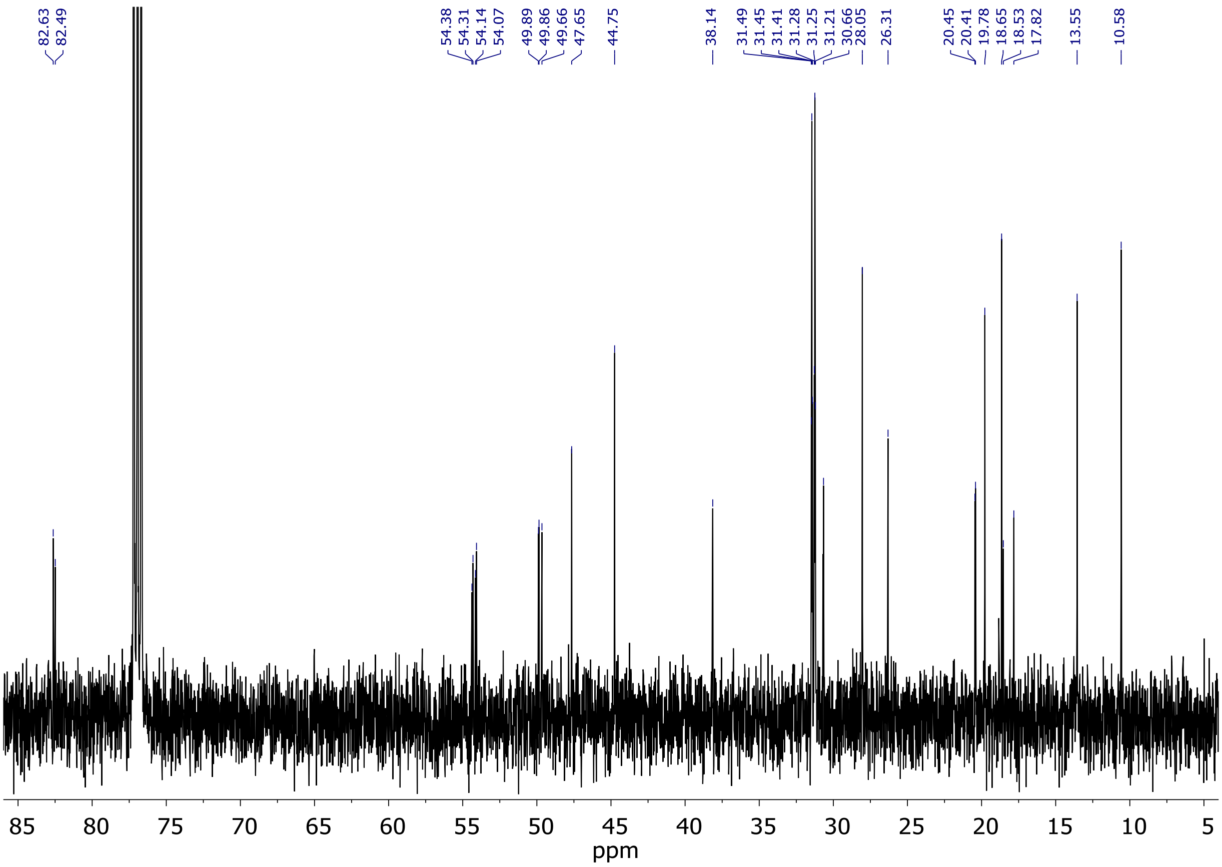

Figure S22e. $^{13}C\{^{1}H\}$ NMR spectrum of **12**-*R* (126 MHz, 298 K, $CDCl_3$).

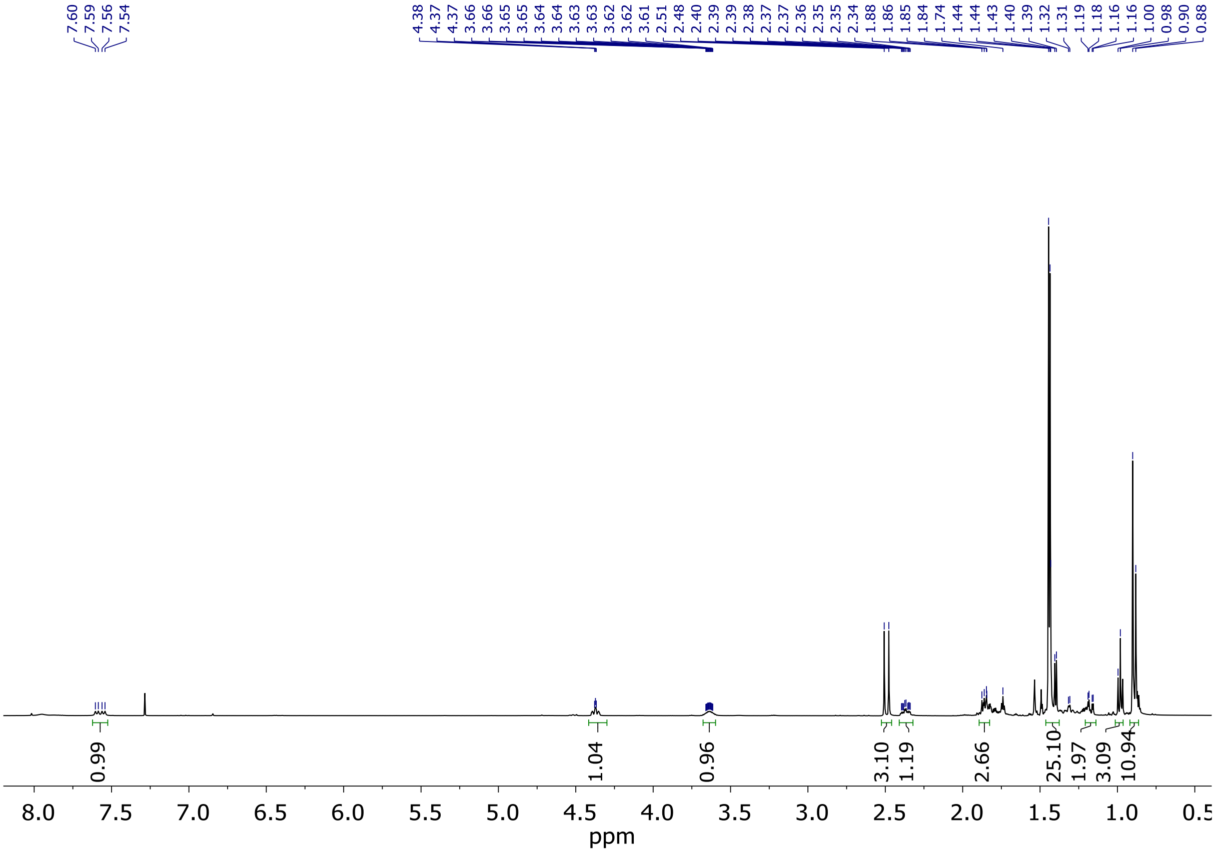

Figure S23a. ^{1}H NMR spectrum of **12**-*S* (500 MHz, 298 K, $CDCl_3$).

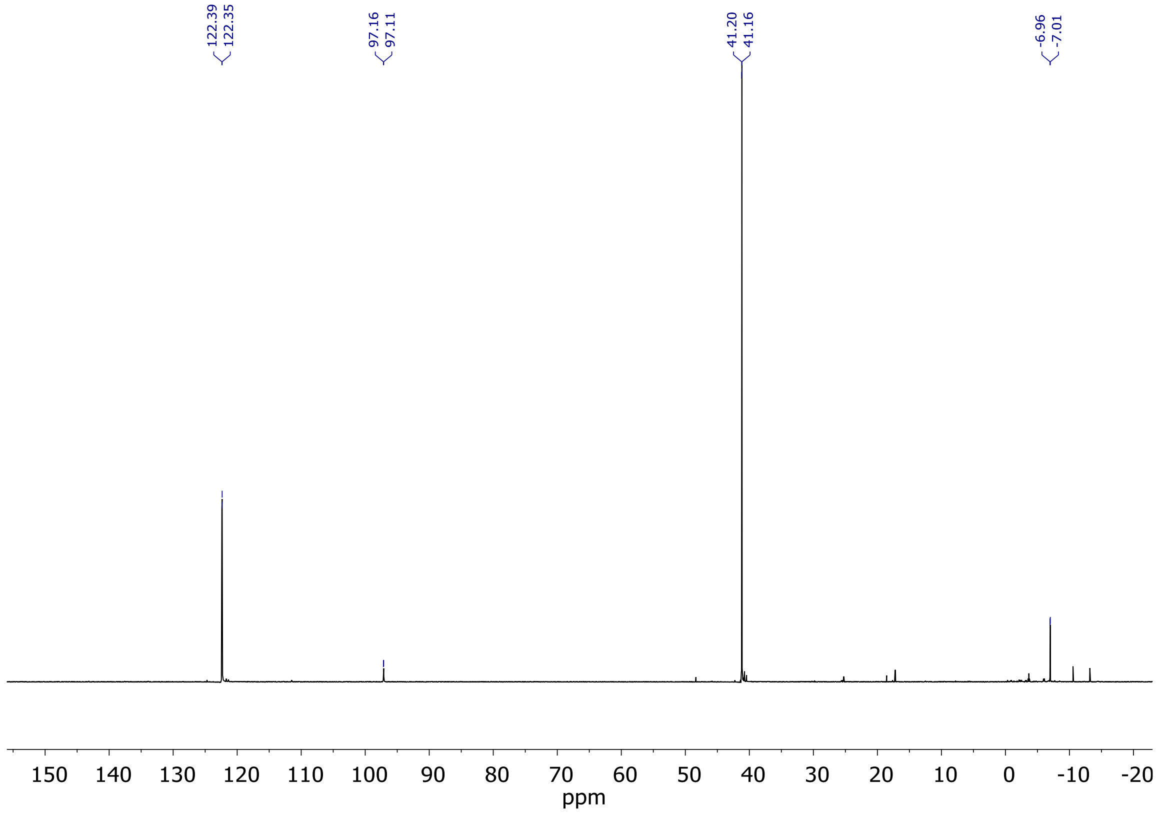

Figure S23b. $^{31}P\{^{1}H\}$ NMR spectrum of **12**-*S* (202 MHz, 298 K, $CDCl_3$). The peaks at δ 97 and −7 ppm correspond to an unidentified minor hydrolysis product.

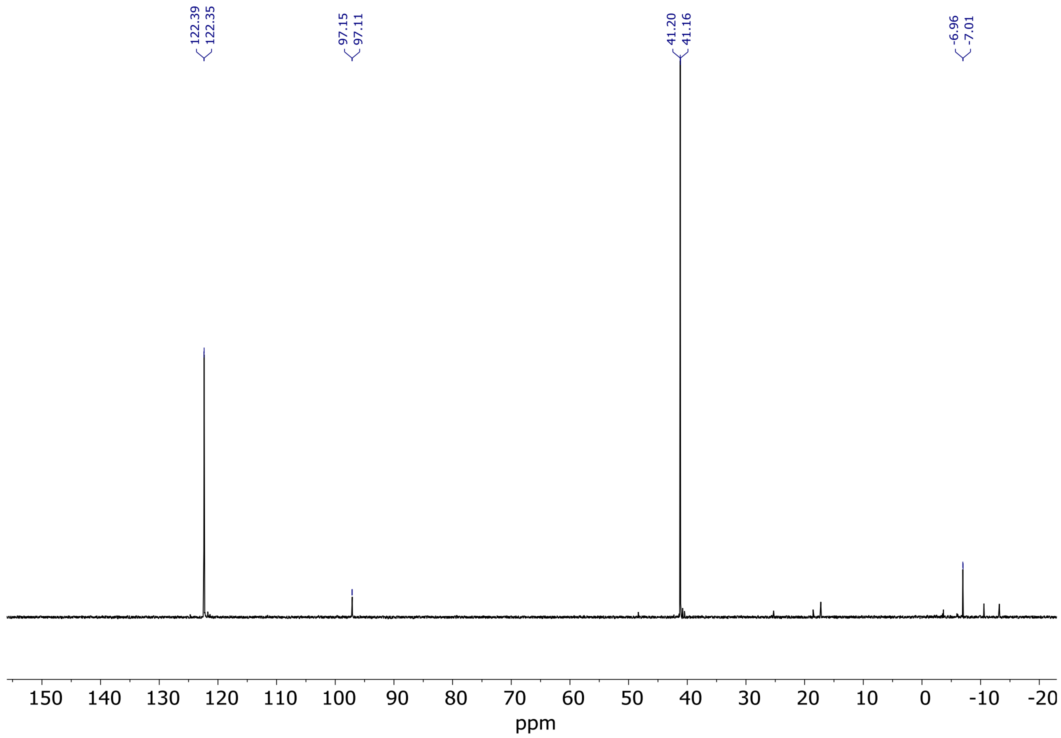

Figure S23c. $^{31}P\{^{1}H\}$ NMR spectrum of **12**-*S* (202 MHz, 298 K, $CDCl_3$; pulse program = zgig30, dummy scans = 0, recycle, delay = 30 s).

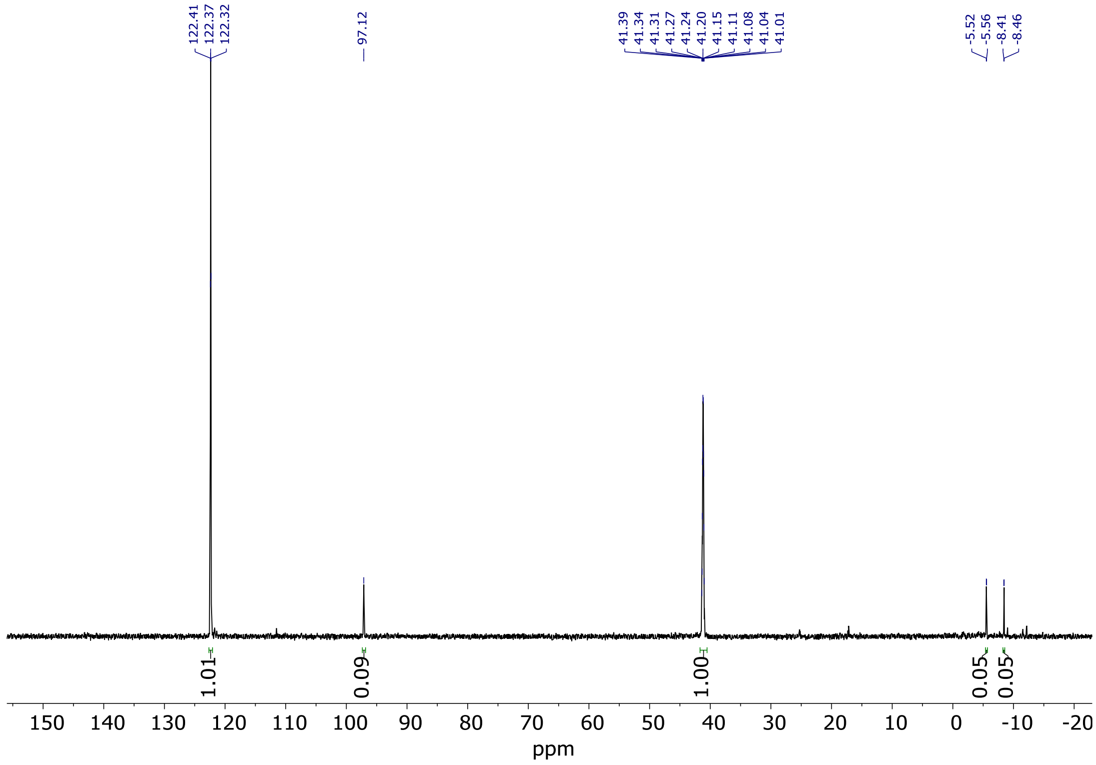

Figure S23d. ^{31}P NMR spectrum of **12**-*S* (202 MHz, 298 K, $CDCl_3$). Integration shows the minor hydrolysis product to be present in a <1:10 ratio to the main product.

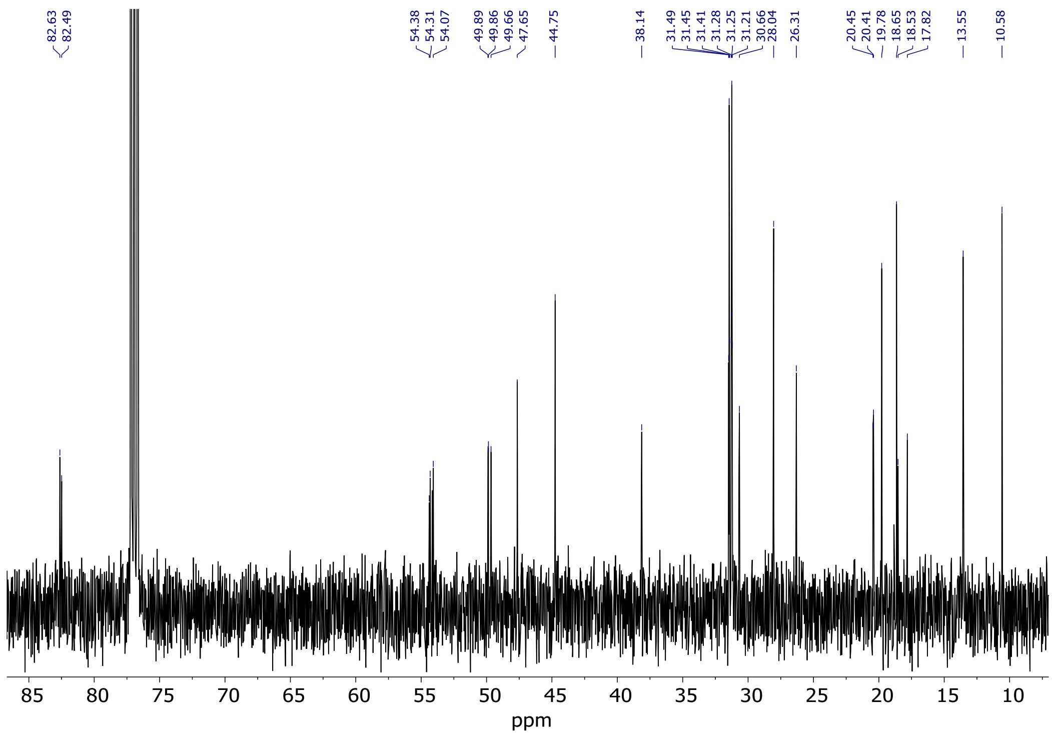

Figure S23e. $^{13}C\{^1H\}$ NMR spectrum of **12**-*S* (126 MHz, 298 K, $CDCl_3$).

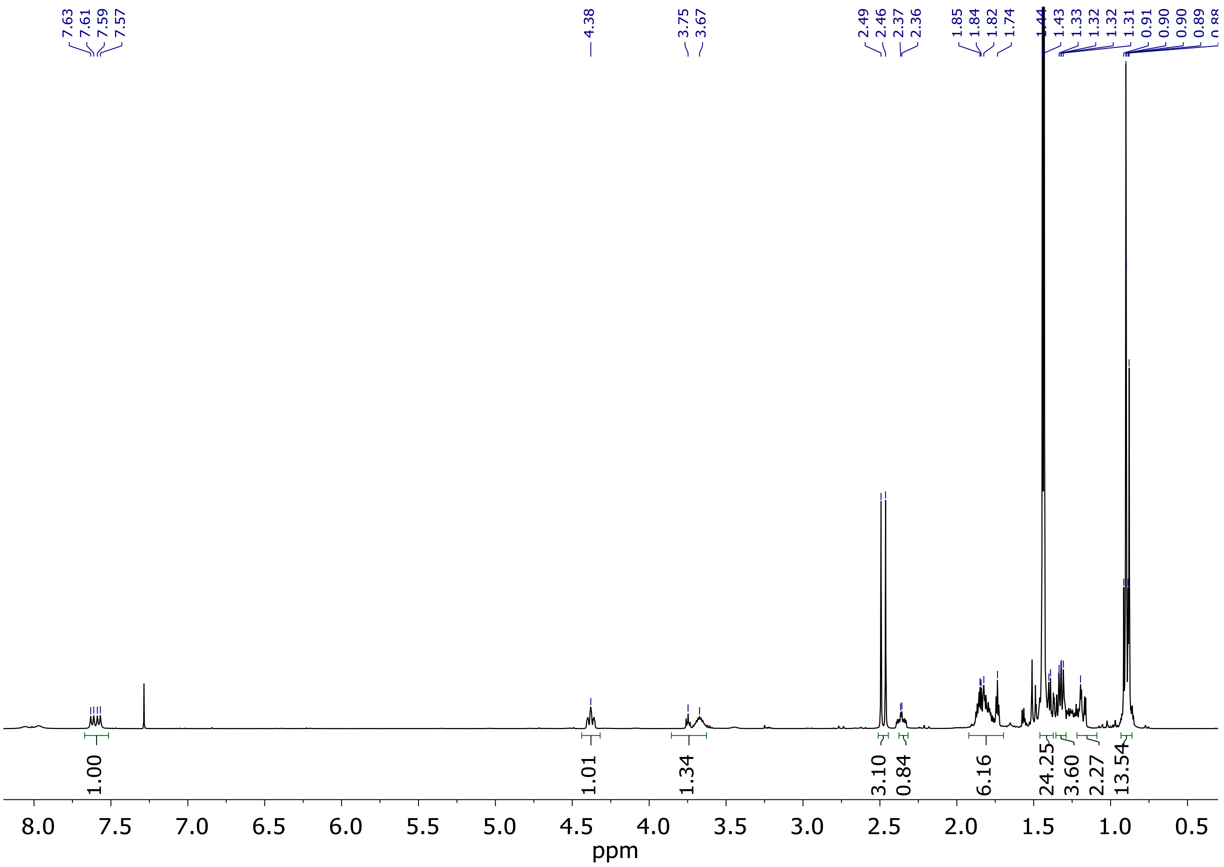

Figure S24a. ^{1}H NMR spectrum of **13**-*R* (500 MHz, 298 K, $CDCl_3$).

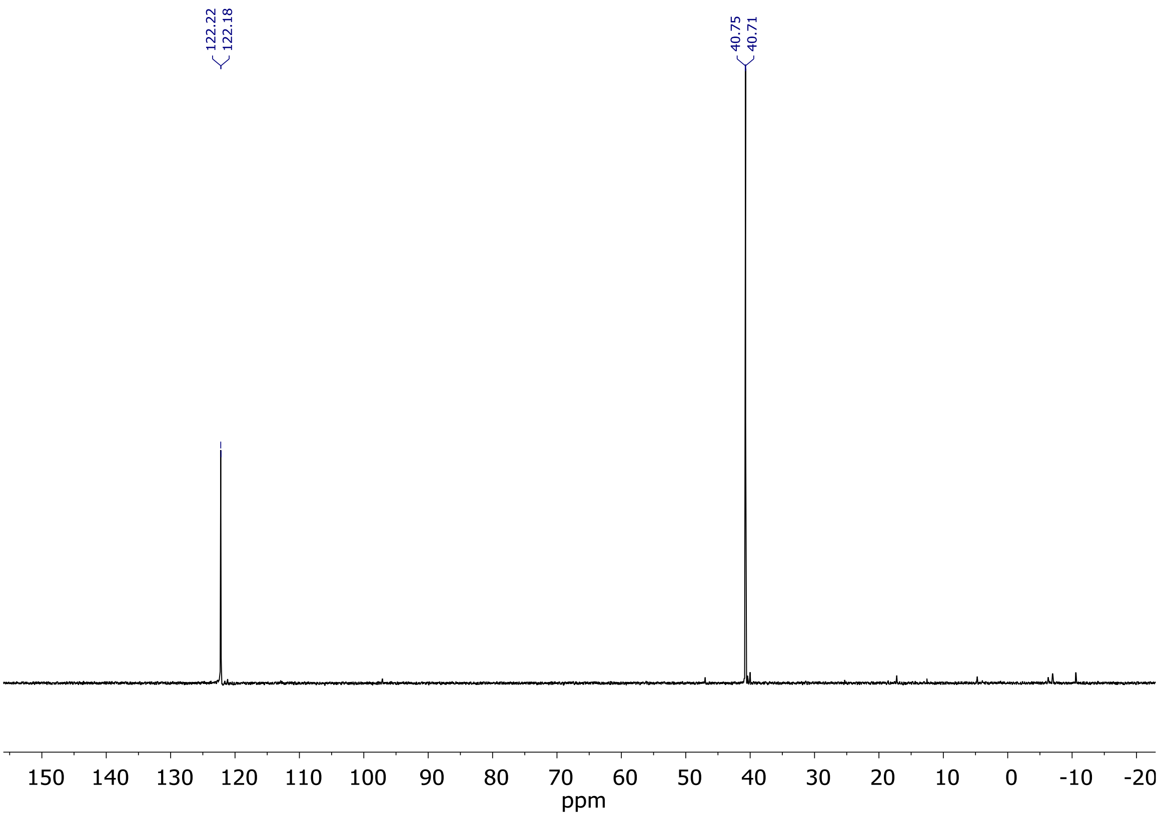

Figure S24b. $^{31}P\{^{1}H\}$ NMR spectrum of **13**-*R* (202 MHz, 298 K, $CDCl_3$).

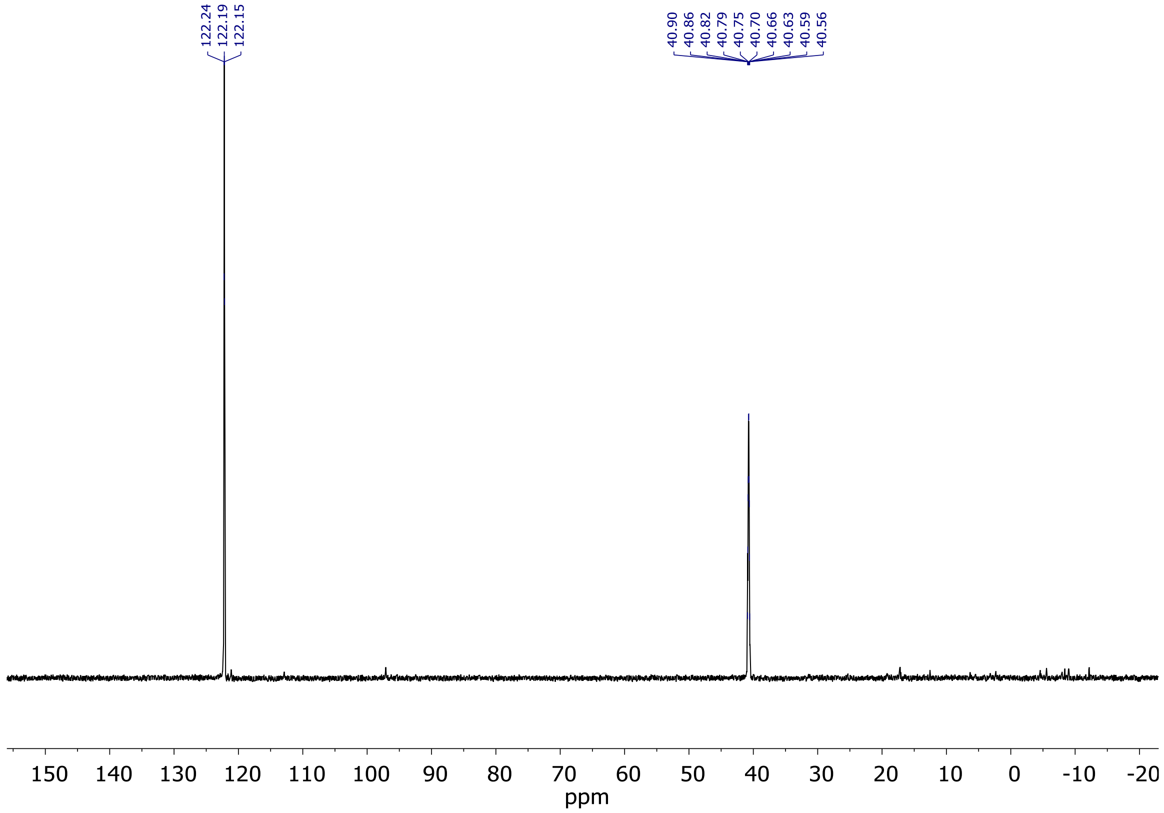

Figure S24c. ^{31}P NMR spectrum of **13**-*R* (202 MHz, 298 K, $CDCl_3$).

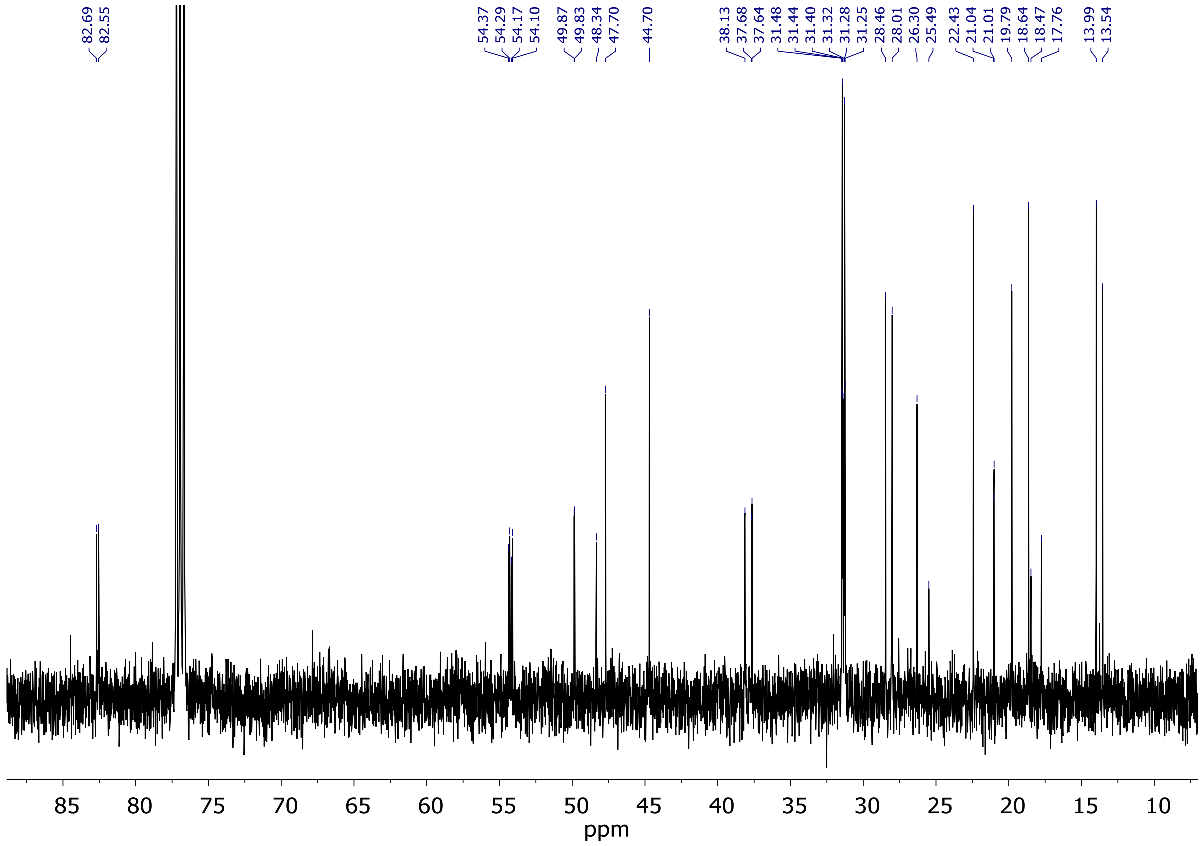

Figure S24d. $^{13}C\{^1H\}$ NMR spectrum of **13**-*R* (126 MHz, 298 K, $CDCl_3$).

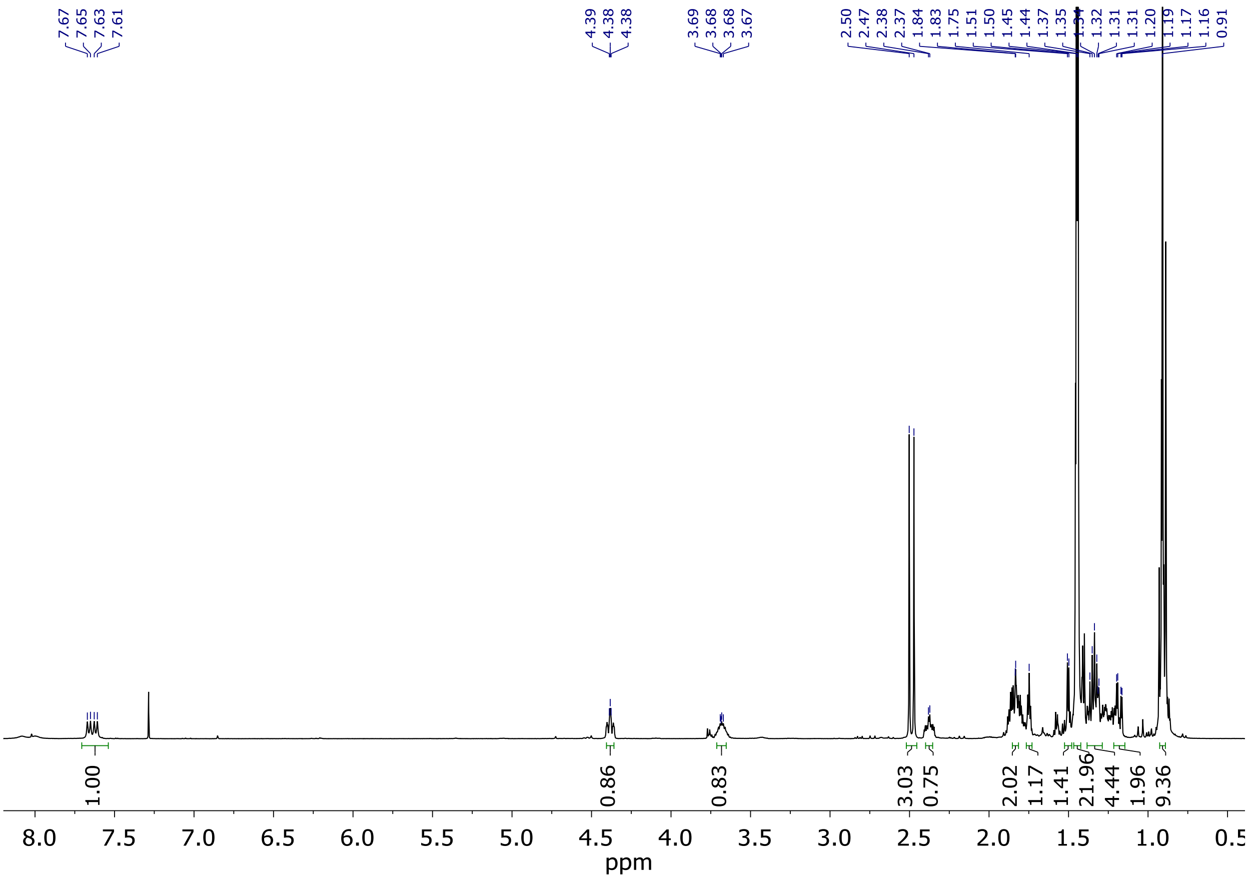

Figure S25a. ^{1}H NMR spectrum of **13**-*S* (500 MHz, 298 K, $CDCl_3$).

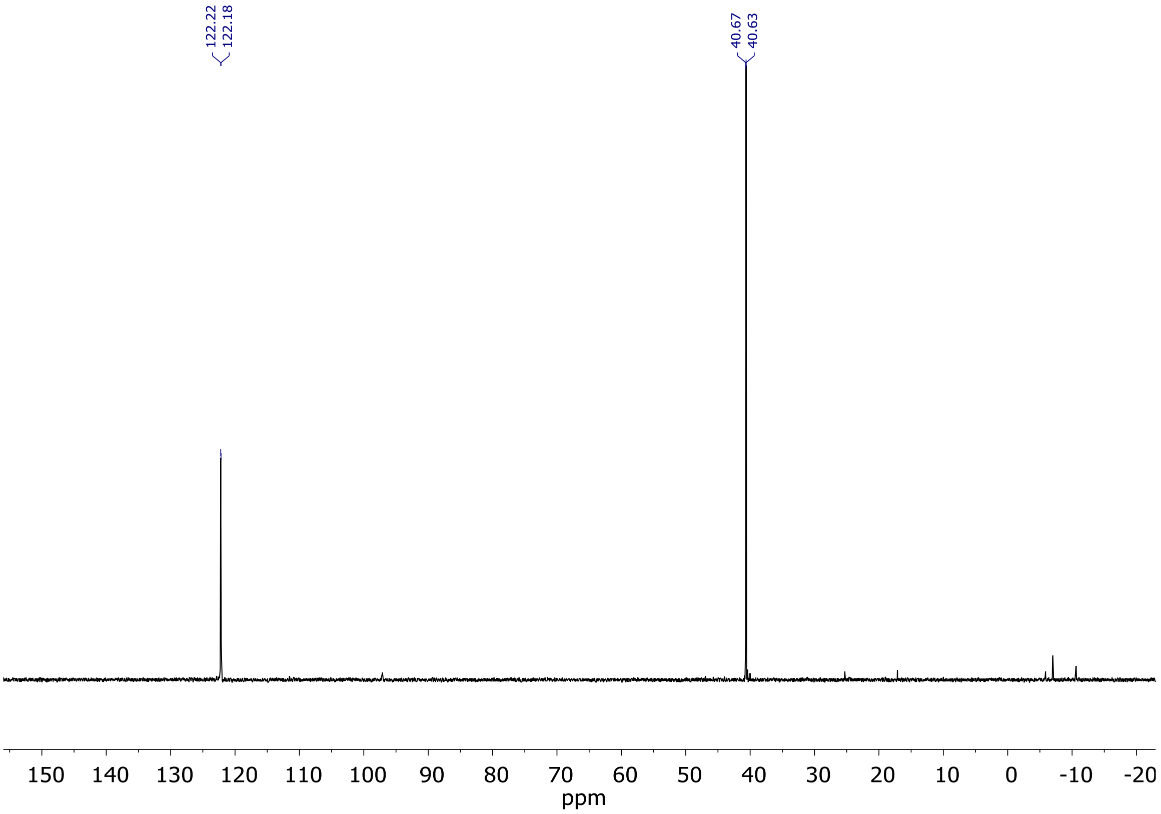

Figure S25b. $^{31}P\{^{1}H\}$ NMR spectrum of **13**-*S* (202 MHz, 298 K, $CDCl_3$).

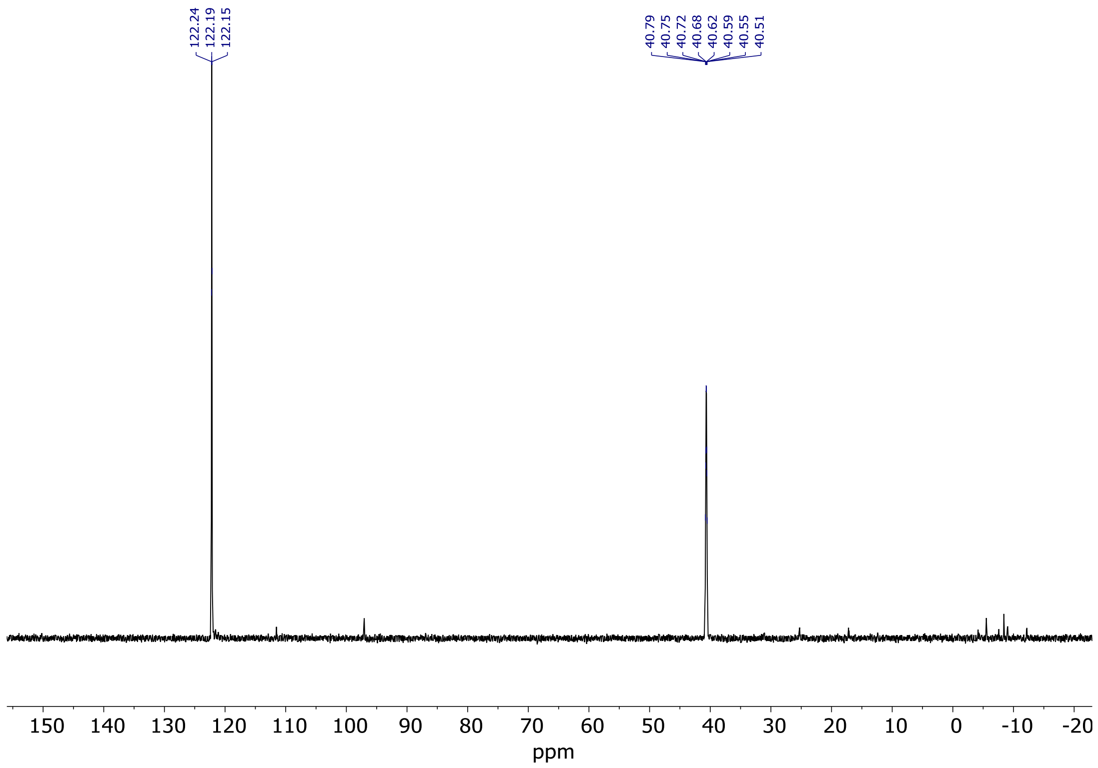

Figure S25c. ^{31}P NMR spectrum of **13**-*S* (202 MHz, 298 K, $CDCl_3$).

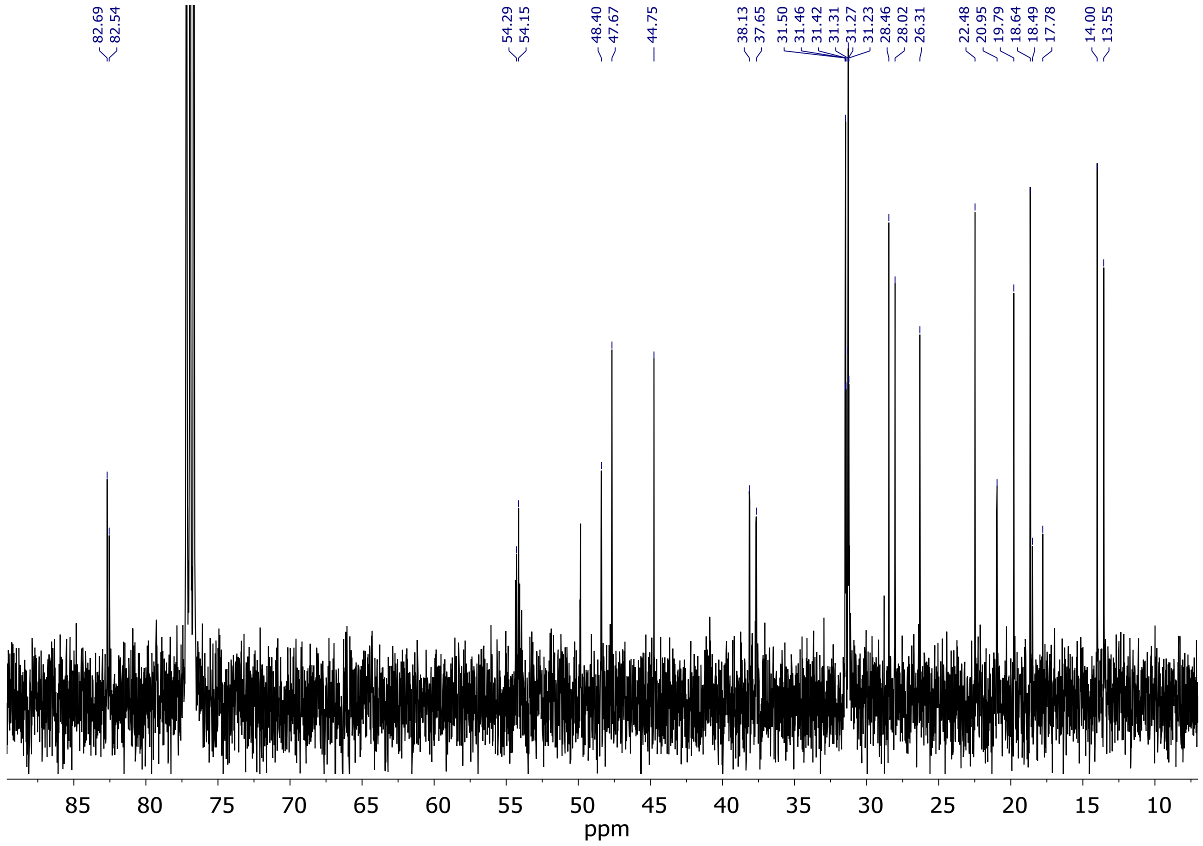

Figure S25d. $^{13}C\{^{1}H\}$ NMR spectrum of **13**-*S* (126 MHz, 298 K, $CDCl_3$).

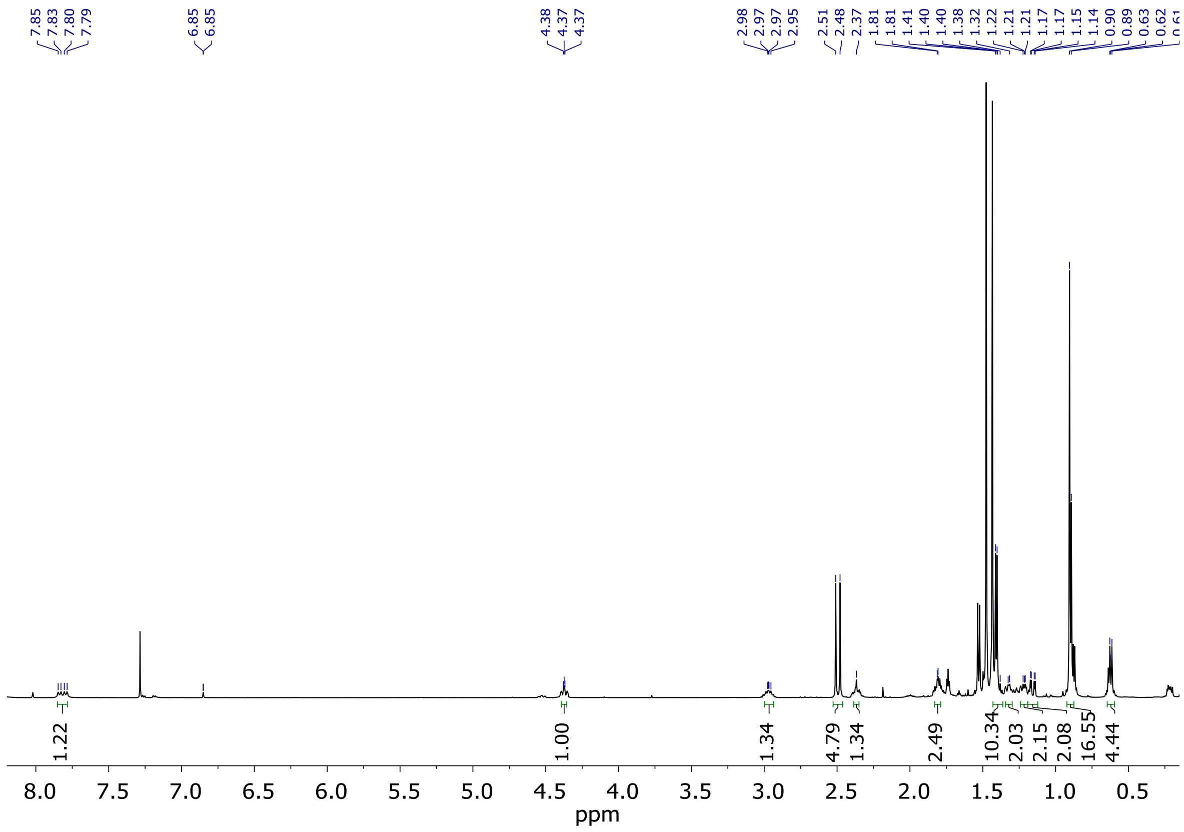

Figure S26a. ^{1}H NMR spectrum of **14**-*R* (500 MHz, 298 K, $CDCl_3$).

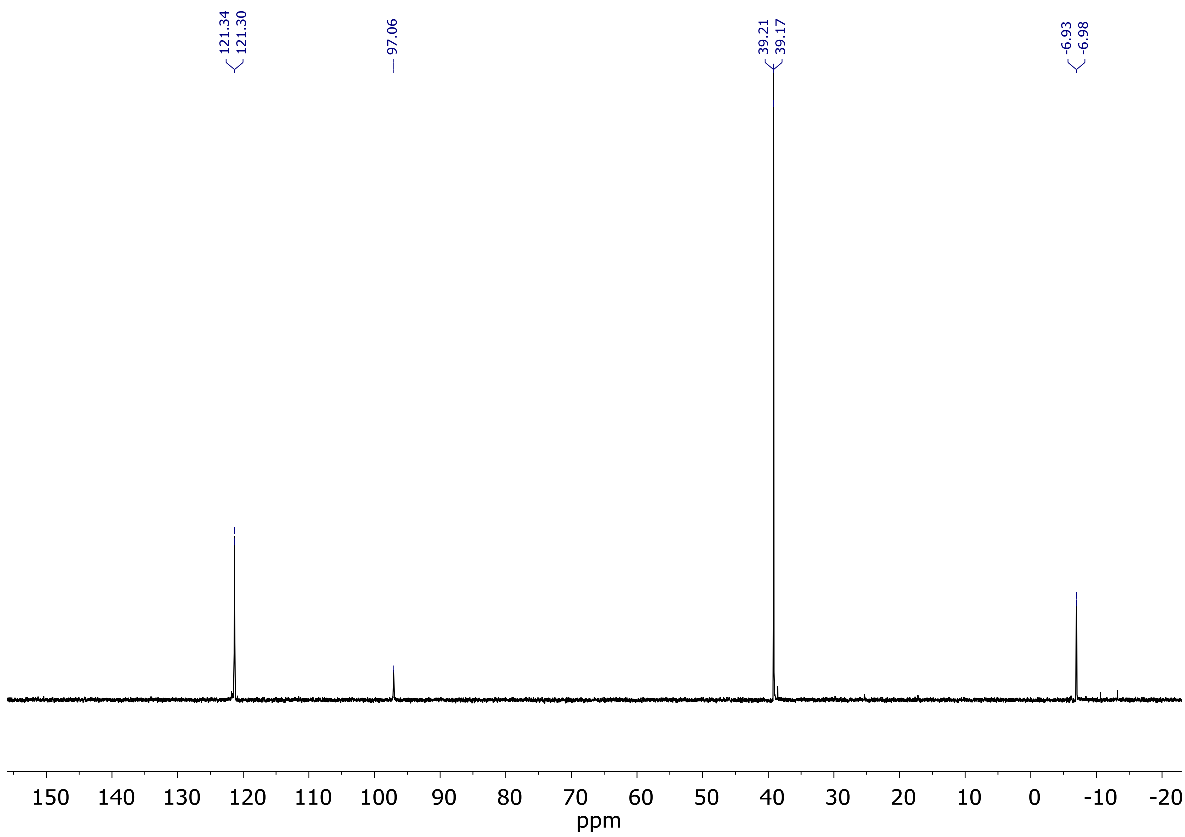

Figure S26b. $^{31}P\{^{1}H\}$ NMR spectrum of **14**-*R* (202 MHz, 298 K, $CDCl_3$). The peaks at *δ* 97 and −7 ppm correspond to an unidentified minor hydrolysis product.

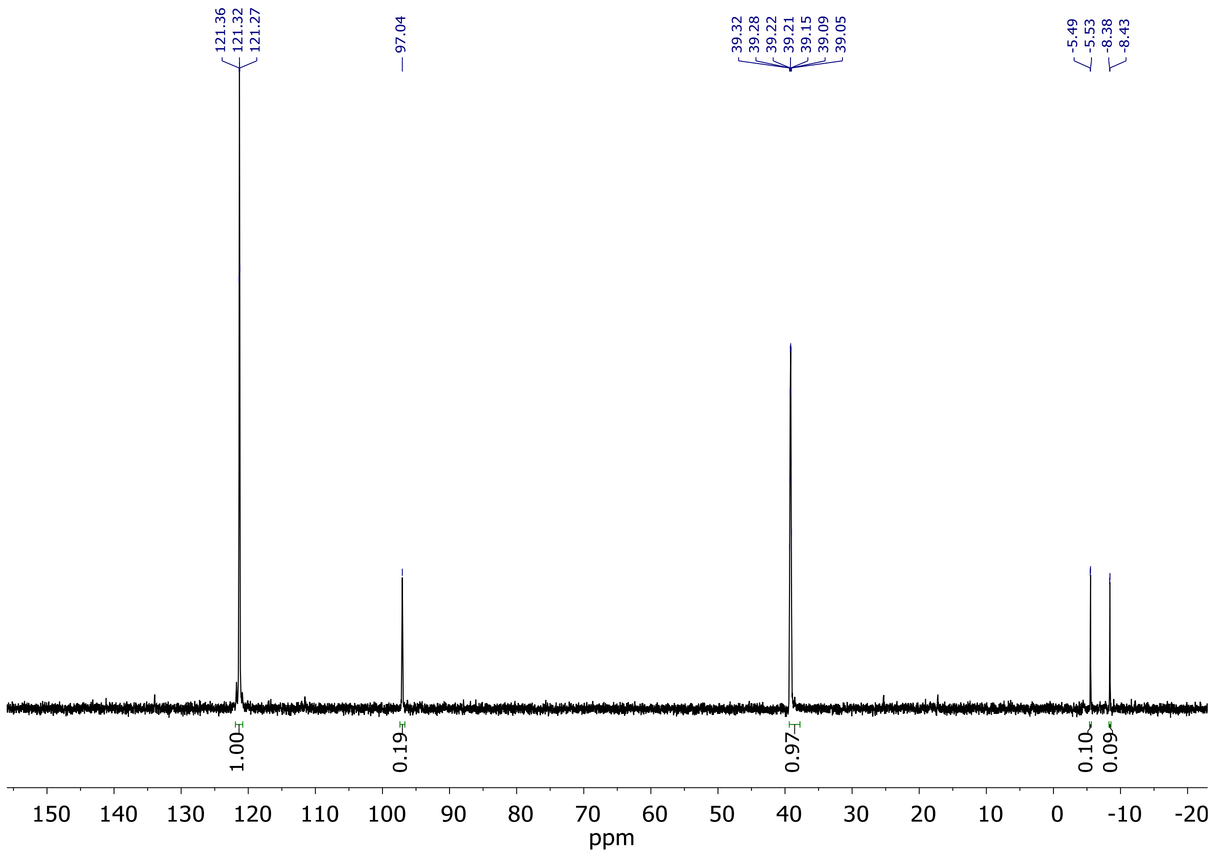

Figure S26c. ^{31}P NMR spectrum of **14**-*R* (202 MHz, 298 K, $CDCl_3$). Integration shows the minor hydrolysis product to be present in a 1:5 ratio to the main product.

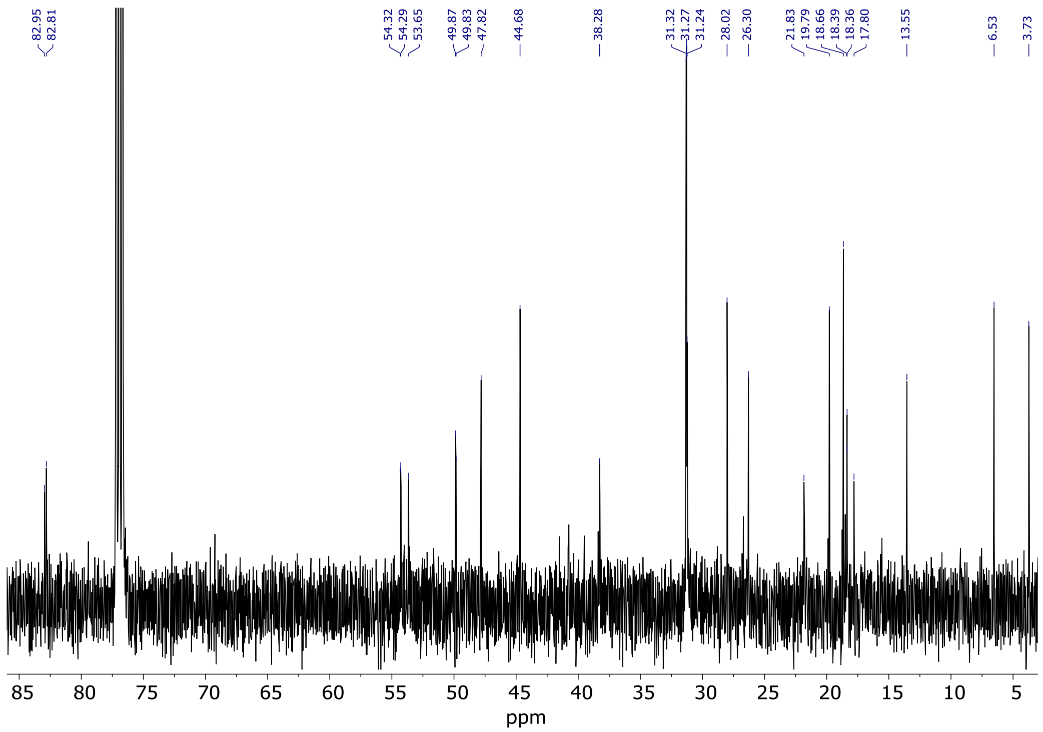

Figure S26d. $^{13}C\{^1H\}$ NMR spectrum of **14**-*R* (126 MHz, 298 K, $CDCl_3$).

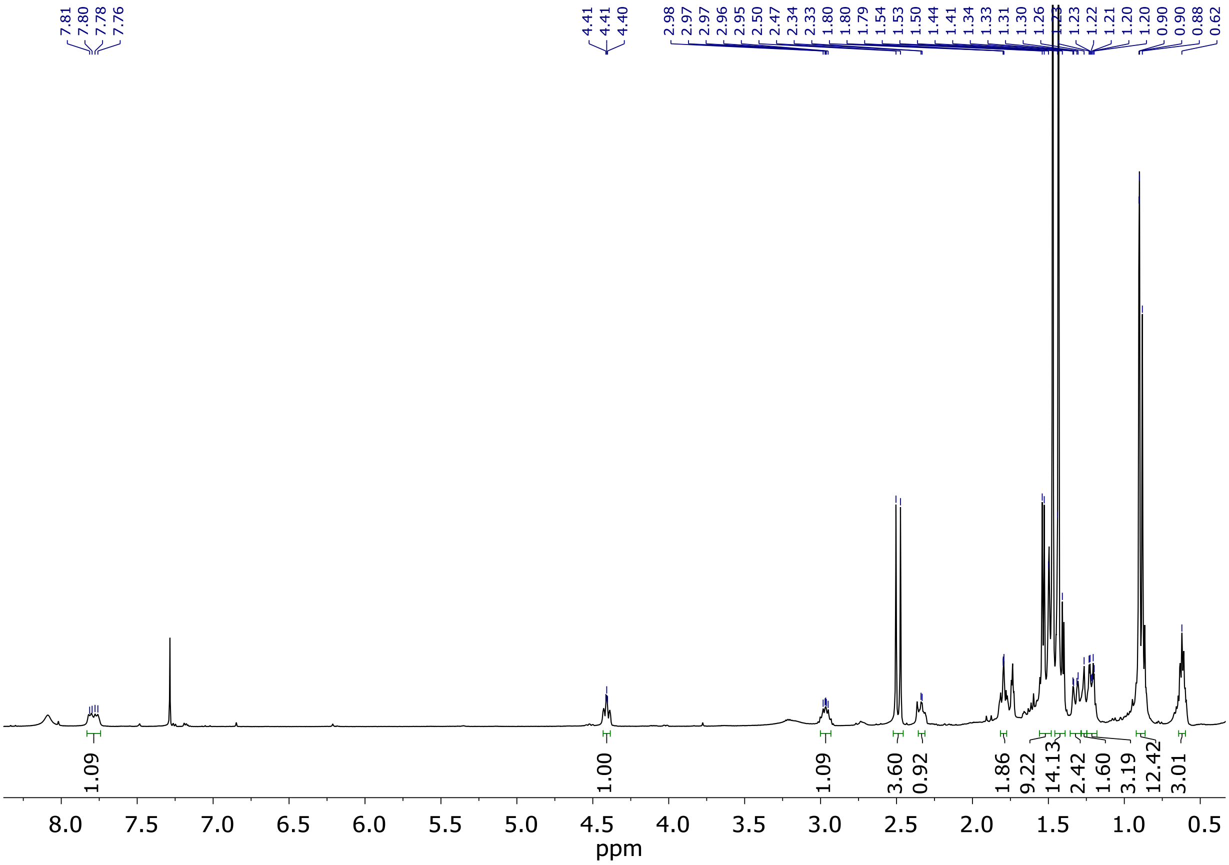

Figure S27a. ^{1}H NMR spectrum of **14**-*S* (500 MHz, 298 K, $CDCl_3$).

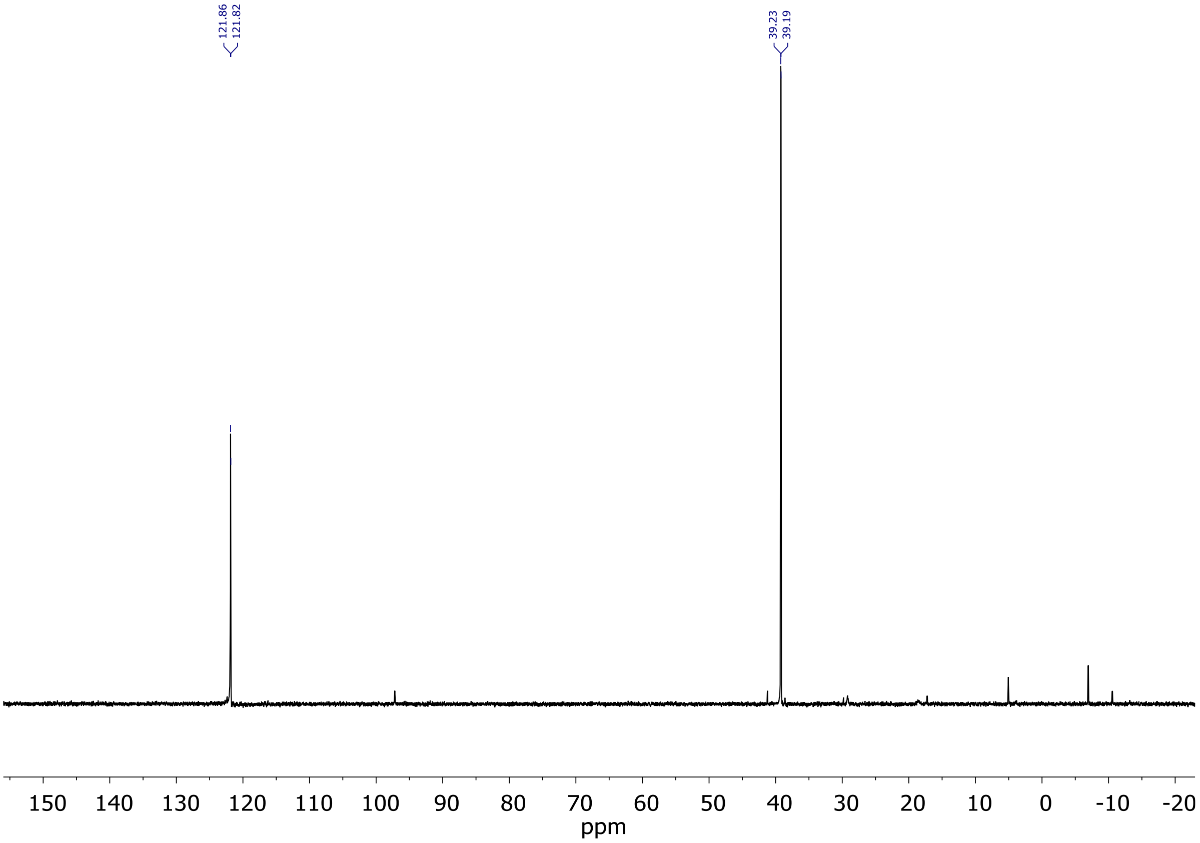

Figure S27b. $^{31}P\{^{1}H\}$ NMR spectrum of **14**-*S* (202 MHz, 298 K, $CDCl_3$).

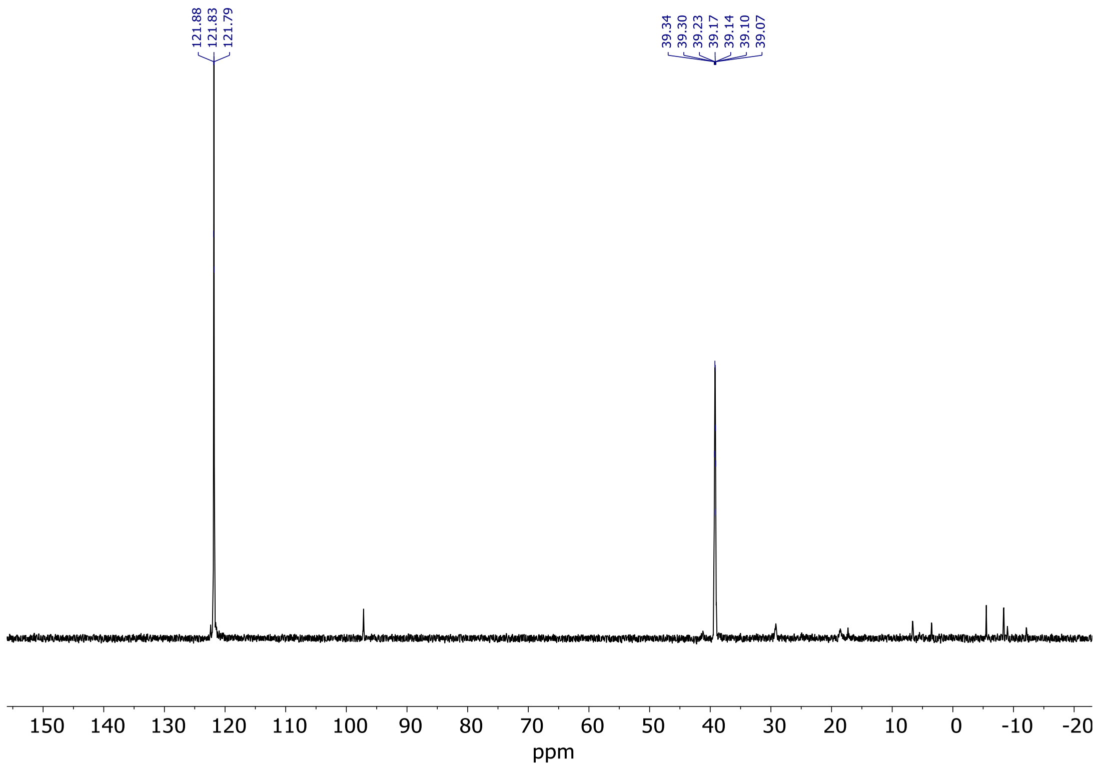

Figure S27c. ^{31}P NMR spectrum of **14**-*S* (202 MHz, 298 K, $CDCl_3$).

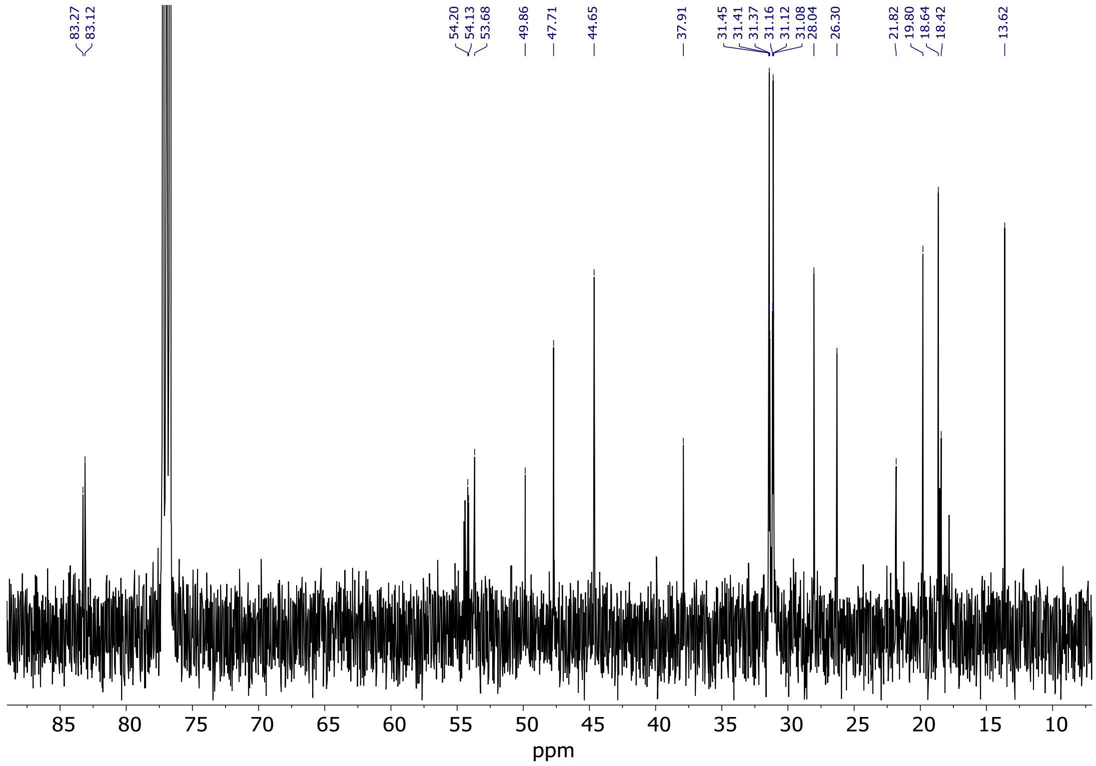

Figure S27d. $^{13}C\{^1H\}$ NMR spectrum of **14**-*S* (126 MHz, 298 K, $CDCl_3$).

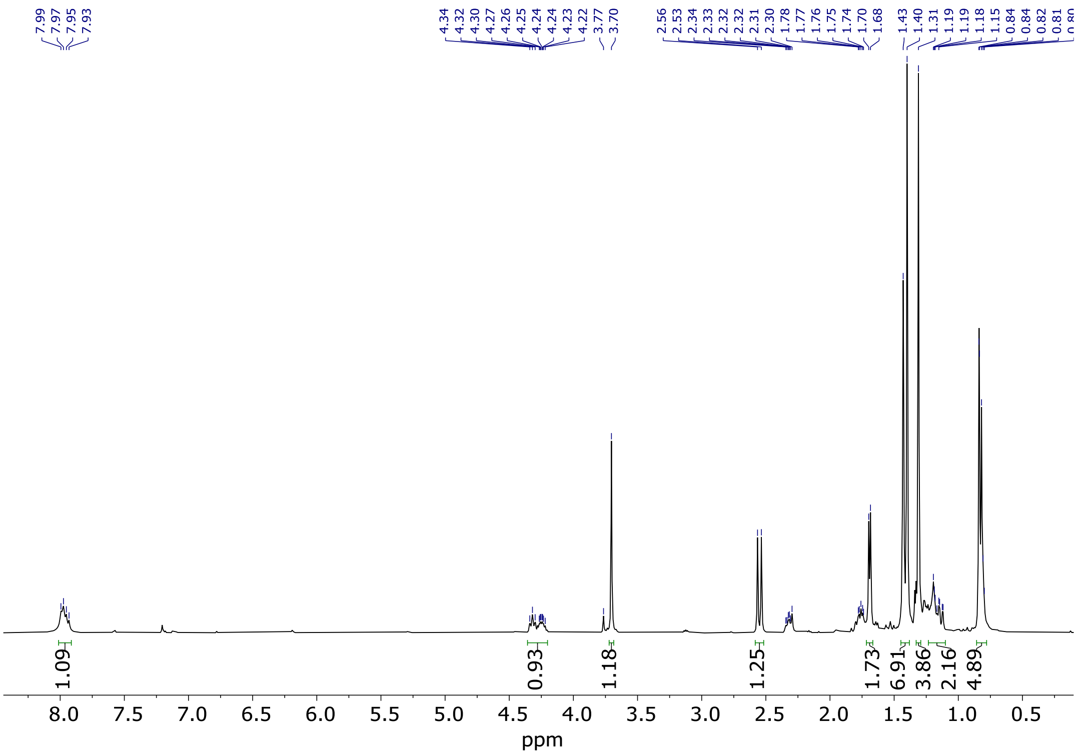

Figure S28a. ^{1}H NMR spectrum of **15**-*R* (500 MHz, 298 K, $CDCl_3$).

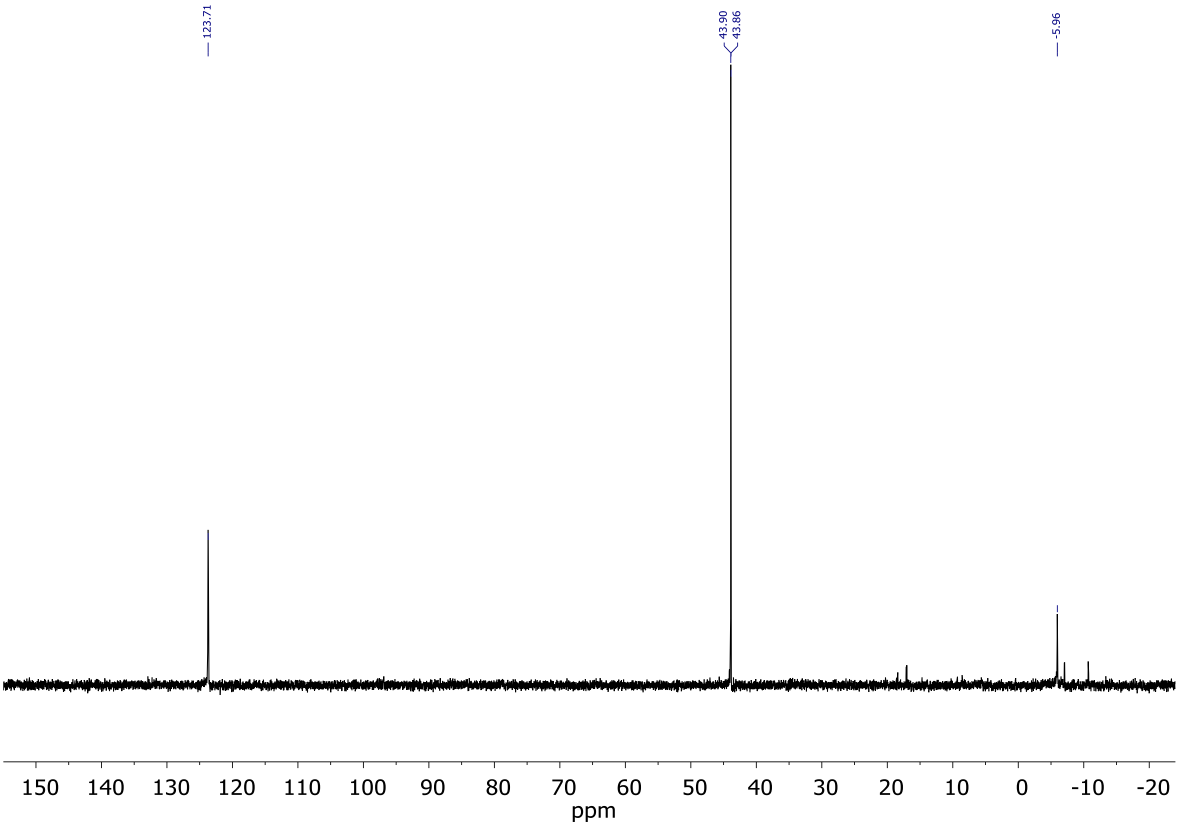

Figure S28b. $^{31}P\{^{1}H\}$ NMR spectrum of **15**-*R* (202 MHz, 298 K, $CDCl_3$). The peak at δ −6 ppm corresponds to an unidentified minor hydrolysis product.

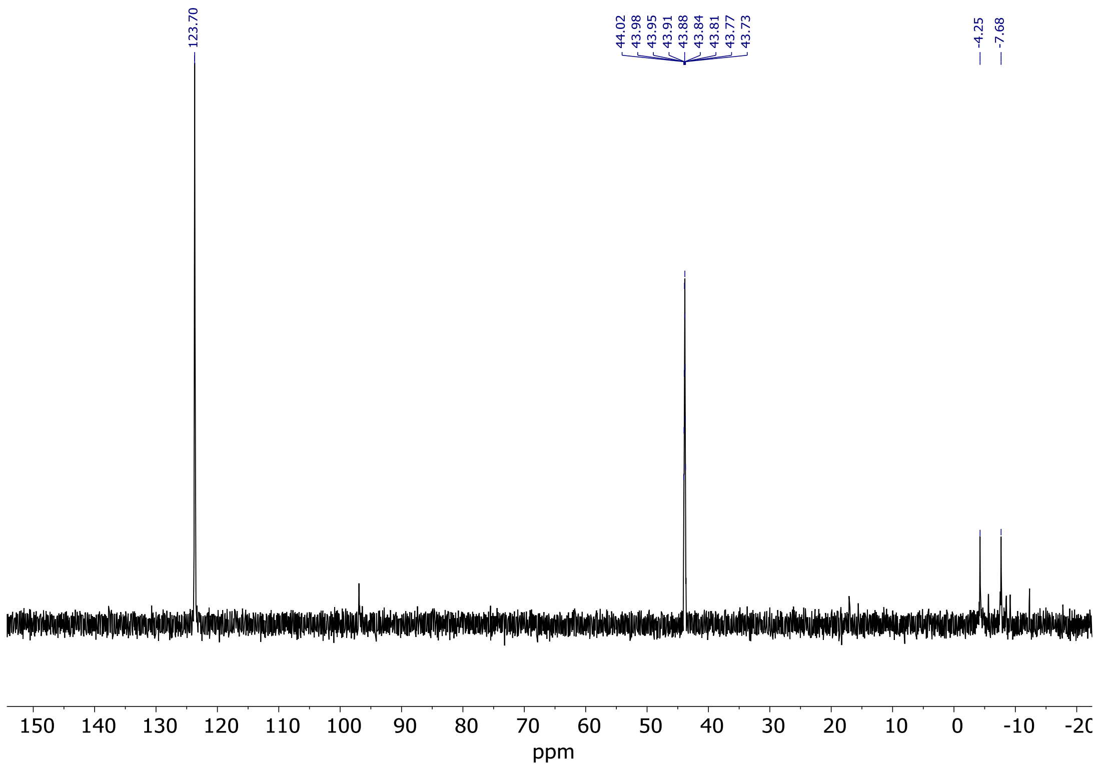

Figure S28c. ^{31}P NMR spectrum of **15**-*R* (202 MHz, 298 K, $CDCl_3$).

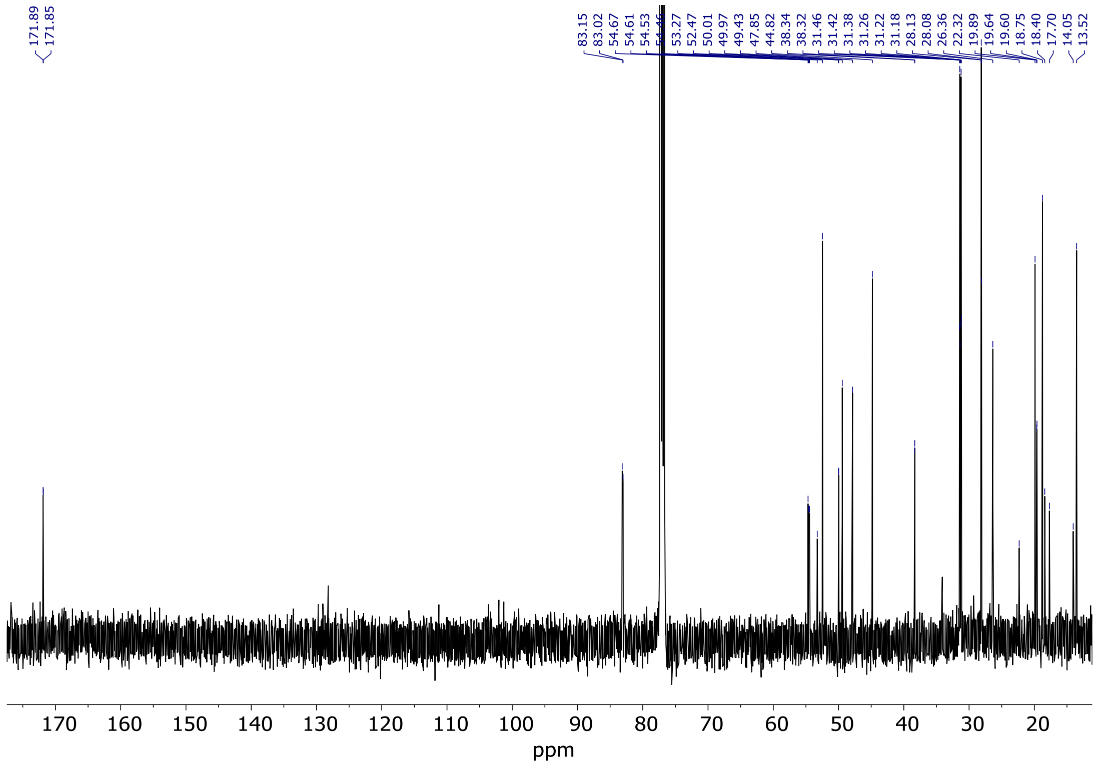

Figure S28d. $^{13}C\{^{1}H\}$ NMR spectrum of **15**-*R* (126 MHz, 298 K, $CDCl_3$).

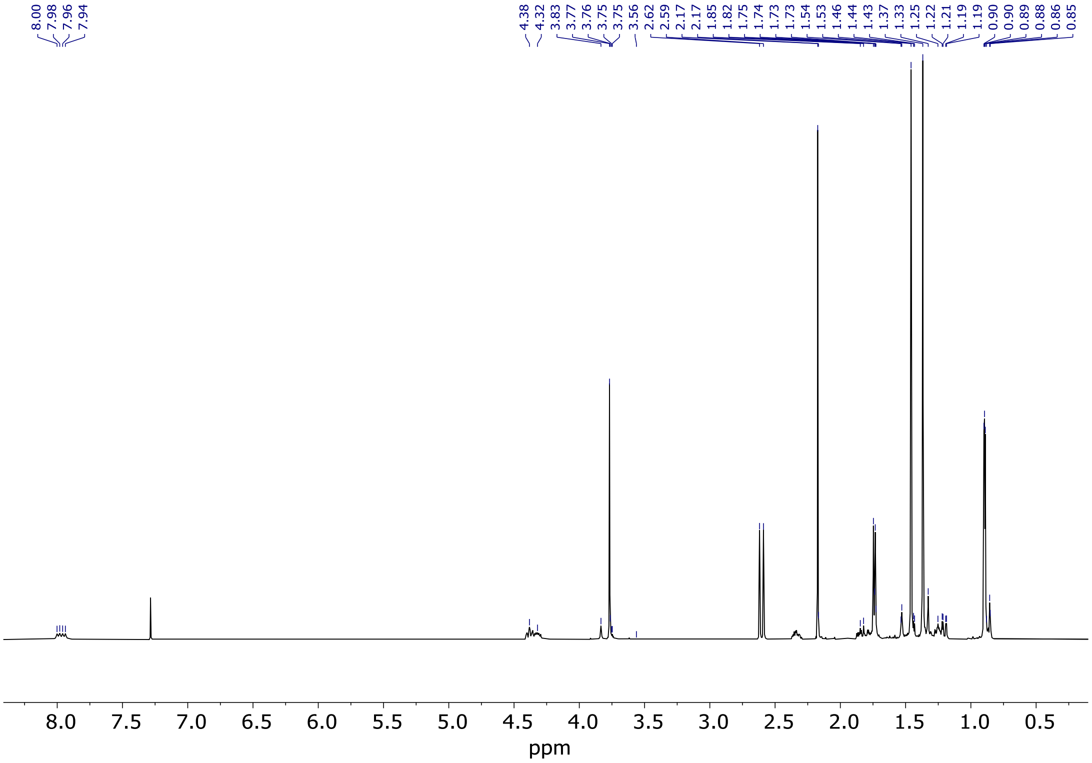

Figure S29a. ^{1}H NMR spectrum of **15-*S*** (500 MHz, 298 K, $CDCl_3$).

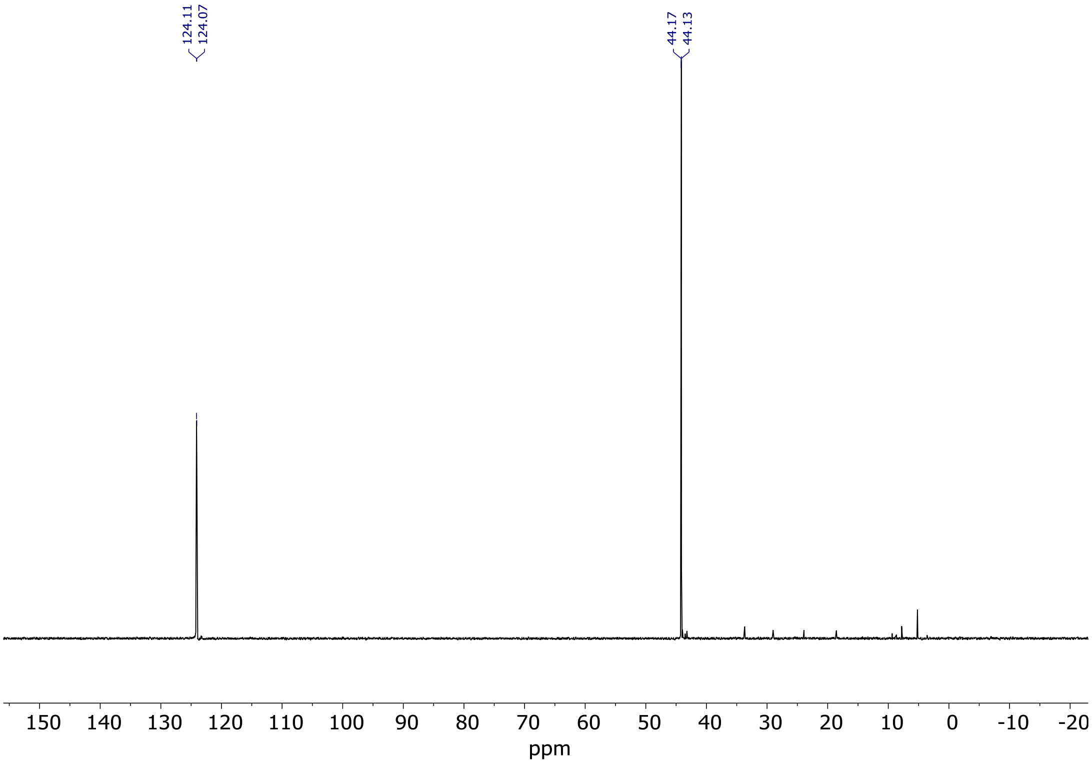

Figure S29b. $^{31}P\{^{1}H\}$ NMR spectrum of **15**-*S* (202 MHz, 298 K, $CDCl_3$).

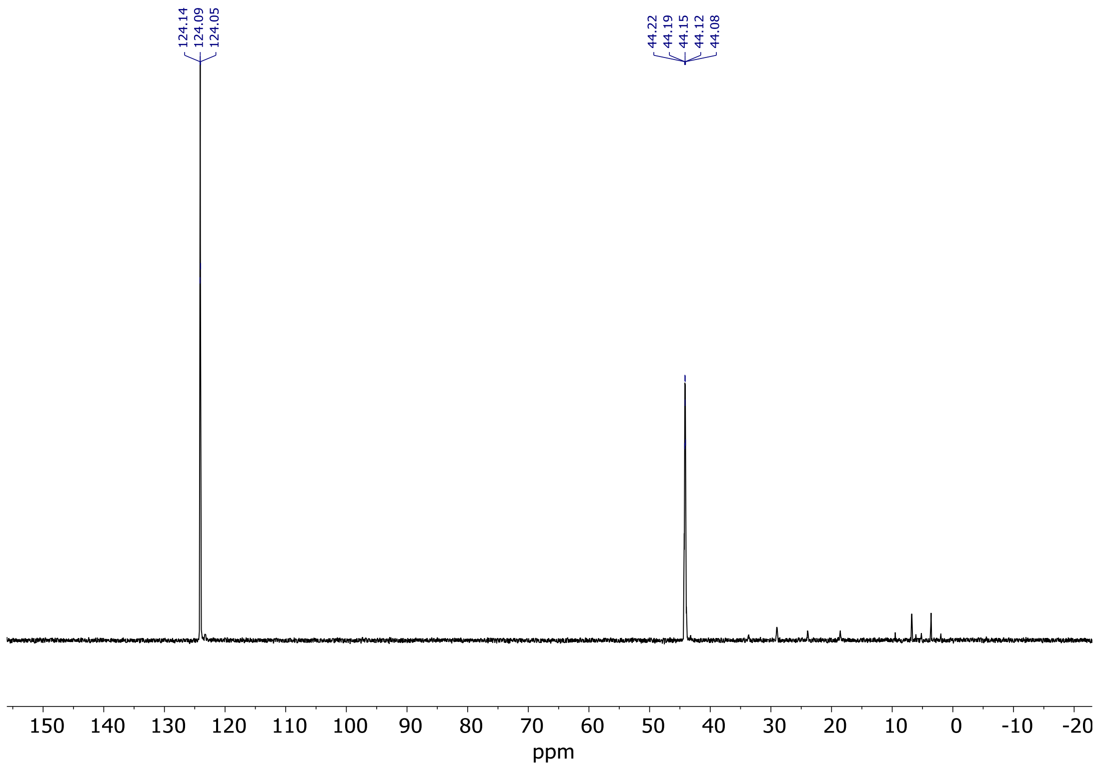

Figure S29c. ^{31}P NMR spectrum of **15**-*S* (202 MHz, 298 K, $CDCl_3$).

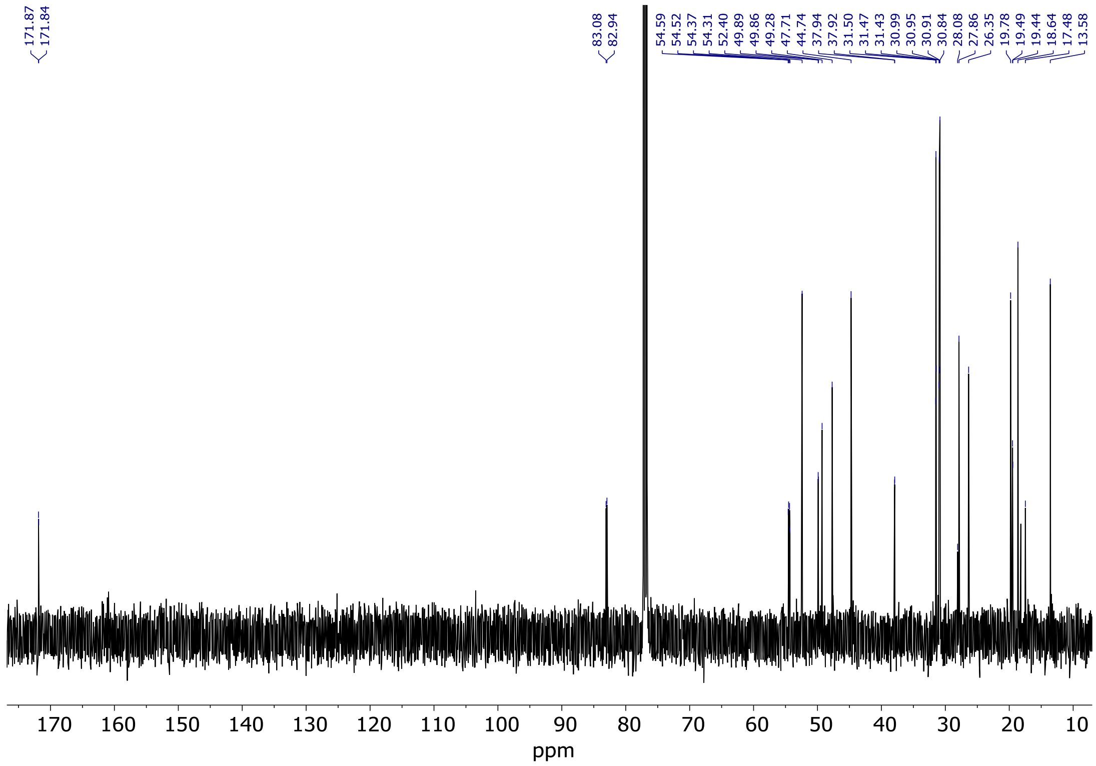

Figure S29d. $^{13}C\{^{1}H\}$ NMR spectrum of **15**-*S* (126 MHz, 298 K, $CDCl_3$).

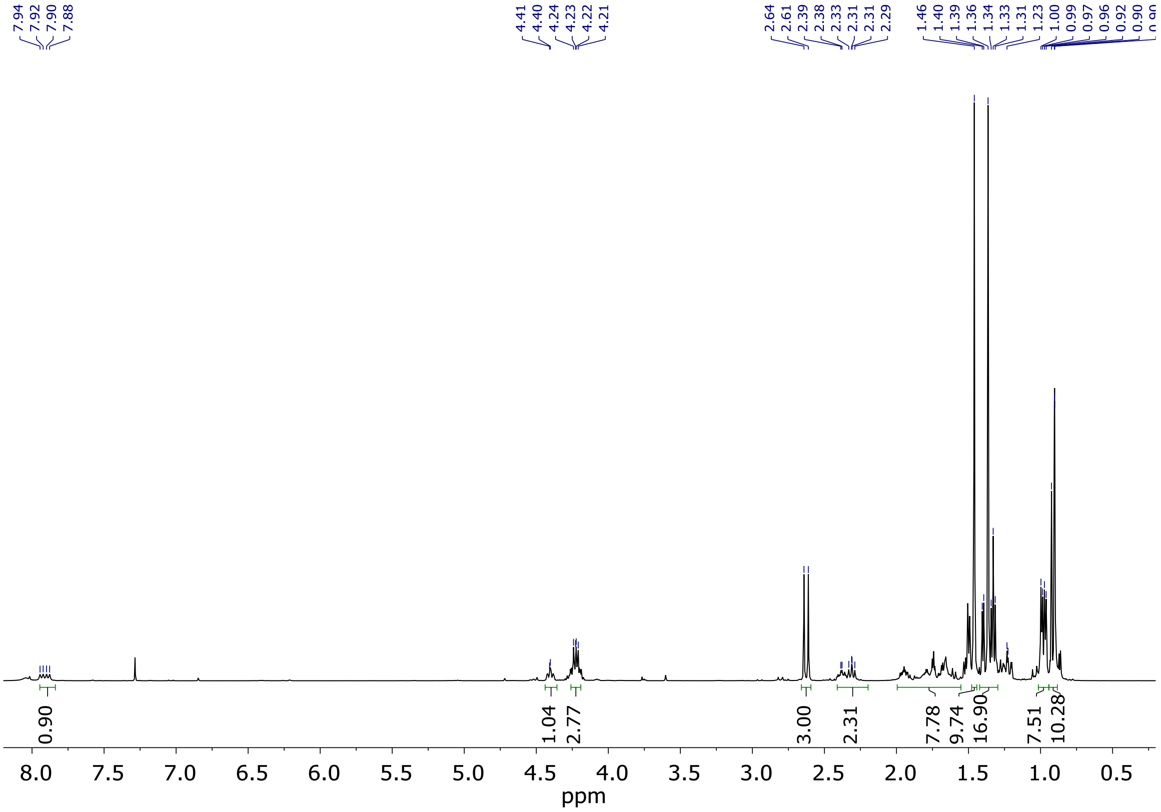

Figure S30a. ^{1}H NMR spectrum of **16**-*R* (500 MHz, 298 K, $CDCl_3$).

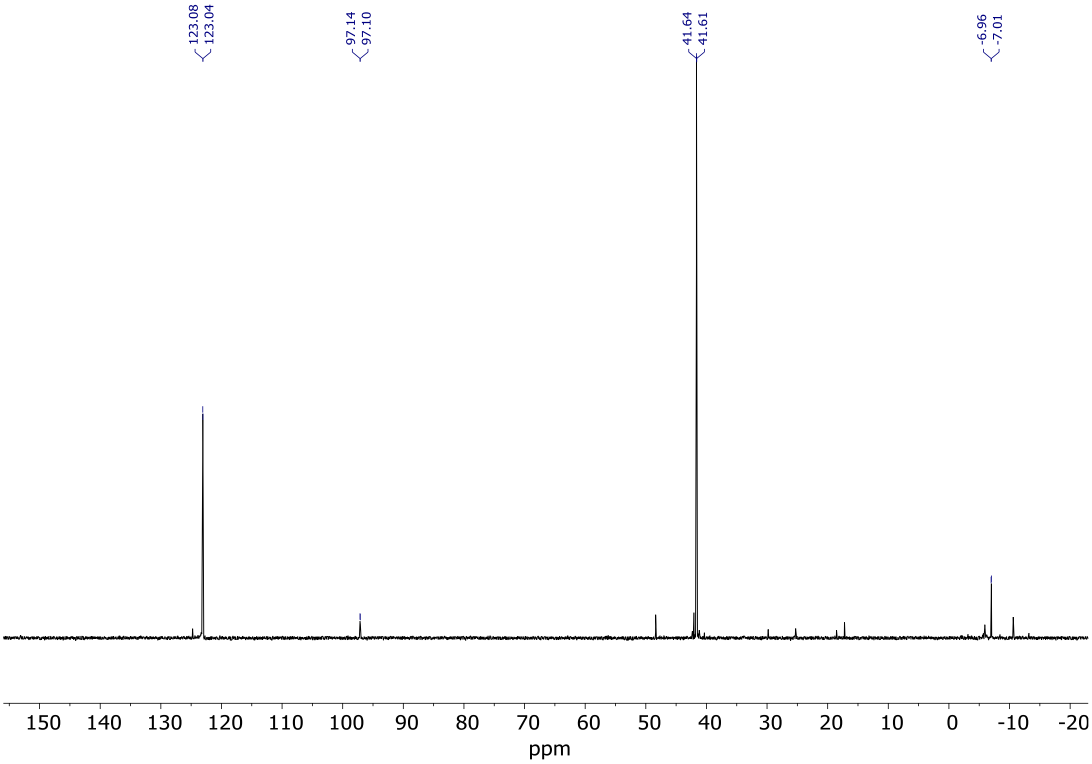

Figure S30b. $^{31}P\{^{1}H\}$ NMR spectrum of **16**-*R* (202 MHz, 298 K, $CDCl_3$). The peaks at δ 97 and −7 ppm correspond to an unidentified minor hydrolysis product.

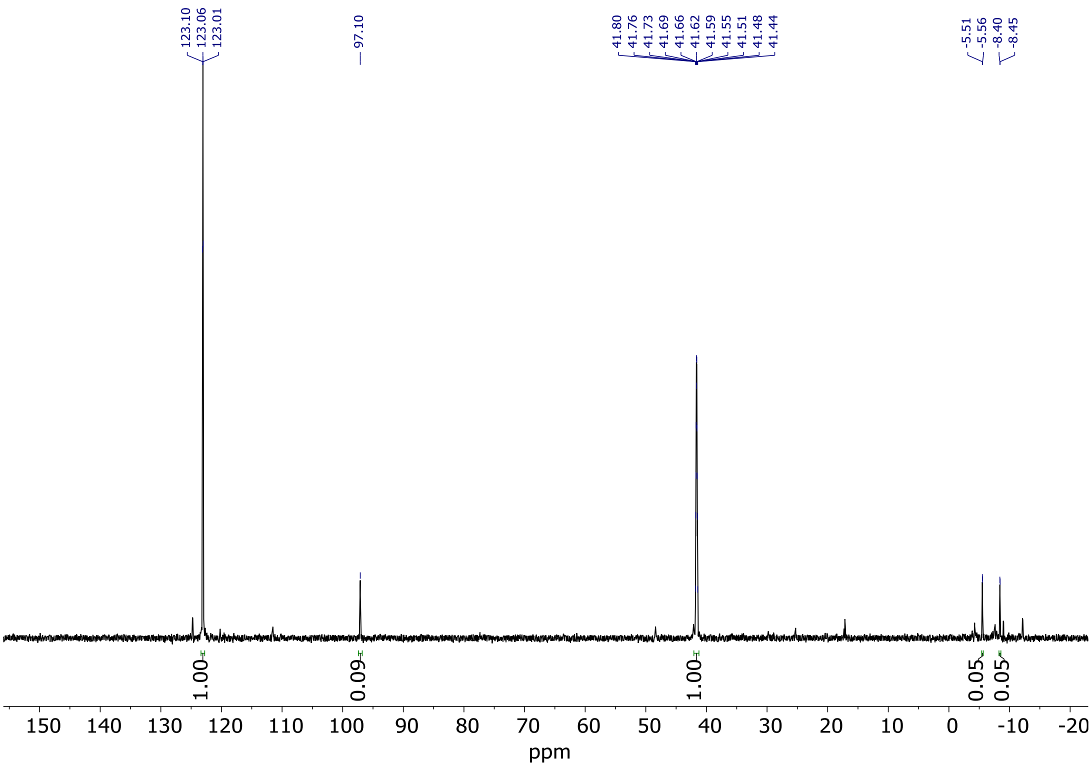

Figure S30c. ^{31}P NMR spectrum of **16**-*R* (202 MHz, 298 K, $CDCl_3$). Integration shows the minor hydrolysis product to be present in a <1:10 ratio to the main product.

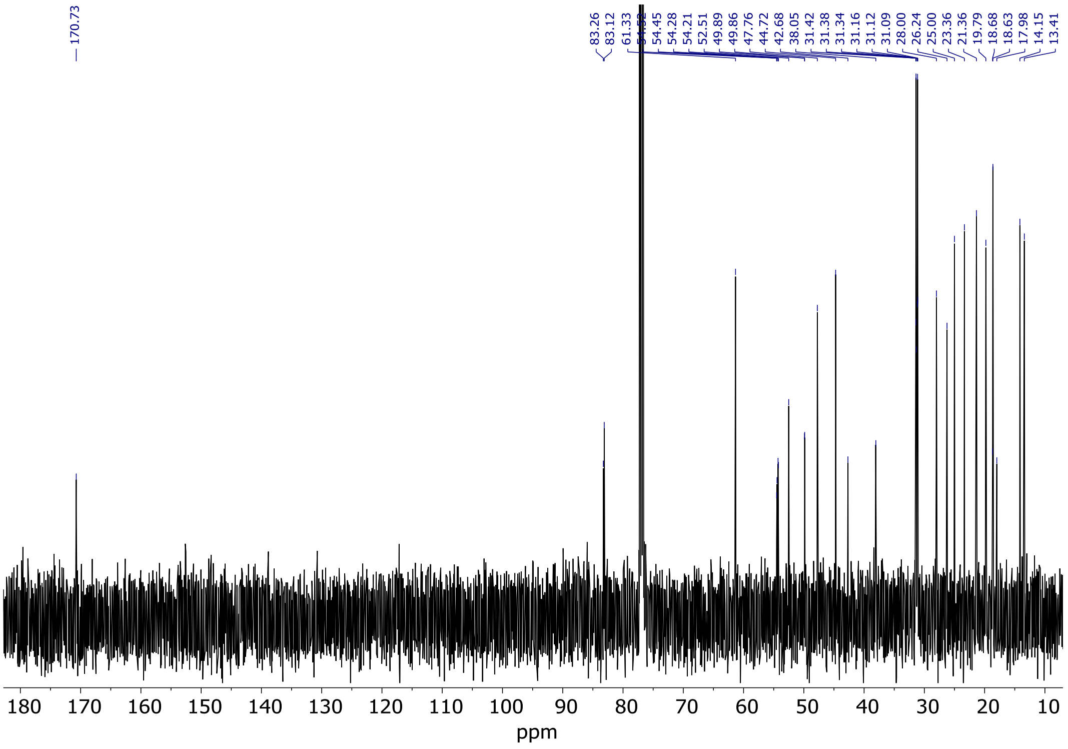

Figure S30d. $^{13}C\{^1H\}$ NMR spectrum of **16**-*R* (126 MHz, 298 K, $CDCl_3$).

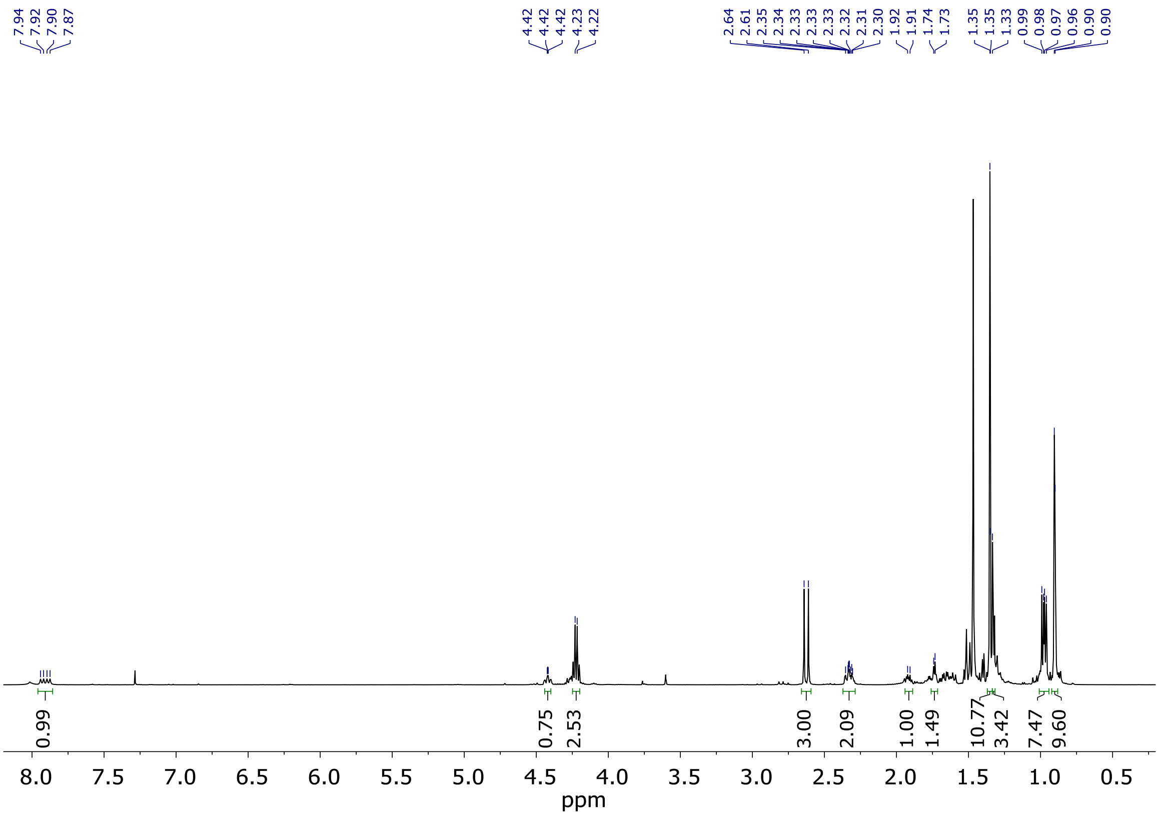

Figure S31a. ^{1}H NMR spectrum of **16**-*S* (500 MHz, 298 K, $CDCl_3$).

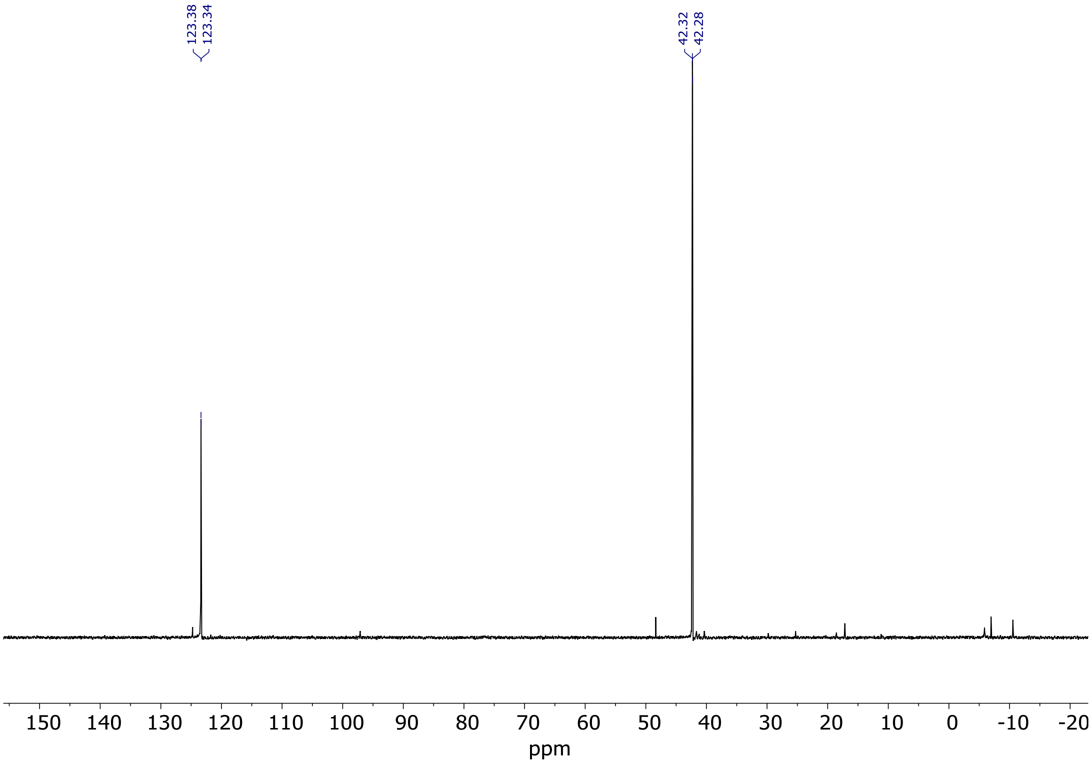

Figure S31b. $^{31}P\{^{1}H\}$ NMR spectrum of **16**-*S* (202 MHz, 298 K, $CDCl_3$).

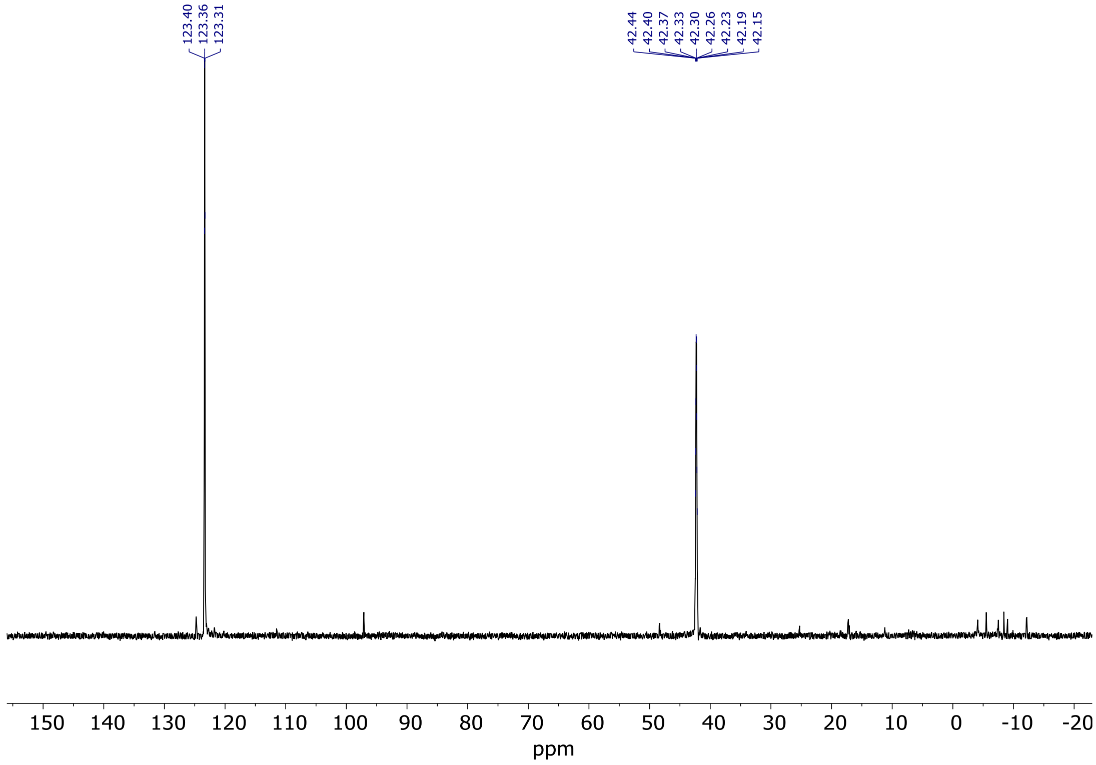

Figure S31c. ^{31}P NMR spectrum of **16**-*S* (202 MHz, 298 K, $CDCl_3$).

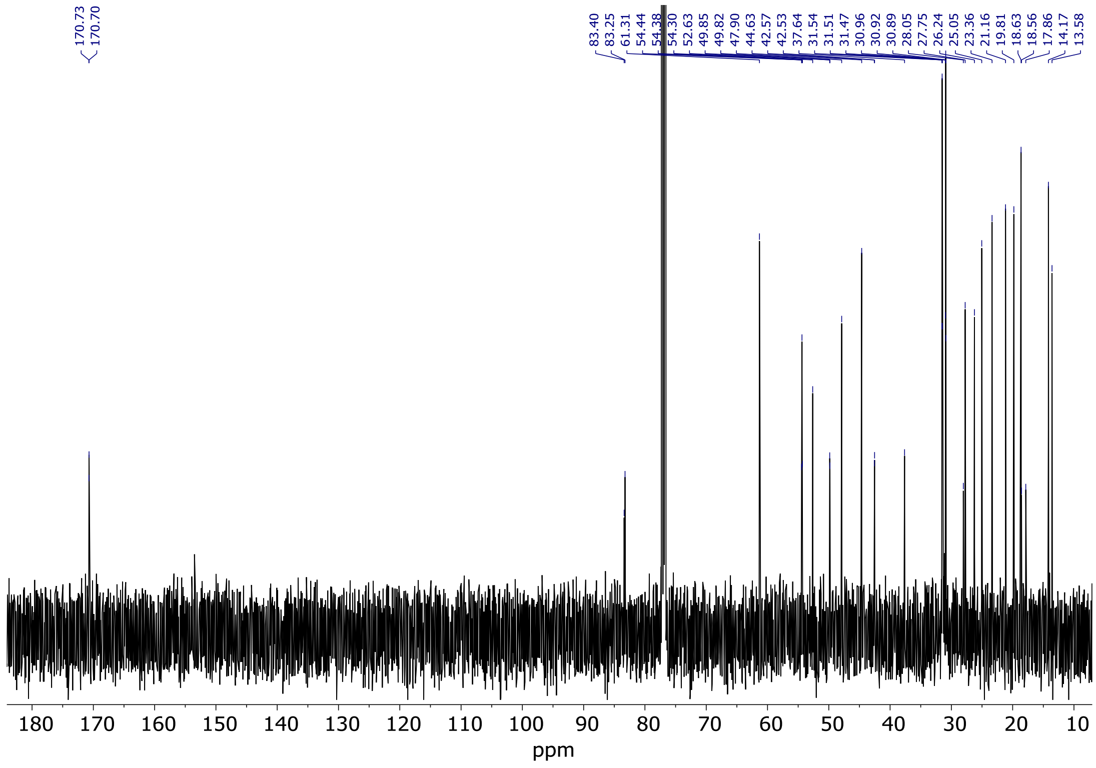

Figure S31d. $^{13}C\{^{1}H\}$ NMR spectrum of **16**-*S* (126 MHz, 298 K, $CDCl_3$).

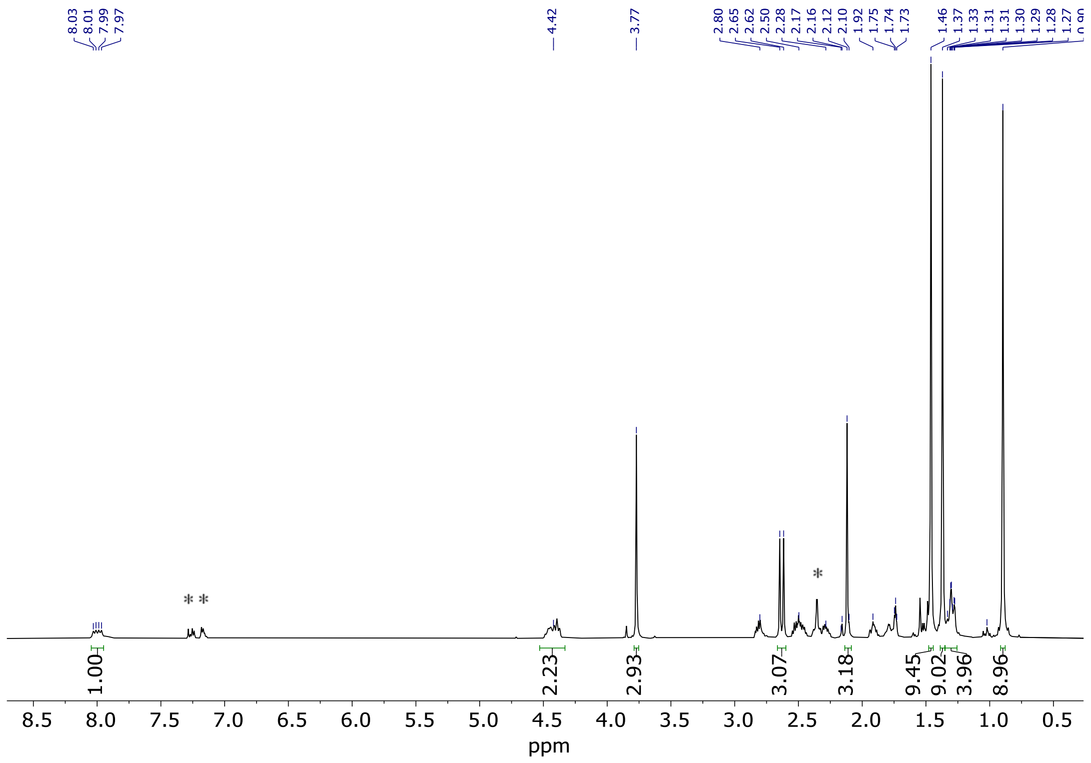

Figure S32a. ^{1}H NMR spectrum of **17**-*R* (500 MHz, 298 K, $CDCl_3$). Starred minor peaks are from remnant toluene (*δ* 7.25, 7.17 and 2.36 ppm).

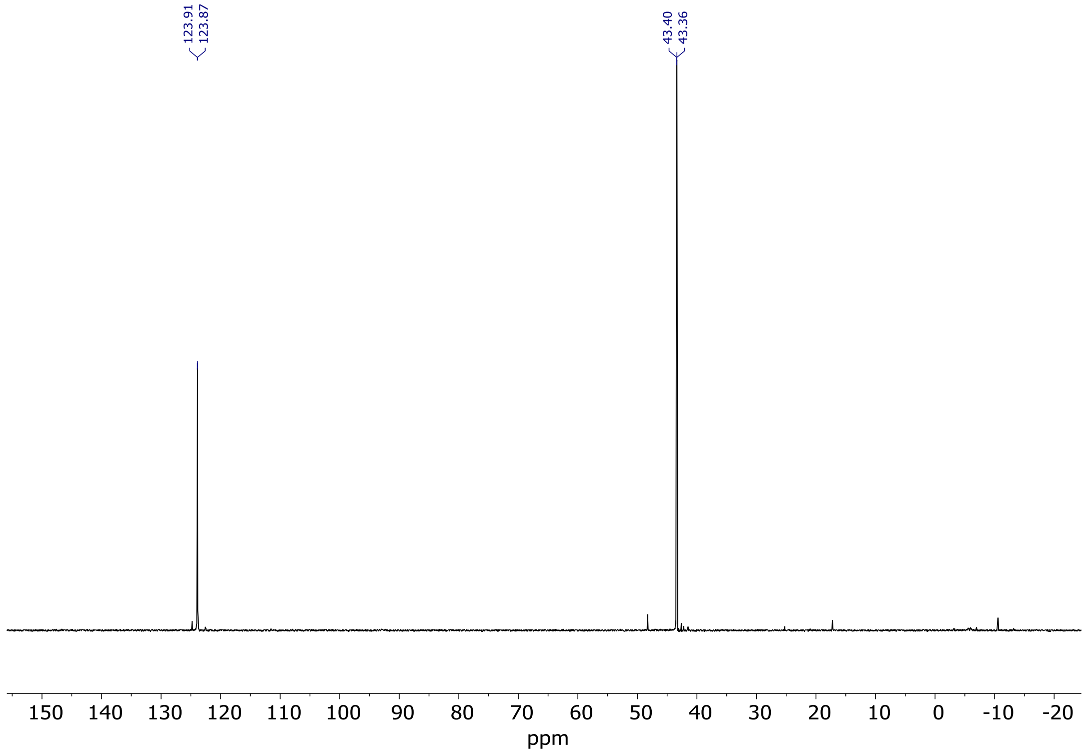

Figure S32b. $^{31}P\{^{1}H\}$ NMR spectrum of **17**-*R* (202 MHz, 298 K, $CDCl_3$).

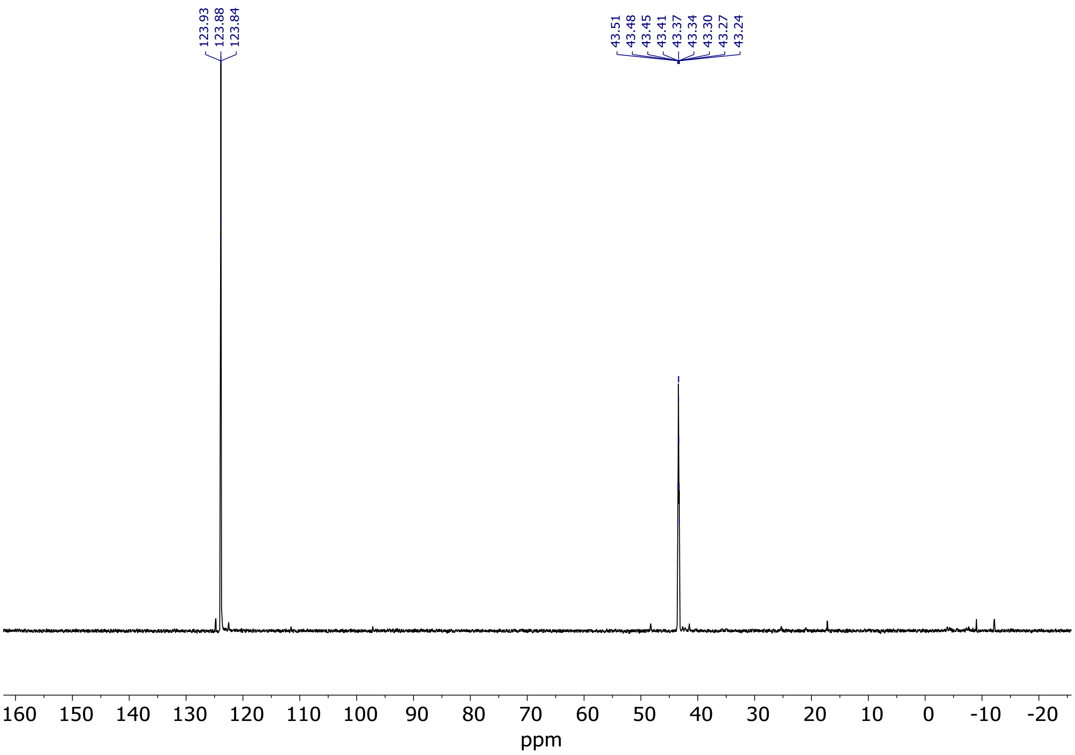

Figure S32c. ^{31}P NMR spectrum of **17**-*R* (202 MHz, 298 K, $CDCl_3$).

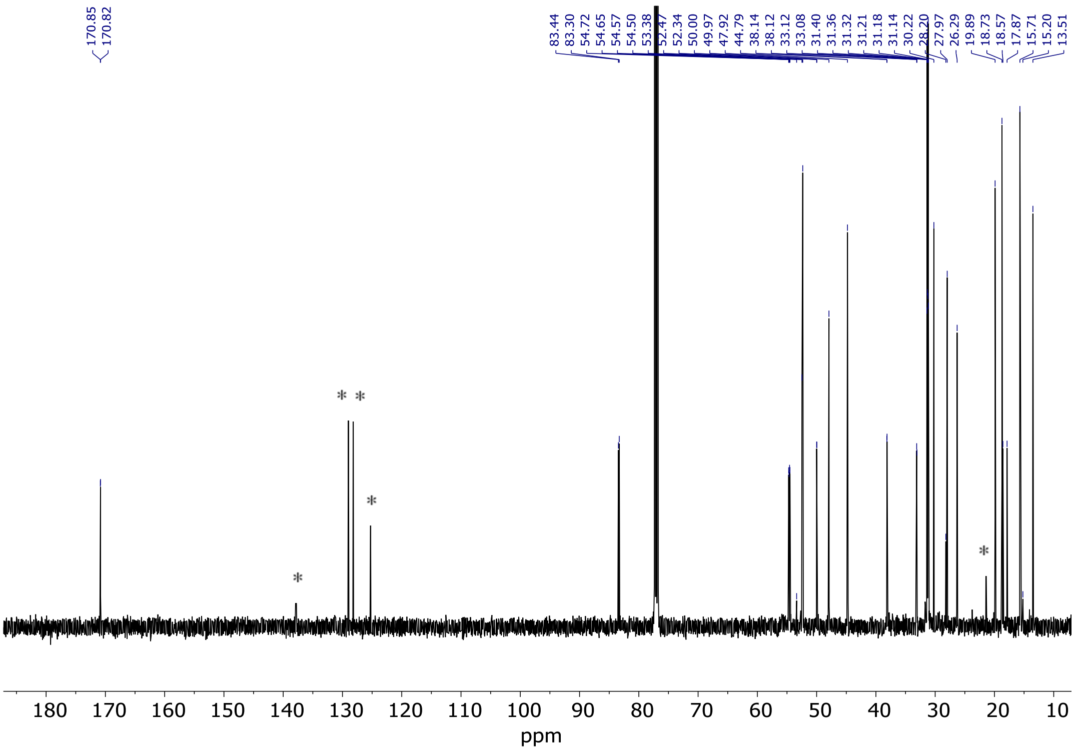

Figure S32d. $^{13}C\{^1H\}$ NMR spectrum of **17**-*R* (126 MHz, 298 K, $CDCl_3$). Starred peaks are from remnant toluene (δ 137.9, 129.1, 128.3, 125.3 and 21.5 ppm).

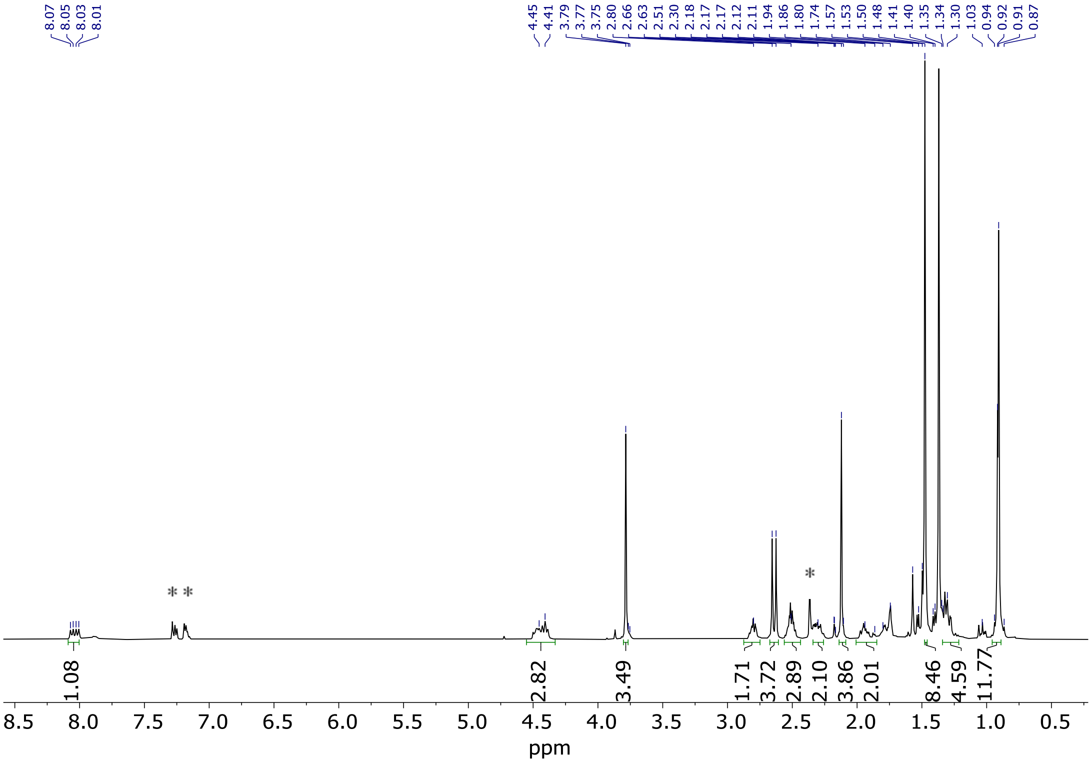

Figure S33a. ^{1}H NMR spectrum of **17**-*S* (500 MHz, 298 K, $CDCl_3$). Starred minor peaks are from remnant toluene (*δ* 7.25, 7.17 and 2.36 ppm).

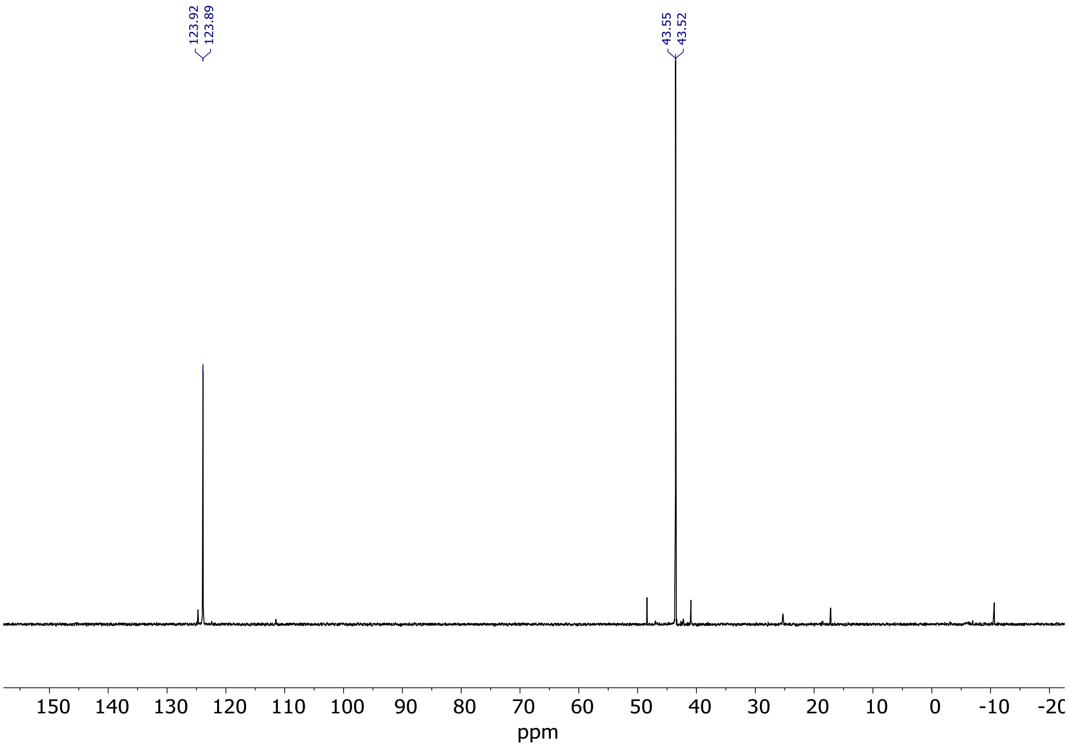

Figure S33b. ^{31}P{^{1}H} NMR spectrum of **17**-*S* (202 MHz, 298 K, $CDCl_3$).

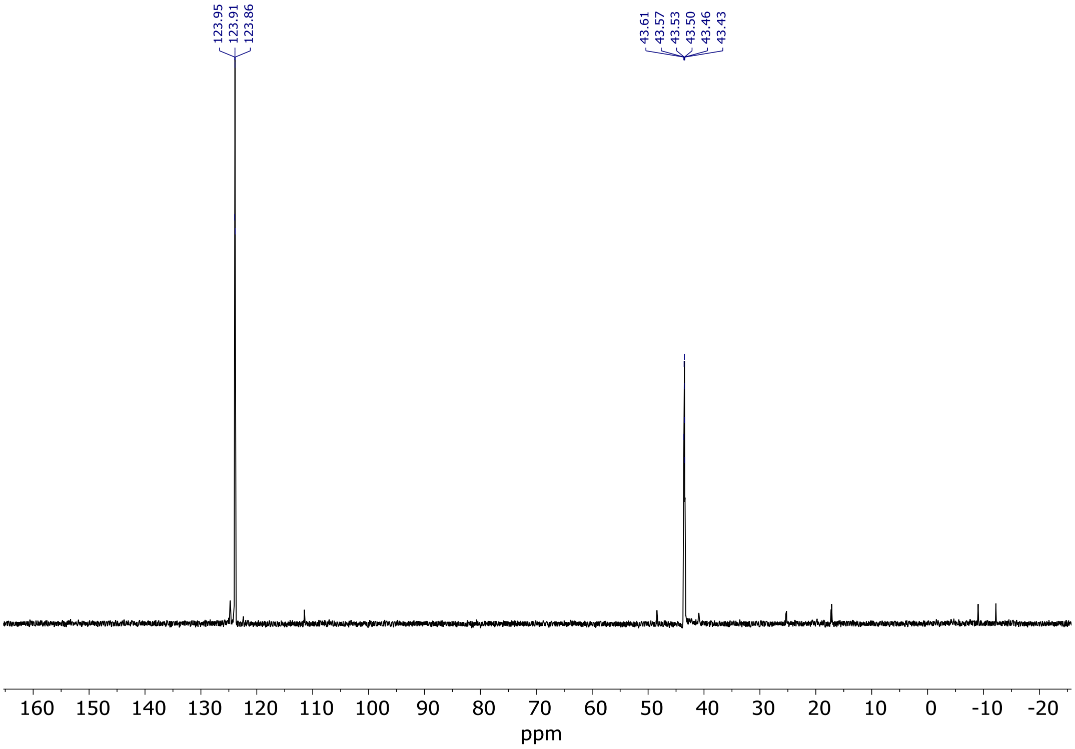

Figure S33c. ^{31}P NMR spectrum of **17**-*S* (202 MHz, 298 K, $CDCl_3$).

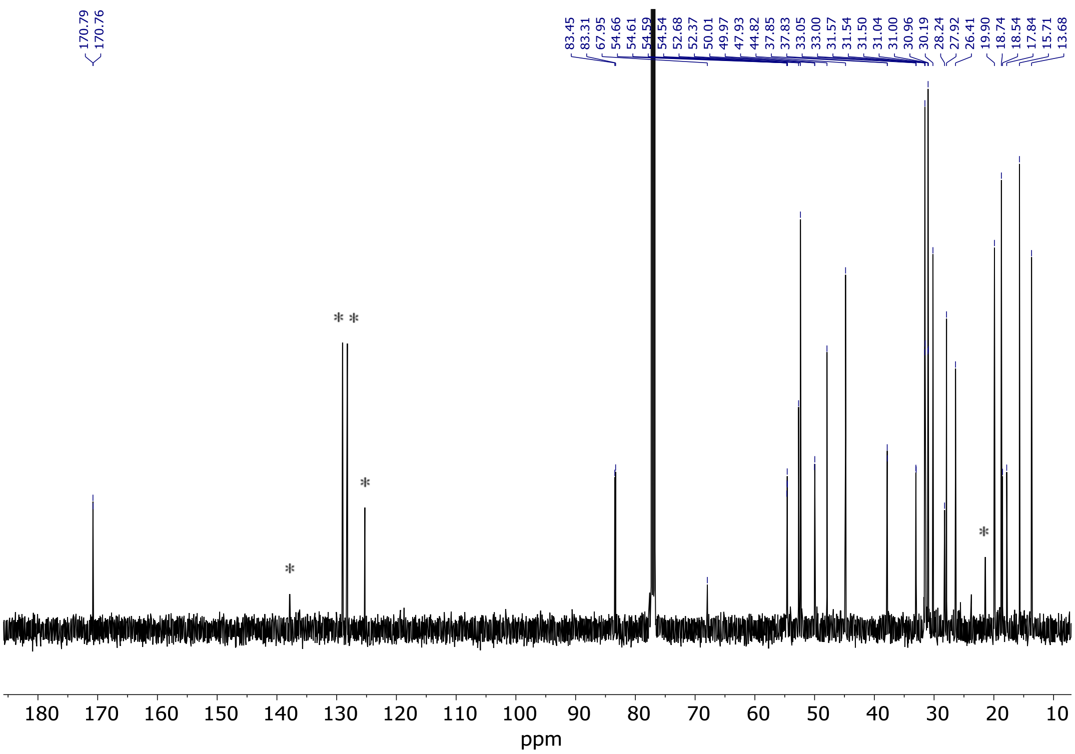

Figure S33d. $^{13}C\{^{1}H\}$ NMR spectrum of **17**-*S* (126 MHz, 298 K, $CDCl_3$). Starred peaks are from remnant toluene (δ 137.9, 129.1, 128.3, 125.3 and 21.5 ppm).

***In situ* studies**

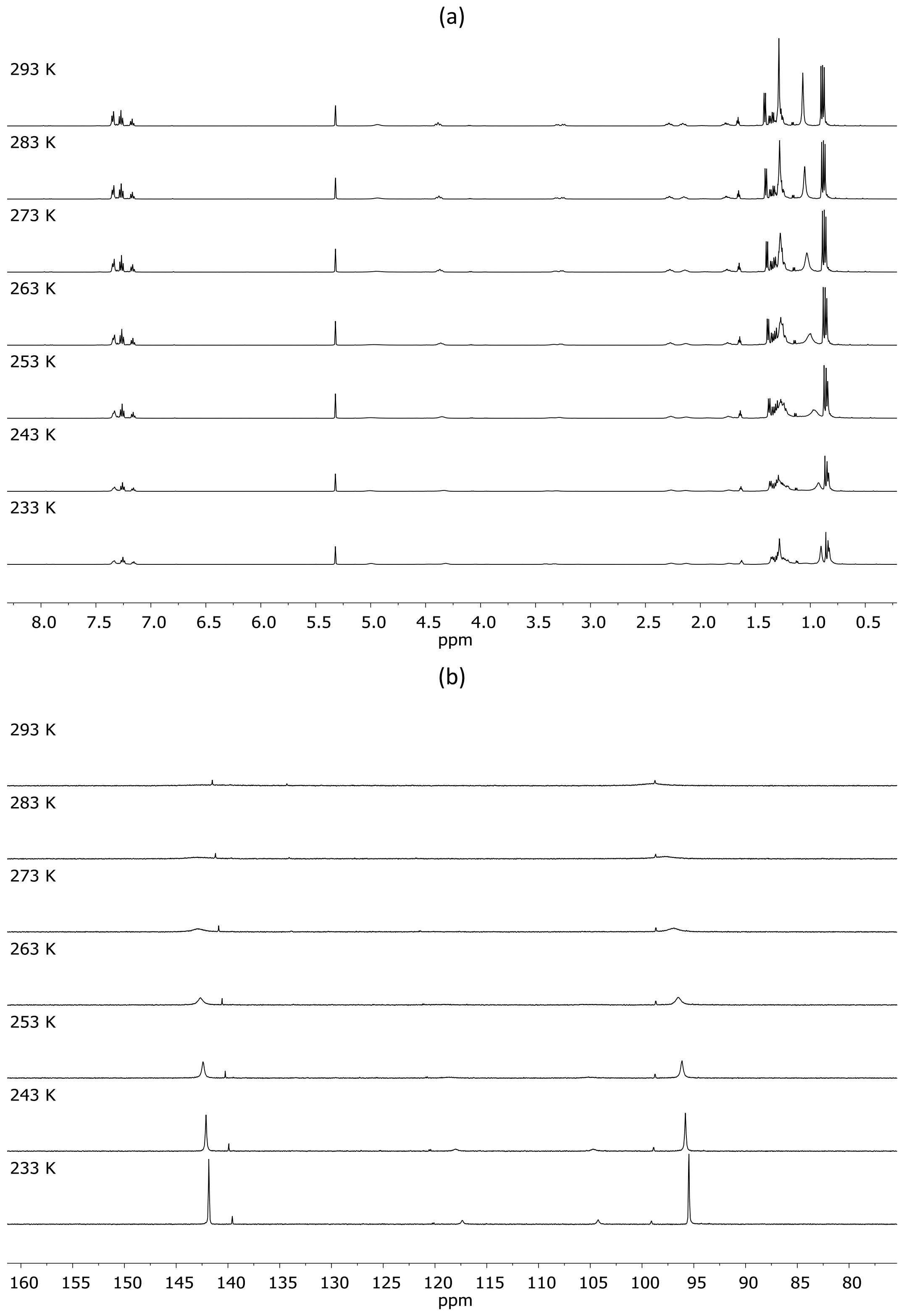

Figure S34. Variable temperature (a) ^{1}H NMR spectra and (b) $^{31}P\{^{1}H\}$ NMR spectra of **4**-*R* in CD_2Cl_2 at the temperature indicated.

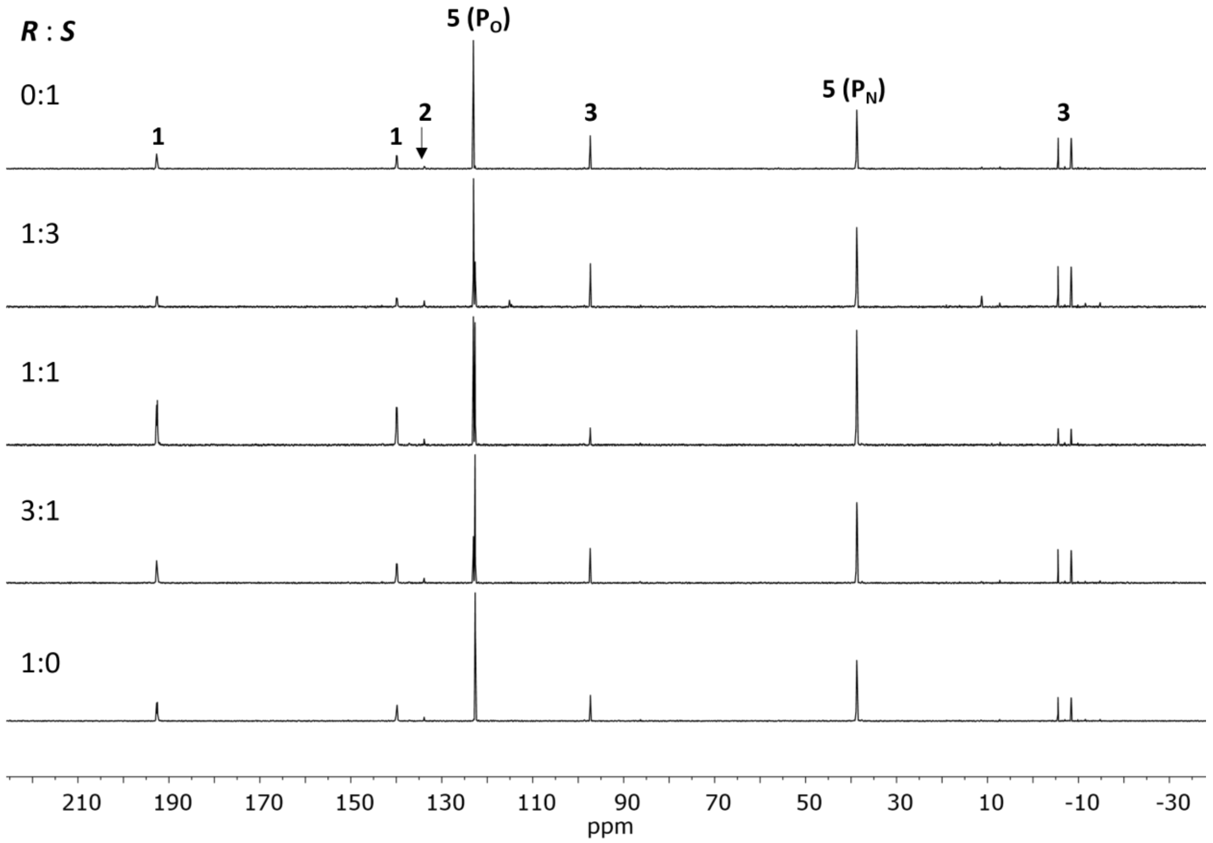

Figure S35. *In situ* ^{31}P NMR spectra of **5**-*R*/**5**-*S* (202 MHz, 298 K, $CDCl_3$).

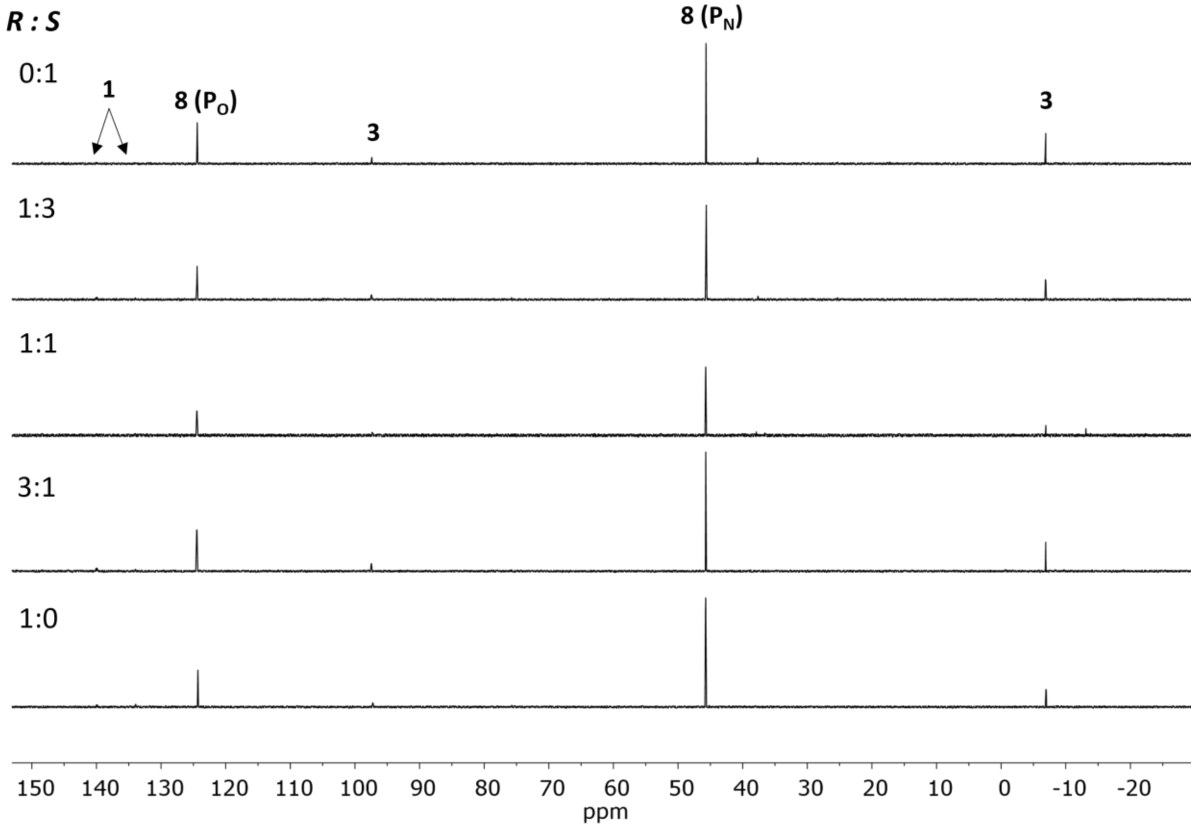

Figure S36. *In situ* ^{31}P{^{1}H} NMR spectra of **8**-*R*/**8**-*S* (202 MHz, 298 K, $CDCl_3$).

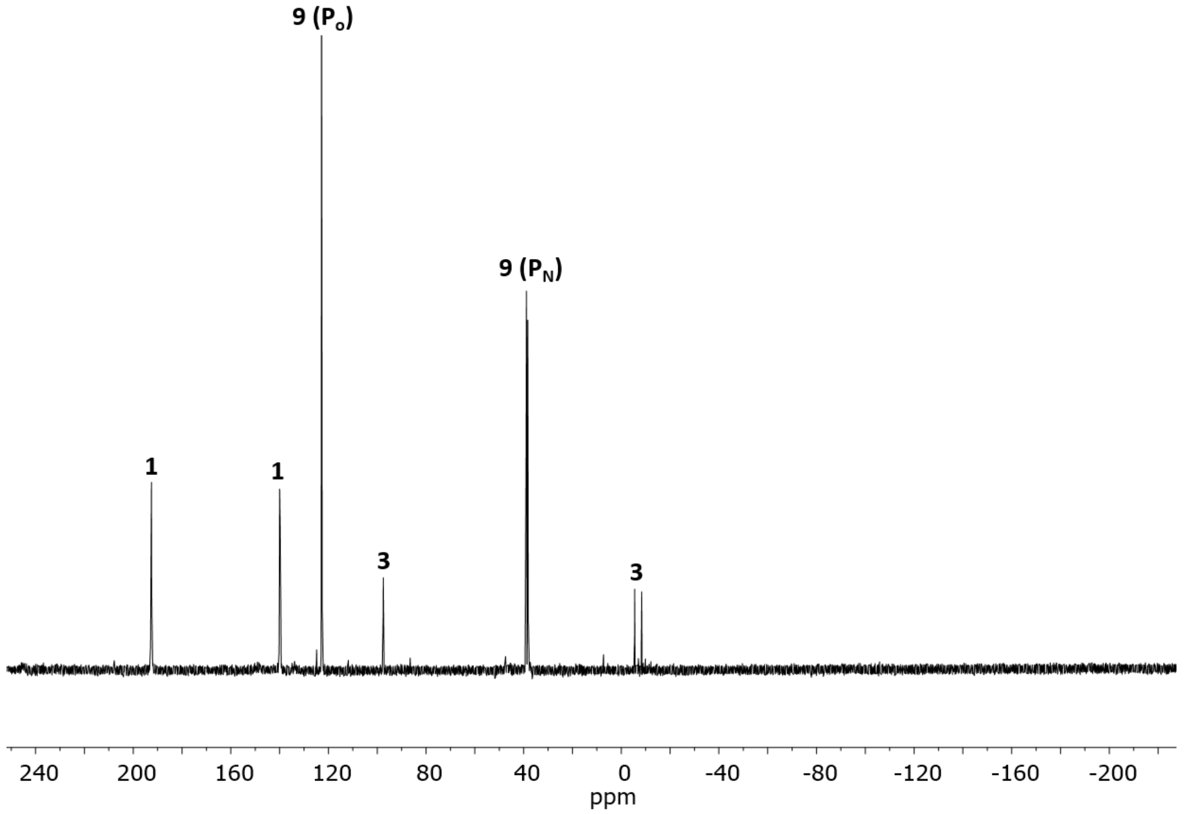

Figure S37. *In situ* ^{31}P NMR spectrum of **9**-*R*/**9**-*S* (202 MHz, 298 K, $CDCl_3$).

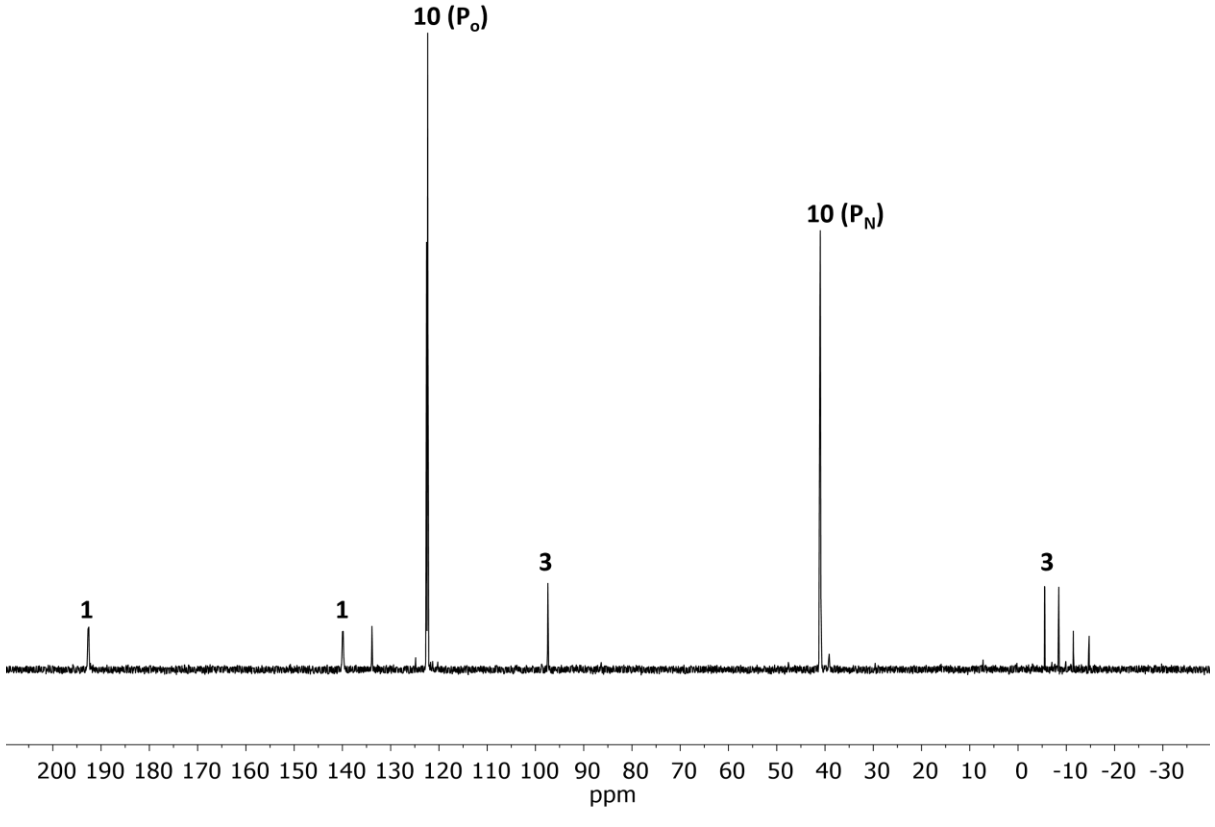

Figure S38. *In situ* ^{31}P NMR spectrum of **10**-*R*/**10**-*S* (202 MHz, 298 K, $CDCl_3$). Peaks at δ 145 and −15 ppm

correspond to an unidentified hydrolysis product.

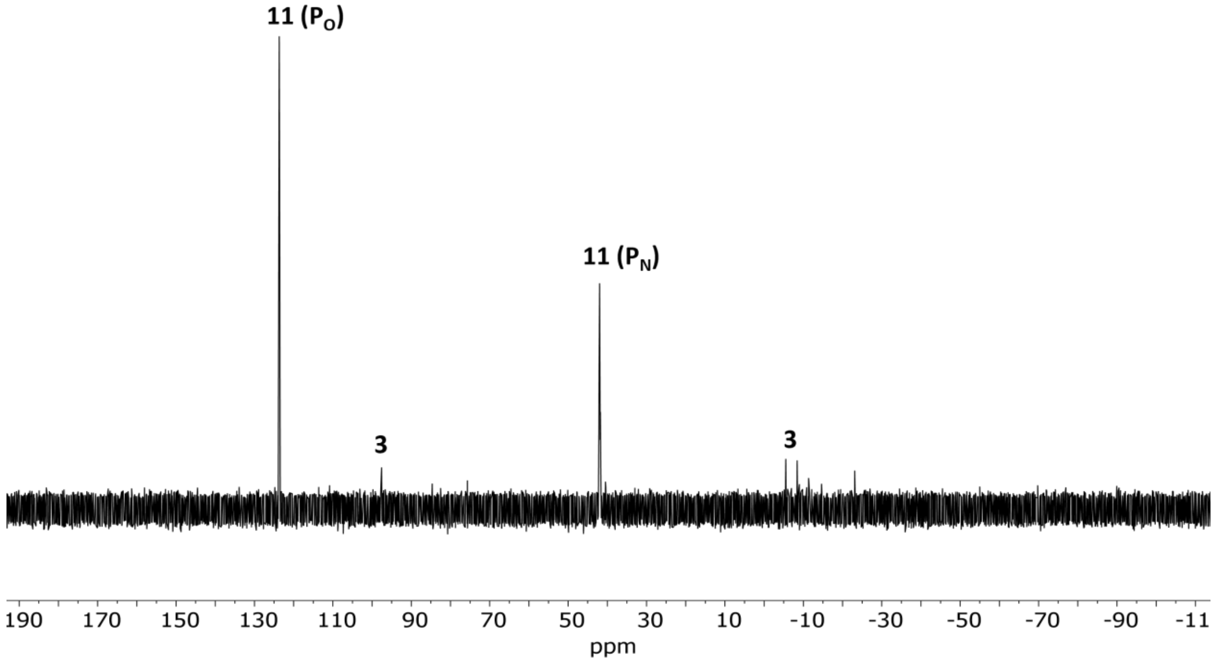

Figure S39. *In situ* ^{31}P NMR spectrum of **11**-*R*/**11**-*S* (202 MHz, 298 K, $CDCl_3$).

3. Substrate Scope (see Table 2 in the text)

Expansions of $^{31}P\{^{1}H\}$ NMR spectra for fully characterised substrates

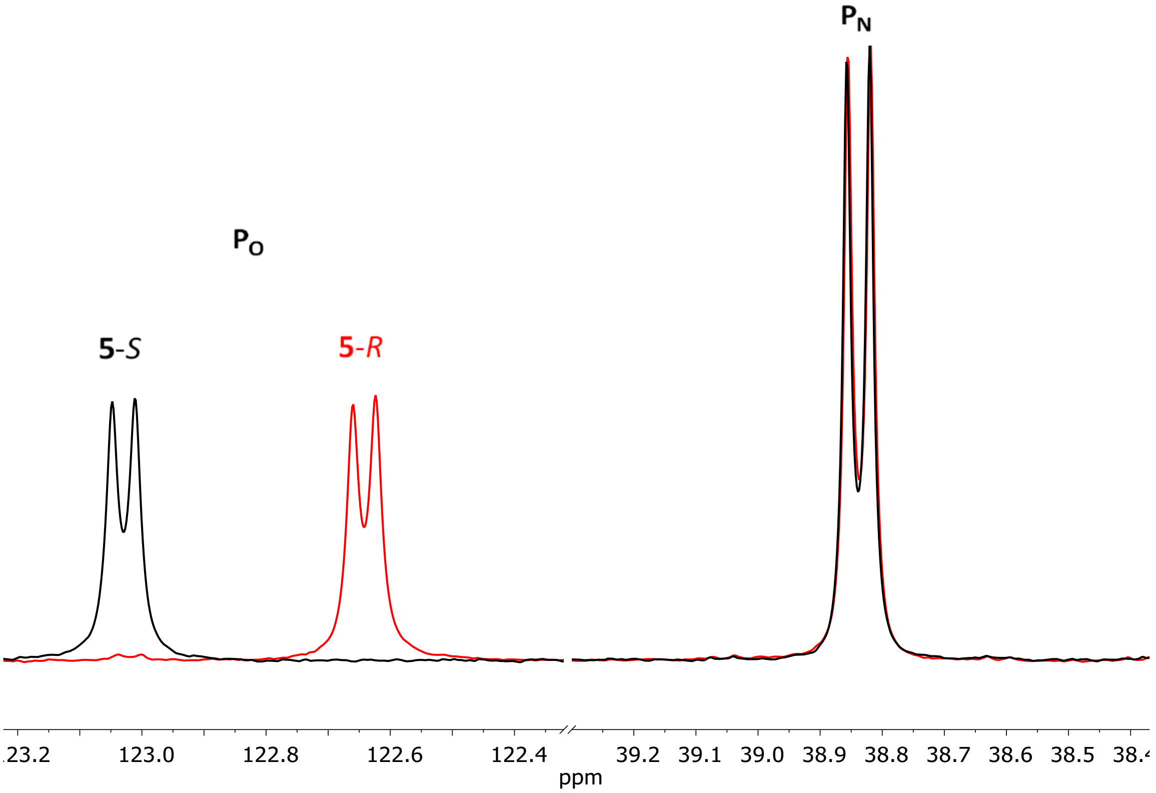

Figure S40. Overlaid expansions of the $^{31}P\{^{1}H\}$ NMR spectra of **5**-*R* & **5**-*S* (202 MHz, 298 K, $CDCl_3$).

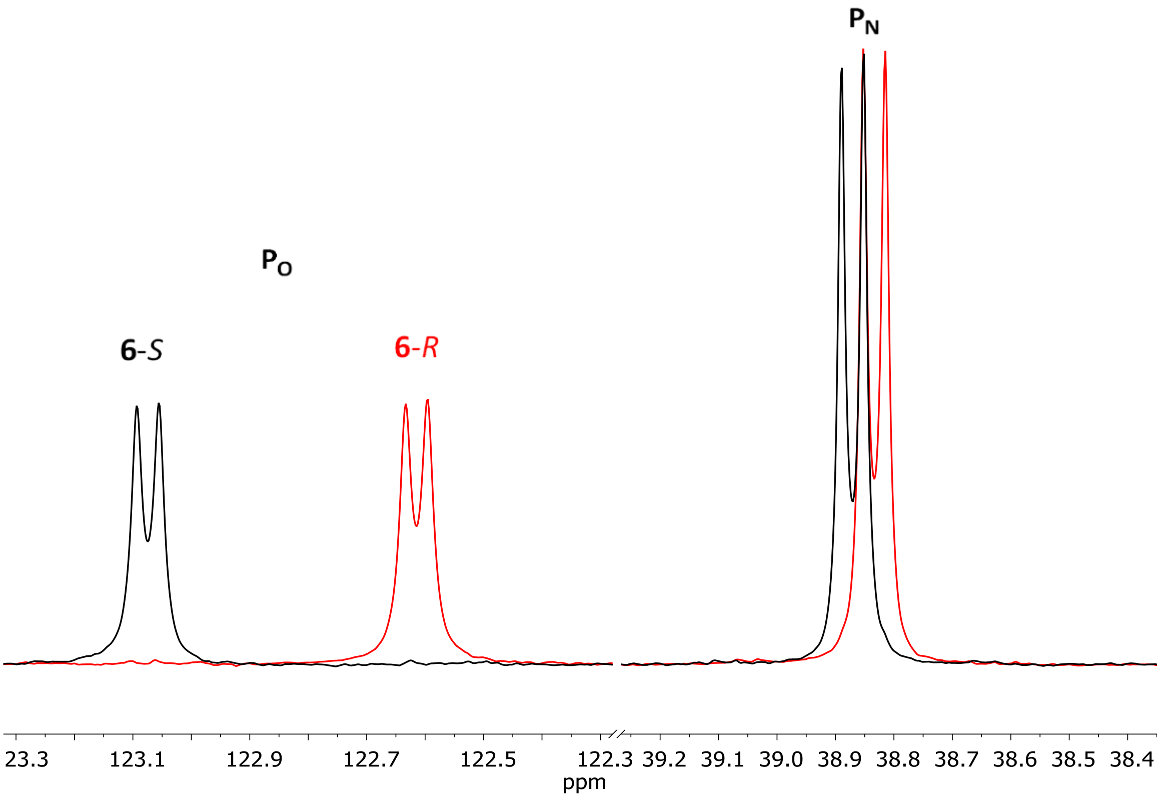

Figure S41. Overlaid expansions of the $^{31}P\{^{1}H\}$ NMR spectra of **6**-*R* & **6**-*S* (202 MHz, 298 K, $CDCl_3$).

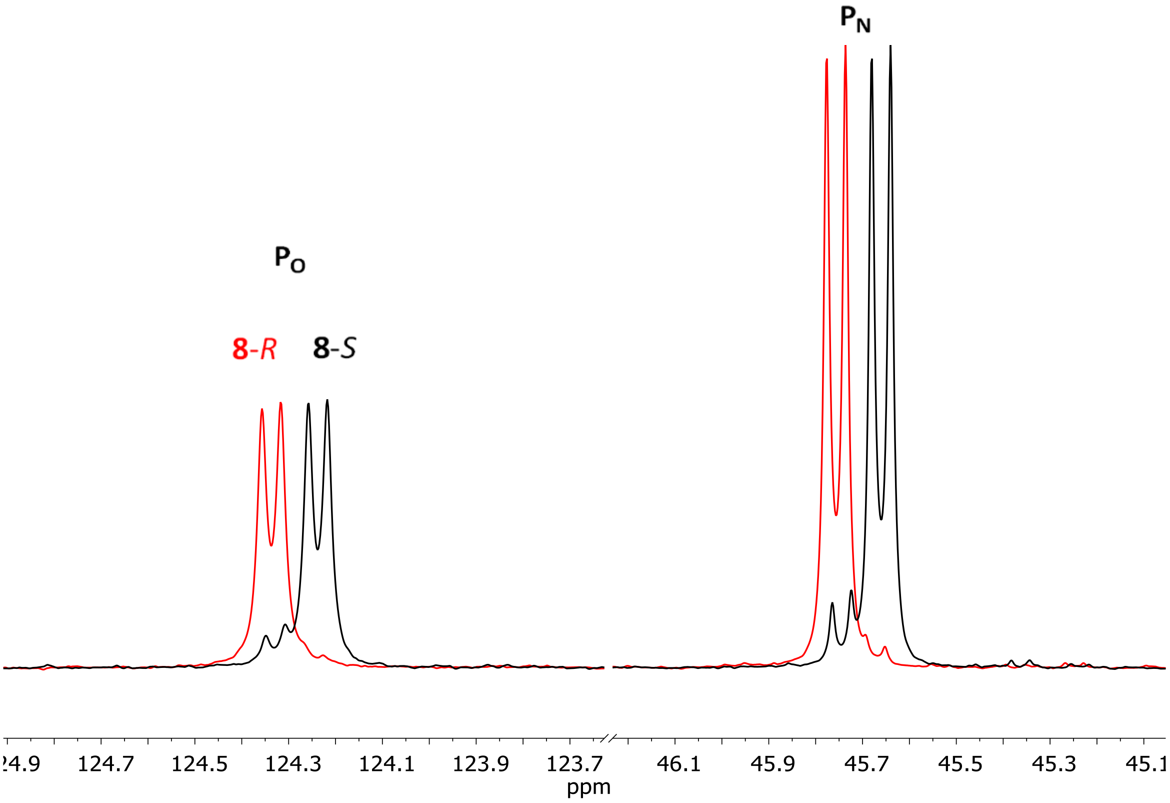

Figure S42. Overlaid expansions of the $^{31}P\{^1H\}$ NMR spectra of **8**-*R* & **8**-*S* (202 MHz, 298 K, $CDCl_3$).

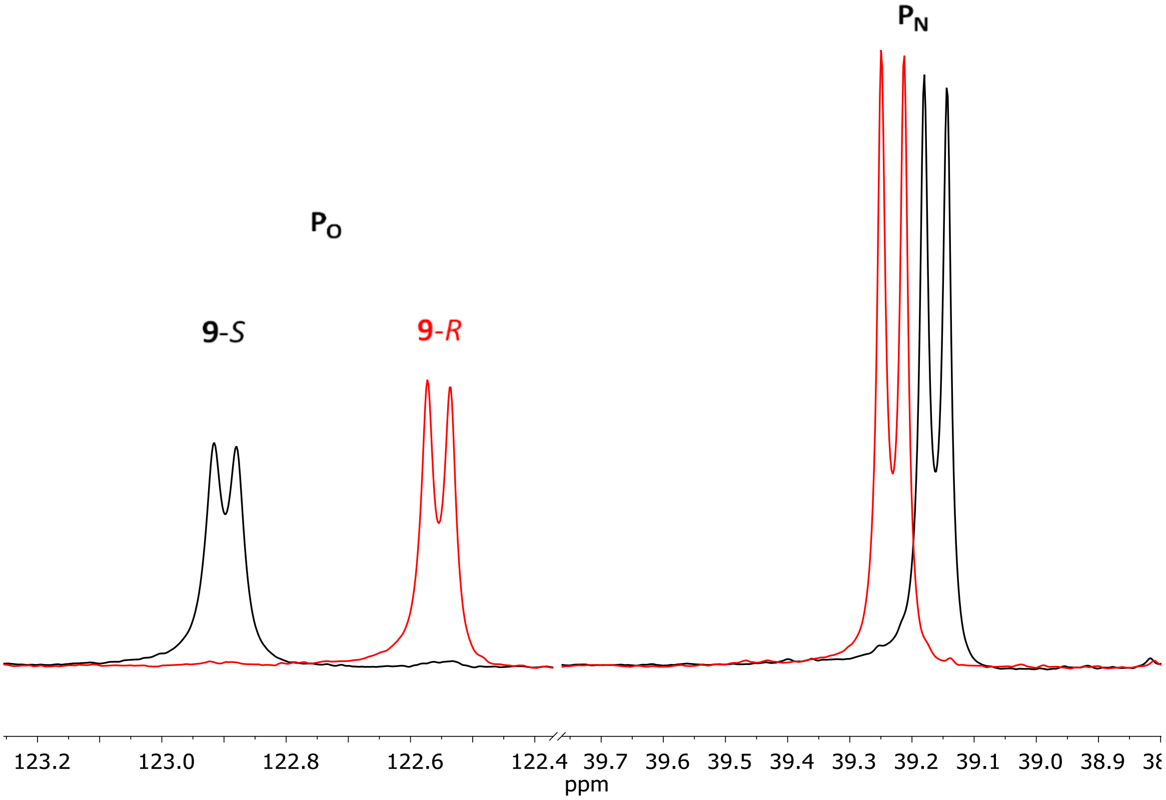

Figure S43. Overlaid expansions of the $^{31}P\{^1H\}$ NMR spectra of **9**-*R* & **9**-*S* (202 MHz, 298 K, $CDCl_3$).

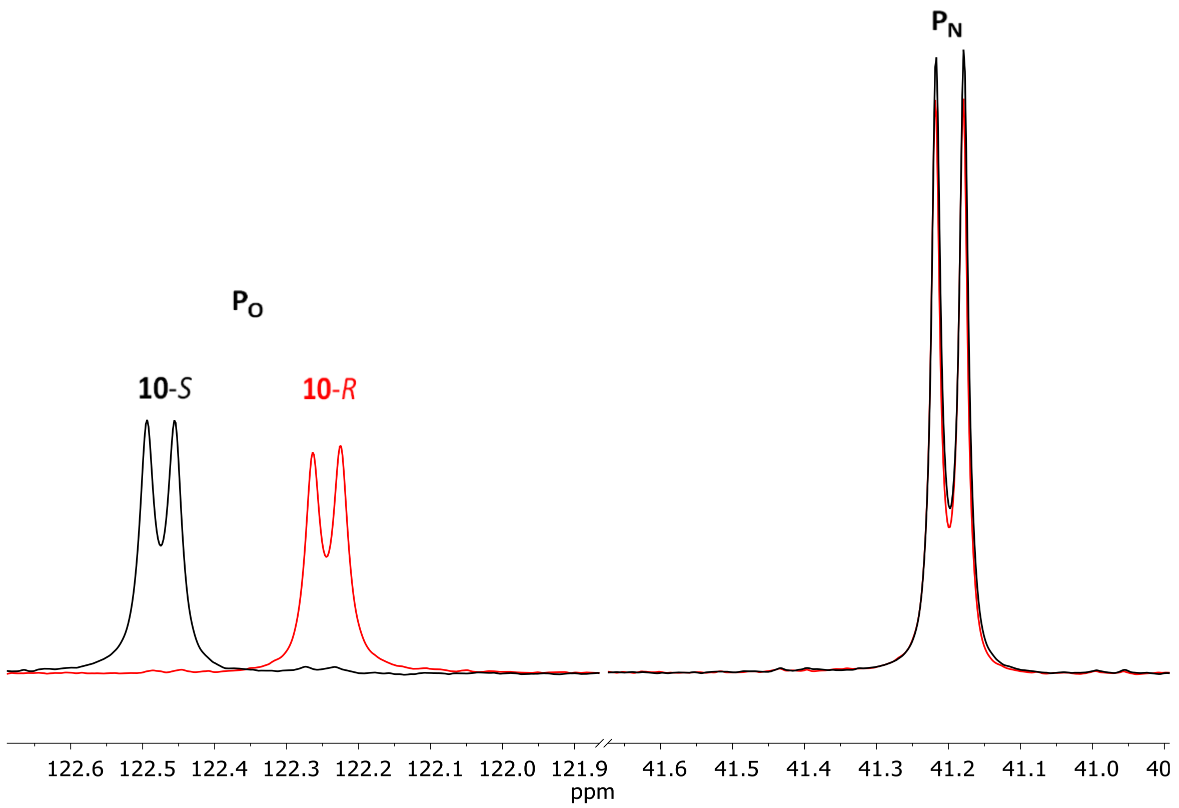

Figure S44. Overlaid expansions of the ^{31}P{^{1}H} NMR spectra of **10**-*R* & **10**-*S* (202 MHz, 298 K, $CDCl_3$).

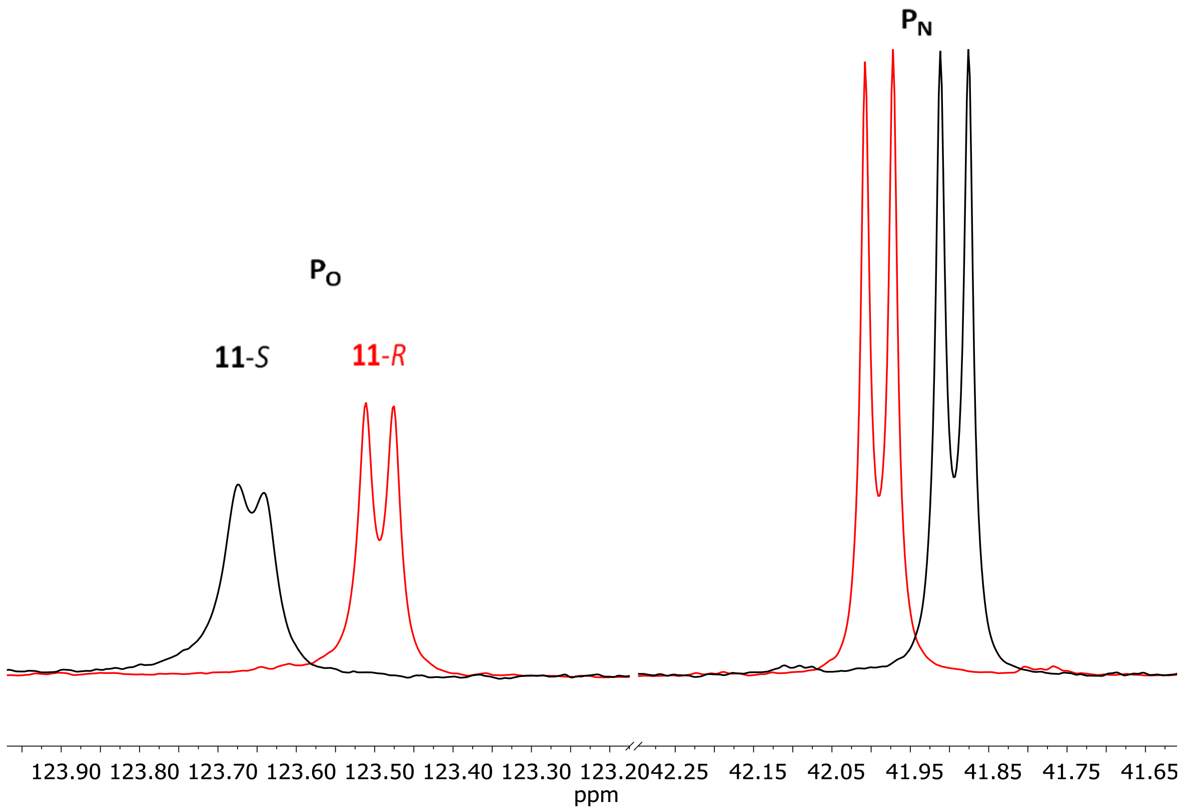

Figure S45. Overlaid expansions of the ^{31}P{^{1}H} NMR spectra of **11**-*R* & **11**-*S* (202 MHz, 298 K, $CDCl_3$).

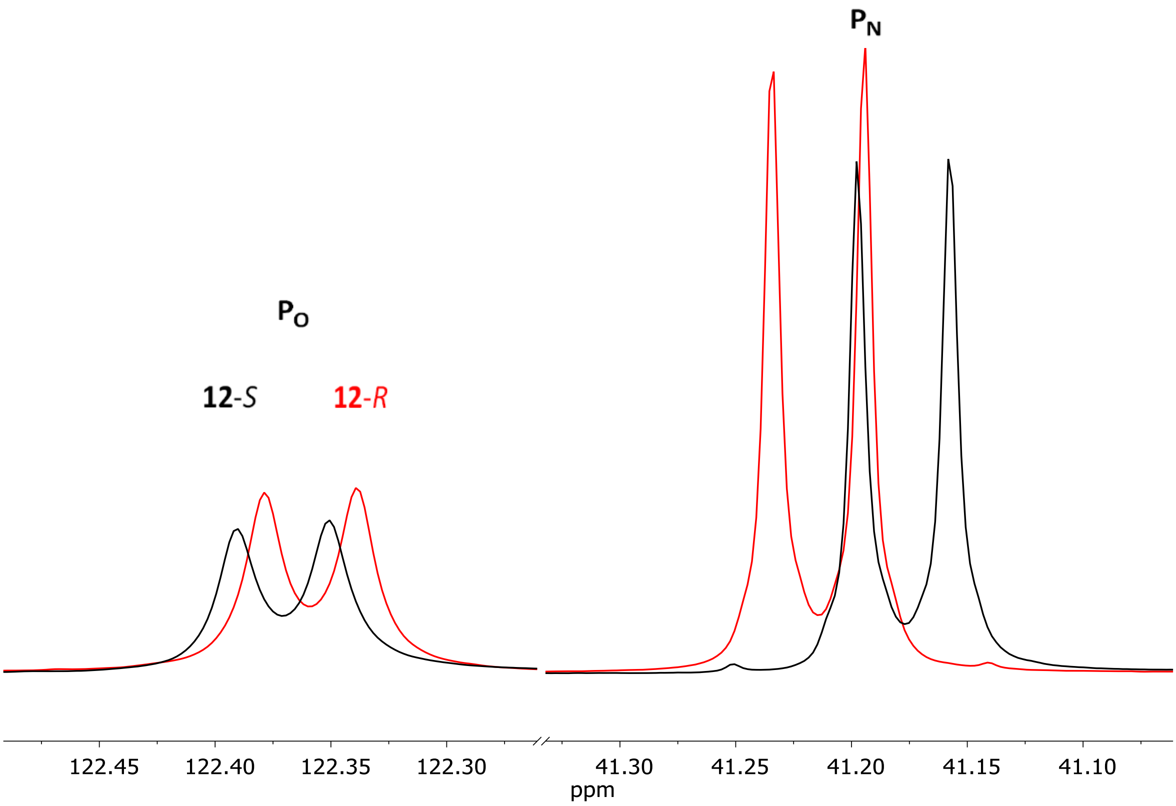

Figure S46. Overlaid expansions of the $^{31}P\{^{1}H\}$ NMR spectra of **12**-*R* & **12**-*S* (202 MHz, 298 K, $CDCl_3$).

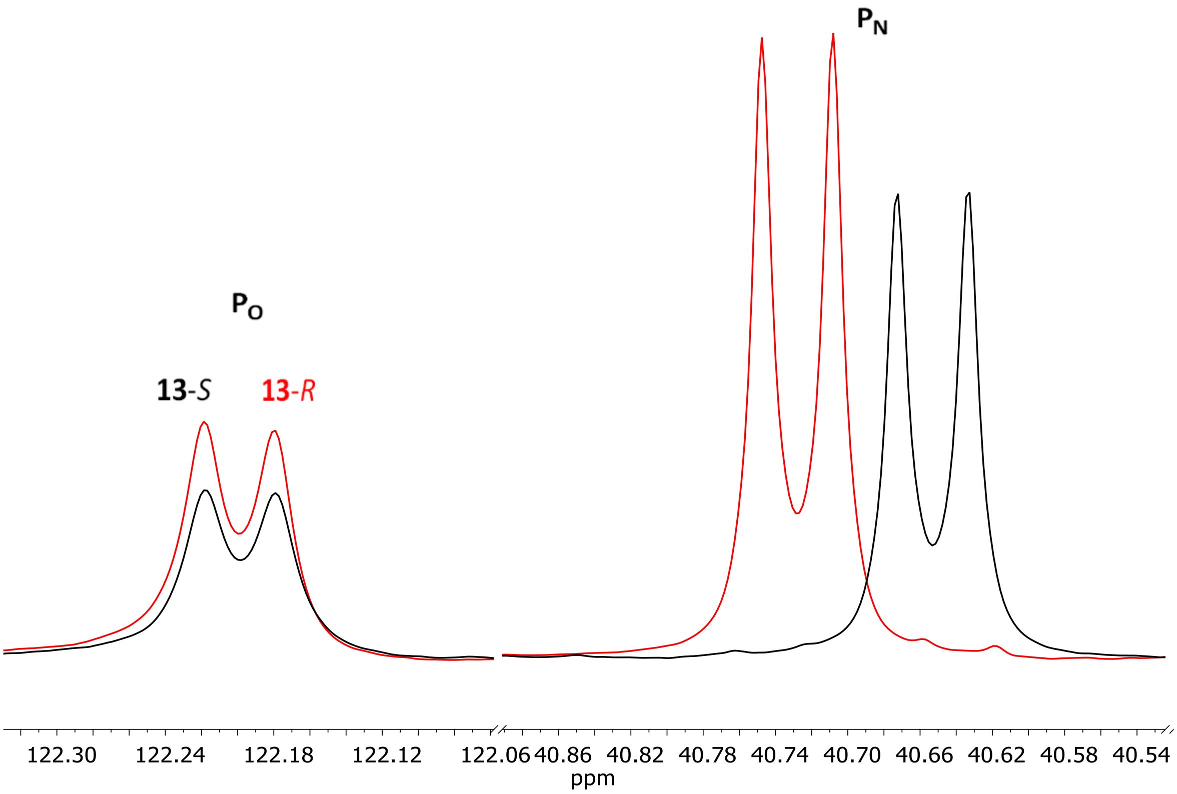

Figure S47. Overlaid expansions of the $^{31}P\{^{1}H\}$ NMR spectra of **13**-*R* & **13**-*S* (202 MHz, 298 K, $CDCl_3$).

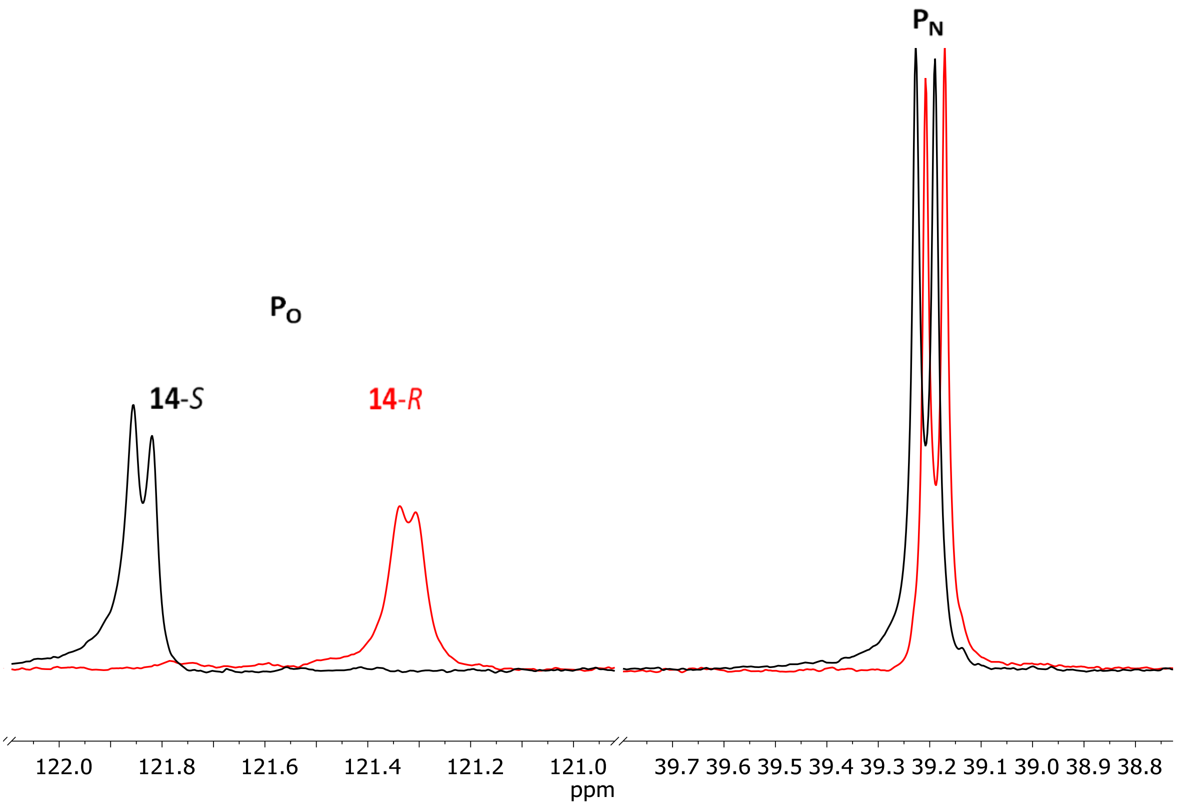

Figure S48. Overlaid expansions of the $^{31}P\{^{1}H\}$ NMR spectra of **14**-*R* & **14**-*S* (202 MHz, 298 K, $CDCl_3$).

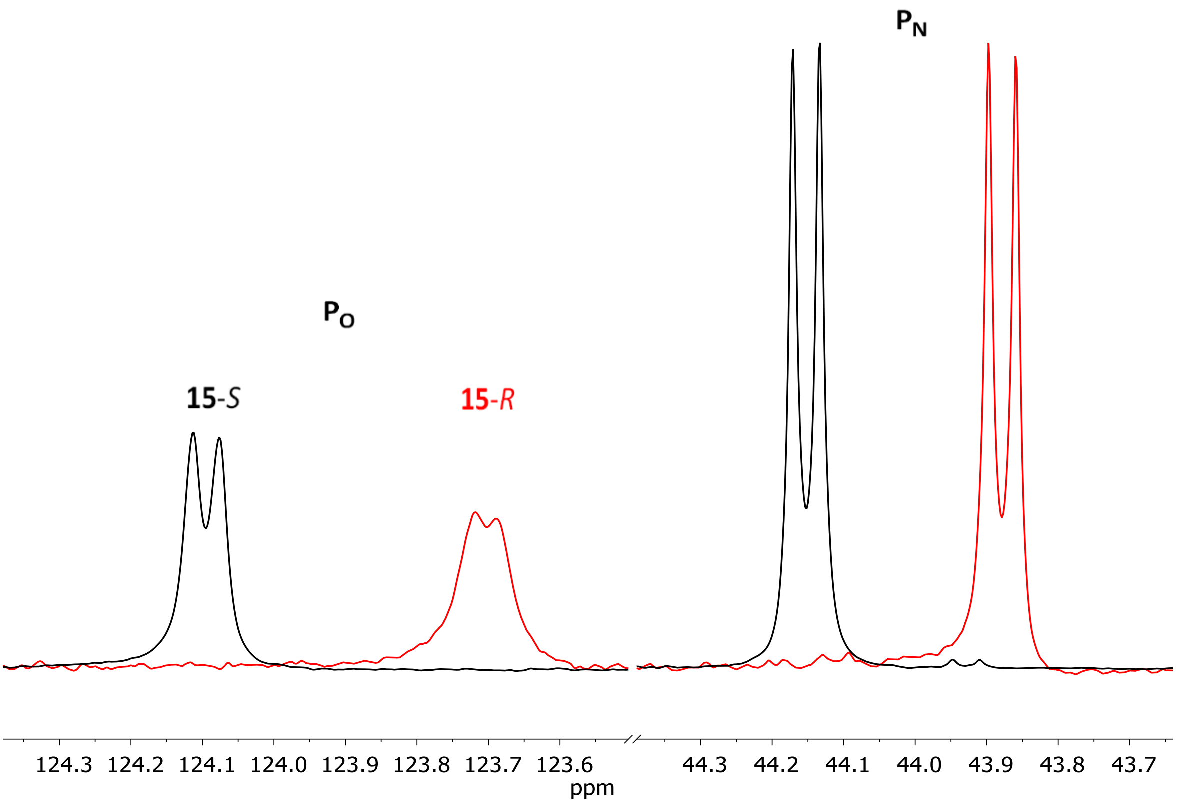

Figure S49. Overlaid expansions of the $^{31}P\{^{1}H\}$ NMR spectra of **15**-*R* & **15**-*S* (202 MHz, 298 K, $CDCl_3$).

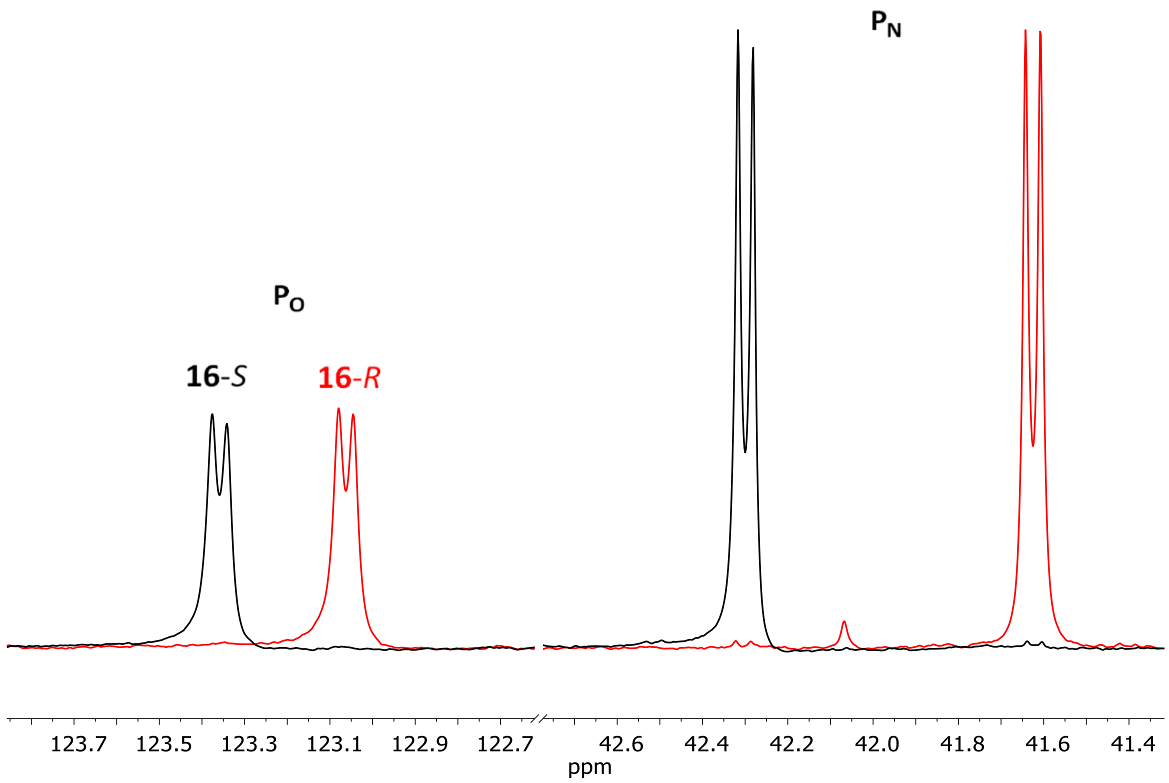

Figure S50. Overlaid expansions of the $^{31}P\{^{1}H\}$ NMR spectra of **16**-*R* & **16**-*S* (202 MHz, 298 K, $CDCl_3$).

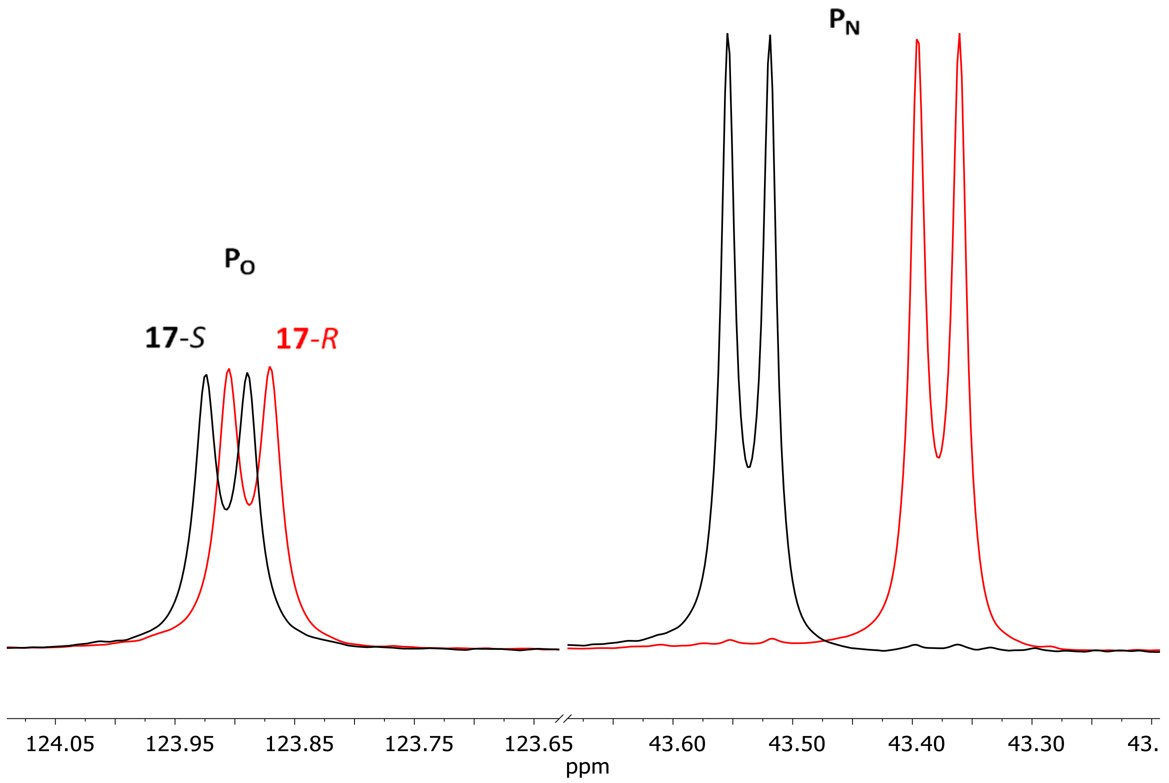

Figure S51. Overlaid expansions of the $^{31}P\{^{1}H\}$ NMR spectra of **17**-*R* & **17**-*S* (202 MHz, 298 K, $CDCl_3$).

Expansions of $^{31}P\{^1H\}$ spectra for substrates studied *in situ*

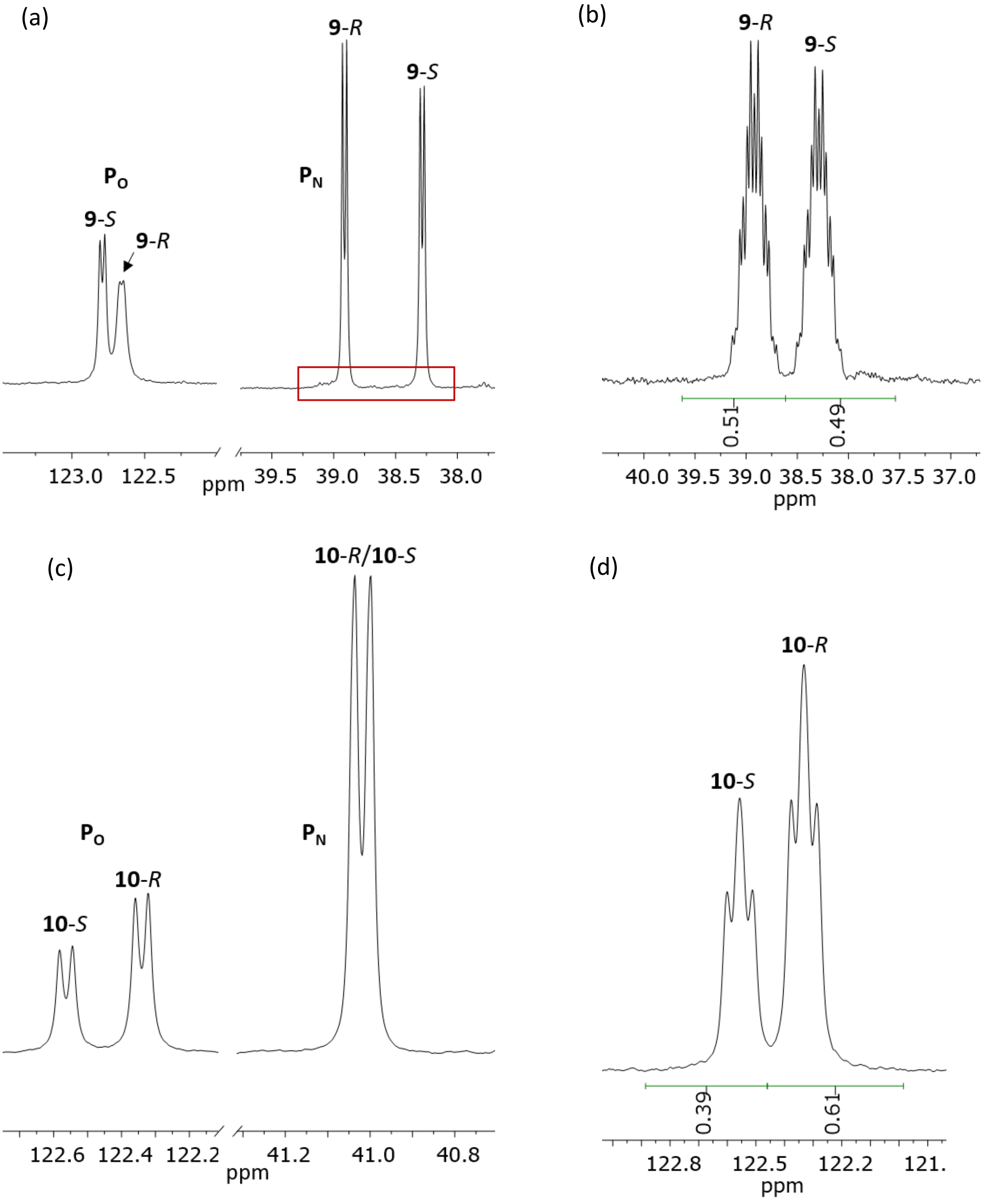

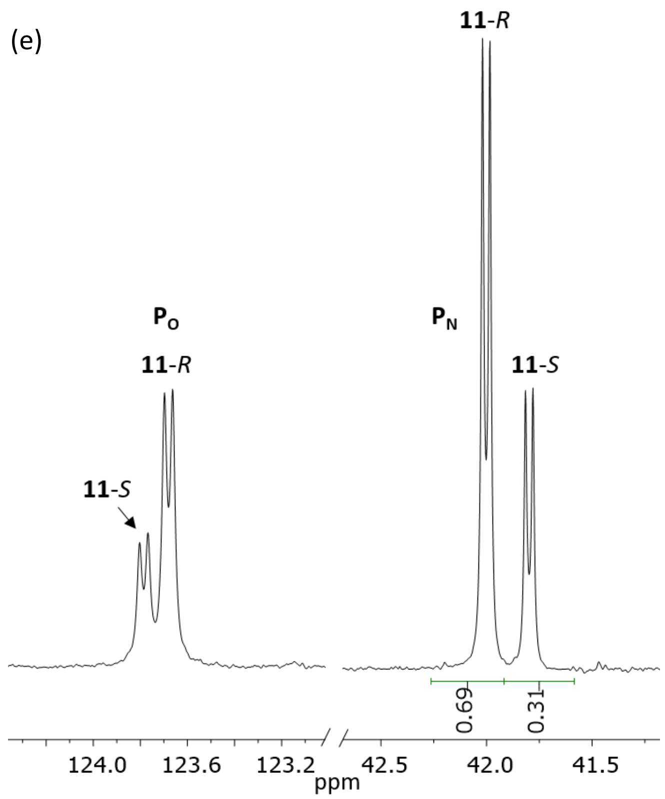

Figure S52. Expansions of (a) the $^{31}P\{^{1}H\}$ NMR spectrum of **9**-*R*/**9**-*S* (from commercial racemic 1-naphthylethylamine), (b) the ^{31}P NMR spectrum of **9**-*R*/**9**-*S*. A shoulder on the signal for **9**-*R* is seen within the red box. Assignment of the diastereomers is based on a separately prepared sample of **9**-*R*, (c) the $^{31}P\{^{1}H\}$ NMR spectra showing signals belonging to **10**-*R*/**10**-*S*, generated *in situ*, (d) expansion of the ^{31}P NMR spectrum showing the low-field multiplets arising from **10**-*R*/**10**-*S*. The assignments are based on the deliberate use of a ca. 1:2 ratio of the *S*:*R* isomers used in the experiment, (e) the $^{31}P\{^{1}H\}$ NMR spectrum of **11**-*R*/**11**-*S*. The assignments are based on the deliberate use of a *ca.* 1:2 ratio of the *S*:*R* isomers used in the experiment.

4. X-ray Crystallography

Tabulated X-ray data and figures of solid state structures

Table S1. Details of the data collections and refinements

	**1**	**2**	**3**	**4**-*R*	**4**-*S*
CCDC number	2105705	2105711	2105707	2105709	2105718
Cambridge data number	dw_k1_0043	dw_k1_0060	dw_k3_0097	dw_k1_0065	dw_k1_0048
Chemical formula	$C_{18}H_{35}ClN_2OP_2$	$C_{28}H_{52}N_2O_2P_2$	$C_{18}H_{36}N_2O_2P_2$	$C_{26}H_{45}N_3OP_2$	$C_{26}H_{45}N_3OP_2$
Moiety formula	$C_{18}H_{35}ClN_2OP_2$	$C_{28}H_{52}N_2O_2P_2$	$C_{18}H_{36}N_2O_2P_2$	$C_{26}H_{45}N_3OP_2$	$C_{26}H_{45}N_3OP_2$
Formula weight	392.87	510.65	374.43	477.59	477.59
Temperature / K	180(2)	180(2)	220(2)	180(2)	180(2)
Crystal system	monoclinic	orthorhombic	triclinic	monoclinic	orthorhombic
Space group	$P2_1$	$P2_12_12_1$	P1	$P2_1$	$P2_12_12_1$
a / Å	10.2116(2)	9.9457(2)	10.1507(2)	15.1934(2)	9.30350(10)
b / Å	22.6532(5)	13.5082(3)	10.4830(3)	13.5842(2)	15.6398(3)
c / Å	10.5288(2)	22.3174(6)	12.0198(4)	20.8780(3)	19.0287(4)
alpha / degrees	90	90	71.0631(9)	90	90
beta / degrees	114.8686(9)	90	77.4831(10)	101.5997(7)	90
gamma / degrees	90	90	65.8533(18)	90	90
Unit-cell volume / $Å^3$	2209.73(8)	2998.31(12)	1098.70(6)	4221.01(10)	2768.77(8)
Z	4	4	2	6	4
Calc. density / g cm^{-3}	1.181	1.131	1.132	1.127	1.146
F(000)	848	1120	408	1560	1040
Radiation type	MoKα	MoKα	MoKα	MoKα	MoKα
Absorption coefficient / mm^{-1}	0.326	0.171	0.210	0.176	0.179
Crystal size / mm^3	0.50x0.40x0.350	0.44x0.44x0.440	0.35x0.20x0.20	0.48x0.48x0.48	0.46x0.32x0.22
2-Theta range / degrees	7.13-54.94	7.05-54.94	7.31-54.87	7.20-50.68	7.14-54.96
Completeness to max 2-theta / %	0.964	0.984	0.975	0.996	0.995
No. of reflections measured	16786	24851	4894	62942	19153
No. of independent reflections	8105	6596	4894	15379	6243
R(int)	0.0337	0.0499	0.0602	0.0550	0.0402
No. parameters / restraints	451 / 1	319 / 0	585 / 401	895 / 1	303 / 0
Final R1 values ($I > 2\sigma(I)$)	0.0356	0.0379	0.0761	0.0390	0.0364
Final wR(F^2) values (all data)	0.0451	0.0620	0.1050	0.0660	0.0531
Goodness-of-fit on F^2	1.035	0.954	1.085	0.980	1.005
Largest difference peak & hole / e $Å^{-3}$	0.226, -0.157	0.272, -0.218	0.508, -0.377	0.219, -0.215	0.189, -0.294
Flack parameter	0.07(5)	-0.01(3)	0.11(14)	-0.02(2)	-0.04(3)

Table S1 Details of the data collections and refinements (*continued*)

	**5**-*R*	**5**-*S*	**6**-*R*	**6**-*S*	**7**-*R*
CCDC number	2105714	2105706	2105708	2105717	2105710
Cambridge data number	dw_k1_0069	dw_k1_0070	dw_k3_0082	dw_k3_0083	dw_k3_0098
Chemical formula	$C_{27}H_{48}IN_3OP_2$	$C_{27}H_{48}IN_3OP_2$	$C_{28}H_{50}IN_3OP_2$	$C_{28}H_{50}IN_3OP_2$	$C_{27}H_{47}N_3OP_2$
Moiety formula	$C_{27}H_{48}N_3OP_2$, I	$C_{27}H_{48}N_3OP_2$, I	$C_{28}H_{50}N_3OP_2$, I	$C_{28}H_{50}$N3OP_2, I	$C_{27}H_{47}N_3OP_2$
Formula weight	619.52	619.52	633.55	633.55	491.61
Temperature / K	180(2)	180(2)	180(2)	180(2)	180(2)
Crystal system	orthorhombic	orthorhombic	orthorhombic	orthorhombic	triclinic
Space group	$P2_12_12_1$	$P2_12_12_1$	$P2_12_12_1$	$P2_12_12_1$	P1
a / Å	11.7716(2)	11.33750(10)	11.2847(2)	11.36910(10)	9.53450(10)
b / Å	15.9064(3)	16.0440(2)	16.6693(3)	16.4031(2)	11.99090(10)
c / Å	16.4323(3)	17.0248(2)	16.9948(3)	17.2940(2)	13.3121(2)
alpha / degrees	90	90	90	90	104.3794(5)
beta / degrees	90	90	90	90	90.1360(5)
gamma / degrees	90	90	90	90	94.2154(7)
Unit-cell volume / Å3	3076.85(10)	3096.79(6)	3196.86(10)	3225.13(6)	1469.94(3)
Z	4	4	4	4	2
Calc. density / g cm-3	1.337	1.329	1.316	1.305	1.111
F(000)	1288	1288	1320	1320	536
Radiation type	MoKα	MoKα	MoKα	MoKα	MoKα
Absorption coefficient / mm^{-1}	1.168	1.160	1.125	1.116	0.170
Crystal size / mm^3	0.53x0.28x0.22	0.35x0.32x0.28	0.25x0.06x0.06	0.22x0.22x0.14	0.35x0.22x0.22
2-Theta range / degrees	7.13-54.90	7.19-54.99	7.22-52.04	7.17-51.37	7.92-50.75
Completeness to max 2-theta / %	0.993	0.995	0.992	0.995	0.993
No. of reflections measured	44228	35098	26953	33511	36151
No. of independent reflections	7002	7075	6213	6107	10609
R(int)	0.0553	0.0461	0.0528	0.0488	0.0383
No. parameters / restraints	318 / 0	307 / 0	331 / 0	331 / 0	623 / 3
Final R1 values ($I > 2\sigma(I)$)	0.0332	0.0291	0.0338	0.0317	0.0414
Final wR(F^2) values (all data)	0.0628	0.0510	0.0478	0.0397	0.0460
Goodness-of-fit on F^2	1.021	0.996	1.049	1.040	1.057
Largest difference peak & hole / e $Å^{-3}$	0.554, -0.412	0.328, -0.486	0.257, -0.585	0.406, -0.647	0.391, -0.205
Flack parameter	-0.027(7)	-0.030(6)	-0.025(9)	-0.030(8)	-0.01(2)

Table S1 Details of the data collections and refinements (*continued*)

	**7**-*S*	**7**-*R*/*S*	**8**-*R*	**8**-*S*	**8**-*R*/*S*
CCDC number	2105715	2105712	2105716	2105713	2105719
Cambridge data number	dw_b2_0300	dw_k3_0084b	dw_k3_0090	dw_k3_0092	dw_k3_0081a
Chemical formula	$C_{27}H_{47}N_3OP_2$	$C_{27}H_{47}N_3OP_2$	$C_{28}H_{50}IN_3OP_2$	$C_{28}H_{50}IN_3OP_2$	$C_{28}H_{50}IN_3OP_2$
Moiety formula	$C_{27}H_{47}N_3OP_2$	$C_{27}H_{47}N_3P_2$	$C_{28}H_{50}N_3OP_2$, I	$C_{28}H_{50}N_3OP_2$, I	$C_{28}H_{50}N_3OP_2$, I
Formula weight	491.61	491.61	633.55	633.55	633.55
Temperature / K	180(2)	180(2)	180(2)	180(2)	180(2)
Crystal system	triclinic	triclinic	triclinic	monoclinic	monoclinic
Space group	P1	P1	P1	$P2_1$	$P2_1$
a / Å	9.53450(10)	9.5547(3)	9.5482(2)	9.98080(10)	10.0206(2)
b / Å	11.99090(10)	11.9843(4)	12.0125(3)	28.8020(3)	28.8500(6)
c / Å	13.3121(2)	13.3167(4)	13.2604(4)	11.8786(2)	11.9155(3)
alpha / degrees	104.3794(5)	103.719(2)	103.8986(9)	90	90
beta / degrees	90.1360(5)	90.305(2)	90.8396(8)	109.4314(4)	110.3822(10)
gamma / degrees	94.2154(7)	94.423(2)	94.8447(15)	90	90
Unit-cell volume / $Å^3$	1469.94(3)	1476.49(8)	1470.17(7)	3220.20(7)	3229.03(13)
Z	2	2	2	4	4
Calc. density / g cm^{-3}	1.111	1.106	1.111	1.307	1.303
F(000)	536	536	536	1320	1320
Radiation type	MoKα	CuKα	MoKα	MoKα	MoKα
Absorption coefficient / mm^{-1}	0.170	1.496	0.170	1.117	1.114
Crystal size / mm^3	0.35x0.22x0.22	0.22x0.16x0.05	0.35x0.35x0.30	0.18x0.18x0.14	0.18x0.18x0.08
2-Theta range / degrees	7.92-50.75	6.83-133.52	7.70-54.89	7.10-55.00	7.07-50.69
Completeness to max 2-theta / %	0.993	0.995	0.965	0.971	0.964
No. of reflections measured	36151	24926	15471	35917	22447
No. of independent reflections	10609	9670	10018	13964	10320
R(int)	0.0383	0.0365	0.0331	0.0522	0.0550
No. parameters / restraints	623 / 3	771 / 477	652 / 99	742 / 220	653 / 98
Final R1 values (I > 2s(I))	0.0414	0.0562	0.0565	0.0423	0.0516
Final wR(F2) values (all data)	0.0460	0.0608	0.0747	0.0743	0.0820
Goodness-of-fit on F2	1.057	1.032	1.040	1.021	1.021
Largest difference peak & hole / e $Å^{-3}$	0.391, -0.205	0.463, -0.230	0.432, -0.253	0.617, -0.514	0.508, -0.937
Flack parameter	-0.01(2)	0.032(19)	-0.04(6)	-0.035(12)	-0.026(15)

Table S1 Details of the data collections and refinements (*continued*)

	**10**-*R*	**10**-*S*	**12**-*R*	**12**-*S*
CCDC number	2157896	2157893	2157895	2157894
Cambridge data number	DW_B2_0365	DW_B2_0363	DW_B2_0353	DW_B2_0354
Chemical formula	$C_{27}H_{54}IN_3OP_2$	$C_{27}H_{54}IN_3OP_2$	$C_{23}H_{48}IN_3OP_2$	$C_{23}H_{48}IN_3OP_2$
Moiety formula	$C_{27}H_{54}N_3OP_2$, I	$C_{27}H_{54}N_3OP_2$, I	$C_{23}H_{48}N_3OP_2$, I	$C_{23}H_{48}N_3OP_2$, I
Formula weight	625.57	625.57	571.48	571.48
Temperature / K	180(2)	180(2)	220(2)	220(2)
Crystal system	monoclinic	orthorhombic	monoclinic	monoclinic
Space group	$P2_1$	$P2_12_12_1$	C2	$P2_1$
a / Å	9.6161(6)	11.0895(6)	14.3333(7)	14.2761(11)
b / Å	35.768(2)	16.8933(9)	11.7334(5)	11.8123(10)
c / Å	9.7814(6)	17.0952(9)	18.0008(8)	18.2335(17)
alpha / degrees	90	90	90	90
beta / degrees	98.955(2)	90	97.111(2)	97.663(5)
gamma / degrees	90	90	90	90
Unit-cell volume / $Å^3$	3323.3(4)	3202.6(3)	3004.1(2)	3047.3(5)
Z	4	4	4	4
Calc. density / g cm^{-3}	1.250	1.297	1.264	1.246
F(000)	1312	1312	1192	1192
Radiation type	CuKα	CuKα	CuKα	CuKα
Absorption coefficient / mm^{-1}	8.635	8.961	9.505	9.370
Crystal size / mm^3	0.35x0.28x0.06	0.20x0.10x0.10	0.25x0.20x0.02	0.14x0.14x0.01
2-Theta range / degrees	4.94-133.04	7.36-133.02	4.95-133.36	4.89-134.79
Completeness to max 2-theta / %	0.998	0.997	0.995	0.993
No. of reflections measured	55121	30670	22428	73492
No. of independent reflections	11587	5628	5150	10812
R(int)	0.0367	0.0346	0.0446	0.1232
No. parameters / restraints	714 / 109	322 / 0	271 / 162	565 / 360
Final R1 values (I > 2σ(I))	0.0330	0.0189	0.0752	0.0656
Final wR(F^2) values (all data)	0.0917	0.0452	0.2129	0.2067
Goodness-of-fit on F^2	1.037	1.072	1.057	1.051
Largest difference peak & hole / e $Å^{-3}$	0.629, -0.684	0.228, -0.526	1.548, -1.297	0.648, -0.819
Flack parameter	-0.007(2)	-0.0135(16)	-0.001(12)	-0.004(5)

Table S1 Details of the data collections and refinements (*continued*)

	**13**-*R*	**13**-*S*
CCDC number	2157891	2157892
Cambridge data number	DW_B1_0426	DW_B2_0359
Chemical formula	$C_{28.5}H_{56}IN_3OP_2$	$C_{28.5}H_{56}IN_3OP_2$
Moiety formula	$C_{25}H_{52}N_3OP_2$, I, 0.5(C_7H_8)	$C_{25}H_{52}N_3OP_2$, I, 0.5(C_7H_8)
Formula weight	645.60	645.60
Temperature / K	220(2)	220(2)
Crystal system	monoclinic	monoclinic
Space group	C2	C2
a / Å	14.361(2)	14.3801(5)
b / Å	11.6776(18)	11.8816(5)
c / Å	20.980(4)	20.9273(9)
alpha / degrees	90	90
beta / degrees	92.239(10)	100.971(2)
gamma / degrees	90	90
Unit-cell volume / $Å^3$	3515.6(10)	3510.3(2)
Z	4	4
Calc. density / g cm^{-3}	1.220	1.222
F(000)	1356	1356
Radiation type	CuKα	CuKα
Absorption coefficient / mm^{-1}	8.179	8.191
Crystal size / mm^3	0.12x0.12x0.01	0.18x0.18x0.02
2-Theta range / degrees	8.44-108.15	8.61-133.19
Completeness to max 2-theta / %	0.998	0.997
No. of reflections measured	14137	30591
No. of independent reflections	4246	6095
R(int)	0.1451	0.0704
No. parameters / restraints	308 / 197	427 / 311
Final R1 values (I > 2σ(I))	0.0719	0.0650
Final wR(F^2) values (all data)	0.1910	0.1836
Goodness-of-fit on F^2	1.036	1.079
Largest difference peak & hole / e $Å^{-3}$	0.418, -0.529	1.160, -1.459
Flack parameter	0.034(17)	0.011(8)

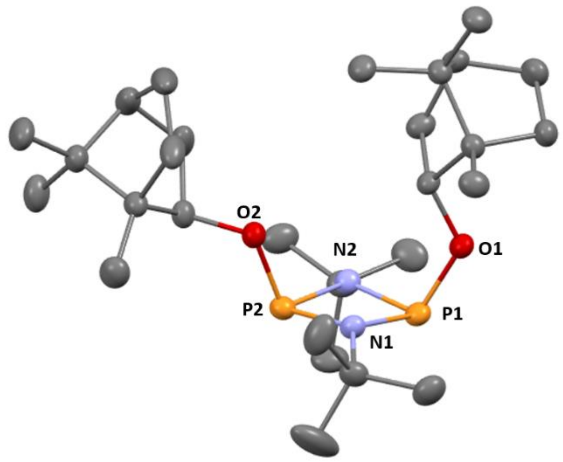

Figure S53. (a) Molecular structure of **2** (ellipsoids at 30% probability) with hydrogen atoms omitted. Selected bond lengths (Å) and angles (°): P1–N1 1.723(2), P2–N1 1.707(2), P2–N2 1.713(2), P1–N2 1.728(2), P1–O1 1.6239(17), P2–O1 1.6492(17), P1–N2–P2 97.68(10), P2–N1–P1 98.10(10), N1–P1–N2 80.94(9), N1–P2–N2 81.83(9), O1–P1–N1 106.15(9), O1–P1–N2 105.86(10), O2–P2–N1 101.41(9) O2–P2–N2 104.73(9).

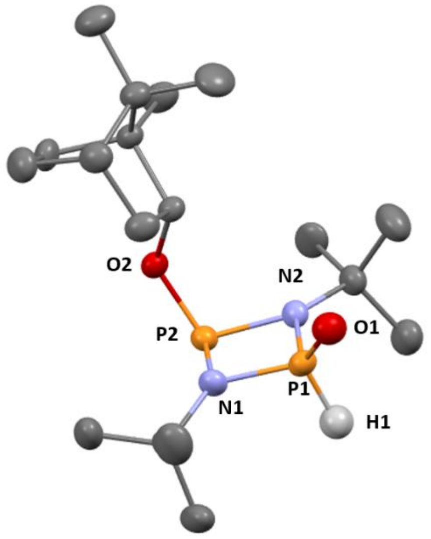

Figure S54. The molecular structure of **3** (thermal ellipsoids drawn at 30% probability). Only one molecule of the asymmetric unit is shown, and H-atoms are omitted for clarity. Metric parameters are not given due to twinning and disorder.

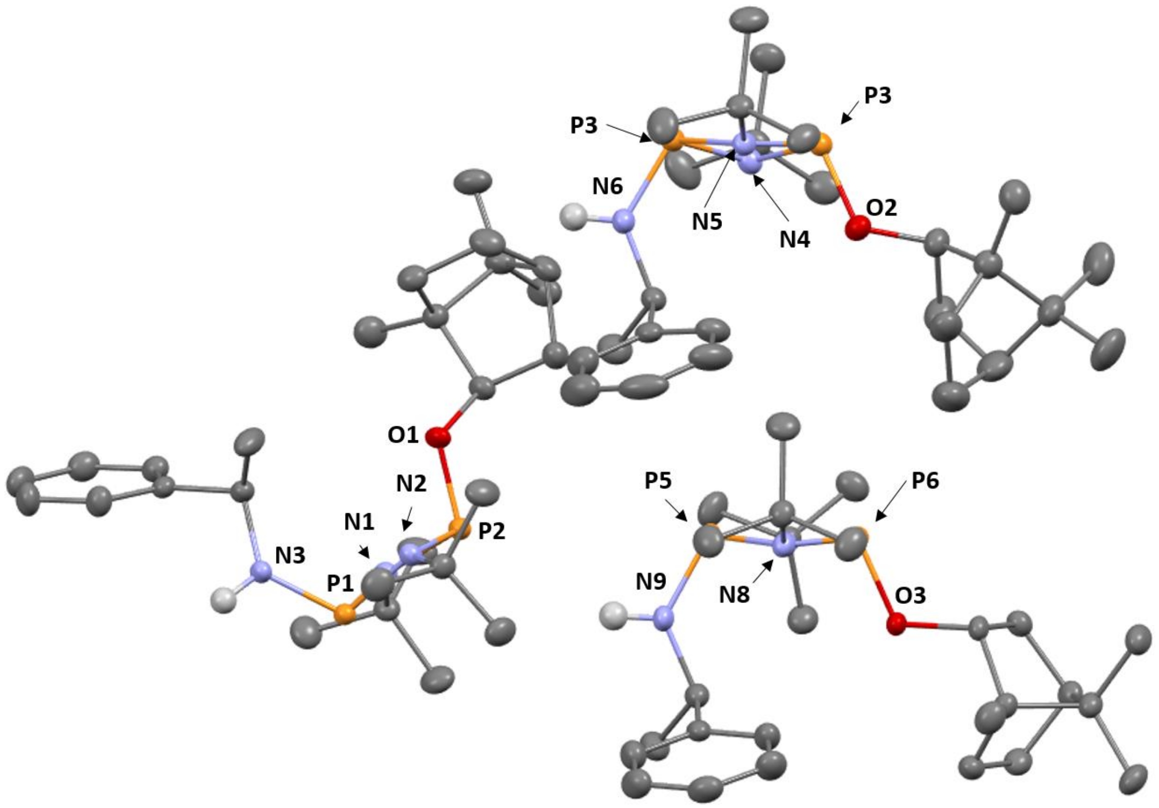

Figure S55. Contents of the asymmetric unit of **4**-*R*. Hydrogen atoms omitted for clarity (ellipsoids set at 30% probability). Selected bond lengths Å and angles (°): Molecule 1: P1–N1 1.734(3), N1–P2 1.706(3), P2–N2 1.698(3), N2–P1 1.730(3), P1–N3 1.660(3), P2–O1 1.6421(19); P1–N1–P2 97.83(13), N1–P2–N2 81.91(12), P2–N2–P1 98.28(13), N2–P1–N1 80.19(12), N1–P1–N3 105.81(12), N2–P1–N3 105.63(12). Molecule 2: P3–N4 1.740(3), N4–P4 1.698(3), P4–N5 1.719(3), N5–P3 1.730(3) P3–N6 1.670(3), P4–O2, 1.644(2); P3–N4–P4 97.96(13), N4–P4–N5 81.65(13), P4–N5–P3 97.55(13), N5–P3–N4 80.15(13), N4–P3–N6 108.10(12), N5–P3–N6 103.74(13), N4–P4–O2 101.17(12), N5–P4–O2 105.94(12). Molecule 3: P5–N7 1.747(3), N7–P6 1.705(3), P6–N8 1.699(3), N8–P5 1.716(3), P5–N9 1.672(3), P6–O3, 1.656(2); P5–N7–P6 97.70(13), N7–P6–N8 81.59(13), P6–N8–P5 99.12(13), N8–P5–N7 79.93(12), N9–P5–N7 107.84(12), N9–P5–N8 105.09(13), O3–P6–N7 104.52(11), O3–P6–N8 103.79(11).

(a)

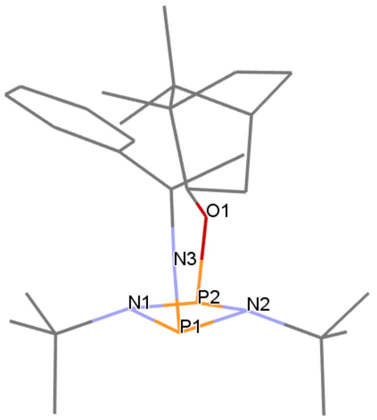

(b)

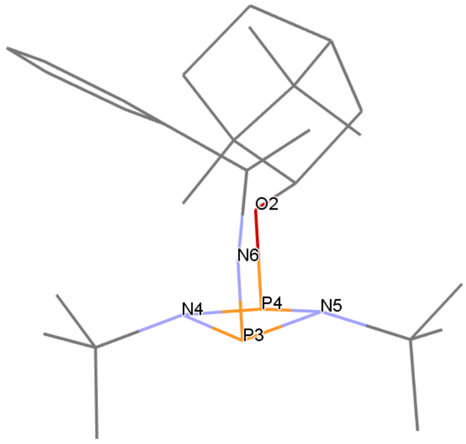

(c)

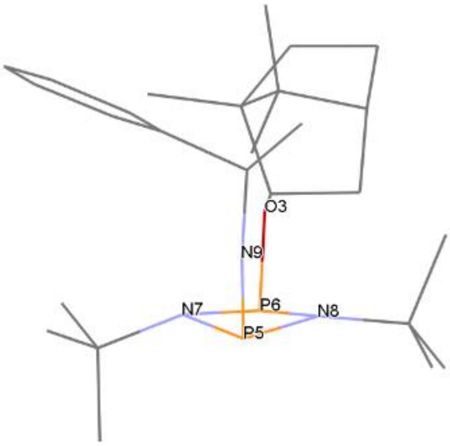

Figure S56. Wireframe representations of the three independent molecules in the asymmetric unit of **4**-*R*.

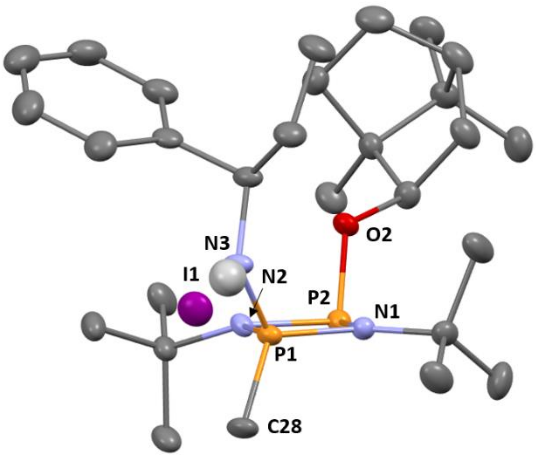

Figure S57. Molecular structure of **6**-*R* (ellipsoids at 30% probability). Hydrogen atoms omitted for clarity. Selected bond lengths (Å) and angles (°): P1–N1 1.647(4), N1–P2 1.743(4), P2–N2 1.737(3), N2–P1 1.651(4), P1–N3 1.598(4), P1–C28 1.788(4), P2–O1 1.628(3); P1–N1–P2 96.85(18), N1–P2–N2 80.35(16), P2–N2–P1 96.95(19), N2–P1–N1 85.78(17), N1–P1–N3 117.71(19), N2–P1–N3 114.63(19),

N1–P1–C28 116.5(2), N2–P1–C28 116.9(2), N3–P1–C28 105.2(2).

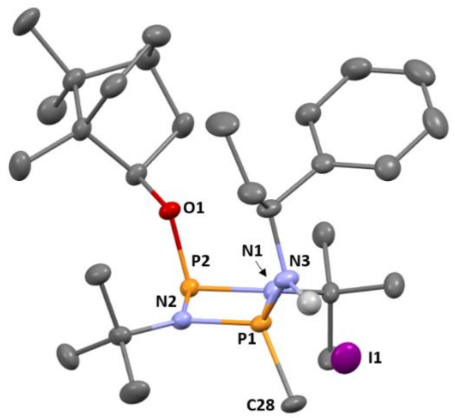

Figure S58. Molecular structure of **6**-*S* (ellipsoids at 30% probability). Hydrogen atoms omitted for clarity. Selected bond lengths (Å) and angles (°): P1–N1 1.653(3), N1–P2 1.745(3), P2–N2 1.730(3), N2–P1 1.647(3), P1–N3 1.607(3), P1–C28 1.780(4), P2–O1 1.632(3); P1–N1–P2 96.60(17), N1–P2–N2 80.37(16), P2–N2–P1 97.40(17), N2–P1–N1 85.60(16), N1–P1–N3 115.25(18), N2–P1–N3 117.55(18), N1–P1–C28 117.3(2), N2–P1–C28 115.7(2), N3–P1–C28 105.2(2).

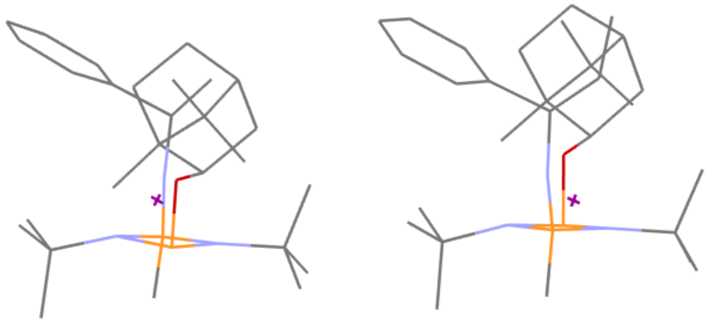

(a)

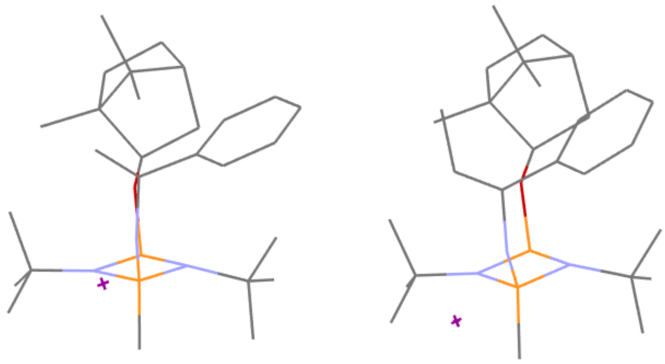

(b)

Figure S59. Projections of (a) **5**-*R* and **6**-*R*, and (b) **5**-*S* and **6**-*S* showing the nearly identical geometrical features between diastereomers of like stereochemistry.

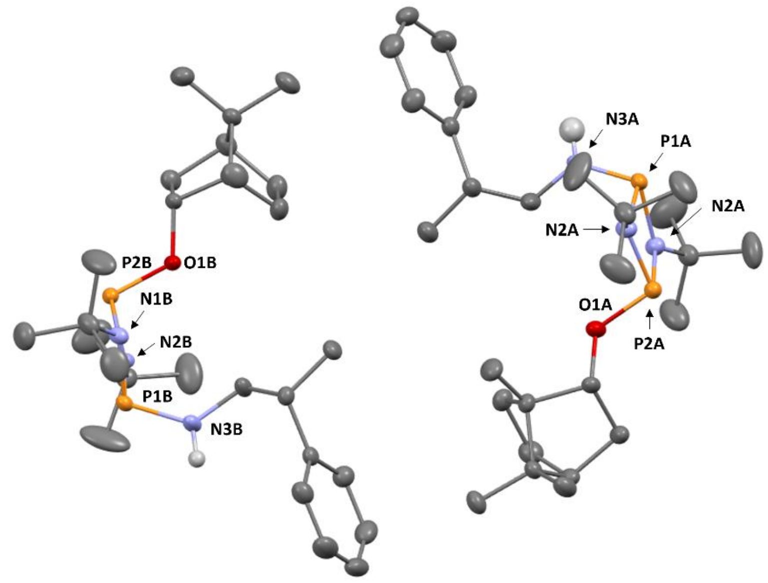

Figure S60. The structures of **7**-*R*. Ellipsoids at 30% probability, disorder omitted for clarity.

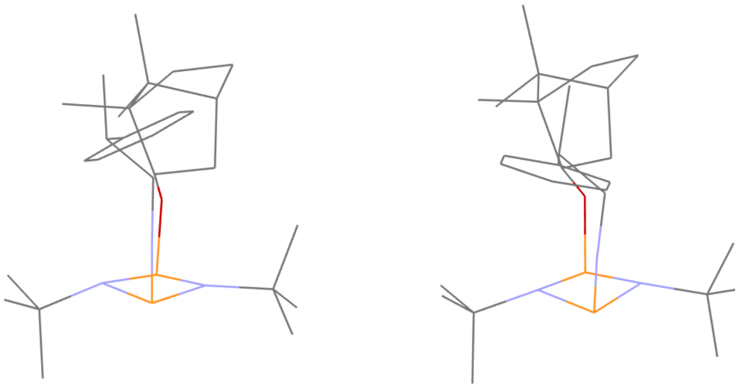

Figure S61. Projections of **7**-*R* showing the different orientation of the amino substituents in the two independent molecules in the unit cell.

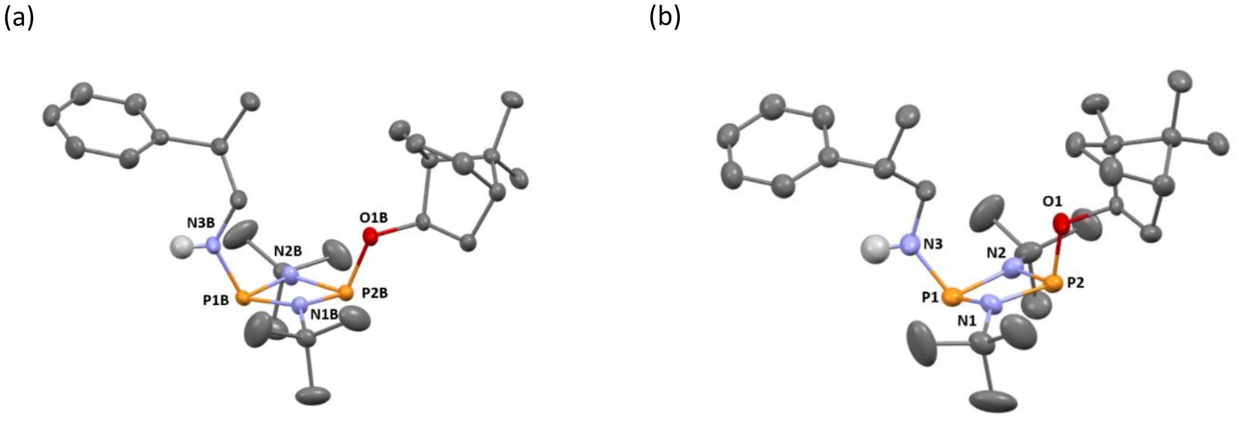

Figure S62. (a) The structure of a representative molecule from the asymmetric unit of **7**-*R* (ellipsoids at 30% probability). C–H hydrogen atoms omitted for clarity. Selected bond lengths (Å) and angles (°): P1B–N1B 1.735(3), N1B–P2B 1.707(3), P2B–N2B 1.708(3), N2B–P1B 1.729(3), P1B–N3B 1.650(4),

P2B–O1B 1.647(3); P1B–N1B–P2B 98.09(17), N1B–P2B–N2B 81.85(16), P2B–N2B–P1B 98.28(17), N2B–P1B-N1B 80.43(16), N1B–P1B–N3B 104.42(19), N2B–P1B–N3B 107.3(2), N1B–P2B–O1B 100.26(16), N2B–P2B–O1B 105.15(16) and (b) The structure of a representative molecule from the asymmetric unit of **7**-*S* (ellipsoids at 30% probability). C–H hydrogen atoms omitted for clarity. Selected bond lengths (Å) and angles (°): Selected bond lengths (Å) and angles (°): P1–N1 1.727(4), N1–P2 1.706(4), P2–N2 1.706(4), N2–P1 1.721(5), P1–N3 1.658(5), P2–O1 1.638(4); P1–N1–P2 98.5(2), N1–P2–N2 81.5(2), P2–N2–P1 98.7(2), N2–P1–N1 80.4(2), N1–P1–N3 107.1(2), N2–P1–N3 105.1(2), N1–P2–O1 101.9(2), N2–P2–O1 103.4(3).

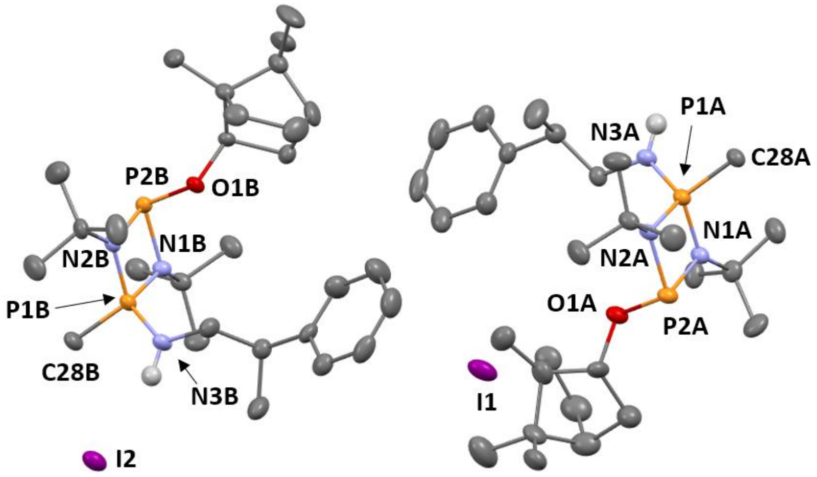

Figure S63. The contents of the asymmetric unit of **8**-*R*. Ellipsoids at 30% probability. Disorder omitted for clarity.

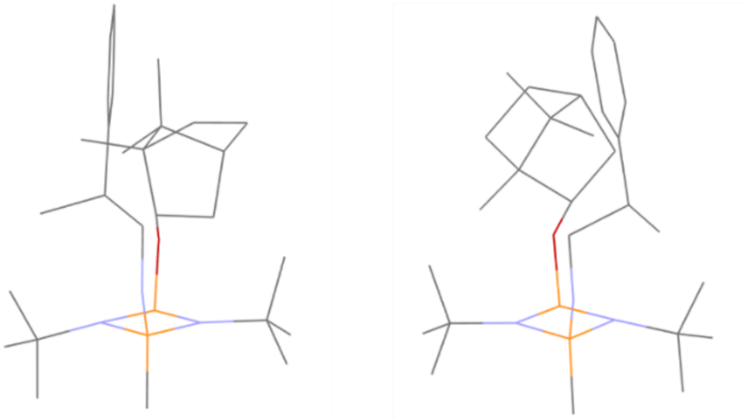

Figure S64. Side-by side projections of the two crystallographically independent cations in **8**-*R*.

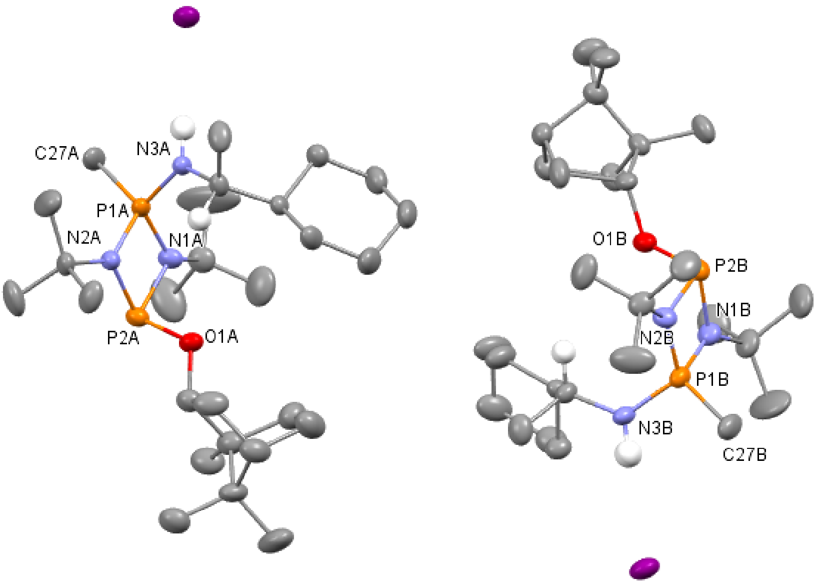

Figure S65. The contents of the asymmetric unit of **10**-*R*, showing the two crystallographically independent molecules. C–H atoms have been omitted for clarity. Ellipsoids at 30% probability. Selected bond lengths (Å) and angles (°): Molecule 1, P1A–N1A 1.646(5), P2A–N1A 1.738(4), P1A–N2A 1.636(4), P2A–N2A 1.736(4), P1A–N3A 1.612(4), P2A–O1A 1.617(4), P1A–N1A–P2A 96.3(2), P1A–N2A–P2A 96.8(2), N1A–P1A–N2A 86.3(2), N1A–P2A–N2A 80.5(2), N1A–P1A–N3A 119.5(2), N2A–P1A–N3A 114.2(2), N1A–P2A–O1A 101.3(2), N2A–P2A–O1A 103.9(2). Molecule 2, P1B–N1B 1.654(5), P2B–N1B 1.724(5), P1B–N2B 1.632(4), P2B–N2B 1.743(5), P1B–N3B 1.600(5), P2B–O1B 1.620(4), P1B–N1B–P2B 96.6(2), P1B–N2B–P2B 96.7(2), N1B–P1B–N2B 86.0(2), N1B–P2B–N2B 80.5(2), N1B–P1B–N3B 119.4(3), N2B–P1B–N3B 114.4(2), N1A–P2A–O1A 100.5(2), N2A–P2A–O1A 103.4(2).

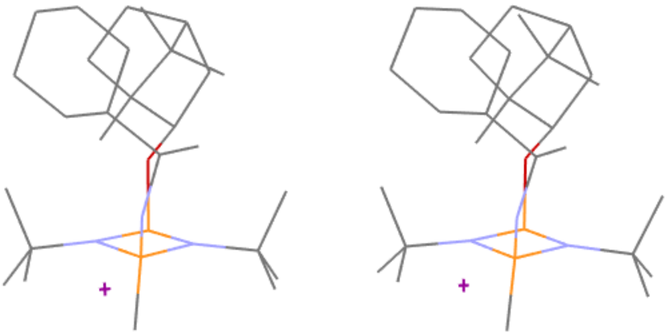

Figure S66. Side-by side projections of **10**-*R* showing the very similar orientation of the amino substituents of the two independent molecules in the unit cell.

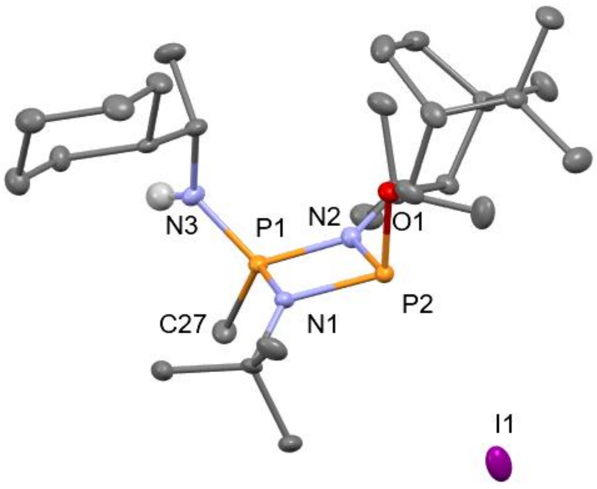

Figure S67. The contents of the asymmetric unit of **10**-*S*. C-H atoms have been omitted for clarity Ellipsoids at 30% probability. P1–N1 1.652(2), P2–N1 1.755(2), P1–N2 1.642(2), P2–N2 1.728(2), P1–N3 1.604(2), P2–O1 1.626(2), P1–N1–P2 96.4(1), P1–N2–P2 97.8(1), N1–P1–N2 85.7(1), N1–P2–N2 80.1(1), N1–P1–N3 115.4(1), N2–P1–N3 117.4(1), N1–P2–O1 103.5(1), N2–P2–O1 100.1(1).

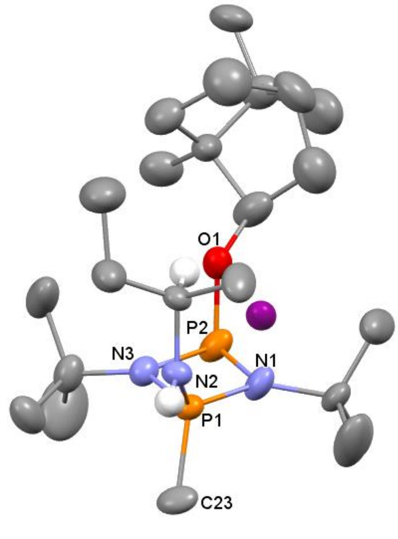

Figure S68. The contents of the asymmetric unit of **12**-*R*. C-H atoms have been omitted for clarity Ellipsoids at 30% probability. P1–N1 1.64(1), P2–N1 1.73(1), P1–N2 1.66(1), P2–N2 1.72(1), P1–N3 1.63(1), P2–O1 1.60(1), P1–N1–P2 97.1(6), P1–N2–P2 96.4(5), N1–P1–N2 85.6(6), N1–P2–N2 80.8(5), N1–P1–N3 118.0(6), N2–P1–N3 115.9(6), N1–P2–O1 104.0(6), N2–P2–O1 100.6(5).

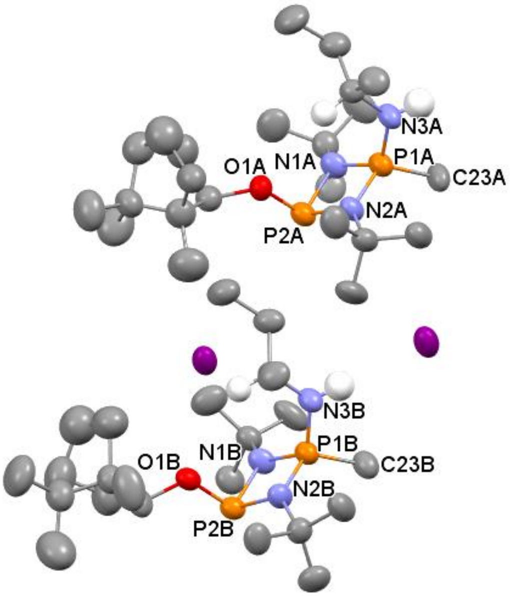

Figure S69. The contents of the asymmetric unit of **12**-*S*, showing the two crystallographically independent molecules. C-H atoms have been omitted for clarity. Ellipsoids at 30% probability. Selected bond lengths (Å) and angles (°): Molecule 1, P1A–N1A 1.65(1), P2A–N1A 1.74(1), P1A–N2A 1.644(9), P2A–N2A 1.724(9), P1A–N3A 1.59(1), P2A–O1A 1.614(9), P1A–N1A–P2A 96.6(5), P1A–N2A–P2A 97.1(4), N1A–P1A–N2A 85.6(5), N1A–P2A–N2A 80.5(4), N1A–P1A–N3A 117.7(6), N2A–P1A–N3A 114.7(6), N1A–P2A–O1A 103.4(5), N2A–P2A–O1A 99.5(5). Molecule 2, P1B–N1B 1.63(1), P2B–N1B 1.728(9), P1B–N2B 1.65(1), P2B–N2B 1.729(9), P1B–N3B 1.59(1), P2B–O1B 1.607(9), P1B–N1B–P2B 97.3(4), P1B–N2B–P2B 96.3(4), N1B–P1B–N2B 85.8(5), N1B–P2B–N2B 80.5(4), N1B–P1B–N3B 116.0(6), N2B–P1B–N3B 116.1(6), N1A–P2A–O1A 100.9(5), N2A–P2A–O1A 100.9(5).

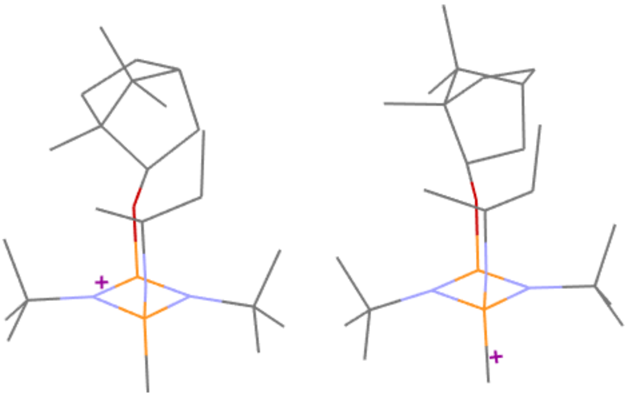

Figure S70. Side-by side projections of the two crystallographically independent cations in **12**-*S* (iodide counterions also shown).

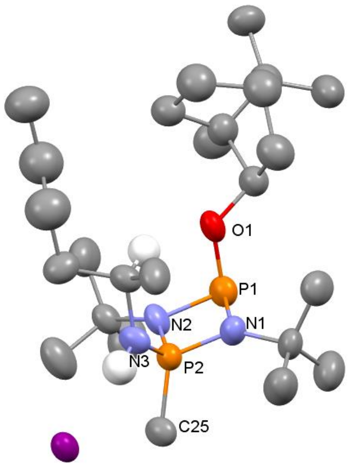

Figure S71. The contents of the asymmetric unit of **13**-*R*. C-H atoms and the disordered toluene molecule in the asymmetric unit have been omitted for clarity. Ellipsoids at 30% probability. P1–N1 1.75(2), P2–N1 1.60(2), P1–N2 1.69(2), P2–N2 1.66(2), P1–N3 1.57(2), P2–O1 1.63(2), P1–N1–P2 97.9(9), P1–N2–P2 97.9(8), N1–P1–N2 79(1), N1–P2–N2 85(1), N1–P1–N3 116(1), N2–P1–N3 118(1), N1–P2–O1 103.1(9), N2–P2–O1 100.6(8).

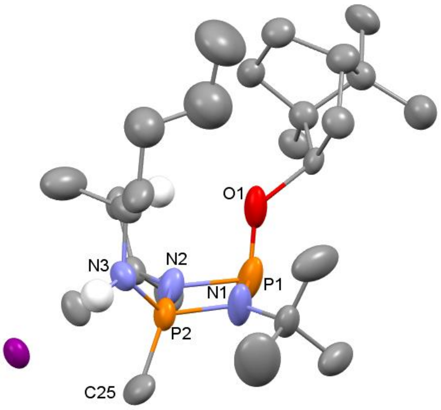

Figure S72. The contents of the asymmetric unit of **13**-*S*. C-H atoms, toluene molecule in the asymmetric unit and the disorder in the OBorn group have been omitted for clarity. Ellipsoids at 30% probability. P1–N1 1.70(1), P2–N1 1.66(1), P1–N2 1.74(1), P2–N2 1.65(1), P1–N3 1.59(1), P2–O1 1.60(2), P1–N1–P2 97.3(6), P1–N2–P2 96.5(6), N1–P1–N2 81.0(6), N1–P2–N2 85.1(6), N1–P1–N3 118.3(7), N2–P1–N3 115.8(6), N1–P2–O1 103.1(7), N2–P2–O1 100.3(6).

Conformational analysis in the solid state

The molecular conformation at the OBorn group is quantified by the P···P–O–C_{Born} and the P–O–C_{Born}–C(Me) torsion angles, denoted τ_1 and τ_2, respectively, as depicted below (see Figure 14a in the paper):

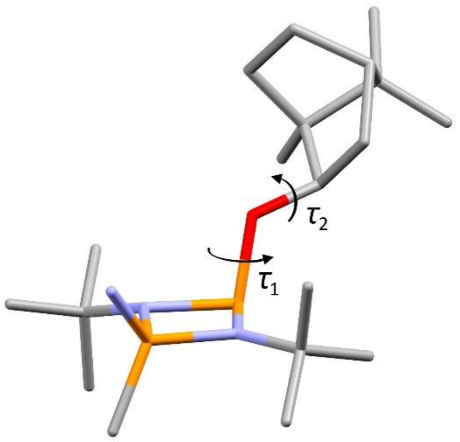

The Born-*endo* conformation has τ_1 close to 0 and Born-*exo* has τ_1 approaching 180°. The τ_2 angle indicates the rotation of the OBorn group (and particularly its peripheral Me group) relative to the P_2N_2 plane. Values are given below for each independent molecule (indicated by _1, _2…) in the crystal structures and each disorder component (indicated by A, B…) where relevant. Values are rounded to the nearest integer, which is realistic within the uncertainties and sufficient to demonstrate similarity. For the disordered cases, the uncertainty on the values can be expected to be larger than for the ordered cases.

Table S2. Torsion angles τ_1 and τ_2 for XRD-characterised solid structures.

Molecule	CIF atom labels	Conformation (Born-*endo* or *exo*)	P···P–O–C angle τ_1 (°)	P–O–C–C(Me) angle τ_2 (°)
**1**_1	P1/P2	*endo*	2	150
**1**_2	P3/P4	*endo*	3	159
**2**	P1	*endo*	2	148
**2**	P2	*exo*	156	124
**3**_1	P1/P2	*endo*	11	160
**3**_2A	P1A/P2A	*endo*	9	156
**3**_2B	P1A/P2A	*endo*	22	123
**4**-*R*_1	P1/P2	*exo*	154	157
**4**-*R*_2	P3/P4	*exo*	157	122
**4**-*R*_3	P5/P6	*exo*	178	157
**4**-*S*	P1/P2	*exo*	176	141
**5**-*R*	P1/P2	*exo*	158	121
**5**-*S*	P1/P2	*exo*	172	141
**6**-*R*	P1/P2	*exo*	157	124
**6**-*S*	P1/P2	*exo*	172	140
**7**-*R*_1	P1A/P2A	*exo*	170	152

**7**-*R*_2	P1B/P2B	*exo*	162	155
**7**-*R*/*S*_1	P1/P2	*exo*	170	156
**7**-*R*/*S*_2	P1A/P2A	*exo*	164	162
**7**-*S*_1	P1A/P2A	*exo*	173	165
**7**-*S*_2A	P1/P2 (C9)	*exo*	175	171
**7**-*S*_2B	P1/P2 (C9')	*exo*	164	111
**8**-*R*_1	P1B/P2B	*exo*	155	117
**8**-*R*_2A	P1A/P2A (C9)	*exo*	173	164
**8**-*R*_2B	P1A/P2A (C9')	*exo*	144	149
**8**-*R*/*S*_1	P1/P2	*exo*	156	115
**8**-*R*/*S*_2A	P1A/P2A (C9A)	*exo*	172	159
**8**-*R*/*S*_2B	P1A/P2A (C9B)	*exo*	140	149
**8**-*S*_1	P1A/P2A	*exo*	167	159
**8**-*S*_2	P1B/P2B	*exo*	156	113
**10**-*R*_1	P1A/P2A	*exo*	155	109
**10**-*R*_2A	P1B/P2B (C9B)	*exo*	154	109
**10**-*R*_2B	P1B/P2B (C9C)	*exo*	166	178
**10**-*S*	P1/P2	*exo*	169	140
**12**-*R*	P1/P2	*exo*	158	129
**12**-*S*_1	P1A/P2A	*exo*	161	128
**12**-*S*_2	P1B/P2B	*exo*	174	158
**13**-*R*	P1/P2	*exo*	157	127
**13**-*S*_1	P1/P2 (C9)	*exo*	157	114
**13**-*S*_2	P1/P2 (C9A)	*exo*	171	160

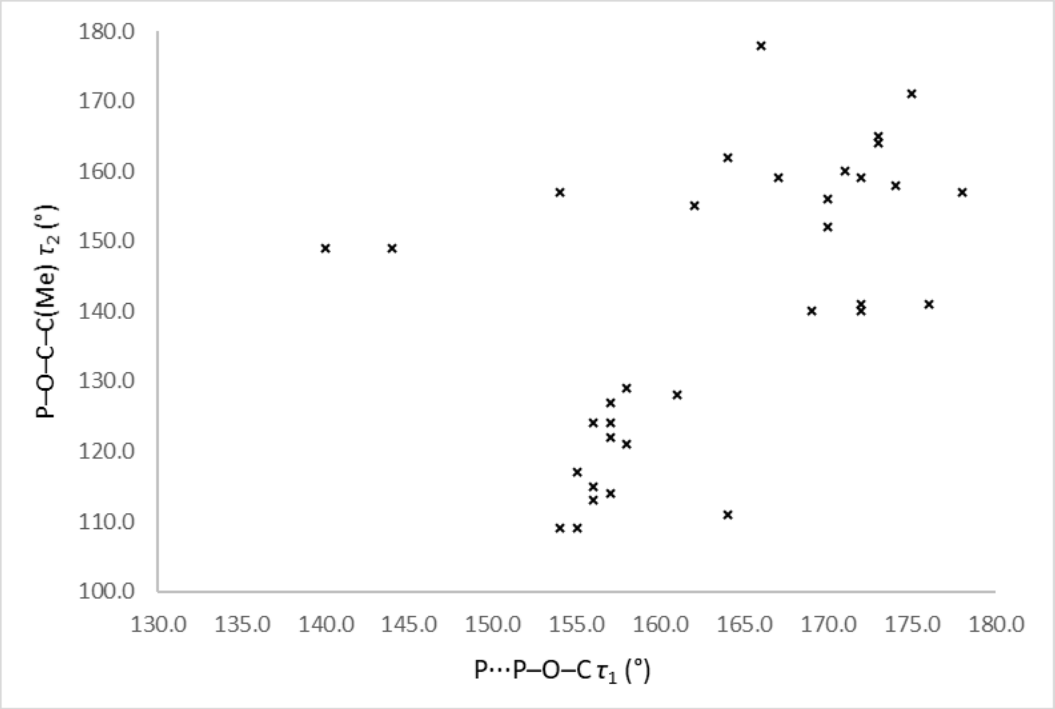

Figure S73. Plot of τ_2 versus τ_1.

The plot of τ_2 versus τ_1 (Figure S73) shows a relatively broad spread overall, but with an obvious cluster centred around $\tau_1 \approx 157°$, $\tau_2 \approx 120°$. This cluster contains representatives from **all** of the molecules showing the Born-*exo* conformation (**2**, **4**, **5**, **6**, **7**, **8**, **10**, **12**, **13**). Hence, the crystal structures demonstrate that this conformation is accessible for all substrates for which structures are determined. It is reasonable to assume that the cluster represents an energetic minimum (at least local, possibly global) for the orientation of the OBorn group.

The similarity between the OBorn conformations observed in corresponding diastereomers can be quantified by the Euclidian distance *d* between their points on the 2D plot: $d = \sqrt{\{(\Delta\tau_1)^2 + (\Delta\tau_2)^2\}}$. A smaller value denotes a greater similarity. For the crystal structures containing multiple independent molecules and/or disorder components, the smallest value (highlighted) for any pair of molecules in the crystal structures indicates the greatest similarity observed for a given pair of diastereomers. This does not necessarily indicate that these conformations are retained in solution, but it indicates whether similar conformations are likely to be accessible.

Table S3. Euclidean distances on the τ_2 versus τ_1 plot for structurally characterised diastereomers.

Molecule 1	Molecule 2	Distance *d* (°)
**4**-*R*_1	**4**-*S*	27.3
**4**-*R*_2	**4**-*S*	27.3
**4**-*R*_3	**4**-*S*	15.5
**5**-*R*	**5**-*S*	24.7
**6**-*R*	**6**-*S*	21.9
**7**-*R*_1	**7**-*S*_1	13.7
**7**-*R*_1	**7**-*S*_2A	20.2
**7**-*R*_1	**7**-*S*_2B	41.6
**7**-*R*_2	**7**-*S*_1	15.2
**7**-*R*_2	**7**-*S*_2A	20.9
**7**-*R*_2	**7**-*S*_2B	44.3

Molecule 1	Molecule 2	Distance *d* (°)
**8**-*R*_1	**8**-*S*_1	44.0
**8**-*R*_1**	**8**-*S*_2**	3.6
**8**-*R*_2A	**8**-*S*_1	8.0
**8**-*R*_2A	**8**-*S*_2	54.1
**8**-*R*_2B	**8**-*S*_1	25.3
**8**-*R*_2B	**8**-*S*_2	38.1
**10**-*R*_1	**10**-*S*	34.4
**10**-*R*_2A	**10**-*S*	34.7
**10**-*R*_2B	**10**-*S*	37.9
**12**-*R*	**12**-*S*_1	2.8
**12**-*R*	**12**-*S*_2	33.5
**13**-*R*	**13**-*S*_1	12.9
**13**-*R*	**13**-*S*_2	36.1

** DFT-optimised structures of **8**-*R* and **8**-*S* show distance *d* = 4.3°.

Considering the quaternised compounds (**5**, **6**, **8**, **10**, **12**, **13**):

Table S4. Minimum Euclidean distances on the τ_2 versus τ_1 plot, ^{31}P NMR P_O chemical shifts, and separations thereof for **5**, **6**, **8**, **10**, **12**, **13**.

**Compound**	**Minimum distance *d* (°) (similarity)**	δ_S	δ_R	$\lvert \delta_S - \delta_R \rvert$
**12**	2.8	122.4	122.4	0.0
**8**	3.6	124.3	124.3	0.0
**13**	12.9	122.2	122.2	0.0
**6**	21.9	123.1	122.6	0.5
**5**	24.7	123.0	122.7	0.3
**10**	34.4	122.5	122.2	0.3

The *R*/*S* diastereomers shown to be able to adopt the most similar OBorn conformations (**8**, **12**, **13**) show the smallest difference between δ_S and δ_R for the P_O resonance in solution. The minimum distance situations arise when both the *R*- and *S*- molecules fall within the cluster on the τ_2 versus τ_1 plot. Where the *R*- and *S*- enantiomers are shown to be able to adopt the OBorn conformation represented by the aforementioned cluster, the difference between δ_S and δ_R for the P_O resonance is small.

The *R*/*S* diastereomers in compounds **5**, **6** and **10** show significantly less similarity in the solid state. For these, the *R*- diastereomer is found amongst the cluster, but the *S*- diastereomer is not. Under these circumstances, the P_O resonance shows the greatest difference between δ_S and δ_R in solution.

We therefore conclude that the conformation represented by the cluster at $\tau_1 \approx 157°$, $\tau_2 \approx 120°$ is likely to be a minimum energy conformation for the OBorn group. It is shown to be accessible for all of the *R*- substrates in the crystal structures determined, but only some of the *S*- substrates. Discrimination between δ_S and δ_R for the P_O resonance arises when the *S*- substrate blocks access to this OBorn conformation.

5. Calculations

The software package Gaussian 16[6] was used for all calculations. Using the crystal structures as starting points for density functional theory (DFT) calculations, the molecules were first optimised at the PBE[7,8] /TZVP[9,10] level of theory without any solvation model applied. The optimised coordinates were then used as the initial structure for optimisation at the PBE/TZVP level of theory with an applied polarised continuum model (PCM)[11] of solvation using the dielectric constant of chloroform. These optimised structures were then further optimised at the B3LYP/TZVP[12,13,14,15] level of theory, again using the PCM model for chloroform. The energetics and vibrational frequencies were calculated for all species of interest. The absence of imaginary frequencies confirms that the obtained geometry corresponds to a ground state structure.

Tables of energies for optimised species/conformers

Table S5. Absolute and Gibbs free energies of optimised species for **4**-*S*, **4**-*R*, **5**-*S*, **8**-*R* and **8**-*S*—note that the calculation for **5**-*R* could not converge despite repeated attempts.

	**Basis / Functional**	**Cis**		**Trans**		**Trans – Cis relative energy / kJ mol^{-1}**
		Energy / Hartree	Gibbs Free Energy / 10^6 kJ mol^{-1}	Energy / Hartree	Gibbs Free Energy / 10^6 kJ mol^{-1}	
**4**-*S*	PBE/ TZVP	-1938.612	-5.088268	-1938.598	-5.088238	29.70
	PBE/ TZVP (PCM)	-1938.616	-5.088279	-1938.603	-5.088250	28.40
	B3LYP/ TZVP (PCM)	-1940.685	-5.093652	-1940.671	-5.093624	26.41
**4**-*R*	PBE/ TZVP	-1938.612	-5.088270	-1938.602	-5.088243	27.34
	PBE/ TZVP (PCM)	-1938.616	-5.088283	-1938.606	-5.088256	26.93
	B3LYP/ TZVP (PCM)	-1940.684	-5.093653	-1940.676	-5.093630	23.69
**5**-*S*	PBE/ TZVP	-1978.289	-5.192338	-1978.283	-5.192326	12.48
	PBE/ TZVP (PCM)	-1978.335	-5.192450	-1978.328	-5.192439	11.12
	B3LYP/ TZVP (PCM)	-1980.469	-5.197990	-1980.461	-5.197979	10.28
**8**-*R*	PBE/ TZVP	-2017.557	-5.295375	-2017.550	-5.295361	14.37
	PBE/ TZVP (PCM)	-2017.604	-5.295489	-2017.596	-5.295474	14.84
	B3LYP/ TZVP (PCM)	-2019.796	-5.301178	-2019.788	-5.301165	12.40
**8**-*S*	PBE/ TZVP	-2017.554	-5.295372	-2017.548	-5.295358	13.72
	PBE/ TZVP (PCM)	-2017.601	-5.295480	-2017.595	-5.295471	9.67
	B3LYP/ TZVP (PCM)	-2019.793	-5.301172	-2019.786	-5.3011631	8.75

Table S6. Absolute, Gibbs Free Energies and Relative Energies of Conformers of **4**-*S*

Conformation	O_{exo} or O_{endo}?	N_{exo} or N_{endo}?	Energy / Hartree	Gibbs Free Energy / 10^6 kJ mol^{-1}	Relative Energy / kJ mol^{-1}
**Cis**	exo	endo	-1940.684679	-5.093651	0.00
**Cis**	exo	exo	-1940.676796	-5.093641	9.33
**Trans**	exo	exo	-1940.671145	-5.093624	26.41
**Trans**	endo	endo	-1940.669472	-5.093680	-28.97
**Trans**	exo	endo	-1940.673936	-5.093625	25.66
**Trans**	exo	exo	-1940.674096	-5.093631	19.93

Optimised structures and NMR calculations for 4-*S*

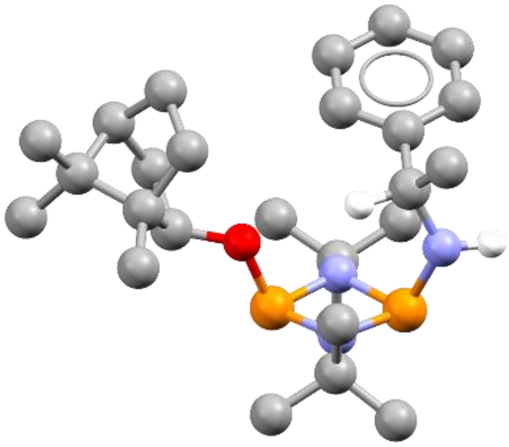

Figure S74. Optimised Structure of *cis*-N_{endo}-O_{exo}-**4**-*S* (the same as the experimentally observed structure).

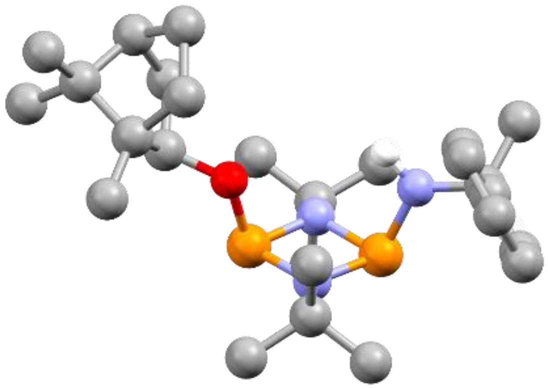

Figure S75. Optimised Structure of *cis*-N_{exo}-O_{exo}-**4**-*S*.

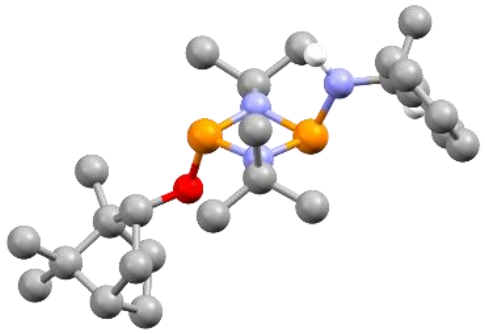

Figure S76. Optimised Structure of *trans*-N_{exo}-O_{exo}-**4**-*S*.

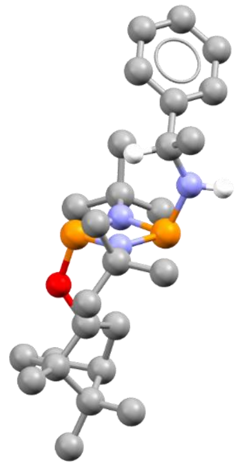

Figure S77. Optimised Structure of *trans*-N_{endo}-O_{endo}-**4**-*S*.

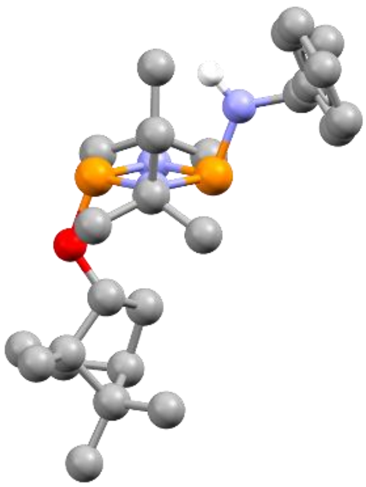

Figure S78. Optimised Structure of *trans*-N_{exo}-O_{endo}-**4**-*S*.

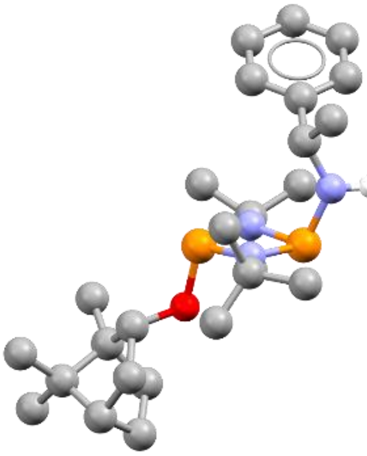

Figure S79. Optimised Structure of *trans*-N_{endo}-O_{exo}-**4**-*S*.

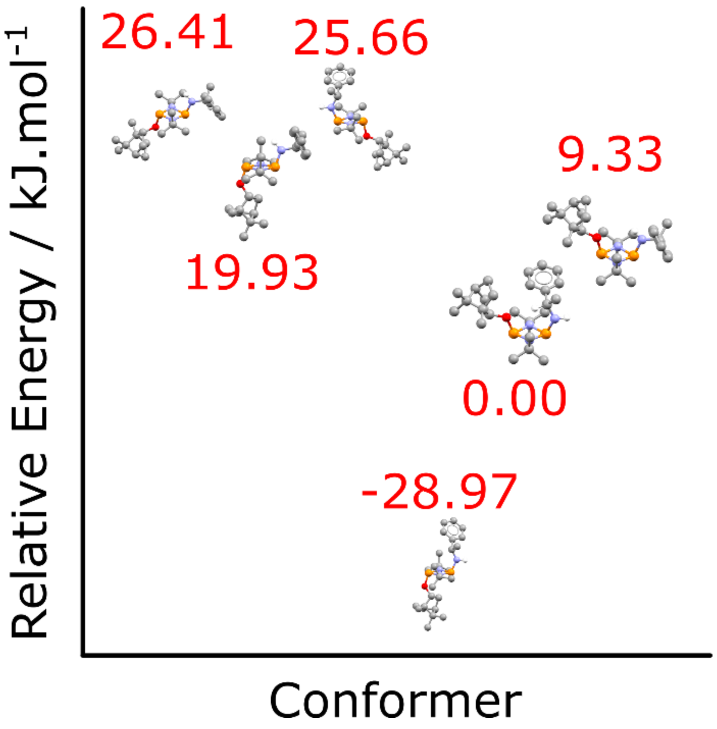

Figure S80. Relative Gibb's free energies of the isomers of **4**-*S* (values are in kJ mol^{-1}).

Table S7. Variation of energy upon rotation of the C–N bond in **4**-*S*

Dihedral Angle (P1–N59–C61–C67) /°	Energies/Hartree (PBE/TZVP)	Relative Energy / kJ mol^{-1}
**269.4043**	-1938.54256	0.00000
**284.4043**	-1938.54107	3.91199
**299.4043**	-1938.53759	13.04872
**314.4043**	-1938.53283	25.54608
**329.4043**	-1938.52832	37.38707
**344.4043**	-1938.52638	42.48054
**359.4043**	-1938.52535	45.18480
**374.4043**	-1938.52520	45.57862
**389.4043**	-1938.52666	41.74540
**404.4043**	-1938.52996	33.08126
**419.4043**	-1938.53324	24.46963
**434.4043**	-1938.53486	20.21633
**449.4043**	-1938.53519	19.34991

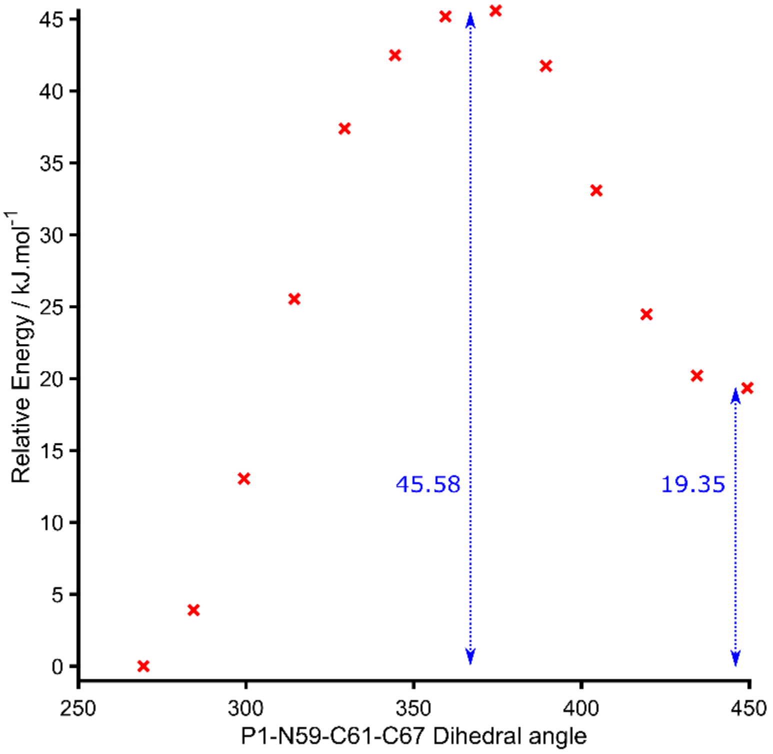

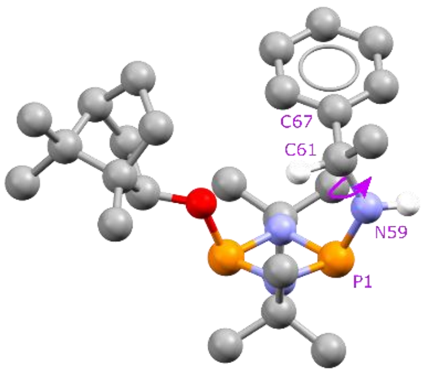

Figure S81. Graph showing the relative variation of energy in kJ mol^{-1} upon rotation of the C–N bond in **4**-*S.*

Optimised structures and NMR calculations for 4-*R*

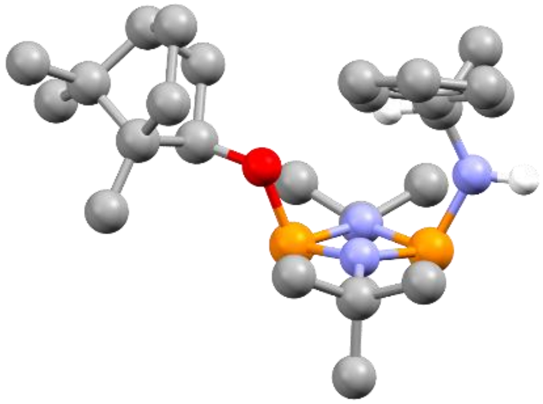

Figure S82. Optimised Structure of *cis*-N_{endo}-O_{exo}-**4**-*R* (the same as the experimentally observed structure).

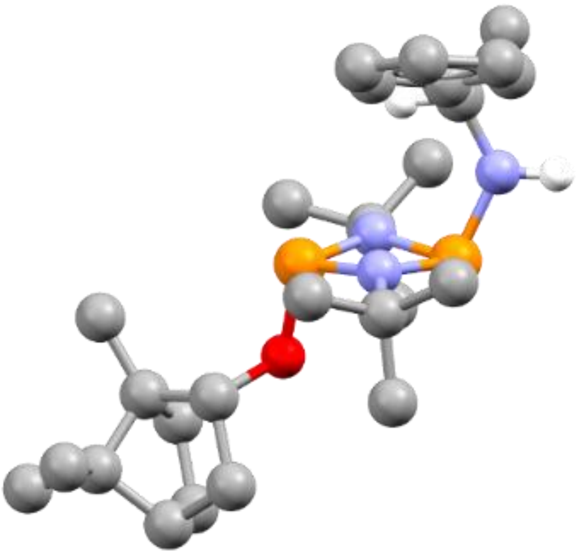

Figure S83. Optimised Structure of *trans*-N_{endo}-O_{exo}-**4**-*R*

Optimised structures for 5-*S*

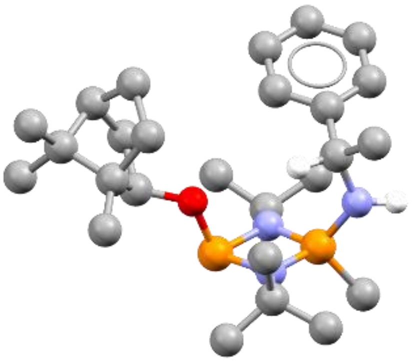

Figure S84. Optimised Structure of *cis*-N_{endo}-O_{exo}-**5**-*S* (the same as the experimentally observed structure).

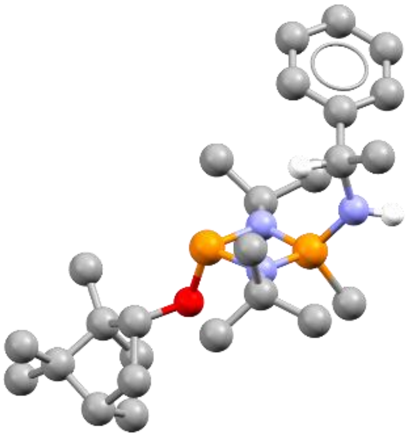

Figure S85. Optimised Structure of *trans*-N_{endo}-O_{exo}-**5**-*S.*

Optimised structures for 8-*R*

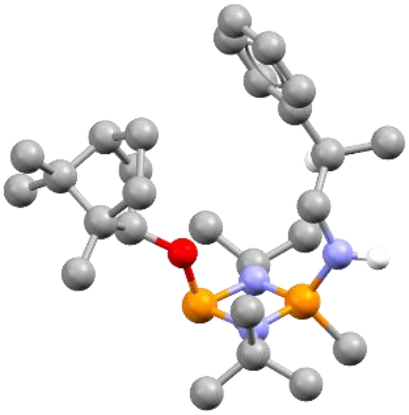

Figure S86. Optimised Structure of *cis*-N_{endo}-O_{exo}-**8**-*R* (the same as the experimentally observed structure).

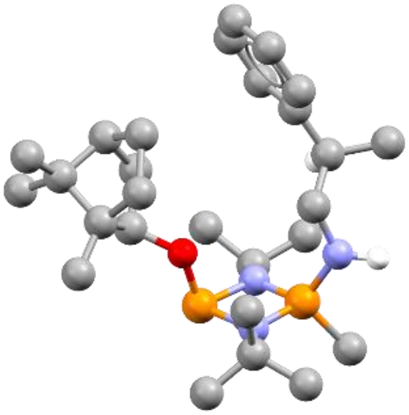

Figure S87. Optimised Structure of *trans*-N_{endo}-O_{exo}-**8**-*R*.

Optimised structures for 8-*S*

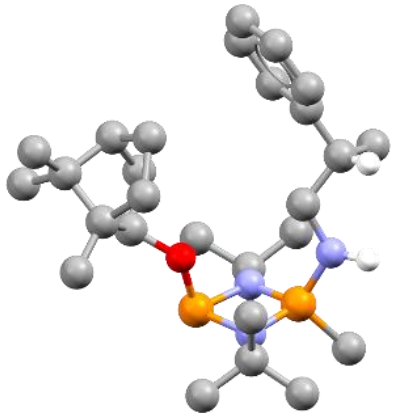

Figure S88. Optimised Structure of *cis*-N_{endo}-O_{exo}-**8**-*S* (the same as the experimentally observed structure).

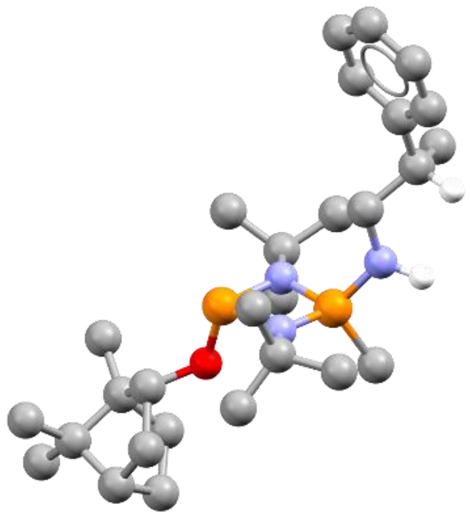

Figure S89. Optimised Structure of *trans*-N_{endo}-O_{exo}-**8**-*S.*

Table S8. Data for geometry optimised structures of **5**-*R* and **5**-*S*, (a) Wiberg bond orders and bond orders, (b) the calculated dihedral C-O-P⋯P angles for the *R* and *S* diastereomers (for comparison, the experimental values are **5**-*S* *ca.* 171.5°, *cf*. **5**-R *ca.* 157.7°), (c) orbital occupancies at the P atoms, and (d) calculated P chemical shifts. Calculations were carried out at the B3LYP/TZVP level of theory with implicit solvation (pcm, chloroform).

(a)

Diastereomer	Bond	Occupancy	Energy	Wiberg Bond Order	Atom overlap-weighted Bond Order
**5**-*S*	P2-O3	1.97588	-0.69026	0.6935	0.5511
**5**-*S*	P2-N4 (Ring)	1.97116	-0.64832	0.8225	0.7150
**5**-*S*	P2-N5 (Ring)	1.96513	-0.61864	0.8138	0.6974
**5**-*S*	P1-N4 (Ring)	1.96885	-0.63316	0.7713	0.6768
**5**-*S*	P1-N5 (Ring)	1.96041	-0.59934	0.7593	0.6580
**5**-*S*	P1-N59 (Arm)	1.97945	-0.63316	0.9002	0.7499
**5**-*R*	P3-O1	1.97626	-0.69515	0.6990	0.5540
**5**-*R*	P3-N4 (Ring)	1.96836	-0.63350	0.8348	0.7175
**5**-*R*	P3-N5 (Ring)	1.96717	-0.61663	0.7995	0.6846
**5**-*R*	P2-N4 (Ring)	1.96356	-0.60253	0.7544	0.6540
**5**-*R*	P2-N5 (Ring)	1.96370	-0.61263	0.7747	0.6732
**5**-*R*	P2-N6 (Arm)	1.97996	-0.68261	0.9111	0.7560

(b)

Diastereomer	C-O-P⋯P dihedral angle ϑ / °
**5**-*S*	172.53447
**5**-*R*	150.31378

(c)

Occupancy	**5**-*S* **P1**	**5**-*S* **P2**	**5**-*R* **P2**	**5**-*R* **P3**
**3s**	1.43	1.47	1.43	1.48
**3p**	2.24	2.11	2.25	2.11
**4s**	0.01	0.01	0.01	0.01
**3d**	0.04	0.04	0.04	0.04
**4p**	0.02	0.02	0.02	0.02

(d)

Phosphorus atom	Chemical shift (unreferenced)	Chemical shift (relative to H_3PO_4)
**5**-*S* **P1**	189.4711	113.8184
**5**-*S* **P2**	130.5430	172.7465
**5**-*R* **P2**	201.4356	101.8539
**5**-*R* **P3**	144.4764	158.8131

Bibliography

[1] A. Bashall, E. L. Doyle, C. Tubb, S. J. Kidd, M. McPartlin, A. D. Woods, and D. S. Wright, *Chem. Commun.*, 2001, 2542–2543.
[2] G. R. Fulmer, A. J. M. Miller, N. H. Sherden, H. E. Gottlieb, A. Nudelman, Brian M. Stoltz, J. E. Bercaw, and K. I. Goldberg, *Organometallics*, 2010, **29,** 2176–2179.
[3] R. K. Harris, E. D. Becker, S. M. Cabral de Menezes, R. Goodfellow and P. Granger, *Pure Appl. Chem*., 2001, **73**, 1795–1818.
[4] G. M. Sheldrick, *Acta Crystallogr. Sect. A*, 2015, **71**, 3–8.
[5] G. M. Sheldrick, *Acta Crystallogr. Sect. C*, 2015, **71**, 3–8.
[6] M. J. Frisch *et al.* Gaussian. (2016).
[7] J. P. Perdew, K. Burke and M. Ernzerhof, *Phys. Rev. Lett.,* 1996, **77**, 3865–3868.
[8] J. P. Perdew, K. Burke, and M. Ernzerhof, *Phys. Rev. Lett.*, 1997, **78**, 1396.
[9] A. Schäfer, C. Huber and R. Ahlrichs, *J. Chem. Phys.*, 1994, **100**, 5829–5835.
[10] A. Schäfer, H. Horn, R. Ahlrichs, *J. Chem. Phys.*, 1992, **97**, 2571–2577.
[11] G. Scalmani, and M. J. Frisch, *J. Chem. Phys.*, 2010, **132**, 114110–114125.
[12] A. D. Becke, *J. Chem. Phys.*, 1993, **98**, 5648–5652.
[13] P. J. Stephens, F. J. Devlin, C. F. Chabalowski and M. J. Frisch, *J. Phys. Chem.*, 1994, **98**, 11623–11627.
[14] S. H. Vosko, S.A. And, L.W. Ilk and M. Nusair, *Can. J. Phys.*, 1980, **58**, 1200–1211.
[15] C. Lee, W. Yang, and R. G. Parr, *Phys. Rev. B,* **1988**, 37, 785–789.
