# Supplementary material for: A chiral phosphazane reagent strategy for the determination of enantiomeric excess of amines
Source: Chem Sci. 2022 Apr 25;13(18):5398–412. doi: 10.1039/d2sc01692c (PMC9093139; doi:10.1039/d2sc01692c)
Supplement: SC-013-D2SC01692C-s002 [file SC-013-D2SC01692C-s002.pdf]

Electronic Supplementary Material (ESI) for Chemical Science.
This journal is © The Royal Society of Chemistry 2022

A chiral phosphazane reagent strategy for the determination of enantiomeric excess of amines

Andrew J. Peel,[a,*] Alexandros Terzopoulos,[a] Rajesh B. Jethwa,[a] Dipanjana Choudhury,[a] Hao-Che Niu,[a] Andrew D. Bond,[a] Jonathan Slaughter[a] and Dominic S. Wright[a,*]

[a] *Yusuf Hamied Department of Chemistry, Cambridge University, Lensfield Road, Cambridge CB2 1EW (U.K.)*

Supplementary Information Addendum:
Coordinates used in Calculations (ESI, Section 5)

Table of Contents

H_3PO_4 2

4-*S* 2

cis-N_{endo}-O_{exo}-**4**-*S* 2

cis-N_{exo}-O_{exo}-**4**-*S* 5

trans-N_{exo}-O_{exo}-**4**-*S* 8

trans-N_{endo}-O_{endo}-**4**-*S* 10

trans-N_{endo}-O_{exo}-**4**-*S* 12

trans-N_{exo}-O_{endo}-**4**-*S* 14

4-*R* 16

cis-N_{endo}-O_{exo}-**4**-*R* 16

trans-N_{endo}-O_{exo}-**4**-*R* 18

5-*S* 20

cis-N_{endo}-O_{exo}-**5**-*S* 20

trans-N_{endo}-O_{exo}-**5**-*S* 22

8-*R* 24

cis-N_{endo}-O_{exo}-**8**-*R* 24

trans-N_{endo}-O_{exo}-**8**-*R* 26

8-*S* 28

cis-N_{endo}-O_{exo}-**8**-*S* 28

trans-N_{endo}-O_{exo}-**8**-*S* 30

H_3PO_4

Element	PBE/TZVP			B3LYP/TZVP		
	x	y	z	x	y	Z
**P**	0.000015	-1E-06	-0.12439	0.000012	-0.00001	0.118561
**O**	0.000219	-3E-06	-1.62486	0.000128	-0.00016	1.604821
**O**	-0.35209	1.415866	0.607693	-1.28874	-0.66269	-0.59703
**H**	-1.11814	-1.83811	0.092857	0.629262	2.055454	-0.09563
**O**	1.402107	-0.40309	0.607911	1.218259	-0.7846	-0.59725
**H**	-1.03282	1.887362	0.092865	-2.0947	-0.48276	-0.09563
**O**	-1.05025	-1.01278	0.607611	0.070335	1.447469	-0.59694
**H**	2.150876	-0.04919	0.093277	1.465417	-1.57269	-0.09606

Note: to obtain a NMR reference value for the phosphorous atoms, a single-point calculation was done under an implicitly solvated environment ($CHCl_3$). The NMR referencing was carried out by subtracting the shift of the atom of interest away from the shift of phosphoric acid.

4-*S*

cis-N_{endo}-O_{exo}-**4**-*S*

Element	PBE/TZVP			PBE/TZVP (CHCl3)			B3LYP/TZVP (CHCl3)		
	X	y	z	x	y	z	x	y	z
**P**	2.552819	1.519292	0.331108	2.539750	1.531442	0.335028	2.564183	1.486841	0.278179
**P**	0.045086	1.311458	1.230927	0.031141	1.313742	1.228029	0.109008	1.303331	1.274898
**O**	-0.946555	0.336775	0.214560	-0.950654	0.329967	0.217529	-0.906237	0.382664	0.272103
**N**	0.960163	2.230908	0.076966	0.945130	2.236657	0.076425	0.996395	2.256891	0.143981
**N**	1.550872	0.416985	1.312367	1.541644	0.428633	1.319189	1.600238	0.407613	1.299662
**C**	0.562396	3.413600	-0.706339	0.541549	3.414648	-0.712932	0.568094	3.420387	-0.649226
**C**	-0.534927	4.180033	0.050469	-0.558792	4.179855	0.040671	-0.569527	4.144880	0.085196
**H**	-1.422053	3.550778	0.217514	-1.442885	3.547195	0.211511	-1.435250	3.494957	0.224101
**H**	-0.851792	5.058160	-0.531294	-0.879850	5.052652	-0.546533	-0.894926	5.009377	-0.496497
**H**	-0.168272	4.519518	1.029806	-0.192276	4.527803	1.017273	-0.239212	4.490218	1.066445
**C**	1.780585	4.330692	-0.898531	1.755036	4.336925	-0.909357	1.748505	4.387260	-0.819451
**H**	2.589243	3.807578	-1.430523	2.566875	3.815458	-1.438262	2.578626	3.909360	-1.343496
**H**	2.170556	4.674810	0.070176	2.141295	4.690062	0.057782	2.108212	4.734898	0.150649
**H**	1.496056	5.210068	-1.494946	1.465187	5.210744	-1.511091	1.437165	5.253494	-1.406624
**C**	0.031710	2.965659	-2.083464	0.012316	2.959337	-2.087970	0.080938	2.953952	-2.032412
**H**	0.830226	2.473956	-2.655790	0.811223	2.465896	-2.658295	0.904422	2.506024	-2.587092
**H**	-0.332138	3.825816	-2.666512	-0.351781	3.817485	-2.673534	-0.309997	3.793001	-2.613237
**H**	-0.791053	2.247889	-1.961261	-0.810378	2.241967	-1.962785	-0.702555	2.204236	-1.924382
**C**	2.033151	-0.302093	2.514417	2.023373	-0.287287	2.523497	2.111346	-0.375767	2.445990
**C**	3.303314	-1.077778	2.130754	3.291081	-1.067608	2.141003	3.331270	-1.183207	1.980270
**H**	3.100339	-1.777784	1.309779	3.083404	-1.766242	1.319736	3.066261	-1.839660	1.153172

H	3.674924	-1.644957	2.997031	3.660183	-1.635276	3.007958	3.711727	-1.790844	2.804001
H	4.103018	-0.390970	1.812105	4.093239	-0.384515	1.820365	4.139432	-0.524605	1.653185
C	0.942162	-1.295351	2.943082	0.931435	-1.276683	2.959180	1.013200	-1.348550	2.897023
H	0.724182	-1.998405	2.127846	0.709923	-1.984372	2.148726	0.734848	-2.011077	2.078589
H	0.012067	-0.770465	3.212504	0.002067	-0.749743	3.227089	0.120617	-0.814450	3.231387
H	1.271792	-1.866397	3.823708	1.262783	-1.842609	3.842387	1.369525	-1.955172	3.732143
C	2.342668	0.670226	3.669698	2.337179	0.687386	3.675684	2.510541	0.538939	3.618284
H	1.439615	1.230899	3.954827	1.435825	1.250987	3.960653	1.650205	1.113687	3.968048
H	3.115616	1.392655	3.366940	3.111813	1.407009	3.370277	3.287953	1.240857	3.309139
H	2.706571	0.128784	4.556775	2.700560	0.146443	4.563151	2.893694	-0.047221	4.457303
C	-2.253299	-0.008819	0.714379	-2.258942	-0.022420	0.713660	-2.219841	0.053309	0.750281
H	-2.470955	0.648379	1.575396	-2.481083	0.631087	1.575948	-2.462231	0.740521	1.565322
C	-2.377069	-1.509976	1.115501	-2.375833	-1.524997	1.109608	-2.351784	-1.424804	1.218829
H	-1.443554	-2.043863	0.893211	-1.440624	-2.054903	0.884270	-1.424186	-1.966156	1.043487
H	-2.565002	-1.623476	2.193041	-2.563183	-1.641871	2.186763	-2.562282	-1.484493	2.287160
C	-3.555596	-2.012171	0.255791	-3.552466	-2.029266	0.248177	-3.513541	-1.965156	0.360187
H	-4.028158	-2.931434	0.632111	-4.022210	-2.950738	0.622342	-3.994544	-2.854399	0.770423
C	-4.487771	-0.762833	0.203568	-4.488806	-0.783077	0.198336	-4.436461	-0.719847	0.221405
C	-3.355821	0.170788	-0.367127	-3.359650	0.156166	-0.369139	-3.288570	0.177041	-0.365633
C	-2.883234	-0.620818	-1.614619	-2.883058	-0.630920	-1.618097	-2.788888	-0.676984	-1.558870
H	-1.847360	-0.358504	-1.867755	-1.847584	-0.364678	-1.869422	-1.754570	-0.435924	-1.798283
H	-3.503593	-0.367459	-2.486917	-3.504339	-0.377680	-2.489741	-3.382108	-0.468631	-2.450263
C	-3.045502	-2.113684	-1.198963	-3.041123	-2.125535	-1.206493	-2.971762	-2.145493	-1.073518
H	-3.765692	-2.638796	-1.842836	-3.759718	-2.650223	-1.852511	-3.677569	-2.690642	-1.701210
H	-2.100430	-2.673236	-1.256303	-2.094193	-2.682286	-1.263225	-2.038660	-2.709749	-1.082018
C	-5.040752	-0.338460	1.573225	-5.044364	-0.364403	1.568842	-5.026641	-0.227798	1.552584
H	-5.760908	-1.088344	1.936031	-5.764231	-1.117134	1.926345	-5.755458	-0.952932	1.923565
H	-5.579805	0.618168	1.492374	-5.583806	0.592183	1.489950	-5.552264	0.719403	1.409433
H	-4.273236	-0.220757	2.348249	-4.278221	-0.249361	2.345719	-4.290466	-0.077685	2.339807
C	-5.690130	-0.921396	-0.737326	-5.689869	-0.943090	-0.744138	-5.619420	-0.922119	-0.735670
H	-6.359000	-1.712549	-0.363583	-6.355392	-1.737940	-0.372188	-6.285527	-1.695145	-0.343437
H	-5.412448	-1.180109	-1.766509	-5.410475	-1.197658	-1.773966	-5.327081	-1.218487	-1.740810
H	-6.276089	0.009918	-0.775878	-6.278445	-0.013277	-0.779824	-6.201625	-0.001517	-0.821467
C	-3.721800	1.617526	-0.642846	-3.731655	1.602071	-0.641591	-3.641093	1.606427	-0.726569
H	-2.860832	2.166350	-1.051459	-2.873116	2.155472	-1.049367	-2.776816	2.122867	-1.145757
H	-4.042249	2.135779	0.274694	-4.053784	2.116430	0.277487	-3.973581	2.169648	0.149117
H	-4.536502	1.689121	-1.379506	-4.547198	1.670823	-1.377574	-4.437463	1.636956	-1.473610
N	2.891197	0.700656	-1.142408	2.895390	0.710720	-1.129559	2.797291	0.696124	-1.216744
H	3.884016	0.630001	-1.360303	3.889530	0.652724	-1.346908	3.767695	0.599161	-1.483470
C	2.013384	-0.223140	-1.872919	2.026583	-0.216391	-1.867261	1.867106	-0.208953	-1.902514
H	0.985506	0.101664	-1.648068	0.995167	0.098725	-1.645337	0.865164	0.128252	-1.636243
C	2.251745	-0.099333	-3.383186	2.270686	-0.085652	-3.376074	2.036787	-0.092081	-3.420645
H	2.097421	0.936802	-3.715786	2.106911	0.949934	-3.705961	1.878523	0.935895	-3.747627
H	1.566543	-0.756973	-3.937126	1.592878	-0.746880	-3.934969	1.322508	-0.738171	-3.934368
H	3.280262	-0.393906	-3.645203	3.303474	-0.368265	-3.633249	3.041451	-0.394879	-3.726014
C	2.144321	-1.668130	-1.407433	2.168703	-1.661806	-1.405551	2.003619	-1.651580	-1.439456

**C**	3.362473	-2.358256	-1.487857	3.395781	-2.338005	-1.479121	3.193361	-2.362088	-1.610872
**H**	4.249758	-1.857205	-1.885179	4.280099	-1.825423	-1.867576	4.047038	-1.885693	-2.079713
**C**	3.464870	-3.680254	-1.048014	3.509441	-3.660533	-1.041769	3.306597	-3.675932	-1.167982
**H**	4.422620	-4.201453	-1.110325	4.473530	-4.170702	-1.098449	4.239009	-4.211550	-1.301114
**C**	2.344009	-4.336259	-0.527188	2.391807	-4.330856	-0.530213	2.224720	-4.302708	-0.551977
**H**	2.424271	-5.368621	-0.181044	2.481252	-5.362988	-0.185369	2.313068	-5.324864	-0.204532
**C**	1.123797	-3.658751	-0.450190	1.162898	-3.667104	-0.460377	1.033601	-3.603147	-0.382715
**H**	0.243642	-4.159937	-0.039628	0.285696	-4.178705	-0.056530	0.188866	-4.077976	0.103003
**C**	1.028683	-2.334770	-0.887068	1.056329	-2.342873	-0.895879	0.928606	-2.287064	-0.823047
**H**	0.090901	-1.784290	-0.792756	0.110756	-1.804462	-0.807708	0.022338	-1.722654	-0.656425

cis-N_{exo}-O_{exo}-**4**-*S*

Element	PBE/TZVP			PBE/TZVP (CHCl3)			B3LYP/TZVP (CHCl3)		
	X	y	z	x	y	z	x	y	z
**P**	-1.841153	1.656902	0.463968	-1.828412	1.601768	0.512210	-1.818477	1.690447	0.448875
**P**	0.716472	1.329258	1.181119	0.737767	1.242032	1.179243	0.707424	1.370331	1.210784
**O**	1.404888	0.122414	0.171577	1.459315	0.108652	0.112408	1.373242	0.150509	0.240404
**N**	-0.833329	0.641336	1.511065	-0.804447	0.520433	1.473912	-0.846190	0.716681	1.551801
**N**	-0.236397	2.279656	0.042369	-0.227396	2.237425	0.089492	-0.218080	2.320068	0.074942
**C**	-1.193633	-0.505311	2.361106	-1.156050	-0.664192	2.275581	-1.219917	-0.443436	2.377144
**C**	-0.257131	-0.534525	3.580065	-0.209981	-0.750389	3.484268	-0.305351	-0.496823	3.609576
**H**	0.794837	-0.637215	3.272667	0.840144	-0.832623	3.165013	0.742135	-0.613005	3.323865
**H**	-0.502428	-1.390773	4.225593	-0.447419	-1.639306	4.086937	-0.573749	-1.348793	4.237334
**H**	-0.352491	0.390269	4.167222	-0.306369	0.142614	4.118833	-0.398933	0.416328	4.200275
**C**	-2.644034	-0.331241	2.837967	-2.602564	-0.517702	2.773081	-2.674666	-0.278413	2.838072
**H**	-3.341686	-0.317100	1.987401	-3.309571	-0.465587	1.931647	-3.359198	-0.257162	1.988448
**H**	-2.762262	0.604030	3.403866	-2.717550	0.388283	3.385711	-2.799615	0.643049	3.409668
**H**	-2.929396	-1.174817	3.483505	-2.877656	-1.391779	3.381337	-2.961622	-1.122725	3.467840
**C**	-1.064136	-1.821626	1.567666	-1.031021	-1.939680	1.417089	-1.080636	-1.745621	1.567911
**H**	-1.745716	-1.821590	0.706202	-1.720136	-1.895416	0.562181	-1.740519	-1.729027	0.701235
**H**	-1.313428	-2.686789	2.201503	-1.274192	-2.835169	2.009818	-1.158181	-2.769441	2.049010
**H**	-0.038657	-1.940874	1.192485	-0.007871	-2.038783	1.028916	0.102814	-1.953853	1.102312
**C**	0.043337	3.682608	-0.332826	0.049451	3.660529	-0.215131	0.190862	3.636433	-0.286973
**C**	-0.896646	4.067912	-1.486925	-0.900273	4.105931	-1.339049	-0.739262	3.974602	-1.461765
**H**	-0.770211	3.380362	-2.334941	-0.777603	3.468577	-2.226056	-0.609684	3.255448	-2.271972
**H**	-0.683693	5.093718	-1.822808	-0.690819	5.149209	-1.618014	-0.519057	4.974721	-1.840715
**H**	-1.949727	4.026849	-1.169249	-1.951187	4.045468	-1.017132	-1.787284	3.957851	-1.153856
**C**	1.501342	3.761677	-0.812690	1.503191	3.765730	-0.702616	1.647963	3.690352	-0.768344
**H**	1.663966	3.090843	-1.667556	1.660505	3.140427	-1.592370	1.812060	2.975474	-1.574937
**H**	2.197418	3.473458	-0.009237	2.207548	3.439021	0.078627	2.341764	3.457207	0.042853
**H**	1.747913	4.789462	-1.117377	1.744099	4.808417	-0.956552	1.887377	4.691289	-1.132987
**C**	-0.168560	4.642426	0.854685	-0.151837	4.557708	1.021911	-0.018304	4.651374	0.851306
**H**	0.505082	4.381332	1.684649	0.528450	4.254563	1.832058	0.641115	4.423567	1.691459
**H**	-1.204926	4.578325	1.219065	-1.185561	4.477328	1.390779	-1.049859	4.619101	1.207952
**H**	0.030081	5.685781	0.564097	0.046637	5.613626	0.781842	0.195560	5.668609	0.513756
**C**	2.838519	0.062937	0.079064	2.899148	0.058712	0.067024	2.919422	0.001910	0.073537
**H**	3.261083	0.699274	0.877923	3.287617	0.658817	0.909342	3.375604	0.613068	0.857755
**C**	3.368012	0.493336	-1.321580	3.472834	0.560639	-1.291035	3.409182	0.446012	-

									1.333985
**H**	2.522713	0.707654	-1.990546	2.651989	0.799034	-1.981908	2.554807	0.668116	-1.973110
**H**	3.980180	1.404817	-1.260764	4.071635	1.473840	-1.162753	4.021653	1.346447	-1.279230
**C**	4.180048	-0.733886	-1.786427	4.315270	-0.635017	-1.783495	4.202857	-0.779012	-1.833903
**H**	4.916876	-0.515337	-2.573412	5.078151	-0.371556	-2.530746	4.918720	-0.553714	-2.625860
**C**	4.780140	-1.267477	-0.448396	4.871686	-1.226511	-0.451214	4.825505	-1.338583	-0.520857
**C**	3.370257	-1.389359	0.240017	3.438478	-1.395037	0.177879	3.432855	-1.455438	0.194582
**C**	2.587645	-2.260285	-0.777521	2.704919	-2.223958	-0.908513	2.619587	-2.302281	-0.817362
**H**	1.508981	-2.076816	-0.690363	1.621174	-2.058139	-0.851860	1.553923	-2.111888	-0.709391
**H**	2.754647	-3.328528	-0.574677	2.879392	-3.298601	-0.751096	2.778724	-3.366296	-0.636405
**C**	3.160514	-1.832353	-2.161512	3.322484	-1.723679	-2.248637	3.168373	-1.861715	-2.206233
**H**	3.644346	-2.672434	-2.680642	3.834558	-2.533761	-2.787861	3.631980	-2.692953	-2.738967
**H**	2.383618	-1.441671	-2.835026	2.566274	-1.308222	-2.930897	2.387754	-1.459787	-2.854289
**C**	5.747223	-0.288216	0.235456	5.802216	-0.272306	0.314256	5.822792	-0.382618	0.152924
**H**	6.674078	-0.203100	-0.353086	6.749642	-0.153652	-0.234360	6.730625	-0.310250	-0.451453
**H**	6.028365	-0.657134	1.234058	6.047622	-0.685621	1.304867	6.113623	-0.764674	1.134497
**H**	5.344398	0.724821	0.358573	5.385623	0.731019	0.467847	5.446976	0.628965	0.293295
**C**	5.518215	-2.606079	-0.587088	5.629203	-2.550073	-0.625981	5.550731	-2.680345	-0.694256
**H**	6.405613	-2.482914	-1.227747	6.538346	-2.386031	-1.225673	6.411926	-2.554357	-1.355811
**H**	4.903109	-3.403933	-1.021024	5.040161	-3.331757	-1.121355	4.926511	-3.467139	-1.112552
**H**	5.870913	-2.956106	0.395415	5.948321	-2.943533	0.351577	5.926588	-3.036639	0.267923
**C**	3.325829	-1.903682	1.667235	3.350543	-1.980080	1.575547	3.413187	-1.993013	1.611852
**H**	2.287832	-1.949912	2.029425	2.300936	-2.065473	1.894601	2.390401	-2.034375	1.991063
**H**	3.888579	-1.249628	2.351658	3.873223	-1.350072	2.312239	3.993979	-1.360501	2.287910
**H**	3.744814	-2.918986	1.739554	3.788810	-2.989104	1.613055	3.822504	-3.004884	1.654954
**N**	-2.204456	0.789823	-0.974241	-2.257376	0.817387	-0.951204	-2.167226	0.579333	-0.750360
**H**	-3.564772	0.451003	-1.394396	-3.643520	0.549786	-1.343917	-3.467005	0.517273	-1.431789
**C**	-4.229660	1.108600	-0.807855	-4.264538	1.219565	-0.724718	-4.060731	1.345166	-1.035319
**H**	-3.766659	0.765504	-2.883251	-3.866463	0.910210	-2.818880	-3.308984	0.719264	-2.941088
**C**	-3.553953	1.826374	-3.075688	-3.613557	1.965777	-2.991765	-2.842718	1.684854	-3.141986
**H**	-4.797120	0.540988	-3.195351	-4.913870	0.740243	-3.107781	-4.279962	0.682725	-3.438973
**H**	-3.088701	0.162667	-3.506871	-3.231866	0.292962	-3.473139	-2.678801	-0.060437	-3.375147
**H**	-3.936681	-0.989206	-1.056699	-4.071431	-0.881282	-1.033283	-4.191184	-0.775040	-1.080746
**C**	-3.244650	-2.066498	-1.632062	-3.425783	-1.974378	-1.633195	-3.660624	-2.015199	-1.446273
**C**	-2.442710	-1.877488	-2.350981	-2.617531	-1.805027	-2.349731	-2.738009	-2.062389	-2.013302

**H**	-3.554478	-3.382877	-1.286659	-3.791888	-3.283960	-1.315208	-4.293145	-3.196848	-1.078263
**C**	-2.999745	-4.207682	-1.738850	-3.273365	-4.121844	-1.786042	-3.864433	-4.149426	-1.365724
**H**	-4.568075	-3.645966	-0.358676	-4.815777	-3.523536	-0.391063	-5.473388	-3.158153	-0.337621
**C**	-4.807977	-4.675167	-0.084870	-5.099312	-4.547240	-0.138921	-5.965654	-4.078584	-0.048382
**H**	-5.270196	-2.583167	0.214034	-5.472179	-2.443700	0.205340	-6.012609	-1.929488	0.028355
**C**	-6.063280	-2.777617	0.939176	-6.272327	-2.619437	0.927536	-6.928241	-1.888000	0.606063
**H**	-4.955035	-1.266196	-0.135752	-5.100750	-1.133575	-0.116596	-5.372736	-0.748010	-0.341472
**C**	-5.503028	-0.437117	0.320766	-5.612186	-0.292097	0.359079	-5.793591	0.205763	-0.042758
**H**	-1.446626	0.266979	-1.418174	-1.536119	0.296961	-1.454558	-1.449701	-0.024819	-1.133850

trans-N_{exo}-O_{exo}-**4**-*S*

Element	PBE/TZVP			PBE/TZVP (CHCl3)			B3LYP/TZVP (CHCl3)		
	X	y	z	x	y	z	x	y	z
**P**	-1.376580	0.700328	0.592807	-1.391347	0.708828	0.587367	-1.388210	0.713037	0.623473
**P**	0.683144	0.234334	-1.049875	0.687322	0.235521	-1.028745	0.670087	0.232686	-1.002067
**O**	1.962534	0.058152	0.100766	1.961041	0.043770	0.124760	1.963902	0.056876	0.090120
**N**	-0.658256	-0.618597	-0.329837	-0.658940	-0.614408	-0.317868	-0.638455	-0.622093	-0.249521
**N**	-0.177545	1.601744	-0.371914	-0.174523	1.605133	-0.359679	-0.157762	1.597627	-0.316963
**C**	-1.161141	-1.981554	-0.574092	-1.166924	-1.972838	-0.585272	-1.161954	-1.971083	-0.531344
**C**	0.032466	-2.911225	-0.843845	0.026122	-2.922455	-0.777266	0.017218	-2.943521	-0.675075
**H**	0.614972	-2.567249	-1.713109	0.664896	-2.594483	-1.612578	0.681126	-2.645192	-1.490194
**H**	-0.321863	-3.929949	-1.059901	-0.331021	-3.937091	-1.007254	-0.348385	-3.948386	-0.896126
**H**	0.699699	-2.949192	0.026872	0.640312	-2.963926	0.131789	0.599536	-2.978841	0.244897
**C**	-1.914727	-2.469121	0.675072	-2.000926	-2.441460	0.619094	-2.045337	-2.423475	0.641163
**H**	-2.795338	-1.842858	0.879899	-2.880708	-1.799322	0.771160	-2.919684	-1.781100	0.753658
**H**	-1.256991	-2.451290	1.555928	-1.395490	-2.434012	1.537071	-1.480416	-2.414692	1.575219
**H**	-2.269244	-3.499044	0.520688	-2.361635	-3.466026	0.445836	-2.402346	-3.439579	0.462266
**C**	-2.110773	-2.001674	-1.791407	-2.041401	-1.984575	-1.856980	-1.990765	-1.973504	-1.829984
**H**	-3.004852	-1.394796	-1.593602	-2.930726	-1.354810	-1.718533	-2.864941	-1.331748	-1.725490
**H**	-2.441919	-3.028239	-2.013096	-2.380383	-3.006261	-2.088499	-2.338028	-2.982658	-2.065665
**H**	-1.599739	-1.603560	-2.680809	-1.469842	-1.606365	-2.717845	-1.388560	-1.614276	-2.667379
**C**	0.315102	2.967564	-0.116507	0.316921	2.972618	-0.103784	0.324778	2.971208	-0.096362
**C**	-0.905599	3.889543	0.051907	-0.903788	3.894649	0.062330	-0.894577	3.888874	0.084048
**H**	-1.553806	3.843391	-0.834748	-1.546297	3.856407	-0.828814	-1.553334	3.831541	-0.783560
**H**	-0.576623	4.928816	0.199753	-0.572782	4.931976	0.218178	-0.567940	4.922944	0.211247
**H**	-1.501816	3.597575	0.930144	-1.507417	3.599268	0.934357	-1.471851	3.610811	0.968768
**C**	1.122133	3.419435	-1.343968	1.127270	3.424495	-1.329021	1.105173	3.418157	-1.340909
**H**	0.492799	3.410832	-2.245535	0.499505	3.419110	-2.231802	0.467196	3.385757	-2.225946
**H**	1.982736	2.757698	-1.521482	1.987811	2.762120	-1.505351	1.970720	2.778496	-1.520702
**H**	1.507349	4.438250	-1.191245	1.513106	4.442499	-1.173520	1.468150	4.439228	-1.208913
**C**	1.187953	3.038972	1.151828	1.187904	3.043625	1.165861	1.223998	3.060093	1.149266
**H**	2.067529	2.392072	1.049591	2.067197	2.395444	1.065567	2.093367	2.415919	1.036216
**H**	0.616729	2.696027	2.027315	0.614098	2.704690	2.041455	0.676447	2.735149	2.036492
**H**	1.522015	4.071413	1.338739	1.523083	4.075905	1.350489	1.560667	4.087274	1.310512
**C**	3.152719	-0.613510	-0.347517	3.155495	-0.619532	-0.334090	3.149784	-0.613111	-0.357774
**H**	3.092004	-0.716435	-1.446657	3.088550	-0.714868	-1.433141	3.092271	-0.707897	-1.446117
**C**	3.360678	-2.000554	0.332943	3.371239	-2.010517	0.334238	3.351118	-2.003338	0.310096
**H**	2.593849	-2.155268	1.104961	2.608731	-2.177439	1.107801	2.591470	-2.162148	1.074477
**H**	3.274925	-2.824385	-0.390136	3.285705	-2.827682	-0.396150	3.262448	-2.813120	-0.414492
**C**	4.774197	-1.894817	0.944626	4.787087	-1.903102	0.940024	4.765645	-1.905350	0.919121
**H**	5.252585	-2.865471	1.142408	5.270230	-2.872967	1.129334	5.238996	-2.870956	1.103757
**C**	5.519791	-0.986430	-0.080924	5.524231	-0.985920	-0.083726	5.509880	-0.988297	-0.094833
**C**	4.448517	0.162673	0.021736	4.450003	0.159552	0.032000	4.440995	0.156411	0.020216
**C**	4.393620	0.417667	1.551973	4.404813	0.405513	1.563637	4.388236	0.397780	1.551191
**H**	3.417008	0.827234	1.839121	3.430542	0.815497	1.858876	3.421917	0.802926	1.843074
**H**	5.155540	1.154762	1.846024	5.169660	1.139992	1.856407	5.147396	1.122633	1.848807

**C**	4.658577	-0.981168	2.183214	4.674202	-0.997262	2.184954	4.652957	-1.007058	2.167494
**H**	5.581542	-0.991681	2.781021	5.599802	-1.008680	2.778613	5.570481	-1.022988	2.757154
**H**	3.841668	-1.304375	2.844618	3.860382	-1.326941	2.847229	3.843706	-1.333611	2.822507
**C**	5.664210	-1.612889	-1.476876	5.662813	-1.602675	-1.484743	5.658072	-1.601736	-1.496342
**H**	6.388781	-2.441445	-1.442474	6.389335	-2.429803	-1.458237	6.380019	-2.421790	-1.465341
**H**	6.049736	-0.872759	-2.195238	6.043371	-0.856268	-2.199254	6.038913	-0.857861	-2.200429
**H**	4.728515	-2.014816	-1.885076	4.726016	-2.003944	-1.891239	4.733714	-2.001321	-1.908913
**C**	6.924204	-0.560189	0.368457	6.929949	-0.558442	0.360766	6.917247	-0.570498	0.353624
**H**	7.575554	-1.443678	0.459975	7.583953	-1.441218	0.439242	7.556321	-1.453097	0.442247
**H**	6.936980	-0.036226	1.332084	6.947300	-0.043674	1.329341	6.938373	-0.050907	1.309294
**H**	7.382351	0.107158	-0.377890	7.380179	0.117598	-0.382584	7.372496	0.089861	-0.388429
**C**	4.683285	1.410122	-0.810005	4.676999	1.412085	-0.794412	4.672293	1.411807	-0.797284
**H**	3.896331	2.155220	-0.622905	3.890074	2.155074	-0.598458	3.888023	2.145247	-0.604218
**H**	4.681447	1.186181	-1.888372	4.668903	1.193325	-1.873768	4.673452	1.198333	-1.869200
**H**	5.645905	1.880955	-0.558069	5.640761	1.882290	-0.545799	5.627020	1.876656	-0.540787
**N**	-2.904711	0.955820	-0.152222	-2.905974	0.963590	-0.184988	-2.890452	0.952771	-0.177356
**H**	-4.176464	1.281472	0.506555	-4.188514	1.293318	0.450436	-4.183891	1.289224	0.431912
**C**	-3.931923	1.429328	1.572465	-3.960778	1.489039	1.512002	-3.981748	1.499790	1.485373
**H**	-4.776166	2.581878	-0.039724	-4.801440	2.559679	-0.157405	-4.780911	2.547051	-0.204012
**C**	-4.076194	3.415330	0.109651	-4.114521	3.409075	-0.039495	-4.097170	3.388331	-0.084608
**H**	-5.724438	2.815183	0.465967	-5.755424	2.801683	0.332978	-5.736417	2.796654	0.261144
**H**	-4.983308	2.501437	-1.118009	-5.000135	2.425636	-1.231872	-4.956334	2.397955	-1.272011
**H**	-5.136299	0.098795	0.421351	-5.134959	0.097253	0.404307	-5.128316	0.096198	0.377401
**C**	-5.699043	-0.292120	-0.803420	-5.629753	-0.388072	-0.816633	-5.555688	-0.423855	-0.847108
**C**	-5.489561	0.284018	-1.708694	-5.368408	0.116220	-1.750665	-5.239627	0.043781	-1.772457
**H**	-6.520278	-1.418069	-0.886417	-6.447369	-1.519512	-0.857558	-6.373107	-1.546780	-0.897705
**C**	-6.945994	-1.709954	-1.848843	-6.819361	-1.886204	-1.816684	-6.690222	-1.939818	-1.856241
**H**	-6.798054	-2.171412	0.259206	-6.789429	-2.183493	0.326471	-6.781990	-2.168735	0.280846
**C**	-7.439742	-3.052293	0.194734	-7.427291	-3.069006	0.294650	-7.416970	-3.045363	0.242285
**H**	-6.249575	-1.788104	1.485389	-6.308580	-1.705342	1.548427	-6.367491	-1.656379	1.505553
**C**	-6.460248	-2.369269	2.385643	-6.568675	-2.216538	2.477783	-6.677451	-2.133522	2.427522
**H**	-5.424521	-0.661793	1.561928	-5.486677	-0.573503	1.583170	-5.545331	-0.532056	1.549781
**C**	-4.986421	-0.373016	2.521328	-5.102105	-0.210679	2.540296	-5.215263	-0.146464	2.508029
**H**	-2.942572	0.945508	-1.174258	-2.924123	0.955232	-1.207523	-2.888440	0.940916	-1.190801

trans-N$_{endo}$-O$_{endo}$-**4**-*S*

Element	PBE/TZVP			B3LYP/TZVP			B3LYP/TZVP (CHCl3)		
	x	y	z	x	y	z	x	y	z
**P**	0.680335	0.388325	1.061442	0.733057	0.411898	1.17928	0.729391	0.405711	1.158762
**P**	-0.256858	0.210321	-1.455953	-0.187573	0.159002	-1.32443	-0.196358	0.162009	-1.339134
**O**	-1.90816	-0.190986	-1.297793	-1.833165	-0.176963	-1.248637	-1.844476	-0.178649	-1.262278
**N**	0.046073	1.43215	-0.237434	0.091607	1.406195	-0.136134	0.086335	1.404443	-0.153675
**N**	0.611194	-0.759919	-0.280357	0.560166	-0.801225	-0.078515	0.559475	-0.802933	-0.106583
**C**	-0.145951	2.891239	-0.335529	-0.092438	2.86378	-0.27421	-0.09875	2.864077	-0.285791
**C**	-1.545704	3.222293	-0.885124	-1.526108	3.193675	-0.722996	-1.529602	3.193792	-0.742339
**H**	-1.722605	2.728016	-1.851712	-1.786704	2.66854	-1.642611	-1.781637	2.677165	-1.669363
**H**	-1.647227	4.307551	-1.035702	-1.626901	4.265802	-0.905847	-1.629973	4.26727	-0.916054
**H**	-2.329881	2.899391	-0.188236	-2.247608	2.913204	0.042997	-2.256011	2.905018	0.015737
**C**	-0.005494	3.510191	1.064035	0.155834	3.536848	1.082776	0.138967	3.528942	1.07696
**H**	0.990181	3.323283	1.490194	1.168098	3.357935	1.443714	1.147731	3.346868	1.44601
**H**	-0.757261	3.094239	1.751155	-0.54453	3.165235	1.833401	-0.570065	3.155562	1.818634
**H**	-0.154394	4.598212	1.007728	0.014224	4.61515	0.990137	0.000522	4.607756	0.987592
**C**	0.904056	3.491947	-1.29388	0.883983	3.420018	-1.328209	0.88504	3.425626	-1.329329
**H**	1.922603	3.301646	-0.933369	1.91762	3.23342	-1.045209	1.916509	3.233319	-1.042549
**H**	0.770667	4.580297	-1.392919	0.751939	4.498107	-1.447506	0.755792	4.504872	-1.439202
**H**	0.806067	3.042998	-2.293549	0.707531	2.949237	-2.297473	0.711981	2.964109	-2.303808
**C**	0.986123	-2.187773	-0.3758	0.917576	-2.235158	-0.096692	0.923721	-2.235263	-0.134984
**C**	1.011926	-2.803429	1.032524	0.719051	-2.83462	1.304395	0.742728	-2.841628	1.265338
**H**	1.74272	-2.285873	1.672295	1.359459	-2.336679	2.0357	1.387681	-2.343195	1.99239
**H**	1.307537	-3.861194	0.972893	0.986338	-3.89334	1.296361	1.01576	-3.898672	1.24944
**H**	0.025834	-2.743305	1.514021	-0.313544	-2.740028	1.637843	-0.287717	-2.754715	1.608071
**C**	2.381102	-2.315056	-1.017524	2.385588	-2.412111	-0.512376	2.388405	-2.401	-0.567247
**H**	3.146528	-1.843245	-0.391641	3.052121	-1.956895	0.215307	3.058872	-1.942014	0.154483
**H**	2.392996	-1.825529	-2.002537	2.568846	-1.939863	-1.478576	2.557171	-1.92397	-1.534022
**H**	2.653139	-3.37326	-1.154769	2.64042	-3.471947	-0.593296	2.648184	-3.459199	-0.653752
**C**	-0.01797	-2.946896	-1.26387	0.035454	-2.979316	-1.113621	0.035921	-2.981511	-1.14527
**H**	-0.045009	-2.522339	-2.279019	0.195377	-2.596919	-2.124117	0.183364	-2.594912	-2.156173
**H**	-1.038151	-2.914033	-0.862451	-1.024701	-2.886611	-0.886796	-1.022172	-2.897361	-0.905478
**H**	0.290103	-3.99934	-1.349416	0.297667	-4.038936	-1.115572	0.3054	-4.039167	-1.153294
**C**	-2.708538	-0.050158	-0.107153	-2.694571	-0.040146	-0.108454	-2.697631	-0.044591	-0.112602
**H**	-2.437403	0.908688	0.357333	-2.430577	0.889871	0.388912	-2.426749	0.883353	0.384465
**C**	-2.602039	-1.194843	0.947445	-2.668432	-1.212863	0.91324	-2.661154	-1.221654	0.903652
**H**	-1.961296	-2.005999	0.585447	-2.037388	-2.020112	0.558688	-2.036591	-2.029411	0.538522
**H**	-2.157128	-0.829334	1.883364	-2.266454	-0.889478	1.87327	-2.247207	-0.902519	1.85987
**C**	-4.063409	-1.660932	1.119183	-4.146753	-1.645703	0.997196	-4.139369	-1.650853	1.005811
**H**	-4.258237	-2.188381	2.064794	-4.400551	-2.186852	1.91027	-4.382652	-2.193771	1.920555
**C**	-4.867746	-0.339893	0.919492	-4.90817	-0.304727	0.788907	-4.899502	-0.30723	0.812043
**C**	-4.216468	-0.048862	-0.482668	-4.175602	0.004048	-0.563853	-4.183909	0.004403	-0.549747
**C**	-4.482373	-1.37319	-1.248066	-4.430254	-1.291644	-1.377597	-4.454477	-1.288134	-1.363412
**H**	-3.730647	-1.515453	-2.03489	-3.648544	-1.433819	-2.12107	-3.684313	-1.432535	-2.118634
**H**	-5.466012	-1.339581	-1.739318	-5.378324	-1.223923	-1.913111	-5.410121	-1.215594	-1.884529

**C**	-4.421919	-2.470908	-0.145036	-4.454166	-2.416934	-0.302405	-4.466391	-2.417243	-0.291776
**H**	-5.38423	-2.991486	-0.0333	-5.4251	-2.911975	-0.255936	-5.438436	-2.908883	-0.234303
**H**	-3.66411	-3.240136	-0.355713	-3.709928	-3.192302	-0.491914	-3.72634	-3.19353	-0.493835
**C**	-4.587965	0.723798	1.993027	-4.66881	0.723857	1.905677	-4.642739	0.716875	1.929192
**H**	-5.032238	0.414876	2.952087	-5.168159	0.398836	2.821994	-5.130237	0.387911	2.850468
**H**	-5.04883	1.684817	1.715677	-5.092488	1.693078	1.630912	-5.068563	1.687616	1.66351
**H**	-3.520678	0.905578	2.172314	-3.619739	0.880422	2.149474	-3.590172	0.871198	2.158919
**C**	-6.388944	-0.534963	0.860979	-6.428577	-0.459384	0.648618	-6.422197	-0.457252	0.691255
**H**	-6.759236	-0.915092	1.826165	-6.850783	-0.859008	1.574535	-6.832012	-0.85787	1.62228
**H**	-6.711451	-1.236108	0.081337	-6.729657	-1.119457	-0.162157	-6.736181	-1.114587	-0.116946
**H**	-6.893384	0.425325	0.671983	-6.894844	0.512493	0.469409	-6.887238	0.516742	0.520603
**C**	-4.67147	1.193953	-1.225559	-4.561006	1.272773	-1.298316	-4.579068	1.277571	-1.271849
**H**	-4.094132	1.317305	-2.153878	-3.947671	1.392175	-2.193575	-3.977587	1.406735	-2.173948
**H**	-4.533109	2.103944	-0.62174	-4.419794	2.158875	-0.676207	-4.431487	2.158891	-0.644721
**H**	-5.73589	1.125273	-1.497325	-5.607541	1.241275	-1.609908	-5.629505	1.246023	-1.570042
**N**	2.34276	0.781363	1.296344	2.405511	0.778984	1.305044	2.395973	0.767292	1.30089
**H**	3.394634	1.172431	0.358483	3.386211	1.165573	0.290865	3.385078	1.162822	0.296888
**C**	2.911517	1.259942	-0.629001	2.840307	1.239614	-0.651666	2.851064	1.23579	-0.652345
**H**	3.990516	2.53609	0.753817	3.998266	2.536057	0.624476	3.985066	2.535691	0.641721
**C**	3.211125	3.308154	0.813196	3.228363	3.300289	0.724089	3.209466	3.295029	0.732702
**H**	4.751983	2.857204	0.028895	4.701026	2.844128	-0.151579	4.693898	2.84937	-0.1264
**H**	4.481697	2.460343	1.736857	4.551161	2.477881	1.565847	4.52445	2.478747	1.590715
**H**	4.523615	0.153527	0.235448	4.508786	0.156633	0.098304	4.514967	0.159274	0.117099
**C**	4.895459	-0.670317	1.305001	5.102656	-0.487053	1.183387	5.086664	-0.497781	1.20671
**C**	4.32687	-0.633964	2.236639	4.727841	-0.311321	2.184583	4.692022	-0.334908	2.202206
**H**	5.957791	-1.569764	1.177292	6.149383	-1.38247	0.993143	6.139775	-1.389079	1.026788
**C**	6.227657	-2.212375	2.018087	6.592114	-1.882336	1.846363	6.56516	-1.899199	1.882801
**H**	6.665322	-1.656385	-0.02465	6.622581	-1.643367	-0.289749	6.641506	-1.632038	-0.249497
**C**	7.489448	-2.364841	-0.127989	7.43312	-2.34589	-0.439752	7.456642	-2.331021	-0.3914
**H**	6.304581	-0.834657	-1.097227	6.043545	-0.998215	-1.377856	6.08484	-0.972274	-1.341606
**C**	6.844634	-0.901669	-2.044028	6.400062	-1.19876	-2.38106	6.464008	-1.157741	-2.33944
**H**	5.244459	0.064049	-0.963843	4.996576	-0.103785	-1.181098	5.031506	-0.082137	-1.155444
**C**	4.954989	0.691804	-1.811589	4.538003	0.38276	-2.035316	4.591937	0.416395	-2.012609
**H**	2.545617	0.96774	2.27699	2.678348	0.968064	2.258706	2.656813	0.964384	2.256862

trans-N_{endo}-O_{exo}-**4**-*S*

Element	PBE/TZVP			B3LYP/TZVP			B3LYP/TZVP (CHCl3)		
	x	y	z	x	y	z	x	y	z
**P**	1.457434	0.939241	1.488381	-1.455601	0.935621	-1.48193	-1.453734	0.931358	-1.479765
**P**	-0.225168	0.305837	-0.504878	0.200976	0.317384	0.512354	0.21362	0.316686	0.504781
**O**	-1.730145	0.154372	0.342065	1.698534	0.154318	-0.291584	1.708369	0.147655	-0.303644
**N**	0.541236	1.716112	0.189418	-0.532937	1.718459	-0.205265	-0.516714	1.71627	-0.213792
**N**	0.89516	-0.484954	0.578021	-0.890897	-0.476546	-0.579013	-0.883849	-0.479671	-0.577595
**C**	0.349318	3.140785	-0.13896	-0.360412	3.141996	0.133898	-0.349885	3.139901	0.134139
**C**	-1.146719	3.495782	-0.11707	1.131005	3.506707	0.128213	1.141123	3.506211	0.141169
**H**	-1.695729	2.933145	-0.885677	1.673993	2.947605	0.89114	1.681241	2.937311	0.899116
**H**	-1.292912	4.56832	-0.316821	1.267423	4.570932	0.334555	1.273129	4.567515	0.363768
**H**	-1.585035	3.252597	0.860185	1.574105	3.275021	-0.840133	1.590214	3.291153	-0.828368
**C**	1.079452	4.004988	0.901596	-1.084647	4.009551	-0.905501	-1.067947	4.010095	-0.907124
**H**	2.156548	3.789371	0.91867	-2.15221	3.791841	-0.93273	-2.135717	3.793828	-0.939989
**H**	0.676717	3.8302	1.910329	-0.674853	3.847434	-1.904421	-0.651828	3.850912	-1.904017
**H**	0.941942	5.068199	0.65692	-0.955702	5.063618	-0.652869	-0.939869	5.063144	-0.65053
**C**	0.916482	3.427263	-1.54408	-0.944042	3.420574	1.531215	-0.943552	3.412034	1.528246
**H**	1.999877	3.247009	-1.573196	-2.017025	3.230862	1.54869	-2.015182	3.215525	1.539718
**H**	0.732864	4.473149	-1.834578	-0.776315	4.461204	1.8196	-0.783439	4.453407	1.817689
**H**	0.439634	2.776148	-2.291982	-0.47219	2.781637	2.28051	-0.471099	2.775647	2.2794
**C**	0.88437	-1.884096	1.053851	-0.880025	-1.879839	-1.037835	-0.885239	-1.887875	-1.021301
**C**	2.238164	-2.187405	1.718516	-2.230928	-2.200936	-1.695257	-2.236863	-2.203982	-1.679254
**H**	3.068776	-2.039629	1.017793	-3.05354	-2.055149	-0.999254	-3.059206	-2.039321	-0.986966
**H**	2.251916	-3.230575	2.067093	-2.237098	-3.240487	-2.029252	-2.251599	-3.247925	-1.998868
**H**	2.402016	-1.541693	2.595314	-2.403224	-1.572654	-2.572308	-2.40099	-1.58519	-2.564559
**C**	0.711257	-2.799584	-0.168625	-0.700784	-2.786879	0.187836	-0.717357	-2.783754	0.21425
**H**	1.55698	-2.677351	-0.859295	-1.530269	-2.652437	0.882	-1.544784	-2.630218	0.907298
**H**	-0.216014	-2.569606	-0.714067	0.227898	-2.568603	0.716969	0.21482	-2.571783	0.74015
**H**	0.663756	-3.851884	0.14806	-0.670151	-3.833819	-0.120756	-0.700308	-3.834117	-0.083304
**C**	-0.240544	-2.131243	2.077793	0.244664	-2.128329	-2.058122	0.240279	-2.158657	-2.034953
**H**	-1.22334	-1.936738	1.633961	1.216256	-1.915385	-1.621625	1.212557	-1.952486	-1.596056
**H**	-0.12346	-1.458845	2.940898	0.119089	-1.476806	-2.925507	0.12436	-1.514672	-2.909565
**H**	-0.212445	-3.169972	2.441961	0.230183	-3.165486	-2.402817	0.215332	-3.199083	-2.368734
**C**	-2.924867	0.133652	-0.464011	2.896766	0.11932	0.495694	2.910807	0.126757	0.481344
**H**	-2.615948	0.148333	-1.52543	2.604984	0.098151	1.550123	2.621318	0.131585	1.536215
**C**	-3.888029	1.317665	-0.145988	3.845599	1.319157	0.207002	3.861315	1.317132	0.163161
**H**	-3.516038	1.882837	0.718756	3.463734	1.904403	-0.627129	3.479451	1.886553	-0.682169
**H**	-3.962898	2.018801	-0.989936	3.924425	1.985809	1.066675	3.943814	2.001109	1.00841
**C**	-5.231447	0.62253	0.161776	5.189535	0.643856	-0.13626	5.202195	0.629739	-0.168025
**H**	-6.110876	1.272567	0.042628	6.058532	1.290428	-0.003441	6.073486	1.276409	-0.052803
**C**	-5.19389	-0.619561	-0.779169	5.169368	-0.628431	0.75797	5.180988	-0.620615	0.756895
**C**	-3.812165	-1.119491	-0.212833	3.78762	-1.113966	0.190264	3.796617	-1.116367	0.204184
**C**	-4.087945	-1.17139	1.314287	4.045946	-1.111607	-1.339603	4.053659	-1.153904	-1.325152
**H**	-3.156129	-1.040036	1.878272	3.117367	-0.965348	-1.885906	3.124587	-1.024221	-1.875081
**H**	-4.504669	-2.149393	1.597344	4.462949	-2.068013	-1.658734	4.47185	-2.118044	-1.618198

**C**	-5.096482	-0.010837	1.562091	5.045731	0.061492	-1.55599	5.053183	0.013454	-1.57304
**H**	-6.063085	-0.381312	1.933261	6.002878	-0.287845	-1.945368	6.00855	-0.347039	-1.956548
**H**	-4.72558	0.717459	2.297856	4.666761	0.806332	-2.257364	4.672562	0.742026	-2.290659
**C**	-5.19149	-0.265938	-2.274878	5.186199	-0.329512	2.265514	5.201825	-0.285292	2.256804
**H**	-6.179846	0.120057	-2.569614	6.169774	0.048147	2.556463	6.187094	0.097598	2.534891
**H**	-4.996778	-1.161818	-2.884787	5.005387	-1.241872	2.839132	5.020837	-1.183701	2.851974
**H**	-4.449224	0.492537	-2.553283	4.451475	0.408041	2.582456	4.469223	0.461079	2.557635
**C**	-6.344421	-1.610291	-0.553032	6.325875	-1.601517	0.489619	6.334598	-1.602965	0.509876
**H**	-7.308188	-1.127633	-0.779761	7.280252	-1.116155	0.710687	7.289673	-1.115064	0.722131
**H**	-6.397728	-1.994611	0.473047	6.368874	-1.960534	-0.536489	6.377732	-1.983608	-0.508476
**H**	-6.246538	-2.475897	-1.226314	6.247026	-2.476532	1.139182	6.251462	-2.463617	1.17786
**C**	-3.257037	-2.407083	-0.793182	3.246507	-2.423596	0.728423	3.25155	-2.410197	0.775868
**H**	-2.32106	-2.6916	-0.292295	2.31698	-2.694656	0.228432	2.31754	-2.687355	0.287041
**H**	-3.043072	-2.308354	-1.869036	3.043412	-2.363721	1.800743	3.054558	-2.32339	1.847395
**H**	-3.964396	-3.239711	-0.658302	3.955535	-3.237663	0.561041	3.955675	-3.231487	0.624002
**N**	3.090181	1.176355	1.008722	-3.075794	1.180563	-0.997962	-3.070619	1.170572	-1.002047
**H**	3.669606	1.060314	-0.332733	-3.649353	1.058755	0.342882	-3.648914	1.060417	0.338461
**C**	2.819641	1.15101	-1.027292	-2.812779	1.161895	1.034457	-2.814479	1.153384	1.034022
**H**	4.644318	2.216574	-0.597087	-4.636223	2.202504	0.604244	-4.623462	2.216057	0.593531
**C**	4.139016	3.184632	-0.470401	-4.144528	3.167709	0.476152	-4.121345	3.175322	0.461706
**H**	5.05027	2.157425	-1.617313	-5.038671	2.141149	1.617085	-5.024458	2.163962	1.60744
**H**	5.494684	2.180148	0.101778	-5.478079	2.155338	-0.091148	-5.465124	2.174041	-0.102094
**H**	4.326985	-0.288153	-0.595407	-4.287536	-0.296716	0.599825	-4.30558	-0.286927	0.59327
**C**	5.390406	-0.745175	0.197123	-5.344994	-0.755363	-0.187935	-5.354909	-0.739271	-0.209908
**C**	5.755626	-0.131274	1.025096	-5.721685	-0.139557	-0.997006	-5.710586	-0.125536	-1.029728
**H**	5.97871	-1.988522	-0.042509	-5.910226	-2.005057	0.035071	-5.937136	-1.981947	0.012404
**C**	6.799515	-2.332821	0.590236	-6.723768	-2.349282	-0.592149	-6.743759	-2.321716	-0.626127
**H**	5.515694	-2.79428	-1.088484	-5.428917	-2.816528	1.059965	-5.481308	-2.792334	1.050832
**C**	5.972177	-3.7684	-1.274289	-5.865259	-3.792669	1.232409	-5.930548	-3.762913	1.221933
**H**	4.464047	-2.34472	-1.891036	-4.383226	-2.365129	1.857556	-4.444079	-2.346441	1.863497
**C**	4.092506	-2.96837	-2.707029	-3.998269	-2.990875	2.653833	-4.078913	-2.970764	2.670154
**H**	3.876432	-1.100516	-1.643337	-3.818868	-1.11375	1.626169	-3.863278	-1.101617	1.633597
**C**	3.038472	-0.760573	-2.25754	-2.98715	-0.777317	2.234379	-3.040761	-0.768915	2.256225
**H**	3.747964	1.272821	1.778923	-3.735833	1.257805	-1.757459	-3.725829	1.280665	-1.762034

trans-N_{exo}-O_{endo}-**4**-*S*

Element	PBE/TZVP			B3LYP/TZVP			B3LYP/TZVP (CHCl3)		
	X	y	z	x	y	z	x	y	z
**P**	0.906311	0.625246	-0.526357	0.906452	0.623193	-0.549406	-1.45373	0.931358	-1.47977
**P**	-0.597921	0.714255	1.714887	-0.570809	0.665915	1.68585	0.21362	0.316686	0.504781
**O**	-2.187866	0.245109	1.338885	-2.140593	0.196849	1.325324	1.708369	0.147655	-0.30364
**N**	0.42776	-0.405137	0.837285	0.436115	-0.432512	0.781676	-0.51671	1.71627	-0.21379
**N**	-0.084089	1.75606	0.397917	-0.089501	1.725157	0.385407	-0.88385	-0.47967	-0.5776
**C**	0.894647	-1.751766	1.209628	0.912653	-1.781035	1.130103	-0.34989	3.139901	0.134139
**C**	-0.117801	-2.386277	2.17567	-0.105286	-2.456341	2.059964	1.141123	3.506211	0.141169
**H**	-0.23011	-1.779938	3.087543	-0.232825	-1.890886	2.985643	1.681241	2.937311	0.899116
**H**	0.230225	-3.385077	2.477116	0.244295	-3.455584	2.326731	1.273129	4.567515	0.363768
**H**	-1.105696	-2.488248	1.708816	-1.078865	-2.548584	1.581778	1.590214	3.291153	-0.82837
**C**	1.00352	-2.611887	-0.061268	1.053613	-2.610936	-0.154969	-1.06795	4.010095	-0.90712
**H**	1.723346	-2.178061	-0.771307	1.785643	-2.168593	-0.832665	-2.13572	3.793828	-0.93999
**H**	0.027551	-2.694036	-0.562166	0.097552	-2.681169	-0.676999	-0.65183	3.850912	-1.90402
**H**	1.355156	-3.623065	0.191708	1.395203	-3.620086	0.084156	-0.93987	5.063144	-0.65053
**C**	2.271061	-1.673571	1.902825	2.273386	-1.700351	1.846432	-0.94355	3.412034	1.528246
**H**	3.028995	-1.253086	1.22938	3.027957	-1.254258	1.201296	-2.01518	3.215525	1.539718
**H**	2.611764	-2.673662	2.212641	2.622435	-2.695055	2.134717	-0.78344	4.453407	1.817689
**H**	2.207243	-1.037654	2.798593	2.185902	-1.092362	2.74968	-0.4711	2.775647	2.2794
**C**	-0.192291	3.220053	0.285277	-0.205259	3.189743	0.297767	-0.88524	-1.88788	-1.0213
**C**	0.995235	3.914429	0.989472	0.977322	3.875779	1.011752	-2.23686	-2.20398	-1.67925
**H**	1.035133	3.616032	2.047927	1.019886	3.561	2.056856	-3.05921	-2.03932	-0.98697
**H**	0.892485	5.009939	0.944655	0.869049	4.963248	0.987035	-2.2516	-3.24793	-1.99887
**H**	1.943665	3.635099	0.511272	1.919242	3.612439	0.53192	-2.40099	-1.58519	-2.56456
**C**	-1.499445	3.683442	0.952122	-1.513546	3.639224	0.968301	-0.71736	-2.78375	0.21425
**H**	-1.518799	3.407945	2.017571	-1.528876	3.36713	2.025787	-1.54478	-2.63022	0.907298
**H**	-2.378826	3.234356	0.471574	-2.382751	3.18657	0.492768	0.21482	-2.57178	0.74015
**H**	-1.585092	4.777996	0.885684	-1.609058	4.724857	0.902507	-0.70031	-3.83412	-0.0833
**C**	-0.204208	3.60534	-1.203823	-0.220903	3.602954	-1.181666	0.240279	-2.15866	-2.03495
**H**	-1.051588	3.135667	-1.7225	-1.052394	3.132086	-1.707254	1.212557	-1.95249	-1.59606
**H**	0.723853	3.284257	-1.701109	0.706331	3.313539	-1.680837	0.12436	-1.51467	-2.90957
**H**	-0.285163	4.696942	-1.313341	-0.321957	4.686784	-1.26832	0.215332	-3.19908	-2.36873
**C**	-2.572918	-0.385136	0.100105	-2.560829	-0.416921	0.097883	2.910807	0.126757	0.481344
**H**	-1.787052	-1.114214	-0.151936	-1.811036	-1.162654	-0.165401	2.621318	0.131585	1.536215
**C**	-2.823053	0.579963	-1.098169	-2.796042	0.557518	-1.092582	3.861315	1.317132	0.163161
**H**	-2.736074	1.624427	-0.776717	-2.663358	1.587621	-0.776287	3.479451	1.886553	-0.68217
**H**	-2.081347	0.426181	-1.894683	-2.083619	0.37642	-1.897385	3.943814	2.001109	1.00841
**C**	-4.261758	0.236517	-1.538934	-4.252728	0.264712	-1.505879	5.202195	0.629739	-0.16803
**H**	-4.496519	0.525784	-2.574079	-4.49445	0.563575	-2.52729	6.073486	1.276409	-0.0528
**C**	-4.35309	-1.291481	-1.241427	-4.391011	-1.257206	-1.211947	5.180988	-0.62062	0.756895
**C**	-3.933682	-1.114907	0.265307	-3.938643	-1.100014	0.282808	3.796617	-1.11637	0.204184
**C**	-4.950287	-0.053962	0.765437	-4.911154	-0.012016	0.806275	4.053659	-1.1539	-1.32515
**H**	-4.533563	0.501946	1.61492	-4.469768	0.52156	1.645515	3.124587	-1.02422	-1.87508
**H**	-5.871939	-0.542038	1.115335	-5.836434	-0.467912	1.161629	4.47185	-2.11804	-1.6182

**C**	-5.206608	0.845233	-0.480318	-5.155744	0.902336	-0.429738	5.053183	0.013454	-1.57304
**H**	-6.256941	0.805044	-0.803872	-6.203752	0.901468	-0.732597	6.00855	-0.34704	-1.95655
**H**	-4.968486	1.902722	-0.292784	-4.878783	1.941015	-0.241633	4.672562	0.742026	-2.29066
**C**	-3.390477	-2.144841	-2.082695	-3.477759	-2.141468	-2.076115	5.201825	-0.28529	2.256804
**H**	-3.731348	-2.172568	-3.12944	-3.831556	-2.140008	-3.110287	6.187094	0.097598	2.534891
**H**	-3.374236	-3.18401	-1.718361	-3.503184	-3.176178	-1.725353	5.020837	-1.1837	2.851974
**H**	-2.356498	-1.777389	-2.086842	-2.436515	-1.825396	-2.091707	4.469223	0.461079	2.557635
**C**	-5.75786	-1.882671	-1.421381	-5.816346	-1.802639	-1.373715	6.334598	-1.60297	0.509876
**H**	-6.067509	-1.807967	-2.475743	-6.134408	-1.713823	-2.415929	7.289673	-1.11506	0.722131
**H**	-6.523744	-1.385453	-0.813453	-6.554542	-1.290917	-0.759824	6.377732	-1.98361	-0.50848
**H**	-5.763966	-2.950681	-1.153592	-5.850343	-2.862802	-1.110959	6.251462	-2.46362	1.17786
**C**	-3.868532	-2.36338	1.12539	-3.893774	-2.350424	1.138246	3.25155	-2.4102	0.775868
**H**	-3.526584	-2.112788	2.140727	-3.522901	-2.112871	2.137345	2.31754	-2.68736	0.287041
**H**	-3.171217	-3.107201	0.708165	-3.234776	-3.108024	0.70644	3.054558	-2.32339	1.847395
**H**	-4.857925	-2.837976	1.212198	-4.888089	-2.790292	1.244273	3.955675	-3.23149	0.624002
**N**	2.522122	1.148904	-0.228407	2.506899	1.145519	-0.229871	-3.07062	1.170572	-1.00205
**H**	3.589985	0.981964	-1.219974	3.594066	1.005357	-1.200511	-3.64891	1.060417	0.338461
**C**	3.073366	0.73777	-2.164209	3.110391	0.780582	-2.154929	-2.81448	1.153384	1.034022
**H**	4.362729	2.291735	-1.420917	4.356944	2.324577	-1.357007	-4.62346	2.216057	0.593531
**C**	3.676452	3.086586	-1.745184	3.675402	3.110127	-1.686219	-4.12135	3.175322	0.461706
**H**	5.1514	2.169212	-2.177607	5.162911	2.223811	-2.086602	-5.02446	2.163962	1.60744
**H**	4.841853	2.620927	-0.486239	4.799098	2.64102	-0.409679	-5.46512	2.174041	-0.10209
**H**	4.495856	-0.197018	-0.881733	4.500103	-0.169761	-0.86076	-4.30558	-0.28693	0.59327
**C**	5.393826	-0.140094	0.19558	5.369851	-0.120302	0.231171	-5.35491	-0.73927	-0.20991
**C**	5.490088	0.780383	0.777403	5.445768	0.78528	0.821902	-5.71059	-0.12554	-1.02973
**H**	6.1629	-1.251784	0.545752	6.134006	-1.226295	0.584464	-5.93714	-1.98195	0.012404
**C**	6.851812	-1.190814	1.390864	6.798145	-1.171453	1.438604	-6.74376	-2.32172	-0.62613
**H**	6.05121	-2.441195	-0.181739	6.044724	-2.403331	-0.154187	-5.48131	-2.79233	1.050832
**C**	6.650998	-3.311045	0.092787	6.638169	-3.266221	0.121814	-5.93055	-3.76291	1.221933
**H**	5.16937	-2.506907	-1.263326	5.189725	-2.461615	-1.249124	-4.44408	-2.34644	1.863497
**C**	5.076971	-3.429787	-1.839802	5.113461	-3.371971	-1.831429	-4.07891	-2.97076	2.670154
**H**	4.400221	-1.391705	-1.608031	4.425329	-1.351499	-1.596877	-3.86328	-1.10162	1.633597
**C**	3.705008	-1.450123	-2.450146	3.75286	-1.407829	-2.445707	-3.04076	-0.76892	2.256225
**H**	2.828089	1.334921	0.728679	2.79385	1.315965	0.726626	-3.72583	1.280665	-1.76203

4-*R*

cis-N$_{endo}$-O$_{exo}$-**4**-*R*

Element	PBE/TZVP			PBE/TZVP (CHCl3)			B3LYP/TZVP (CHCl3)		
	X	y	z	x	y	z	x	y	z
O	0.916228	0.259785	0.121538	0.921690	0.252185	0.127717	0.878587	0.293955	0.153265
P	-2.588165	1.404922	0.382021	-2.573320	1.423926	0.377321	-2.599884	1.373914	0.369730
P	0.001485	1.398874	1.018867	0.013755	1.397091	1.019614	-0.042353	1.378939	1.068416
N	-1.418527	0.444727	1.328302	-1.414493	0.455707	1.328302	-1.457916	0.427763	1.338765
N	-1.068839	2.123288	-0.178103	-1.047818	2.132067	-0.178217	-1.090324	2.144090	-0.097704
N	-3.056223	0.439418	-0.951705	-3.051625	0.461858	-0.952404	-3.001344	0.438811	-0.991549
H	-4.058324	0.284612	-1.043977	-4.055697	0.321384	-1.050327	-3.988614	0.272206	-1.126059
C	-1.725470	-0.300786	2.567907	-1.728509	-0.289600	2.566372	-1.773109	-0.387779	2.528626
C	-1.965407	0.660290	3.748804	-1.975694	0.670380	3.746705	-2.094413	0.504728	3.740908
H	-1.070680	1.273963	3.933350	-1.082209	1.284352	3.936494	-1.242038	1.143320	3.982993
H	-2.198917	0.105238	4.670579	-2.213451	0.113419	4.666153	-2.329425	-0.100444	4.620051
H	-2.806798	1.334752	3.529636	-2.816726	1.344255	3.523960	-2.952304	1.145763	3.527024
C	-0.545214	-1.232130	2.881388	-0.549825	-1.220402	2.887693	-0.567259	-1.281855	2.846148
H	0.371472	-0.655644	3.076869	0.363996	-0.643002	3.093952	0.306863	-0.685436	3.114228
H	-0.355037	-1.909298	2.038031	-0.349933	-1.893477	2.043115	-0.313153	-1.897732	1.984464
H	-0.762825	-1.831042	3.778079	-0.775581	-1.822446	3.780180	-0.796823	-1.933139	3.691924
C	-2.985610	-1.145771	2.319289	-2.986918	-1.135211	2.311173	-2.982024	-1.277898	2.205683
H	-3.851148	-0.505641	2.087447	-3.851333	-0.496330	2.071780	-3.864553	-0.677726	1.971959
H	-3.230785	-1.725245	3.221408	-3.237005	-1.713556	3.212612	-3.224014	-1.900268	3.069522
H	-2.833310	-1.840135	1.482546	-2.827145	-1.829145	1.475287	-2.770120	-1.923822	1.355168
C	-0.918791	3.481574	-0.741032	-0.884523	3.491692	-0.737425	-0.918896	3.483300	-0.691727
C	0.517311	3.619291	-1.272255	0.549972	3.613921	-1.276361	0.532353	3.614635	-1.177337
H	1.250073	3.530070	-0.454802	1.287141	3.512064	-0.464341	1.234805	3.569377	-0.341990
H	0.658303	4.604367	-1.740836	0.699431	4.599473	-1.741033	0.676341	4.574264	-1.677409
H	0.730827	2.839111	-2.016689	0.749637	2.834937	-2.025963	0.776999	2.814467	-1.877340
C	-1.189306	4.565613	0.321243	-1.135657	4.575059	0.330187	-1.226565	4.591669	0.331273
H	-0.485571	4.463309	1.160839	-0.428077	4.462181	1.165281	-0.561418	4.510465	1.193475
H	-2.211964	4.468280	0.715327	-2.157420	4.489615	0.729693	-2.256412	4.509333	0.684864
H	-1.077652	5.575565	-0.102853	-1.013718	5.584140	-0.092721	-1.094813	5.581720	-0.112216
C	-1.907681	3.638987	-1.907196	-1.878278	3.667073	-1.896732	-1.860762	3.616056	-1.896865
H	-1.717218	2.882457	-2.681233	-1.703123	2.912209	-2.676028	-1.648833	2.842835	-2.635836
H	-1.805889	4.638142	-2.356204	-1.764380	4.666912	-2.340914	-1.731082	4.594025	-2.364887
H	-2.946779	3.522938	-1.565353	-2.917065	3.565392	-1.548793	-2.906108	3.522231	-1.597197
C	2.308427	0.545316	-0.114100	2.317681	0.529059	-0.108129	2.264204	0.572146	-0.093897
H	2.517160	1.578437	0.215459	2.533866	1.558416	0.227459	2.477259	1.597405	0.218148
C	2.661753	0.344563	-1.616197	2.668330	0.336329	-1.611695	2.603651	0.359899	-1.594796
H	1.777074	-0.008184	-2.164743	1.781767	-0.007425	-2.163013	1.723642	0.003255	-2.129927
H	2.987885	1.283809	-2.085854	3.000457	1.277035	-2.073794	2.922614	1.288506	-2.069047
C	3.784735	-0.711317	-1.573295	3.784434	-0.727179	-1.576522	3.729610	-0.691452	-1.550509
H	4.396250	-0.755232	-2.486434	4.396123	-0.767970	-2.489605	4.331434	-0.738217	-2.459251
C	4.557915	-0.319928	-0.276355	4.560134	-0.350523	-0.276788	4.507461	-0.291726	-0.262707

**C**	3.273729	-0.426274	0.629100	3.275085	-0.454837	0.627935	3.230625	-0.393656	0.646652
**C**	2.781575	-1.860442	0.319170	2.773901	-1.883659	0.307590	2.740884	-1.829971	0.351374
**H**	1.708689	-1.952701	0.530134	1.700369	-1.971857	0.517851	1.679749	-1.927138	0.567518
**H**	3.300048	-2.588487	0.960296	3.289197	-2.619304	0.942491	3.263786	-2.546472	0.986236
**C**	3.129181	-2.058072	-1.186578	3.119918	-2.072423	-1.199773	3.079933	-2.036629	-1.154126
**H**	3.818884	-2.900953	-1.337751	3.804015	-2.918890	-1.356273	3.766221	-2.871468	-1.300648
**H**	2.238957	-2.259123	-1.800933	2.227794	-2.261852	-1.815319	2.193785	-2.242172	-1.756747
**C**	5.670114	-1.306405	0.105385	5.665661	-1.347411	0.097650	5.627359	-1.270082	0.117807
**H**	6.473957	-1.276418	-0.646891	6.469422	-1.315302	-0.654653	6.416187	-1.242025	-0.638536
**H**	5.328110	-2.345678	0.183248	5.317029	-2.385186	0.166712	5.297440	-2.302714	0.209435
**H**	6.117593	-1.029851	1.072621	6.113817	-1.080993	1.067454	6.077679	-0.982572	1.071001
**C**	5.193591	1.078721	-0.322976	5.205080	1.044291	-0.313168	5.145530	1.105494	-0.323025
**H**	4.494280	1.879759	-0.593511	4.511100	1.851933	-0.577828	4.453500	1.902426	-0.588139
**H**	6.012560	1.095606	-1.058943	6.023873	1.059750	-1.049383	5.949969	1.113600	-1.062963
**H**	5.629055	1.336382	0.654819	5.641967	1.291144	0.666821	5.587130	1.363430	0.642501
**C**	3.436165	-0.118415	2.106361	3.440967	-0.159171	2.107473	3.398428	-0.079073	2.120283
**H**	2.471937	-0.196391	2.630355	2.477139	-0.236579	2.632743	2.445162	-0.164184	2.645358
**H**	3.815878	0.902417	2.267323	3.827440	0.858148	2.274703	3.769054	0.937570	2.271796
**H**	4.130979	-0.824310	2.586628	4.133116	-0.872543	2.580464	4.098785	-0.771845	2.592490
**C**	-2.188071	-0.419621	-1.766692	-2.189706	-0.406264	-1.764879	-2.093452	-0.390303	-1.790564
**H**	-1.173240	-0.012080	-1.637112	-1.169877	-0.014624	-1.627707	-1.097863	0.025226	-1.632984
**C**	-2.573307	-0.312888	-3.247742	-2.563153	-0.291506	-3.248338	-2.439690	-0.272730	-3.278234
**H**	-2.514155	0.732329	-3.582679	-2.480516	0.752611	-3.581807	-2.384001	0.768702	-3.597870
**H**	-3.601319	-0.670232	-3.417716	-3.596569	-0.628714	-3.424910	-3.451152	-0.636743	-3.475928
**H**	-1.902309	-0.925969	-3.866753	-1.897613	-0.914845	-3.863159	-1.747734	-0.864832	-3.880341
**C**	-2.166892	-1.866288	-1.290089	-2.192037	-1.853859	-1.289986	-2.065692	-1.838636	-1.327722
**C**	-0.965905	-2.437761	-0.851535	-1.001819	-2.442177	-0.842918	-0.881744	-2.389385	-0.841639
**H**	-0.068694	-1.816182	-0.828177	-0.095798	-1.833433	-0.813521	-0.004830	-1.760514	-0.776489
**C**	-0.924496	-3.760075	-0.400511	-0.981778	-3.765256	-0.390897	-0.838637	-3.706636	-0.393450
**H**	0.021379	-4.184937	-0.055787	-0.044926	-4.203253	-0.037928	0.089903	-4.113297	-0.009820
**C**	-2.090296	-4.530727	-0.379486	-2.158766	-4.520195	-0.378741	-1.986702	-4.492522	-0.424065
**H**	-2.063214	-5.561968	-0.021755	-2.148615	-5.551671	-0.020566	-1.959130	-5.515793	-0.069617
**C**	-3.295637	-3.969882	-0.816360	-3.353529	-3.942175	-0.824693	-3.175991	-3.951306	-0.909933
**H**	-4.211667	-4.564535	-0.801943	-4.277918	-4.523987	-0.816924	-4.075469	-4.554992	-0.935092
**C**	-3.330280	-2.649858	-1.272081	-3.367107	-2.621081	-1.280495	-3.211767	-2.636101	-1.361546
**H**	-4.281165	-2.224859	-1.606146	-4.309667	-2.182387	-1.619664	-4.146471	-2.226780	-1.728379

trans-N$_{endo}$-O$_{exo}$-**4**-*R*

Element	PBE/TZVP			PBE/TZVP (CHCl3)			B3LYP/TZVP (CHCl3)		
	x	y	z	x	y	z	x	y	z
**O**	-1.682879	0.193483	-0.430717	1.695130	0.189455	0.434836	-1.648343	0.166832	-0.423028
**P**	1.481492	0.989058	-1.434515	-1.475430	0.974882	1.426737	1.495957	0.989263	-1.435807
**P**	-0.192016	0.120384	0.461419	0.209019	0.125006	-0.465491	-0.171347	0.128852	0.442859
**N**	1.012645	-0.529730	-0.627362	-1.000862	-0.537365	0.608762	1.007878	-0.521417	-0.651782
**N**	0.537123	1.621298	-0.066908	-0.526594	1.618924	0.066860	0.529197	1.620221	-0.099456
**N**	3.088454	1.318138	-0.939212	-3.082831	1.302784	0.947980	3.084152	1.321112	-0.928188
**H**	3.747820	1.472580	-1.698451	-3.733092	1.466229	1.713745	3.752893	1.452044	-1.673136
**C**	1.079855	-1.886779	-1.204259	-1.070562	-1.902280	1.168024	1.085771	-1.883688	-1.211380
**C**	0.084786	-2.048168	-2.371279	-0.082304	-2.080688	2.338318	0.087248	-2.063228	-2.369661
**H**	-0.946214	-1.882534	-2.035371	0.950665	-1.909191	2.011039	-0.932393	-1.887405	-2.032738
**H**	0.154917	-3.057478	-2.805857	-0.154404	-3.097037	2.755491	0.150112	-3.074295	-2.779869
**H**	0.301657	-1.316101	-3.163400	-0.305825	-1.362202	3.141206	0.301982	-1.354625	-3.172291
**C**	0.759047	-2.901188	-0.095133	-0.743122	-2.902367	0.047782	0.775687	-2.893359	-0.096990
**H**	-0.262283	-2.764622	0.294229	0.281631	-2.763217	-0.331584	-0.245273	-2.781762	0.275607
**H**	1.465109	-2.794944	0.740215	-1.442967	-2.784243	-0.791593	1.464949	-2.764475	0.738071
**H**	0.831788	-3.925109	-0.489861	-0.820536	-3.930998	0.428958	0.876908	-3.910834	-0.479380
**C**	2.506657	-2.139216	-1.719292	-2.499913	-2.161890	1.671696	2.510356	-2.135184	-1.726355
**H**	2.780644	-1.408447	-2.495729	-2.778301	-1.443319	2.457882	2.779334	-1.420593	-2.507613
**H**	2.570073	-3.141818	-2.167659	-2.565687	-3.172020	2.102374	2.576830	-3.136693	-2.156606
**H**	3.239272	-2.068499	-0.906542	-3.226540	-2.075373	0.854903	3.236202	-2.050436	-0.921114
**C**	-0.015070	2.987686	0.048429	0.015777	2.989988	-0.048371	-0.001547	2.990111	0.042228
**C**	-0.815249	3.082556	1.356175	0.817569	3.090722	-1.354628	-0.790859	3.083402	1.355003
**H**	-1.699094	2.430194	1.330974	1.704195	2.441812	-1.328890	-1.675371	2.447074	1.333504
**H**	-1.165465	4.114207	1.504949	1.163654	4.124235	-1.499483	-1.125732	4.110338	1.510987
**H**	-0.192635	2.799705	2.217884	0.197669	2.807964	-2.218306	-0.169933	2.791593	2.204364
**C**	-0.924494	3.322366	-1.151284	0.922655	3.331475	1.151206	-0.919453	3.346455	-1.141643
**H**	-1.731391	2.582845	-1.234820	1.741076	2.603712	1.227981	-1.723302	2.616577	-1.226003
**H**	-0.347082	3.301316	-2.087873	0.347458	3.299045	2.088954	-0.357189	3.341390	-2.077897
**H**	-1.362768	4.326344	-1.041553	1.346411	4.341764	1.044025	-1.350931	4.341778	-1.010413
**C**	1.151727	3.989591	0.105208	-1.157124	3.984148	-0.106311	1.173637	3.977952	0.102047
**H**	1.783449	3.796757	0.984255	-1.789636	3.783493	-0.982846	1.811303	3.760482	0.959960
**H**	0.764995	5.017517	0.171117	-0.775645	5.013556	-0.177826	0.799684	4.999721	0.196231
**H**	1.781269	3.917387	-0.793187	-1.783912	3.912478	0.794074	1.785813	3.921848	-0.798881
**C**	-2.713836	-0.741781	-0.056835	2.729085	-0.743027	0.053619	-2.688930	-0.753726	-0.058114
**H**	-2.245820	-1.589348	0.475628	2.262015	-1.583184	-0.490519	-2.237343	-1.603900	0.459949
**C**	-3.488759	-1.228120	-1.316346	3.498149	-1.246978	1.309736	-3.467697	-1.221840	-1.319217
**H**	-3.128322	-0.693088	-2.205934	3.138108	-0.723372	2.206480	-3.106113	-0.691927	-2.200116
**H**	-3.341769	-2.303225	-1.495238	3.347625	-2.323957	1.472849	-3.334569	-2.288978	-1.499894
**C**	-4.955830	-0.885056	-0.985857	4.967130	-0.902829	0.988814	-4.929342	-0.864754	-0.985277
**H**	-5.692347	-1.444591	-1.581074	5.700311	-1.472143	1.578664	-5.666025	-1.410248	-1.576898
**C**	-5.019623	-1.134146	0.551708	5.035553	-1.131925	-0.551526	-4.994471	-1.118338	0.548052
**C**	-3.831206	-0.144489	0.852710	3.850335	-0.135545	-0.843604	-3.796639	-0.146713	0.850095
**C**	-4.314635	1.154609	0.162194	4.334434	1.153394	-0.134881	-4.264446	1.160655	0.167468

**H**	-3.463477	1.793216	-0.104411	3.483421	1.791004	0.135606	-3.415105	1.785737	-0.095929
**H**	-4.954722	1.735382	0.842671	4.979384	1.740074	-0.805539	-4.893956	1.739738	0.844548
**C**	-5.095522	0.650433	-1.087746	5.109718	0.631055	1.111418	-5.052944	0.671003	-1.082518
**H**	-6.149562	0.962842	-1.063296	6.164483	0.941357	1.093371	-6.094969	0.991847	-1.052956
**H**	-4.668656	1.028297	-2.028176	4.680510	0.997300	2.055571	-4.627124	1.045622	-2.014724
**C**	-6.349230	-0.717354	1.195748	6.368354	-0.709593	-1.185457	-6.320346	-0.694071	1.195190
**H**	-7.165214	-1.355947	0.822289	7.180677	-1.355277	-0.816249	-7.135007	-1.313583	0.810887
**H**	-6.625866	0.325134	0.996117	6.646417	0.329811	-0.971864	-6.584806	0.345702	1.014792
**H**	-6.306940	-0.846737	2.288298	6.329064	-0.825563	-2.279645	-6.280703	-0.840131	2.277297
**C**	-4.760812	-2.593425	0.959093	4.774767	-2.585296	-0.978840	-4.757790	-2.583034	0.950218
**H**	-3.839578	-3.018524	0.541424	3.851574	-3.014214	-0.569376	-3.849768	-3.019237	0.538527
**H**	-5.595679	-3.229558	0.625900	5.608105	-3.226335	-0.651261	-5.595669	-3.198472	0.612732
**H**	-4.700291	-2.683651	2.054681	4.717168	-2.660087	-2.075763	-4.702182	-2.675441	2.037471
**C**	-3.425451	0.018883	2.306836	3.452254	0.047385	-2.297605	-3.393265	0.009375	2.304177
**H**	-2.553385	0.682116	2.406835	2.587116	0.720412	-2.393945	-2.542209	0.684167	2.410025
**H**	-3.147445	-0.946707	2.757053	3.169622	-0.911334	-2.759545	-3.103907	-0.949440	2.741019
**H**	-4.244173	0.453272	2.901024	4.277851	0.481405	-2.882405	-4.215975	0.419139	2.894819
**C**	3.658723	1.224406	0.410186	-3.666872	1.223316	-0.397166	3.638248	1.228601	0.426171
**H**	2.802679	1.339119	1.094080	-2.818375	1.329796	-1.091402	2.792404	1.363503	1.101485
**C**	4.636060	2.382166	0.650685	-4.634502	2.392702	-0.621370	4.630633	2.371105	0.665071
**H**	4.131900	3.346955	0.502812	-4.118812	3.351404	-0.474162	4.143174	3.333628	0.508514
**H**	5.489353	2.329907	-0.043919	-5.480565	2.344532	0.081929	5.480017	2.303305	-0.019170
**H**	5.039078	2.341538	1.672803	-5.045588	2.362222	-1.640650	5.019706	2.332473	1.684162
**C**	4.303902	-0.124917	0.699902	-4.333533	-0.116627	-0.682883	4.254489	-0.130386	0.720128
**C**	3.782867	-0.956961	1.698836	-3.850083	-0.943533	-1.705196	3.694864	-0.964160	1.686560
**H**	2.902766	-0.629838	2.258363	-2.984417	-0.619321	-2.288527	2.814939	-0.635014	2.226774
**C**	4.353371	-2.205604	1.963143	-4.441028	-2.184115	-1.965969	4.231666	-2.223267	1.942665
**H**	3.927573	-2.844032	2.740338	-4.043401	-2.819227	-2.760604	3.776322	-2.861210	2.690720
**C**	5.459166	-2.640230	1.227389	-5.531384	-2.614300	-1.203887	5.342383	-2.665754	1.230983
**H**	5.903304	-3.617414	1.426675	-5.991411	-3.584800	-1.400187	5.758705	-3.647082	1.422910
**C**	5.992167	-1.815156	0.231026	-6.028100	-1.792833	-0.184872	5.914017	-1.837867	0.266558
**H**	6.855326	-2.147543	-0.349636	-6.879014	-2.121438	0.415773	6.777835	-2.174851	-0.293926
**C**	5.419820	-0.567671	-0.025679	-5.434830	-0.553929	0.069025	5.374452	-0.580813	0.017571
**H**	5.839976	0.061090	-0.815436	-5.825495	0.071314	0.876291	5.821108	0.046263	-0.745556

5-*S*

cis-N_{endo}-O_{exo}-**5**-*S*

Element	PBE/TZVP			PBE/TZVP (CHCl3)			B3LYP/TZVP (CHCl3)		
	X	y	z	x	y	z	x	y	z
**P**	-2.303718	-1.224410	0.265326	-2.305231	-1.225425	0.283171	-2.350242	-1.150668	0.266583
**O**	1.054588	-0.379205	0.281683	1.069005	-0.368735	0.267597	1.018965	-0.417607	0.313845
**N**	-1.418907	-0.345925	1.394213	-1.398749	-0.340733	1.396913	-1.449138	-0.293956	1.387622
**C**	-1.835768	0.524990	2.534220	-1.802077	0.516991	2.550241	-1.846097	0.621825	2.497134
**P**	0.089997	-1.315270	1.268878	0.100956	-1.312380	1.254862	0.013566	-1.303928	1.285868
**N**	-0.921567	-2.143962	0.051278	-0.911671	-2.124880	0.036448	-0.998945	-2.105773	0.074473
**C**	-0.641393	1.423549	2.880534	-0.612182	1.428820	2.877329	-0.630323	1.488872	2.839543
**H**	-0.903101	2.083315	3.719070	-0.866578	2.078763	3.725810	-0.885560	2.175633	3.647220
**H**	0.232265	0.828992	3.190612	0.275257	0.843480	3.164159	0.212780	0.882748	3.178747
**H**	-0.361509	2.044054	2.019842	-0.358491	2.058654	2.015023	-0.320128	2.070410	1.973617
**N**	-2.861735	-0.480360	-1.112010	-2.886102	-0.476304	-1.077473	-2.851809	-0.397009	-1.107712
**H**	-3.870610	-0.366633	-1.208647	-3.898307	-0.390364	-1.170784	-3.846735	-0.277170	-1.244239
**C**	-3.015387	1.397099	2.084189	-3.001640	1.377387	2.130567	-2.987734	1.523947	2.014751
**H**	-3.302289	2.073005	2.901398	-3.266396	2.056321	2.952797	-3.246915	2.229232	2.804999
**H**	-2.751776	2.003328	1.208361	-2.765614	1.979797	1.243878	-2.694876	2.088219	1.131482
**H**	-3.904741	0.794379	1.842003	-3.891908	0.766645	1.917550	-3.890903	0.954613	1.784873
**C**	-2.222873	-0.331867	3.750410	-2.152372	-0.351092	3.769812	-2.279421	-0.191070	3.724961
**H**	-2.475343	0.311148	4.605612	-2.396004	0.286261	4.631957	-2.533211	0.477027	4.549716
**H**	-3.099631	-0.961025	3.538712	-3.021722	-0.993898	3.571093	-3.156351	-0.802228	3.506835
**H**	-1.390915	-0.985089	4.051821	-1.302829	-0.991703	4.048461	-1.474797	-0.849794	4.056745
**C**	-0.555353	-3.255031	-0.875761	-0.560985	-3.242911	-0.886072	-0.668186	-3.234981	-0.840785
**C**	0.070804	-2.684703	-2.158519	0.101079	-2.686296	-2.157044	0.021573	-2.698794	-2.102299
**H**	0.433893	-3.500370	-2.799730	0.449135	-3.512429	-2.793137	0.364283	-3.528142	-2.722913
**H**	-0.668260	-2.110274	-2.734263	-0.611236	-2.087834	-2.741451	-0.667328	-2.099084	-2.696620
**H**	0.915528	-2.024945	-1.922652	0.960608	-2.051834	-1.906552	0.878483	-2.079896	-1.843372
**C**	-1.816603	-4.059354	-1.218805	-1.838481	-4.009271	-1.253852	-1.959695	-3.966501	-1.222830
**H**	-1.552465	-4.881746	-1.897584	-1.583398	-4.833134	-1.934289	-1.720082	-4.798975	-1.884901
**H**	-2.269411	-4.498738	-0.318362	-2.317361	-4.439993	-0.362956	-2.462757	-4.368064	-0.342098
**H**	-2.561680	-3.436607	-1.737514	-2.556854	-3.360752	-1.778113	-2.645796	-3.309009	-1.761229
**C**	0.446571	-4.156520	-0.140751	0.404531	-4.174992	-0.140450	0.261882	-4.193835	-0.088418
**H**	0.740810	-4.990685	-0.792262	0.688575	-5.010928	-0.794264	0.514171	-5.037317	-0.731738
**H**	1.363130	-3.610581	0.128236	1.328824	-3.653351	0.150399	1.196825	-3.708618	0.197749
**H**	0.006776	-4.570198	0.777645	-0.064446	-4.581528	0.766570	-0.219207	-4.574798	0.813466
**C**	2.407288	-0.052033	0.755988	2.414099	-0.040427	0.743791	2.366444	-0.115858	0.777749
**H**	2.599323	-0.674416	1.645878	2.604648	-0.652788	1.640756	2.550303	-0.727422	1.663232
**C**	2.564812	1.461069	1.065712	2.573477	1.475815	1.040085	2.559750	1.393633	1.079262
**H**	1.650209	2.008146	0.799932	1.660344	2.020302	0.766135	1.664735	1.955542	0.821364
**H**	2.748667	1.633454	2.135275	2.752834	1.657175	2.108767	2.753467	1.563302	2.138073
**C**	3.762062	1.875652	0.183935	3.772936	1.882205	0.157955	3.760092	1.775885	0.189036
**H**	4.264221	2.793645	0.518654	4.275336	2.803360	0.484769	4.280582	2.677340	0.512868
**C**	4.647311	0.591769	0.197260	4.658231	0.598970	0.185509	4.615816	0.476488	0.202983

**C**	3.478307	-0.330430	-0.326171	3.491639	-0.328271	-0.331488	3.427324	-0.419887	-0.304770
**C**	3.028030	0.419802	-1.608749	3.044868	0.408368	-1.623123	2.986260	0.328127	-1.589910
**H**	1.981461	0.192005	-1.854100	1.998965	0.177944	-1.866767	1.942841	0.124523	-1.823996
**H**	3.632140	0.094530	-2.467136	3.652389	0.075183	-2.476475	3.574657	-0.010557	-2.442769
**C**	3.255683	1.924092	-1.273038	3.270431	1.916177	-1.301124	3.247125	1.828497	-1.264391
**H**	3.997732	2.380356	-1.941984	4.014573	2.366428	-1.972738	3.990649	2.260451	-1.934086
**H**	2.338186	2.524220	-1.363241	2.351658	2.513182	-1.397481	2.348690	2.440395	-1.352748
**C**	5.850008	0.652529	-0.753870	5.863848	0.651176	-0.762646	5.817357	0.507274	-0.751330
**H**	5.581276	0.853353	-1.798005	5.595698	0.844327	-1.808503	5.552881	0.699621	-1.788815
**H**	6.541495	1.445355	-0.430992	6.554922	1.446252	-0.442914	6.516417	1.286710	-0.438527
**H**	6.409567	-0.294574	-0.730967	6.420022	-0.298074	-0.731549	6.352821	-0.444212	-0.719937
**C**	5.188555	0.219660	1.587025	5.195903	0.241555	1.580508	5.163345	0.103844	1.590108
**H**	5.678170	-0.765499	1.561280	5.683132	-0.745327	1.565375	5.629500	-0.883653	1.563887
**H**	5.949871	0.950516	1.897858	5.956638	0.976669	1.884339	5.933268	0.820597	1.884932
**H**	4.427964	0.191452	2.378164	4.431223	0.222608	2.367547	4.415738	0.092949	2.381249
**C**	3.799038	-1.800022	-0.530203	3.812977	-1.799898	-0.519077	3.711850	-1.896312	-0.499973
**H**	4.092114	-2.287119	0.412764	4.110571	-2.272871	0.429560	4.007297	-2.373706	0.437351
**H**	2.934341	-2.345416	-0.936236	2.944911	-2.350692	-0.909400	2.833205	-2.419996	-0.878356
**H**	4.623090	-1.926048	-1.247296	4.632424	-1.933761	-1.240559	4.514678	-2.044154	-1.224697
**C**	-2.022250	0.410908	-1.960722	-2.061696	0.399957	-1.948206	-1.958597	0.417866	-1.968312
**H**	-0.993232	0.034527	-1.853270	-1.029688	0.028727	-1.852921	-0.963465	-0.011165	-1.851675
**C**	-2.455486	0.259059	-3.419469	-2.513373	0.226853	-3.399306	-2.387548	0.257102	-3.425143
**H**	-1.816163	0.877459	-4.063236	-1.878939	0.831552	-4.060793	-1.709235	0.812157	-4.073138
**H**	-2.381616	-0.787475	-3.746797	-2.442886	-0.826709	-3.704174	-2.372348	-0.794429	-3.714480
**H**	-3.493634	0.594184	-3.564508	-3.554022	0.557026	-3.536377	-3.396555	0.641517	-3.586548
**C**	-2.036661	1.851046	-1.477275	-2.071256	1.846362	-1.479769	-1.888659	1.861851	-1.508046
**C**	-3.208805	2.620988	-1.501633	-3.245607	2.613130	-1.497629	-2.993795	2.710800	-1.588680
**H**	-4.141154	2.198688	-1.886961	-4.179067	2.181509	-1.866867	-3.928047	2.354810	-2.006447
**C**	-3.200708	3.937743	-1.035985	-3.238104	3.931488	-1.035779	-2.911633	4.019750	-1.126178
**H**	-4.118342	4.528134	-1.059277	-4.159154	4.517197	-1.050814	-3.776453	4.668487	-1.189637
**C**	-2.016559	4.503497	-0.550383	-2.052830	4.502001	-0.557357	-1.719901	4.497872	-0.584629
**H**	-2.010187	5.534510	-0.192968	-2.047982	5.532188	-0.196861	-1.657208	5.517406	-0.225482
**C**	-0.842506	3.745892	-0.533345	-0.876789	3.746760	-0.544361	-0.612898	3.658965	-0.508110
**H**	0.086881	4.184327	-0.163293	0.051757	4.185109	-0.172156	0.316615	4.022873	-0.087019
**C**	-0.855571	2.425734	-0.992201	-0.888940	2.425797	-1.002451	-0.700883	2.347582	-0.965574
**H**	0.054550	1.822681	-0.960845	0.021784	1.823669	-0.970514	0.147437	1.681903	-0.878355
**C**	-3.756926	-2.105575	0.914537	-3.735476	-2.114762	0.956556	-3.824605	-1.970988	0.917852
**H**	-3.428031	-2.722716	1.759257	-3.385964	-2.732054	1.792362	-3.522717	-2.575529	1.770874
**H**	-4.512762	-1.388612	1.262049	-4.487767	-1.399302	1.314362	-4.556794	-1.228180	1.232784
**H**	-4.185946	-2.747705	0.135231	-4.175130	-2.751980	0.179546	-4.260244	-2.607142	0.149551

trans-N_{endo}-O_{exo}-**5**-*S*

Element	PBE/TZVP			PBE/TZVP (CHCl3)			B3LYP/TZVP (CHCl3)		
	X	y	z	x	y	z	x	y	z
**P**	1.406402	0.822177	1.070458	1.417737	0.842951	1.093004	1.408141	0.836935	1.079634
**O**	-1.800546	0.108239	0.232624	-1.805340	0.097447	0.250550	-1.774964	0.085985	0.209975
**N**	0.766674	-0.582362	0.401934	0.765715	-0.571979	0.448210	0.775981	-0.571370	0.431514
**C**	0.860511	-2.025326	0.780649	0.869647	-2.009701	0.836601	0.883738	-2.007299	0.825210
**P**	-0.408714	0.214032	-0.684388	-0.396293	0.207227	-0.654440	-0.375153	0.200466	-0.671682
**N**	0.427021	1.657970	0.007637	0.423071	1.658177	0.020958	0.425411	1.644583	0.008061
**C**	0.740752	-2.844151	-0.511945	0.757143	-2.841993	-0.448210	0.788812	-2.850203	-0.451410
**H**	0.777864	-3.916960	-0.278033	0.807110	-3.911657	-0.202384	0.860610	-3.908627	-0.198381
**H**	-0.213001	-2.651447	-1.026376	-0.199188	-2.664450	-0.963162	-0.162486	-2.698634	-0.964863
**H**	1.564334	-2.607231	-1.199017	1.576663	-2.600916	-1.138735	1.598052	-2.600226	-1.137085
**N**	3.027132	1.141704	0.906765	3.035708	1.135665	0.891021	3.013544	1.136713	0.885966
**H**	3.587415	1.114737	1.757774	3.603615	1.184219	1.736142	3.579588	1.189790	1.721880
**C**	2.228066	-2.285556	1.426084	2.237450	-2.257309	1.486768	2.242652	-2.249148	1.491327
**H**	2.307461	-3.351436	1.679564	2.320570	-3.322015	1.744083	2.322921	-3.303636	1.756236
**H**	3.052968	-2.031149	0.750428	3.060516	-2.001131	0.809244	3.065114	-2.000526	0.825609
**H**	2.350252	-1.726199	2.367230	2.354973	-1.690449	2.423259	2.350703	-1.678692	2.416688
**C**	-0.259166	-2.396718	1.764936	-0.248657	-2.380369	1.823255	-0.244369	-2.371265	1.798512
**H**	-0.187904	-3.461048	2.030520	-0.170126	-3.442538	2.095982	-0.165229	-3.421183	2.085408
**H**	-0.182009	-1.811634	2.693332	-0.174195	-1.784992	2.744806	-0.187184	-1.764974	2.704281
**H**	-1.248522	-2.213483	1.330326	-1.237840	-2.206149	1.383933	-1.218555	-2.208735	1.344555
**C**	0.315428	3.085191	-0.411511	0.306828	3.079768	-0.414045	0.293732	3.071147	-0.404553
**C**	1.117556	3.322791	-1.700909	1.066644	3.293907	-1.733004	1.047099	3.306524	-1.720373
**H**	0.953495	4.346003	-2.067775	0.893209	4.312731	-2.107707	0.874028	4.323832	-2.075043
**H**	2.195703	3.198589	-1.532739	2.149164	3.166313	-1.600092	2.121012	3.176456	-1.591764
**H**	0.802498	2.625998	-2.491283	0.721863	2.585705	-2.500442	0.703853	2.615612	-2.492300
**C**	0.836990	3.982418	0.719514	0.869413	3.988644	0.687741	0.855985	3.970548	0.702576
**H**	0.762381	5.035487	0.415633	0.789137	5.036872	0.368786	0.760972	5.014052	0.401051
**H**	0.239571	3.854964	1.633973	0.300834	3.875267	1.621951	0.302224	3.838784	1.633574
**H**	1.894063	3.777738	0.945241	1.931462	3.782191	0.884969	1.913682	3.775294	0.885855
**C**	-1.168653	3.385120	-0.662718	-1.182438	3.388330	-0.619717	-1.196113	3.374399	-0.596058
**H**	-1.291234	4.434414	-0.964447	-1.305684	4.437304	-0.922147	-1.326721	4.420746	-0.873977
**H**	-1.570933	2.760795	-1.475911	-1.614687	2.762688	-1.415996	-1.628084	2.767037	-1.394498
**H**	-1.765787	3.206920	0.240543	-1.748790	3.219513	0.305009	-1.750172	3.184820	0.321733
**C**	-3.081869	0.160998	-0.488677	-3.070423	0.153503	-0.485449	-3.047614	0.156221	-0.493905
**H**	-2.846611	0.277988	-1.560196	-2.824257	0.270179	-1.554431	-2.827092	0.288319	-1.555310
**C**	-3.984476	1.310616	0.031966	-3.980764	1.304625	0.021786	-3.945291	1.299703	0.045265
**H**	-3.534077	1.780864	0.917674	-3.539089	1.778969	0.909282	-3.493192	1.752342	0.927184
**H**	-4.118392	2.093776	-0.726857	-4.106497	2.084731	-0.741302	-4.077910	2.086394	-0.696523
**C**	-5.305800	0.588861	0.379850	-5.304993	0.584124	0.358227	-5.266429	0.576686	0.386168
**H**	-6.181567	1.251885	0.396544	-6.181905	1.246497	0.366496	-6.134937	1.235108	0.412169
**C**	-5.356897	-0.553313	-0.681417	-5.347684	-0.558256	-0.702723	-5.323523	-0.551659	-0.684717
**C**	-3.935710	-1.111440	-0.280937	-3.932172	-1.118115	-0.289307	-3.906253	-1.112722	-0.297882
**C**	-4.088806	-1.310391	1.252128	-4.101001	-1.319550	1.241280	-4.051576	-1.331870	1.231493

**H**	-3.117547	-1.243565	1.758967	-3.133141	-1.257870	1.754748	-3.086259	-1.269523	1.728387
**H**	-4.491356	-2.310663	1.464953	-4.510845	-2.318350	1.448458	-4.452259	-2.325430	1.433800
**C**	-5.064563	-0.179863	1.694200	-5.075861	-0.185002	1.675280	-5.023937	-0.206426	1.690454
**H**	-6.000634	-0.586304	2.100442	-6.016425	-0.588650	2.075451	-5.952173	-0.614211	2.090822
**H**	-4.634486	0.470477	2.469645	-4.646861	0.465492	2.451368	-4.591463	0.428717	2.465126
**C**	-6.490370	-1.564060	-0.461104	-6.486577	-1.565422	-0.493304	-6.461762	-1.559771	-0.476502
**H**	-6.465341	-2.050930	0.521243	-6.472956	-2.048543	0.491288	-6.433904	-2.063710	0.487180
**H**	-7.464588	-1.061552	-0.558439	-7.457833	-1.059051	-0.604328	-7.425508	-1.050923	-0.556835
**H**	-6.455527	-2.355351	-1.224924	-6.442173	-2.359486	-1.254114	-6.432522	-2.329952	-1.250498
**C**	-5.473330	-0.052544	-2.130189	-5.449173	-0.057288	-2.152487	-5.450089	-0.034418	-2.126798
**H**	-5.334734	-0.881282	-2.840953	-5.310536	-0.888912	-2.860183	-5.319043	-0.851761	-2.839628
**H**	-6.480823	0.354674	-2.301989	-6.452398	0.358865	-2.330753	-6.450523	0.376136	-2.281570
**H**	-4.758950	0.737504	-2.396443	-4.724148	0.724958	-2.411389	-4.739561	0.747809	-2.388397
**C**	-3.433302	-2.330746	-1.032520	-3.422569	-2.338032	-1.035146	-3.403406	-2.322842	-1.060314
**H**	-3.333825	-2.132338	-2.110927	-3.305996	-2.136434	-2.111192	-3.306054	-2.112471	-2.128034
**H**	-2.449562	-2.653348	-0.658763	-2.444629	-2.660343	-0.647086	-2.425615	-2.638365	-0.691637
**H**	-4.118549	-3.181195	-0.904844	-4.111803	-3.187215	-0.917697	-4.082822	-3.168726	-0.940189
**C**	3.785671	1.084470	-0.371637	3.767680	1.074150	-0.397987	3.742203	1.082382	-0.403991
**H**	3.035945	1.223861	-1.165083	3.007339	1.205179	-1.182342	2.989567	1.217340	-1.180444
**C**	4.777026	2.249620	-0.415987	4.755364	2.241058	-0.470746	4.725984	2.249794	-0.473451
**H**	5.316795	2.244229	-1.372281	5.276860	2.227533	-1.437056	5.241770	2.242037	-1.433759
**H**	4.260819	3.213525	-0.304805	4.236055	3.203297	-0.361452	4.206133	3.201566	-0.359552
**H**	5.525091	2.164174	0.386364	5.513772	2.165452	0.322267	5.478826	2.173979	0.313217
**C**	4.447219	-0.264041	-0.595987	4.431514	-0.274047	-0.626184	4.401480	-0.264696	-0.630281
**C**	5.438660	-0.739880	0.275105	5.437585	-0.743759	0.231785	5.412439	-0.723624	0.216748
**H**	5.763181	-0.135483	1.126948	5.769278	-0.136093	1.077733	5.753374	-0.109680	1.042403
**C**	6.023832	-1.990429	0.068021	6.019906	-1.995726	0.022359	5.983858	-1.975353	0.020413
**H**	6.792510	-2.351171	0.753640	6.796781	-2.353191	0.700748	6.762362	-2.322978	0.688032
**C**	5.632346	-2.776228	-1.021439	5.610368	-2.790132	-1.054877	5.556847	-2.781752	-1.032974
**H**	6.093435	-3.751481	-1.185552	6.065046	-3.768909	-1.217293	6.001152	-3.757298	-1.185130
**C**	4.657221	-2.303646	-1.903059	4.620243	-2.323162	-1.923167	4.559586	-2.326675	-1.888554
**H**	4.354628	-2.907792	-2.760311	4.297331	-2.935396	-2.767295	4.222274	-2.946946	-2.709618
**C**	4.067729	-1.054173	-1.688266	4.035223	-1.071331	-1.707461	3.985832	-1.074267	-1.685386
**H**	3.300579	-0.689305	-2.376735	3.254563	-0.711964	-2.382892	3.199012	-0.730242	-2.346796
**C**	1.081359	1.056772	2.846780	1.119220	1.114244	2.864117	1.091106	1.099020	2.842684
**H**	0.000553	0.933577	2.992131	0.037417	1.029718	3.026060	0.017326	1.007776	2.997445
**H**	1.615749	0.300924	3.438559	1.641614	0.352396	3.458069	1.612488	0.347318	3.434642
**H**	1.387373	2.062904	3.161023	1.464916	2.114394	3.154835	1.426620	2.093204	3.134477

8-*R*

cis-N_{endo}-O_{exo}-**8**-*R*

Elements	PBE/TZVP			PBE/TZVP (CHCl3)			B3LYP/TZVP (CHCl3)		
	X	y	z	x	y	z	x	y	z
**P**	-2.900583	-0.006851	-0.140280	-2.905759	-0.024625	-0.136537	-2.899312	0.111262	-0.159913
**P**	-1.179873	-1.788800	0.637379	-1.169167	-1.786179	0.650471	-1.290046	-1.726530	0.660286
**N**	-2.023525	-0.313103	1.257498	-2.007442	-0.311415	1.257050	-2.064111	-0.223868	1.246093
**N**	-2.207371	-1.404502	-0.763883	-2.191869	-1.421400	-0.751057	-2.264533	-1.318414	-0.752157
**N**	-2.543525	1.416194	-0.913096	-2.556359	1.399476	-0.905892	-2.447640	1.490428	-0.931885
**H**	-3.300487	2.032112	-1.201450	-3.321485	1.981524	-1.239463	-3.161566	2.110230	-1.285088
**C**	-2.241936	0.039118	2.691427	-2.222290	0.049207	2.689200	-2.248228	0.198256	2.664685
**C**	-2.716341	1.494595	2.791011	-2.724251	1.495811	2.780949	-2.708219	1.659195	2.712021
**H**	-2.859664	1.761747	3.846924	-2.869963	1.761767	3.836864	-2.819113	1.966761	3.752183
**H**	-3.679808	1.653991	2.284035	-3.689762	1.634992	2.273485	-3.675617	1.802261	2.228767
**H**	-1.977594	2.183341	2.360653	-1.997109	2.195376	2.348531	-1.982590	2.317830	2.238244
**C**	-3.276037	-0.907209	3.323510	-3.232887	-0.911815	3.336068	-3.285276	-0.703286	3.348916
**H**	-2.957589	-1.955439	3.227966	-2.886921	-1.952634	3.256036	-2.977215	-1.749508	3.311059
**H**	-4.262527	-0.799286	2.849247	-4.219523	-0.836056	2.857249	-4.260289	-0.613390	2.867041
**H**	-3.395492	-0.685911	4.393727	-3.355858	-0.673826	4.402265	-3.399614	-0.420930	4.396819
**C**	-0.888216	-0.106006	3.401091	-0.861814	-0.061659	3.391779	-0.888130	0.074967	3.360764
**H**	-0.518541	-1.142245	3.349043	-0.466915	-1.088707	3.342910	-0.527052	-0.956161	3.355939
**H**	-0.992615	0.150904	4.464117	-0.969695	0.198812	4.453656	-0.975831	0.385158	4.402522
**H**	-0.136671	0.555817	2.950446	-0.130341	0.617551	2.934186	-0.147014	0.704209	2.869248
**C**	-2.188005	-2.003424	-2.131905	-2.178670	-2.041291	-2.107918	-2.269156	-1.934472	-2.109915
**C**	-1.089100	-1.358851	-2.990184	-1.072542	-1.420529	-2.975403	-1.093247	-1.405574	-2.940311
**H**	-0.105328	-1.479679	-2.520494	-0.089285	-1.550782	-2.507270	-0.146465	-1.634430	-2.456769
**H**	-1.277130	-0.284855	-3.129765	-1.246407	-0.345309	-3.122316	-1.160975	-0.324560	-3.066511
**H**	-1.061528	-1.829182	-3.983352	-1.056297	-1.901391	-3.963944	-1.098624	-1.865186	-3.930048
**C**	-3.560733	-1.785268	-2.783173	-3.546724	-1.819316	-2.767444	-3.594177	-1.596234	-2.802687
**H**	-4.366094	-2.233168	-2.183610	-4.358864	-2.243038	-2.160184	-4.446705	-1.946623	-2.219268
**H**	-3.575044	-2.257019	-3.775095	-3.560723	-2.313271	-3.748585	-3.626264	-2.084269	-3.776976
**H**	-3.768656	-0.714331	-2.934674	-3.740260	-0.749723	-2.941808	-3.692755	-0.522191	-2.976652
**C**	-1.927530	-3.506661	-1.965651	-1.933515	-3.544770	-1.919031	-2.157838	-3.452918	-1.933828
**H**	-2.712576	-3.981765	-1.360822	-2.726677	-3.999947	-1.309406	-2.990640	-3.838780	-1.344390
**H**	-0.955295	-3.696439	-1.486037	-0.965519	-3.736862	-1.431531	-1.225072	-3.733102	-1.440381
**H**	-1.906085	-3.990128	-2.951856	-1.913527	-4.042242	-2.898276	-2.168857	-3.936087	-2.911414
**O**	0.261858	-1.166867	0.082916	0.278821	-1.165992	0.087368	0.179429	-1.189379	0.125745
**C**	1.511751	-1.577335	0.735461	1.523648	-1.572553	0.735909	1.405531	-1.618554	0.776133
**H**	1.259362	-2.373918	1.455673	1.278433	-2.373947	1.452914	1.141666	-2.390839	1.501973
**C**	2.218221	-0.376627	1.418263	2.229744	-0.373577	1.424716	2.146944	-0.431200	1.441411
**H**	1.685074	0.560553	1.203577	1.688663	0.561079	1.221119	1.637498	0.505866	1.221808
**H**	2.253424	-0.499419	2.509759	2.270807	-0.504591	2.514908	2.185093	-0.542036	2.524779
**C**	3.622323	-0.390266	0.775246	3.629819	-0.373099	0.773737	3.544324	-0.487098	0.789445
**H**	4.387013	0.127586	1.370389	4.395488	0.146676	1.366702	4.320568	0.014126	1.367977
**C**	3.874071	-1.913699	0.548127	3.891558	-1.892831	0.536101	3.757708	-2.014904	0.575545

**C**	2.547942	-2.099433	-0.287043	2.563119	-2.083958	-0.291858	2.424839	-2.176098	-0.242638
**C**	2.702200	-1.004482	-1.376844	2.703686	-0.983571	-1.377927	2.595542	-1.100965	-1.347229
**H**	3.264209	-1.404613	-2.232418	3.265700	-1.374930	-2.238012	3.138844	-1.518951	-2.195212
**H**	1.724975	-0.684417	-1.761058	1.721031	-0.670598	-1.753192	1.631831	-0.763573	-1.721079
**C**	3.462473	0.149349	-0.661360	3.457975	0.172700	-0.659711	3.386620	0.041671	-0.650292
**H**	4.437439	0.345323	-1.128283	4.428995	0.380391	-1.131091	4.354489	0.210312	-1.122708
**H**	2.906234	1.096058	-0.676608	2.891813	1.113698	-0.664754	2.853174	0.989950	-0.670536
**C**	3.941127	-2.737701	1.844208	3.972083	-2.722886	1.827459	3.820777	-2.826840	1.879235
**H**	3.967302	-3.814594	1.618834	4.008462	-3.798099	1.594237	3.824791	-3.897641	1.662704
**H**	3.106816	-2.563901	2.536474	3.136325	-2.561265	2.520290	3.000798	-2.634385	2.568955
**H**	4.866623	-2.499294	2.389325	4.896985	-2.475238	2.370485	4.747942	-2.597803	2.409835
**C**	5.146559	-2.224446	-0.250524	5.162934	-2.189193	-0.270247	5.019371	-2.365547	-0.224192
**H**	5.242668	-3.307574	-0.419327	5.261792	-3.270606	-0.450172	5.083973	-3.445031	-0.378929
**H**	6.032623	-1.904831	0.318589	6.049127	-1.869508	0.299469	5.907554	-2.061303	0.335125
**H**	5.186174	-1.731276	-1.229098	5.193987	-1.686033	-1.244191	5.067236	-1.890670	-1.201607
**C**	2.244299	-3.485895	-0.825055	2.265873	-3.469854	-0.835033	2.077815	-3.557401	-0.762517
**H**	3.046180	-3.834025	-1.492100	3.063005	-3.806480	-1.514210	2.849850	-3.924789	-1.441244
**H**	1.313598	-3.486082	-1.413664	1.326490	-3.473650	-1.408958	1.137701	-3.537004	-1.318342
**H**	2.130464	-4.223215	-0.015407	2.167740	-4.210995	-0.026794	1.968778	-4.277926	0.051648
**C**	-1.158608	1.875297	-1.127046	-1.178960	1.864559	-1.130833	-1.038987	1.859449	-1.153563
**H**	-0.988210	2.000871	-2.208559	-1.008457	1.974876	-2.214142	-0.882360	2.006649	-2.224139
**H**	-0.487629	1.072944	-0.785797	-0.498906	1.077245	-0.773370	-0.420330	1.015445	-0.848391
**C**	-0.832273	3.181785	-0.378134	-0.868533	3.188172	-0.402991	-0.623337	3.116832	-0.371278
**H**	-1.103359	3.017174	0.677557	-1.151220	3.043061	0.651551	-0.869352	2.931071	0.675494
**C**	-1.649396	4.369901	-0.905789	-1.688688	4.358928	-0.963551	-1.397058	4.358790	-0.833514
**H**	-1.398390	5.286334	-0.355230	-1.459210	5.286024	-0.420839	-1.080803	5.237963	-0.271177
**H**	-2.733369	4.208455	-0.788430	-2.770566	4.179759	-0.863546	-2.472524	4.240368	-0.680448
**H**	-1.444627	4.554410	-1.971060	-1.468560	4.527408	-2.028296	-1.222990	4.556512	-1.893585
**C**	0.663440	3.441557	-0.418930	0.625216	3.457156	-0.430947	0.877693	3.320921	-0.446466
**C**	1.406293	3.456417	0.770884	1.361920	3.458734	0.763467	1.644830	3.309460	0.720208
**H**	0.896623	3.284352	1.723298	0.847718	3.265137	1.709118	1.159212	3.139883	1.674613
**C**	2.781548	3.705086	0.757646	2.737415	3.709445	0.760889	3.020615	3.516251	0.676520
**H**	3.340302	3.720207	1.695302	3.291294	3.707459	1.701804	3.596509	3.501334	1.593659
**C**	3.437336	3.937777	-0.453676	3.400785	3.958092	-0.443863	3.653712	3.734283	-0.542482
**H**	4.510646	4.134131	-0.468342	4.474946	4.151680	-0.450303	4.724450	3.890638	-0.581194
**C**	2.710422	3.919036	-1.647789	2.680273	3.950214	-1.642747	2.901855	3.741979	-1.714364
**H**	3.216060	4.101522	-2.597759	3.191737	4.138941	-2.588707	3.387045	3.907421	-2.668505
**C**	1.335043	3.674459	-1.630039	1.304712	3.703036	-1.635608	1.526323	3.539330	-1.665672
**H**	0.781922	3.680537	-2.572643	0.756365	3.710353	-2.580721	0.956357	3.562240	-2.586847
**C**	-4.714214	-0.036149	-0.020602	-4.714186	-0.077386	-0.011935	-4.703539	0.157359	-0.059006
**H**	-5.159435	0.067333	-1.018498	-5.159032	0.024296	-1.010047	-5.128077	0.256765	-1.056601
**H**	-5.066464	0.776948	0.627267	-5.072500	0.735065	0.632942	-5.025874	0.994581	0.557499
**H**	-5.008555	-1.002013	0.407006	-4.996504	-1.046667	0.415399	-5.035709	-0.779302	0.384666

trans-N_{endo}-O_{exo}-**8**-*R*

Elements	PBE/TZVP			PBE/TZVP (CHCl3)			B3LYP/TZVP (CHCl3)		
	X	y	z	x	y	z	x	y	z
P	-2.900583	-0.006851	-0.140280	-2.905759	-0.024625	-0.136537	-2.899312	0.111262	-0.159913
P	-1.179873	-1.788800	0.637379	-1.169167	-1.786179	0.650471	-1.290046	-1.726530	0.660286
N	-2.023525	-0.313103	1.257498	-2.007442	-0.311415	1.257050	-2.064111	-0.223868	1.246093
N	-2.207371	-1.404502	-0.763883	-2.191869	-1.421400	-0.751057	-2.264533	-1.318414	-0.752157
N	-2.543525	1.416194	-0.913096	-2.556359	1.399476	-0.905892	-2.447640	1.490428	-0.931885
H	-3.300487	2.032112	-1.201450	-3.321485	1.981524	-1.239463	-3.161566	2.110230	-1.285088
C	-2.241936	0.039118	2.691427	-2.222290	0.049207	2.689200	-2.248228	0.198256	2.664685
C	-2.716341	1.494595	2.791011	-2.724251	1.495811	2.780949	-2.708219	1.659195	2.712021
H	-2.859664	1.761747	3.846924	-2.869963	1.761767	3.836864	-2.819113	1.966761	3.752183
H	-3.679808	1.653991	2.284035	-3.689762	1.634992	2.273485	-3.675617	1.802261	2.228767
H	-1.977594	2.183341	2.360653	-1.997109	2.195376	2.348531	-1.982590	2.317830	2.238244
C	-3.276037	-0.907209	3.323510	-3.232887	-0.911815	3.336068	-3.285276	-0.703286	3.348916
H	-2.957589	-1.955439	3.227966	-2.886921	-1.952634	3.256036	-2.977215	-1.749508	3.311059
H	-4.262527	-0.799286	2.849247	-4.219523	-0.836056	2.857249	-4.260289	-0.613390	2.867041
H	-3.395492	-0.685911	4.393727	-3.355858	-0.673826	4.402265	-3.399614	-0.420930	4.396819
C	-0.888216	-0.106006	3.401091	-0.861814	-0.061659	3.391779	-0.888130	0.074967	3.360764
H	-0.518541	-1.142245	3.349043	-0.466915	-1.088707	3.342910	-0.527052	-0.956161	3.355939
H	-0.992615	0.150904	4.464117	-0.969695	0.198812	4.453656	-0.975831	0.385158	4.402522
H	-0.136671	0.555817	2.950446	-0.130341	0.617551	2.934186	-0.147014	0.704209	2.869248
C	-2.188005	-2.003424	-2.131905	-2.178670	-2.041291	-2.107918	-2.269156	-1.934472	-2.109915
C	-1.089100	-1.358851	-2.990184	-1.072542	-1.420529	-2.975403	-1.093247	-1.405574	-2.940311
H	-0.105328	-1.479679	-2.520494	-0.089285	-1.550782	-2.507270	-0.146465	-1.634430	-2.456769
H	-1.277130	-0.284855	-3.129765	-1.246407	-0.345309	-3.122316	-1.160975	-0.324560	-3.066511
H	-1.061528	-1.829182	-3.983352	-1.056297	-1.901391	-3.963944	-1.098624	-1.865186	-3.930048
C	-3.560733	-1.785268	-2.783173	-3.546724	-1.819316	-2.767444	-3.594177	-1.596234	-2.802687
H	-4.366094	-2.233168	-2.183610	-4.358864	-2.243038	-2.160184	-4.446705	-1.946623	-2.219268
H	-3.575044	-2.257019	-3.775095	-3.560723	-2.313271	-3.748585	-3.626264	-2.084269	-3.776976
H	-3.768656	-0.714331	-2.934674	-3.740260	-0.749723	-2.941808	-3.692755	-0.522191	-2.976652
C	-1.927530	-3.506661	-1.965651	-1.933515	-3.544770	-1.919031	-2.157838	-3.452918	-1.933828
H	-2.712576	-3.981765	-1.360822	-2.726677	-3.999947	-1.309406	-2.990640	-3.838780	-1.344390
H	-0.955295	-3.696439	-1.486037	-0.965519	-3.736862	-1.431531	-1.225072	-3.733102	-1.440381
H	-1.906085	-3.990128	-2.951856	-1.913527	-4.042242	-2.898276	-2.168857	-3.936087	-2.911414
O	0.261858	-1.166867	0.082916	0.278821	-1.165992	0.087368	0.179429	-1.189379	0.125745
C	1.511751	-1.577335	0.735461	1.523648	-1.572553	0.735909	1.405531	-1.618554	0.776133
H	1.259362	-2.373918	1.455673	1.278433	-2.373947	1.452914	1.141666	-2.390839	1.501973
C	2.218221	-0.376627	1.418263	2.229744	-0.373577	1.424716	2.146944	-0.431200	1.441411
H	1.685074	0.560553	1.203577	1.688663	0.561079	1.221119	1.637498	0.505866	1.221808
H	2.253424	-0.499419	2.509759	2.270807	-0.504591	2.514908	2.185093	-0.542036	2.524779
C	3.622323	-0.390266	0.775246	3.629819	-0.373099	0.773737	3.544324	-0.487098	0.789445
H	4.387013	0.127586	1.370389	4.395488	0.146676	1.366702	4.320568	0.014126	1.367977
C	3.874071	-1.913699	0.548127	3.891558	-1.892831	0.536101	3.757708	-2.014904	0.575545
C	2.547942	-2.099433	-0.287043	2.563119	-2.083958	-0.291858	2.424839	-2.176098	-0.242638
C	2.702200	-1.004482	-1.376844	2.703686	-0.983571	-1.377927	2.595542	-1.100965	-1.347229

H	3.264209	-1.404613	-2.232418	3.265700	-1.374930	-2.238012	3.138844	-1.518951	-2.195212
H	1.724975	-0.684417	-1.761058	1.721031	-0.670598	-1.753192	1.631831	-0.763573	-1.721079
C	3.462473	0.149349	-0.661360	3.457975	0.172700	-0.659711	3.386620	0.041671	-0.650292
H	4.437439	0.345323	-1.128283	4.428995	0.380391	-1.131091	4.354489	0.210312	-1.122708
H	2.906234	1.096058	-0.676608	2.891813	1.113698	-0.664754	2.853174	0.989950	-0.670536
C	3.941127	-2.737701	1.844208	3.972083	-2.722886	1.827459	3.820777	-2.826840	1.879235
H	3.967302	-3.814594	1.618834	4.008462	-3.798099	1.594237	3.824791	-3.897641	1.662704
H	3.106816	-2.563901	2.536474	3.136325	-2.561265	2.520290	3.000798	-2.634385	2.568955
H	4.866623	-2.499294	2.389325	4.896985	-2.475238	2.370485	4.747942	-2.597803	2.409835
C	5.146559	-2.224446	-0.250524	5.162934	-2.189193	-0.270247	5.019371	-2.365547	-0.224192
H	5.242668	-3.307574	-0.419327	5.261792	-3.270606	-0.450172	5.083973	-3.445031	-0.378929
H	6.032623	-1.904831	0.318589	6.049127	-1.869508	0.299469	5.907554	-2.061303	0.335125
H	5.186174	-1.731276	-1.229098	5.193987	-1.686033	-1.244191	5.067236	-1.890670	-1.201607
C	2.244299	-3.485895	-0.825055	2.265873	-3.469854	-0.835033	2.077815	-3.557401	-0.762517
H	3.046180	-3.834025	-1.492100	3.063005	-3.806480	-1.514210	2.849850	-3.924789	-1.441244
H	1.313598	-3.486082	-1.413664	1.326490	-3.473650	-1.408958	1.137701	-3.537004	-1.318342
H	2.130464	-4.223215	-0.015407	2.167740	-4.210995	-0.026794	1.968778	-4.277926	0.051648
C	-1.158608	1.875297	-1.127046	-1.178960	1.864559	-1.130833	-1.038987	1.859449	-1.153563
H	-0.988210	2.000871	-2.208559	-1.008457	1.974876	-2.214142	-0.882360	2.006649	-2.224139
H	-0.487629	1.072944	-0.785797	-0.498906	1.077245	-0.773370	-0.420330	1.015445	-0.848391
C	-0.832273	3.181785	-0.378134	-0.868533	3.188172	-0.402991	-0.623337	3.116832	-0.371278
H	-1.103359	3.017174	0.677557	-1.151220	3.043061	0.651551	-0.869352	2.931071	0.675494
C	-1.649396	4.369901	-0.905789	-1.688688	4.358928	-0.963551	-1.397058	4.358790	-0.833514
H	-1.398390	5.286334	-0.355230	-1.459210	5.286024	-0.420839	-1.080803	5.237963	-0.271177
H	-2.733369	4.208455	-0.788430	-2.770566	4.179759	-0.863546	-2.472524	4.240368	-0.680448
H	-1.444627	4.554410	-1.971060	-1.468560	4.527408	-2.028296	-1.222990	4.556512	-1.893585
C	0.663440	3.441557	-0.418930	0.625216	3.457156	-0.430947	0.877693	3.320921	-0.446466
C	1.406293	3.456417	0.770884	1.361920	3.458734	0.763467	1.644830	3.309460	0.720208
H	0.896623	3.284352	1.723298	0.847718	3.265137	1.709118	1.159212	3.139883	1.674613
C	2.781548	3.705086	0.757646	2.737415	3.709445	0.760889	3.020615	3.516251	0.676520
H	3.340302	3.720207	1.695302	3.291294	3.707459	1.701804	3.596509	3.501334	1.593659
C	3.437336	3.937777	-0.453676	3.400785	3.958092	-0.443863	3.653712	3.734283	-0.542482
H	4.510646	4.134131	-0.468342	4.474946	4.151680	-0.450303	4.724450	3.890638	-0.581194
C	2.710422	3.919036	-1.647789	2.680273	3.950214	-1.642747	2.901855	3.741979	-1.714364
H	3.216060	4.101522	-2.597759	3.191737	4.138941	-2.588707	3.387045	3.907421	-2.668505
C	1.335043	3.674459	-1.630039	1.304712	3.703036	-1.635608	1.526323	3.539330	-1.665672
H	0.781922	3.680537	-2.572643	0.756365	3.710353	-2.580721	0.956357	3.562240	-2.586847
C	-4.714214	-0.036149	-0.020602	-4.714186	-0.077386	-0.011935	-4.703539	0.157359	-0.059006
H	-5.159435	0.067333	-1.018498	-5.159032	0.024296	-1.010047	-5.128077	0.256765	-1.056601
H	-5.066464	0.776948	0.627267	-5.072500	0.735065	0.632942	-5.025874	0.994581	0.557499
H	-5.008555	-1.002013	0.407006	-4.996504	-1.046667	0.415399	-5.035709	-0.779302	0.384666

8-*S*

cis-N$_{endo}$-O$_{exo}$-**8**-*S*

Element	PBE/TZVP			PBE/TZVP (CHCl3)			B3LYP/TZVP (CHCl3)		
	X	y	z	x	y	z	x	y	z
**P**	2.854941	0.185319	0.245598	-2.854318	0.004220	-0.238402	-2.831713	0.418472	-0.232669
**P**	1.364868	-1.84685	-0.37079	-1.229797	-1.933378	0.342162	-1.564651	-1.676991	0.557294
**O**	-0.15619	-1.25468	-0.03981	0.269236	-1.256989	0.033498	-0.025994	-1.289062	0.088058
**N**	2.160016	-0.41907	-1.15039	-2.096334	-0.570758	1.142913	-2.172228	-0.117491	1.197595
**N**	2.240377	-1.17706	1.026073	-2.130639	-1.292635	-1.043254	-2.429142	-1.084969	-0.861657
**N**	2.280597	1.698917	0.627087	-2.404088	1.563228	-0.594647	-2.063334	1.766430	-0.795536
**H**	2.867615	2.306542	1.197576	-3.041846	2.116293	-1.167388	-2.549263	2.337968	-1.474677
**C**	2.488869	-0.25475	-2.59698	-2.428295	-0.446546	2.592366	-2.392333	0.273648	2.618809
**C**	2.750916	1.229006	-2.88407	-2.732939	1.023343	2.906138	-2.436595	1.801780	2.715468
**H**	1.878725	1.841444	-2.62091	-1.870166	1.659041	2.668433	-1.509198	2.239315	2.349132
**H**	2.960534	1.366023	-3.95378	-2.960706	1.130451	3.975611	-2.570025	2.097321	3.756523
**H**	3.620786	1.611684	-2.3295	-3.602009	1.395553	2.343819	-3.265403	2.225994	2.146536
**C**	3.714144	-1.10613	-2.9668	-3.626840	-1.339230	2.950865	-3.700296	-0.340590	3.135625
**H**	3.540818	-2.16543	-2.7275	-3.421443	-2.388959	2.695622	-3.680128	-1.427266	3.036357
**H**	4.613832	-0.77116	-2.43005	-4.536000	-1.022796	2.420209	-4.561912	0.039303	2.583900
**H**	3.92309	-1.03124	-4.04349	-3.832015	-1.283298	4.029571	-3.842837	-0.096312	4.189657
**C**	1.262501	-0.72244	-3.39326	-1.187520	-0.891006	3.378958	-1.204554	-0.251529	3.433003
**H**	0.376485	-0.12567	-3.13912	-0.322439	-0.259458	3.137575	-0.268987	0.175732	3.074008
**H**	1.039859	-1.78376	-3.20043	-0.930263	-1.940495	3.165513	-1.135827	-1.340851	3.384640
**H**	1.456002	-0.61949	-4.46968	-1.386360	-0.813497	4.456436	-1.329902	0.021488	4.481150
**C**	2.127912	-1.55731	2.464596	-2.004993	-1.649388	-2.484800	-2.439289	-1.621359	-2.251341
**C**	0.922899	-0.86627	3.120849	-0.858905	-0.862364	-3.139941	-1.216897	-1.122893	-3.032410
**H**	-0.0061	-1.11467	2.593472	0.087780	-1.041422	-2.615239	-0.296843	-1.427430	-2.538962
**H**	1.043645	0.227086	3.113764	-1.065680	0.217656	-3.123599	-1.221977	-0.034372	-3.112601
**H**	0.826937	-1.1859	4.168227	-0.742361	-1.167820	-4.189532	-1.224644	-1.534794	-4.042937
**C**	3.421824	-1.1488	3.182585	-3.331481	-1.332576	-3.189171	-3.728690	-1.163925	-2.943479
**H**	4.303102	-1.61785	2.722605	-4.169611	-1.870562	-2.724598	-4.610140	-1.472683	-2.379808
**H**	3.376202	-1.46754	4.232833	-3.267964	-1.638744	-4.242291	-3.784192	-1.606755	-3.938162
**H**	3.552538	-0.05475	3.187869	-3.545722	-0.252849	-3.180330	-3.751024	-0.079598	-3.074578
**C**	1.967882	-3.08288	2.519804	-1.732129	-3.158141	-2.558976	-2.425715	-3.151092	-2.158213
**H**	2.82905	-3.58909	2.061668	-2.554822	-3.729758	-2.107189	-3.292524	-3.515394	-1.605134
**H**	1.054416	-3.41154	2.001483	-0.797538	-3.425480	-2.043156	-1.521904	-3.515267	-1.666191
**H**	1.886547	-3.4096	3.565452	-1.627796	-3.462624	-3.609258	-2.447436	-3.578909	-3.160992
**C**	-1.32235	-1.86359	-0.68713	1.453443	-1.850402	0.647192	1.135005	-1.818783	0.778155
**H**	-0.97293	-2.76712	-1.21461	1.145840	-2.800951	1.113964	0.788458	-2.586805	1.473160
**C**	-2.03034	-0.86768	-1.64516	2.116581	-0.891750	1.674427	1.933908	-0.704570	1.505856
**H**	-1.56394	0.126399	-1.57953	1.606206	0.082219	1.673415	1.501148	0.274036	1.301336
**H**	-1.96424	-1.19995	-2.69056	2.059820	-1.296609	2.694316	1.926497	-0.851258	2.585492
**C**	-3.48271	-0.85063	-1.11848	3.568723	-0.776539	1.160193	3.348012	-0.840714	0.903559
**H**	-4.2195	-0.51561	-1.86155	4.289078	-0.461350	1.928246	4.133666	-0.409111	1.524040
**C**	-3.67208	-2.31349	-0.6085	3.824309	-2.191089	0.553385	3.462696	-2.374415	0.658243

**C**	-2.41434	-2.2405	0.341414	2.567995	-2.109909	-0.395932	2.155704	-2.422158	-0.214146
**C**	-2.71516	-0.96366	1.172734	2.812699	-0.767956	-1.138574	2.445254	-1.336194	-1.282530
**H**	-3.32155	-1.22284	2.051951	3.431423	-0.938883	-2.031095	2.991294	-1.771246	-2.120039
**H**	-1.79045	-0.50606	1.547527	1.867061	-0.329400	-1.483037	1.522669	-0.923483	-1.682864
**C**	-3.47754	-0.03034	0.188073	3.532958	0.128306	-0.089086	3.285142	-0.268712	-0.526870
**H**	-2.98105	0.940824	0.05598	2.992713	1.065265	0.104486	2.821596	0.716048	-0.543712
**H**	-4.49846	0.179994	0.535018	4.545500	0.403680	-0.416390	4.280702	-0.157877	-0.956564
**C**	-3.58537	-3.37796	-1.71426	3.782356	-3.330494	1.584262	3.416732	-3.220655	1.940375
**H**	-2.70982	-3.2818	-2.36972	2.900791	-3.314552	2.237775	2.583746	-2.990247	2.602301
**H**	-4.47625	-3.32004	-2.35728	4.669153	-3.276384	2.234033	4.334554	-3.069915	2.513535
**H**	-3.56607	-4.3888	-1.27951	3.806220	-4.308966	1.080391	3.356666	-4.283185	1.693804
**C**	-4.98995	-2.5477	0.141439	5.153733	-2.315193	-0.203006	4.728676	-2.793173	-0.101259
**H**	-5.84026	-2.4018	-0.54181	5.994487	-2.174492	0.493729	5.612033	-2.566103	0.500726
**H**	-5.13593	-1.88007	0.999017	5.268183	-1.586892	-1.014996	4.849726	-2.298879	-1.062589
**H**	-5.04529	-3.5824	0.511826	5.255407	-3.320238	-0.640165	4.724687	-3.870398	-0.282835
**C**	-2.08857	-3.46979	1.170072	2.302340	-3.293065	-1.308805	1.736748	-3.762673	-0.784782
**H**	-1.21548	-3.29054	1.816529	1.425773	-3.107113	-1.947690	0.830535	-3.660815	-1.385853
**H**	-1.86523	-4.34253	0.537157	2.112126	-4.214053	-0.736364	1.535669	-4.491064	0.004453
**H**	-2.92787	-3.73367	1.829656	3.157615	-3.474567	-1.976319	2.514313	-4.170038	-1.433821
**C**	0.839674	2.024994	0.573857	-0.997046	2.000930	-0.556146	-0.596493	1.906784	-0.729896
**H**	0.31841	1.62893	1.459875	-0.465506	1.687964	-1.469325	-0.114651	1.308068	-1.506004
**H**	0.419817	1.503898	-0.29901	-0.517253	1.482790	0.287148	-0.277637	1.496951	0.226851
**C**	0.575002	3.538209	0.46523	-0.861666	3.526857	-0.392747	-0.133521	3.367294	-0.854049
**H**	0.946774	4.006854	1.395053	-1.305077	3.994039	-1.290041	-0.358672	3.699298	-1.872757
**C**	1.292953	4.194957	-0.71933	-1.593504	4.071950	0.840399	-0.847429	4.314388	0.117921
**H**	1.075679	5.270962	-0.74702	-1.514303	5.167122	0.874800	-0.483386	5.333299	-0.018786
**H**	0.971357	3.765839	-1.67906	-1.164665	3.676759	1.772198	-0.673447	4.034167	1.157185
**H**	2.3831	4.069102	-0.65231	-2.659148	3.803477	0.823887	-1.925250	4.312916	-0.047173
**C**	-0.93569	3.729658	0.442285	0.621514	3.865289	-0.393422	1.378093	3.387624	-0.688733
**C**	-1.6788	3.528779	-0.73179	1.437570	3.535252	0.701367	1.966979	3.207286	0.567024
**H**	-1.17299	3.271048	-1.66528	1.004838	3.046619	1.578091	1.345014	3.066888	1.442835
**C**	-3.06873	3.669318	-0.73048	2.804307	3.823884	0.688237	3.349555	3.202487	0.713339
**H**	-3.62943	3.518168	-1.65494	3.422341	3.560053	1.548900	3.785523	3.058717	1.694336
**C**	-3.74038	4.00409	0.448677	3.381592	4.443351	-0.425085	4.171278	3.371358	-0.397646
**H**	-4.82575	4.117361	0.448751	4.449575	4.668719	-0.435655	5.248219	3.364595	-0.285268
**C**	-3.01285	4.198587	1.625365	2.581389	4.771751	-1.522896	3.598596	3.541606	-1.653587
**H**	-3.5276	4.466661	2.549819	3.021858	5.256643	-2.396437	4.228633	3.670483	-2.525153
**C**	-1.62202	4.061829	1.618991	1.212946	4.483117	-1.504567	2.213028	3.548548	-1.795083
**H**	-1.05854	4.227106	2.54164	0.591972	4.744908	-2.365516	1.775854	3.685069	-2.777885
**C**	4.664244	0.334916	0.325563	-4.665526	0.004308	-0.300118	-4.595408	0.801452	-0.287295
**H**	4.972868	0.683674	1.319961	-5.009106	0.404290	-1.262770	-4.871703	1.122669	-1.290396
**H**	5.012345	1.044924	-0.43545	-5.060458	0.626678	0.512810	-4.813121	1.602942	0.416466
**H**	5.097925	-0.65527	0.142507	-5.011916	-1.029701	-0.189165	-5.151952	-0.095619	-0.025697

trans-N_{endo}-O_{exo}-**8**-*S*

Element	PBE/TZVP			PBE/TZVP (CHCl3)			B3LYP/TZVP (CHCl3)		
	X	y	z	x	y	z	x	y	z
P	0.809632	-0.031261	1.409562	-0.817569	0.062433	-1.474827	-0.803501	0.092925	-1.454470
P	-0.777940	0.195661	-0.627302	0.749405	0.186464	0.587462	0.729355	0.182128	0.613627
O	-2.269648	0.024821	0.105079	2.261964	0.017514	-0.120028	2.234856	0.042260	-0.067190
N	0.149028	-1.059080	0.260117	-0.171059	-1.018065	-0.361887	-0.164091	-0.999709	-0.367466
N	0.128811	1.226833	0.535296	-0.112684	1.273161	-0.545206	-0.121898	1.280020	-0.499200
N	2.459040	-0.127571	1.556573	-2.469216	-0.011822	-1.598308	-2.442243	0.032083	-1.585635
H	2.837292	0.012106	2.492668	-2.861458	0.222460	-2.510458	-2.835002	0.250528	-2.491671
C	-0.032411	-2.541026	0.203559	-0.020733	-2.503175	-0.350934	-0.027590	-2.484587	-0.387403
C	1.259138	-3.215415	0.682447	-1.303236	-3.139047	-0.901272	-1.305662	-3.094349	-0.970705
H	2.116891	-2.916220	0.066026	-2.176990	-2.849111	-0.303750	-2.178502	-2.804350	-0.388314
H	1.148282	-4.306173	0.613007	-1.207068	-4.232808	-0.863446	-1.225430	-4.181572	-0.952899
H	1.485550	-2.974563	1.731903	-1.486206	-2.859685	-1.949274	-1.466067	-2.794508	-2.007671
C	-1.217104	-2.983835	1.076733	1.187468	-2.937992	-1.195063	1.186987	-2.898811	-1.227660
H	-2.140669	-2.475273	0.775643	2.110436	-2.473499	-0.828517	2.098155	-2.450842	-0.839098
H	-1.034045	-2.763175	2.138735	1.055308	-2.655163	-2.249293	1.068630	-2.582873	-2.265529
H	-1.368685	-4.068654	0.984671	1.302327	-4.030328	-1.149584	1.297440	-3.984302	-1.215725
C	-0.281541	-2.901087	-1.267675	0.165360	-2.920995	1.114345	0.136454	-2.948915	1.064128
H	0.568102	-2.599887	-1.896452	-0.705581	-2.629533	1.718010	-0.728004	-2.659233	1.663206
H	-1.190367	-2.417973	-1.656766	1.063738	-2.464571	1.556544	1.032340	-2.529481	1.525160
H	-0.420093	-3.986403	-1.365103	0.285682	-4.011169	1.174095	0.231442	-4.034818	1.091731
C	0.372768	2.696528	0.460095	-0.324407	2.744325	-0.429990	-0.306219	2.754911	-0.394591
C	1.187142	3.021539	-0.802055	-1.122076	3.053954	0.846803	-1.089247	3.082107	0.883892
H	0.660595	2.684305	-1.706707	-0.597892	2.677852	1.737464	-0.563797	2.717881	1.768397
H	2.173440	2.537835	-0.779183	-2.120059	2.595594	0.815215	-2.080266	2.628291	0.864178
H	1.340963	4.106583	-0.887408	-1.246912	4.140123	0.960502	-1.208850	4.161957	0.984473
C	1.133494	3.141222	1.716780	-1.087800	3.236077	-1.667439	-1.073470	3.253898	-1.624672
H	0.558672	2.926732	2.630071	-0.524461	3.036692	-2.590816	-0.522608	3.051470	-2.545130
H	1.298300	4.226437	1.678354	-1.234089	4.322176	-1.594009	-1.207421	4.333193	-1.550476
H	2.121744	2.663056	1.787220	-2.083064	2.773935	-1.742696	-2.065111	2.803495	-1.693435
C	-0.987781	3.404502	0.402524	1.050604	3.423722	-0.366460	1.076505	3.413886	-0.350280
H	-1.594315	3.166059	1.286556	1.644683	3.189557	-1.260045	1.650707	3.165706	-1.242939
H	-1.552366	3.107520	-0.493945	1.613930	3.097786	0.520549	1.642555	3.088151	0.524068
H	-0.845134	4.493187	0.356644	0.928527	4.513852	-0.299216	0.972038	4.498117	-0.290764
C	-3.450643	0.468359	-0.650819	3.423572	0.434112	0.666655	3.405126	0.417372	0.712170
H	-3.110204	0.709691	-1.672087	3.063688	0.678689	1.680338	3.068733	0.615509	1.732178
C	-4.150560	1.677449	0.025003	4.165461	1.633538	0.015949	4.141001	1.640947	0.106346
H	-3.681556	1.900519	0.993491	3.721579	1.874781	-0.959810	3.684562	1.927593	-0.839952
H	-4.077886	2.582647	-0.593921	4.098092	2.534462	0.641192	4.091480	2.504025	0.769796
C	-5.606736	1.190331	0.192719	5.614181	1.117672	-0.123708	5.582716	1.124013	-0.082487
H	-6.337847	2.003815	0.295373	6.364894	1.915991	-0.207132	6.330782	1.914941	-0.140748
C	-5.801620	0.274689	-1.055063	5.764735	0.193965	1.123791	5.749812	0.141854	1.112950
C	-4.548545	-0.619835	-0.707456	4.502775	-0.674062	0.747083	4.478062	-0.695438	0.719584
C	-4.849390	-1.037713	0.757842	4.828714	-1.095275	-0.711614	4.772403	-1.050310	-0.761781

H	-5.458798	-1.952180	0.770952	5.424245	-2.019316	-0.712641	5.357296	-1.968602	-0.818854
H	-3.923782	-1.264738	1.303144	3.910954	-1.305041	-1.275733	3.851387	-1.223206	-1.314140
C	-5.610440	0.180972	1.358967	5.622094	0.112054	-1.293765	5.563249	0.178116	-1.299373
H	-5.114111	0.592322	2.249983	5.146552	0.535969	-2.190272	5.077898	0.643395	-2.158708
H	-6.633296	-0.085762	1.657385	6.645822	-0.172206	-1.574738	6.571511	-0.096111	-1.609910
C	-5.712450	1.019923	-2.396694	5.661477	0.936674	2.465624	5.681571	0.818986	2.491136
H	-4.835723	1.673164	-2.496519	4.791161	1.600156	2.548361	4.823778	1.475024	2.628156
H	-6.601953	1.653595	-2.529495	6.558117	1.557155	2.615862	6.577625	1.424174	2.647218
H	-5.694857	0.308249	-3.235960	5.616086	0.221084	3.300718	5.652661	0.068472	3.284280
C	-7.124005	-0.503787	-1.059115	7.071852	-0.609694	1.152971	7.054138	-0.666478	1.083091
H	-7.970382	0.197723	-1.109915	7.929610	0.075860	1.231780	7.908802	0.008985	1.169137
H	-7.270342	-1.135176	-0.174506	7.225756	-1.234999	0.265147	7.188750	-1.254187	0.177675
H	-7.187641	-1.153266	-1.945050	7.098521	-1.269746	2.033340	7.094639	-1.354773	1.930298
C	-4.241691	-1.774947	-1.643238	4.152433	-1.825491	1.672170	4.134083	-1.885820	1.593517
H	-3.385919	-2.364494	-1.281546	3.290082	-2.392419	1.291151	3.261304	-2.415606	1.208659
H	-4.005010	-1.424853	-2.659972	3.901654	-1.471823	2.684192	3.915063	-1.580660	2.619548
H	-5.097474	-2.462093	-1.711418	4.992106	-2.531379	1.754277	4.960080	-2.599066	1.619721
C	3.415362	0.030043	0.444721	-3.390705	0.081634	-0.453733	-3.365434	0.097202	-0.441111
H	3.507669	1.092480	0.166622	-3.458790	1.121918	-0.096245	-3.432381	1.120798	-0.066124
H	3.012906	-0.507225	-0.427431	-2.975289	-0.522822	0.366796	-2.955431	-0.520958	0.358966
C	4.812247	-0.507389	0.808501	-4.806308	-0.403818	-0.820469	-4.776272	-0.381206	-0.822578
H	5.140007	0.036316	1.714428	-5.155133	0.229649	-1.655720	-5.116931	0.258284	-1.643960
C	4.819326	-2.009460	1.120494	-4.846852	-1.867317	-1.280497	-4.808008	-1.835284	-1.309244
H	5.826657	-2.329480	1.418102	-5.865794	-2.137806	-1.589336	-5.816985	-2.101203	-1.626352
H	4.531023	-2.601191	0.239602	-4.550188	-2.551161	-0.472491	-4.515107	-2.526167	-0.517484
H	4.126030	-2.260503	1.936555	-4.173560	-2.039902	-2.132084	-4.133688	-1.987368	-2.153057
C	5.778078	-0.131919	-0.304744	-5.730945	-0.135988	0.356604	-5.711401	-0.141997	0.350014
C	5.665195	-0.702979	-1.582465	-5.578657	-0.828749	1.569218	-5.556472	-0.843730	1.549630
H	4.891228	-1.448162	-1.785067	-4.798446	-1.587336	1.672296	-4.769845	-1.582700	1.648443
C	6.543227	-0.339974	-2.605937	-6.420104	-0.565721	2.653045	-6.406258	-0.611171	2.625740
H	6.444884	-0.796273	-3.592718	-6.288182	-1.114962	3.587479	-6.271970	-1.165361	3.546720
C	7.550303	0.599868	-2.366631	-7.431381	0.394565	2.541824	-7.429344	0.328026	2.519958
H	8.239601	0.879815	-3.164992	-8.090659	0.597812	3.387761	-8.092790	0.507430	3.356839
C	7.672901	1.173940	-1.099136	-7.593197	1.089758	1.340223	-7.593787	1.031975	1.331449
H	8.460041	1.904011	-0.902076	-8.381178	1.839228	1.242578	-8.387705	1.762919	1.238168
C	6.790224	0.810799	-0.078101	-6.746725	0.825829	0.258731	-6.738674	0.798059	0.257393
H	6.895042	1.260544	0.913146	-6.878287	1.372669	-0.678802	-6.874290	1.351248	-0.665240
C	0.196937	-0.200678	3.115423	-0.218295	-0.036206	-3.184972	-0.185733	0.025524	-3.153299
H	0.573558	0.621746	3.739046	-0.587268	0.823170	-3.760663	-0.553771	0.884960	-3.713107
H	0.507329	-1.163963	3.541842	-0.552827	-0.969776	-3.655435	-0.509147	-0.893911	-3.639888
H	-0.898250	-0.149342	3.068553	0.877466	-0.004624	-3.145659	0.901177	0.062006	-3.104459
